# H-NS Facilitates Sequence Diversification of Horizontally Transferred DNAs during Their Integration in Host Chromosomes

**DOI:** 10.1371/journal.pgen.1005796

**Published:** 2016-01-20

**Authors:** Koichi Higashi, Toru Tobe, Akinori Kanai, Ebru Uyar, Shu Ishikawa, Yutaka Suzuki, Naotake Ogasawara, Ken Kurokawa, Taku Oshima

**Affiliations:** 1 Department of Biological Information, Graduate School of Bioscience and Biotechnology, Tokyo Institute of Technology, Meguro-ku, Tokyo, Japan; 2 Department of Biomedical Informatics, Graduate School of Medicine, Osaka University, Suita, Osaka, Japan; 3 Department of Medical Genome Sciences, Graduate School of Frontier Sciences, The University of Tokyo, Kashiwa-shi, Chiba, Japan; 4 Graduate School of Biological Sciences, Nara Institute of Science and Technology, Nara, Japan; 5 Earth-Life Science Institute, Tokyo Institute of Technology, Meguro-ku, Tokyo, Japan; University of Edinburgh, UNITED KINGDOM

## Abstract

Bacteria can acquire new traits through horizontal gene transfer. Inappropriate expression of transferred genes, however, can disrupt the physiology of the host bacteria. To reduce this risk, *Escherichia coli* expresses the nucleoid-associated protein, H-NS, which preferentially binds to horizontally transferred genes to control their expression. Once expression is optimized, the horizontally transferred genes may actually contribute to *E*. *coli* survival in new habitats. Therefore, we investigated whether and how H-NS contributes to this optimization process. A comparison of H-NS binding profiles on common chromosomal segments of three *E*. *coli* strains belonging to different phylogenetic groups indicated that the positions of H-NS-bound regions have been conserved in *E*. *coli* strains. The sequences of the H-NS-bound regions appear to have diverged more so than H-NS-unbound regions only when H-NS-bound regions are located upstream or in coding regions of genes. Because these regions generally contain regulatory elements for gene expression, sequence divergence in these regions may be associated with alteration of gene expression. Indeed, nucleotide substitutions in H-NS-bound regions of the *ybdO* promoter and coding regions have diversified the potential for H-NS-independent negative regulation among *E*. *coli* strains. The *ybdO* expression in these strains was still negatively regulated by H-NS, which reduced the effect of H-NS-independent regulation under normal growth conditions. Hence, we propose that, during *E*. *coli* evolution, the conservation of H-NS binding sites resulted in the diversification of the regulation of horizontally transferred genes, which may have facilitated *E*. *coli* adaptation to new ecological niches.

## Author Summary

Horizontal gene transfer among bacteria is the major means of acquiring genetic diversity and has been a central factor in bacterial evolution. The expression of horizontally transferred genes could potentially be optimized to permit the host bacteria to expand their habitat. The results of our study suggest that DNA regions bound by the nucleoid-associated protein, H-NS, which preferentially binds to horizontally transferred genes, have been conserved during *Escherichia coli* evolution. Interestingly, H-NS-bound regions have evolved faster than H-NS-unbound regions, but only in gene regulatory and coding regions. We show that DNA sequence substitutions in H-NS-bound regions actually alter the regulation of gene expression in different *E*. *coli* strains. Thus, our results support the hypothesis that H-NS accelerates the diversification of the regulation of horizontally transferred genes such that their selective expression could potentially allow *E*. *coli* strains to adapt to new habitats.

## Introduction

The *Escherichia coli* species consists of genetically diverse strains, for example, in terms of nutrient metabolism, stress responses, and pathogenicity [1]. One of the well-known factors causing genetic diversity in bacteria is horizontal gene transfer; an estimated 10–16% of genes in *E*. *coli* strains have been acquired horizontally [2]. However, unregulated expression of newly acquired genes could disrupt the physiology of the host cell [3,4]. Both *E*. *coli* and *Salmonella* express the protein H-NS, which preferentially binds adenine and thymine (AT)-rich DNA [3,5–7]. Many horizontally transferred genes (HTGs) have a high AT content relative to *E*. *coli* genes, which facilitates H-NS binding to, and repression of, the foreign genes [8]. This repression guards host cells from potential physiological perturbations caused by expression of HTGs [3,5].

Deficiency in the gene *hns* impairs *Salmonella* growth during laboratory cultivation [9]. Compensatory mutations for this growth impairment have been identified in the gene *stpA*, encoding StpA, which is the H-NS paralog. These mutations alter StpA functionality to resemble that of H-NS [9]. In addition, loss of virulence genes in the *Salmonella* pathogenic island-1 (SPI-1) and frameshift and missense mutations in *phoPQ*, which encodes the positive transcriptional regulator of virulence genes, could also compensate for the fitness loss of *hns* deficiency [9]. Therefore, the major role of H-NS in *Salmonella* is purportedly the silencing of genes within SPI-1 [9].

In addition, H-NS suppresses transcription of pervasive non-coding and antisense sequences in both coding regions and intergenic regions [10–12] by inhibiting the recruitment of RNA polymerase to promoters, trapping this polymerase at promoters, or inhibiting transcriptional elongation [8,10,11,13–17]. However, AT-rich sequences bound by H-NS can be highly expressed when both *hns* and *stpA* are disrupted [18]. In this scenario, the spurious expression of non-coding and antisense RNAs and the higher expression of AT-rich genes impose high metabolic costs and reduce the fitness of *hns*-deficient cells [11,18].

Furthermore, H-NS can both directly and indirectly regulate global gene expression in *E*. *coli* [19,20]. Mutations that counter the slow growth observed for the *hns/stpA* double mutant have been identified. One mutation inactivates the sigma factor for stress response, namely RpoS, which is involved in the expression of many genes induced by the *hns/stpA* double mutation. The other mutation amplifies ~40% of the *E*. *coli* chromosome centered near the origin of replication, which causes remodeling of the transcriptome and partially reverses the imbalance in global gene expression caused by the double mutation [21]. Interestingly, the transcriptional repression activity of H-NS is affected by the location of H-NS binding sites throughout the *E*. *coli* chromosome. H-NS is a strong repressor of the *hns* promoter when this promoter is ectopically placed in the Ter or Left macrodomain of the chromosome [22]. It is also known that environmental factors, such as pH, temperature, and osmolarity, can alter H-NS-mediated gene repression [23]. Hence, a change in environmental conditions, i.e., an abiotic stressor, can activate a large number of genes that normally are repressed by H-NS, thereby potentiating the stress response. [5,23].

Any HTG should be expressed only when its function is beneficial to the host bacteria. However, transcriptional regulators are not well conserved and transcriptional networks are highly diversified among bacterial species [24]. For acquired genes, therefore, the regulation that occurs via a host-cell transcriptional regulator(s) and/or regulatory element(s) would need to be optimized [25]. It has been suggested that, in bacteria, such optimization requires a long time, and this is accomplished through several steps: 1) upon integration of the HTG(s) into the host genome, the initial expression would be lower than for native host genes; 2) a host-cell activator is required to express HTGs; and 3) the expression of the transferred genes must be fine-tuned to match the needs of host cells [25]. On the other hand, Dorman [3] proposed that H-NS-mediated repression of HTGs could be an effective way to reduce the risk of inappropriate expression of such genes until expression could be optimized. Although H-NS-mediated repression of virulence genes, which are HTGs, may reduce the fitness cost raised by the expression of virulence genes and contributes to the evolution of the *Salmonella* [9], it remains unclear whether H-NS actually contributes to the optimization of expression of transferred genes so as to benefit host cells.

The aim of our study was to improve our knowledge of how H-NS contributes to the integration of HTGs into *E*. *coli*. Genome-wide H-NS binding profiles were recently obtained with the *E*. *coli* K-12 genome using chromatin immunoprecipitation (ChIP)-chip and ChIP-seq analyses [19,26–28]. Using this information, it is possible to examine the conservation/diversification of H-NS-bound regions within the *E*. *coli* genome during evolution. Hence, we used chromatin affinity precipitation (ChAP)-seq to compare H-NS-bound regions within the genomes of genetically diverse *E*. *coli* strains belonging to different subgroups, specifically, laboratory strain K-12 (subgroup A), commensal strain SE11 (subgroup B1), and commensal strain SE15 (subgroup B2) [29,30]. This analysis enabled us to investigate the influence of H-NS binding on the diversification of genomic sequences.

Our analysis suggests that the distribution of H-NS-bound regions within *E*. *coli* genomes has been highly conserved during evolution. In addition, sequence diversity in the H-NS-bound regulatory regions tended to be greater than in H-NS-unbound regulatory regions. Hence, we propose that transcriptional repression by H-NS increases the propensity for nucleotide substitutions in transcriptional regulatory regions of HTGs, which may alter the expression of transferred genes to facilitate adaptation of *E*. *coli* cells to new habitats.

## Results

### ChAP-seq analysis of H-NS-bound regions in three *E*. *coli* strains

Phylogenetic analysis has indicated that group B2 is the ancestral phylogroup in the *E*. *coli* lineage, whereas groups A and B1 have diverged [31–33]. To assess the impact of *E*. *coli* evolution on H-NS binding, we comprehensively compared the localization of H-NS-bound regions on chromosomes among the three *E*. *coli* strains K-12 (group A), SE11 (group B1), and SE15 (group B2). Notably, the amino acid sequence of H-NS is completely conserved among these strains.

**Fig 1:**
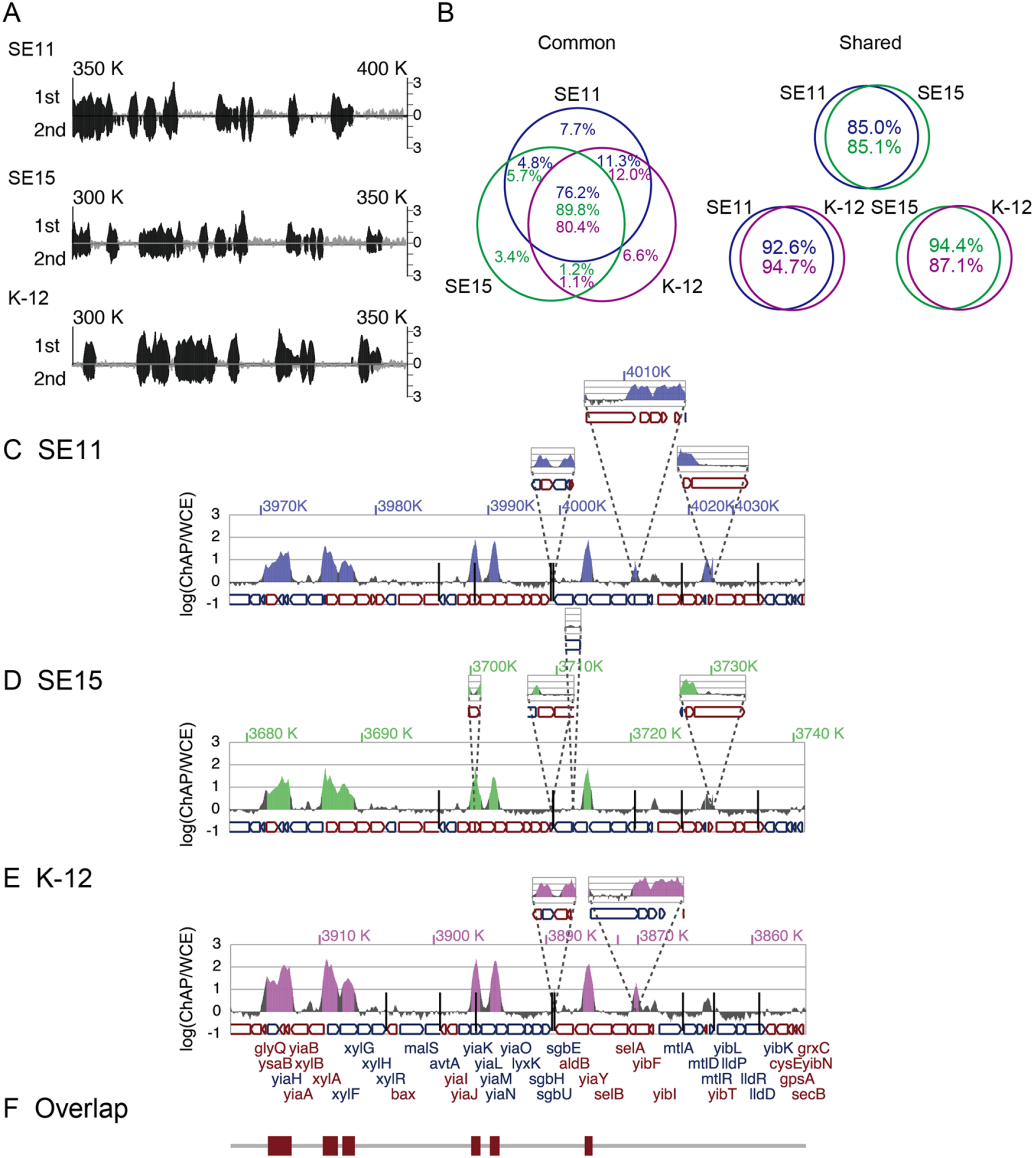
Overlap of H-NS binding regions in the three *E*. *coli* strains. (A) H-NS binding profiles in biological duplicates (1st and 2nd experiment) along the reference genome in the 300K–350K region of *E*. *coli* strain K-12 and corresponding regions of stains SE11 (350K–400K) and SE15 (300K–350K). Signal intensity of H-NS binding in the 1st experiment is indicated by upward bars, while that in the 2nd experiment is indicated by downward bars. (B) Venn diagram of H-NS binding regions in the “common” and “shared” segments of the three strains. Percentages in the diagram indicate the proportions of conserved, shared, and unique H-NS binding sequences relative to the total length of H-NS binding regions in the “common” (left panel) and “shared” (right panel) segments of each genome (blue, green, and purple indicate percentages in the SE11, SE15, and K-12 genomes, respectively). (C–E) Typical H-NS binding profiles for “common” segments; the *glyS* (3,918 kb) through *yibN* (3,855 kb) region (according to K-12 genome annotation) of SE11 (C), SE15 (D), and K-12 (E) genomes. At the bottom of panel E, the arrangement of coding sequences (CDSs) is shown after connecting “common” segments. The CDS color indicates the direction of translation: red, clockwise; blue, counter clockwise. Shared and specific segments larger than 500 bp are superimposed at the corresponding positions on the connected “common” segments. Positions of smaller shared and specific segments (<500 bp) are indicated by vertical bars. H-NS binding intensities [ChAP / WCE (log_10_)] at every nucleotide, determined in one of the ChAP-seq experiments performed duplicate (1st experiment), are presented on the vertical axis. Defined H-NS binding regions in each strain are depicted as colored areas in the H-NS binding peaks. (F) Conserved H-NS-bound regions among the three strains are shown as brown rectangles.

We created H-NS-12His-expressing recombinant K-12 (W3110), SE11, and SE15 strains and determined H-NS binding profiles on the chromosomes for the three strains using ChAP-seq. Each strain was grown to mid-log phase (OD_600_ ≈ 0.4) in LB medium under aerobic condition and treated with formaldehyde to crosslink H-NS-12His to DNA, followed by ChAP of the crosslinked DNA fragments with H-NS, as described [34]. Purified DNA from ChAP and whole-cell extract (WCE; pre-ChAP) was subjected to high-throughput Illumina sequencing, and the H-NS-bound regions were determined (See details in Materials and Methods). We performed duplicate ChAP analyses for each strain, and the H-NS binding profiles were highly reproducible (Fig 1A). Thus, we defined overlapping regions of H-NS binding regions in duplicate ChAP analyses as reproducible H-NS binding regions, and used these defined regions in subsequent analyses. We identified H-NS-bound regions covering 802,561 bp in SE11, 642,859 bp in SE15, and 697,762 bp in K-12, corresponding to 14–16% of each genome (Table 1).

**Table 1:** Summary of the comparative analysis of the three *E*. *coli* strains.

	SE11	SE15	K-12
Length of genome (bp)	4,887,515	4,717,338	4,646,332
Total length of "common" segments (bp)^a^	3,886,369	3,886,157	3,886,242
Total length of "shared" segments between SE11 and K-12 (bp)	392,082	-	391,987
Total length of "shared" segments between SE15 and SE11 (bp)	52,289	52,238	-
Total length of "shared" segments between SE15 and K-12 (bp)	-	87,649	87,582
Total length of "specific" segments (bp)	556,775	691,294	280,521
Total length of H-NS-bound regions (bp)	802,561	642,859	697,762
Number of H-NS-bound regions	506	510	436
Proportion of H-NS-bound regions in total genome sequences (%)	16.4	13.6	15.0
Total length of H-NS-bound regions in "common" segments (bp)	451,643	383,226	427,731
Proportion of H-NS-bound regions in "common" segments (%)	11.6	9.9	11.0
Total length of H-NS-bound regions in "shared" segments (bp)	170,778	50,917	181,704
Proportion of H-NS-bound regions in "shared" segments (%)	38.4	36.4	37.9
Total length of H-NS-bound regions in "specific" sequences (bp)	180,140	208,716	88,327
Proportion of H-NS-bound regions in "specific" sequences (%)	32.4	30.2	31.5

^a^: Total length of consensus sequences of “common” segments is 3,888,365 bp.

### Comparison of H-NS binding profiles from the three *E*. *coli* strains

To compare the H-NS-bound regions among the *E*. *coli* strains, we aligned the three chromosome sequences using the Mauve program developed for the multiple alignment of bacterial chromosome sequences [35,36]. We identified the “common” (conserved in all three strains), “shared” (conserved in two strains), and “specific” (unique for each strain) chromosome segments (Table 1). Whereas the common segments would have been in the ancestral genome before divergence of the *E*. *coli* lineage, the “shared” and “specific” segments would have become integrated in the *E*. *coli* genome after the divergence. We calculated the proportions of H-NS-bound regions in each of the “common”, “shared”, and “specific” segments of the three strains (Table 1). The proportion of H-NS-bound sequences was higher in the “specific” and “shared” segments (~30–38%) than in the “common” segments (~10–12%), suggesting that many genes in the “specific” and “shared” regions were horizontally transferred during *E*. *coli* evolution and retained preferential binding to H-NS.

### H-NS-bound regions on “common” genome segments are conserved in the *E*. *coli* strains

Although many of the “specific” and “shared” segments were bound by H-NS, more than half of the H-NS-bound regions were located within “common” segments, with similar total length among the chromosomes of SE11 (451,643 bp), SE15 (383,226 bp), and K-12 (427,731 bp) (Table 1).

Specifically, 76.2% (SE11), 89.8% (SE15), and 80.4% (K-12) of H-NS-bound regions in “common” segments overlapped among the three strains (Fig 1B left [Common] and Fig 1C–1F). In addition, very few H-NS-bound regions in “common” segments (3.4% to 7.7%) were identified as unique in each strain, and the remainder of the binding regions were shared by two strains (Fig 1B left [Common]). We manually examined these unique and shared H-NS-bound regions and found that most of these regions (84% of the unique and shared H-NS-bound regions in common segments) had H-NS binding signals on a certain level in all three strains, although signal intensities were below the threshold to be categorized as H-NS-bound regions in one or two strains. We concluded that the H-NS-bound regions in “common” segments are highly conserved in the three strains. It has been reported that H-NS binding to orthologous genes in *E*. *coli* and *Salmonella* is highly conserved [19]. This and our current result indicate that the H-NS-bound regions have been retained in the *E*. *coli* lineage during evolution. Notably, the H-NS-bound regions within “shared” segments between two strains are also conserved (85.0–94.7%, Fig 1B right [Shared]).

### Non-synonymous sites in H-NS-bound genes evolve faster than those in H-NS-unbound genes

We concluded that the H-NS binding in “common” segments has been conserved during the evolution of *E*. *coli*. Therefore, we were interested in the effects of the conservation of H-NS binding on sequence diversification/conservation among the *E*. *coli* genomes. We initially compared sequence diversities between the H-NS-bound and -unbound orthologous genes. OrthoMCL was used to search for conserved orthologs that are present in SE15, SE11, and K-12 and at least 37 other *E*. *coli* strains, of the 44 strains in the curated non-redundant genome collection of reference sequences (RefSeq) at NCBI, when we started this analysis [37] (See details in Materials and Methods and S1 Fig). Then, 2,702 genes were selected as being well conserved orthologs (S2 Table), and these were used to estimate the synonymous (dS) and non-synonymous (dN) substitution rates based on multiple sequence alignment. Genes among these were defined as H-NS bound if their coding regions overlapped with H-NS-bound regions identified in at least one of the SE15, SE11, and K-12 strains as determined by ChAP-seq analysis.

**Fig 2:**
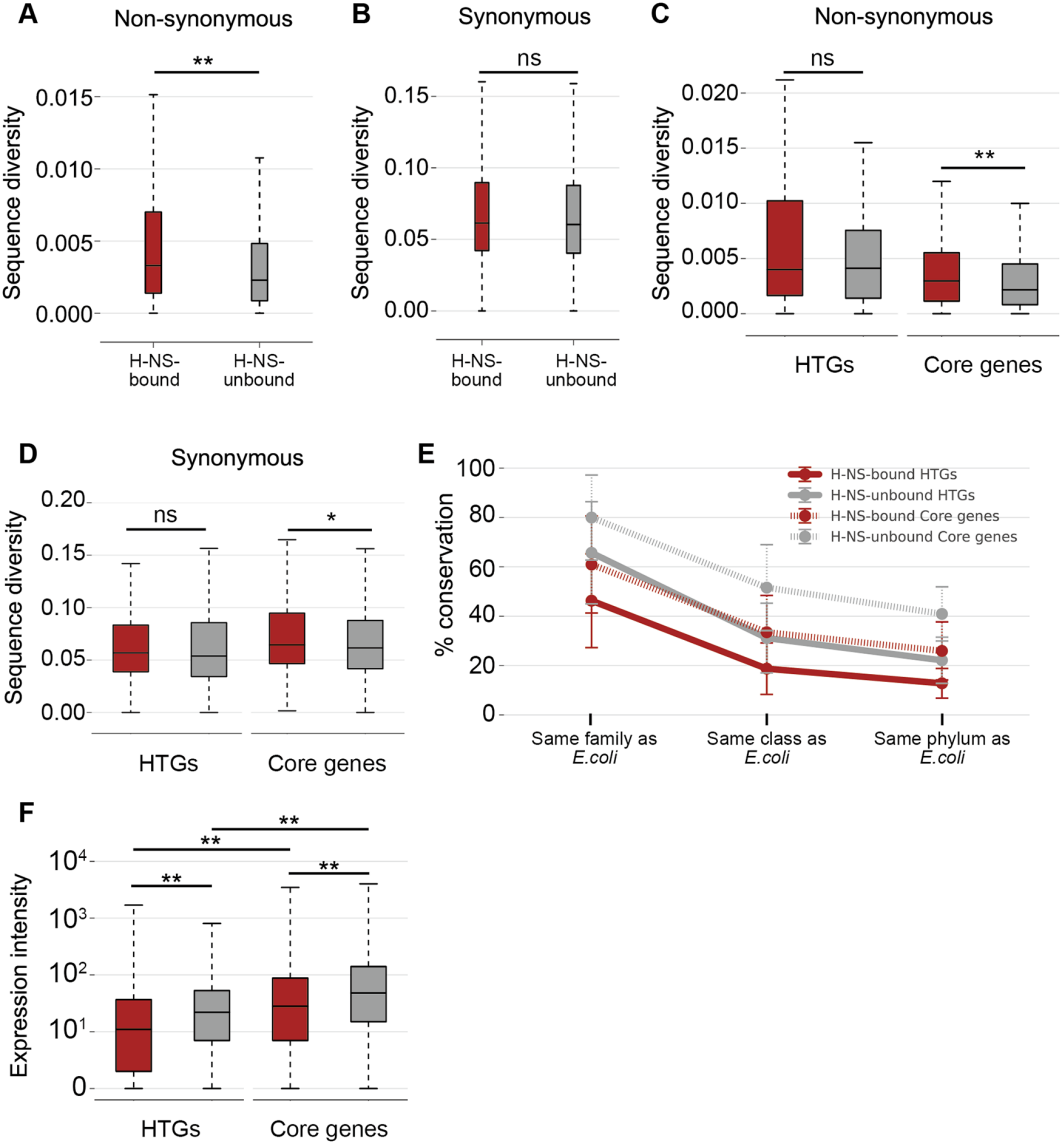
Comparison of sequence diversities of H-NS-bound and -unbound orthologous genes. Diversity of dN and dS for each orthologous gene cluster was computed by averaging all pairwise evolutionary distances of orthologous genes in *E*. *coli* strains. (A–D) Box plots represent the distribution of sequence diversity; shown are the median (horizontal black lines in boxes), the upper and lower quartile values (boxes), and the most extreme data points within 1.5× of the interquartile range (whiskers). (A) Distribution of dN in the H-NS-bound (red; N = 519, median value = 0.0033) and -unbound (gray; N = 2,183, median value = 0.0023) genes. (B) Distribution of dS in the H-NS-bound (red; N = 519, median value = 0.061) and -unbound (gray; N = 2,183, median value = 0.060) genes. (C) Distribution of dN in the H-NS-bound HTGs (red; N = 230, median value = 0.0040), the H-NS-unbound HTGs (gray; N = 271, median value = 0.0041), the H-NS-bound Core genes (red; N = 289, median value = 0.0030), and the H-NS-unbound Core genes (gray; N = 1,912, median value = 0.0022). (D) Distribution of dS in the H-NS-bound HTGs (red; N = 230, median value = 0.057), the H-NS-unbound HTGs (gray; N = 271, median value = 0.054), the H-NS-bound Core genes (red; N = 289, median value = 0.065) and the H-NS-unbound Core genes (gray; N = 1,912, median value = 0.062). (E) Conservation of each class of genes averaged by species belonging to the same family (Enterobacteriaceae) but different genus, in the same class (Gammaproteobacteria) but different family, or in the same phylum (Proteobacteria) but different class as *E*. *coli*. Error bars denote standard deviation. Red solid line, H-NS-bound HTGs; gray solid line, H-NS-unbound HTGs, red dotted line, H-NS-bound Core genes; gray dotted line, H-NS-unbound Core genes. (F) Distribution of transcription level of the H-NS-bound HTGs (red; N = 230, median value = 10), the H-NS-unbound HTGs (gray; N = 270, median value = 21), the H-NS-bound Core genes (red; N = 289, median value = 27), and the H-NS-unbound Core genes (gray; N = 1,912, median value = 47). The transcription level of each gene in *E*. *coli* K-12 was acquired from RNA-seq data [47] deposited with accession number GSE21341. The gene *phnE* is missing in the RNA-seq data, and thus we ignored *phnE* in this analysis. Asterisks indicate the statistical significance of differences in sequence diversity between the H-NS-bound and -unbound genes as assessed with the Wilcoxon rank-sum test (**p < 0.001, *p < 0.05, ns: not significant).

As expected, dS was higher than dN for the orthologous genes regardless of H-NS binding (see sequence diversity scales of Fig 2A and 2B), whereas dN in the H-NS-bound genes tended to be higher than that in the H-NS-unbound genes (Fig 2A; *p* < 0.001, Wilcoxon rank-sum test). In contrast, the dS between H-NS-bound and -unbound genes was not significantly different (Fig 2B; *p* = 0.08). These observations indicated that the non-synonymous sites in the H-NS-bound genes evolved faster than those in the H-NS-unbound genes. Because H-NS preferentially binds to horizontally transferred genes (HTGs) [3,6,7,27], this apparent faster evolution of non-synonymous sites in H-NS-bound genes could simply reflect the rapid evolution of genes recently transferred to host cells, which was indicated in the *Bacillus cereus* group [38] and *E*. *coli* lineages [39]. To assess the effect of H-NS binding and horizontal transfer, orthologous genes were classified into HTGs which were estimated as HTGs in at least one of previous predictions [40–42] or Core genes (other non-HTGs), and the tendency of dS and dN in each class of H-NS-bound and—unbound genes was evaluated. The dN of HTGs with or without H-NS binding was greater than that of Core genes (Fig 2C), which is consistent with previous observations [38,39]. In addition, dN of H-NS-bound Core genes was greater than that of H-NS-unbound Core genes (Fig 2C Core genes; *p* < 0.001). Furthermore, dS of H-NS-bound Core genes was also greater than that of H-NS-unbound Core genes; this difference in dS was smaller than that of dN, but statistically significant (Fig 2D Core genes; *p* = 0.0072). These results indicated that the non-synonymous and synonymous sites in H-NS-bound Core genes evolve faster than those in H-NS-unbound Core genes in the *E*. *coli* lineage. In contrast, dN of H-NS-bound and -unbound HTGs indicated no significant difference (Fig 2C HTGs; *p* = 0.097). However, the variance of dN of H-NS-bound HTGs and that of H-NS-unbound HTGs were significantly different (Fig 2C HTGs; *p* = 0.010, Levene's test). As shown in Fig 2C, the 75th percentile of dN for H-NS-bound HTGs was shifted upward compared with that for H-NS-unbound HTGs (Fig 2C HTGs; compare the height of the upper edges in boxes and whiskers for H-NS-bound [red] and -unbound HTGs [gray]), suggesting that dN of a certain fraction of H-NS-bound HTGs tended to be greater than that of H-NS-unbound HTGs. These results suggested that the observed larger dN for H-NS-bound regions did not result only from the tendency of HTGs to evolve rapidly.

### H-NS-bound Core genes may have been horizontally transferred in ancient ancestors of *E*. *coli*

To characterize H-NS-bound Core genes, we investigated the conservation of each class of genes in proteobacteria classified into the same family, the same class, or the same phylum with *E*. *coli*, using the ortholog table acquired from the Microbial Genome Database for Comparative Analysis (MBGD) [43–46]. The results indicated that H-NS-bound Core genes have been less conserved in proteobacteria than H-NS-unbound Core genes, but more conserved than H-NS-bound HTGs (Fig 2E). This result suggested that H-NS-bound Core genes were acquired by ancient ancestors of *E*. *coli*. In contrast, the conservation of H-NS-bound HTGs was lowest in bacteria belonging to the same family as *E*. *coli*, suggesting that the genes were more recently acquired by ancestors of *E*. *coli*. To evaluate whether the adaptation of H-NS-bound Core genes to host cells could be assessed based on gene expression level, quantitative RNA-seq data [47] were analyzed (Fig 2F). This analysis revealed that the expression of both H-NS-bound and -unbound Core genes was greater than that of H-NS-bound and -unbound HTGs, respectively (Fig 2F; *p* < 0.001). This suggested that H-NS-bound Core genes have adapted to host cells. However, the expression level of H-NS-bound Core genes tended to be lower than that of H-NS-unbound Core genes (Fig 2F; *p* < 0.001). Interestingly, the analysis indicated that cellular protein level, rather than functional category, essentiality, or metabolic cost of a protein’s amino acid composition, has been the principal driving force constraining non-synonymous substitutions [48]. Therefore, one possible explanation for the tendency of a higher dN in the H-NS-bound Core genes than in H-NS-unbound Core genes might be the H-NS-mediated transcriptional repression of H-NS-bound Core genes.

### The H-NS-bound intergenic regions evolve faster than the H-NS-unbound intergenic regions

**Fig 3:**
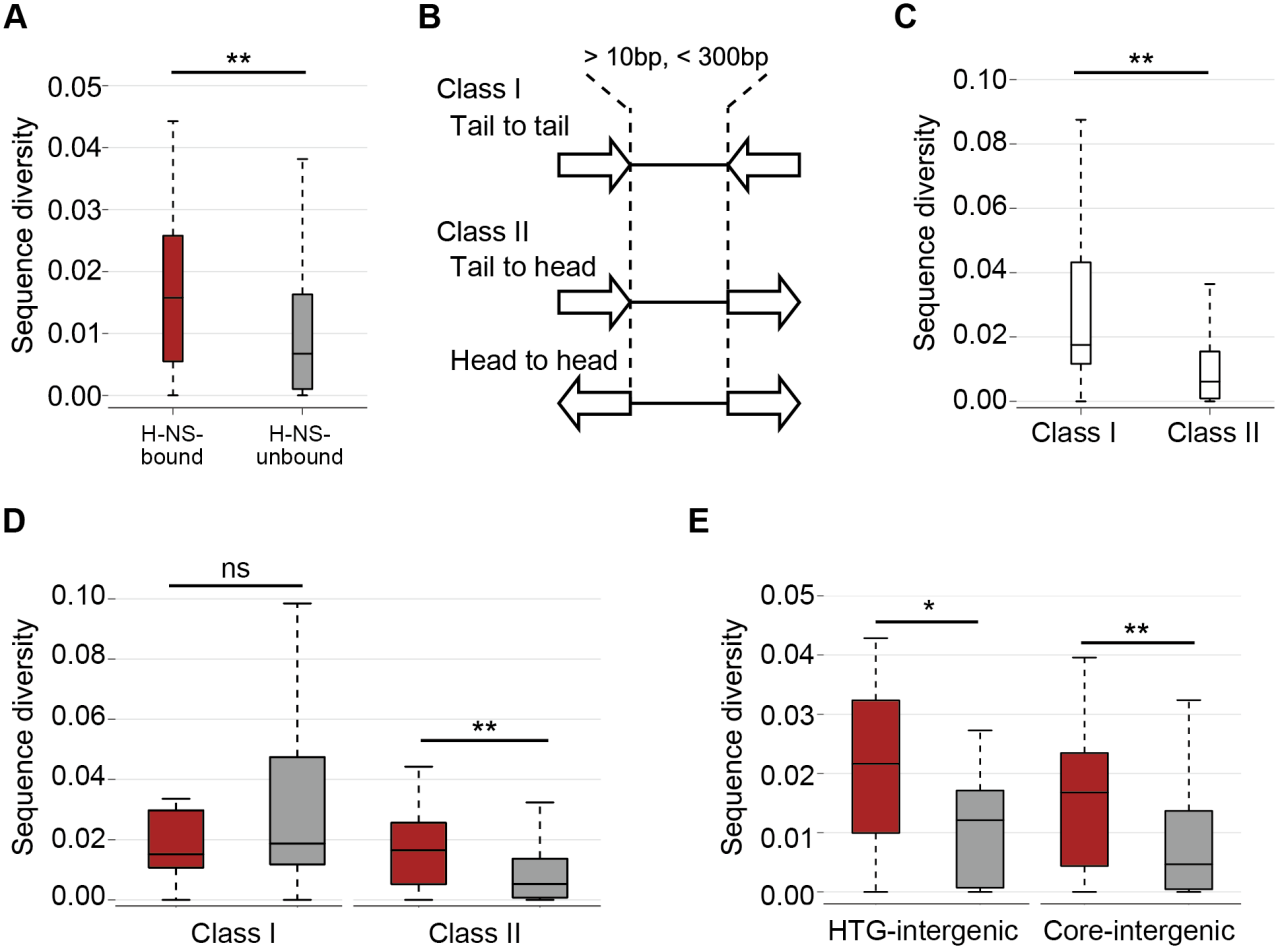
Comparison of sequence diversity between H-NS-bound and -unbound intergenic regions located upstream or downstream of genes. The diversity of each conserved intergenic region cluster was computed by averaging all pairwise evolutionary distances of the conserved intergenic regions in *E*. *coli* strains. Box plots are shown in the same manner as in Fig 2: red, H-NS-bound intergenic regions; gray, H-NS-unbound intergenic regions. (A) Distribution of sequence diversity in the H-NS-bound (red; N = 94, median value = 0.016) and -unbound (gray; N = 609, median value = 0.0067) conserved intergenic regions. (B) Schematic view and definitions of the classes and subclasses of conserved intergenic regions. (C) Sequence diversity of class I (N = 92, median value = 0.018) and class II (N = 611, median value = 0.0061) intergenic regions. (D) Sequence diversity of H-NS-bound (N = 14, median value = 0.015) and -unbound (N = 78, median value = 0.019) class I intergenic regions (left) and of H-NS-bound (N = 80, median value = 0.017) and -unbound (N = 531, median value = 0.0052) class II intergenic regions (right). (E) Sequence diversity of H-NS-bound (N = 24, median value = 0.022) and—unbound (N = 16, median value = 0.012) class II HTG-intergenic regions (left); H-NS-bound (N = 28, median value = 0.017) and -unbound (N = 411, median value = 0.0047) class II Core-intergenic regions (right). The asterisks indicate the statistical significance of the difference between the sequence diversity in the H-NS-bound and -unbound intergenic regions as assessed with the Wilcoxon rank-sum test (**p < 0.001, *p < 0.05, ns: not significant).

To investigate the relationship between H-NS binding and the evolution of the intergenic regions, we compared sequence diversity between the H-NS-bound and -unbound intergenic regions. To avoid spurious alignments of the intergenic regions caused by recombination, insertion, or deletion, we selected the “conserved” intergenic regions, i.e., those that were 10–300 bp and were located between two neighbouring orthologous genes in *E*. *coli* strains. In addition, after the multiple alignment of each conserved intergenic region, if there was a difference of ≥ 10% in the length of the aligned sequence with at least one strain, the region was considered as a region with an insertion/deletion and it was removed from the set of “conserved” intergenic regions. Furthermore, after the likelihood phylogenetic analysis, the intergenic regions that showed too large an evolutionary distance for accurate alignment (evolutionary distance > 1.0) were removed from the analysis. Ultimately, 703 intergenic regions, which included 94 H-NS-bound intergenic regions, were selected for the purpose of calculating sequence diversity (S3 Table). The results indicated that sequence diversity in H-NS-bound intergenic regions tended to be higher than in H-NS-unbound intergenic regions (Fig 3A; *p* < 0.001), suggesting that the H-NS-bound intergenic regions have evolved faster than the H-NS-unbound intergenic regions.

### Greater sequence diversity of H-NS-bound intergenic regions is observed only in intergenic regions upstream of genes

In general, H-NS functions as a transcriptional repressor in *E*. *coli* [8]. We investigated whether the higher sequence diversification in the H-NS-bound intergenic regions is related to the regulation of gene expression. We categorized the intergenic regions into two classes (Fig 3B) based on the assumption that the regulatory elements for transcription (i.e., promoters and binding sites of transcriptional regulators) are more frequently present upstream of genes than downstream of genes. Class I was defined as the region sandwiched between the tails (3’ ends) of two convergently transcribed genes, representing the non-regulatory intergenic region (Fig 3B); class II included two subtypes, namely the region sandwiched between the heads (5’ ends) of two divergently transcribed genes (head-to-head region) or that between the tail and the head of two genes (tail-to-head region), representing the regulatory intergenic regions (Fig 3B). Then, we compared the sequence diversification between the H-NS-bound and -unbound regions in each class.

The sequence diversity of the class I regions tended to be greater than that of the class II regions (Fig 3C; *p* < 0.001). In addition, there was no significant difference in sequence diversity between the H-NS-bound and -unbound class I regions (Fig 3D, class I; *p* = 0.29). In contrast, the sequence diversity in the H-NS-bound regions tended to be greater than in the H-NS-unbound regions within the class II regions (Fig 3D, class II; *p* < 0.001). These results suggested that the regulatory intergenic regions have evolved slower than non-regulatory intergenic regions, whereas the H-NS-bound regions have evolved faster than the H-NS-unbound regions among the regulatory intergenic regions. In addition, we extracted the horizontally transferred intergenic regions (HTG-intergenic) sandwiched by HTGs and core intergenic regions (Core-intergenic) sandwiched by Core genes, respectively, from the class II intergenic regions to evaluate any difference in the effects of H-NS binding on sequence diversification of HTG- and Core-intergenics. To avoid mixing the Core-intergenic and HTG-intergenic characteristics, which might have occurred in the intergenic regions between Core genes and HTGs, we used the intergenic regions that were uniquely sandwiched only by HTGs or Core genes, as “HTG-intergenic” or “Core-intergenic”, respectively. The sequence substitution rates for H-NS-bound HTG-intergenic were higher than that for H-NS-unbound HTG-intergenic (Fig 3E; *p* = 0.031). This tendency was also observed in Core-intergenics (Fig 3E; *p* < 0.001). We thus concluded that the higher sequence substitution rates of H-NS-bound class II intergenic regions could not be explained exclusively by the rapid adaptation of the regulatory regions of recent HTGs.

### Evaluation of the effects of sequence substitutions on transcriptional regulation in H-NS-bound regions

Our analysis indicated that the sequence substitution rate of H-NS-bound regulatory regions was higher than that of H-NS-unbound regulatory regions. We hypothesized that these sequence substitutions in H-NS-bound transcriptional regulatory regions could alter the expression of HTGs. To test this, we selected one of the H-NS-bound HTGs, namely *ybdO*, which has a large number of sequence substitutions in the upstream intergenic and coding regions (within the rank of top 50 for sequence substitution rate in the class II and coding regions, S1 Fig), and seems to be a single cistron in strains SE11, SE15 and K12. In addition, the H-NS binding profile encompassing the upstream and/or coding regions of *ybdO* was highly conserved among strains SE11, SE15, and K-12, suggesting that H-NS represses *ybdO* expression in these strains (S2A Fig). Thus, the effects of sequence substitutions within *ybdO* on its transcriptional regulation were examined.

**Fig 4:**
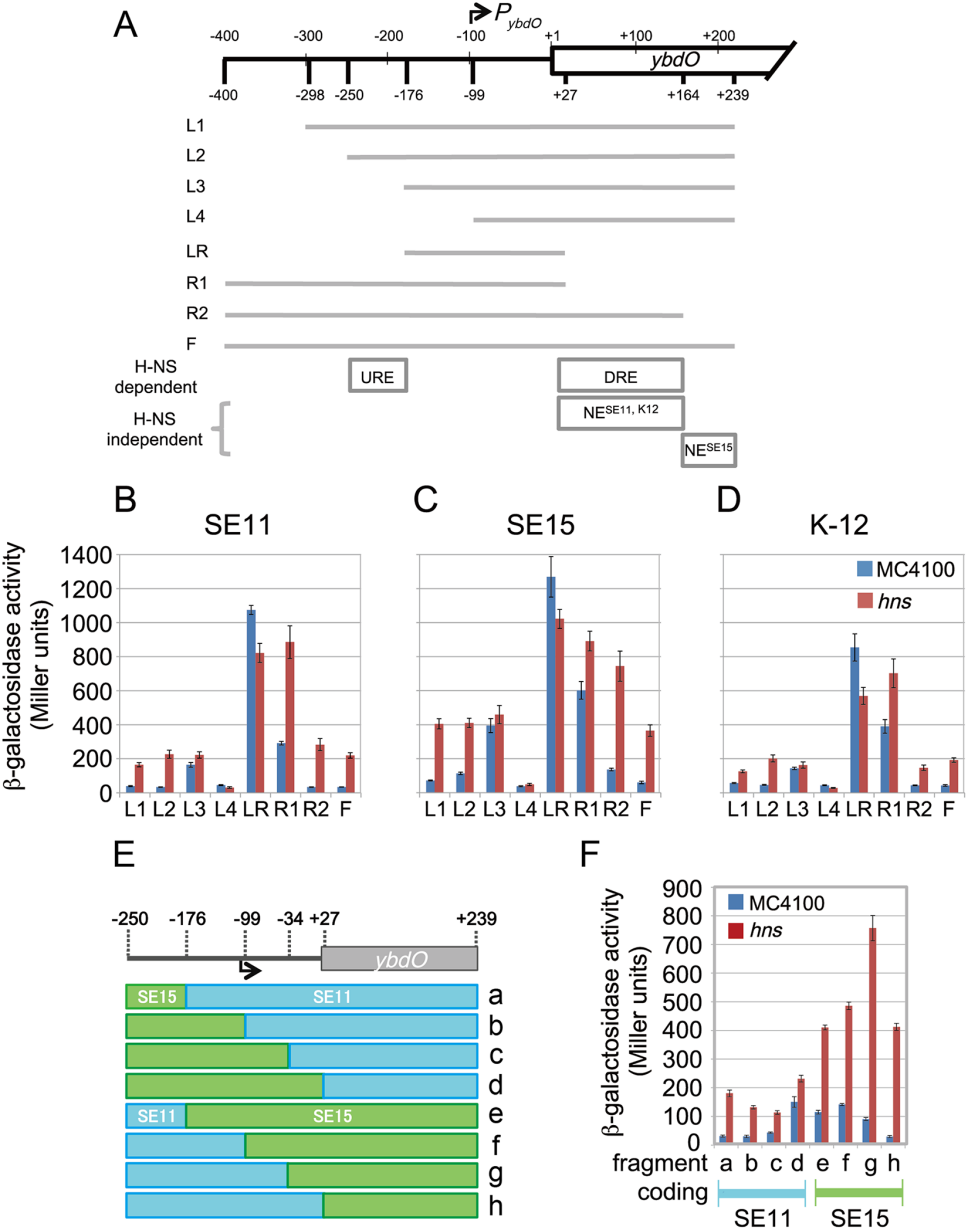
Comparison of the *ybdO* transcriptional activity in each of strains SE11, SE15, and K-12. (A) Schematic representation of various lengths of DNA fragments carrying the *ybdO* regulatory region used in this analysis. The locations of the 5’ and 3’ ends of fragments are indicated by the distances from the *ybdO* start codon (the base numbers correspond to the nucleotide positions in K-12) with the transcriptional start site (TSS) of *ybdO* shown by an arrow indicating *P*_*ybdO*_ (top). The regions carried in each fragment (L1–F) are shown as gray lines (middle). The locations of the H-NS-dependent regulatory elements, URE and DRE, and H-NS-independent negative regulatory elements, NE^SE11, K-12^ and NE^SE15^, are indicated (bottom). (B–D) Comparison of the activities of SE11, SE15, and K-12 *ybdO* promoters as measured by β-galactosidase activity. The activity for each of the wild type (MC4100: blue bars) and *hns* mutants (MC4100 Δ*hns*::km, *hns*: red bars) harboring pRW50 plasmids carrying the different *ybdO* promoter fragments (L1–F) of SE11 (B), SE15 (C), and K-12 (D) are indicated. The values represent the average of three independent assays. Standard errors are shown with error bars. (E) Schematic representation of hybrid fragments. Junction points are indicated above the upstream and coding regions of *ybdO* as nucleotide numbers relative to the initiation codon of *ybdO* in K-12. (F) Comparison of the transcriptional activities of the hybrid fragments as measured by β-galactosidase activity. β-galactosidase activity is shown for each of the wild type (MC4100: blue bars) and *hns* mutants (MC4100 Δ*hns*::km, *hns*: red bars) carrying recombinant pRW50 plasmids containing the different hybrid fragments. The name (a ~ h) of each hybrid fragment (indicated at the bottom of the graph) corresponds to the name of the hybrid fragment in Fig 4E (indicated on the right of Fig 4E). The fragments are classified at the bottom of the panel as the fragment containing SE11 coding regions (pale blue bar) or SE15 coding regions (pale green bar).

Although the transcription start site of *ybdO* in K-12 was recently identified [49] (Fig 4A), the transcriptional regulation of *ybdO* has not been thoroughly investigated. We, therefore, identified transcriptional regulatory elements for *ybdO*. We systematically constructed *ybdO*-*lac* operon fusions on the low-copy-number plasmid, pRW50 [50], by inserting DNA segments containing upstream intergenic regions and the 5'-proximal coding region of *ybdO* or its deleted derivatives (Fig 4A). The activities of the *ybdO* promoters from different *E*. *coli* strains were monitored using the recombinant pRW50 plasmids introduced into the *E*. *coli* K-12 wild-type and the *hns* mutant strains. The presence of the Shine-Dalgarno sequence for the *lac* operon on the plasmids implies that the β-galactosidase activity of transformants represented the transcriptional activity of the particular DNA segment inserted into pRW50.

First, we examined the β-galactosidase activity for *ybdO* promoters from SE11, SE15, and K-12 in cloned L2 fragments, which contained the region from –250 bp to +239 bp (Fig 4A, L2, nucleotide positions are relative to the first nucleotide of the initiation codon [+1] of K-12 *ybdO*) in growing cells. We found that transcription from the *ybdO* promoters was maximally induced from the early stationary phase in LB medium (S2B Fig). In addition, *ybdO* transcription in all strains was higher in the *hns* mutant cells compared with wild-type cells (S2B Fig), suggesting that H-NS repressed *ybdO* transcription in all strains. We also determined transcription start sites of *ybdO* in SE11 and SE15 during the early stationary phase using 5’-RACE as described in Materials and Methods. The 5’ end of SE11 and SE15 *ybdO* mRNAs was mapped at 1 bp downstream of the transcription start site of K-12 *ybdO* (S3A and S3B Fig), which localized at 107 bp upstream from the initiation codon of *ybdO* [49] (S3B Fig). The results suggested that the promoters of *ybdO* in the three strains overlap (S3B Fig; putative –10 element is indicated by a red line). We then looked closely at regions both upstream and downstream of the *ybdO* promoter, which revealed a number of sequence substitutions in the promoter proximal region among *E*. *coli* strains (S1B Fig).

We also determined the elements necessary for H-NS dependent repression by comparing the activities of *ybdO*-*lac* operon fusions with systematic deletions in the wild-type and *hns* mutant strains. The results indicated that deletions of two specific regions, namely upstream (from –250 to –176 bp) and downstream (from +27 to +164 bp) of the region of the genome surrounding the *ybdO* promoter in SE11, SE15, and K-12, enhanced β-galactosidase activity in the wild-type cells (Fig 4B–4D, compare blue bars of L2 and L3, R2 and R1). In addition, comparison of transcriptional activities of L3 and R1 fragments in the wild type cells with those in the *hns* mutant indicated that H-NS-mediated repression was abolished or weakened in L3 and R1 fragments in the wild type cells (Fig 4B–4D, compare blue bars with red bars in L3 and R1), indicating that there are H-NS-dependent negative transcriptional regulatory elements in these regions. We concluded that H-NS represses *ybdO* expression dependent on these two specific regions—upstream and downstream regulatory regions (URE and DRE)—which are in the same location in each of the three *E*. *coli* strains (Fig 4A, bottom of the panel, H-NS-dependent regions). URE and DRE are required for H-NS-mediated repression of the *bgl* and *proU* operons and repression via URE and DRE is synergistic in both operons [51]. H-NS may bind both URE and DRE to form a bridge and a stable nucleoprotein complex with consequent spreading of H-NS binding away from the high-affinity H-NS binding sites [51]. The URE and DRE of *ybdO* may also function in a manner similar to that of the URE and DRE for the *bgl* and *proU* operons with respect to the effect of H-NS binding.

The β-galactosidase assays of the systematic deletions surrounding the *ybdO* promoter also indicated that there are sequences involved in repression of promoter activity independent on H-NS. The β-galactosidase activity for fragment R2 of SE15 was greater than that for fragment F of SE15 in the *hns* mutant cells (Fig 4C, compare red bars in R2 and F), suggesting that the region from +164 bp to +239 bp is sufficient to reduce *ybdO* transcription independent of H-NS in SE15. In contrast, in the case of SE11 and K-12, deletion of the same region did not increase the activity for fragment F in the *hns* mutant cells (Fig 4B and 4D, compare red bars in R2 and F). Rather, deletion of the region from +27 bp to +164 bp (fragment R1 lacking +27 bp to +164 bp in fragment R2 and +27 bp to +239 bp in fragment F) increased β-galactosidase activity (Fig 4B and 4D, compare red bars in R1 and F), suggesting that this region reduces *ybdO* transcription independent of H-NS in SE11 and K-12. These results indicated that there are H-NS-independent transcriptional regulatory elements that reduce *ybdO* transcription, and that the location of these elements differs in the *ybdO* loci of SE11 and K-12, and SE15; these elements were designated as negative elements (NE, Fig 4A, bottom panel).

The β-galactosidase activity for the longest DNA fragment, F, of SE15 was ~2-fold higher than that for SE11 and K-12 in the *hns* mutant cells (Fig 4B, 4C and 4D, compare red bars for F of Fig 4C to those of Fig 4B and 4D), whereas the fragment LR, lacking negative elements (URE, DRE and NE), showed similar β-galactosidase levels amongst all strains (Fig 4B, 4C and 4D, compare red and blue bars for LR of Fig 4B or 4C to those of Fig 4D). This suggested that in addition to the difference in the locations of NEs for SE15, and SE11 and K-12, the ability of NEs to reduce transcription in SE15, and SE11 and K-12 differed.

To confirm the different effects of NEs on the promoter activity, we constructed hybrid DNA fragments of the upstream and downstream regions of *ybdO* promoter for SE11 and SE15 (Fig 4E). As seen in Fig 4F, the transcription for all hybrid fragments containing the SE11 coding region (Fig 4F, red bars in lanes a–d) tended to be lower than all hybrid fragments containing the SE15 coding region in the *hns* mutant cells (Fig 4F, red bars in lanes e–h). We thus concluded that the diversity of *ybdO* transcription between SE11 and SE15 is a consequence of sequence divergence downstream of the *ybdO* promoter, including NEs.

## Discussion

In this analysis, we determined that H-NS-bound regions in *E*. *coli* genome have been highly conserved during *E*. *coli* evolution. This is supported by the previous finding that H-NS-bound genes are conserved in *Salmonella* and *E*. *coli* [19]. Phylogenetic analysis indicated that the sequence diversity in H-NS-bound regions tended to be greater than that in H-NS-unbound regions. This tendency was limited to the regulatory intergenic regions (upstream of genes) and coding regions, in which transcriptional regulatory elements often exist. These findings suggest that H-NS-bound regulatory regions are much freer to evolve than H-NS-unbound regulatory regions because H-NS-mediated repression of genes would reduce the negative impact of sequence substitutions for instances in which such substitutions result in altered expression and/or function of genes that are toxic to host cells.

We have also evaluated whether sequence diversity in H-NS-bound regions contributes to variations in transcription using *ybdO* as a test gene. The results indicate that transcription of *ybdO* differs among *E*. *coli* strains and that *ybdO* expression is repressed by H-NS in wild-type *E*. *coli*. This observation supports our hypothesis that sequence substitutions in H-NS-bound regions contribute to the observed diversity of transcriptional regulation of H-NS-bound genes among *E*. *coli* strains, which may provide *E*. *coli* strains the opportunity to adapt to new habitats by integrating HTGs.

Interestingly, the H-NS-bound orthologous genes located within the “common” segments among SE11, SE15, and K-12 significantly overlapped with HTGs (*p* < 0.001, Fisher’s exact test; S2 Table), which were predicted as HTGs based on at least one prediction method [40–42]. We have also showed, that, in proteobacteria, H-NS-bound Core genes were less conserved than H-NS-unbound Core genes (Fig 2E), suggesting that the H-NS-bound Core genes tend to be genes acquired by ancestors of *E*. *coli*. These observations suggest that H-NS-bound genes located within the “common” segments were horizontally transferred into the ancestors of *E*. *coli*, and these genes persist in contemporary *E*. *coli* strains.

Our analysis reveals that the tendency for greater sequence divergence of H-NS-bound intergenic regions compared with those in H-NS-unbound intergenic regions has been limited to regions upstream of genes (class II intergenic regions). This relative greater sequence diversity of H-NS-bound intergenic regions was observed in both types of intergenic regions: HTG-intergenic regions sandwiched by HTGs, and Core-intergenic regions sandwiched by Core genes (Fig 3E). Therefore, the relatively greater sequence diversity in the H-NS-bound class II intergenic regions cannot be explained only by the rapid adaptation of horizontally transferred DNAs to host cells. It is likely that, compared with H-NS-unbound class II intergenic regions, H-NS has made H-NS-bound class II intergenic regions much freer to evolve by repressing the expression of HTGs.

It was difficult to clearly determine the contribution of H-NS binding to the observed greater dN values calculated for H-NS-bound genes. We found that the dN values for H-NS-bound Core genes were significantly greater than that for H-NS-unbound Core genes. This can be simply explained by the apparently slower evolution of the H-NS-unbound Core genes because these include many essential genes, including “information” proteins, e.g., translation-related proteins that have evolved at a significantly slower rate compared with metabolic proteins including those encoded by HTGs [48], and H-NS-bound Core genes may have been horizontally transferred in ancient ancestors of *E*. *coli*. Interestingly, we found that the dS values for H-NS-bound Core genes were also greater than those of H-NS-unbound Core genes (Fig 2D, Core genes). In addition, the expression of H-NS-bound Core genes tended to be lesser than that of H-NS-unbound Core genes (Fig 2F, Core genes). It was known that the dN and dS values for low-expression genes are greater than those of high-expression genes [48]. Therefore, H-NS-mediated repression may increase the sequence diversification of H-NS-bound genes by reducing the expression of H-NS-bound genes. Furthermore, there are H-NS-bound HTGs that have a greater dN than many H-NS-unbound HTGs (Fig 2C). Taken together, our results suggest that H-NS-mediated repression contributes, at least partially, to the observed higher rate of sequence substitution in H-NS-bound coding regions compared with H-NS-unbound coding regions.

Recent work indicated that the average mutation rate in regions bound by one of four *E*. *coli* nucleoid association proteins(NAPs), H-NS, Fis, IHF-A, IHF-B, in the *E*. *coli* genome, is lower than that of NAP-unbound regions [52]. In contrast to the analysis by Warnecke et al., our analysis indicated that the rate of sequence substitution in H-NS-bound regions was higher than that of H-NS-unbound regions. In our analysis, the effects of H-NS binding were limited to class II intergenic regions and coding regions, while Warnecke et al. reported an average of sequence substitutions at four-fold non-synonymous sites in coding and intergenic regions [52]. Therefore, the apparent discrepancy between our results and those of Warnecke et al. may be a consequence of differences in the genes and protein binding regions used for the two analyses.

We also evaluated whether the sequence diversity in H-NS-bound regions could alter transcription of the affected genes. This indeed was the case for at least one of the H-NS-bound genes, namely *ybdO*. We identified H-NS-independent NEs in the coding regions of *ybdO*, whose locations and activities differed among *E*. *coli* strains (Fig 4A). Although further analyses are needed to reveal the molecular mechanism by which an NE inhibits *ybdO* transcription, our results suggest that sequence substitutions downstream of *ybdO* promoters, including NE, dictate the *ybdO* transcription level. Recently, hundreds to ~20,000 RNA polymerase (RNAP) pause sites were identified in exponentially growing *E*. *coli* cells, and it was suggested that RNAP pausing is one of the common mechanisms by which gene expression is controlled [53–55]. It is difficult to directly evaluate the possibility that RNAP will pause at NEs based on the data from those studies because *ybdO* expression remained low in exponentially growing cells. Nevertheless, differential pausing of the transcription machinery at NE sites constitutes one possible explanation for the observed variation in NE potency among *E*. *coli* strains.

The assignment of transcription start sites for *ybdO* in SE11, SE15, and K-12 indicated that the location of the *ybdO* promoter is conserved among *E*. *coli* strains, although we found that the nucleotide sequences in *ybdO* promoter proximal regions were different (S3B Fig). Although we could not find any typical transcriptional regulator that recognizes sequences affected by substitutions near the *ybdO* promoter, such substitutions would provide the opportunity to acquire positive regulation because it has been shown that, during evolution, HTGs acquired positive regulation when they became integrated in the host transcriptional network [25]. Because HTGs have contributed to the evolution of host-cell metabolic networks that allow adaptation to new environments [56], further investigation of *ybdO* transcriptional regulation under different growth conditions, e.g., in minimal medium, will be needed to clearly define the effects of sequence substitutions on *ybdO* promoter function.

In our present study, the β-galactosidase assay did not allow us to directly evaluate whether H-NS-mediated repression is crucial for introducing sequence substitutions that alter the transcriptional regulation of HTGs. It is possible that H-NS directly enhances the sequence substitution rate in class II intergenic regions and coding regions by unknown mechanisms. To delineate the importance of H-NS-mediated repression in the evolution of the transcriptional regulation, further investigations must directly evaluate the relationship between transcriptional repression and sequence substitutions, i.e., *in vitro* evolution experiments using the *hns* deletion mutant.

It has been reported that variance in gene expression contributes to the heterogeneity of *E*. *coli* strains, which could potentiate the ability of *E*. *coli* strains to adapt new ecological niches. The *mat* (meningitis-associated and temperature regulated) fimbrial gene cluster is conserved across many *E*. *coli* strains [57]. However, B2 group strains have acquired the ability to express *mat* genes despite H-NS-mediated repression at low temperature, low pH, and high acetate concentration, conditions under which *mat* is not expressed in strains of groups A and B1 [57]. Differences in *mat* regulation among *E*. *coli* strains is caused by polymorphisms in gene promoters repressed by H-NS [57]. Thus, *mat* and *ybdO* might exemplify the biological importance of sequence diversity in H-NS-bound regions for adaptation of *E*. *coli* strains to different ecological niches.

Based on our observations, we hypothesize that H-NS-mediated repression helps HTGs to adapt their transcriptional regulation to the local environment for host *E*. *coli* strains by accelerating the rate of sequence polymorphism in H-NS-bound regulatory regions. This hypothesis is supported by the finding that the optimization of HTG expression was initially found to occur via the evolution of regulatory regions rather than coding regions [58]. Our results support the proposal that H-NS-mediated repression is a valuable mechanism by which host cells can integrate HTGs into the host transcriptional regulatory network [3].

## Materials and Methods

### Primers

The primers used in this study are listed in S4 Table.

### Construction of strains used for ChAP-seq experiments and the β-galactosidase assay

Strains used in this study are listed in S5 Table. To generate the K-12 (W3110) derivative expressing H-NS C-terminally tagged with 12 histidines (12His), we used a modified one-step gene inactivation method [59]. Plasmid pSTV28-C-12His, which was kindly provided by Dr. Mika Yoshimura, was constructed by inserting the chemically synthesized 12His coding sequence and a kanamycin resistance gene derived from plasmid pKD4 [59] into the multiple cloning site of pSTV28 (Takara Bio, Japan). We amplified a DNA fragment containing the 12His sequence flanked with the Arg-Gly-Ser linker and kanamycin resistance gene by PCR using pSTV28-C-12His and the TOP705-TOP706 primer set. To facilitate insertion of the PCR product into the chromosome, we added a ~70-bp sequence of the *hns* coding region and its downstream region to the TOP705 and TOP706 primer sequences, respectively. The BW25113 cells harboring pKD46 encoding Red recombinase [59] were transformed with the amplified DNA fragment, and transformants in which linker and 12His sequences were inserted at the 3’ end of the chromosomal *hns* through a double-crossover at the coding and downstream regions of *hns*, were selected with kanamycin to obtain the K-12 (BW25113) H-NS-12His strain. *hns* fused with the 12His sequence was transferred into the K-12 (W3110) chromosome, together with the kanamycin resistance gene, via phage P1 transduction.

Because the SE11 and SE15 strains are resistant to P1, to construct the derivatives expressing 12His-tagged H-NS, we adopted the gene-doctoring method [60] using plasmid pDEX harboring an I-*Sce*I recognition site and *sucB* and pACBSR harboring I-*Sce*I and the kanamycin resistance gene [61]. The 12His coding sequence and kanamycin resistance gene in pSTV28-C-12His were amplified by PCR using primers hns-His12-H1 and hns-His12-H2-1 (for SE11) or primers hns-His12-H1 and hns-His12-H2-2 (for SE15). Amplified fragments were inserted into the *Eco*RV site of pDEX. SE11 and SE15 were co-transformed with two plasmids—pACBSR and the appropriate pDEX-H-NS-His12—with subsequent selection for kanamycin and sucrose resistance. Transformants were cultured in LB liquid medium containing 25 μg/ml chloramphenicol and 0.2% arabinose for a few hours, inducing inactivation of pDEX-H-NS-His12 by I-*Sce*1. Cells were harvested by centrifugation and regrown in LB liquid medium containing 5% sucrose at 30°C for 2 h to cure pACBSR. Finally, kanamycin- and sucrose-resistant colonies were selected on an LB plate containing 50 μg/ml kanamycin and 5% sucrose to isolate transformants in which the 12His sequence and kanamycin resistance gene were integrated into the chromosome via homologous recombination at the *hns* coding sequence and sequences downstream of *hns* introduced at the 5’ and 3’ ends of the PCR products, respectively.

Expression of H-NS-12His in the created strains was confirmed by western blotting using an antibody against His tag (MBL, Japan). Sequencing of the introduced *hns* tagged with 12His revealed a point mutation within the *hns* coding region in the K-12 derivative, probably attributable to an error during synthesis of the primer used to generate the strain. Because the identified point mutation (from AAG [136K] to AAA [136K]) did not lead to an amino acid substitution in H-NS, the strain was employed for further analysis. Noteworthy, the C-terminal 12His tag did not negatively affect the growth of K-12, SE11 and SE15 in Luria-Bertani (LB) medium under aerobic conditions.

The *hns* deletion mutant (MC4100 Δ*hns*::Km) used in the β-galactosidase assay was constructed using P1 transduction of the *hns*::km allele from K-12 (W3110) *hns*::km [62] into MC4100.

### ChAP-seq experiments

ChAP was performed according to the reported procedure [34] using 50-ml cultures of *E*. *coli* grown in LB medium under aerobic conditions at 37°C. DNA fragments that co-purified with H-NS-12His and in the supernatant fraction before ChAP were sequenced using the Illumina GA sequencer (Illumina, USA). We performed ChAP-seq experiments twice for each strain, and 36-bp single-end reads provided 8–11 million reads (first set of sequencing results of ChAP and WCE fractions of three strains) and 5–10 million reads (second set). The sequence data used in this publication have been deposited in the DRA database (DDBJ Sequence Read Archive: http://trace.ddbj.nig.ac.jp/dra/index_e.shtml) with accession number: DRA000539.

### Multiple alignment of genome sequences of the three strains

Complete sequences and annotations of genes in the three genomes (SE11 [AP009240.1], SE15 [AP009378.1], and K-12 [W3110; AP009048.1]) were obtained from the NCBI GenBank database. We compared the three chromosome sequences and their synteny of gene arrangement using the Mauve 2.3.1 program for Progressive Mauve algorithm with default parameters [35,36] and determined the segments that were conserved in all three strains (“common”) and unique to two (“shared”) or one (“specific”) strain(s). The K-12 (W3110) chromosome contains a large inverted region (~800 kbp) surrounded by two ribosomal operons (3,423,096–4,216,800 bp). To avoid eliminating this region from “common” segments by the above analysis, we manually reversed this region in the chromosome sequence of K-12 (W3110) before alignment using the Mauve program.

The sum of the consensus sequences of “common” segments was 3,888,365 bp. However, the DNA sequences of “common” segments in each strain occasionally had small gaps compared with the consensus “common” segments of all three strains. Thus, the total length of the “common” segment in each strain was shorter than that of the consensus segments, specifically, SE11: 3,886,369 bp, SE15: 3,886,157 bp, K-12 (W3110): 3,886,242 bp.

### Short reads mapping, normalization of mapped reads, and estimation of H-NS binding intensities for each nucleotide

Short reads (36 bp) obtained from the Illumina GA sequencer were uniquely mapped on to the reference genome sequences of K-12 (W3110), SE11, and SE15, allowing no gaps and up to two mismatches using the BLAT program [63]. Because the purpose of this study was to compare the DNA binding profiles of H-NS in these three strains, we mapped the short reads only on the chromosome in each strain. Uniquely aligned reads were specifically used for further analysis. In addition, because it is impossible to specifically map 36-bp reads to one of seven rRNA genes in the *E*. *coli* genome and the rRNA genes were not used for the phylogenetic analysis, rRNA coding regions were not included in this study. Next, mapped reads were extended to 200 bp in length from the 3’ end of each read, taking into account the length of DNA fragments to construct the sequence library. We subsequently normalized the number of mapped read numbers at every nucleotide in each experiment by global scaling, in which the number of mapped reads at each nucleotide was divided by the median number of mapped reads at all nucleotides in each sample. Finally, to estimate the H-NS binding intensity at every nucleotide, we divided the scaled number of mapped reads for DNA from the ChAP fraction by that from WCE before ChAP-mediated purification to remove the effects of sequence preference of Illumina GA. In cases where the number of mapped reads at some positions was zero for the ChAP or WCE fraction, the H-NS binding intensity of the position was defined as zero. As H-NS binding intensity spanned a wide range of values, log_10_-scaled values were used for subsequent analysis.

To evaluate our normalization procedure in the comparison of different sequencing outputs, the average H-NS binding intensity in 200-bp windows was calculated in 100-bp steps along whole-genome sequences. Scatter plots shown in S4A Fig demonstrate that correlation coefficients of estimated average H-NS binding intensities in each window obtained in all experiments for each strain were high (*r* > 0.8). In addition, correlation coefficients of the binding intensities of corresponding windows in “common” segments of different strains were greater than 0.69 for all combinations (S4B Fig), indicating that our normalization procedure was adequate.

### Determination of the H-NS binding regions

H-NS binding intensity showed a bimodal distribution of “noise” components at ~1.0, and “signal” components, which ranged from 10.0 to 1000.0 (S5 Fig). In four experiments (all data from the 1st experiment and K-12 data from the 2nd experiment), the bimodal distribution was clear, and noise components could be clearly discriminated from signal components. In these cases, noise components could be approximated as a normal distribution in which *μ* represents mode and σ is 0.2 (S5 Fig). Thus, we set the threshold value to remove noise components as mode + 3σ (= 0.6). In the two remaining experiments (data for SE11 and SE15 in the 2nd experiment), noise components were not clearly separable from signal components, and the two possibly overlapped. However, we referred to the threshold value from other experiments (mode + 0.6) to infer signal components in these cases (S5 Fig). Next, we searched for regions in which H-NS binding intensity was greater than the threshold. To remove the effects of the remaining noise signals by our threshold setting, we extracted regions longer than 200 bp as possible H-NS binding sequences. Finally, we compared the H-NS-bound regions obtained in the two experiments for each strain, and overlapping regions were identified as H-NS-bound regions for further analysis. To evaluate the accuracy of our mapping and determination of H-NS-bound regions, we required the second mapping result of our short reads that was acquired with a different mapping program, namely Bowtie 2 [64], and we also required a determination of H-NS-bound regions with the Bowtie 2 mapping results. Comparison of H-NS-bound regions determined by BLAT mapping (original result) and by Bowtie 2 mapping (second mapping) indicated that the H-NS-bound regions that were determined with the two mapping procedures were 97% identical. This result clearly indicated that our mapping and determination of H-NS-bound regions were highly reliable, and thus we conducted subsequent analyses using the BLAT mapping results for the H-NS-bound regions. The reproducibility of H-NS binding profiles for the whole genome of each strain (SE11, SE15, K-12) are indicated in S6 Fig. In addition, the conservation of H-NS-bound regions in “common” segments within each whole genome is presented in S7 Fig.

### Phylogenetic analysis of orthologous genes

The 44 *E*. *coli* strains whose genome sequences had been annotated in RefSeq were used for our phylogenetic analysis (S1 Table). All chromosome sequences and the annotations of the 44 strains were obtained from the RefSeq (NCBI Reference Sequence database). Because RefSeq represents reference sequences for which gene annotation is consistent and standardized, it enabled us to precisely identify orthologous genes in the *E*. *coli* lineage.

To identify the conserved orthologous genes in the *E*. *coli* strains, we initially evaluated the level of conservation of the amino acid sequence translated from each gene. We carried out all-against-all reciprocal BLASTP comparisons for all proteins in all strains followed by clustering of the BLASTP hits using OrthoMCL [65]. To remove genes encoding mobile elements, duplicate genes, and pseudogenes, which have repetitive sequences, and paralogs that interfere with phylogenetic analysis, the proteins encoded by prophage and insertion (IS) genes were searched by BLASTP against the ACLAME database [66] and ISFinder [67] and excluded from further analysis. Paralogs and hidden paralogs were also removed from the orthologous proteins by excluding the gene clusters containing more than two copies of the proteins present in one strain. Then, we selected the 3,107 orthologous proteins (gene clusters) that were conserved in >90% of strains (40 of 44), in which K-12, SE11, and SE15 were always included. From the selected orthologous proteins, the 405 orthologous proteins encoded by genes that had at least one broken codon with one or two nucleotide deletions or insertions in at least one strain were excluded to remove pseudogenes. Ultimately, 2,702 orthologous protein clusters were selected for subsequent analysis (S8 Fig). Multiple sequence alignment for each orthologous protein cluster was performed using MAFFT [68] (G-INS-i algorithm) and back-translated into the aligned nucleotide sequence. GBLOCKs [69] (codon model, default settings) was used to remove gaps and unreliably aligned positions. To assess the accuracy of our orthologous gene sets, we constructed a representative phylogenetic tree based on the concatenated super-alignment. We concatenated the alignments of 100 randomly chosen orthologous genes and inferred the maximum likelihood (ML) tree using PhyML [70] with the following parameters: -b 100 -d nt -m HKY85 -v 0 -c 4 -a 1. The resulting ML tree reflected the phylogenic relationships revealed in previous studies [71] (S9 Fig). The dN and dS values for orthologous genes were computed using Codeml from PAML [72] (settings: tree = ML gene tree from PhyML, CodonFreq = F3X4, clock = 0, kappa = estimated by ML, omega = estimated by ML, alpha = 0, rho = 0).

In this analysis, we identified H-NS-bound genes as those that overlapped with H-NS-bound regions determined in at least one strain of SE11, SE15, and K-12, because the H-NS-bound regions in common segments were essentially overlapping. To evaluate this classification, we manually inspected H-NS binding signals in each H-NS-bound gene, which also indicated that, even if the H-NS-bound region overlapped with the H-NS-bound gene in only one or two strains, possible H-NS binding signals were observed in the H-NS-bound gene in the other strains, albeit the H-NS binding intensity for the gene was lower than the threshold value in most cases. There were 42 genes (S6 Table) that were specifically bound by H-NS in only one or two strains, in which H-NS binding was dependent on the specific or shared segments that were localized in the vicinity (in many cases, neighbors) of these 42 genes in the chromosomes (a typical example is presented in S10 Fig, where *ytfI* is the H-NS-bound specific segment), because H-NS binding was not detected for strains in which the specific segments were absent from the chromosomes. Therefore, we regarded these 42 genes as H-NS-unbound genes.

We verified the significance of the higher dN in the H-NS-bound regions compared with that in the H-NS-unbound regions by modifying the definition of the H-NS-bound genes. The results indicated that the dN of the H-NS-bound genes was significantly greater than that of the H-NS-unbound genes, even when we excluded the genes in which H-NS binding was limited to the 3’ end and the length overlapping with the H-NS-bound regions was ≤10% of the total gene length or if the genes included in transcriptional units whose promoters, intergenic, or coding regions could bind H-NS were considered as H-NS-bound genes (S11B and S11C Fig). Furthermore, even when we regarded the 42 genes that bound to H-NS in a specific- or shared segment—dependent manner (described above) as H-NS-bound genes, the dN in the H-NS-bound genes was still significantly greater than that in the H-NS-unbound genes (*p* < 0.001). These results suggested that our conclusion concerning the sequence diversity of H-NS-bound genes was not affected by the definition of the H-NS-bound genes.

Although we carefully selected orthologous genes based on the above criteria, it was possible that horizontal gene transfer and recombination events among *E*. *coli* strains might have affected our results—particularly the horizontal transfer and recombination events in H-NS-bound orthologous genes. To validate the potential effects of horizontal transfer and recombination events on our analysis, we calculated minimal tree split compatibilities between H-NS-bound and -unbound orthologs by which we could evaluate whether the genes had been vertically evolved in the *E*. *coli* lineage [73,74]. If the orthologs were present in the ancestral *E*. *coli* genome before the divergence of the *E*. *coli* lineage and had not been involved in horizontal transfer or recombination events among *E*. *coli* strains, their phylogenies should be similar. Therefore, if H-NS-bound orthologs tend to be transferred horizontally more so than H-NS-unbound orthologs, phylogenies of trees would differ between H-NS-bound and -unbound orthologs. To avoid a sample-size bias, we reconstructed five datasets: set A, trees of H-NS-unbound orthologs (N = 2,183); set B, trees of H-NS-bound orthologs (N = 519); set C, trees of downsampled H-NS-unbound orthologs (N = 519, randomly sampled without replacement); set D, trees of H-NS-bound orthologs with a simulated horizontal transfer event (N = 519, constructed by a minimal perturbation of set B where for each tree a randomly selected branch was pruned and then regrafted at a random branch); set E, trees of H-NS-bound orthologs with two simulated horizontal transfer events (N = 519). We used set A as a reference dataset and calculated minimal tree split compatibilities for each tree in sets B, C, D, and E against set A. The distributions of compatibility scores for each dataset were compared using the two-sided Kolmogorov-Smirnov test. We could not reject the null hypothesis that H-NS-bound and H-NS-unbound tree sets were drawn from the same distribution (S12 Fig, *p* = 0.16), whereas the slightest perturbation (a single horizontal transfer event) strongly rejected the null hypothesis (S12 Fig, *p* < 0.001). This suggested that there was no bias for sequence substitutions caused by horizontal gene transfer or recombination events by which the number of H-NS-bound orthologs would have been much greater than H-NS-unbound orthologs.

It is also known that gaps in alignments can reduce the accuracy of the estimation of sequence diversity because of the difficulty in achieving an accurate alignment around gap positions [75]. Thus, we calculated the dN and dS values for coding regions only using the orthologous gene clusters without gaps in their alignments and compared the sequence substitution rates in H-NS-bound and -unbound regions. The results are shown in S13A and S13B Fig. The sequence diversity at non-synonymous positions in the H-NS-bound coding regions was significantly greater than that in H-NS-unbound regions. Therefore, this result suggested that our conclusion concerning the sequence diversity of coding regions was not affected by misalignment caused by insertions/deletions in coding regions.

When we investigated the conservation of the four classes (H-NS-bound HTGs, H-NS-unbound HTGs, H-NS-bound Core genes, and H-NS-unbound Core genes) of genes in proteobacterial species classified in the same family, the same class, or the same phylum as *E*. *coli*, MBGD was used for the comparison of the conservation of genes [43–46]. First, we constructed the ortholog cluster table by using 48 completely sequenced bacterial genomes from MBGD. Of these 48 genomes, one was *E*. *coli* K-12 MG1655, 25 strains belonged to the same family but different genus than *E*. *coli* (family Enterobacteriaceae), 14 strains belonged to the same class but different family than *E*. *coli* (class Gammaproteobacteria), and 8 were strains belonged to the same phylum but different class than *E*. *coli* (phylum Proteobacteria). Strains used in this analysis are listed in S7 Table. For clustering parameters, we used the default values of MBGD. From this ortholog cluster table, we searched our *E*. *coli* orthologous genes by gene name. In total, 2,098 genes were identified (H-NS-bound HTGs: N = 157; H-NS-unbound HTGs: N = 224; H-NS-bound Core genes: N = 174; H-NS-unbound Core genes: N = 1,543). Then, we checked for the presence or absence of these genes in each of the 48 genomes (S14 Fig). The conservation rate for each class of genes was calculated for each genome separately. Finally, the average conservation rates were calculated separately for the same family genomes, the same class genomes, and the same phylum genomes, and we compared these values for each class of genes.

### Phylogenetic analysis of conserved intergenic regions

Intergenic regions used for phylogenetic analysis should be carefully selected because these regions often have large insertion/deletion sequences that lead to spurious alignments. We defined "conserved" intergenic regions as the regions presently between two neighbouring orthologous genes we had determined (see above) in the same order and direction in *E*. *coli* strains in which these orthologous genes were identified. In addition, we selected the regions whose length was no less than 10 bp nor more than 300 bp in all chromosomes. The multiple alignment of each set of conserved intergenic regions was performed using MAFFT (G-INS-i algorithm). After the multiple sequence alignment, we selected a cluster in which the lengths of all intergenic regions in each cluster were different, less than 10% of the aligned sequence length of a cluster, implying that no intergenic regions with long insertions and/or deletions were used for subsequent analyses. Consequently, 712 regions were selected as conserved intergenic regions (average length was 94.8 bp).

We then estimated the sequence diversity matrices for those intergenic regions using Baseml from PAML (setting: tree = ML tree from PhyML, model = 7, clock = 0, kappa = 2.5 (starting value), fix_kappa = 0 (ML estimation of kappa), alpha = 0, fix_alpha = 1 (fixed value), rho = 0, fix_rho = 1 (fixed value), npark = 0, nhome = 0, Mgene = 0.). In addition, we removed sets in which the evolutionary distance of at least a pair of strains was >1.0, meaning that the sequences of those regions were too divergent to yield a correct alignment. Finally, we selected the 703 conserved intergenic regions, including 94 H-NS-bound intergenic regions that overlapped with the H-NS-bound regions identified in at least one strain, and compared the sequence diversification rates in intergenic regions bound or not bound to H-NS. In this analysis, we also calculated the sequence substitution rate for each intergenic region only using the set of the intergenic regions without gaps and concluded that the sequence diversity of intergenic regions was not affected by any potential misalignment caused by insertions/deletions in coding regions (S13C Fig).

We further assessed the impact of the presence of a promoter(s) on the extent of proximal sequence diversification. The intergenic regions with known promoters were selected from the class II regions using the information about promoters in the K-12 strain acquired from RegulonDB [76]. The sequence diversity of the H-NS-bound regions was greater than that of the H-NS-unbound regions, although the difference in sequence diversity between H-NS-bound and H-NS-unbound was even greater in regions with known promoters than in the regions without known promoters (S15 Fig; *p* < 0.001 [with known promoters], *p* = 0.0079 [without known promoters]). These results suggested that the presence of other transcriptional regulatory elements, such as pause and termination signals, may also affect the observed H-NS binding—dependent increase of sequence substitution rates.

### Analysis of transcriptional activity of sequence-divergent promoters using the β-galactosidase assay

#### Plasmid construction for the β-galactosidase assay

To investigate the effects of sequence diversity without the influence of differences of genetic backgrounds in the three strains, we examined the effects of sequence divergence of *ybdO* in different strains under the same genetic background using the β-galactosidase assay. Plasmids used for the β-galactosidase assay are listed in S5 Table and were constructed using plasmid pRW50 [50]. Various DNA fragments including *ybdO* promoter regions and regions up- and downstream of the promoters indicated in Fig 4A were amplified by PCR using chromosomal DNA purified from strains SE11, SE15, and K-12 as templates and appropriate primers (S4 Table), and the products were cloned into pRW50 as *EcoR*I/*Hind*III fragments. Hybrid DNA fragments fused the SE11 promoter proximal region (or the upstream region of SE11promoter) and the downstream region of SE15 promoter (or SE15 coding region), or the SE15 promoter proximal region (or the upstream region of SE15 promoter) and the downstream region of SE11 promoter (or SE11 coding region) were amplified via recombinant PCR using four primers for each hybrid fragment (S4 Table). Two DNA fragments were independently amplified by PCR using purified SE11 and SE15 chromosomal DNA, and the resultant DNA fragments were purified and used as template DNA for the second PCR. The first PCR was performed with primers that have sequences corresponding to the 5’ or 3’ ends of the fragments and the junction points. A second PCR was performed with the primers corresponding to the 5’and 3’ ends of the fragments. The junction point of each fragment is indicated in Fig 4E. The resultant DNA fragments had *EcoR*I/*Hind*III sites at both ends and were cloned into pRW50. *E*. *coli* K-12 DH5α cells were transformed with the plasmids, and the transformants were selected on the basis of tetracycline (5 μg/ml) resistance. These plasmids were subsequently introduced into the strains MC4100 and MC4100 Δ*hns*::km to prepare the reporter strains. Pre-cultures of the reporter strains were grown overnight at 37°C in 2 ml of LB medium containing 5 μg/ml tetracycline and then used to reinoculate the *E*. *coli* cells in 10 ml fresh LB medium containing 5 μg/ml tetracycline at 1:500 (v/v). The cells were cultivated under aerobic conditions at 37°C and harvested at various times (for time course experiments), after 5 h (for the assay of wild-type cells in stationary phase), or after 7 h (for *hns* mutant cells in stationary phase) from the start of cultivation. β-galactosidase activity was measured as described by Miller [77] and is expressed in Miller units.

#### Investigation of growth phase—dependent altered expression and H-NS-mediated repression of the *ybdO* promoter

To monitor the growth phase—dependent transcriptional alteration of *ybdO*, we performed a β-galactosidase assay using pRW50 carrying DNA segments from −250 to +239 bp relative to the *ybdO* start codon (the L2 fragment in Fig 4A) of the three strains. The plasmids were introduced into K-12 wild-type (MC4100) or the *hns* mutant (MC4100 Δ*hns*::km) strain in which *hns* was replaced by the kanamycin resistance gene. β-galactosidase activity was measured using cells grown in LB medium at 37°C under aerobic conditions. The transcriptional activities of all three fusions gradually increased during growth at log phase and then plateaued during the early stationary phase both in the wild type and the *hns* mutant (S2B Fig). In addition, the transcriptional activities of three strains decreased in the wild type as compared with the *hns* mutant, indicating that *ybdO* transcription is repressed by H-NS in K-12, SE11 and SE15.

#### Mapping of H-NS-dependent regulatory elements (URE and DRE) for *ybdO* and NEs

To determine the regions responsible for the regulating *ybdO* transcription, we utilized the β-galactosidase assay using *lac*-operon fusions involving various lengths of segments in the upstream and coding regions of *ybdO* of the three strains (Fig 4A; segments L1, L2, L3, L4, LR, R1, R2, and F). The segments fused were selected based on sequence conservation in the upstream and coding regions of *ybdO* of the three strains. Because the transcriptional activity of *ybdO* plateaued early during the stationary phase, we measured the activity only during the early stationary phase, which corresponded to 5 h of cultivation for the wild type and 7 h of cultivation for the *hns* mutant (S2B Fig).

As compared with the transcriptional activity of the fusion with the L2 fragment, the addition of further upstream sequences did not significantly affect the *ybdO* transcription of the SE11 and K-12 fusions in wild-type cells (Fig 4B and 4D, compare blue bars of L1 or F with those of L2). The addition of the upstream sequence to −298 bp reduced the transcriptional activity of the SE15 fusion to basal level (fully repressed; Fig 4C, compare blue bars of L1 and L2) in wild-type cells. On the other hand, deletion of the sequence from −250 bp to −176 bp (see Fig 4A, compare L3 with L2) increased the transcriptional activities of all three fusions in wild-type cells (Fig 4B–4D, compare blue bars of L3 and L2). The transcriptional activities with F, L1, L2, and L3 in the *hns* mutant (Fig 4B–4D, red bars of F, L1, L2 and L3) were at the same level as that with L3 in wild type (Fig 4B–4D, blue bars of L3). These results indicated that the nucleotide sequence between −250 and −176 bp was necessary for H-NS-dependent negative regulation of *ybdO* transcription of all three strains. Therefore, this region was denoted as an URE (upstream regulatory element; Fig 4A). The sequence from −298 to −250 bp also contributed to the full H-NS-mediated repression of the SE15 promoter along with the URE. Because further deletion of upstream sequences to −99 bp abolished transcriptional activity in each of the three strains (even in the *hns* mutant; Fig 4B–4D, L4), the sequence from −179 to −99 bp appeared to be essential for the promoter activity of *ybdO* of all three strains. This result was consistent with the mapping of the 5’ end of SE11 and SE15 *ybdO* mRNA with 5’-RACE and the transcription start site of K-12 *ybdO* determined by differential RNA-seq [49] (Fig 4A).

As compared with the promoter activity in the longest fragment F, deletion of +239 to +164 bp (see Fig 4A, compare R2 with F) increased the transcriptional activity of the SE15 fusion in the *hns* mutant (Fig 4C, compare red bars of R2 and F), whereas the same deletion had little effect on the transcriptional activities of the SE11 and K-12 fusions (Fig 4B and 4D, compare red bars of R2 with those of F). Thus, the region from +239 to +164 is crucial for H-NS-independent negative regulation of SE15 *ybdO*. Further deletion of the sequence from +164 to +27 bp increased the transcriptional activities of the SE11 and K-12 fusions in the *hns* mutant (Fig 4B and 4D, compare red bars of R1 with those of R2) but had little effect on the transcriptional activity of the SE15 fusion (Fig 4C, compare red bars of R1 and R2). Hence, the region from +164 to +27 bp is most crucial for the H-NS-independent negative regulation of SE11 and K-12 *ybdO*. This sequence of SE15 was required only for the H-NS-dependent negative regulation because deletion of the sequence increased the activity of the SE15 promoter only in the wild type (Fig 4C, compare blue bars of R1 and R2). The corresponding regions of SE11 and K-12 are involved both in H-NS-dependent and -independent negative regulation because the H-NS-mediated repression was lower in R1 than in R2 (Fig 4B and 4D; in each fusion, the relative ratio of *hns* mutant and wild type [red bar / blue bar] of R2 was greater than that of R1; R2 and R1 of SE11 were 8.4 and 3.0, respectively; R2 and R1 of K-12 were 3.3 and 1.8, respectively). From these results, we defined the downstream regulatory regions of *ybdO* as NE^SE15^ and NE^SE11, K12^, which are the H-NS-independent negative elements for SE15 and for SE11 and K-12, respectively, and as DRE, the downstream regulatory element, which is necessary for H-NS-dependent repression for all three strains (Fig 4A). Although the activities of the *ybdO* promoters of SE11 and SE15 with LR and R1 in the *hns* mutant were comparable to each other, the transcriptional activity of the longest SE15 segment (Fig 4C, red bar of F) was higher than that of SE11 (Fig 4B, red bar of F). This result indicated that the repression potential of NE^SE15^ was weaker than that of NE^SE11, K-12^. We further evaluated the difference in transcriptional regulation of NEs using pRW50 carrying hybrid DNA fragments including the SE11 promoter (or upstream region of SE11 promoter) with the SE15 coding region (or SE15 promoter and coding regions) or the SE15 promoter (or upstream region of SE15 promoter) with the SE11 coding region (or SE11 promoter and coding regions). We amplified DNA fragments including those containing regions up- and downstream of the *ybdO* promoter including the 5’ end of the *ybdO* coding region of SE11 and SE15 and fused at –176, –99, –34 and +27 bp and vice versa (Fig 4E). In wild-type cells, H-NS still repressed *ybdO* expression in the hybrid DNA fragments, except for fragment d in which H-NS-mediated repression was quite reduced compared with other hybrid fragments. Because the mechanism of H-NS-mediated repression was not the focus of our present study, we did not further investigate this phenomenon. In *hns* mutant cells, the β-galactosidase activity of each hybrid having the SE15 coding region tended to be greater than that of the hybrids having the SE11 coding region. The largest difference was detected when the DNA fragments were fused at –33 bp; therefore, part of the intergenic regions might also contribute to H-NS-independent negative regulation of *ybdO*. Taken together, *ybdO* expression has diverged between SE15 and SE11 (or K-12), mainly attributable to the difference in the activities of NE^SE15^ and NE^SE11, K12^. Notably, H-NS-dependent negative regulation kept the promoter activity at the basal level (low expression) in wild-type *E*. *coli* cells (Fig 4B–4D, blue bars of F).

### 5’ RACE and determination of the 5’ end of *ybdO* transcripts

Total RNA was extracted and purified from *E*. *coli* K-12 (MC4100) transformed by pRW50 carrying LR fragments, in which the major negative regulation of *ybdO* in an H-NS-dependent or -independent manner were cancelled by the deletion of the NEs, URE, and DRE for SE11 and SE15 using the RNeasy Mini kit (Qiagen). RACE was performed with First-Choice RLM-RACE kit (Ambion) using the manufacturer’s manual with modifications. Specifically, RNA (5 μg) was treated with tobacco acid pyrophosphatase or left untreated, and then the 5’ RACE adaptor was ligated to each RNA molecule. cDNA was synthesized from adapter-attached RNA with a random decamer. The 5’ end of *ybdO* was amplified by PCR with primers (5’ RACE Outer Primer and ybdO-D2: CAAGTCGTAGAGATTGGCCATACA [for SE11 *ybdO*] or ybdO-SE15-D2: TAGATCATAAAGATTAGCCATAAC [for SE15 *ybdO*]), and products were visualized after electrophoresis in Gel-Red containing agarose gel and cloned with pGEM-easy (Promega). Sequences of cloned fragments were determined and 5’ ends were mapped on the genome sequences of SE11 and SE15.

### Data deposition

The raw data and their tables are available in our web page, http://palaeo.bio.titech.ac.jp/Resources/hns2015/.

## Supporting Information

S1 Fig Nucleotide substitutions in the promoter and the 5’ end of *ybdO*. (A) Schematic diagram of fragments used in the β-galactosidase assay. Shown is a multiple sequence alignment of *ybdO* including the upstream region for the *E*. *coli* strains. At the top of the first page, the locations of the 5’ and 3’ ends of the fragments are shown according to distance from the *ybdO* start codon. Blocks correspond to the regions that were truncated in shorter fragments cloned into the reporter plasmids for the β-galactosidase assay. The horizontal arrow denote the positions of transcription start sites suggested by 5’-RACE and differential RNA-seq [49]. The alignment of the *ybdO* promoter region and downstream region are shown below each schematic representation of blocks. In this analysis, we independently aligned coding and intergenic regions by different methods (see Materials and Methods). Therefore, we separately indicate the alignment in ortholog0270 (*dsbG* in K-12), intergenic region intergenic0112 (*dsbG*–*ybdO*), and ortholog2573 (*ybdO*). Numbers at the top of the alignment show the positions relative to the start codon of *ybdO* in K-12. The location of each block is indicated at the bottom of the alignment. Sequences for ortholog0270 and ortholog2573 were aligned using protein-based alignment, which was then back-translated to yield DNA sequences. Sequence alignment of the intergenic0112 was performed by the DNA-based alignment. The Alignment of ortholog0270 (*dsbG*) are constructed by sequences of all 44 *E*.*coli* strains, while alignments of intergenic0112(*dsbG*–*ybdO*) and ortholog2573 (*ybdO*) are constructed by 41 sequences. It is due to the fact that *ybdO* are conserved only in 41 *E*.*coli* strains and 3 strains do not possess *ybdO* ortholog. In these 3 strains, recombination or HGT event might have been occurred at the downstream of ortholog0270. Alignments are depicted by UGENE environment [78]. At positions 107 and 106 bp upstream of the *ybdO* initiation codon, we indicate the transcription start site for each of SE11, SE15, and K-12 *ybdO*. (B) Top panel: schematic diagram of the location of block A ~ G in the upstream and coding regions of *ybdO*. Block A~D, F and G are the regions systemtically deleted from the fragments F and Block E + D was corresponding to fragment LR, which were cloned into pRW50 for the β-galactosidase assay (Fig 4A). The frequency of segregating sites in each block among all strains was calculated by dividing the number of segregating sites at which at least one strain had a substitution by the sum of alignment positions in each block. The segregation frequencies among the *E*. *coli* strains used to identify orthologous genes in blocks are shown in the second panel, followed by the frequencies of segregating sites between two strains “SE11 vs K-12”, “SE15 vs SE11”, and “SE15 vs K-12”. The alignment positions at which there are gaps in at least one strain were ignored to calculate the sum of segregating sites in each block both in the total and pairwise comparisons.
(PDF)

S2 Fig Analysis of *ybdO* expression with the β-galactosidase assay (time course). (A) H-NS binding profiles near *ybdO* are presented with CDS maps for SE11 (top), SE15 (middle), and K-12 (bottom), which are segments of the maps in S2 Fig. The yellow arrows show the locations of *ybdO* in K-12, SE11, and SE15. (B) Expression profiles of the SE11, SE15, and K-12 *ybdO* promoters in the time course. The wild type (MC4100) and the *hns* mutant (MC4100 Δ*hns*::km) transformed with pRW derivatives carrying the L2 fragments of SE11 (left), SE15 (middle), and K-12 (right) were grown at 37°C in LB medium under aerobic conditions. The optical density (OD_600_) of the wild-type (open circles with black line) and *hns* mutant (open triangles with dashed black line) cultures and the β-galactosidase activities (Miller units) of the wild type (cross with bold dashed line) and the *hns* mutant (open diamond with bold black line) were measured every hour and plotted on the same graph. The time points of the early stationary phase, when β-galactosidase activity of the various fragments (L1–F) was measured and compared (Fig 4B–4D), are indicated by black (wild type) and dashed arrows (*hns* mutant) on the growth and β-galactosidase activity curves. The values represent the average of three independent assays. Standard errors are shown with error bars.
(PDF)

S3 Fig Raw sequencing results for 5’-RACE and the mapping positions of the 5’ edge of SE11 and SE15 *ybdO* mRNAs and transcription start site of K-12 mapped by differential RNA-seq. (A) Raw sequencing data for 5’-RACE. The 5’ edge position of each *ybdO* mRNA is denoted by an arrow. (B) The represents the region encompassing the *ybdO* transcription start site (indicated by an arrow) and promoter regions for each of SE11, SE15, and K-12 in the context of the alignment of *E*. *coli* genomes with the putative promoter sequence (the location of the putative -10 sequence is indicated by a red horizontal bar). This is a part of S1 Fig.
(PDF)

S4 Fig Scatter plots of the H-NS binding intensity as measured in duplicate experiments and with different strains. (A) Average H-NS binding intensity (logarithmic scale) in 200-bp windows was calculated at 100-bp steps along the whole genome to compare results obtained from duplicate experiments using scatter plots. (B) Average H-NS binding intensity (200-bp windows at 100-bp steps, logarithmic scale) along connected “common” segments was calculated to compare all combinations of ChAP-seq results. *r*: Pearson product-moment correlation coefficient.
(TIF)

S5 Fig Distribution of H-NS binding intensity. Distribution of H-NS binding intensity for all nucleotides in the *E*. *coli* genome obtained with ChAP-seq was assessed via Kernel density estimation using the R program with default parameters. Vertical axis values represent nucleotide density, with binding intensity [ChAP/WCE (log_10_)] shown on the horizontal axis. The mode value of the noise component and threshold value (mode + 0.6) to extract H-NS binding regions in each experiment are indicated.
(TIF)

S6 Fig Comparison of H-NS binding profiles in duplicate experiments. H-NS binding profiles in duplicate experiments are presented in CDS maps, which are the original H-NS binding profiles shown in Fig 1A, for SE11, SE15, and K-12. Overlapping binding regions in the two experiments are indicated with rectangles above the CDS maps.
(PDF)

S7 Fig H-NS binding profiles on whole “common” segments in SE11, SE15, and K-12. The H-NS binding profiles on the connected “common” segments in SE11, SE15, and K-12 are shown as for Fig 1C–1F.
(PDF)

S8 Fig Identification of orthologous genes. The bar graph shows the number of orthologous genes conserved in SE11, SE15, K-12, and the additional *E*. *coli* strains used in this study (see S1 Table). A total of 3,107 genes were conserved in >90% of strains (40 of 44, surrounded by a black rectangle) and were used as orthologous genes in this study. Among the selected 3107 orthologous proteins, the 405 orthologs encoded by genes that had at least one broken codon (with one or two nucleotide deletions or insertions) in at least one strain were excluded to remove pseudogenes. Ultimately, 2,702 orthologous protein clusters were selected for phylogenetic analysis.
(PDF)

S9 Fig Phylogenetic tree for 44 *E*. *coli* strains estimated by the ML method. The ML phylogenetic tree for 44 *E*. *coli* strains constructed via the concatenated superalignment of 100 randomly chosen orthologous genes. The reliability of the internal branches was assessed by bootstrapping with 100 pseudo-replicates. Strains used in ChAP-seq analysis are indicated with different colored underlines: blue, SE11; green, SE15; purple, K-12.
(PDF)

S10 Fig An example of how the H-NS binding depends on a strain-specific insertion sequence. There is a locus in which a specific sequence (*ytfI*, red arrow) is inserted into the chromosome (in this case, the K-12 chromosome), and H-NS binding to neighboring genes (in this case, *cpdB* [yellow], *cysQ* [green], *ytfJ* [blue] and *ytfK* [purple]) is observed (bottom panel). Without *ytfI*, H-NS binding to neighboring genes in SE11 and SE15 did not occur (top and middle panels).
(PDF)

S11 Fig Comparison of sequence diversities of H-NS-bound and -unbound orthologous genes determined using various definitions of “H-NS-bound” genes. Box plots were prepared as for Fig 2. (A) The same figures are shown as in Fig 2A and 2B. (B) Similar to (A), but orthologous genes in which H-NS bound only 10% of its gene length at the 3' end were regarded as “H-NS unbound” (red; H-NS bound, N = 474, gray; H-NS unbound, N = 2,228). (C) Similar to (A), but orthologous genes whose promoter sequence or the upstream region of its transcriptional unit was bound by H-NS were included as H-NS-bound genes (red; H-NS bound, N = 752, gray; H-NS unbound, N = 1,950). The asterisks indicate the statistical significance of the difference between the sequence diversities in the H-NS-bound and -unbound genes as assessed with the Wilcoxon rank-sum test (**p < 0.001, *p<0.05, ns: not significant).
(TIF)

S12 Fig Comparison of sets of trees for H-NS-bound and -unbound orthologs. Cumulative distributions of tree compatibility scores with the H-NS-unbound reference dataset. The *p*-values were calculated using the two-sided Kolmogorov-Smirnov test. Black dots: set C (H-NS-unbound); red dots: set B (H-NS-bound); blue dots: set D (H-NS-bound with random pruning and regrafting); green dots: set E (H-NS-bound with two rounds of random pruning and regrafting).
(PDF)

S13 Fig Sequence diversities of homologous genes and conserved intergenic regions that contain no gap sites in their alignments. Each distribution of sequence diversity is indicated as for Fig 3, but the orthologous genes and intergenic regions including gaps were excluded from the analysis. (A) Distribution of dN in the H-NS-bound (red; N = 159, median value = 0.0026) and -unbound (gray, N = 940, median value = 0.0019) genes. (B) Distribution of dS in the H-NS-bound (red, N = 159, median value = 0.054) and -unbound (gray, N = 940, median value = 0.058) genes. (C) Distribution of sequence diversity of H-NS-bound (red, N = 56, median value = 0.013) and -unbound (N = 458, median value = 0.0050) conserved intergenic regions. The asterisks indicate the statistical significance of the difference between the sequence diversities in the H-NS-bound and -unbound genes and intergenic regions as assessed with the Wilcoxon rank-sum test (*p < 0.05, ns: not significant).
(PDF)

S14 Fig Distribution of *E*. *coli* orthologous genes in Proteobacteria. For each gene cluster (columns), boxes indicate the presence (black) or absence (white) of genes in the corresponding genomes (rows). Left panel shows the reference phylogenetic tree for proteobacteria species computed using DnaK protein sequences of these species.
(PDF)

S15 Fig The relevance of evolutionary distance in class II intergenic regions and the presence (+known promoter) or absence (−known promoter) of known promoters. Each distribution of sequence diversity is indicated as for Fig 3. The information regarding promoters was acquired from the RegulonDB database [76]. Sequence diversity of H-NS-bound (+known promoter; N = 50, median value = 0.019) and -unbound (+known promoter; N = 267, median value = 0.0062) class II intergenic regions with known promoters (left) and of H-NS-bound (−known promoter; N = 30, median value = 0.014) and H-NS-unbound (−known promoter; N = 264, median value = 0.0043) class II intergenic regions without known promoters (right). The asterisks indicate the statistical significance of the difference between the sequence diversities in the H-NS-bound and -unbound genes and intergenic regions as assessed with the Wilcoxon rank-sum test (*p < 0.05, ns: not significant).
(TIF)

S1 Table *E*. *coli* strains applied to the phylogenetic analysis. (XLSX)

S2 Table Orthologous genes used in this study. (XLSX)

S3 Table Conserved intergenic regions in this study. (XLSX)

S4 Table List of primers used in this study. (XLSX)

S5 Table List of strains and plasmids used in this study. (XLSX)

S6 Table Summary of unique insertion sequences in the SE11, SE15, and K-12 chromosomes. (XLSX)

S7 Table List of strains used in the gene conservation analysis. (XLSX)
