# Supplementary material for: H-NS Facilitates Sequence Diversification of Horizontally Transferred DNAs during Their Integration in Host Chromosomes
Source: PLoS Genet. 2016 Jan 20;12(1):e1005796. doi: 10.1371/journal.pgen.1005796 (PMC4720273; doi:10.1371/journal.pgen.1005796)
Supplement: S1 Fig — (A) Schematic diagram of fragments used in the β-galactosidase assay. Shown is a multiple sequence alignment of ybdO including the upstream region for the E. coli strains. At the top of the first page, the locations of the 5’ and 3’ ends of the fragments are shown according to distance from the ybdO start codon. Blocks correspond to the regions that were truncated in shorter fragments cloned into the reporter plasmids for the β-galactosidase assay. The horizontal arrow denote the positions of transcription start sites suggested by 5’-RACE and differential RNA-seq [49]. The alignment of the ybdO promoter region and downstream region are shown below each schematic representation of blocks. In this analysis, we independently aligned coding and intergenic regions by different methods (see Materials and Methods). Therefore, we separately indicate the alignment in ortholog0270 (dsbG in K-12), intergenic region intergenic0112 (dsbG–ybdO), and ortholog2573 (ybdO). Numbers at the top of the alignment show the positions relative to the start codon of ybdO in K-12. The location of each block is indicated at the bottom of the alignment. Sequences for ortholog0270 and ortholog2573 were aligned using protein-based alignment, which was then back-translated to yield DNA sequences. Sequence alignment of the intergenic0112 was performed by the DNA-based alignment. The Alignment of ortholog0270 (dsbG) are constructed by sequences of all 44 E.coli strains, while alignments of intergenic0112(dsbG–ybdO) and ortholog2573 (ybdO) are constructed by 41 sequences. It is due to the fact that ybdO are conserved only in 41 E.coli strains and 3 strains do not possess ybdO ortholog. In these 3 strains, recombination or HGT event might have been occurred at the downstream of ortholog0270. Alignments are depicted by UGENE environment [78]. At positions 107 and 106 bp upstream of the ybdO initiation codon, we indicate the transcription start site for each of SE11, SE15, and K-12 ybdO. (B) Top panel: [file pgen.1005796.s001.pdf]

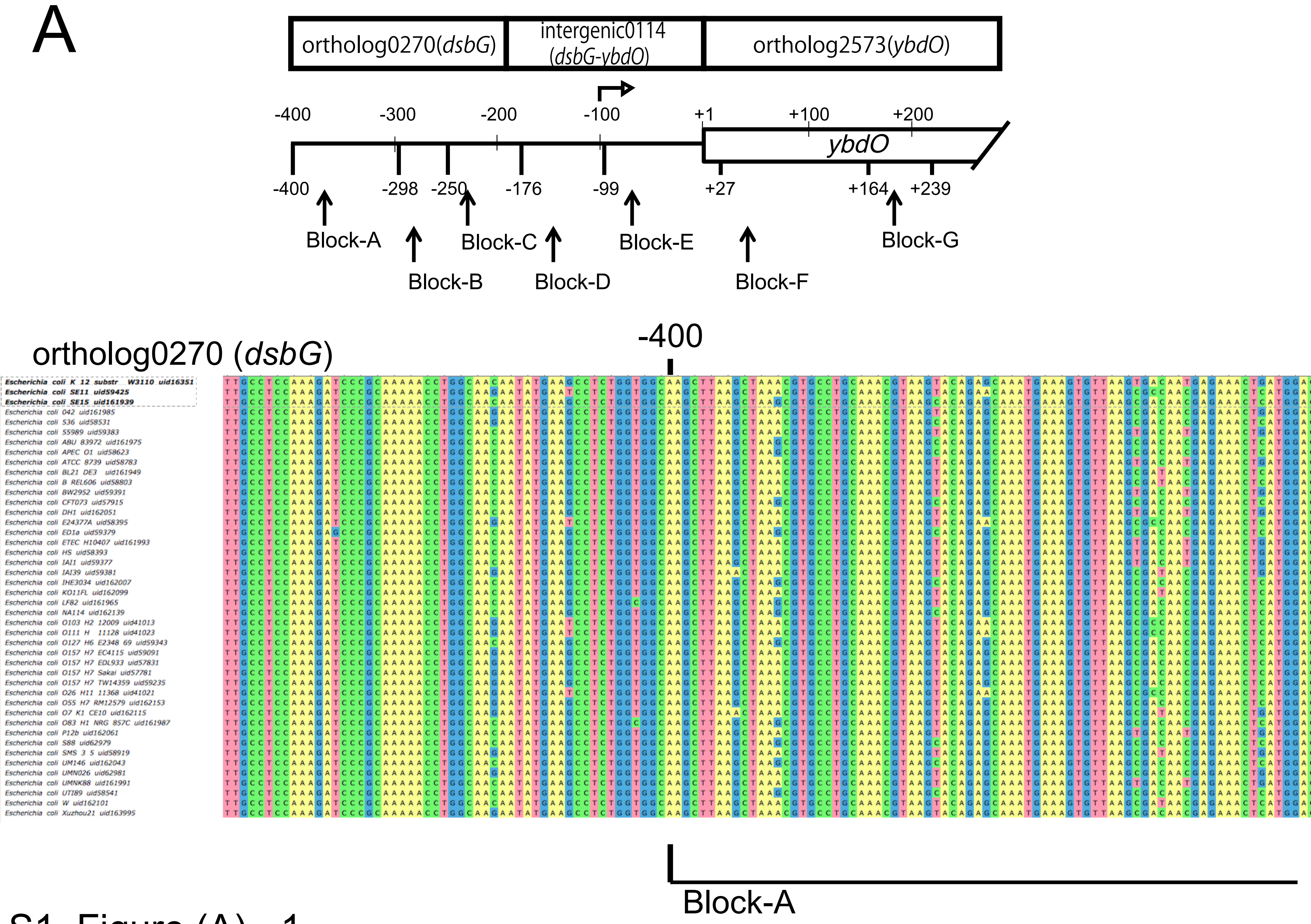
A
ortholog0270(dsbG)
intergenic0114 (dsbG-ybdO)
ortholog2573(ybdO)
-400
-300
-200
-100
+1
+100
+200
ybdO
-400
-298
-250
-176
-99
+27
+164
+239
Block-A
Block-B
Block-C
Block-D
Block-E
Block-F
Block-G
ortholog0270 (dsbG)
-400
Block-A

S1_Figure (A) - 1

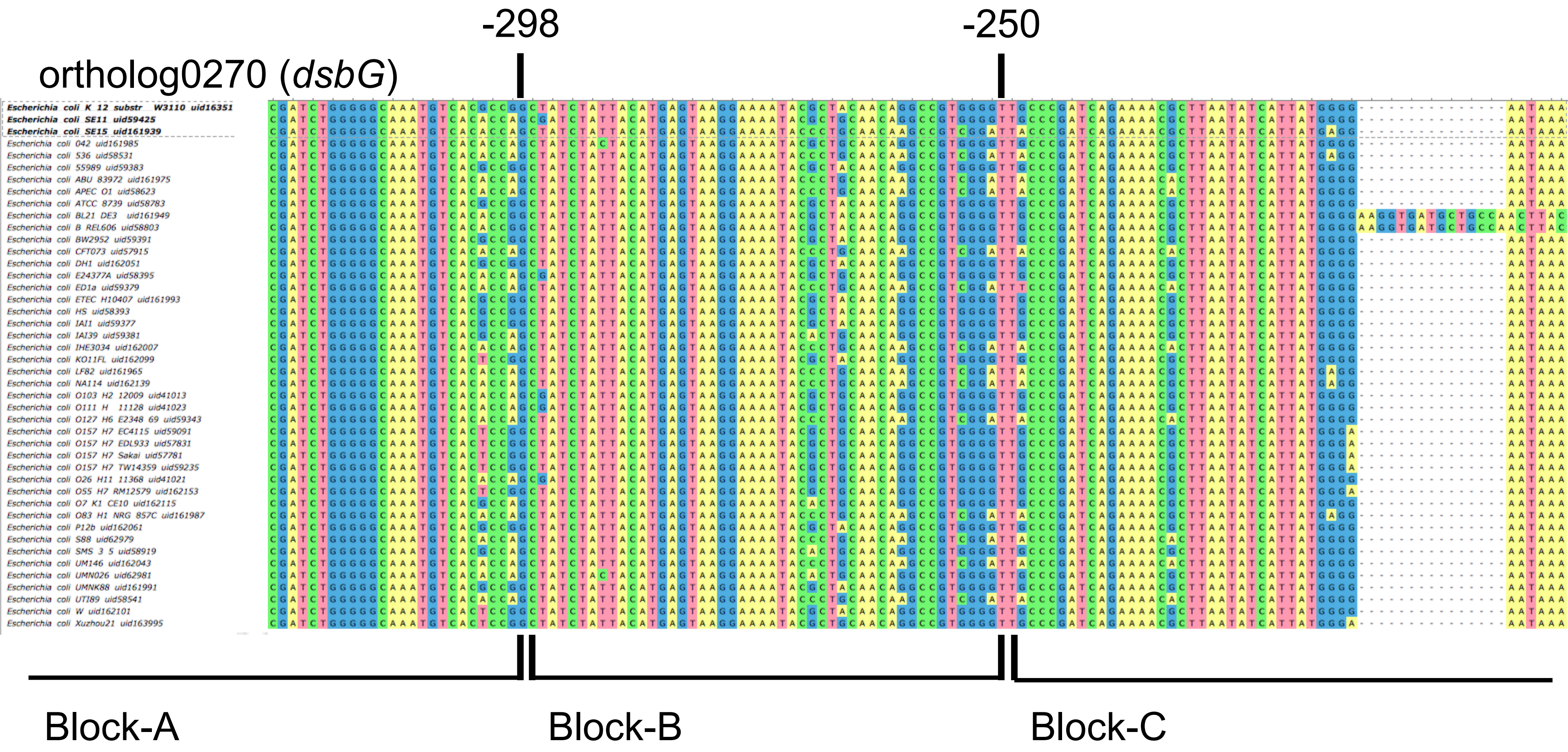

S1_Figure (A) - 2

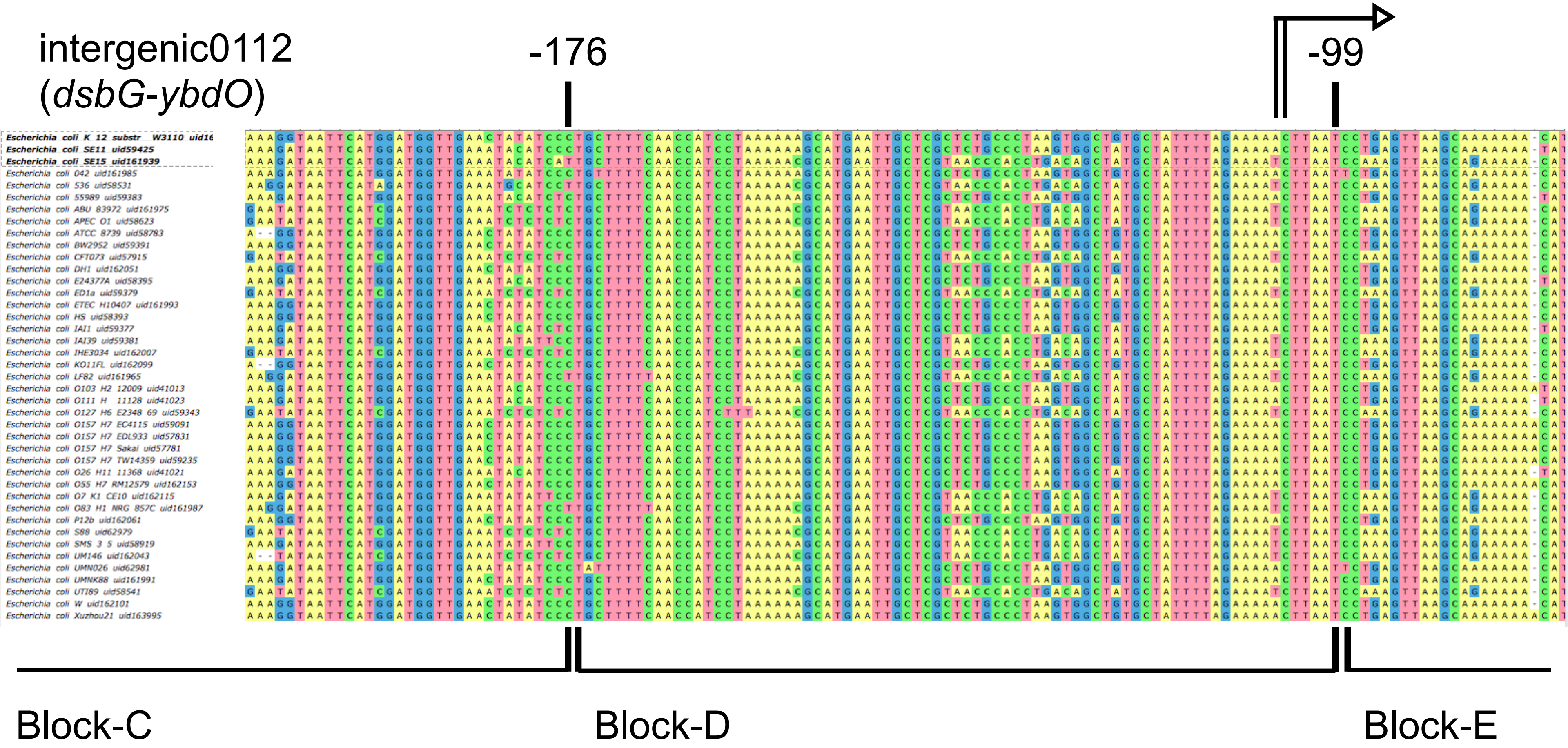

S1_Figure (A) - 3

intergenic0112
(*dsbG-ybdO*)

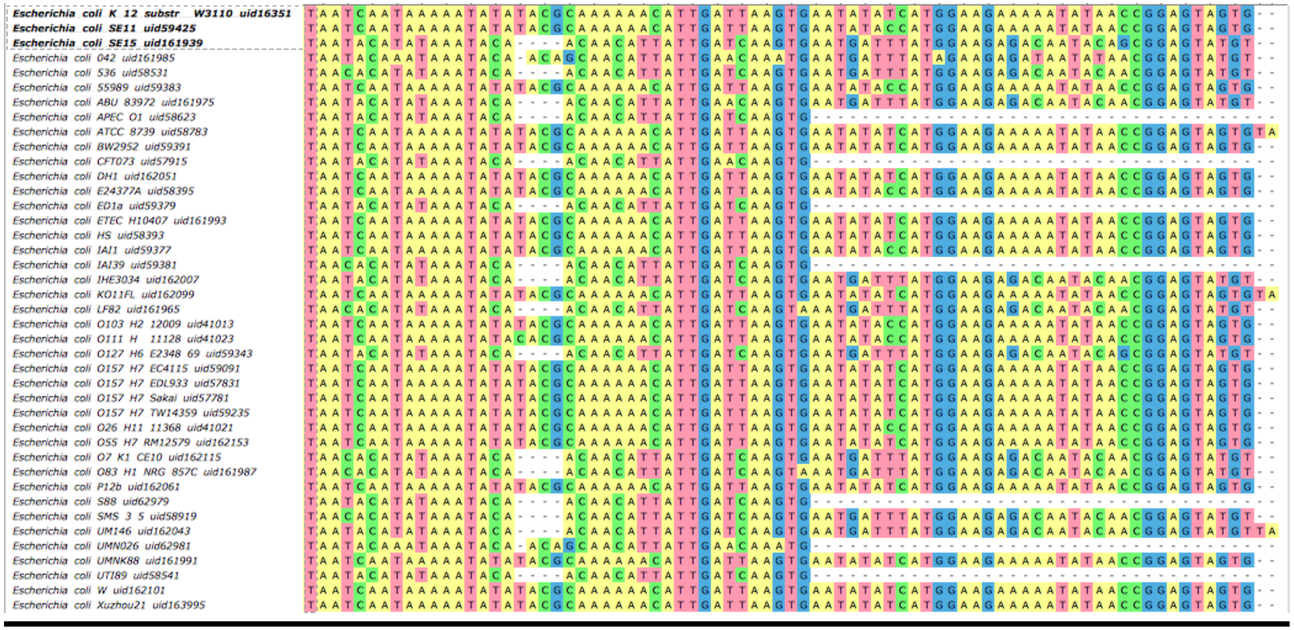

Block-E

S1_Figure (A) - 4

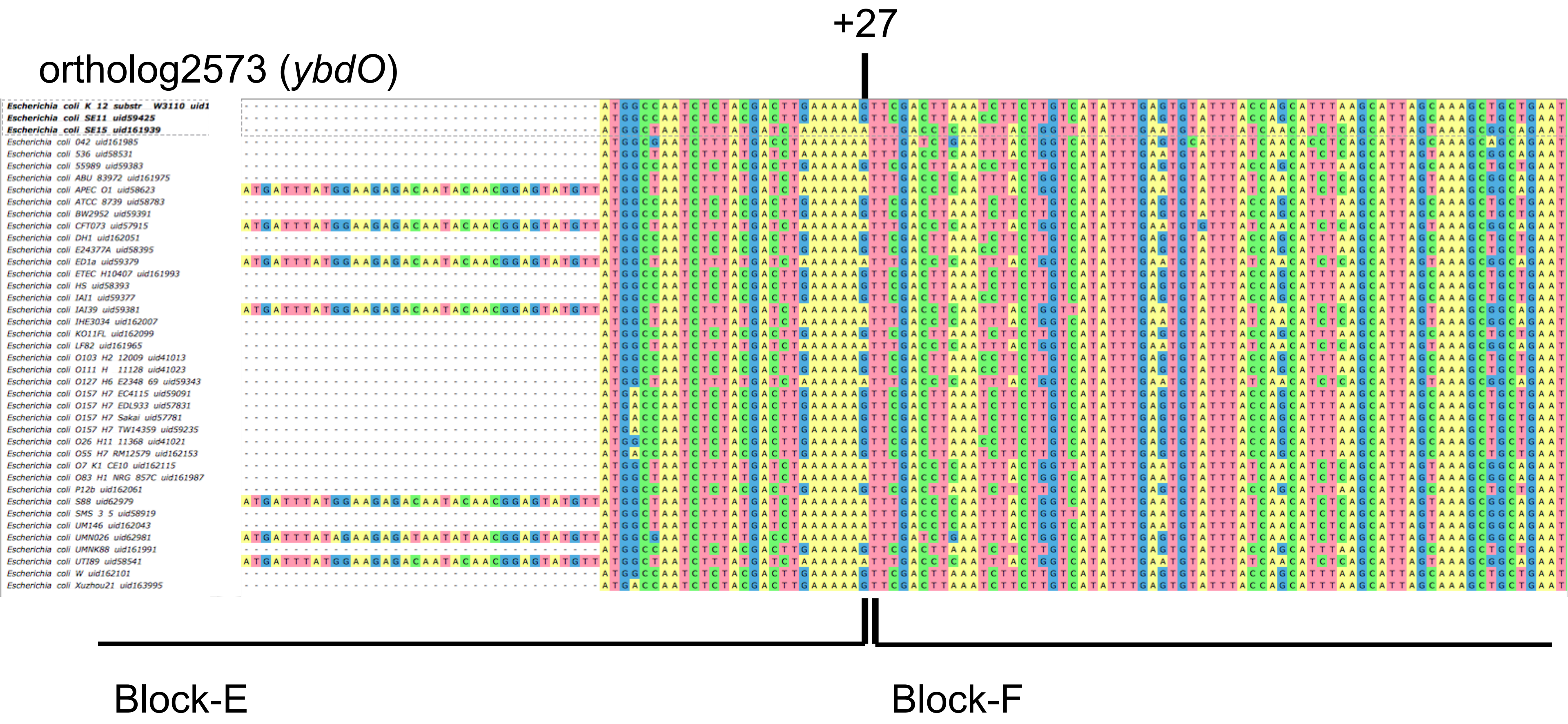

S1_Figure (A) - 5

ortholog2573 (*ybdO*)

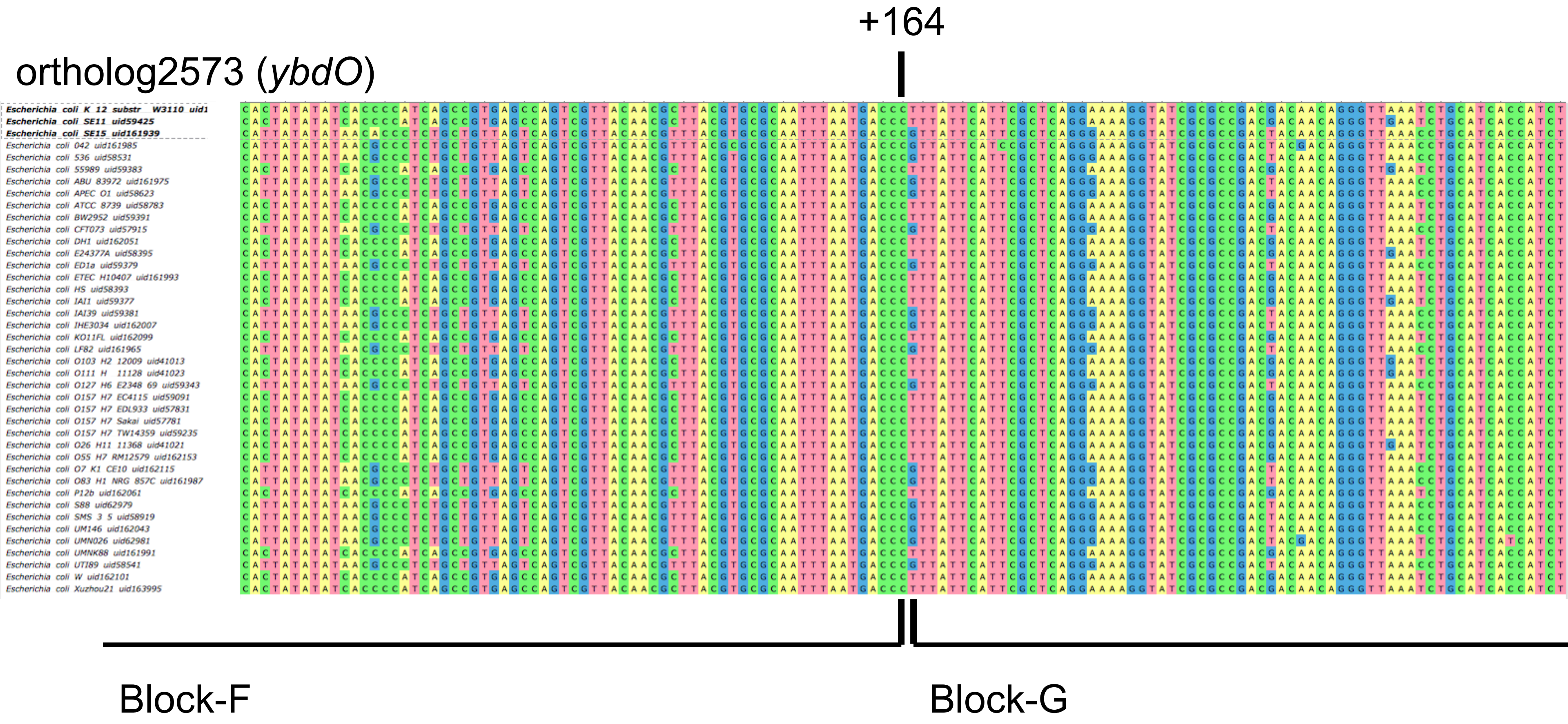

S1_Figure (A) - 6

ortholog2573 (*ybdO*)

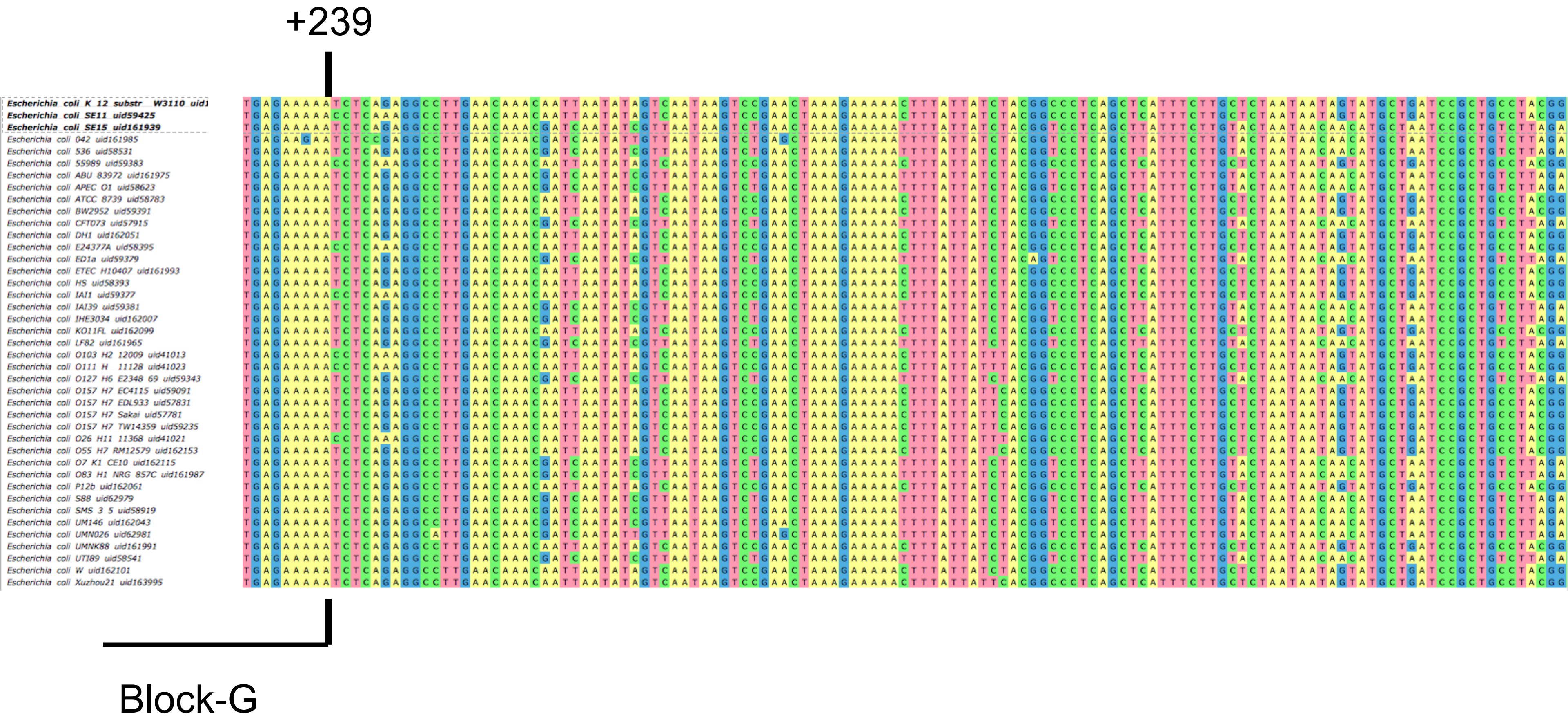

S1_Figure (A) - 7

B

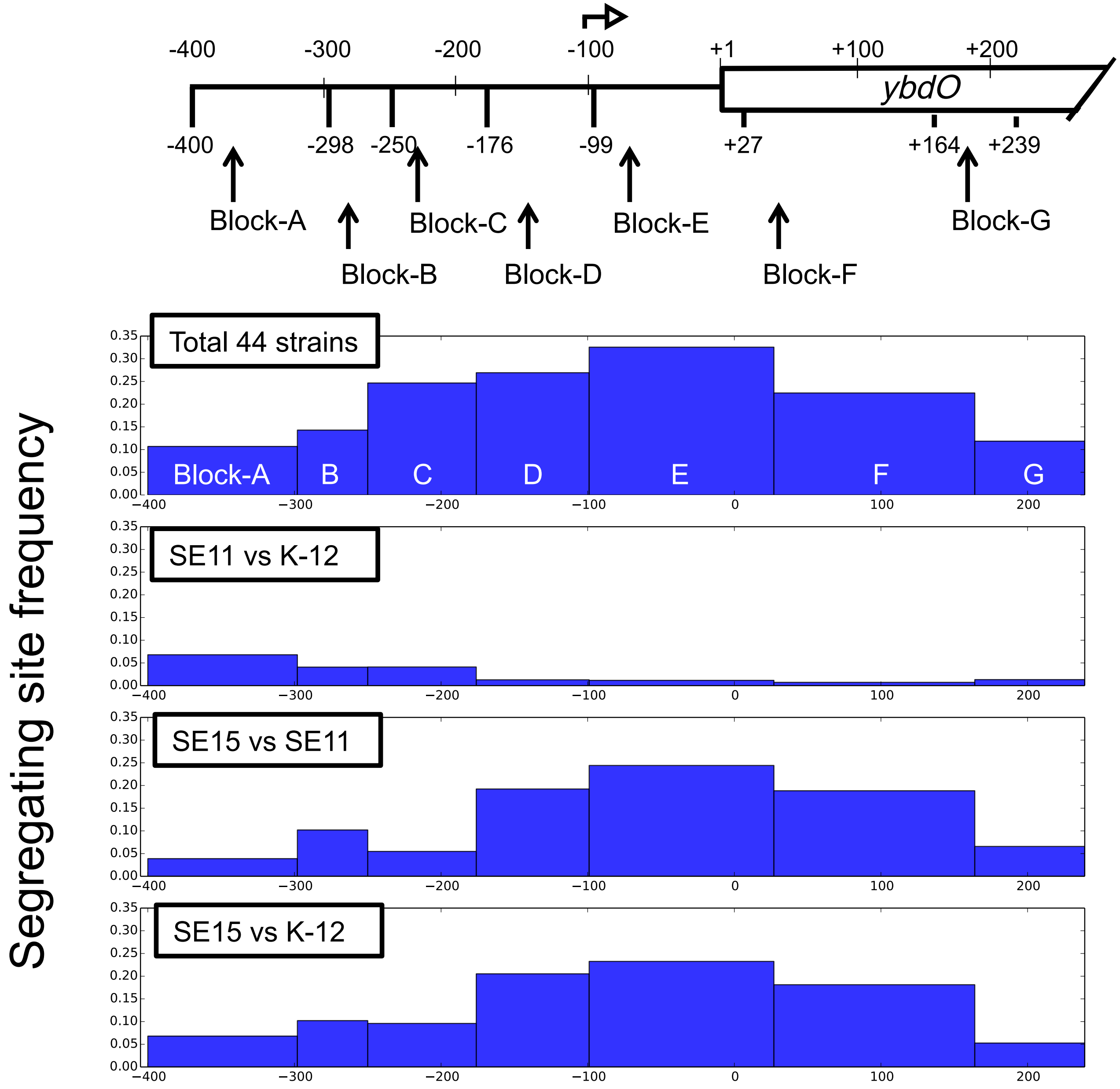

-400
-300
-200
-100
+1
+100
+200
ybdO
-400
-298
-250
-176
-99
+27
+164
+239
Block-A
Block-B
Block-C
Block-D
Block-E
Block-F
Block-G
Segregating site frequency
Total 44 strains
SE11 vs K-12
SE15 vs SE11
SE15 vs K-12
Block-A
B
C
D
E
F
G
0.35
0.30
0.25
0.20
0.15
0.10
0.05
0.00
−400
−300
−200
−100
0
100
200
