# Supplementary figures and images for: H-NS Facilitates Sequence Diversification of Horizontally Transferred DNAs during Their Integration in Host Chromosomes
Source: PLoS Genet. 2016 Jan 20;12(1):e1005796. doi: 10.1371/journal.pgen.1005796 (PMC4720273; doi:10.1371/journal.pgen.1005796)

A

SE11

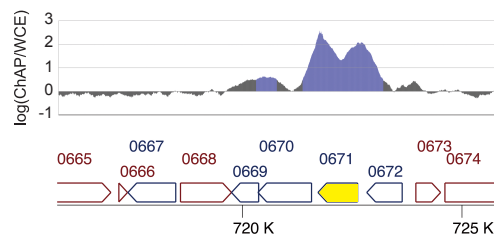

SE15

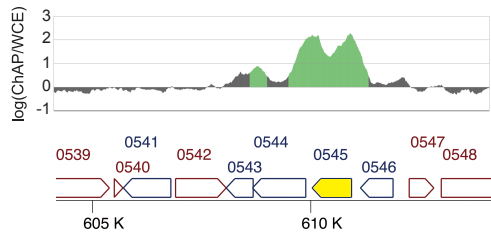

K12

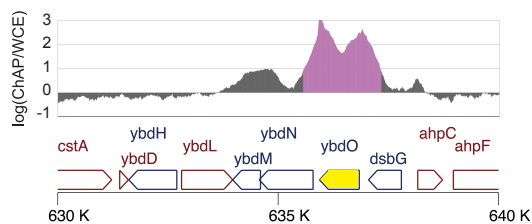

B

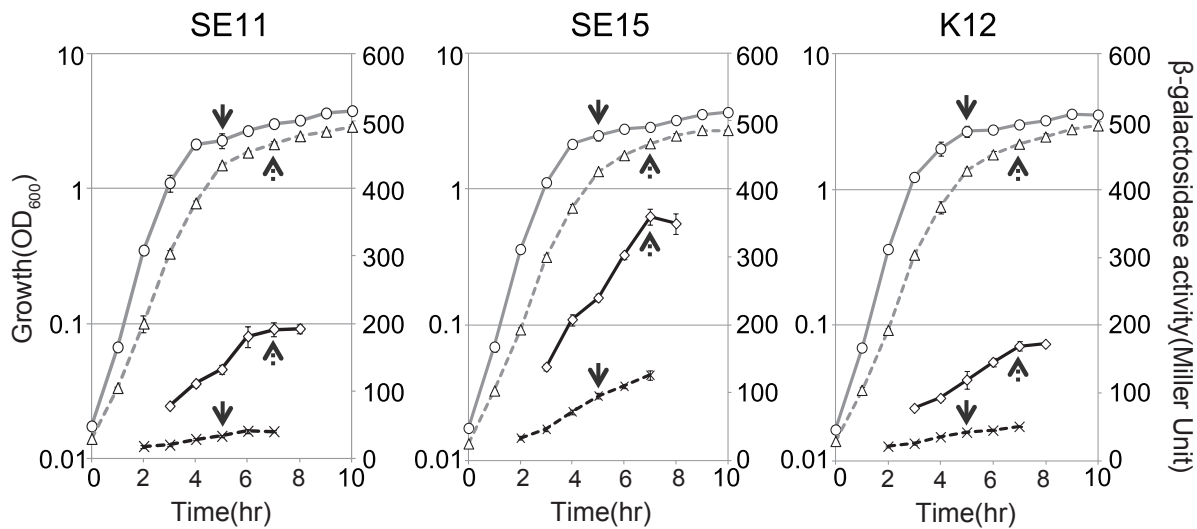

Supplement: S2 Fig — (A) H-NS binding profiles near ybdO are presented with CDS maps for SE11 (top), SE15 (middle), and K-12 (bottom), which are segments of the maps in S2 Fig. The yellow arrows show the locations of ybdO in K-12, SE11, and SE15. (B) Expression profiles of the SE11, SE15, and K-12 ybdO promoters in the time course. The wild type (MC4100) and the hns mutant (MC4100 Δhns::km) transformed with pRW derivatives carrying the L2 fragments of SE11 (left), SE15 (middle), and K-12 (right) were grown at 37°C in LB medium under aerobic conditions. The optical density (OD600) of the wild-type (open circles with black line) and hns mutant (open triangles with dashed black line) cultures and the β-galactosidase activities (Miller units) of the wild type (cross with bold dashed line) and the hns mutant (open diamond with bold black line) were measured every hour and plotted on the same graph. The time points of the early stationary phase, when β-galactosidase activity of the various fragments (L1–F) was measured and compared (Fig 4B–4D), are indicated by black (wild type) and dashed arrows (hns mutant) on the growth and β-galactosidase activity curves. The values represent the average of three independent assays. Standard errors are shown with error bars. (PDF) [file pgen.1005796.s002.pdf]

# A

## SE11

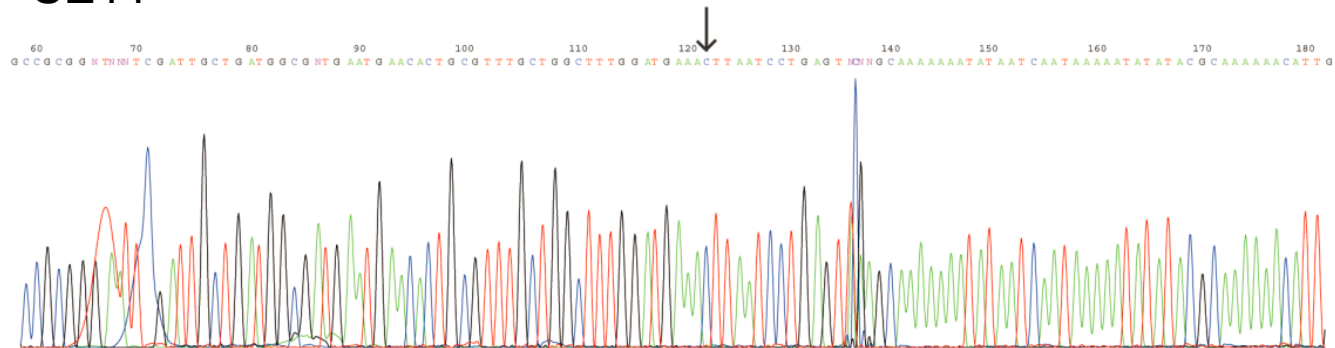

## SE15

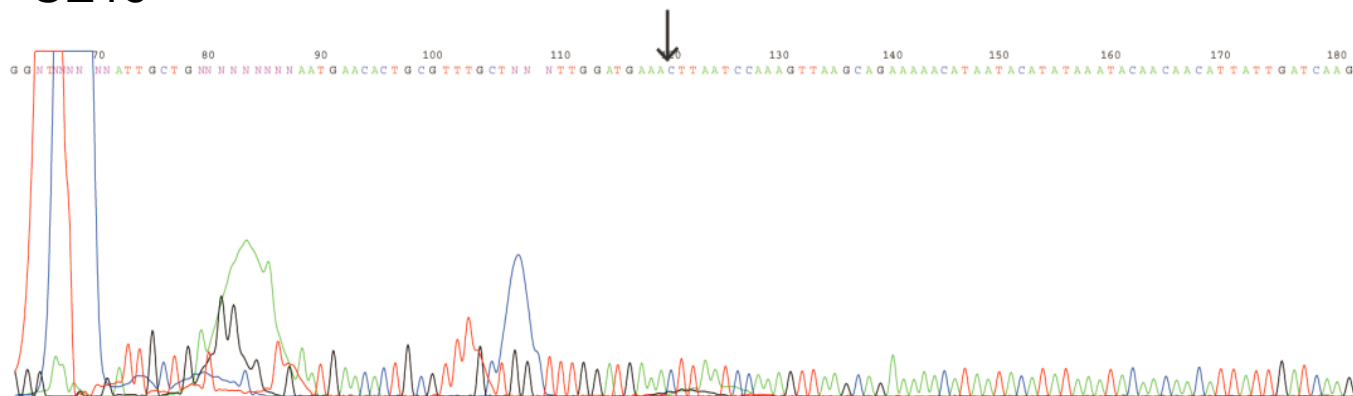

# B

## intergenic0112 (*dsbG-ybdO*)

-176

-10

-99

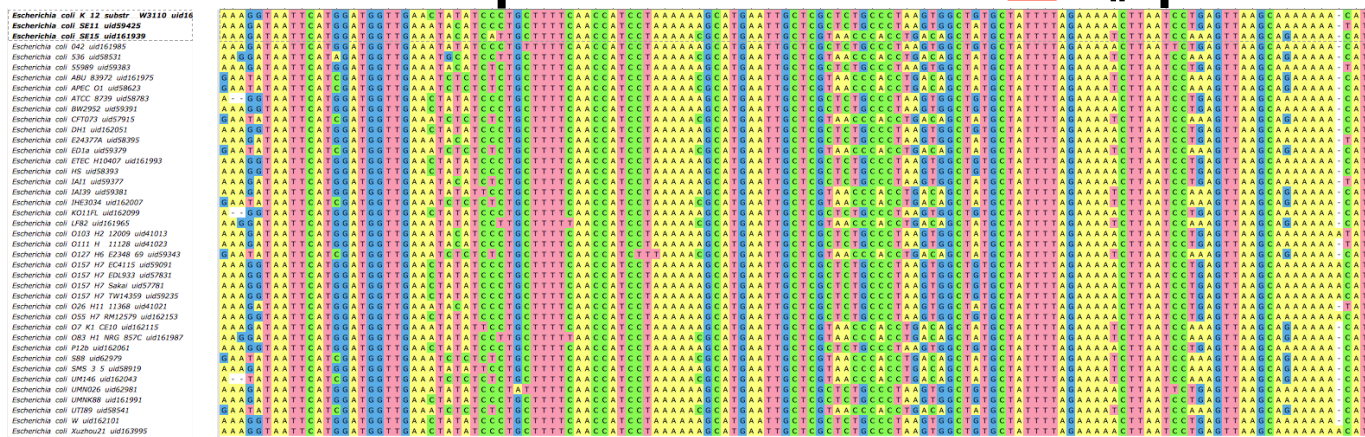

Supplement: S3 Fig — (A) Raw sequencing data for 5’-RACE. The 5’ edge position of each ybdO mRNA is denoted by an arrow. (B) The represents the region encompassing the ybdO transcription start site (indicated by an arrow) and promoter regions for each of SE11, SE15, and K-12 in the context of the alignment of E. coli genomes with the putative promoter sequence (the location of the putative -10 sequence is indicated by a red horizontal bar). This is a part of S1 Fig. (PDF) [file pgen.1005796.s003.pdf]

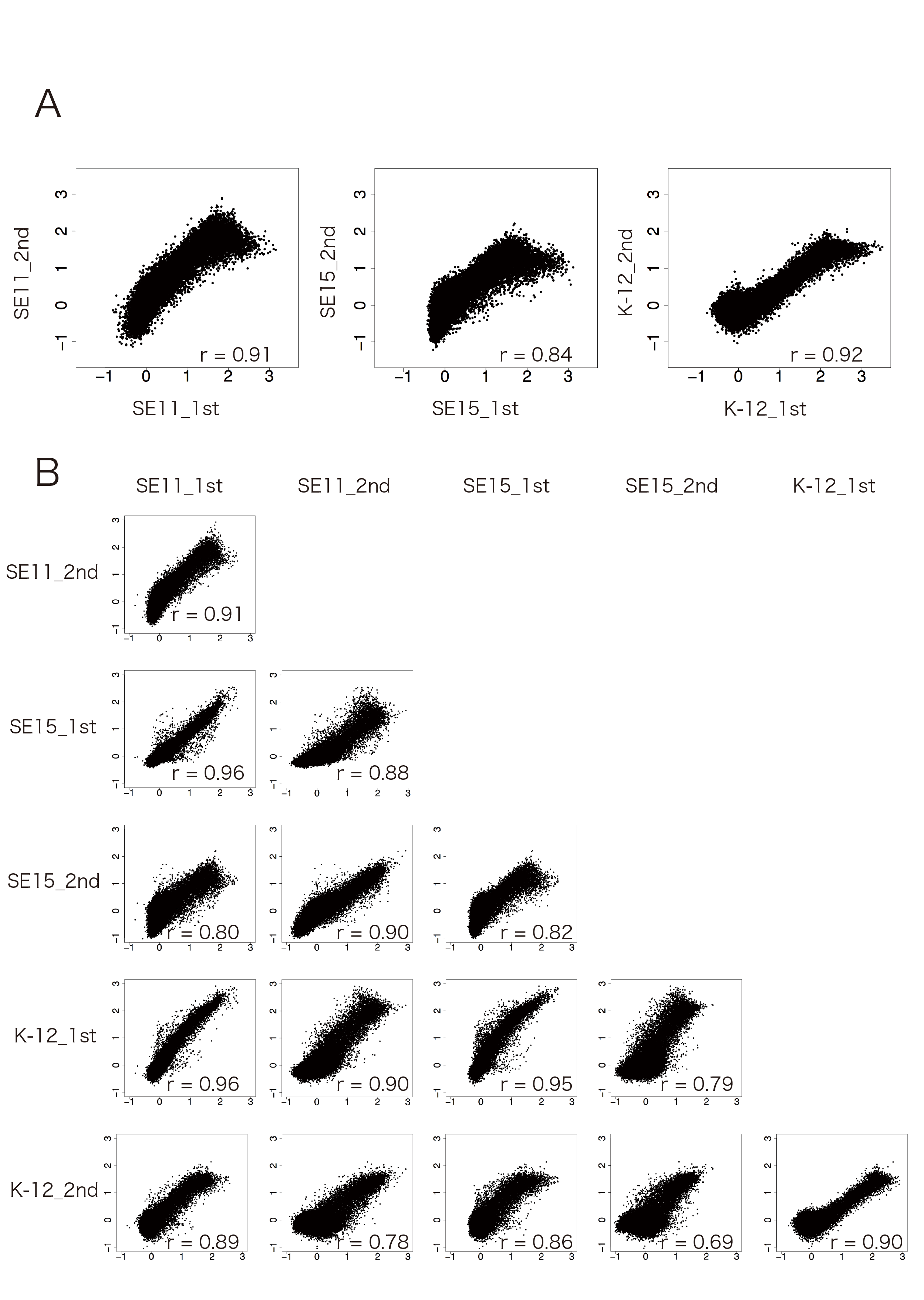

Supplement: S4 Fig — (A) Average H-NS binding intensity (logarithmic scale) in 200-bp windows was calculated at 100-bp steps along the whole genome to compare results obtained from duplicate experiments using scatter plots. (B) Average H-NS binding intensity (200-bp windows at 100-bp steps, logarithmic scale) along connected “common” segments was calculated to compare all combinations of ChAP-seq results. r: Pearson product-moment correlation coefficient. (TIF) [file pgen.1005796.s004.tif]

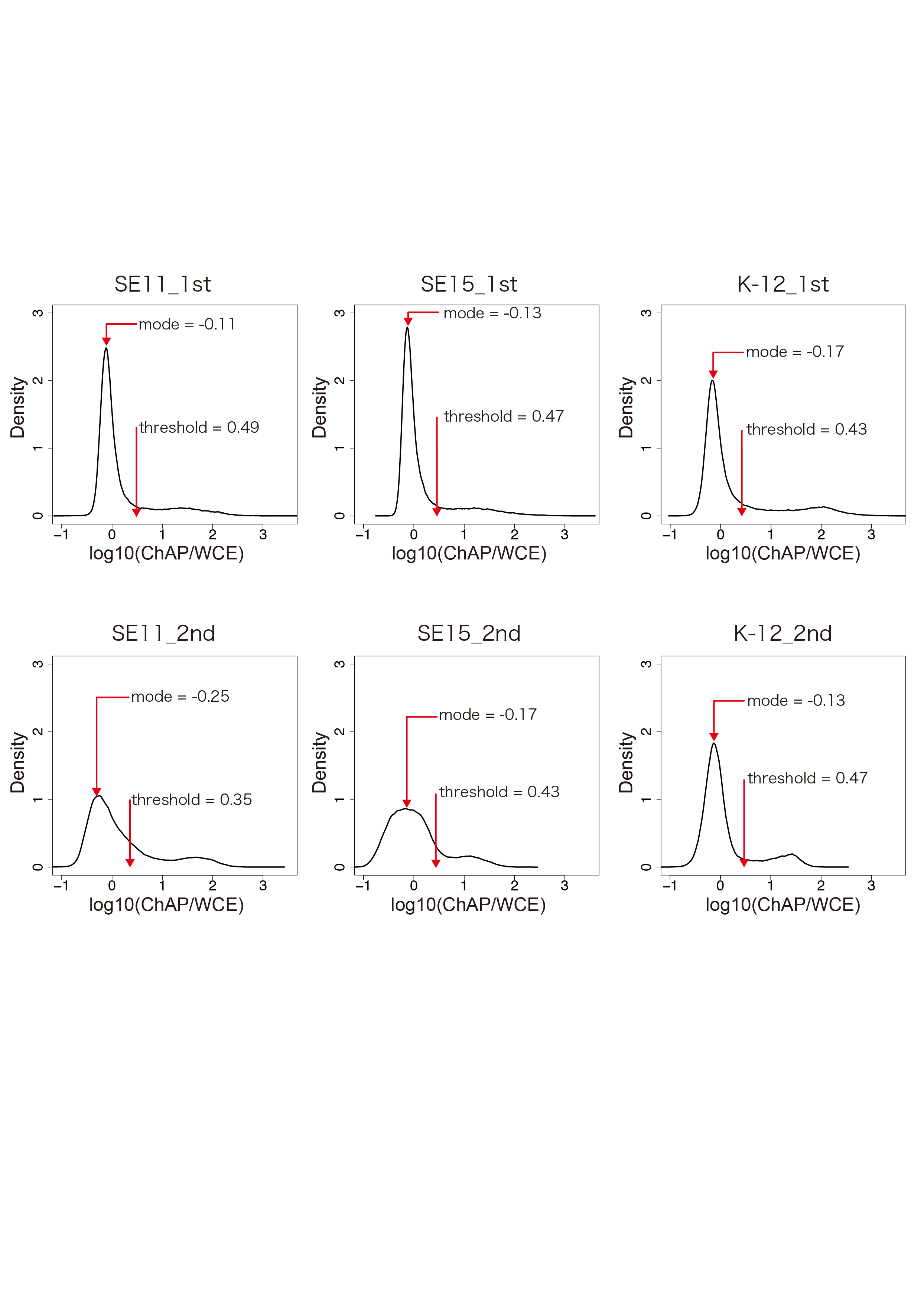

Supplement: S5 Fig — Distribution of H-NS binding intensity for all nucleotides in the E. coli genome obtained with ChAP-seq was assessed via Kernel density estimation using the R program with default parameters. Vertical axis values represent nucleotide density, with binding intensity [ChAP/WCE (log10)] shown on the horizontal axis. The mode value of the noise component and threshold value (mode + 0.6) to extract H-NS binding regions in each experiment are indicated. (TIF) [file pgen.1005796.s005.tif]

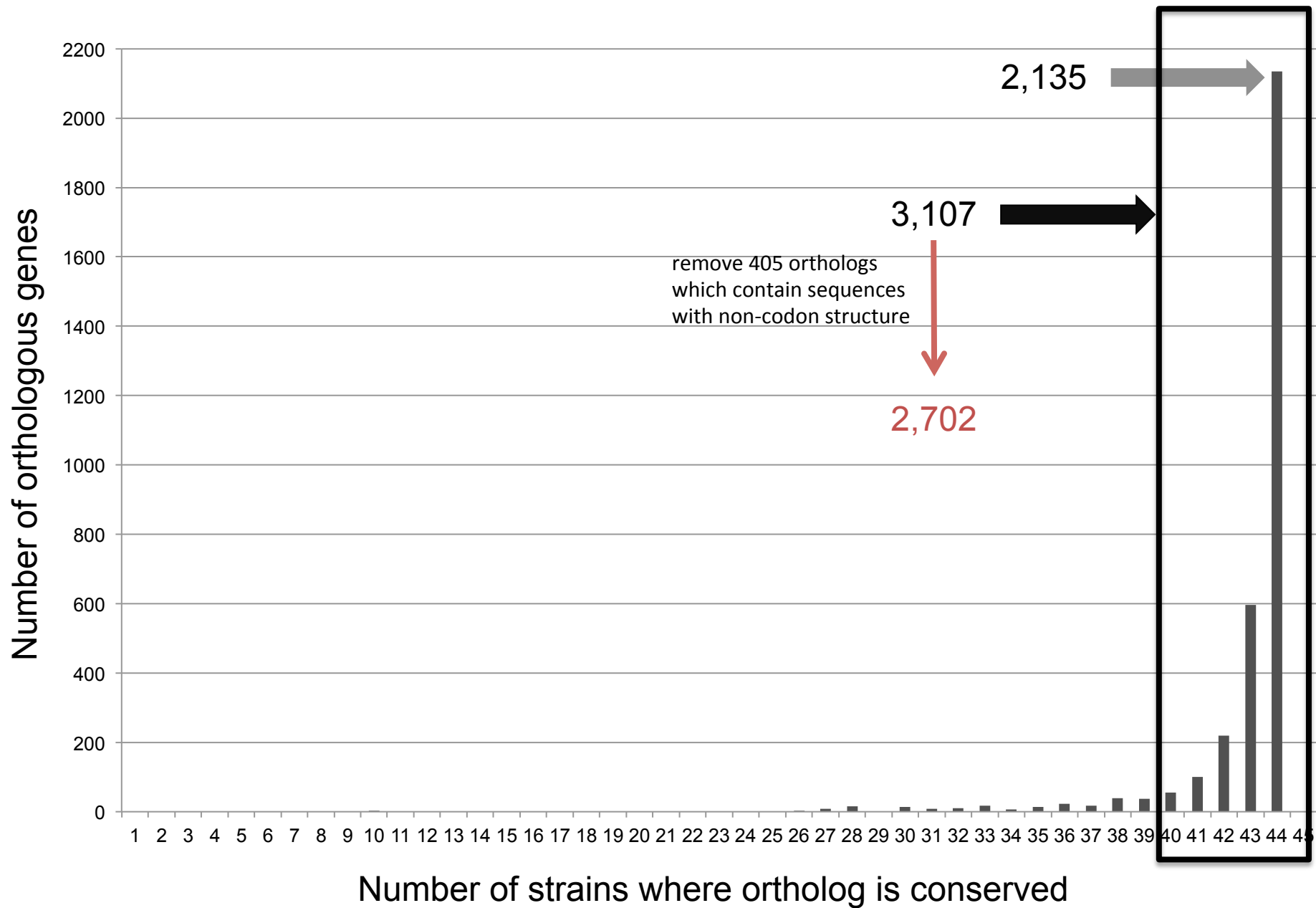

Supplement: S8 Fig — The bar graph shows the number of orthologous genes conserved in SE11, SE15, K-12, and the additional E. coli strains used in this study (see S1 Table). A total of 3,107 genes were conserved in >90% of strains (40 of 44, surrounded by a black rectangle) and were used as orthologous genes in this study. Among the selected 3107 orthologous proteins, the 405 orthologs encoded by genes that had at least one broken codon (with one or two nucleotide deletions or insertions) in at least one strain were excluded to remove pseudogenes. Ultimately, 2,702 orthologous protein clusters were selected for phylogenetic analysis. (PDF) [file pgen.1005796.s008.pdf]

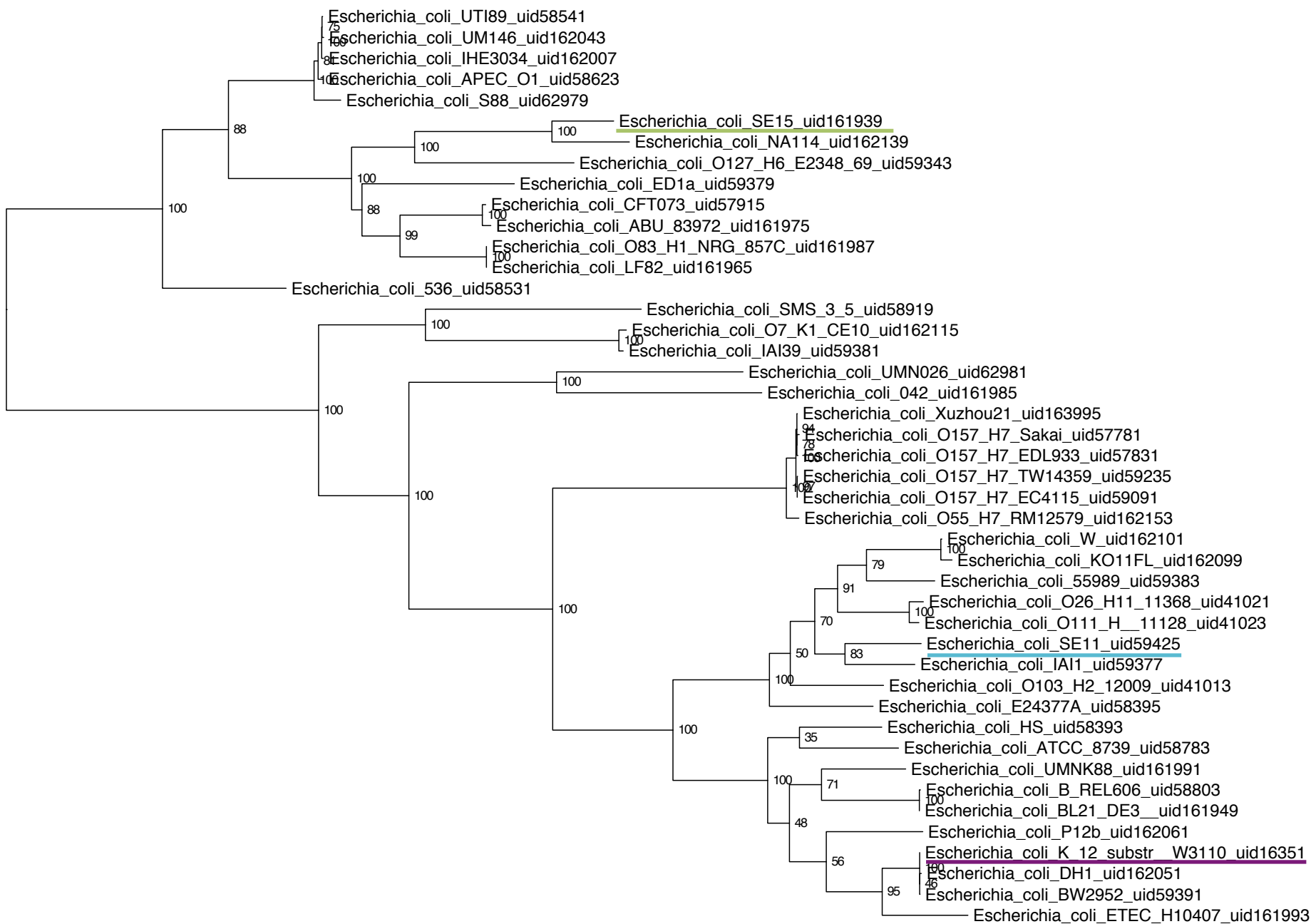

0.0050

Supplement: S9 Fig — The ML phylogenetic tree for 44 E. coli strains constructed via the concatenated superalignment of 100 randomly chosen orthologous genes. The reliability of the internal branches was assessed by bootstrapping with 100 pseudo-replicates. Strains used in ChAP-seq analysis are indicated with different colored underlines: blue, SE11; green, SE15; purple, K-12. (PDF) [file pgen.1005796.s009.pdf]

SE11

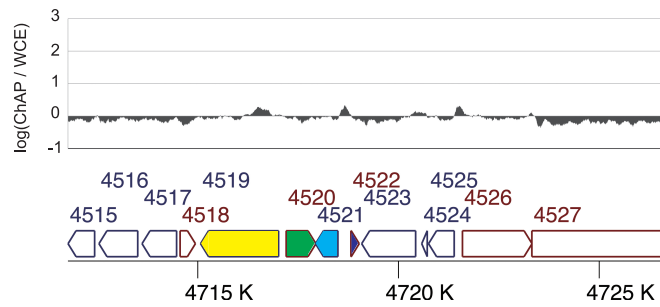

SE15

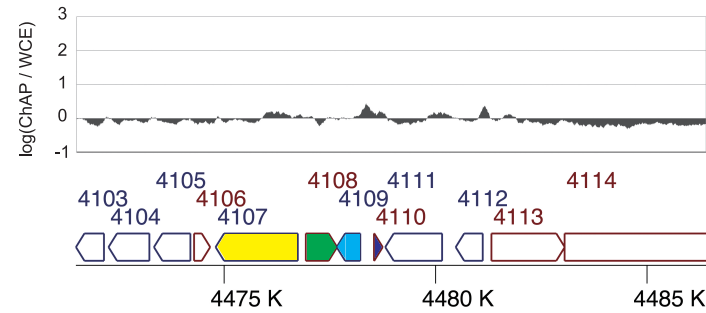

K-12

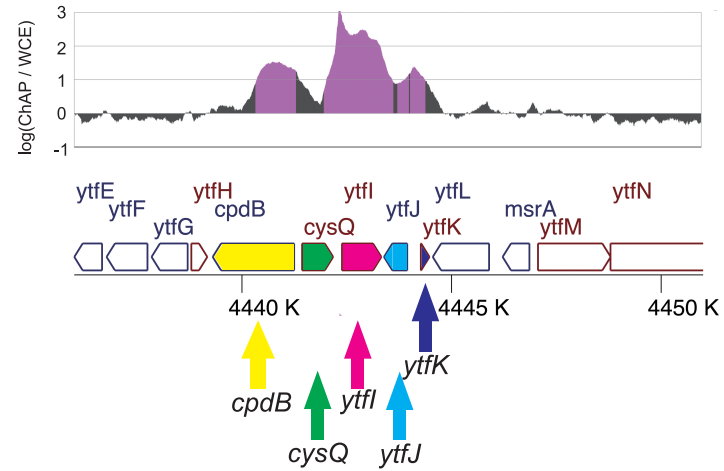

Supplement: S10 Fig — There is a locus in which a specific sequence (ytfI, red arrow) is inserted into the chromosome (in this case, the K-12 chromosome), and H-NS binding to neighboring genes (in this case, cpdB [yellow], cysQ [green], ytfJ [blue] and ytfK [purple]) is observed (bottom panel). Without ytfI, H-NS binding to neighboring genes in SE11 and SE15 did not occur (top and middle panels). (PDF) [file pgen.1005796.s010.pdf]

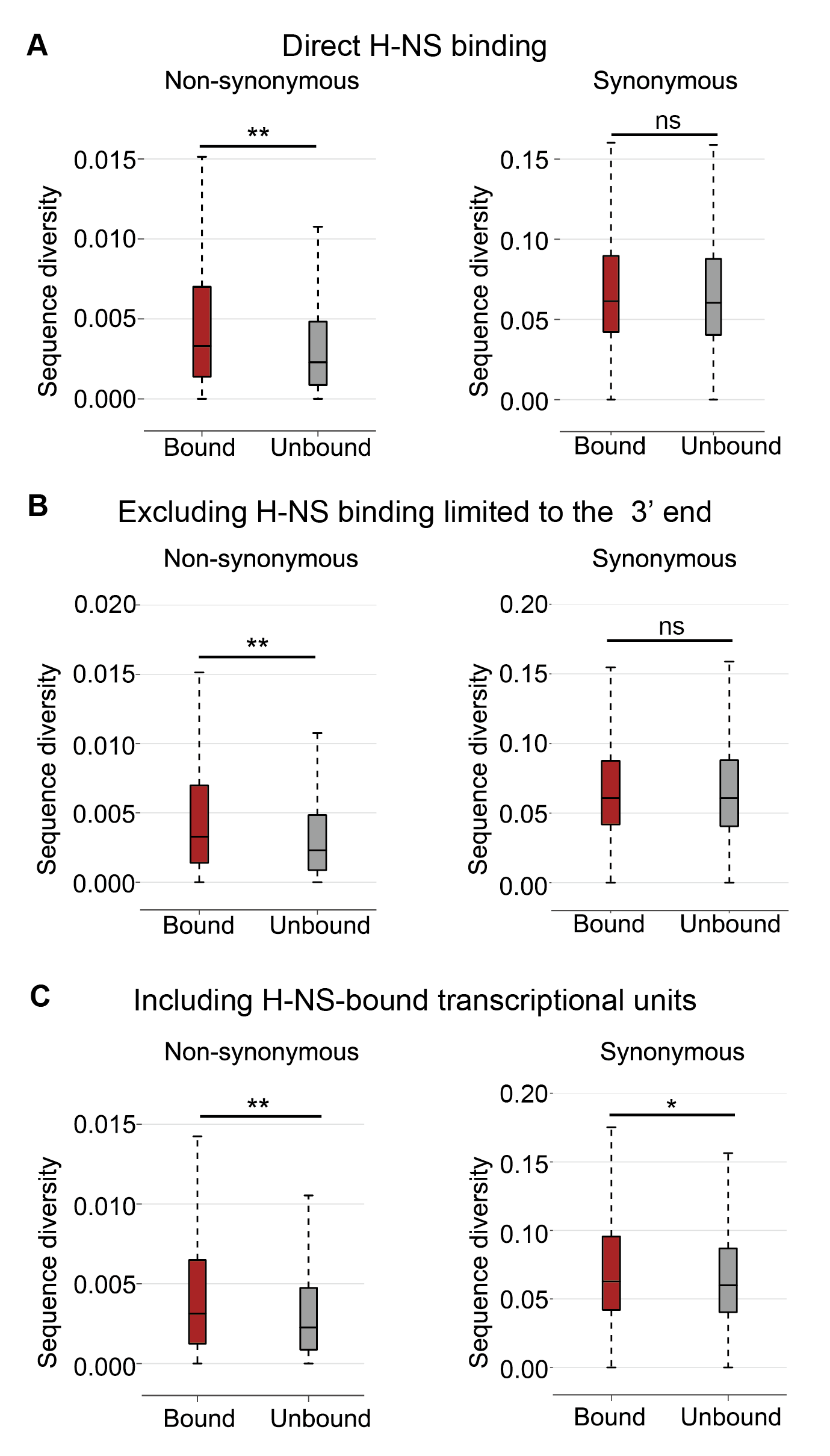

Supplement: S11 Fig — Box plots were prepared as for Fig 2. (A) The same figures are shown as in Fig 2A and 2B. (B) Similar to (A), but orthologous genes in which H-NS bound only 10% of its gene length at the 3' end were regarded as “H-NS unbound” (red; H-NS bound, N = 474, gray; H-NS unbound, N = 2,228). (C) Similar to (A), but orthologous genes whose promoter sequence or the upstream region of its transcriptional unit was bound by H-NS were included as H-NS-bound genes (red; H-NS bound, N = 752, gray; H-NS unbound, N = 1,950). The asterisks indicate the statistical significance of the difference between the sequence diversities in the H-NS-bound and -unbound genes as assessed with the Wilcoxon rank-sum test (**p < 0.001, *p<0.05, ns: not significant). (TIF) [file pgen.1005796.s011.tif]

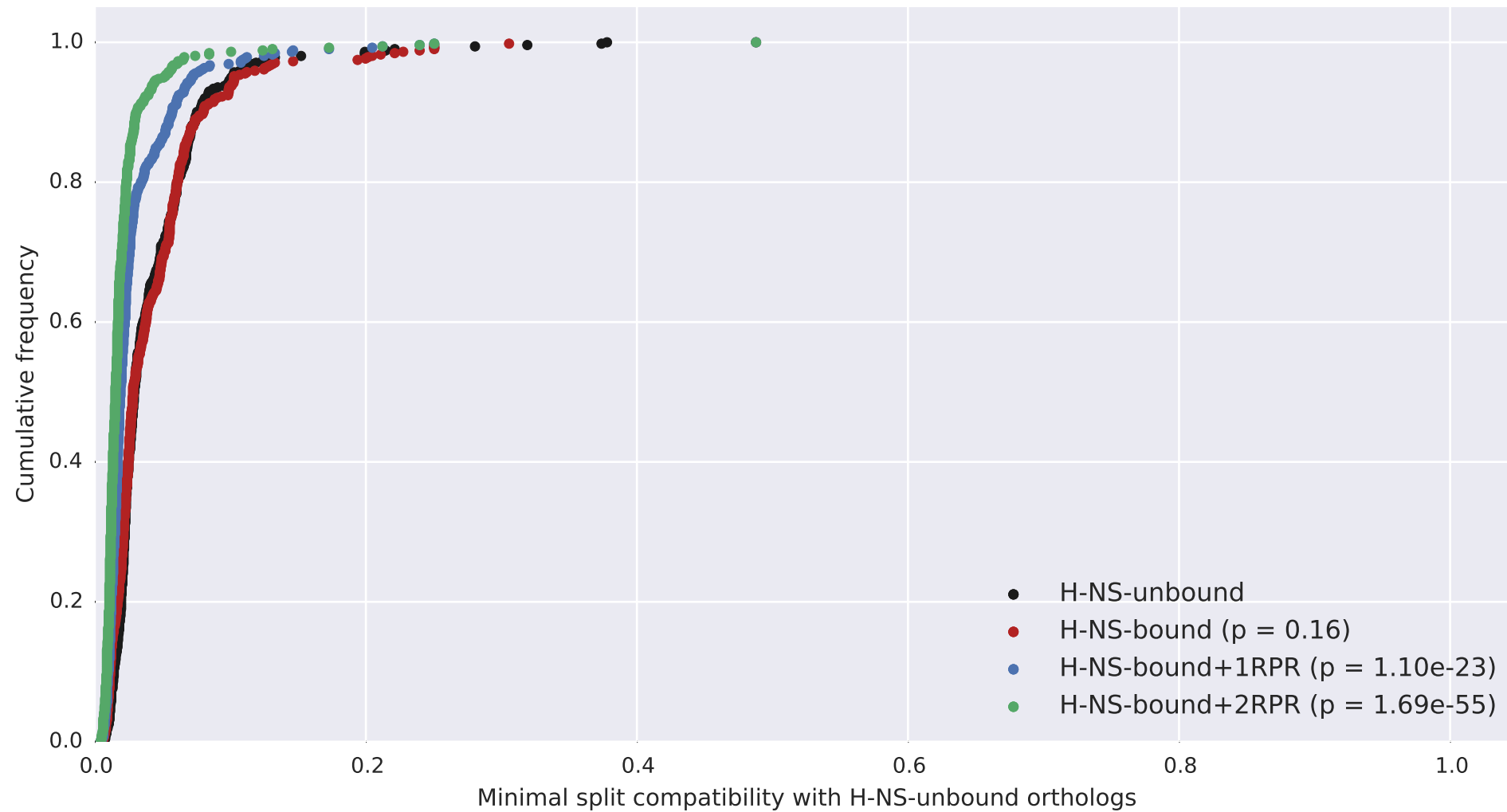

Supplement: S12 Fig — Cumulative distributions of tree compatibility scores with the H-NS-unbound reference dataset. The p-values were calculated using the two-sided Kolmogorov-Smirnov test. Black dots: set C (H-NS-unbound); red dots: set B (H-NS-bound); blue dots: set D (H-NS-bound with random pruning and regrafting); green dots: set E (H-NS-bound with two rounds of random pruning and regrafting). (PDF) [file pgen.1005796.s012.pdf]

**A**

Non-synonymous

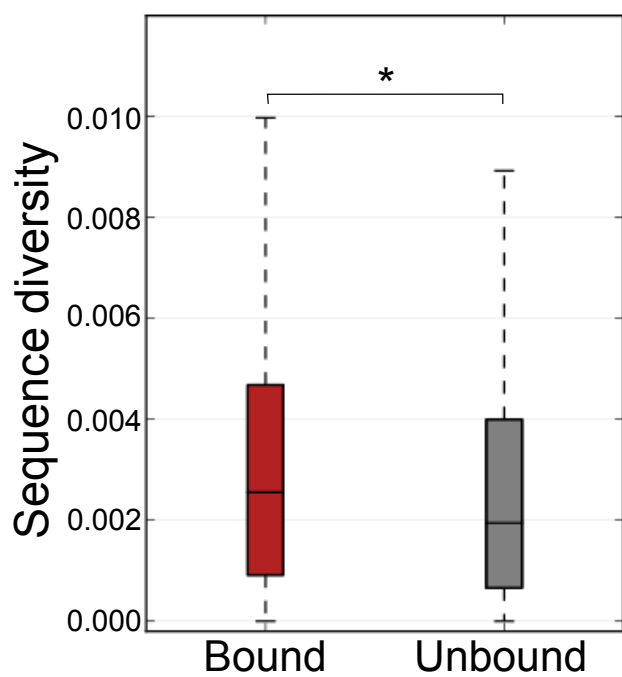**B**

Synonymous

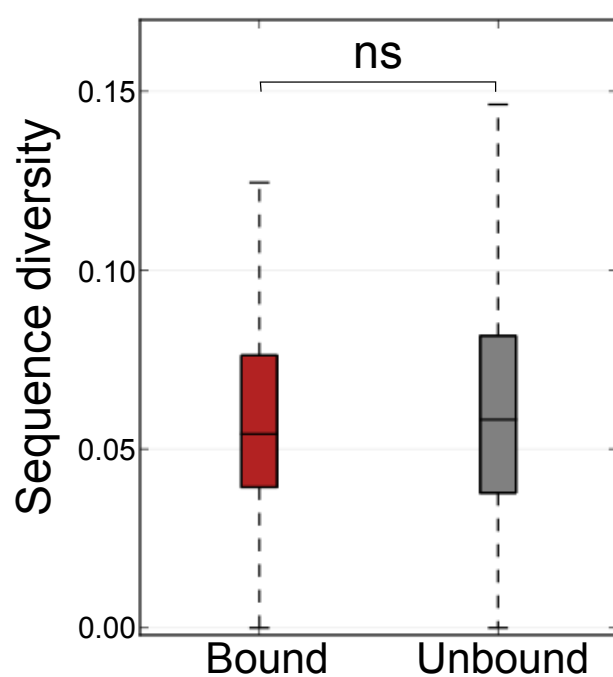**C**

Intergenic

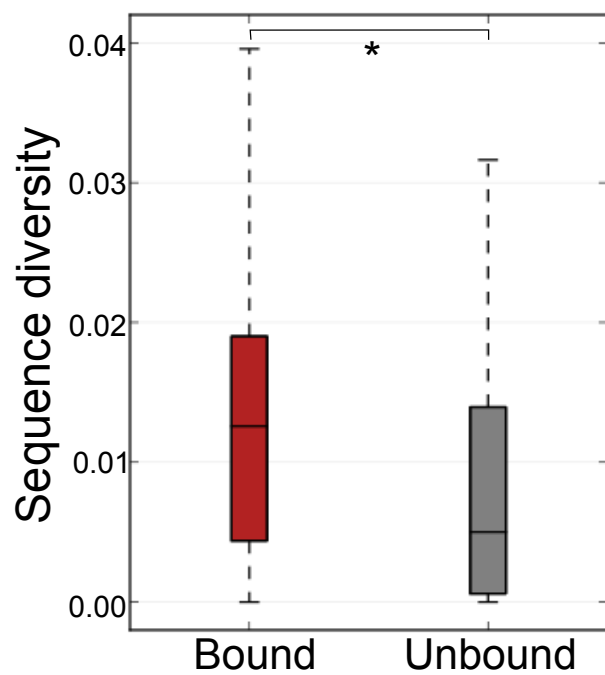

Supplement: S13 Fig — Each distribution of sequence diversity is indicated as for Fig 3, but the orthologous genes and intergenic regions including gaps were excluded from the analysis. (A) Distribution of dN in the H-NS-bound (red; N = 159, median value = 0.0026) and -unbound (gray, N = 940, median value = 0.0019) genes. (B) Distribution of dS in the H-NS-bound (red, N = 159, median value = 0.054) and -unbound (gray, N = 940, median value = 0.058) genes. (C) Distribution of sequence diversity of H-NS-bound (red, N = 56, median value = 0.013) and -unbound (N = 458, median value = 0.0050) conserved intergenic regions. The asterisks indicate the statistical significance of the difference between the sequence diversities in the H-NS-bound and -unbound genes and intergenic regions as assessed with the Wilcoxon rank-sum test (*p < 0.05, ns: not significant). (PDF) [file pgen.1005796.s013.pdf]

H-NS-bound  
HGT-genes  
(N = 157)

H-NS-unbound  
HGT-genes  
(N = 224)

H-NS-bound  
Core-genes  
(N = 174)

H-NS-unbound  
Core-genes  
(N = 1543)

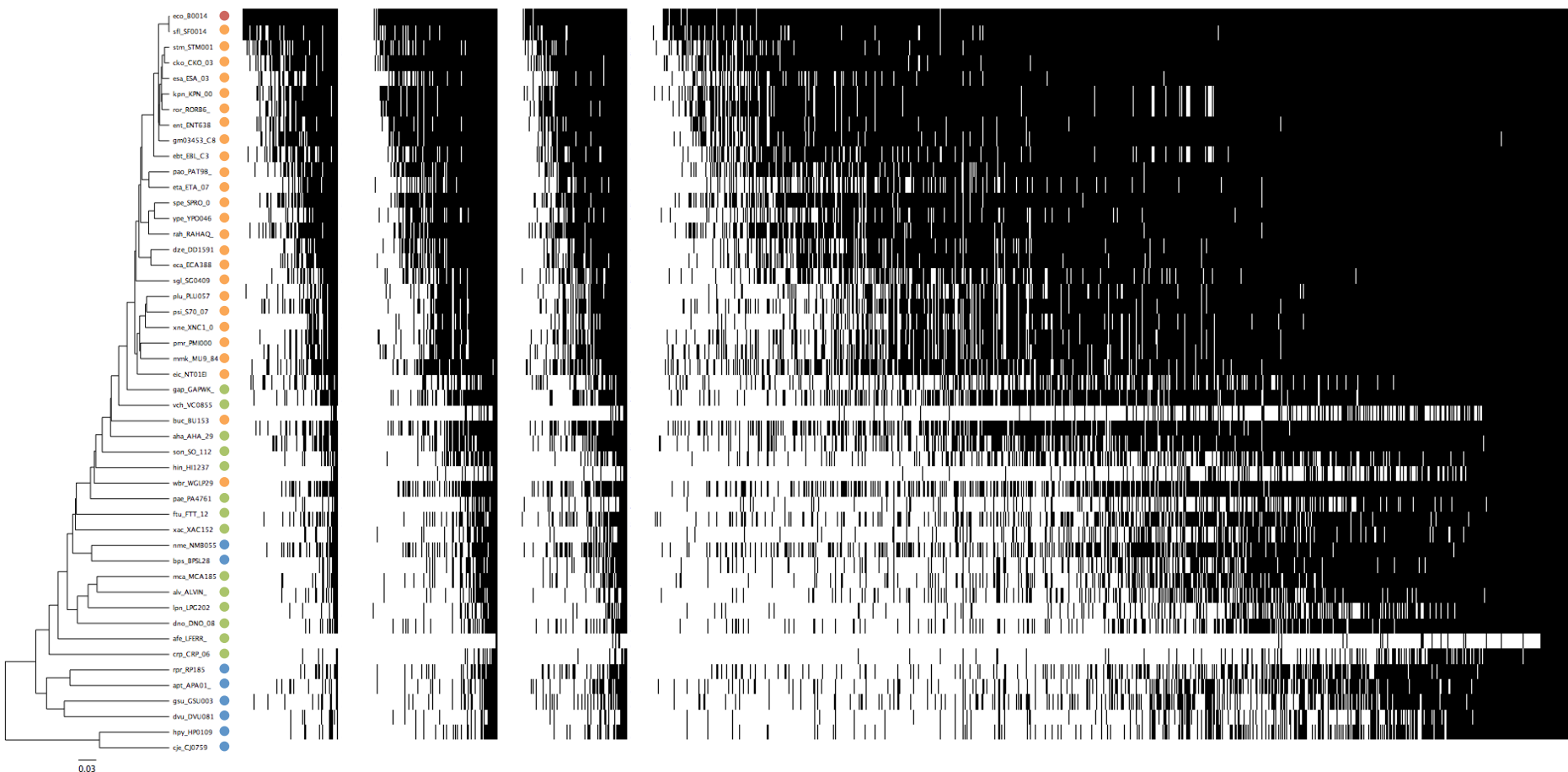

Supplement: S14 Fig — For each gene cluster (columns), boxes indicate the presence (black) or absence (white) of genes in the corresponding genomes (rows). Left panel shows the reference phylogenetic tree for proteobacteria species computed using DnaK protein sequences of these species. (PDF) [file pgen.1005796.s014.pdf]

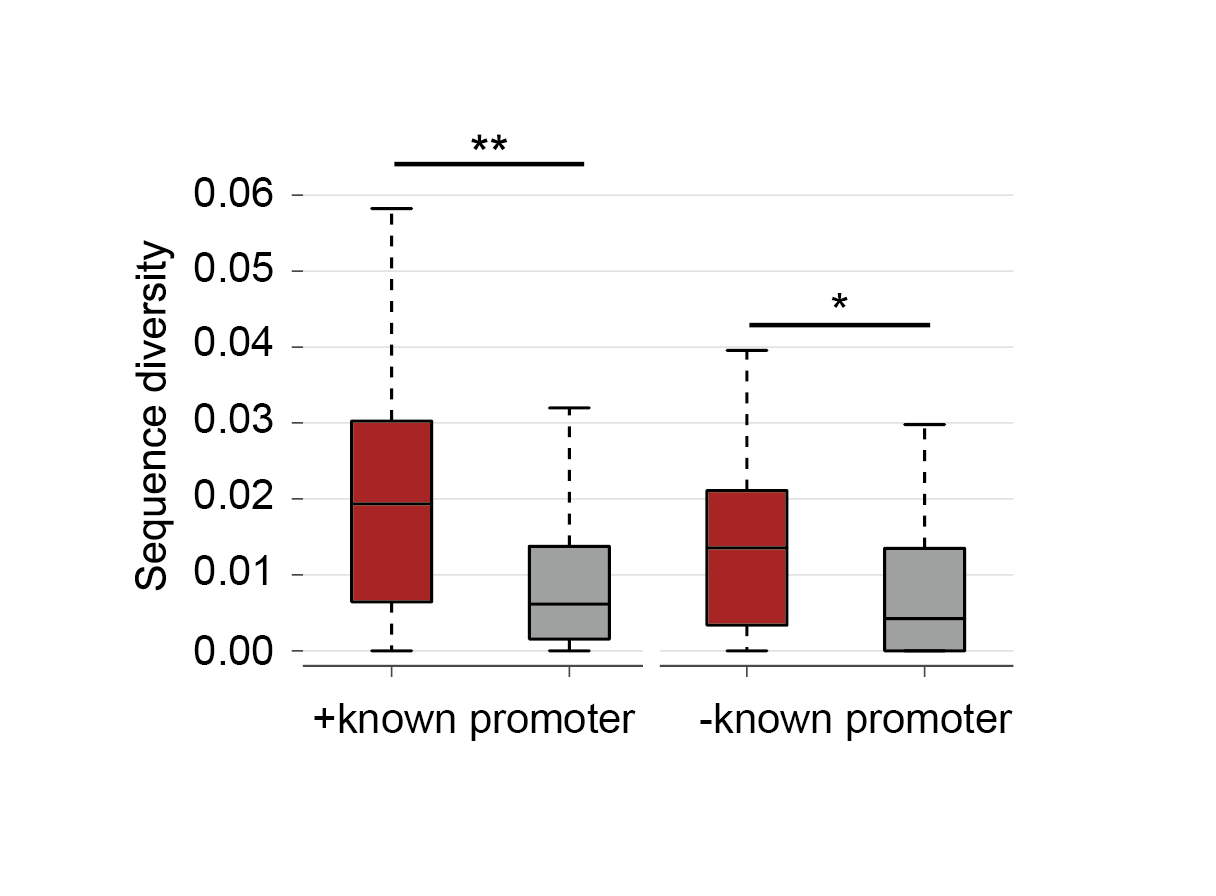

Supplement: S15 Fig — Each distribution of sequence diversity is indicated as for Fig 3. The information regarding promoters was acquired from the RegulonDB database [76]. Sequence diversity of H-NS-bound (+known promoter; N = 50, median value = 0.019) and -unbound (+known promoter; N = 267, median value = 0.0062) class II intergenic regions with known promoters (left) and of H-NS-bound (−known promoter; N = 30, median value = 0.014) and H-NS-unbound (−known promoter; N = 264, median value = 0.0043) class II intergenic regions without known promoters (right). The asterisks indicate the statistical significance of the difference between the sequence diversities in the H-NS-bound and -unbound genes and intergenic regions as assessed with the Wilcoxon rank-sum test (*p < 0.05, ns: not significant). (TIF) [file pgen.1005796.s015.tif]
