# Supplementary material for: H-NS Facilitates Sequence Diversification of Horizontally Transferred DNAs during Their Integration in Host Chromosomes
Source: PLoS Genet. 2016 Jan 20;12(1):e1005796. doi: 10.1371/journal.pgen.1005796 (PMC4720273; doi:10.1371/journal.pgen.1005796)
Supplement: S6 Fig — H-NS binding profiles in duplicate experiments are presented in CDS maps, which are the original H-NS binding profiles shown in Fig 1A, for SE11, SE15, and K-12. Overlapping binding regions in the two experiments are indicated with rectangles above the CDS maps. (PDF) [file pgen.1005796.s006.pdf]

H-NS binding profiles in *Escherichia coli* SE11

(1st experiment and 2nd experiment)

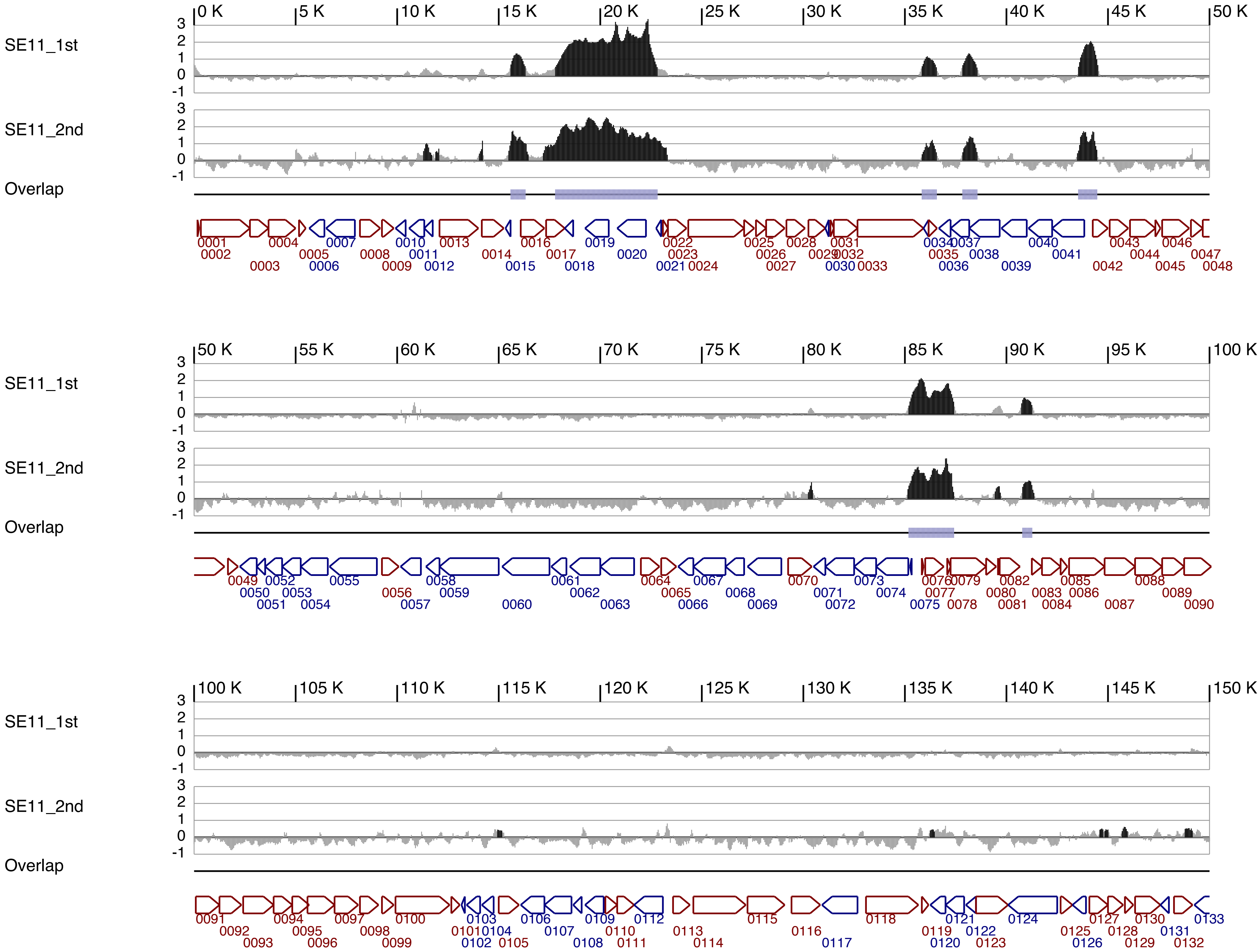

0 K
5 K
10 K
15 K
20 K
25 K
30 K
35 K
40 K
45 K
50 K
SE11_1st
SE11_2nd
Overlap
3
2
1
0
-1
0001 0002 0003 0004 0005 0006 0007 0008 0009 0010 0011 0012 0013 0014 0015 0016 0017 0018 0019 0020 0021 0022 0023 0024 0025 0026 0027 0028 0029 0030 0031 0032 0033 0034 0035 0036 0037 0038 0039 0040 0041 0042 0043 0044 0045 0046 0047 0048
50 K
55 K
60 K
65 K
70 K
75 K
80 K
85 K
90 K
95 K
100 K
SE11_1st
SE11_2nd
Overlap
0049 0050 0051 0052 0053 0054 0055 0056 0057 0058 0059 0060 0061 0062 0063 0064 0065 0066 0067 0068 0069 0070 0071 0072 0073 0074 0075 0076 0077 0078 0079 0080 0081 0082 0083 0084 0085 0086 0087 0088 0089 0090
100 K
105 K
110 K
115 K
120 K
125 K
130 K
135 K
140 K
145 K
150 K
SE11_1st
SE11_2nd
Overlap
0091 0092 0093 0094 0095 0096 0097 0098 0099 0100 0101 0102 0103 0104 0105 0106 0107 0108 0109 0110 0111 0112 0113 0114 0115 0116 0117 0118 0119 0120 0121 0122 0123 0124 0125 0126 0127 0128 0129 0130 0131 0132 0133

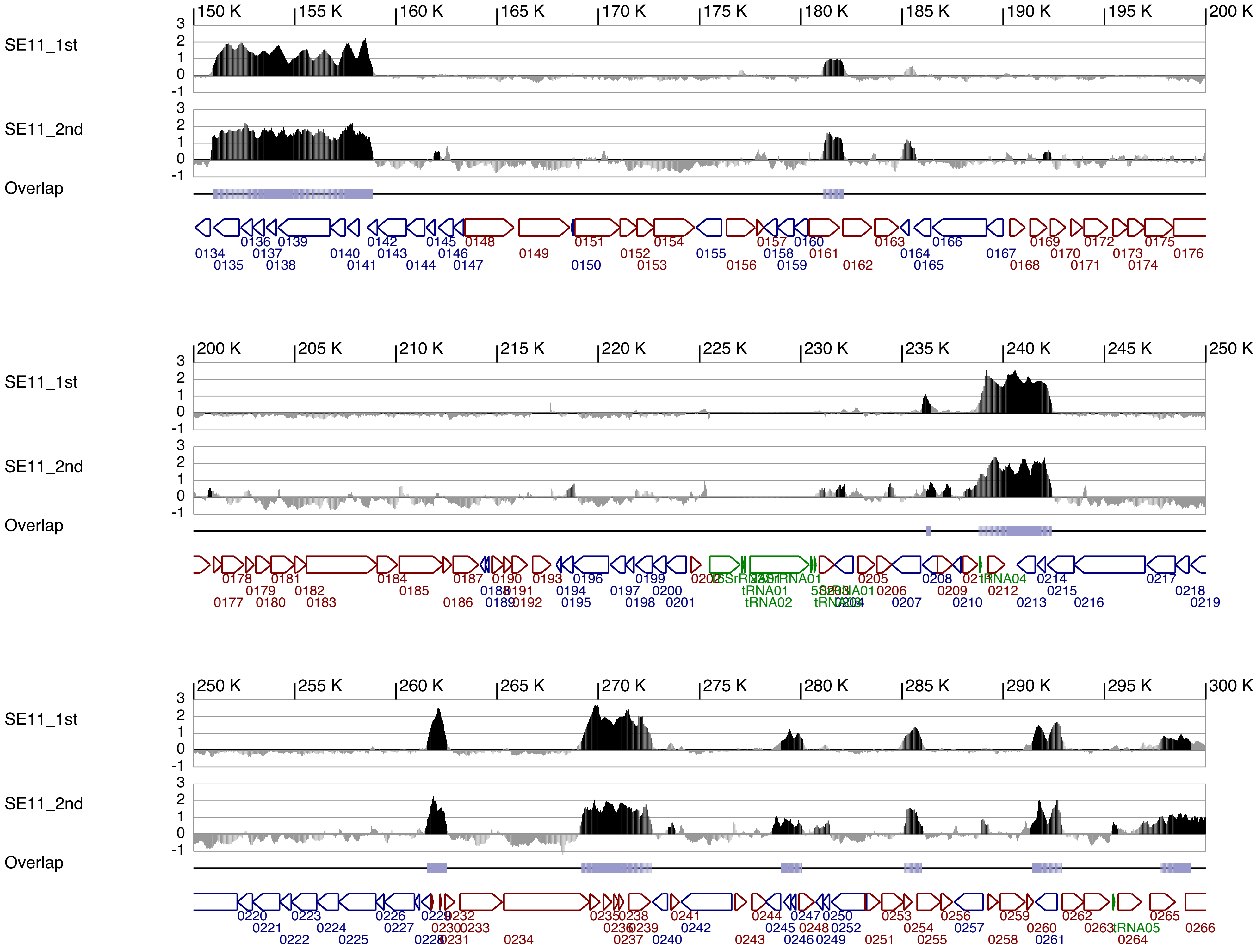

150 K
155 K
160 K
165 K
170 K
175 K
180 K
185 K
190 K
195 K
200 K
SE11_1st
SE11_2nd
Overlap
200 K
205 K
210 K
215 K
220 K
225 K
230 K
235 K
240 K
245 K
250 K
SE11_1st
SE11_2nd
Overlap
250 K
255 K
260 K
265 K
270 K
275 K
280 K
285 K
290 K
295 K
300 K
SE11_1st
SE11_2nd
Overlap

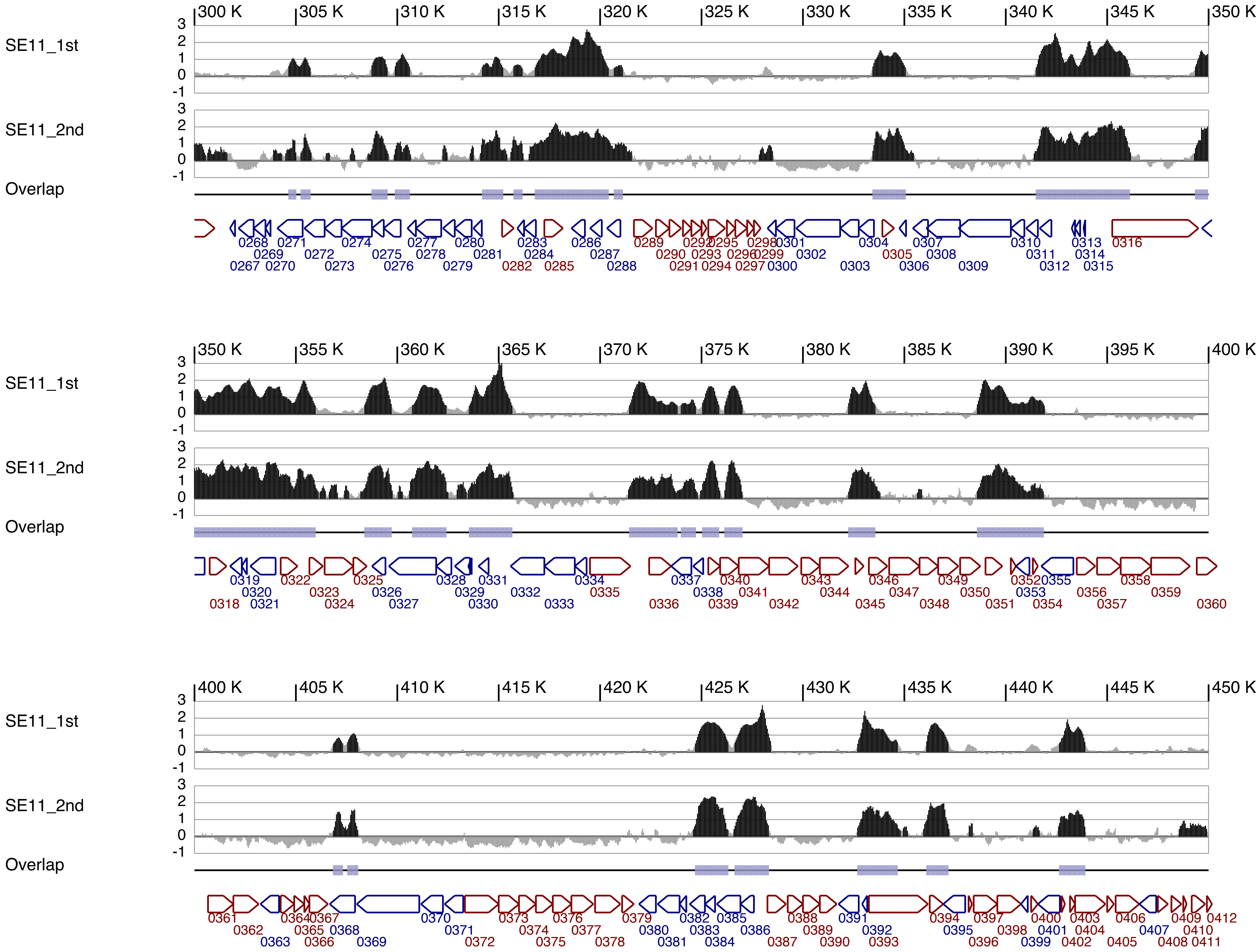
300 K 305 K 310 K 315 K 320 K 325 K 330 K 335 K 340 K 345 K 350 K
SE11_1st
SE11_2nd
Overlap
3 2 1 0 -1
0268 0271 0274 0277 0280 0283 0286 0289 0292 0295 0298 0301 0304 0307 0310 0313 0316
0269 0272 0275 0278 0281 0284 0287 0290 0293 0296 0299 0302 0305 0308 0311 0314
0267 0270 0273 0276 0279 0282 0285 0288 0291 0294 0297 0300 0303 0306 0309 0312 0315
350 K 355 K 360 K 365 K 370 K 375 K 380 K 385 K 390 K 395 K 400 K
SE11_1st
SE11_2nd
Overlap
0319 0322 0325 0328 0331 0334 0337 0340 0343 0346 0349 0352 0355 0358
0320 0323 0326 0329 0332 0335 0338 0341 0344 0347 0350 0353 0356 0359
0318 0321 0324 0327 0330 0333 0336 0339 0342 0345 0348 0351 0354 0357 0360
400 K 405 K 410 K 415 K 420 K 425 K 430 K 435 K 440 K 445 K 450 K
SE11_1st
SE11_2nd
Overlap
0361 0364 0367 0370 0373 0376 0379 0382 0385 0388 0391 0394 0397 0400 0403 0406 0409 0412
0362 0365 0368 0371 0374 0377 0380 0383 0386 0389 0392 0395 0398 0401 0404 0407 0410
0363 0366 0369 0372 0375 0378 0381 0384 0387 0390 0393 0396 0399 0402 0405 0408 0411

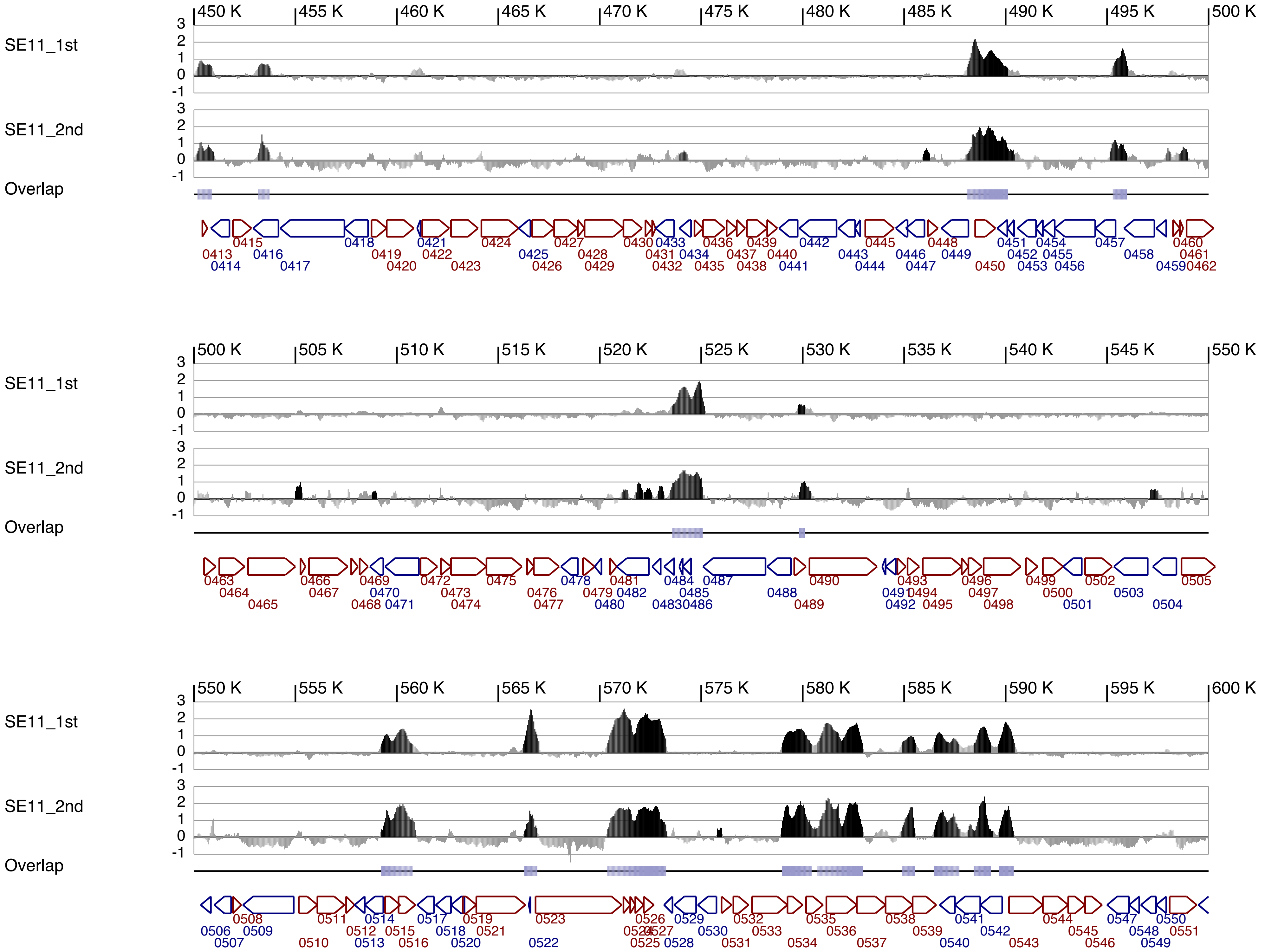
450 K
455 K
460 K
465 K
470 K
475 K
480 K
485 K
490 K
495 K
500 K
SE11_1st
SE11_2nd
Overlap
505 K
510 K
515 K
520 K
525 K
530 K
535 K
540 K
545 K
550 K
555 K
560 K
565 K
570 K
575 K
580 K
585 K
590 K
595 K
600 K

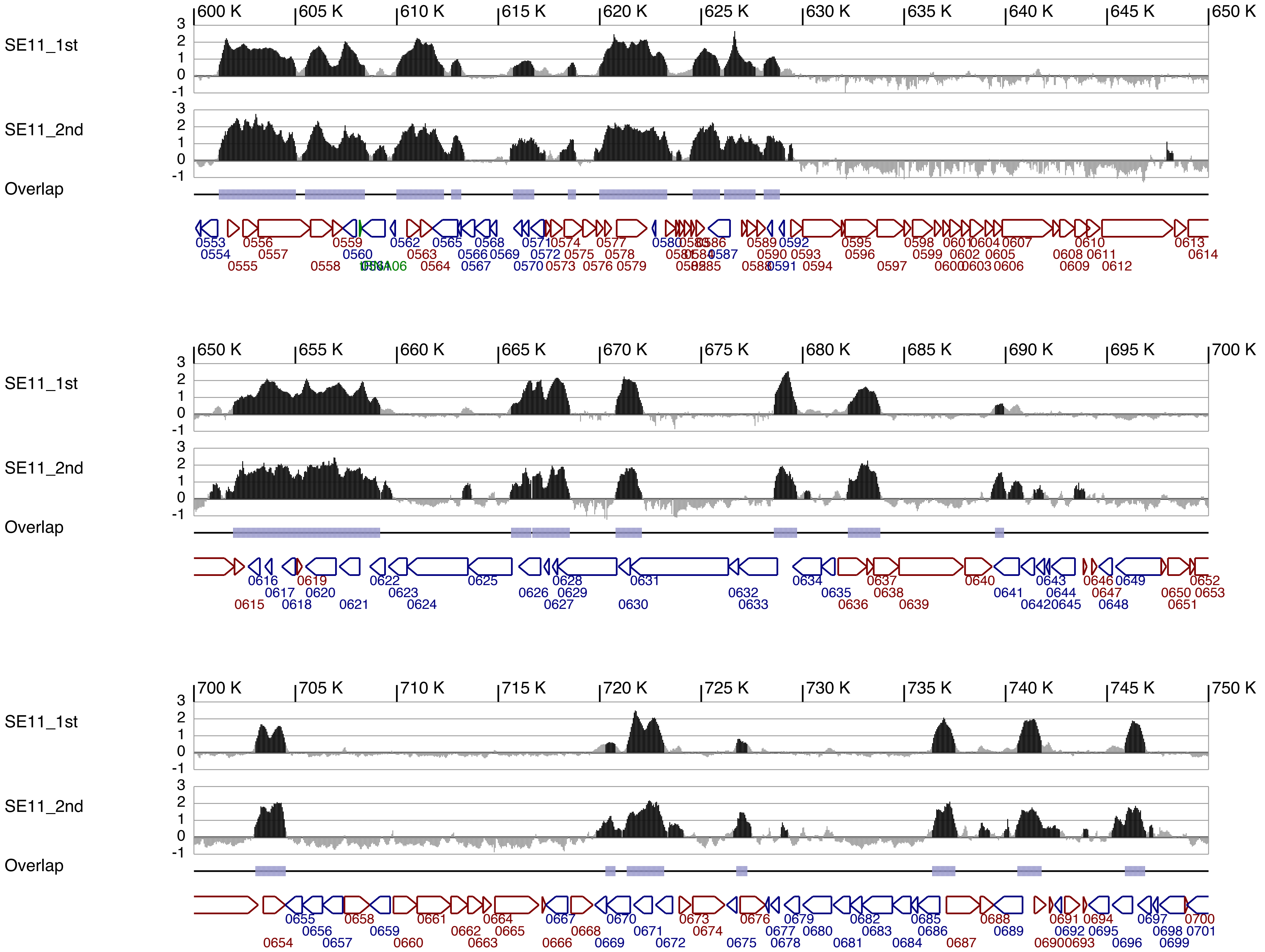

600 K
605 K
610 K
615 K
620 K
625 K
630 K
635 K
640 K
645 K
650 K
SE11_1st
SE11_2nd
Overlap
3
2
1
0
-1
0553 0554 0555 0556 0557 0558 0559 0560 0562 0563 0564 0565 0566 0567 0568 0569 0570 0572 0573 0574 0575 0576 0577 0578 0579 0580 0586 0587 0588 0589 0590 0591 0592 0593 0594 0595 0596 0597 0598 0599 0600 0602 0603 0604 0605 0606 0607 0608 0609 0610 0611 0612 0613 0614
655 K
660 K
665 K
670 K
675 K
680 K
685 K
690 K
695 K
700 K
0615 0616 0617 0618 0619 0620 0621 0622 0623 0624 0625 0626 0627 0628 0629 0630 0631 0632 0633 0634 0635 0636 0637 0638 0639 0640 0641 0643 0644 0646 0647 0648 0649 0650 0651 0652 0653
705 K
710 K
715 K
720 K
725 K
730 K
735 K
740 K
745 K
750 K
0654 0655 0656 0657 0658 0659 0660 0661 0662 0663 0664 0665 0666 0667 0668 0669 0670 0671 0672 0673 0674 0675 0676 0677 0678 0679 0680 0681 0682 0683 0684 0685 0686 0687 0688 0689 0691 0692 0694 0695 0696 0697 0698 0699 0700 0701

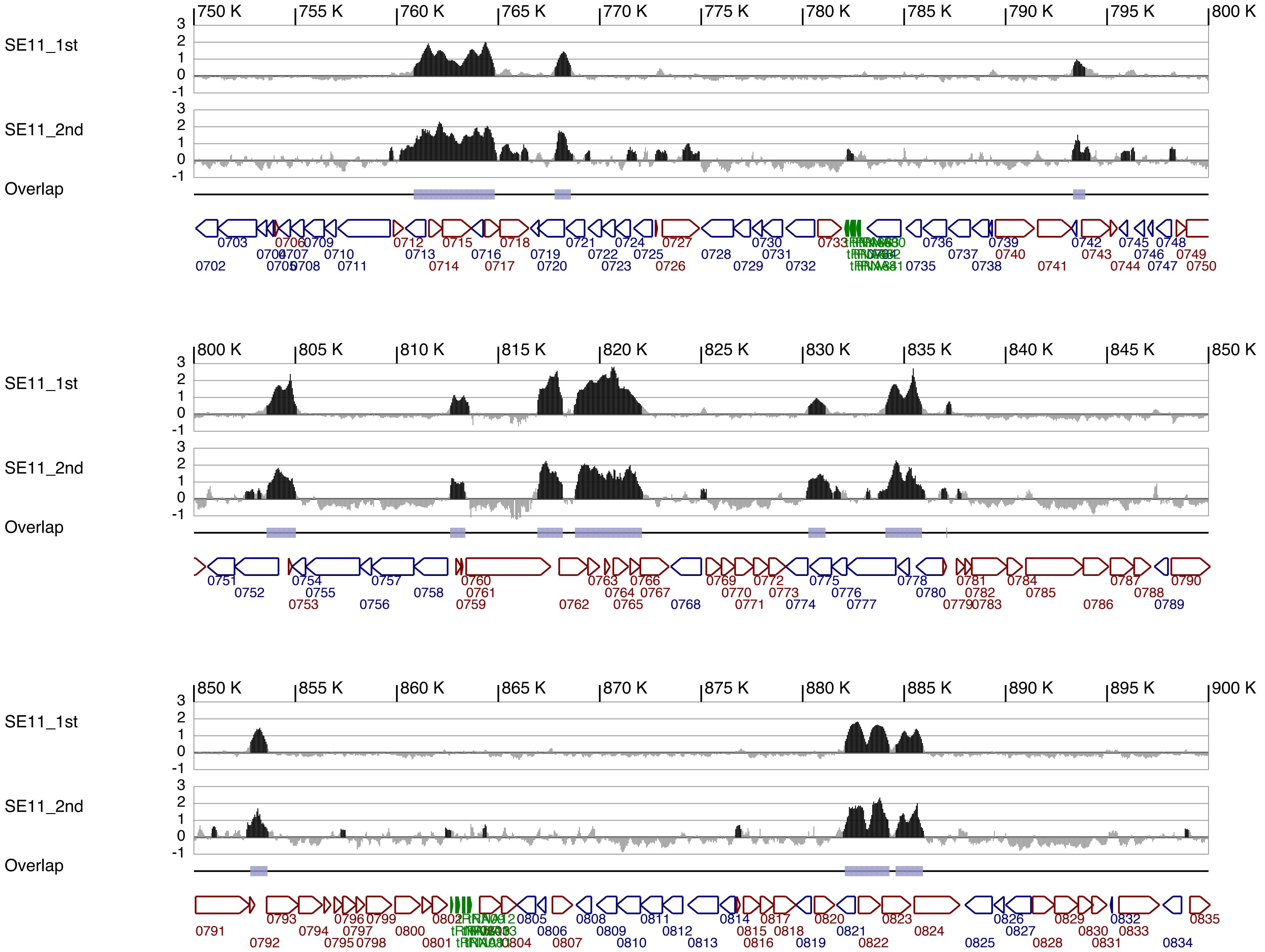
750 K
755 K
760 K
765 K
770 K
775 K
780 K
785 K
790 K
795 K
800 K
SE11_1st
SE11_2nd
Overlap
3
2
1
0
-1
0703 0706 0709 0712 0715 0718 0721 0724 0727 0730 0736 0739 0742 0745 0748
0704 0707 0710 0713 0716 0719 0722 0725 0728 0731 0737 0740 0743 0746 0749
0702 0705 0708 0711 0714 0717 0720 0723 0726 0729 0732 0735 0738 0741 0744 0747 0750
0733
805 K
810 K
815 K
820 K
825 K
830 K
835 K
840 K
845 K
850 K
0751 0754 0757 0760 0763 0766 0769 0772 0775 0778 0781 0784 0787 0790
0752 0755 0758 0761 0764 0767 0770 0773 0776 0780 0782 0785 0788
0753 0756 0759 0762 0765 0768 0771 0774 0777 0779 0783 0786 0789
855 K
860 K
865 K
870 K
875 K
880 K
885 K
890 K
895 K
900 K
0793 0796 0799 0802 0805 0808 0811 0814 0817 0820 0823 0826 0829 0832 0835
0791 0794 0797 0800 0806 0809 0812 0815 0818 0821 0824 0827 0830 0833
0792 0795 0798 0801 0804 0807 0810 0813 0816 0819 0822 0825 0828 0831 0834

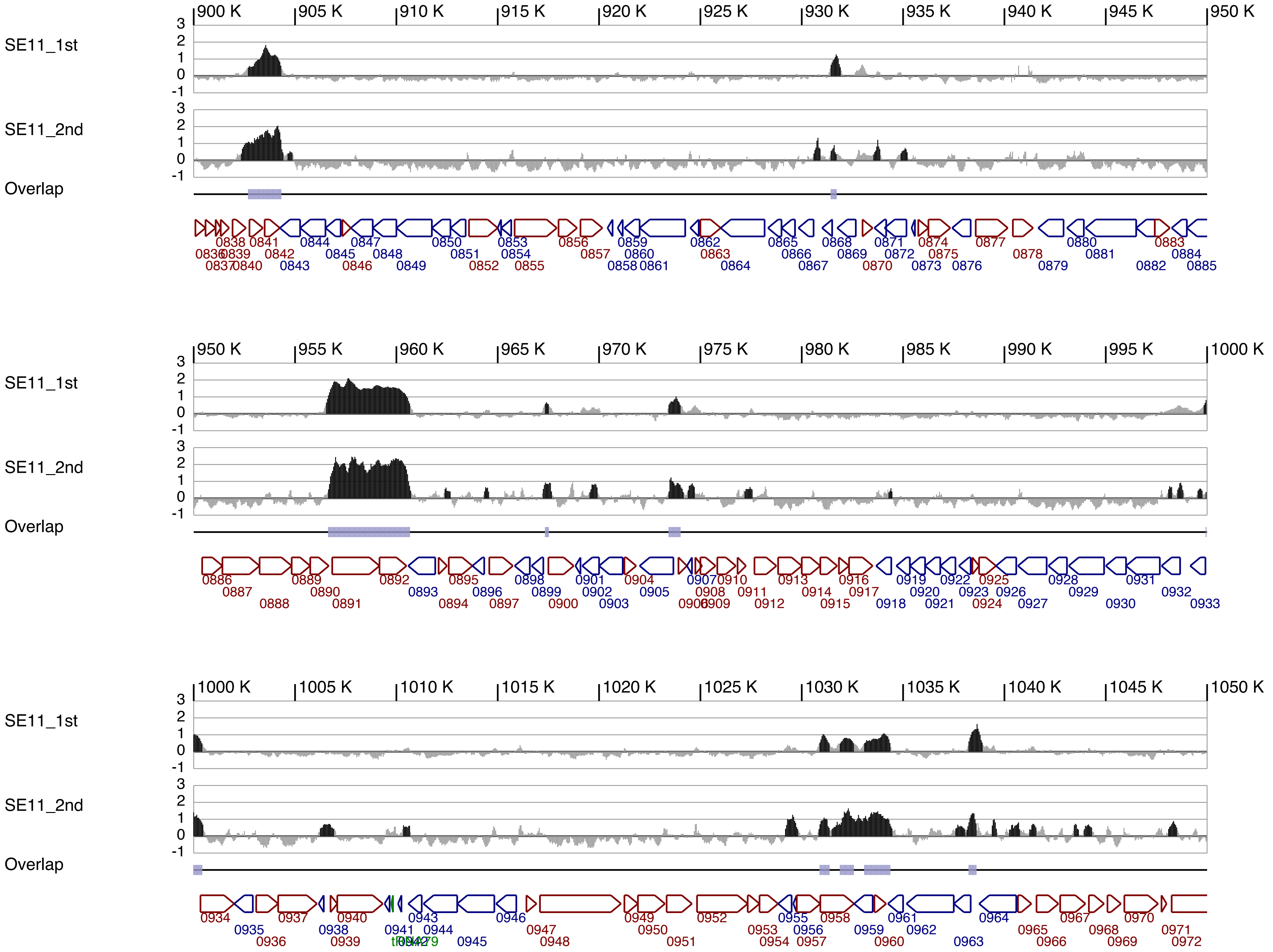

900 K
905 K
910 K
915 K
920 K
925 K
930 K
935 K
940 K
945 K
950 K
SE11_1st
SE11_2nd
Overlap
0838 0841 0844 0847 0850 0853 0856 0859 0862 0865 0868 0871 0874 0877 0880 0883
0839 0842 0845 0848 0851 0854 0857 0860 0863 0866 0869 0872 0875 0878 0881 0884
0843 0846 0849 0852 0855 0858 0861 0864 0867 0870 0873 0876 0879 0882 0885
950 K
955 K
960 K
965 K
970 K
975 K
980 K
985 K
990 K
995 K
1000 K
SE11_1st
SE11_2nd
Overlap
0886 0889 0892 0895 0898 0901 0904 0913 0916 0919 0922 0925 0928 0931
0887 0890 0893 0896 0899 0902 0905 0908 0911 0914 0917 0920 0923 0926 0929 0932
0888 0891 0894 0897 0900 0903 0909 0912 0915 0918 0921 0924 0927 0930 0933
1000 K
1005 K
1010 K
1015 K
1020 K
1025 K
1030 K
1035 K
1040 K
1045 K
1050 K
SE11_1st
SE11_2nd
Overlap
0934 0937 0940 0943 0946 0949 0952 0955 0958 0961 0964 0967 0970
0935 0938 0941 0944 0947 0950 0953 0956 0959 0962 0965 0968 0971
0936 0939 0945 0948 0951 0954 0957 0960 0963 0966 0969 0972

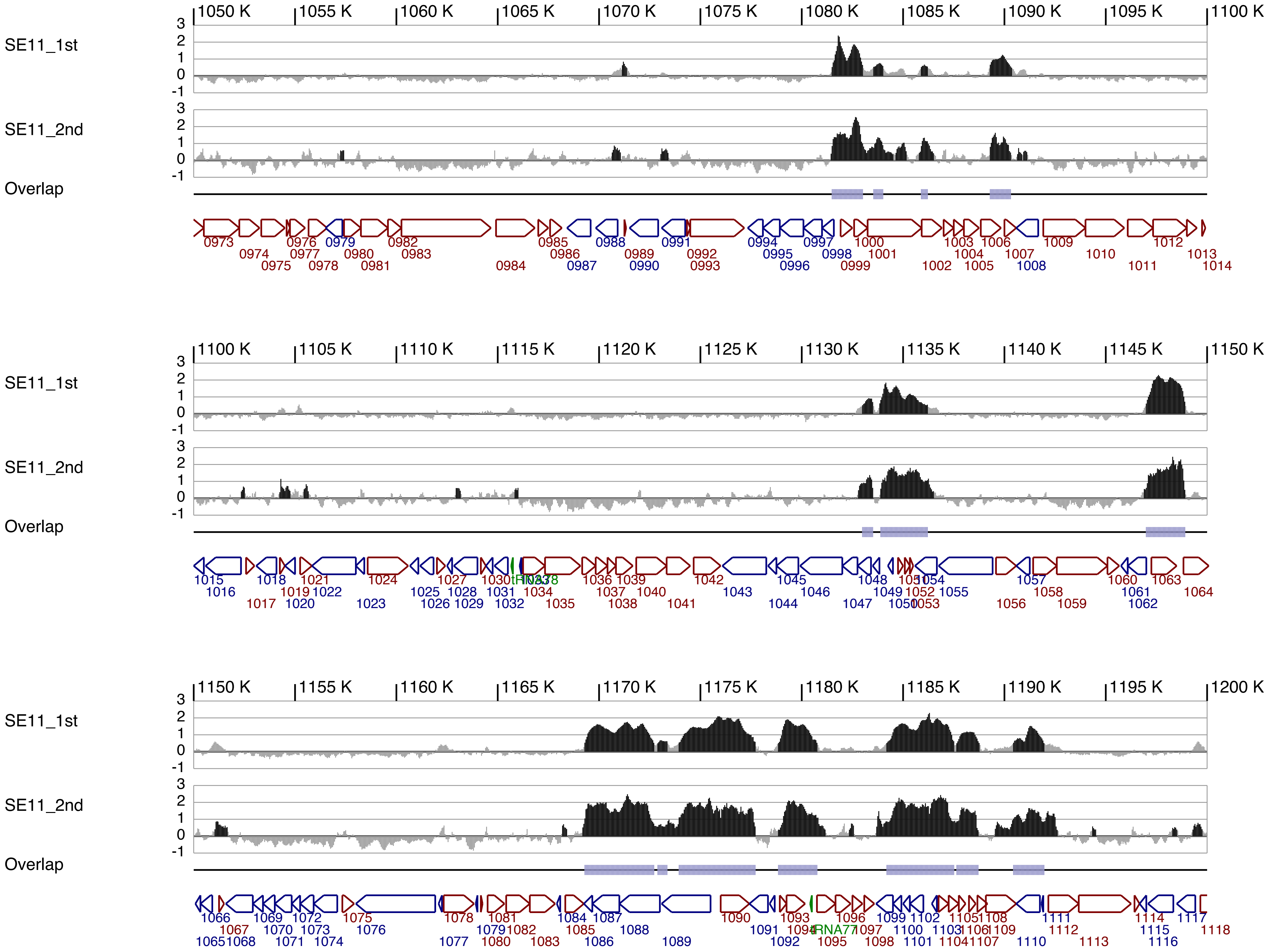

1050 K
1055 K
1060 K
1065 K
1070 K
1075 K
1080 K
1085 K
1090 K
1095 K
1100 K
SE11_1st
SE11_2nd
Overlap
3
2
1
0
-1
0973 0974 0975 0976 0977 0978 0979 0980 0981 0982 0983 0984 0985 0986 0987 0988 0989 0990 0991 0992 0993 0994 0995 0996 0997 0998 0999 1000 1001 1002 1003 1004 1005 1006 1007 1008 1009 1010 1011 1012 1013 1014
1100 K
1105 K
1110 K
1115 K
1120 K
1125 K
1130 K
1135 K
1140 K
1145 K
1150 K
SE11_1st
SE11_2nd
Overlap
1015 1016 1017 1018 1019 1020 1021 1022 1023 1024 1025 1026 1027 1028 1029 1030 1031 1032 1034 1035 1036 1037 1038 1039 1040 1041 1042 1043 1044 1045 1046 1047 1048 1049 1052 1054 1055 1056 1057 1058 1059 1060 1061 1062 1063 1064
1150 K
1155 K
1160 K
1165 K
1170 K
1175 K
1180 K
1185 K
1190 K
1195 K
1200 K
SE11_1st
SE11_2nd
Overlap
1066 1067 1069 1070 1071 1072 1073 1074 1075 1076 1077 1078 1080 1081 1082 1083 1084 1085 1086 1087 1088 1089 1090 1091 1092 1093 1095 1096 1097 1098 1099 1100 1101 1102 1103 1109 1110 1111 1112 1113 1114 1115 1116 1117 1118

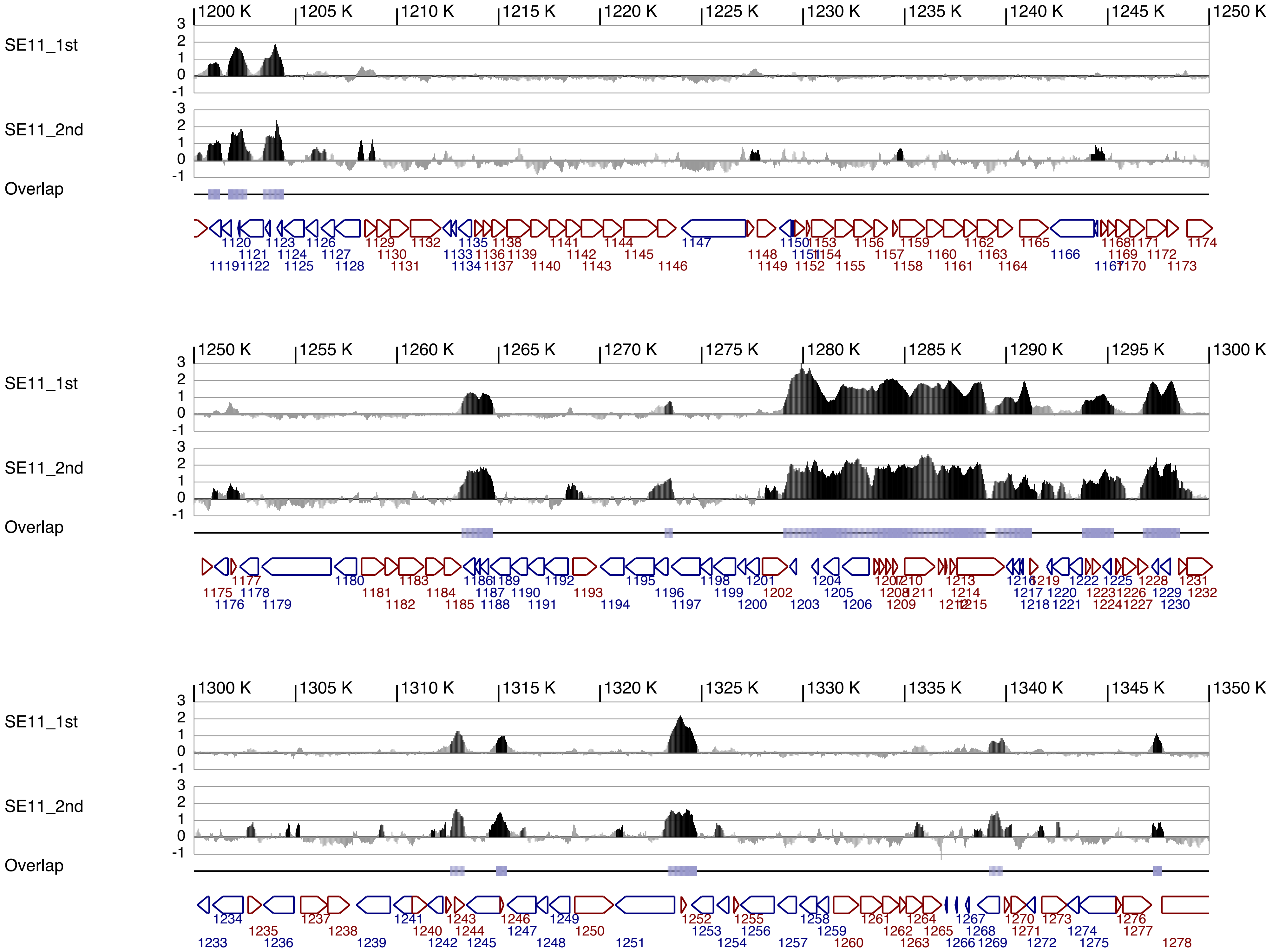

1200 K
1205 K
1210 K
1215 K
1220 K
1225 K
1230 K
1235 K
1240 K
1245 K
1250 K
SE11_1st
SE11_2nd
Overlap
3
2
1
0
-1
1120 1123 1126 1129 1132 1135 1138 1141 1144 1147 1150 1153 1156 1159 1162 1165 1168 1171 1174
1121 1124 1127 1130 1133 1136 1139 1142 1145 1148 1151 1154 1157 1160 1163 1166 1169 1172
1119 1122 1125 1128 1131 1134 1137 1140 1143 1146 1149 1152 1155 1158 1161 1164 1167 1170 1173
1255 K
1260 K
1265 K
1270 K
1275 K
1280 K
1285 K
1290 K
1295 K
1300 K
1177 1180 1183 1186 1189 1192 1195 1198 1201 1204 1207 1210 1213 1216 1219 1222 1225 1228 1231
1175 1178 1181 1184 1187 1190 1193 1196 1199 1202 1205 1208 1211 1214 1217 1220 1223 1226 1229 1232
1176 1179 1182 1185 1188 1191 1194 1197 1200 1203 1206 1209 1212 1215 1218 1221 1224 1227 1230
1305 K
1310 K
1315 K
1320 K
1325 K
1330 K
1335 K
1340 K
1345 K
1350 K
1234 1237 1241 1243 1246 1249 1252 1255 1258 1261 1264 1267 1270 1273 1276
1235 1238 1240 1244 1247 1250 1253 1256 1259 1262 1265 1268 1271 1274 1277
1233 1236 1239 1242 1245 1248 1251 1254 1257 1260 1263 1266 1269 1272 1275 1278

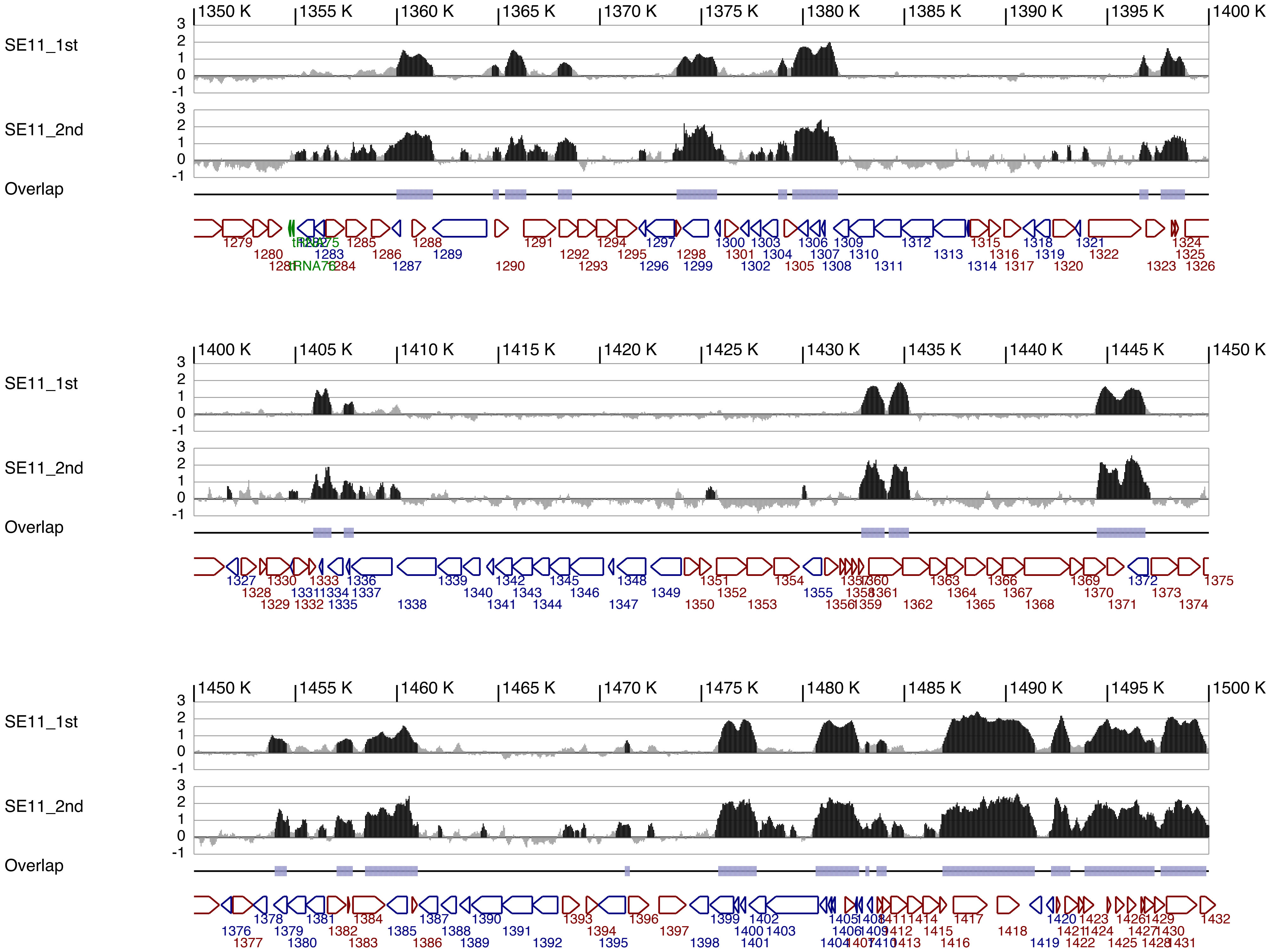
1350 K 1355 K 1360 K 1365 K 1370 K 1375 K 1380 K 1385 K 1390 K 1395 K 1400 K
SE11_1st
SE11_2nd
Overlap
1400 K 1405 K 1410 K 1415 K 1420 K 1425 K 1430 K 1435 K 1440 K 1445 K 1450 K
SE11_1st
SE11_2nd
Overlap
1450 K 1455 K 1460 K 1465 K 1470 K 1475 K 1480 K 1485 K 1490 K 1495 K 1500 K
SE11_1st
SE11_2nd
Overlap

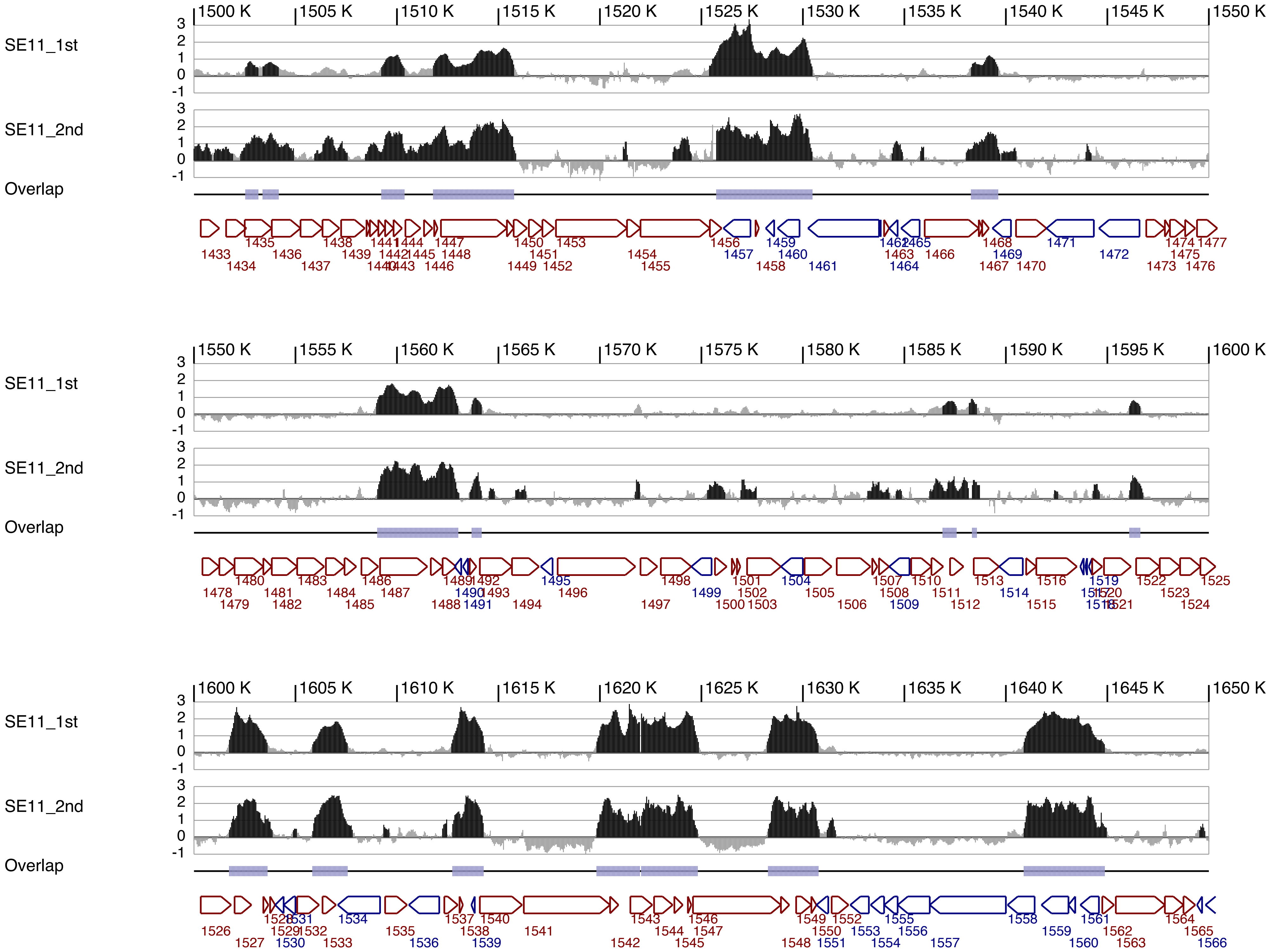

1500 K 1505 K 1510 K 1515 K 1520 K 1525 K 1530 K 1535 K 1540 K 1545 K 1550 K
SE11_1st
SE11_2nd
Overlap
3 2 1 0 -1
1435 1438 1441 1444 1447 1450 1453 1456 1459 1465 1468 1471 1474 1477
1433 1436 1439 1442 1445 1448 1451 1454 1457 1460 1463 1466 1469 1472 1475
1434 1437 1443 1446 1449 1452 1455 1458 1461 1464 1467 1470 1473 1476
1550 K 1555 K 1560 K 1565 K 1570 K 1575 K 1580 K 1585 K 1590 K 1595 K 1600 K
SE11_1st
SE11_2nd
Overlap
1480 1483 1486 1492 1495 1498 1501 1504 1507 1510 1513 1516 1519 1522 1525
1478 1481 1484 1487 1490 1493 1496 1499 1502 1505 1508 1511 1514 1520 1523
1479 1482 1485 1488 1491 1494 1497 1500 1503 1506 1509 1512 1515 1521 1524
1600 K 1605 K 1610 K 1615 K 1620 K 1625 K 1630 K 1635 K 1640 K 1645 K 1650 K
SE11_1st
SE11_2nd
Overlap
1531 1534 1537 1540 1543 1546 1549 1552 1555 1558 1561 1564
1526 1532 1535 1538 1541 1544 1547 1550 1553 1556 1559 1562 1565
1527 1530 1533 1536 1539 1542 1545 1548 1551 1554 1557 1560 1563 1566

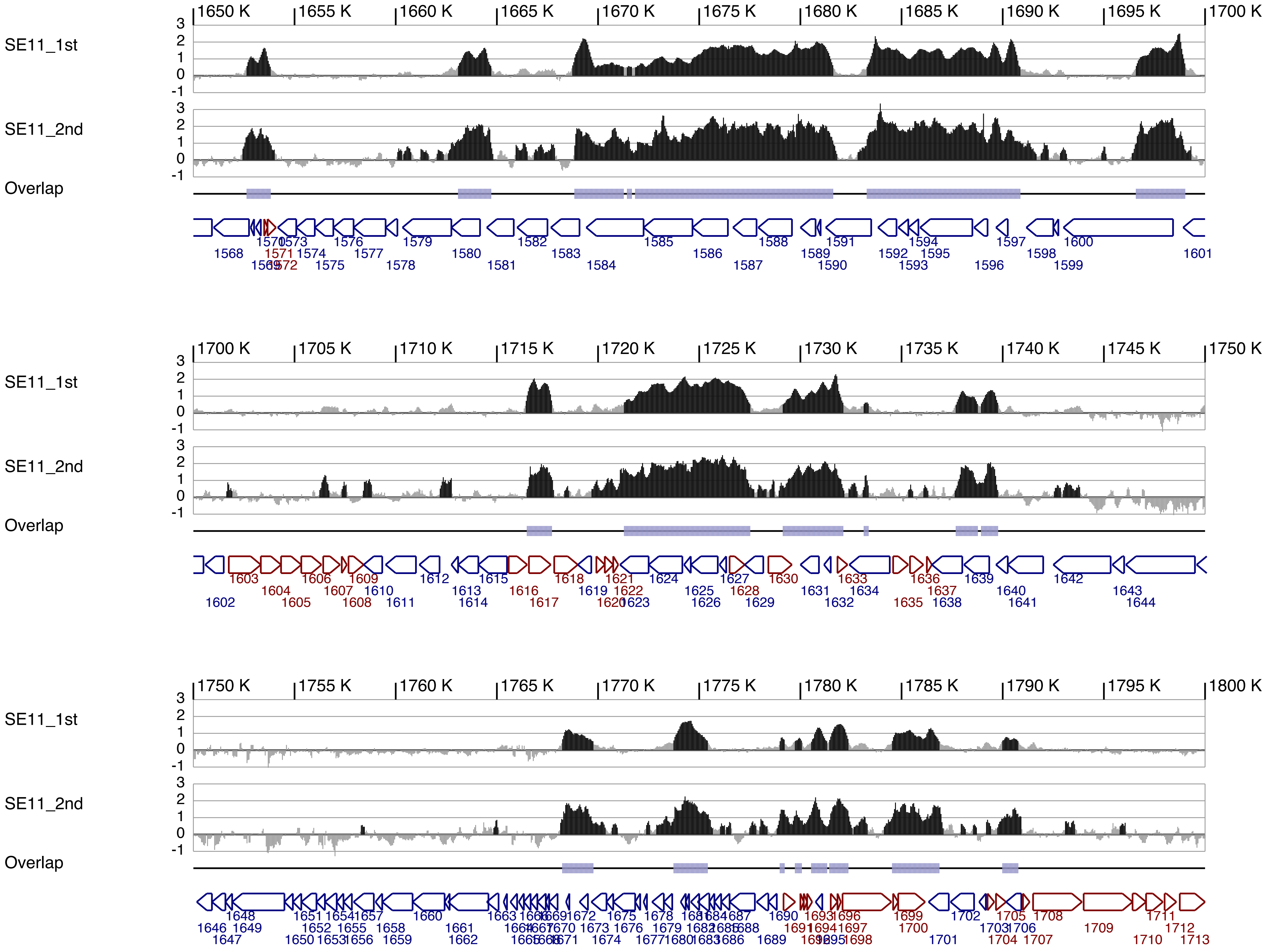

SE11_1st
SE11_2nd
Overlap
1650 K
1655 K
1660 K
1665 K
1670 K
1675 K
1680 K
1685 K
1690 K
1695 K
1700 K
1568 1574 1575 1576 1577 1578 1579 1580 1581 1582 1583 1584 1585 1586 1587 1588 1589 1590 1591 1592 1593 1594 1595 1596 1597 1598 1599 1600 1601
1705 K
1710 K
1715 K
1720 K
1725 K
1730 K
1735 K
1740 K
1745 K
1750 K
1602 1603 1604 1605 1606 1607 1608 1609 1610 1611 1612 1613 1614 1615 1616 1617 1618 1619 1621 1622 1624 1625 1626 1627 1628 1629 1630 1631 1632 1633 1634 1635 1636 1637 1638 1639 1640 1641 1642 1643 1644
1755 K
1760 K
1765 K
1770 K
1775 K
1780 K
1785 K
1790 K
1795 K
1800 K
1646 1647 1648 1649 1650 1651 1652 1655 1658 1659 1660 1661 1662 1672 1673 1674 1675 1676 1678 1679 1688 1689 1690 1696 1697 1698 1699 1700 1701 1702 1704 1705 1706 1707 1708 1709 1710 1711 1712 1713

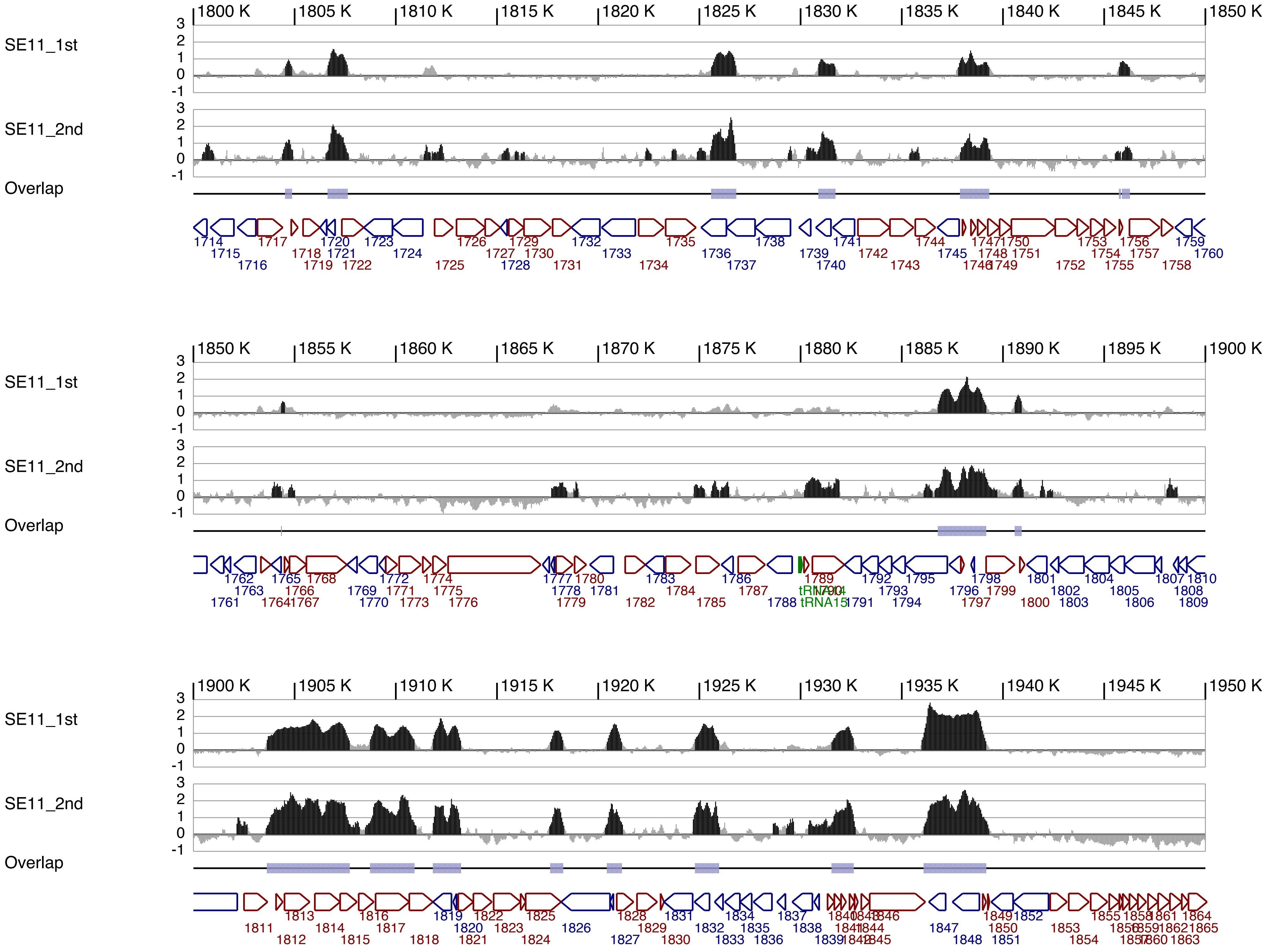
1800 K 1805 K 1810 K 1815 K 1820 K 1825 K 1830 K 1835 K 1840 K 1845 K 1850 K
SE11_1st
SE11_2nd
Overlap
3 2 1 0 -1
1714 1715 1716 1717 1718 1719 1720 1721 1722 1723 1724 1725 1726 1727 1728 1729 1730 1731 1732 1733 1734 1735 1736 1737 1738 1739 1740 1741 1742 1743 1744 1745 1748 1749 1750 1751 1752 1753 1754 1755 1756 1757 1758 1759 1760
1850 K 1855 K 1860 K 1865 K 1870 K 1875 K 1880 K 1885 K 1890 K 1895 K 1900 K
SE11_1st
SE11_2nd
Overlap
1761 1762 1763 1765 1766 1767 1768 1769 1770 1771 1772 1773 1774 1775 1776 1777 1778 1779 1780 1781 1782 1783 1784 1785 1786 1787 1788 1789 tRNA15 1791 1792 1793 1794 1795 1796 1797 1798 1799 1800 1801 1802 1803 1804 1805 1806 1808 1809 1810
1900 K 1905 K 1910 K 1915 K 1920 K 1925 K 1930 K 1935 K 1940 K 1945 K 1950 K
SE11_1st
SE11_2nd
Overlap
1811 1812 1813 1814 1815 1816 1817 1818 1819 1820 1821 1822 1823 1824 1825 1826 1827 1828 1829 1830 1831 1832 1833 1834 1835 1836 1837 1838 1844 1845 1846 1847 1848 1850 1851 1852 1853 1854 1855 1861 1862 1863 1864 1865

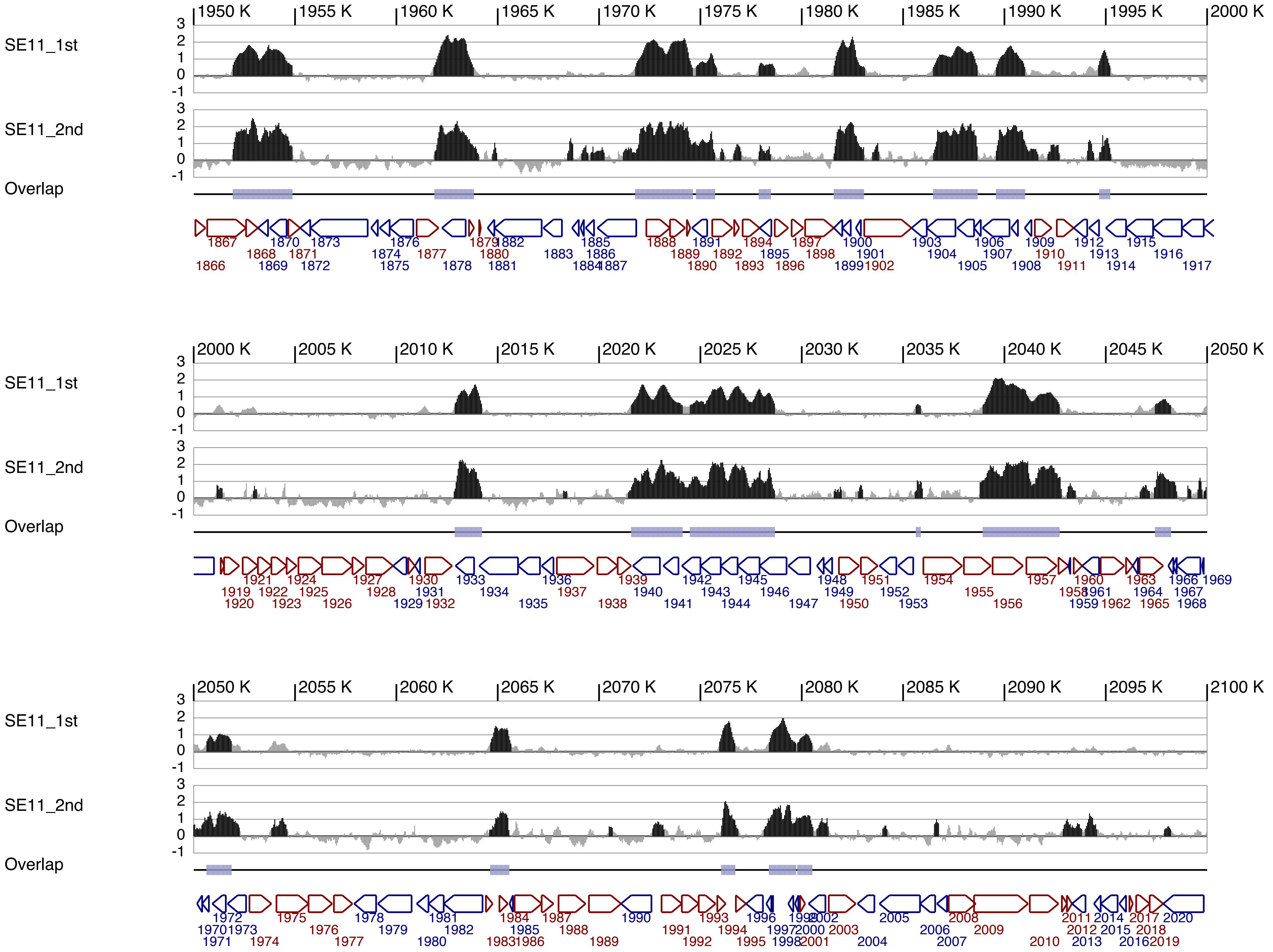

1950 K 1955 K 1960 K 1965 K 1970 K 1975 K 1980 K 1985 K 1990 K 1995 K 2000 K
SE11_1st
SE11_2nd
Overlap
3 2 1 0 -1
1867 1870 1873 1876 1879 1882 1885 1888 1891 1894 1897 1900 1903 1906 1909 1912 1915
1868 1871 1874 1877 1880 1883 1886 1889 1892 1895 1898 1901 1904 1907 1910 1913 1916
1866 1869 1872 1875 1878 1881 1884 1887 1890 1893 1896 1899 1902 1905 1908 1911 1914 1917
2000 K 2005 K 2010 K 2015 K 2020 K 2025 K 2030 K 2035 K 2040 K 2045 K 2050 K
SE11_1st
SE11_2nd
Overlap
3 2 1 0 -1
1921 1924 1927 1930 1933 1936 1939 1942 1945 1948 1951 1954 1957 1960 1963 1966 1969
1919 1922 1925 1928 1931 1934 1937 1940 1943 1946 1949 1952 1955 1958 1961 1964 1967
1920 1923 1926 1929 1932 1935 1938 1941 1944 1947 1950 1953 1956 1959 1962 1965 1968
2050 K 2055 K 2060 K 2065 K 2070 K 2075 K 2080 K 2085 K 2090 K 2095 K 2100 K
SE11_1st
SE11_2nd
Overlap
3 2 1 0 -1
1972 1975 1978 1981 1984 1987 1990 1993 1996 1999 2002 2005 2008 2011 2014 2017 2020
1970 1973 1976 1979 1982 1985 1988 1991 1994 1997 2000 2003 2006 2009 2012 2015 2018
1971 1974 1977 1980 1983 1986 1989 1992 1995 1998 2001 2004 2007 2010 2013 2016 2019

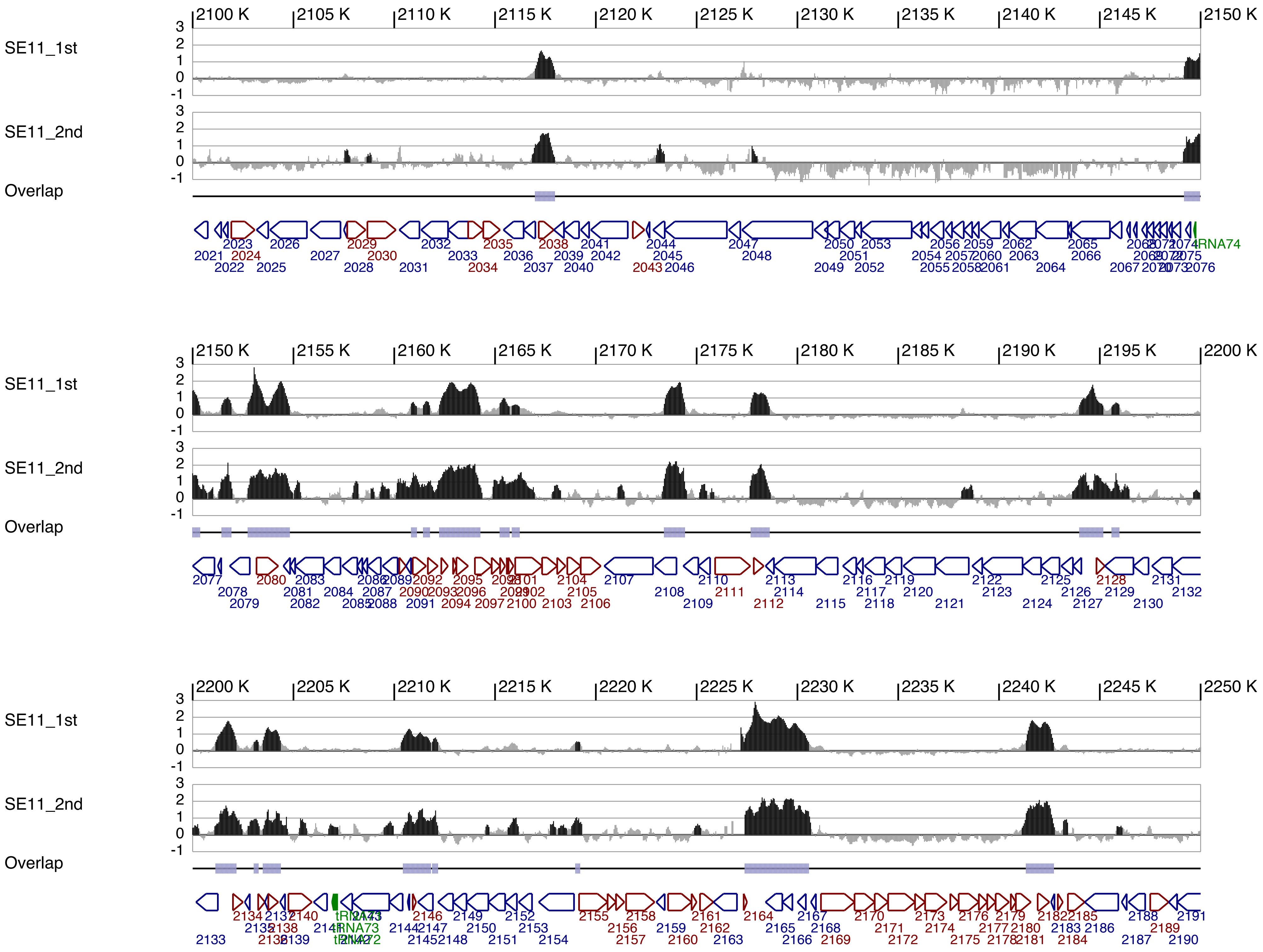
2100 K
2105 K
2110 K
2115 K
2120 K
2125 K
2130 K
2135 K
2140 K
2145 K
2150 K
SE11_1st
SE11_2nd
Overlap
3
2
1
0
-1
2023 2026 2029 2032 2035 2038 2041 2044 2047 2050 2053 2056 2059 2062 2065 tRNA74
2021 2024 2027 2030 2033 2036 2039 2042 2045 2048 2051 2054 2057 2060 2063 2066 2075
2022 2025 2028 2031 2034 2037 2040 2043 2046 2049 2052 2055 2058 2061 2064 2067 2076
2150 K
2155 K
2160 K
2165 K
2170 K
2175 K
2180 K
2185 K
2190 K
2195 K
2200 K
SE11_1st
SE11_2nd
Overlap
2077 2080 2083 2089 2092 2095 2101 2104 2107 2110 2113 2116 2119 2122 2125 2128 2131
2078 2081 2084 2087 2090 2093 2096 2105 2108 2111 2114 2117 2120 2123 2126 2129 2132
2079 2082 2088 2091 2094 2097 2100 2103 2106 2109 2112 2115 2118 2121 2124 2127 2130
2200 K
2205 K
2210 K
2215 K
2220 K
2225 K
2230 K
2235 K
2240 K
2245 K
2250 K
SE11_1st
SE11_2nd
Overlap
2134 2140 2146 2149 2152 2155 2158 2161 2164 2167 2170 2173 2176 2179 2185 2188 2191
2138 2144 2147 2150 2153 2156 2159 2162 2165 2168 2171 2174 2177 2180 2183 2186 2189
2133 2139 2148 2151 2154 2157 2160 2163 2166 2169 2172 2175 2178 2181 2184 2187 2190

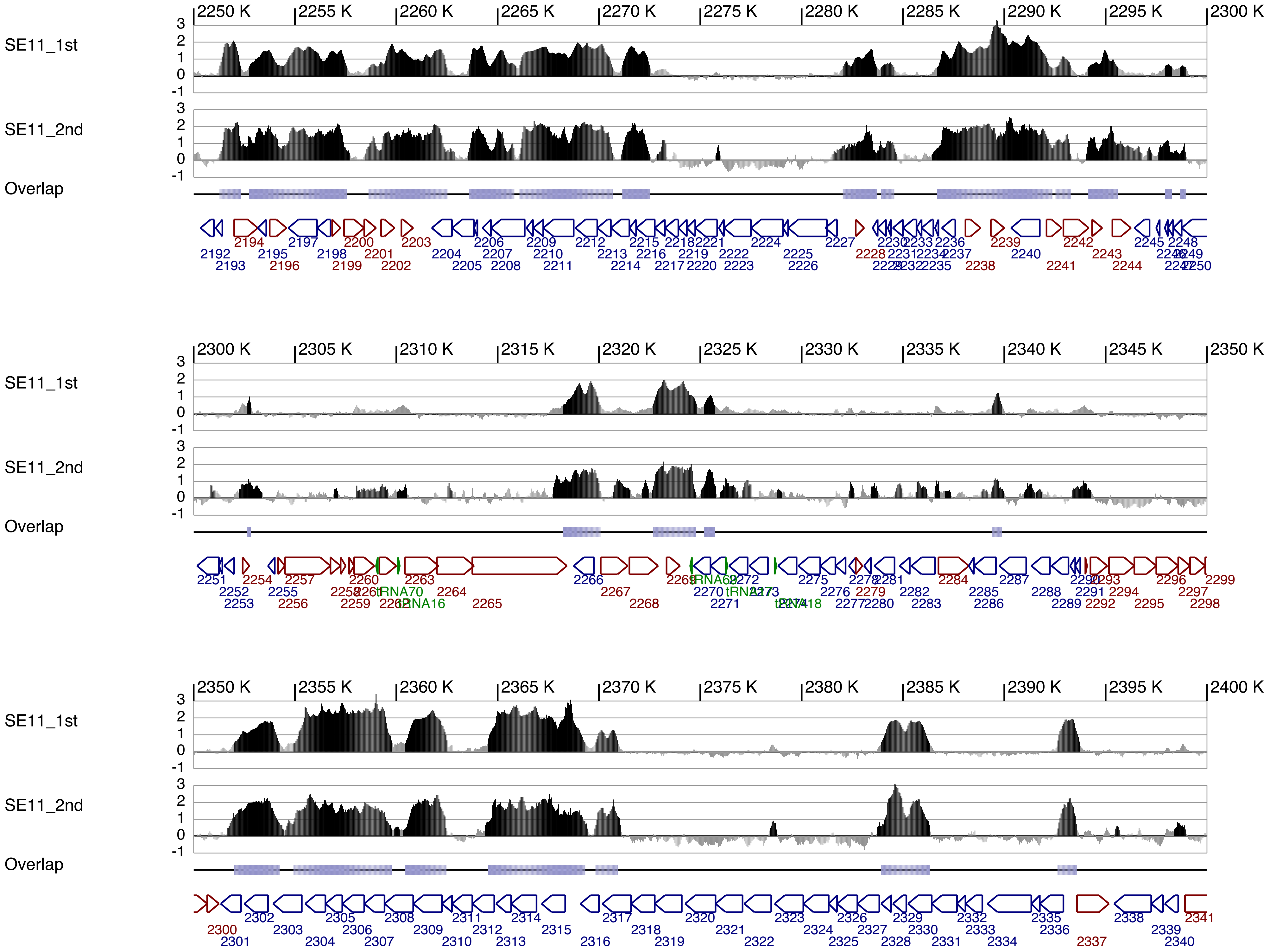

2250 K
2255 K
2260 K
2265 K
2270 K
2275 K
2280 K
2285 K
2290 K
2295 K
2300 K
SE11_1st
SE11_2nd
Overlap
2305 K
2310 K
2315 K
2320 K
2325 K
2330 K
2335 K
2340 K
2345 K
2350 K
2355 K
2360 K
2365 K
2370 K
2375 K
2380 K
2385 K
2390 K
2395 K
2400 K

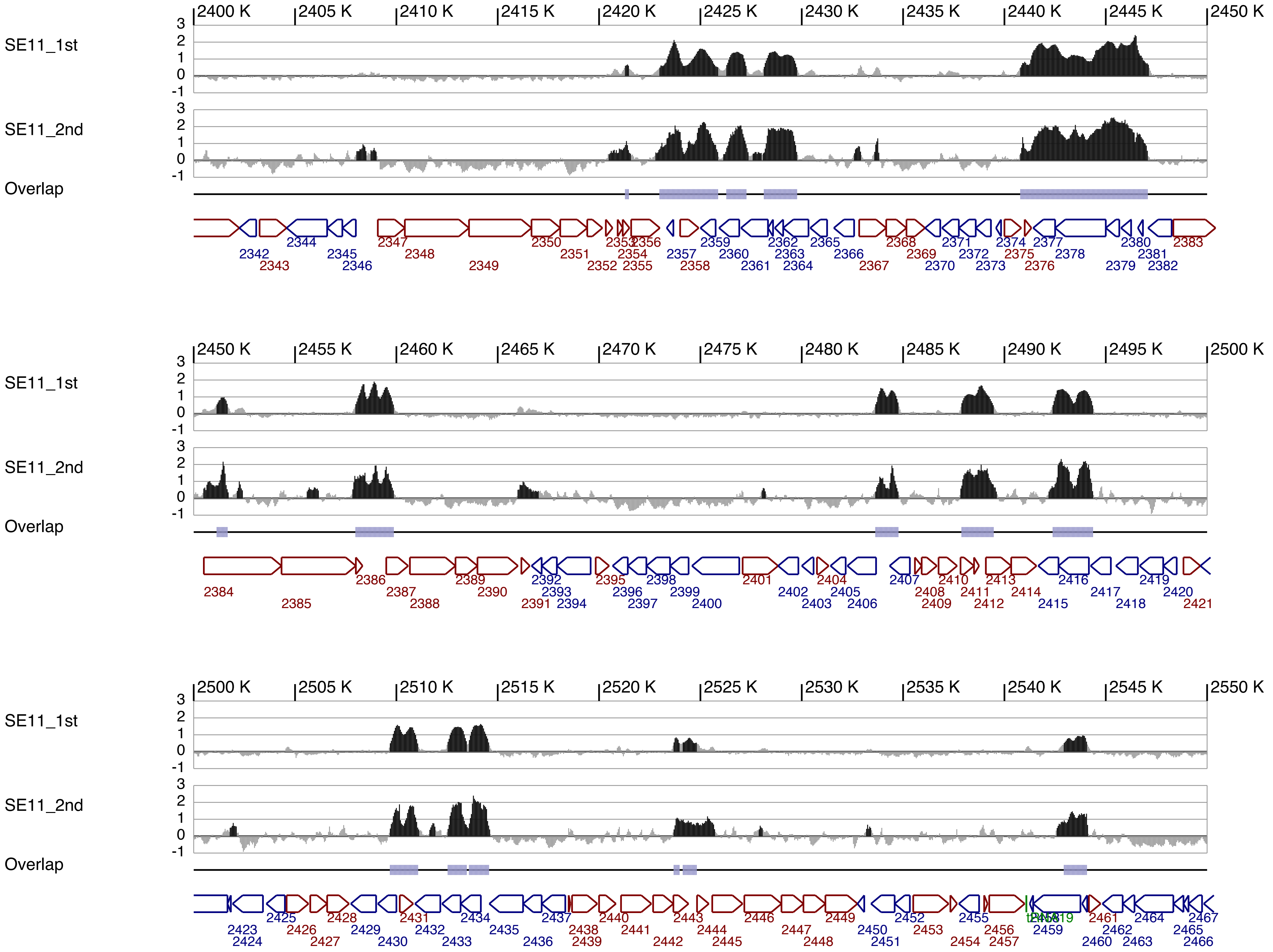
2400 K 2405 K 2410 K 2415 K 2420 K 2425 K 2430 K 2435 K 2440 K 2445 K 2450 K
SE11_1st
SE11_2nd
Overlap
3 2 1 0 -1
2344 2347 2350 2356 2359 2362 2365 2368 2371 2374 2377 2380 2383
2342 2345 2348 2351 2354 2357 2360 2363 2366 2369 2372 2375 2378 2381
2343 2346 2349 2352 2355 2358 2361 2364 2367 2370 2373 2376 2379 2382
2450 K 2455 K 2460 K 2465 K 2470 K 2475 K 2480 K 2485 K 2490 K 2495 K 2500 K
SE11_1st
SE11_2nd
Overlap
3 2 1 0 -1
2386 2389 2392 2395 2398 2401 2404 2407 2410 2413 2416 2419
2384 2387 2390 2393 2396 2399 2402 2405 2408 2411 2414 2417 2420
2385 2388 2391 2394 2397 2400 2403 2406 2409 2412 2415 2418 2421
2500 K 2505 K 2510 K 2515 K 2520 K 2525 K 2530 K 2535 K 2540 K 2545 K 2550 K
SE11_1st
SE11_2nd
Overlap
3 2 1 0 -1
2425 2428 2431 2434 2437 2440 2443 2446 2449 2452 2455 2461 2464 2467
2423 2426 2429 2432 2435 2438 2441 2444 2447 2450 2453 2456 2459 2462 2465
2424 2427 2430 2433 2436 2439 2442 2445 2448 2451 2454 2457 2460 2463 2466

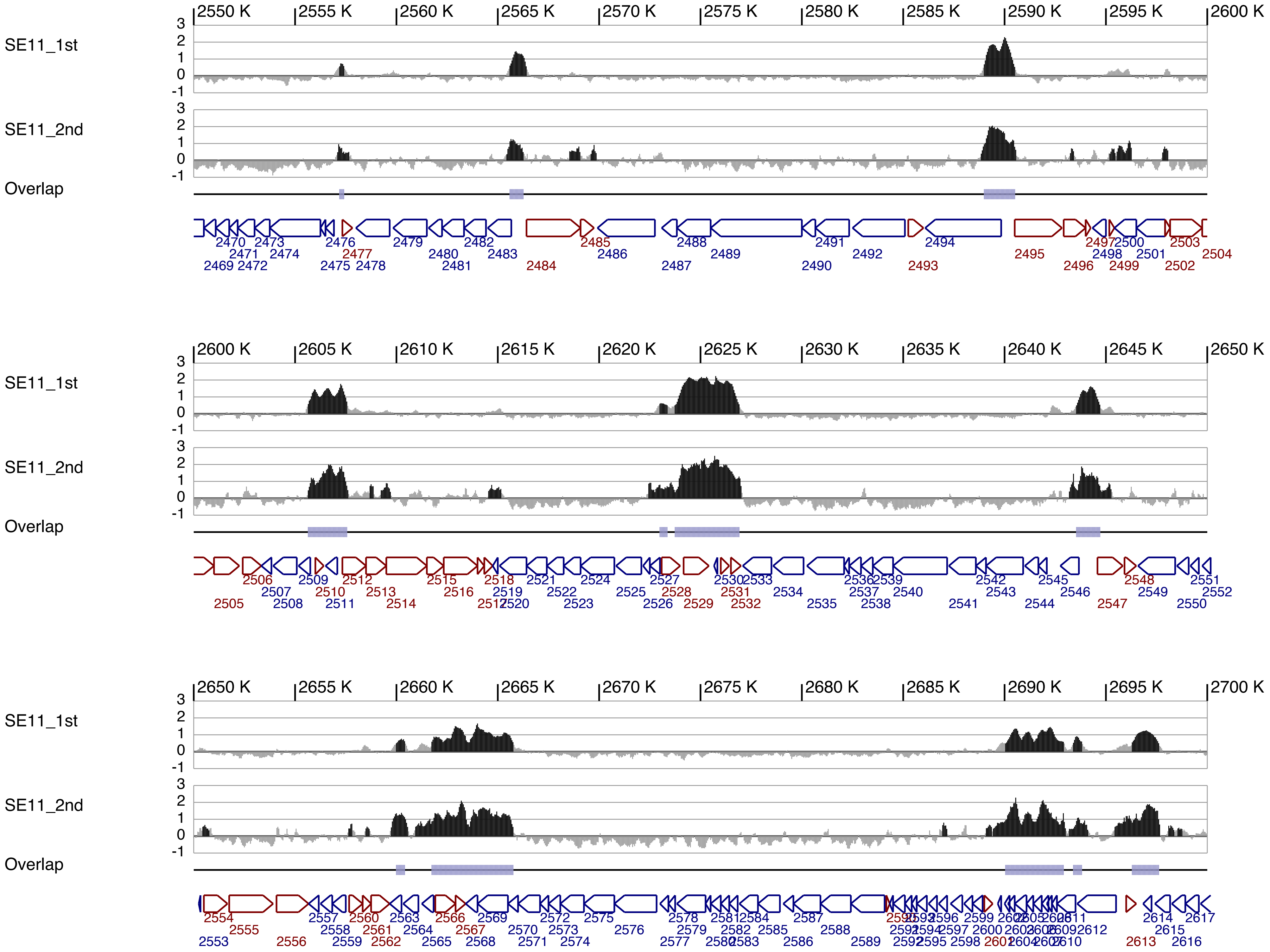

2550 K
2555 K
2560 K
2565 K
2570 K
2575 K
2580 K
2585 K
2590 K
2595 K
2600 K
SE11_1st
SE11_2nd
Overlap
3
2
1
0
-1
2470 2473 2476 2479 2482 2485 2488 2491 2494 2497 2500 2503
2471 2474 2477 2480 2483 2486 2489 2492 2495 2498 2501 2504
2469 2472 2475 2478 2481 2484 2487 2490 2493 2496 2499 2502
2605 K
2610 K
2615 K
2620 K
2625 K
2630 K
2635 K
2640 K
2645 K
2650 K
2506 2509 2512 2515 2518 2521 2524 2527 2530 2533 2536 2539 2542 2545 2548 2551
2507 2510 2513 2516 2519 2522 2525 2528 2531 2534 2537 2540 2543 2546 2549 2552
2505 2508 2511 2514 2517 2520 2523 2526 2529 2532 2535 2538 2541 2544 2547 2550
2655 K
2660 K
2665 K
2670 K
2675 K
2680 K
2685 K
2690 K
2695 K
2700 K
2554 2557 2560 2563 2566 2569 2572 2575 2578 2581 2584 2587 2596 2599 2611 2614 2617
2555 2558 2561 2564 2567 2570 2573 2576 2579 2582 2585 2588 2594 2597 2600 2612 2615
2553 2556 2559 2562 2565 2568 2571 2574 2577 2583 2586 2589 2595 2598 2601 2604 2610 2613 2616

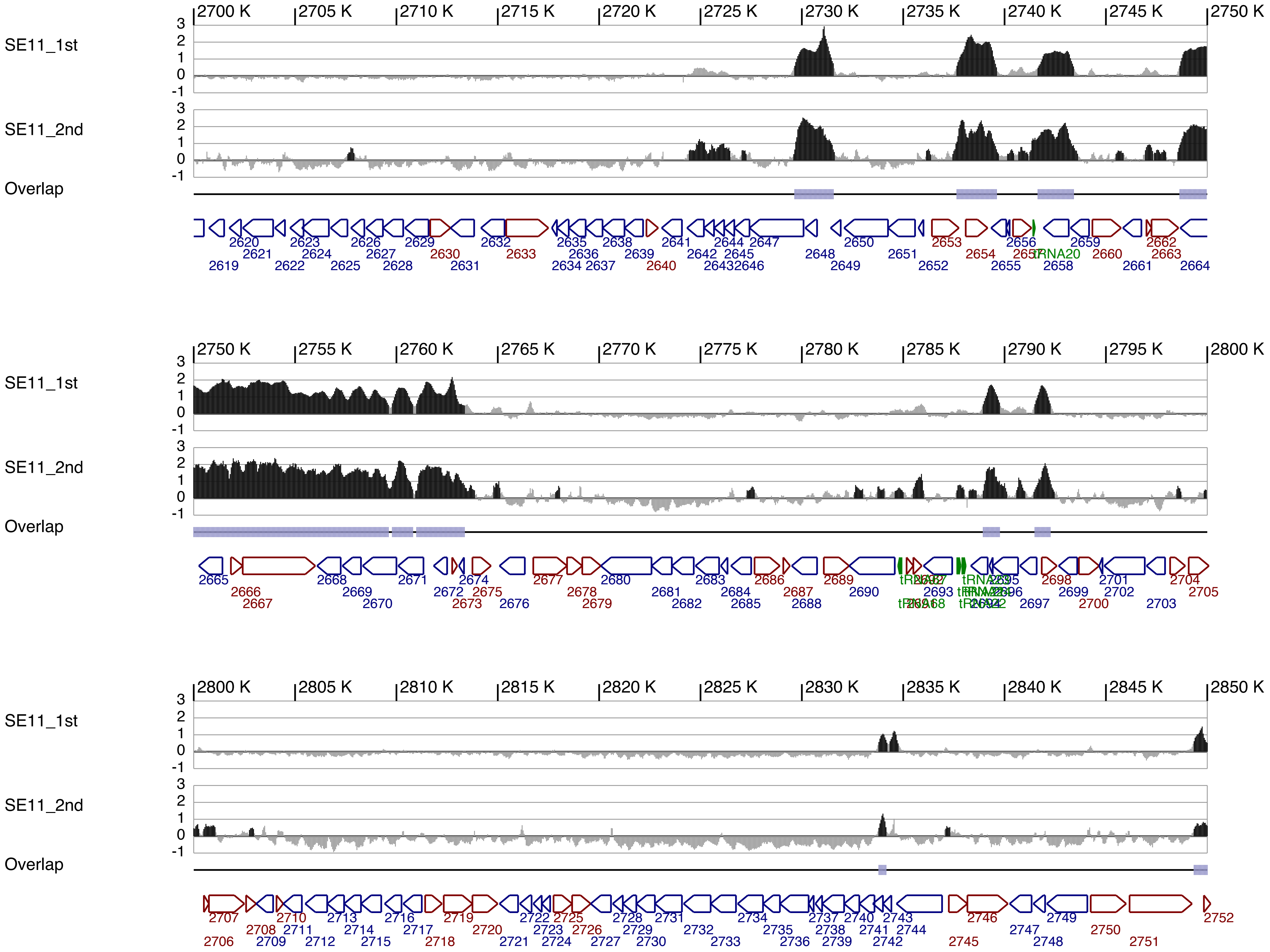

2700 K
2705 K
2710 K
2715 K
2720 K
2725 K
2730 K
2735 K
2740 K
2745 K
2750 K
SE11_1st
SE11_2nd
Overlap
3
2
1
0
-1
2620 2623 2626 2629 2632 2635 2638 2641 2644 2647 2650 2653 2656 2659 2662
2621 2624 2627 2630 2633 2636 2639 2642 2645 2648 2651 2654 2657 tRNA20 2660 2663
2619 2622 2625 2628 2631 2634 2637 2640 2643 2646 2649 2652 2655 2658 2661 2664
2755 K
2760 K
2765 K
2770 K
2775 K
2780 K
2785 K
2790 K
2795 K
2800 K
2665 2668 2671 2674 2677 2680 2683 2686 2689 2698 2701 2704
2666 2669 2672 2675 2678 2681 2684 2687 2690 2693 2699 2702 2705
2667 2670 2673 2676 2679 2682 2685 2688 2697 2700 2703
2805 K
2810 K
2815 K
2820 K
2825 K
2830 K
2835 K
2840 K
2845 K
2850 K
2707 2710 2713 2716 2719 2722 2725 2728 2731 2734 2737 2740 2743 2746 2749 2752
2708 2711 2714 2717 2720 2723 2726 2729 2732 2735 2738 2741 2744 2747 2750
2706 2709 2712 2715 2718 2721 2724 2727 2730 2733 2736 2739 2742 2745 2748 2751

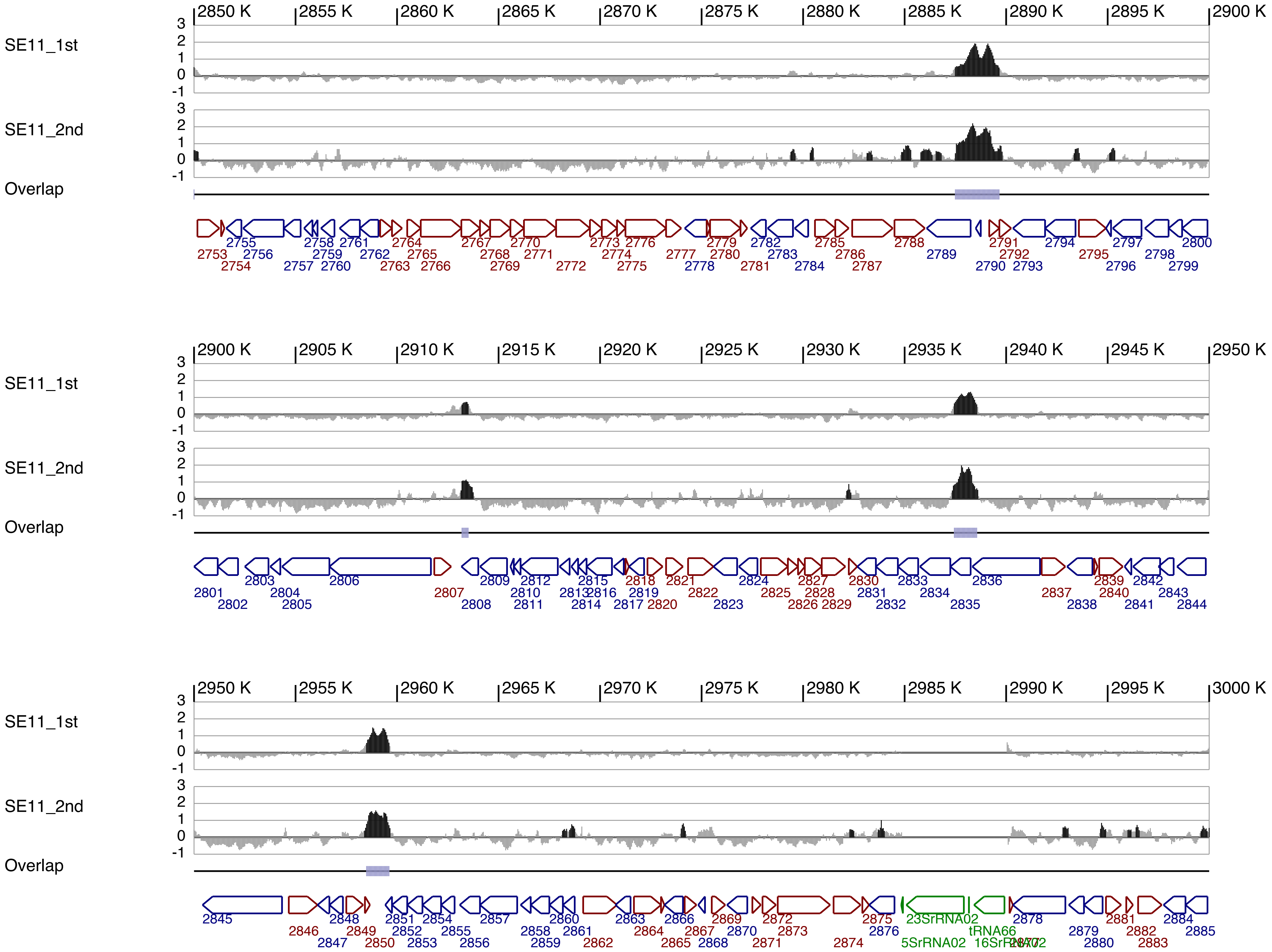
2850 K 2855 K 2860 K 2865 K 2870 K 2875 K 2880 K 2885 K 2890 K 2895 K 2900 K
SE11_1st
SE11_2nd
Overlap
3 2 1 0 -1
2755 2758 2761 2764 2767 2770 2773 2776 2779 2782 2785 2788 2791 2794 2797 2800
2753 2756 2759 2762 2765 2768 2771 2774 2777 2780 2783 2786 2789 2792 2795 2798
2754 2757 2760 2763 2766 2769 2772 2775 2778 2781 2784 2787 2790 2793 2796 2799
2900 K 2905 K 2910 K 2915 K 2920 K 2925 K 2930 K 2935 K 2940 K 2945 K 2950 K
SE11_1st
SE11_2nd
Overlap
2803 2806 2809 2812 2815 2818 2821 2824 2827 2830 2833 2836 2839 2842
2801 2804 2807 2810 2813 2816 2819 2822 2825 2828 2831 2834 2837 2840 2843
2802 2805 2808 2811 2814 2817 2820 2823 2826 2829 2832 2835 2838 2841 2844
2950 K 2955 K 2960 K 2965 K 2970 K 2975 K 2980 K 2985 K 2990 K 2995 K 3000 K
SE11_1st
SE11_2nd
Overlap
2845 2848 2851 2854 2857 2860 2863 2866 2869 2872 2875 23SrRNA02 2878 2881 2884
2846 2849 2852 2855 2858 2861 2864 2867 2870 2873 2876 tRNA66 2879 2882 2885
2847 2850 2853 2856 2859 2862 2865 2868 2871 2874 5SrRNA02 16SrRNA02 2880 2883

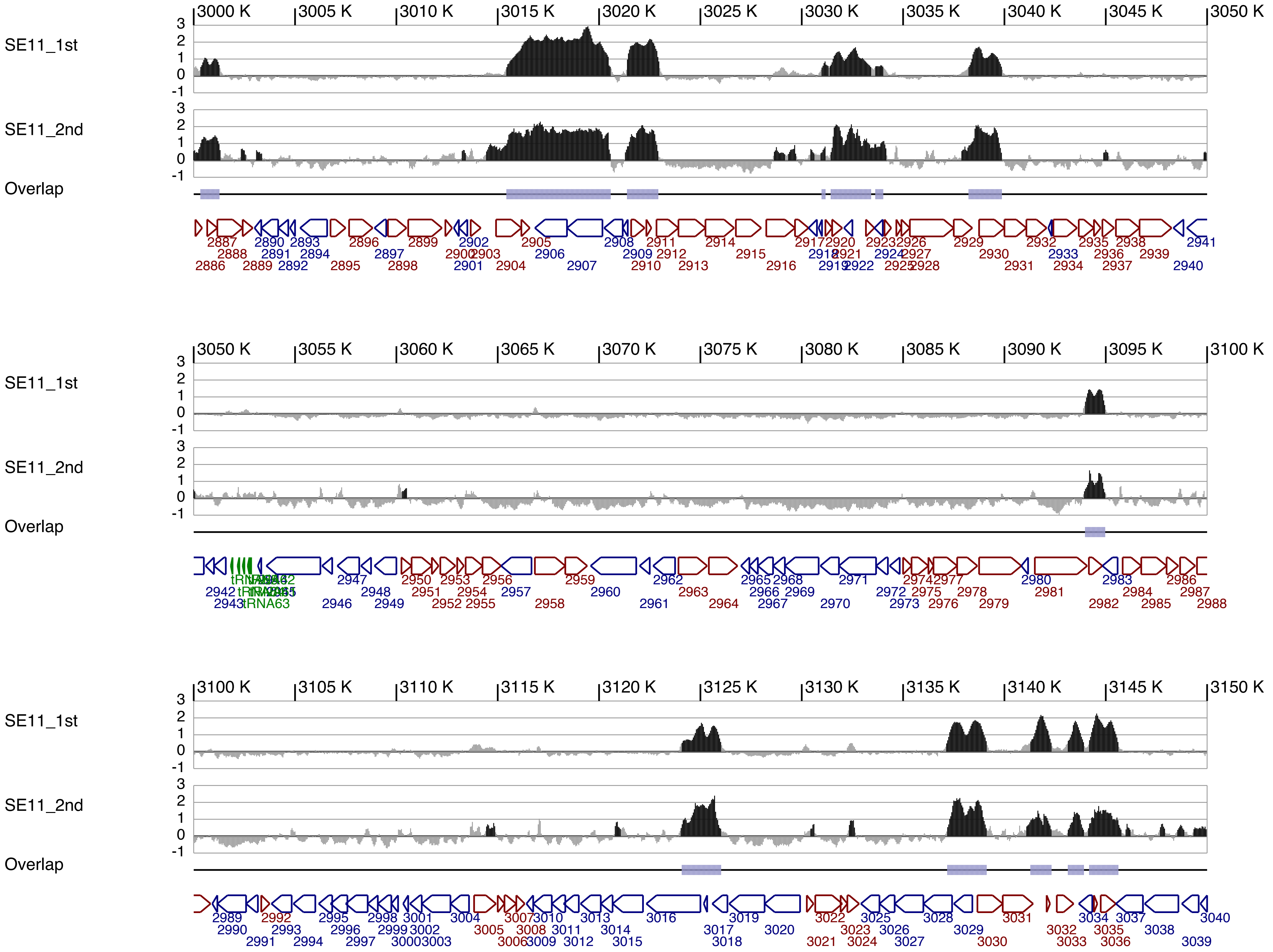
3000 K
3005 K
3010 K
3015 K
3020 K
3025 K
3030 K
3035 K
3040 K
3045 K
3050 K
SE11_1st
SE11_2nd
Overlap
3050 K
3055 K
3060 K
3065 K
3070 K
3075 K
3080 K
3085 K
3090 K
3095 K
3100 K
SE11_1st
SE11_2nd
Overlap
3100 K
3105 K
3110 K
3115 K
3120 K
3125 K
3130 K
3135 K
3140 K
3145 K
3150 K
SE11_1st
SE11_2nd
Overlap

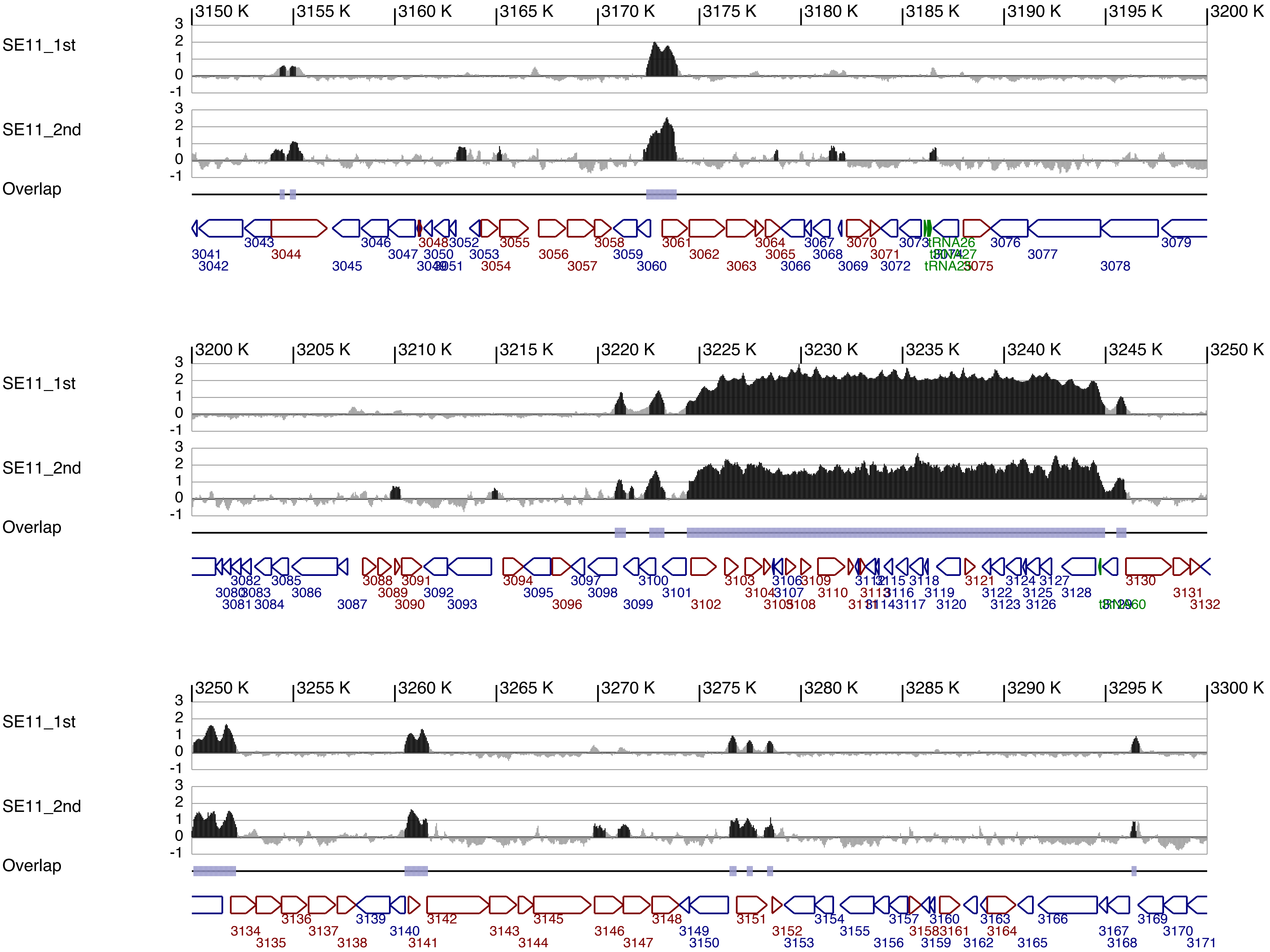

3150 K
3155 K
3160 K
3165 K
3170 K
3175 K
3180 K
3185 K
3190 K
3195 K
3200 K
SE11_1st
SE11_2nd
Overlap
3041
3042
3043
3044
3045
3046
3047
3048
3050
3052
3053
3054
3055
3056
3057
3058
3059
3060
3061
3062
3063
3064
3065
3066
3067
3068
3069
3070
3071
3072
3073
tRNA26
3075
3076
3077
3078
3079
3200 K
3205 K
3210 K
3215 K
3220 K
3225 K
3230 K
3235 K
3240 K
3245 K
3250 K
SE11_1st
SE11_2nd
Overlap
3082
3081
3084
3083
3085
3086
3087
3088
3089
3090
3091
3092
3093
3094
3095
3096
3097
3098
3099
3100
3101
3102
3103
3104
3106
3107
3108
3109
3110
3112
3115
3116
3117
3118
3119
3120
3121
3122
3123
3124
3125
3126
3127
3128
3130
3131
3132
3250 K
3255 K
3260 K
3265 K
3270 K
3275 K
3280 K
3285 K
3290 K
3295 K
3300 K
SE11_1st
SE11_2nd
Overlap
3134
3135
3136
3137
3138
3139
3140
3141
3142
3143
3144
3145
3146
3147
3148
3149
3150
3151
3152
3153
3154
3155
3156
3157
3159
3160
3162
3163
3164
3165
3166
3167
3168
3169
3170
3171

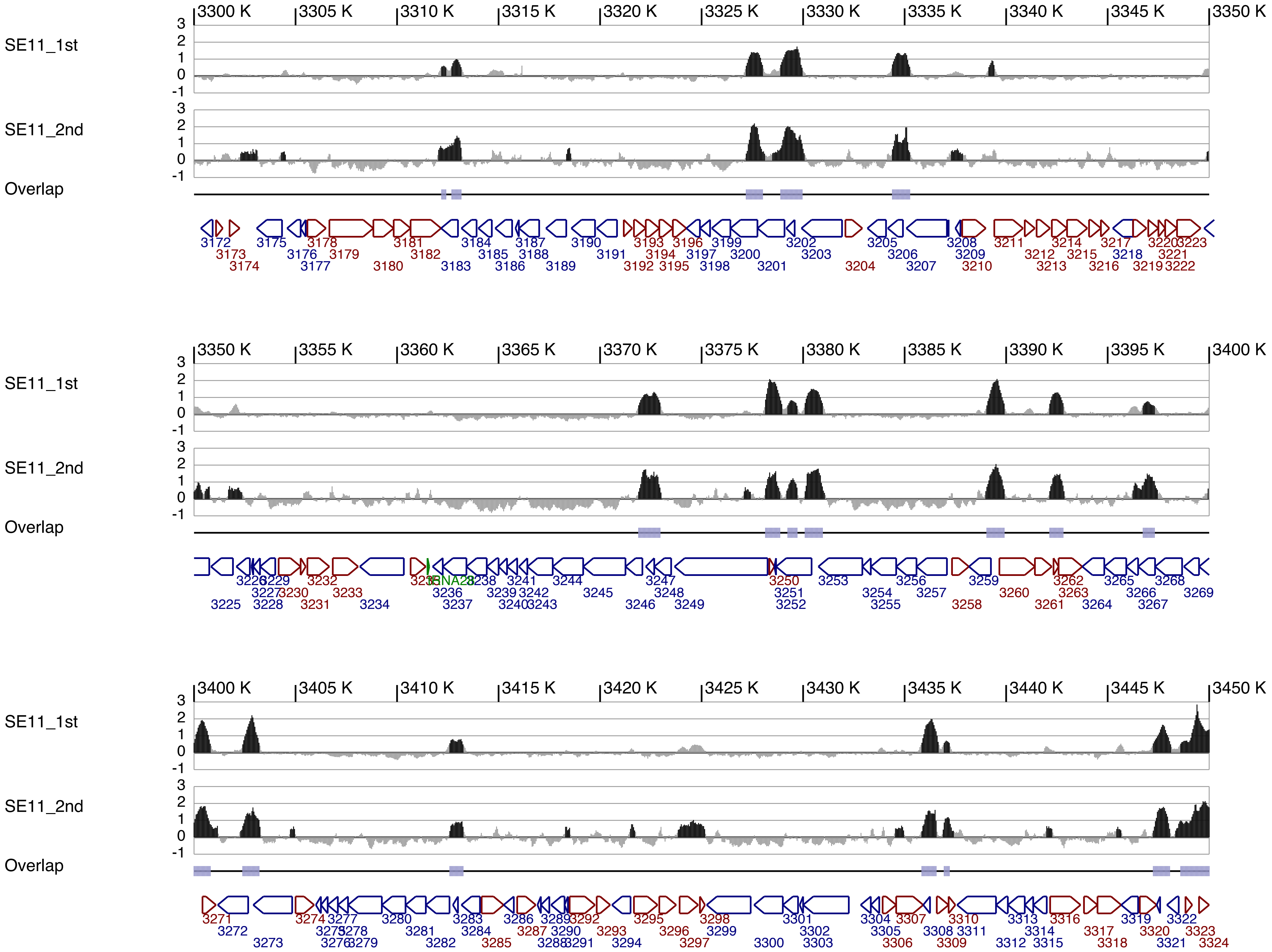

3300 K
3305 K
3310 K
3315 K
3320 K
3325 K
3330 K
3335 K
3340 K
3345 K
3350 K
SE11_1st
SE11_2nd
Overlap
3172
3173
3174
3175
3176
3177
3178
3179
3180
3181
3182
3183
3184
3185
3186
3187
3188
3189
3190
3191
3192
3193
3194
3195
3196
3197
3198
3199
3200
3201
3202
3203
3204
3205
3206
3207
3208
3209
3210
3211
3212
3213
3214
3215
3216
3217
3218
3219
3220
3221
3222
3223
3350 K
3355 K
3360 K
3365 K
3370 K
3375 K
3380 K
3385 K
3390 K
3395 K
3400 K
SE11_1st
SE11_2nd
Overlap
3225
3226
3227
3228
3229
3230
3231
3232
3233
3234
3236
3237
3238
3239
3240
3241
3242
3243
3244
3245
3246
3247
3248
3249
3250
3251
3252
3253
3254
3255
3256
3257
3258
3259
3260
3261
3262
3263
3264
3265
3266
3267
3268
3269
3400 K
3405 K
3410 K
3415 K
3420 K
3425 K
3430 K
3435 K
3440 K
3445 K
3450 K
SE11_1st
SE11_2nd
Overlap
3271
3272
3273
3274
3277
3278
3279
3280
3281
3282
3283
3284
3285
3286
3287
3290
3292
3293
3294
3295
3296
3297
3298
3299
3300
3301
3302
3303
3304
3305
3306
3307
3308
3309
3310
3311
3312
3313
3314
3315
3316
3317
3318
3319
3320
3321
3322
3323
3324

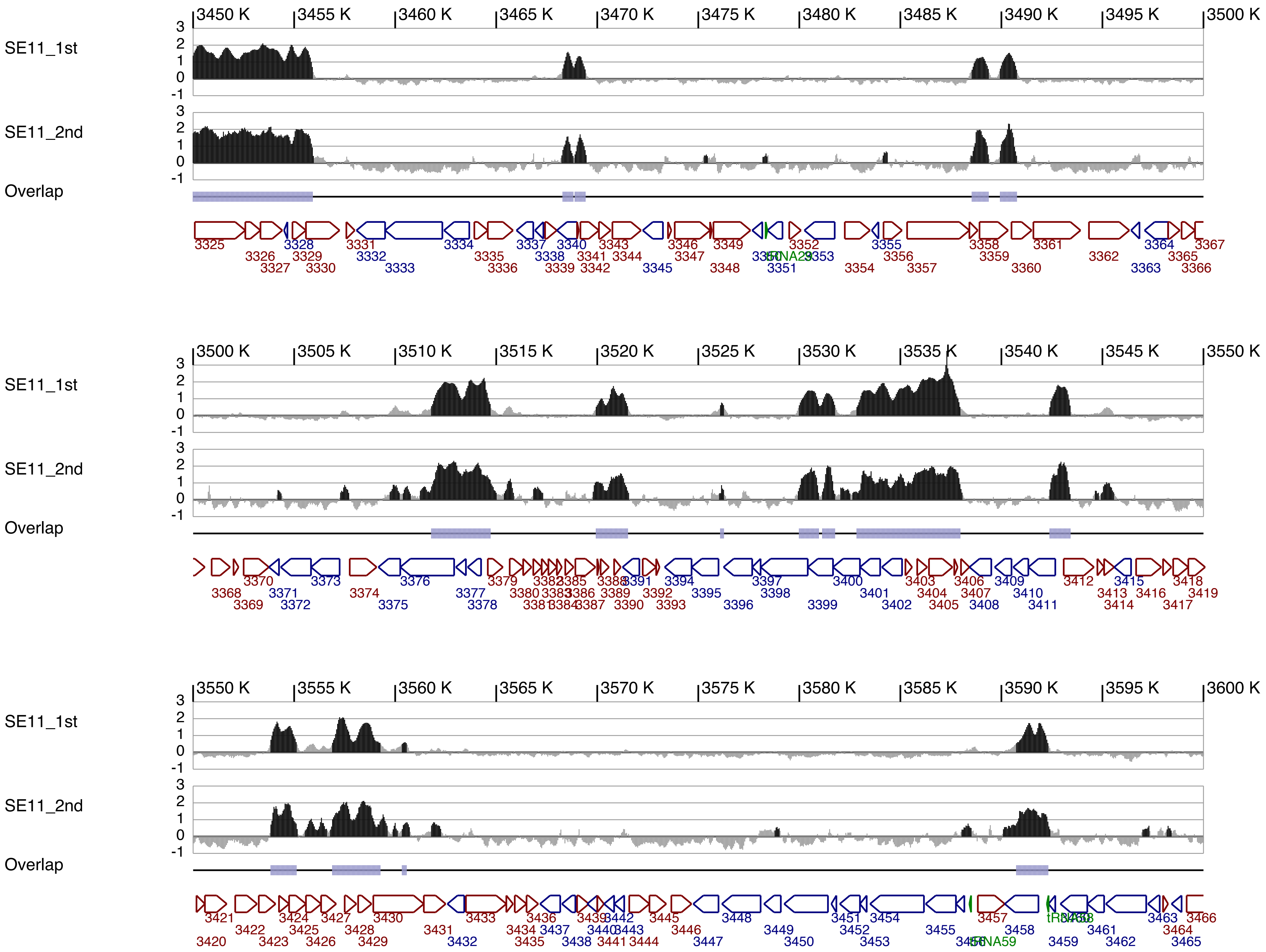

3450 K
3455 K
3460 K
3465 K
3470 K
3475 K
3480 K
3485 K
3490 K
3495 K
3500 K
SE11_1st
SE11_2nd
Overlap
3
2
1
0
-1
3325 3326 3327 3328 3329 3330 3331 3332 3333 3334 3335 3336 3337 3338 3339 3340 3341 3342 3343 3344 3345 3346 3347 3348 3349 3351 3352 3353 3354 3355 3356 3357 3358 3359 3360 3361 3362 3363 3364 3365 3366 3367
3500 K
3505 K
3510 K
3515 K
3520 K
3525 K
3530 K
3535 K
3540 K
3545 K
3550 K
SE11_1st
SE11_2nd
Overlap
3368 3369 3370 3371 3372 3373 3374 3375 3376 3377 3378 3379 3380 3385 3386 3387 3389 3390 3391 3392 3393 3394 3395 3396 3397 3398 3399 3400 3401 3402 3403 3404 3405 3406 3407 3408 3409 3410 3411 3412 3413 3414 3415 3416 3417 3418 3419
3550 K
3555 K
3560 K
3565 K
3570 K
3575 K
3580 K
3585 K
3590 K
3595 K
3600 K
SE11_1st
SE11_2nd
Overlap
3420 3421 3422 3423 3424 3425 3426 3427 3428 3429 3430 3431 3432 3433 3434 3435 3436 3437 3438 3441 3442 3443 3444 3445 3446 3447 3448 3449 3450 3451 3452 3453 3454 3455 3457 3458 3459 3461 3462 3463 3464 3465 3466

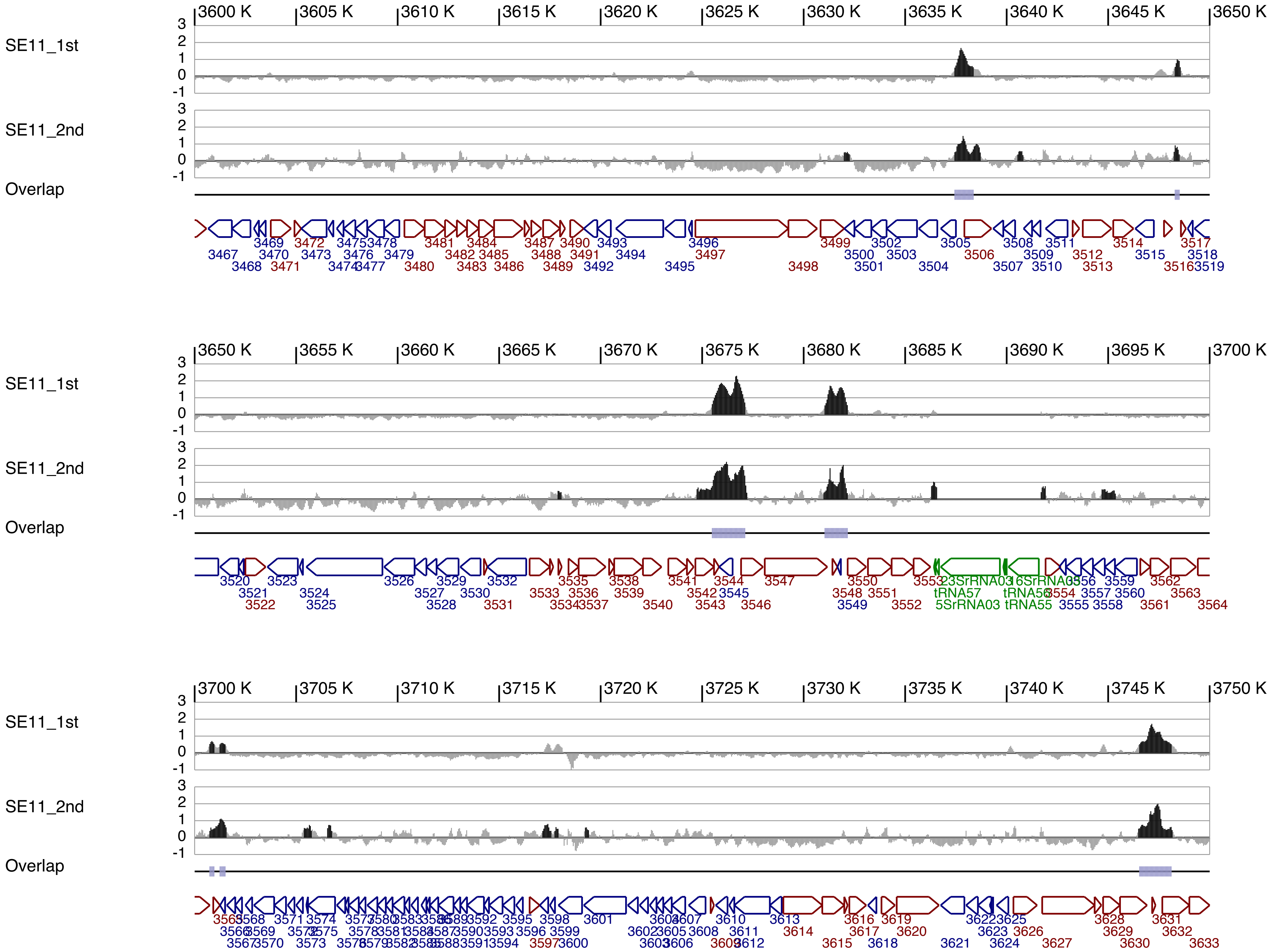

SE11_1st
SE11_2nd
Overlap
3600 K
3605 K
3610 K
3615 K
3620 K
3625 K
3630 K
3635 K
3640 K
3645 K
3650 K
3655 K
3660 K
3665 K
3670 K
3675 K
3680 K
3685 K
3690 K
3695 K
3700 K
3705 K
3710 K
3715 K
3720 K
3725 K
3730 K
3735 K
3740 K
3745 K
3750 K
23SrRNA03
16SrRNA03
5SrRNA03
tRNA57
tRNA56
tRNA55

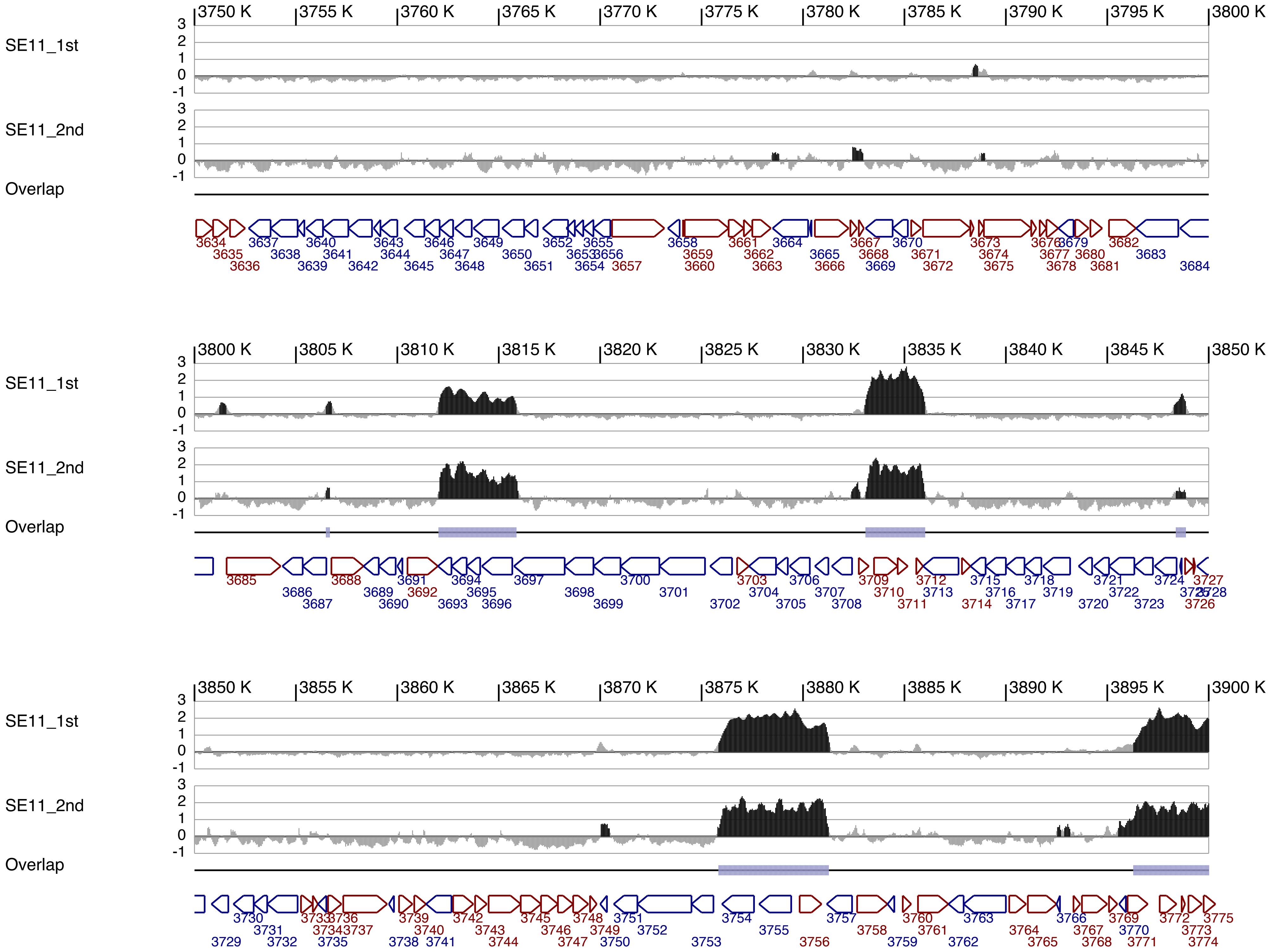

3750 K
3755 K
3760 K
3765 K
3770 K
3775 K
3780 K
3785 K
3790 K
3795 K
3800 K
SE11_1st
SE11_2nd
Overlap
3800 K
3805 K
3810 K
3815 K
3820 K
3825 K
3830 K
3835 K
3840 K
3845 K
3850 K
SE11_1st
SE11_2nd
Overlap
3850 K
3855 K
3860 K
3865 K
3870 K
3875 K
3880 K
3885 K
3890 K
3895 K
3900 K
SE11_1st
SE11_2nd
Overlap

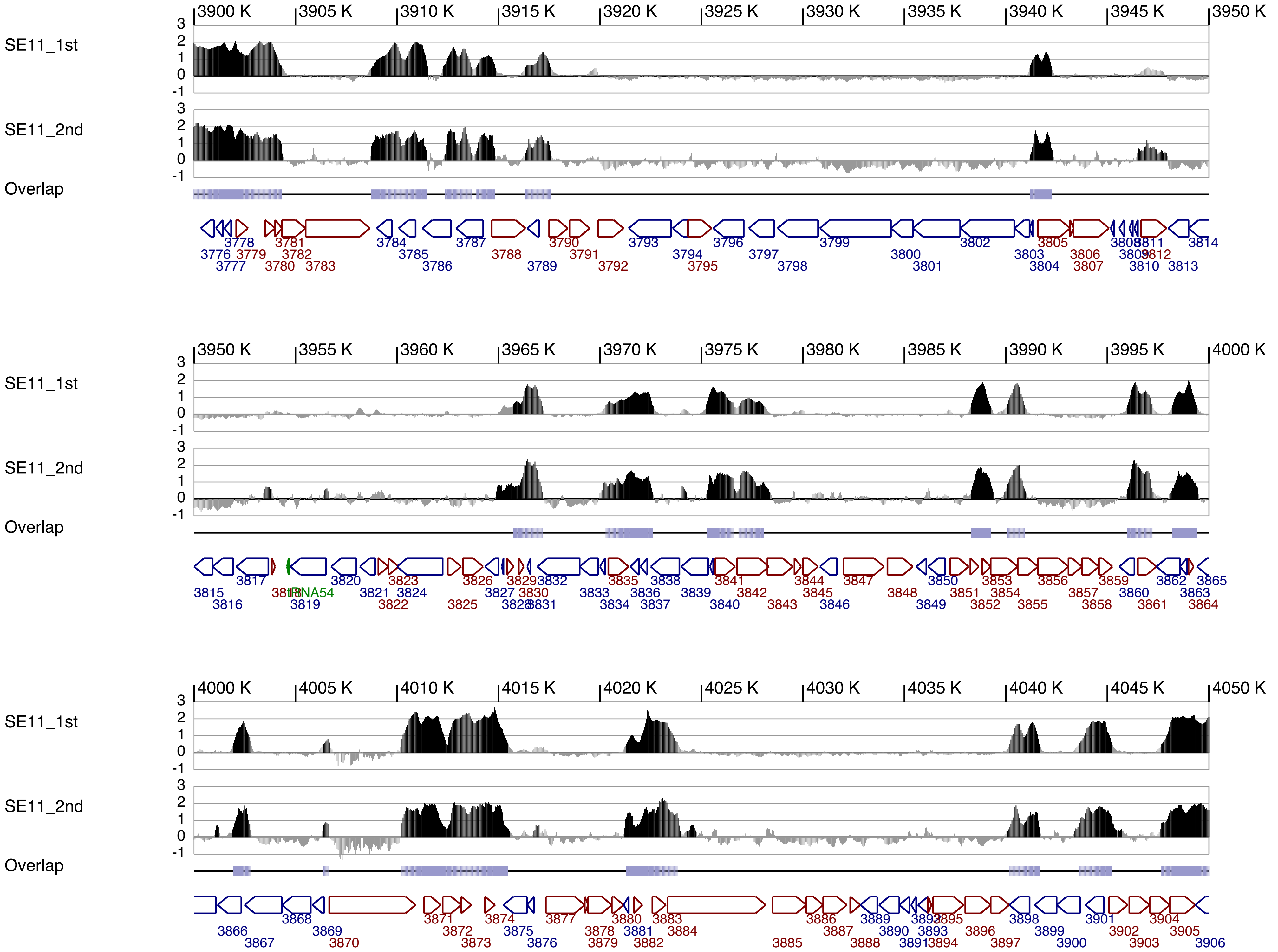
3900 K
3905 K
3910 K
3915 K
3920 K
3925 K
3930 K
3935 K
3940 K
3945 K
3950 K
SE11_1st
SE11_2nd
Overlap
3
2
1
0
-1
3776 3777 3778 3779 3780 3781 3782 3783 3784 3785 3786 3787 3788 3789 3790 3791 3792 3793 3794 3795 3796 3797 3798 3799 3800 3801 3802 3803 3804 3805 3806 3807 3808 3809 3810 3811 3812 3813 3814
3950 K
3955 K
3960 K
3965 K
3970 K
3975 K
3980 K
3985 K
3990 K
3995 K
4000 K
SE11_1st
SE11_2nd
Overlap
3815 3816 3817 3818 tRNA54 3819 3820 3821 3822 3823 3824 3825 3826 3827 3828 3829 3830 3831 3832 3833 3834 3835 3836 3837 3838 3839 3840 3841 3842 3843 3844 3845 3846 3847 3848 3849 3850 3851 3852 3853 3854 3855 3856 3857 3858 3859 3860 3861 3862 3863 3864 3865
4000 K
4005 K
4010 K
4015 K
4020 K
4025 K
4030 K
4035 K
4040 K
4045 K
4050 K
SE11_1st
SE11_2nd
Overlap
3866 3867 3868 3869 3870 3871 3872 3873 3874 3875 3876 3877 3878 3879 3880 3881 3882 3883 3884 3885 3886 3887 3888 3889 3890 3891 3892 3893 3894 3895 3896 3897 3898 3899 3900 3901 3902 3903 3904 3905 3906

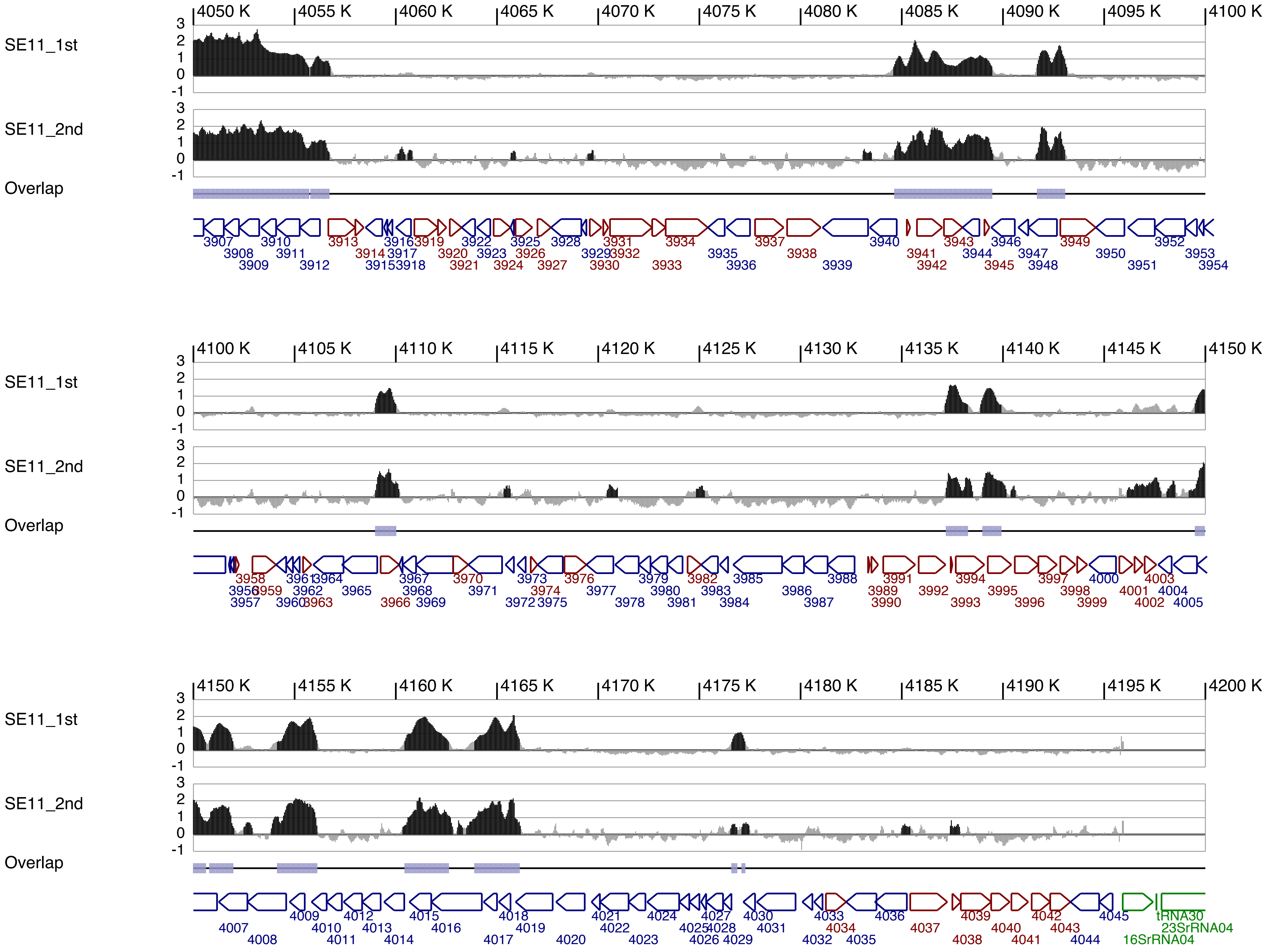
4050 K
4055 K
4060 K
4065 K
4070 K
4075 K
4080 K
4085 K
4090 K
4095 K
4100 K
SE11_1st
SE11_2nd
Overlap
3907 3908 3909 3910 3911 3912 3913 3914 3915 3916 3917 3918 3919 3920 3921 3922 3923 3924 3925 3926 3927 3928 3929 3930 3931 3932 3933 3934 3935 3936 3937 3938 3939 3940 3941 3942 3943 3944 3945 3946 3947 3948 3949 3950 3951 3952 3953 3954
4100 K
4105 K
4110 K
4115 K
4120 K
4125 K
4130 K
4135 K
4140 K
4145 K
4150 K
SE11_1st
SE11_2nd
Overlap
3956 3957 3958 3959 3960 3961 3962 3963 3964 3965 3966 3967 3968 3969 3970 3971 3972 3973 3974 3975 3976 3977 3978 3979 3980 3981 3982 3983 3984 3985 3986 3987 3988 3989 3990 3991 3992 3993 3994 3995 3996 3997 3998 3999 4000 4001 4002 4003 4004 4005
4150 K
4155 K
4160 K
4165 K
4170 K
4175 K
4180 K
4185 K
4190 K
4195 K
4200 K
SE11_1st
SE11_2nd
Overlap
4007 4008 4009 4010 4011 4012 4013 4014 4015 4016 4017 4018 4019 4020 4021 4022 4023 4024 4025 4026 4027 4028 4029 4030 4031 4032 4033 4034 4035 4036 4037 4038 4039 4040 4041 4042 4043 4044 4045
tRNA30
23SrRNA04
16SrRNA04

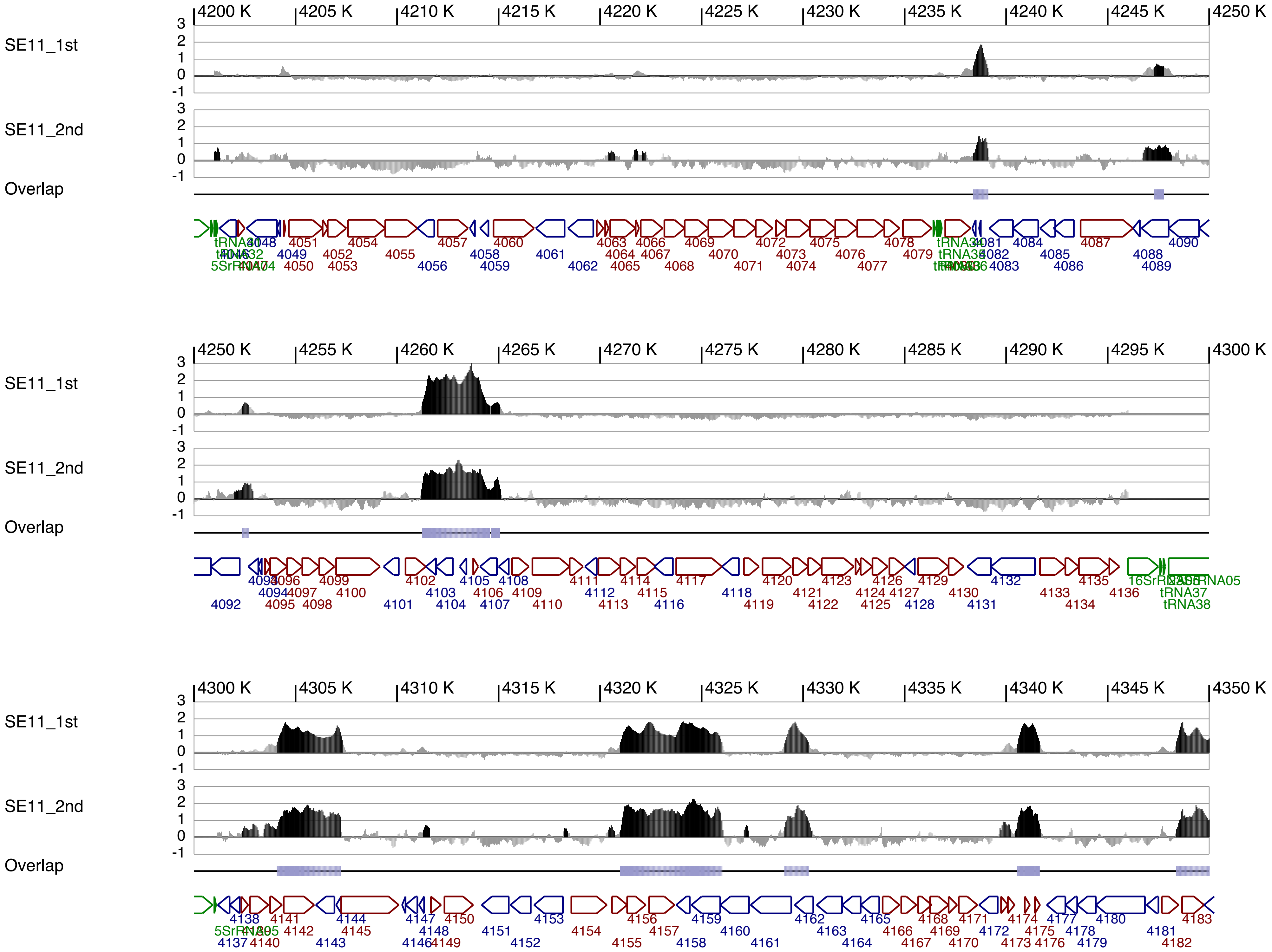

4200 K
4205 K
4210 K
4215 K
4220 K
4225 K
4230 K
4235 K
4240 K
4245 K
4250 K
SE11_1st
SE11_2nd
Overlap
3
2
1
0
-1
4048
4049
4050
4051
4052
4053
4054
4055
4056
4057
4058
4059
4060
4061
4062
4063
4064
4065
4066
4067
4068
4069
4070
4071
4072
4073
4074
4075
4076
4077
4078
4079
4081
4082
4083
4084
4085
4086
4087
4088
4089
4090
4250 K
4255 K
4260 K
4265 K
4270 K
4275 K
4280 K
4285 K
4290 K
4295 K
4300 K
SE11_1st
SE11_2nd
Overlap
4092
4094
4095
4096
4097
4098
4099
4100
4101
4102
4103
4104
4105
4106
4107
4108
4109
4110
4111
4112
4113
4114
4115
4116
4117
4118
4119
4120
4121
4122
4123
4124
4125
4126
4127
4128
4129
4130
4131
4132
4133
4134
4135
4136
tRNA37
tRNA38
4300 K
4305 K
4310 K
4315 K
4320 K
4325 K
4330 K
4335 K
4340 K
4345 K
4350 K
SE11_1st
SE11_2nd
Overlap
4137
4138
4140
4141
4142
4143
4144
4145
4146
4147
4148
4149
4150
4151
4152
4153
4154
4155
4156
4157
4158
4159
4160
4161
4162
4163
4164
4165
4166
4167
4168
4169
4170
4171
4172
4173
4174
4175
4176
4177
4178
4179
4180
4181
4182
4183

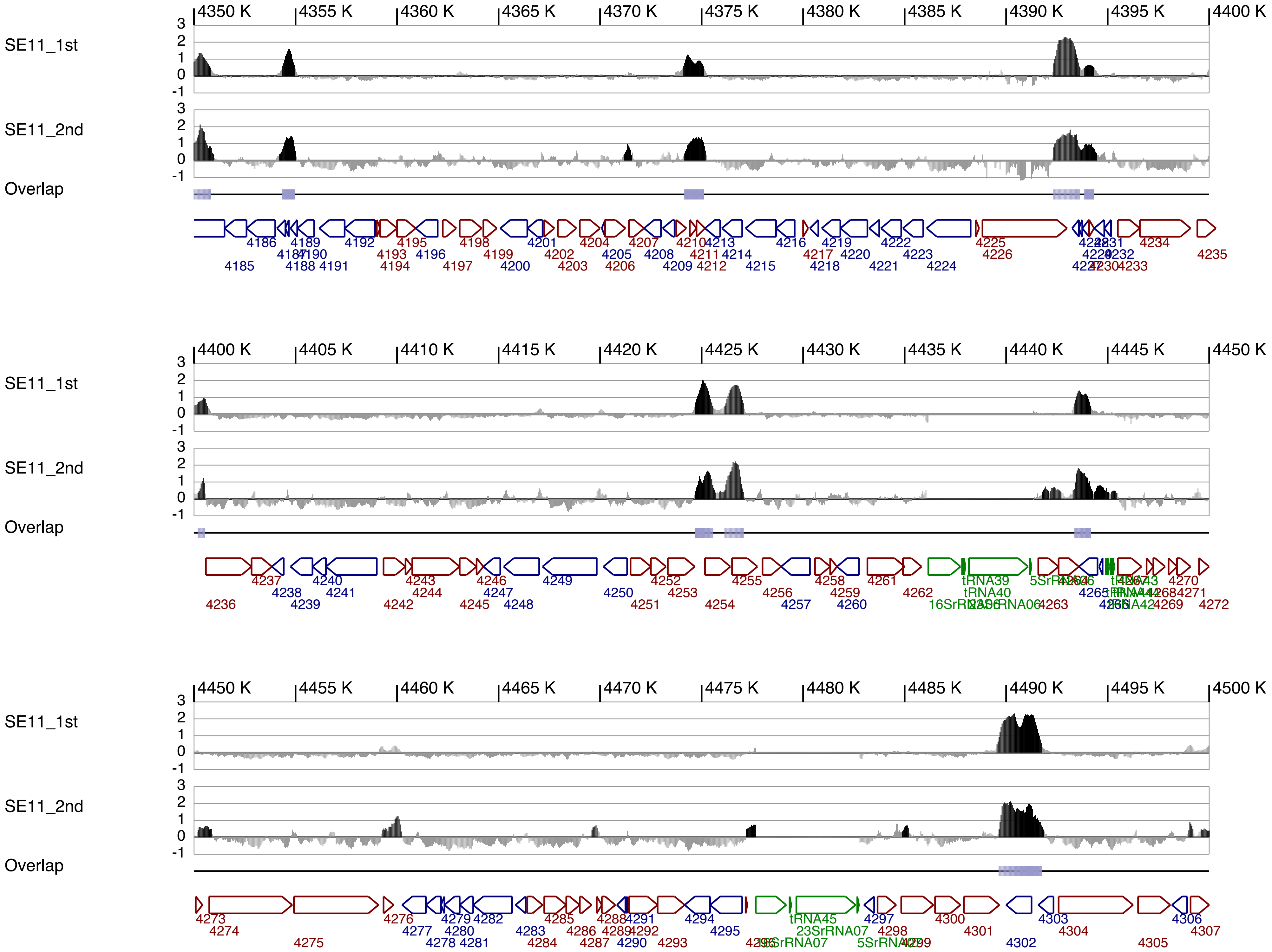

4350 K
4355 K
4360 K
4365 K
4370 K
4375 K
4380 K
4385 K
4390 K
4395 K
4400 K
SE11_1st
SE11_2nd
Overlap
3
2
1
0
-1
4185
4186
4188
4189
4191
4192
4193
4194
4195
4196
4197
4198
4199
4200
4201
4202
4203
4204
4205
4206
4207
4208
4209
4212
4213
4214
4215
4216
4217
4218
4219
4220
4221
4222
4223
4224
4225
4226
4232
4233
4234
4235
4405 K
4410 K
4415 K
4420 K
4425 K
4430 K
4435 K
4440 K
4445 K
4450 K
4236
4237
4238
4239
4240
4241
4242
4243
4244
4245
4246
4247
4248
4249
4250
4251
4252
4253
4254
4255
4256
4257
4258
4259
4260
4261
4262
tRNA39
tRNA40
4263
4269
4270
4271
4272
4455 K
4460 K
4465 K
4470 K
4475 K
4480 K
4485 K
4490 K
4495 K
4500 K
4273
4274
4275
4276
4277
4278
4280
4281
4282
4283
4284
4285
4286
4287
4290
4292
4293
4294
4295
tRNA45
23SrRNA07
4297
4298
4300
4301
4302
4303
4304
4305
4306
4307

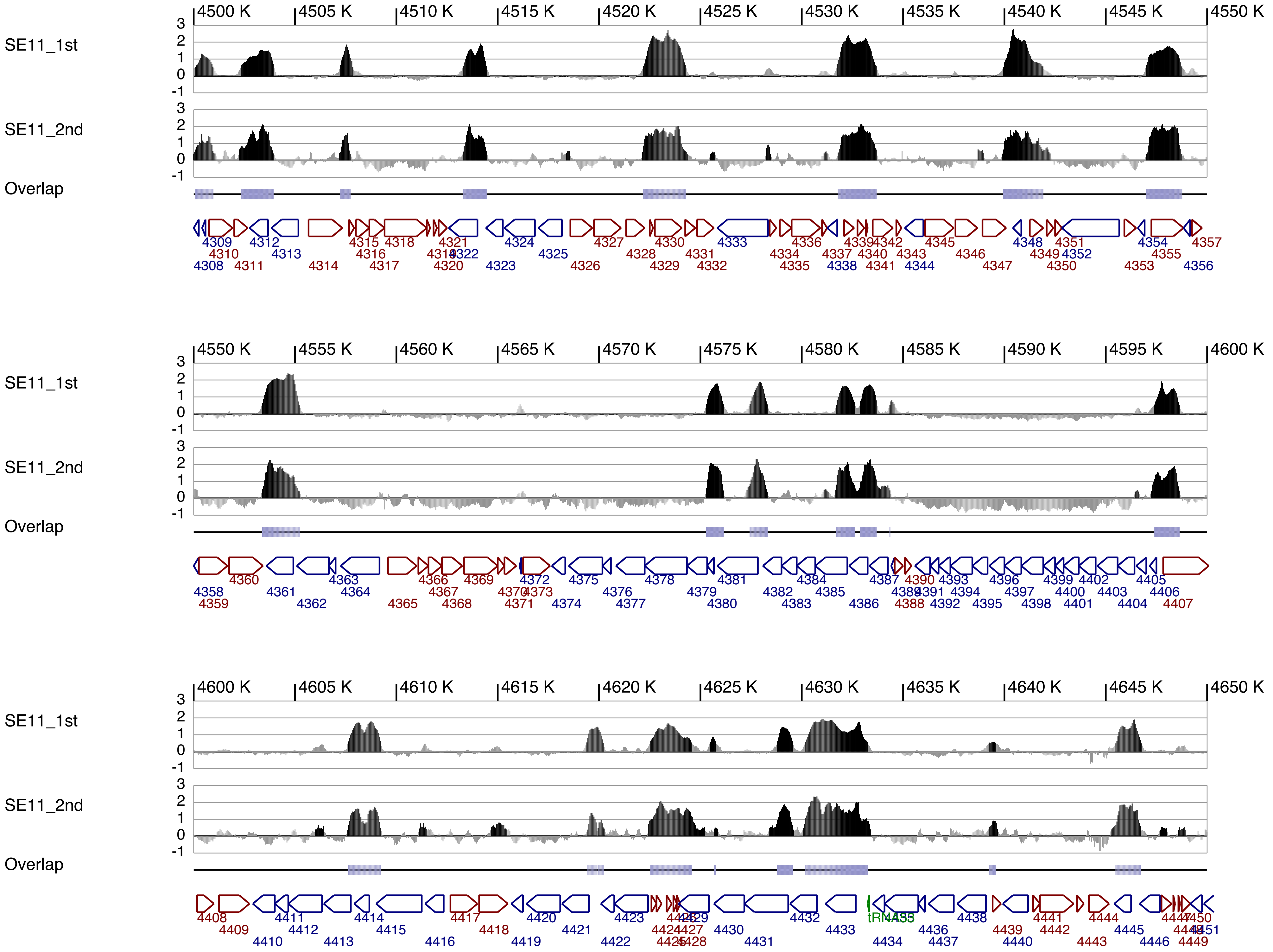

4500 K
4505 K
4510 K
4515 K
4520 K
4525 K
4530 K
4535 K
4540 K
4545 K
4550 K
SE11_1st
SE11_2nd
Overlap
3
2
1
0
-1
4309 4312 4315 4318 4321 4324 4327 4330 4333 4336 4339 4342 4345 4348 4351 4354 4357
4310 4313 4316 4319 4322 4325 4328 4331 4334 4337 4340 4343 4346 4349 4352 4355
4308 4311 4314 4317 4320 4323 4326 4329 4332 4335 4338 4341 4344 4347 4350 4353 4356
4550 K
4555 K
4560 K
4565 K
4570 K
4575 K
4580 K
4585 K
4590 K
4595 K
4600 K
SE11_1st
SE11_2nd
Overlap
4360 4363 4366 4369 4372 4375 4378 4381 4384 4387 4390 4393 4396 4399 4402 4405
4358 4361 4364 4367 4370 4373 4376 4379 4382 4385 4389 4391 4394 4397 4400 4403 4406
4359 4362 4365 4368 4371 4374 4377 4380 4383 4386 4388 4392 4395 4398 4401 4404 4407
4600 K
4605 K
4610 K
4615 K
4620 K
4625 K
4630 K
4635 K
4640 K
4645 K
4650 K
SE11_1st
SE11_2nd
Overlap
4408 4411 4414 4417 4420 4423 4426 4429 4432 tRNA 4435 4438 4441 4444 4447 4450
4409 4412 4415 4418 4421 4424 4427 4430 4433 4436 4439 4442 4445 4448 4451
4410 4413 4416 4419 4422 4425 4428 4431 4434 4437 4440 4443 4446 4449

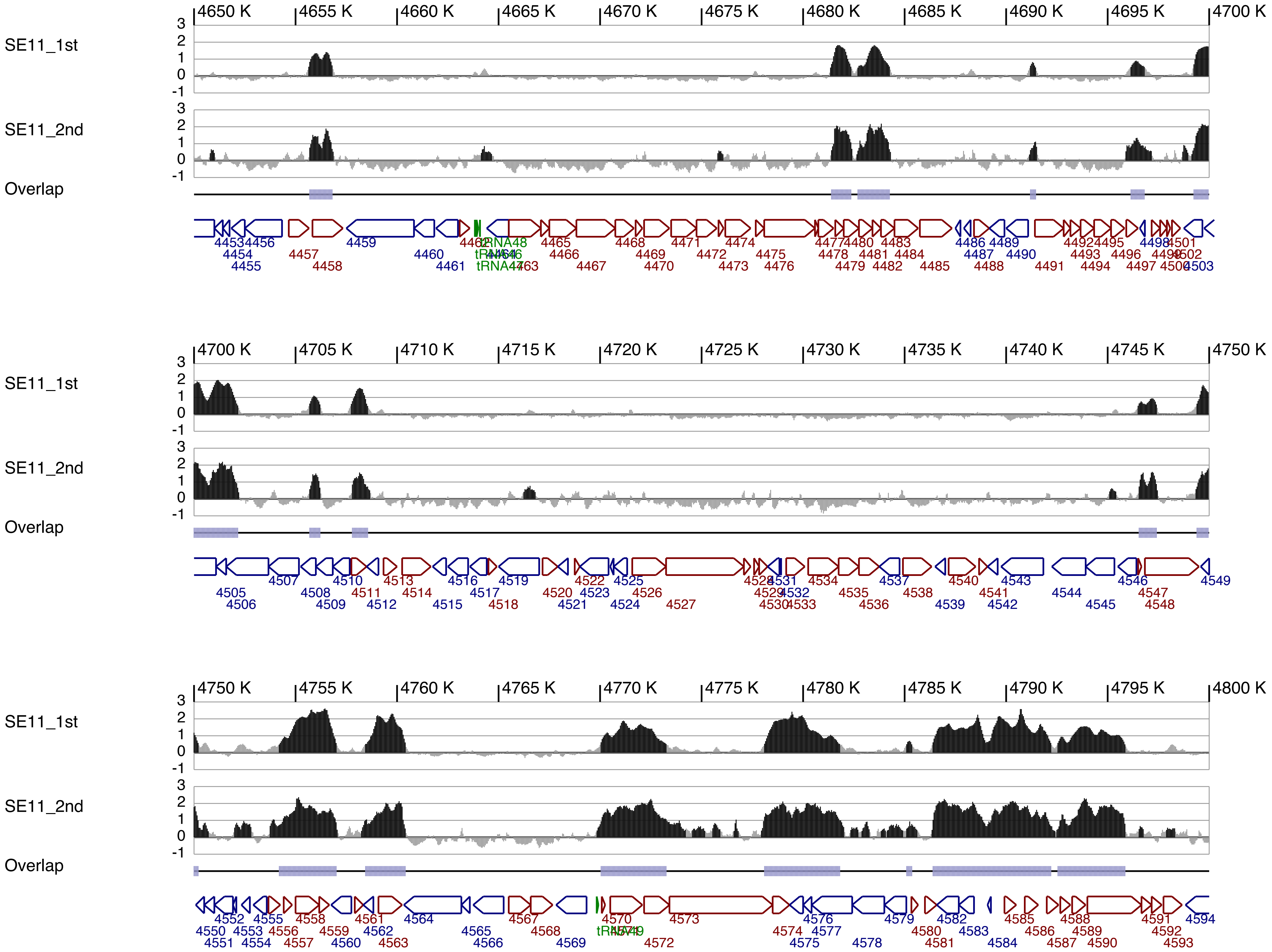

4650 K 4655 K 4660 K 4665 K 4670 K 4675 K 4680 K 4685 K 4690 K 4695 K 4700 K
SE11_1st
SE11_2nd
Overlap
44534456 4454 4455 4457 4458 4459 4460 4461 4462 tRNA48 tRNA46 tRNA47 4463 4465 4466 4467 4468 4469 4470 4471 4472 4473 4474 4475 4476 44774480 4483 4478 4481 4484 4479 4482 4485 4486 4489 4487 4490 4488 4491 44924495 4493 4496 4494 4498 4501 4497 4499 4502 4500 4503
4700 K 4705 K 4710 K 4715 K 4720 K 4725 K 4730 K 4735 K 4740 K 4745 K 4750 K
SE11_1st
SE11_2nd
Overlap
4505 4506 4507 4508 4509 4510 4511 4512 4513 4514 4515 4516 4517 4518 4519 4520 4521 4522 4523 4524 4525 4526 4527 4528 4529 4530 4531 4532 4533 4534 4535 4536 4537 4538 4539 4540 4541 4542 4543 4544 4545 4546 4547 4548 4549
4750 K 4755 K 4760 K 4765 K 4770 K 4775 K 4780 K 4785 K 4790 K 4795 K 4800 K
SE11_1st
SE11_2nd
Overlap
4550 4551 4552 4553 4554 4555 4556 4557 4558 4559 4560 4561 4562 4563 4564 4565 4566 4567 4568 4569 4570 tRNA49 4571 4572 4573 4574 4575 4576 4577 4578 4579 4580 4581 4582 4583 4584 4585 4586 4587 4588 4589 4590 4591 4592 4593 4594

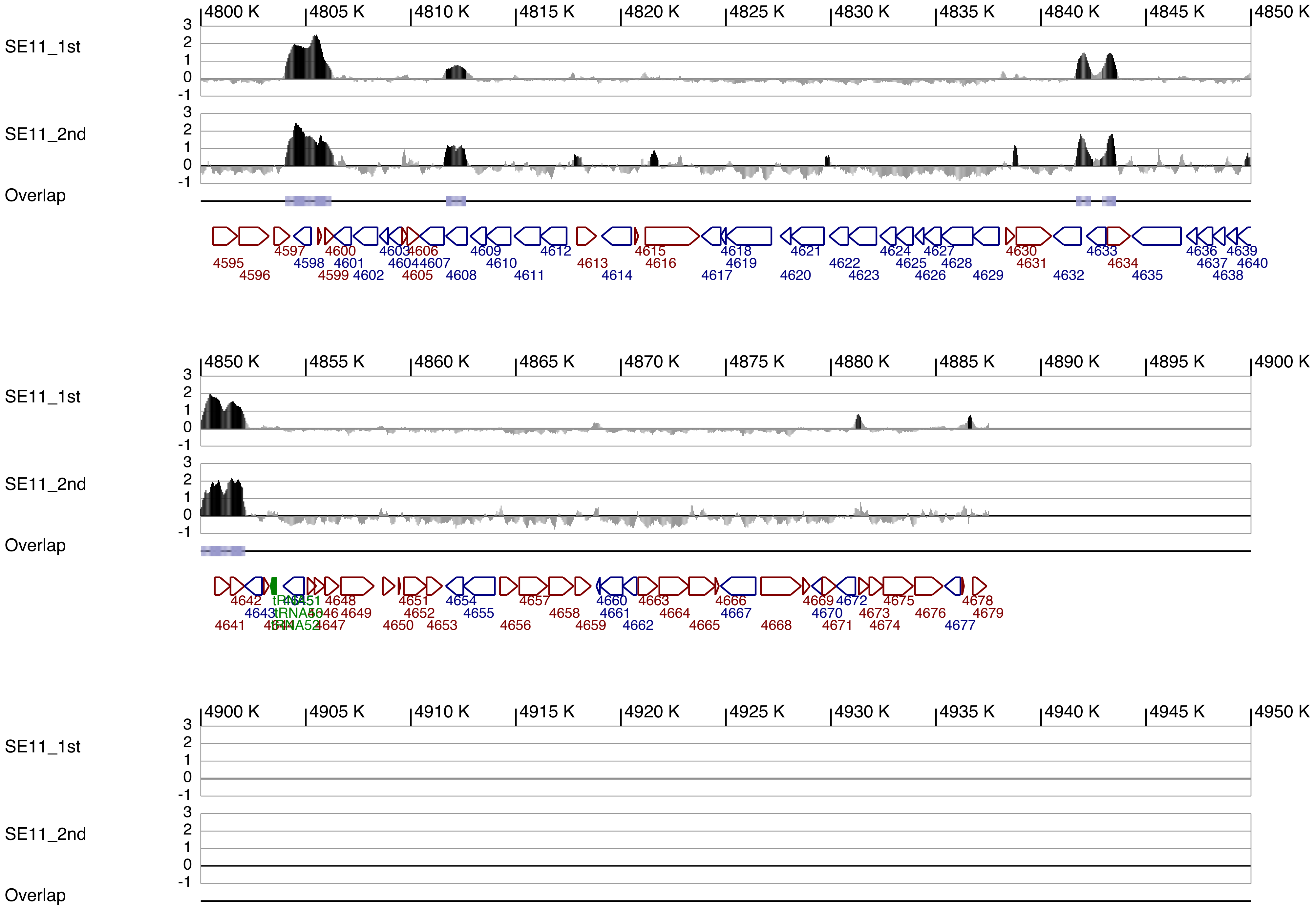
4800 K
4805 K
4810 K
4815 K
4820 K
4825 K
4830 K
4835 K
4840 K
4845 K
4850 K
SE11_1st
SE11_2nd
Overlap
3
2
1
0
-1
4595 4596 4597 4598 4599 4600 4601 4602 4603 4604 4605 4606 4607 4608 4609 4610 4611 4612 4613 4614 4615 4616 4617 4618 4619 4620 4621 4622 4623 4624 4625 4626 4627 4628 4629 4630 4631 4632 4633 4634 4635 4636 4637 4638 4639 4640
4850 K
4855 K
4860 K
4865 K
4870 K
4875 K
4880 K
4885 K
4890 K
4895 K
4900 K
SE11_1st
SE11_2nd
Overlap
4641 4642 4643 4647 4648 4649 4650 4651 4652 4653 4654 4655 4656 4657 4658 4659 4660 4661 4662 4663 4664 4665 4666 4667 4668 4669 4670 4671 4672 4673 4674 4675 4676 4677 4678 4679
4900 K
4905 K
4910 K
4915 K
4920 K
4925 K
4930 K
4935 K
4940 K
4945 K
4950 K
SE11_1st
SE11_2nd
Overlap

H-NS binding profiles in *Escherichia coli* SE15

(1st experiment and 2nd experiment)

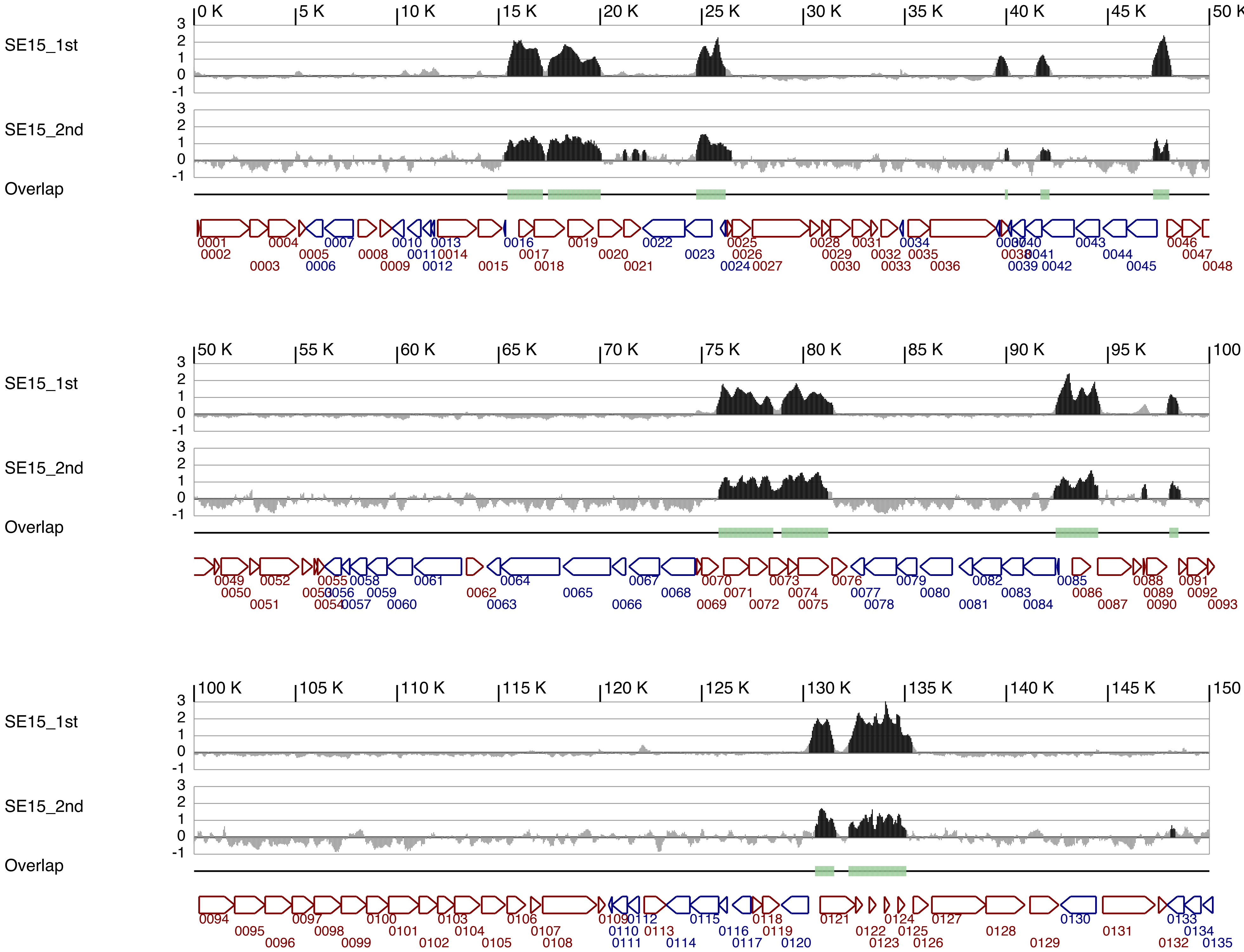

0 K
5 K
10 K
15 K
20 K
25 K
30 K
35 K
40 K
45 K
50 K
SE15_1st
SE15_2nd
Overlap
3
2
1
0
-1
0001 0002 0003 0004 0005 0006 0007 0008 0009 0010 0011 0012 0013 0014 0015 0016 0017 0018 0019 0020 0021 0022 0023 0024 0025 0026 0027 0028 0029 0030 0031 0032 0033 0034 0035 0036 0039 0041 0042 0043 0044 0045 0046 0047 0048
50 K
55 K
60 K
65 K
70 K
75 K
80 K
85 K
90 K
95 K
100 K
SE15_1st
SE15_2nd
Overlap
0049 0050 0051 0052 0054 0055 0056 0057 0058 0059 0060 0061 0062 0063 0064 0065 0066 0067 0068 0069 0070 0071 0072 0073 0074 0075 0076 0077 0078 0079 0080 0081 0082 0083 0084 0085 0086 0087 0088 0089 0090 0091 0092 0093
100 K
105 K
110 K
115 K
120 K
125 K
130 K
135 K
140 K
145 K
150 K
SE15_1st
SE15_2nd
Overlap
0094 0095 0096 0097 0098 0099 0100 0101 0102 0103 0104 0105 0106 0107 0108 0110 0111 0112 0113 0114 0115 0116 0117 0118 0119 0120 0121 0122 0123 0124 0125 0126 0127 0128 0129 0130 0131 0132 0133 0134 0135

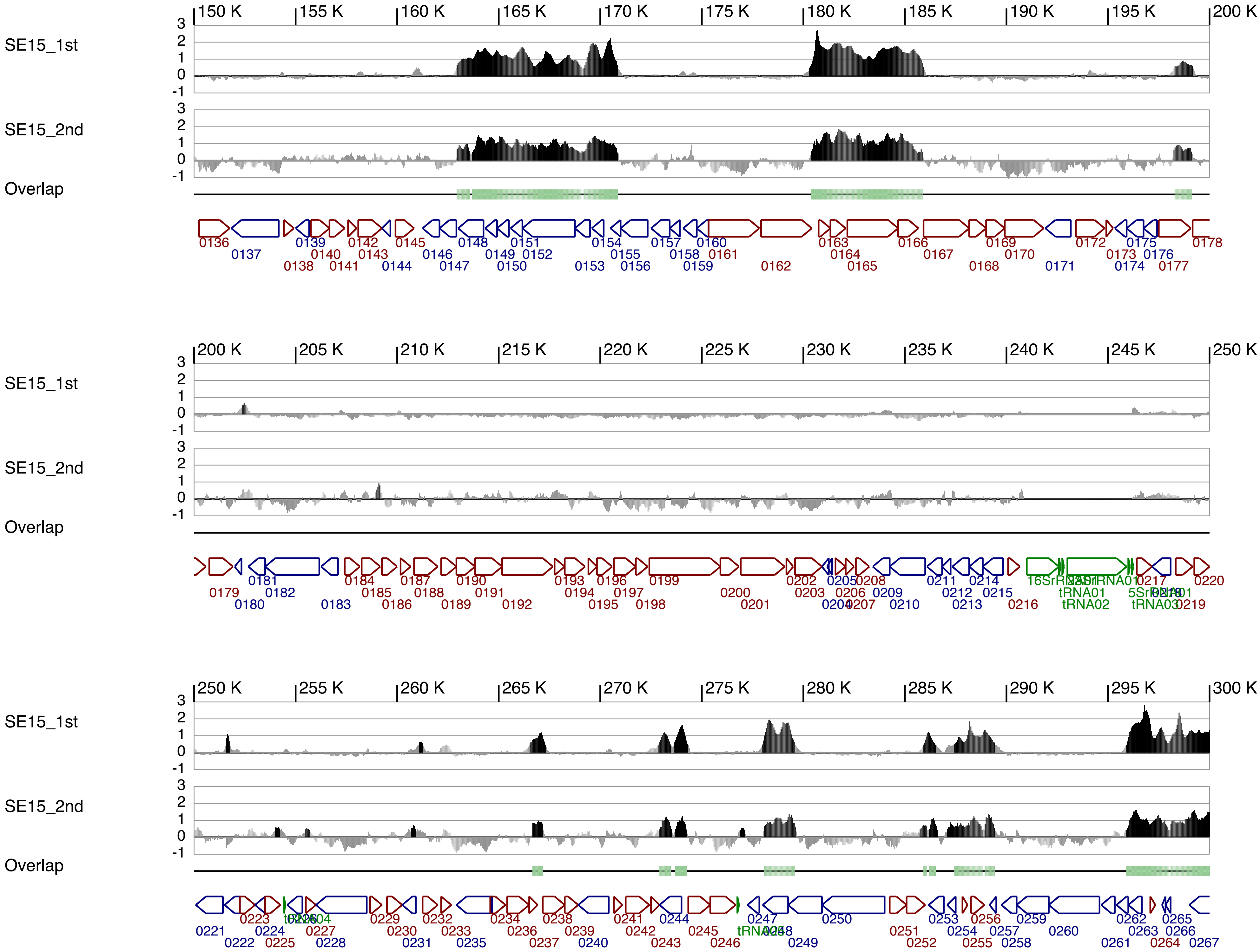

150 K
155 K
160 K
165 K
170 K
175 K
180 K
185 K
190 K
195 K
200 K
SE15_1st
SE15_2nd
Overlap
3
2
1
0
-1
0136 0137 0138 0139 0140 0141 0142 0143 0144 0145 0146 0147 0148 0149 0150 0151 0152 0153 0154 0155 0156 0157 0158 0159 0160 0161 0162 0163 0164 0165 0166 0167 0168 0169 0170 0171 0172 0173 0174 0175 0176 0177 0178
205 K
210 K
215 K
220 K
225 K
230 K
235 K
240 K
245 K
250 K
0179 0180 0181 0182 0183 0184 0185 0186 0187 0188 0189 0190 0191 0192 0193 0194 0195 0196 0197 0198 0199 0200 0201 0202 0203 0205 0206 0207 0208 0209 0210 0211 0212 0213 0214 0215 0216 0217 0219 0220
tRNA01
tRNA02
tRNA03
255 K
260 K
265 K
270 K
275 K
280 K
285 K
290 K
295 K
300 K
0221 0222 0223 0224 0225 0227 0228 0229 0230 0231 0232 0233 0235 0234 0236 0237 0238 0239 0240 0241 0242 0243 0244 0245 0246 0247 0249 0250 0251 0252 0253 0254 0255 0256 0257 0258 0259 0260 0261 0262 0263 0264 0265 0266 0267

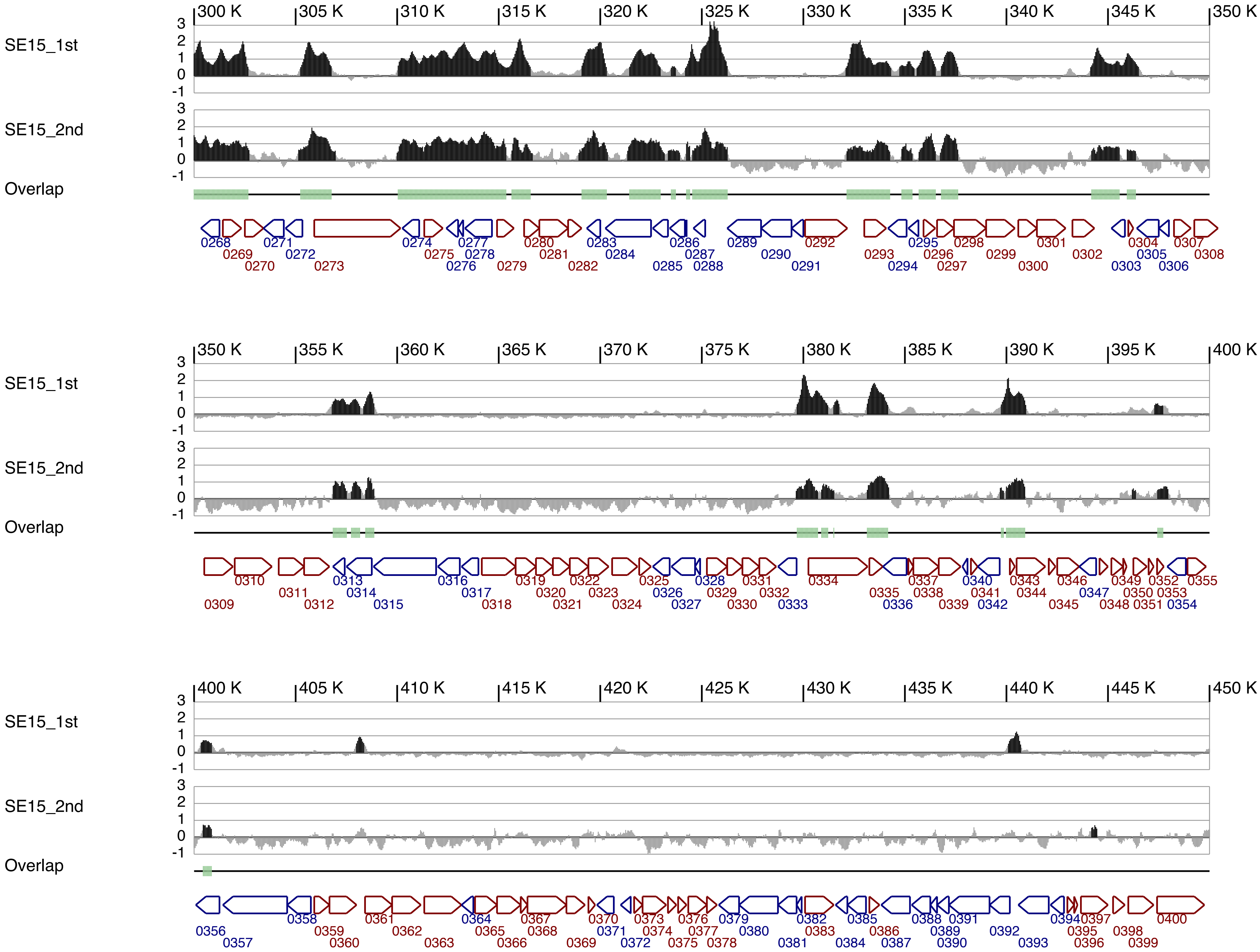
300 K 305 K 310 K 315 K 320 K 325 K 330 K 335 K 340 K 345 K 350 K
SE15_1st
SE15_2nd
Overlap
0268 0269 0270 0271 0272 0273 0274 0275 0276 0277 0278 0279 0280 0281 0282 0283 0284 0285 0286 0287 0288 0289 0290 0291 0292 0293 0294 0295 0296 0297 0298 0299 0300 0301 0302 0303 0304 0305 0306 0307 0308
350 K 355 K 360 K 365 K 370 K 375 K 380 K 385 K 390 K 395 K 400 K
SE15_1st
SE15_2nd
Overlap
0309 0310 0311 0312 0313 0314 0315 0316 0317 0318 0319 0320 0321 0322 0323 0324 0325 0326 0327 0328 0329 0330 0331 0332 0333 0334 0335 0336 0337 0338 0339 0340 0341 0342 0343 0344 0345 0346 0347 0348 0349 0350 0351 0352 0353 0354 0355
400 K 405 K 410 K 415 K 420 K 425 K 430 K 435 K 440 K 445 K 450 K
SE15_1st
SE15_2nd
Overlap
0356 0357 0358 0359 0360 0361 0362 0363 0364 0365 0366 0367 0368 0369 0370 0371 0372 0373 0374 0375 0376 0377 0378 0379 0380 0381 0382 0383 0384 0385 0386 0387 0388 0389 0390 0391 0392 0393 0394 0395 0396 0397 0398 0399 0400

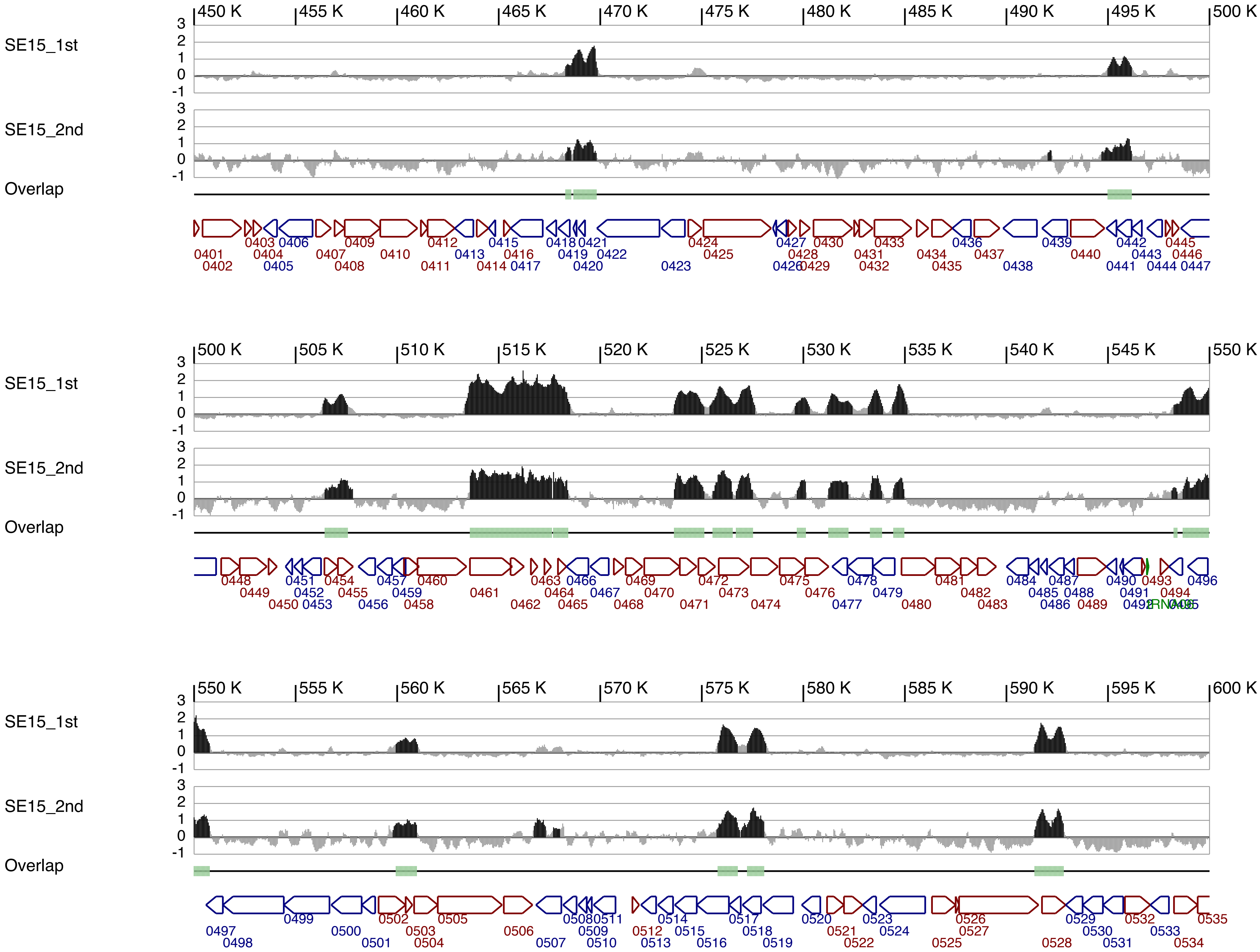
450 K
455 K
460 K
465 K
470 K
475 K
480 K
485 K
490 K
495 K
500 K
SE15_1st
SE15_2nd
Overlap
0401 0402 0403 0404 0405 0406 0407 0408 0409 0410 0411 0412 0413 0414 0415 0416 0417 0418 0419 0420 0421 0422 0423 0424 0425 0426 0427 0428 0429 0430 0431 0432 0433 0434 0435 0436 0437 0438 0439 0440 0441 0442 0443 0444 0445 0446 0447
505 K
510 K
515 K
520 K
525 K
530 K
535 K
540 K
545 K
550 K
0448 0449 0450 0451 0452 0453 0454 0455 0456 0457 0458 0459 0460 0461 0462 0463 0464 0465 0466 0467 0468 0469 0470 0471 0472 0473 0474 0475 0476 0477 0478 0479 0480 0481 0482 0483 0484 0485 0486 0487 0488 0489 0490 0491 0492 0493 0494 0495 0496 tRNA05
555 K
560 K
565 K
570 K
575 K
580 K
585 K
590 K
595 K
600 K
0497 0498 0499 0500 0501 0502 0503 0504 0505 0506 0507 0508 0509 0510 0511 0512 0513 0514 0515 0516 0517 0518 0519 0520 0521 0522 0523 0524 0525 0526 0527 0528 0529 0530 0531 0532 0533 0534 0535

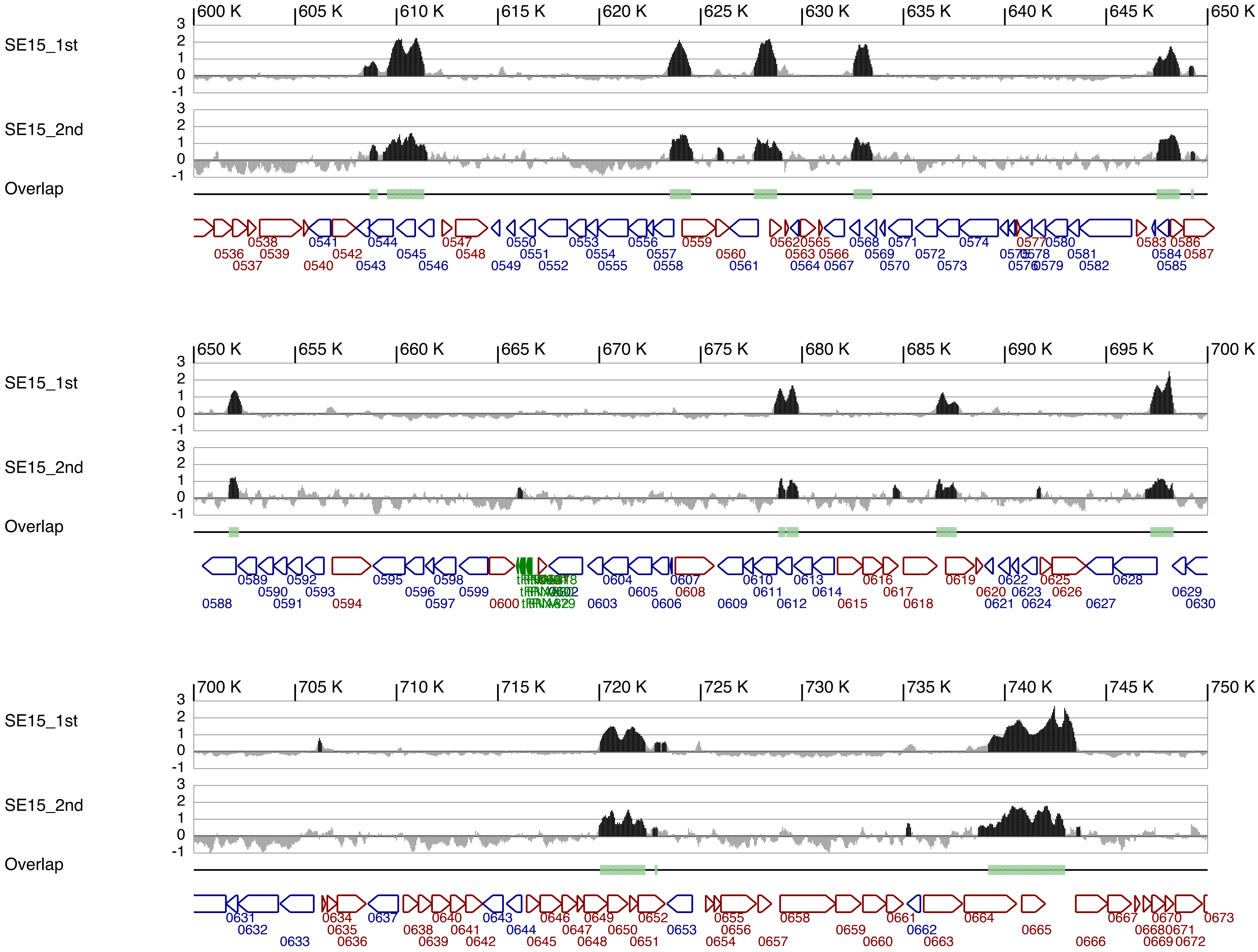
600 K 605 K 610 K 615 K 620 K 625 K 630 K 635 K 640 K 645 K 650 K
SE15_1st
SE15_2nd
Overlap
0538 0541 0544 0547 0550 0553 0556 0559 0562 0565 0568 0571 0574 0577 0580 0583 0586
0536 0539 0542 0545 0548 0551 0554 0557 0560 0563 0566 0569 0572 0578 0581 0584 0587
0537 0540 0543 0546 0549 0552 0555 0558 0561 0564 0567 0570 0573 0579 0582 0585
650 K 655 K 660 K 665 K 670 K 675 K 680 K 685 K 690 K 695 K 700 K
SE15_1st
SE15_2nd
Overlap
0589 0592 0595 0598 0604 0607 0610 0613 0616 0619 0622 0625 0628
0590 0593 0596 0599 0605 0608 0611 0614 0617 0620 0623 0626 0629
0588 0591 0594 0597 0600 0603 0606 0609 0612 0615 0618 0621 0624 0627 0630
700 K 705 K 710 K 715 K 720 K 725 K 730 K 735 K 740 K 745 K 750 K
SE15_1st
SE15_2nd
Overlap
0631 0634 0637 0640 0643 0646 0649 0652 0655 0658 0661 0664 0667 0670 0673
0632 0635 0638 0641 0644 0647 0650 0653 0656 0659 0662 0665 0668 0671
0633 0636 0639 0642 0645 0648 0651 0654 0657 0660 0663 0666 0669 0672

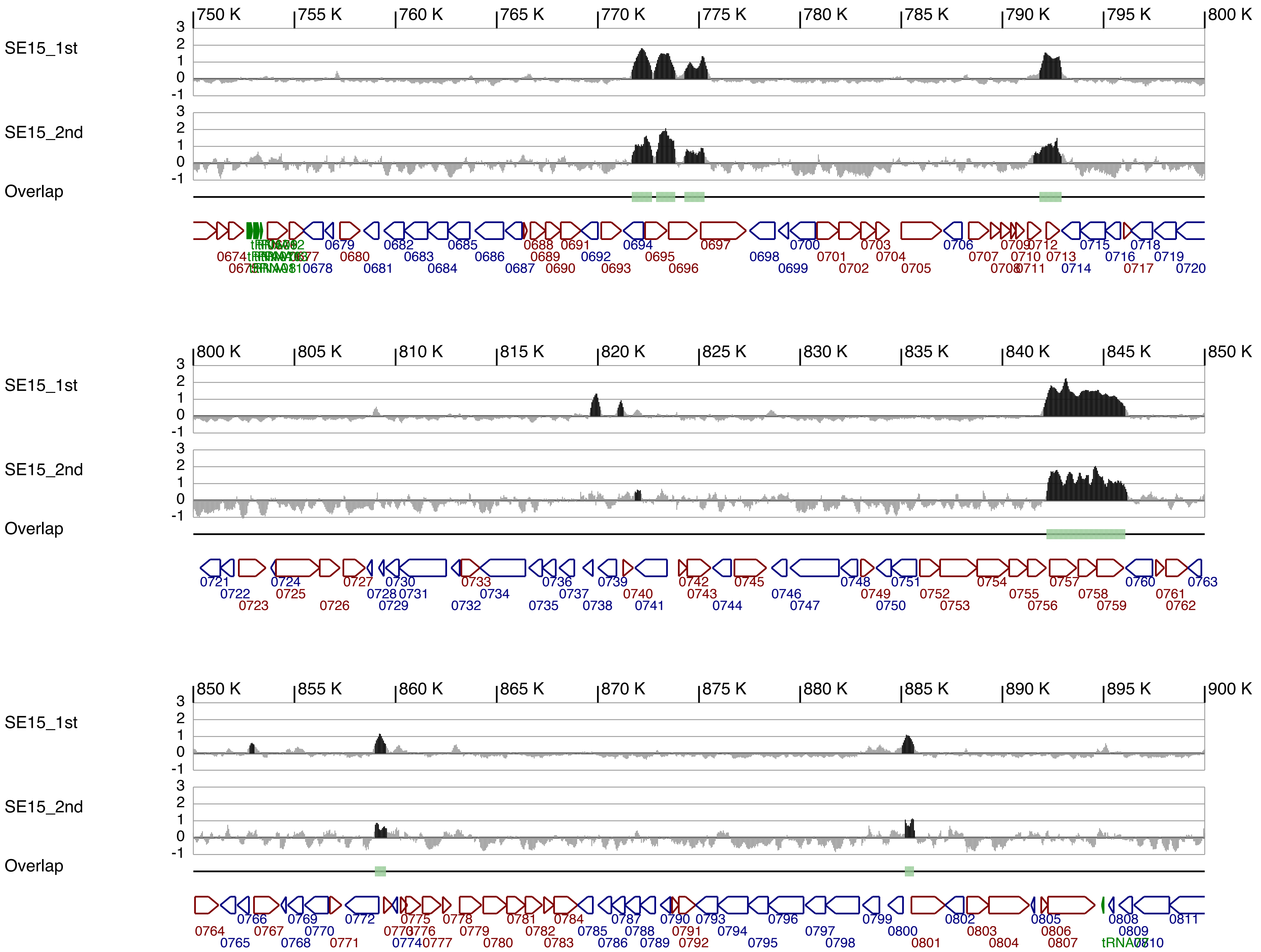

750 K
755 K
760 K
765 K
770 K
775 K
780 K
785 K
790 K
795 K
800 K
SE15_1st
SE15_2nd
Overlap
3
2
1
0
-1
0679 0682 0685 0688 0691 0694 0697 0700 0703 0706 0712 0715 0718
0674 0677 0680 0683 0686 0689 0692 0695 0698 0701 0704 0707 0710 0713 0716 0719
0678 0681 0684 0687 0690 0693 0696 0699 0702 0705 0711 0714 0717 0720
805 K
810 K
815 K
820 K
825 K
830 K
835 K
840 K
845 K
850 K
0721 0724 0727 0730 0733 0736 0739 0742 0745 0748 0751 0754 0757 0760 0763
0722 0725 0728 0731 0734 0737 0740 0743 0746 0749 0752 0755 0758 0761
0723 0726 0729 0732 0735 0738 0741 0744 0747 0750 0753 0756 0759 0762
855 K
860 K
865 K
870 K
875 K
880 K
885 K
890 K
895 K
900 K
0766 0769 0772 0775 0778 0781 0784 0787 0790 0793 0796 0799 0802 0805 0808 0811
0764 0767 0770 0776 0779 0782 0785 0788 0791 0794 0797 0800 0803 0806 0809
0765 0768 0771 0777 0780 0783 0786 0789 0792 0795 0798 0801 0804 0807 0810

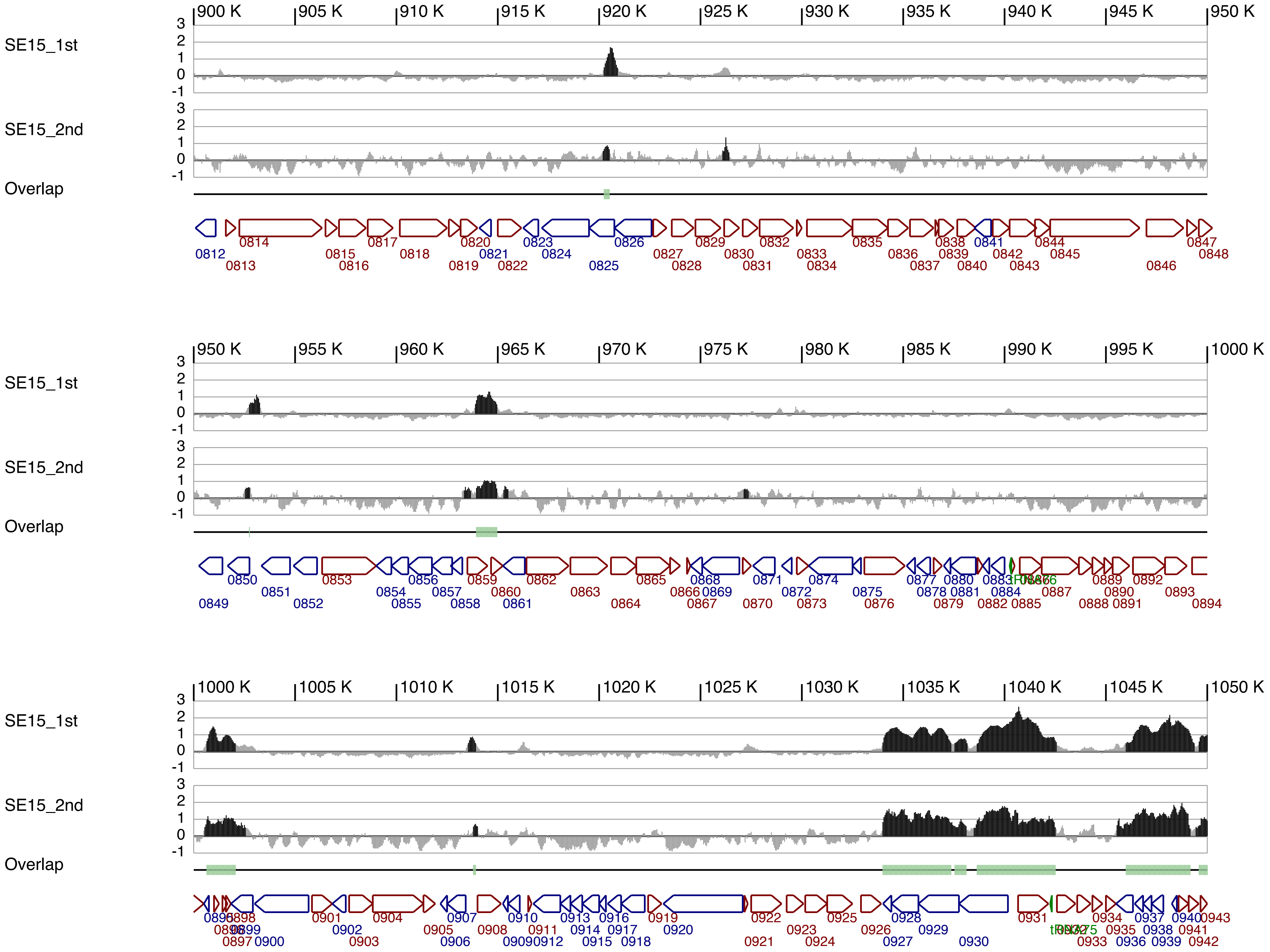

900 K
905 K
910 K
915 K
920 K
925 K
930 K
935 K
940 K
945 K
950 K
SE15_1st
SE15_2nd
Overlap
3
2
1
0
-1
0812 0813 0814 0815 0816 0817 0818 0819 0820 0821 0822 0823 0824 0825 0826 0827 0828 0829 0830 0831 0832 0833 0834 0835 0836 0837 0838 0839 0840 0841 0842 0843 0844 0845 0846 0847 0848
950 K
955 K
960 K
965 K
970 K
975 K
980 K
985 K
990 K
995 K
1000 K
SE15_1st
SE15_2nd
Overlap
0849 0850 0851 0852 0853 0854 0855 0856 0857 0858 0859 0860 0861 0862 0863 0864 0865 0866 0867 0868 0869 0870 0871 0872 0873 0874 0875 0876 0877 0878 0879 0880 0881 0882 0884 0885 0887 0888 0889 0890 0891 0892 0893 0894
1000 K
1005 K
1010 K
1015 K
1020 K
1025 K
1030 K
1035 K
1040 K
1045 K
1050 K
SE15_1st
SE15_2nd
Overlap
0897 0898 0899 0900 0901 0902 0903 0904 0905 0906 0907 0908 0910 0911 0913 0914 0915 0916 0917 0918 0919 0920 0921 0922 0923 0924 0925 0926 0927 0928 0929 0930 0931 0933 0934 0935 0936 0937 0938 0939 0940 0941 0942 0943

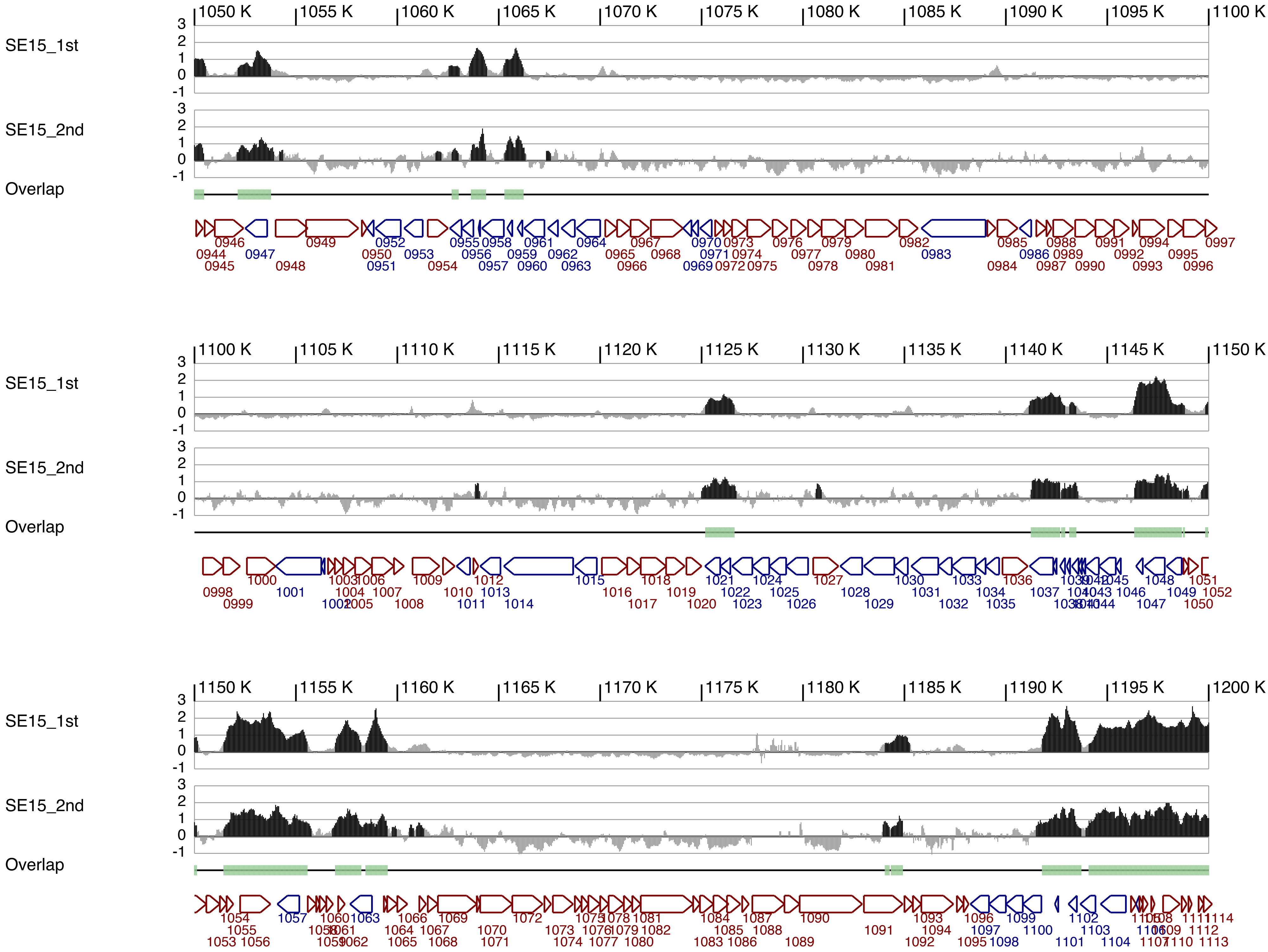
1050 K
1055 K
1060 K
1065 K
1070 K
1075 K
1080 K
1085 K
1090 K
1095 K
1100 K
SE15_1st
SE15_2nd
Overlap
3
2
1
0
-1
0946 0947 0949 0952 0955 0958 0961 0964 0967 0970 0973 0976 0979 0982 0985 0988 0991 0994 0997
0944 0950 0953 0956 0959 0962 0965 0968 0971 0974 0977 0980 0983 0986 0989 0992 0995
0945 0948 0951 0954 0957 0960 0963 0966 0969 0972 0975 0978 0981 0984 0987 0990 0993 0996
1100 K
1105 K
1110 K
1115 K
1120 K
1125 K
1130 K
1135 K
1140 K
1145 K
1150 K
SE15_1st
SE15_2nd
Overlap
1000 1003 1006 1009 1012 1015 1018 1021 1024 1027 1030 1033 1036 1045 1048 1051
0998 1001 1004 1007 1010 1013 1016 1019 1022 1025 1028 1031 1034 1037 1046 1049 1052
0999 1005 1008 1011 1014 1017 1020 1023 1026 1029 1032 1035 1047 1050
1150 K
1155 K
1160 K
1165 K
1170 K
1175 K
1180 K
1185 K
1190 K
1195 K
1200 K
SE15_1st
SE15_2nd
Overlap
1054 1057 1060 1063 1066 1069 1072 1075 1078 1081 1084 1087 1090 1093 1096 1099 1102 1108 1114
1055 1061 1064 1067 1070 1073 1076 1079 1082 1085 1088 1091 1094 1097 1100 1103 1109 1112
1053 1056 1062 1065 1068 1071 1074 1077 1080 1083 1086 1089 1092 1095 1098 1101 1104 1110 1113

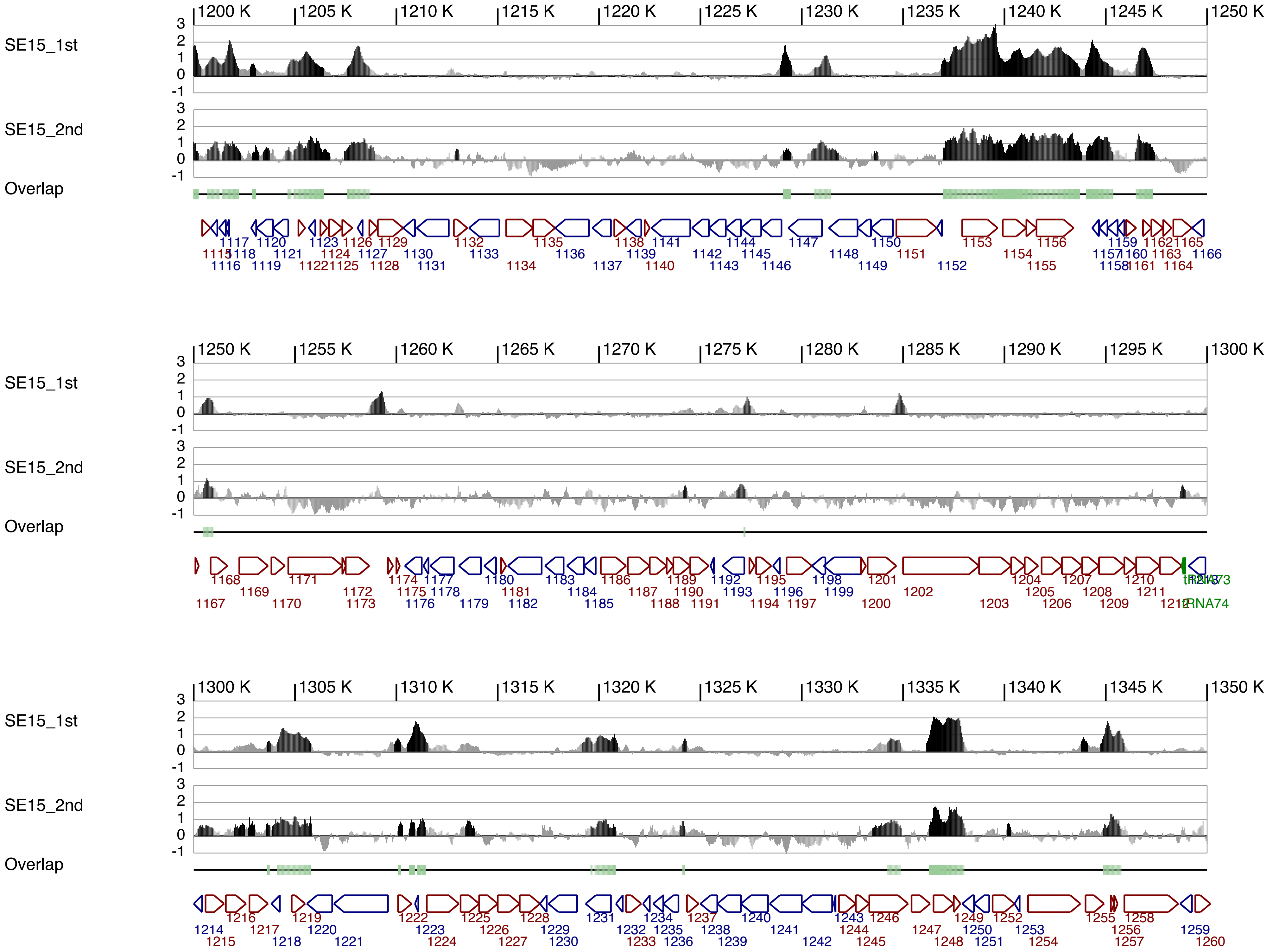

1200 K
1205 K
1210 K
1215 K
1220 K
1225 K
1230 K
1235 K
1240 K
1245 K
1250 K
SE15_1st
SE15_2nd
Overlap
1255 K
1260 K
1265 K
1270 K
1275 K
1280 K
1285 K
1290 K
1295 K
1300 K
1305 K
1310 K
1315 K
1320 K
1325 K
1330 K
1335 K
1340 K
1345 K
1350 K
tRNA73
tRNA74

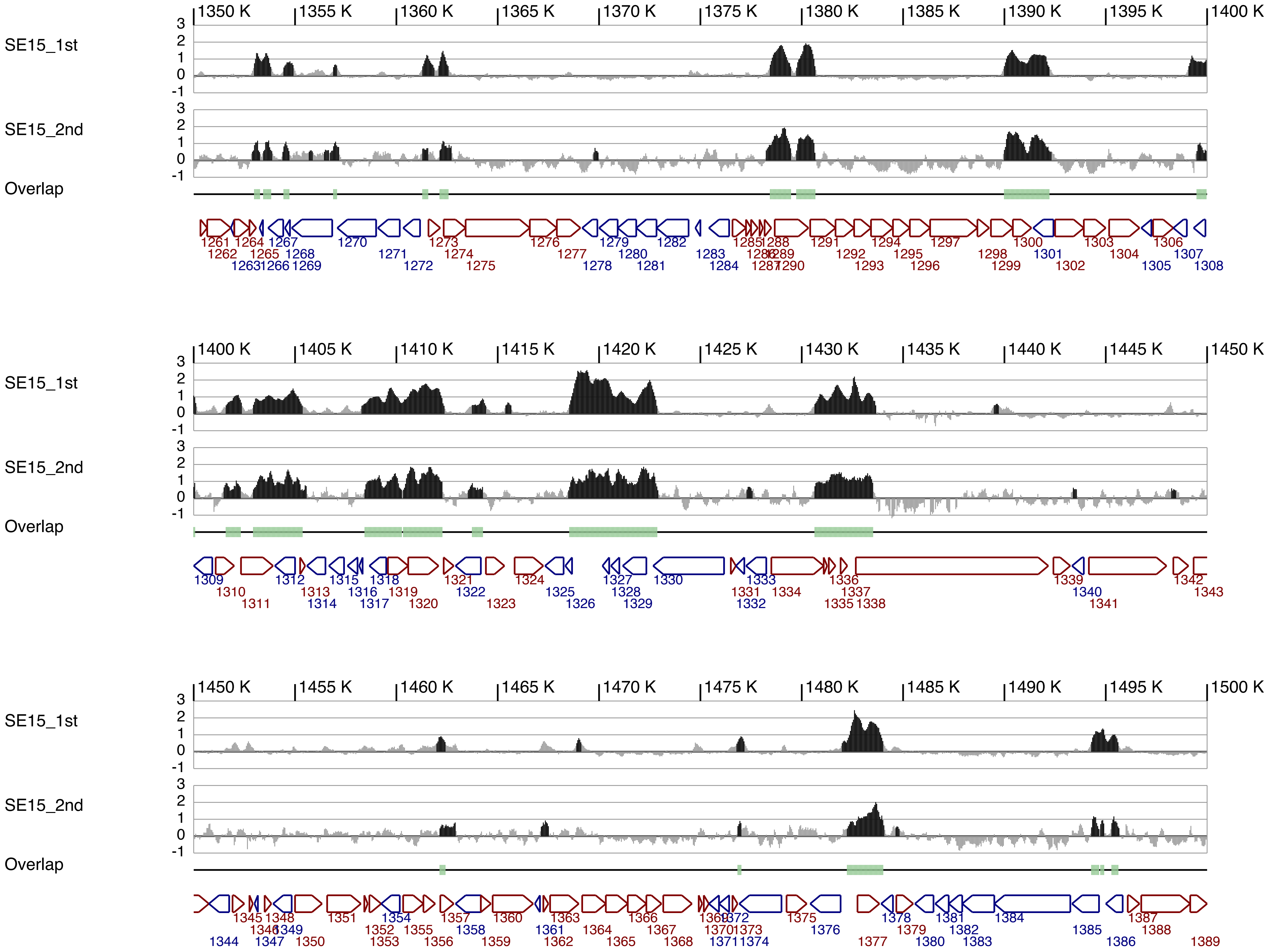
1350 K
1355 K
1360 K
1365 K
1370 K
1375 K
1380 K
1385 K
1390 K
1395 K
1400 K
SE15_1st
SE15_2nd
Overlap
3
2
1
0
-1
1261 1264 1267 1270 1273 1276 1279 1282 1285 1288 1291 1294 1297 1300 1303 1306
1262 1265 1268 1271 1274 1277 1280 1283 1286 1289 1292 1295 1298 1301 1304 1307
1263 1266 1269 1272 1275 1278 1281 1284 1287 1290 1293 1296 1299 1302 1305 1308
1405 K
1410 K
1415 K
1420 K
1425 K
1430 K
1435 K
1440 K
1445 K
1450 K
1309 1312 1315 1318 1321 1324 1327 1330 1333 1336 1339 1342
1310 1313 1316 1319 1322 1325 1328 1331 1334 1337 1340 1343
1311 1314 1317 1320 1323 1326 1329 1332 1335 1338 1341
1455 K
1460 K
1465 K
1470 K
1475 K
1480 K
1485 K
1490 K
1495 K
1500 K
1345 1348 1351 1354 1357 1360 1363 1366 1369 1372 1375 1378 1381 1384 1387
1346 1349 1352 1355 1358 1361 1364 1367 1370 1373 1376 1379 1382 1385 1388
1344 1347 1350 1353 1356 1359 1362 1365 1368 1371 1374 1377 1380 1383 1386 1389

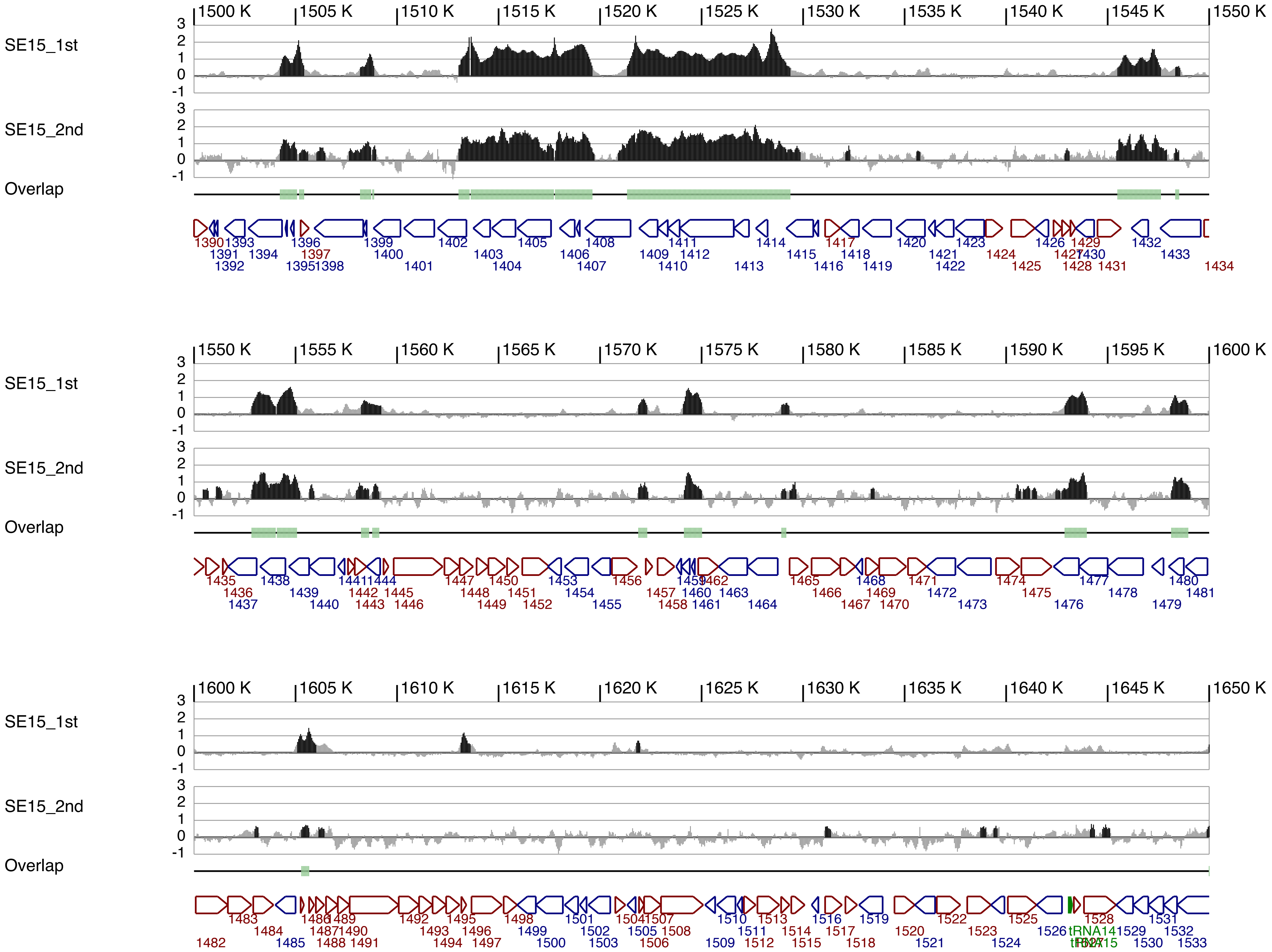
1500 K
1505 K
1510 K
1515 K
1520 K
1525 K
1530 K
1535 K
1540 K
1545 K
1550 K
SE15_1st
SE15_2nd
Overlap
1550 K
1555 K
1560 K
1565 K
1570 K
1575 K
1580 K
1585 K
1590 K
1595 K
1600 K
SE15_1st
SE15_2nd
Overlap
1600 K
1605 K
1610 K
1615 K
1620 K
1625 K
1630 K
1635 K
1640 K
1645 K
1650 K
SE15_1st
SE15_2nd
Overlap

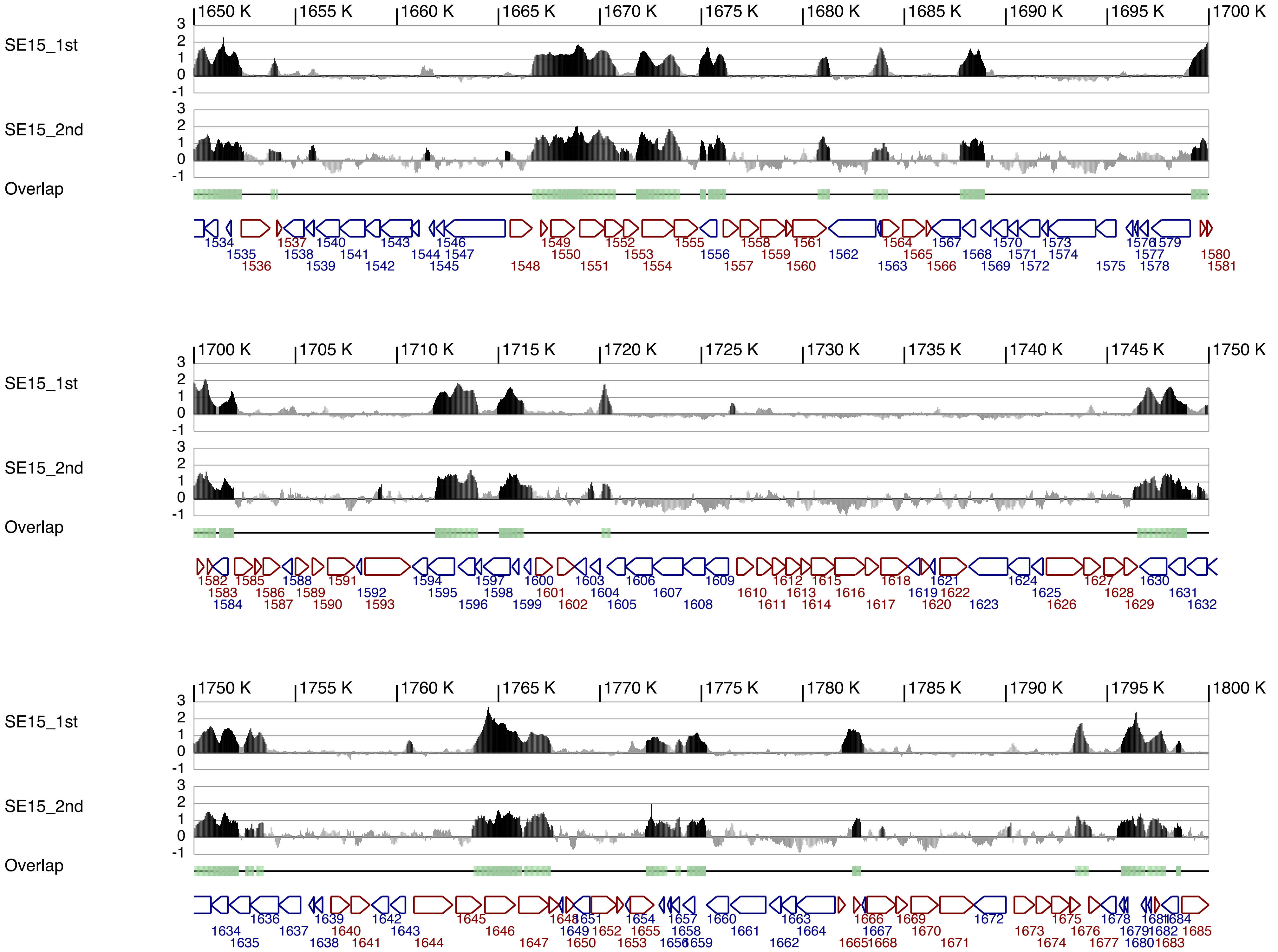

1650 K 1655 K 1660 K 1665 K 1670 K 1675 K 1680 K 1685 K 1690 K 1695 K 1700 K
SE15_1st
SE15_2nd
Overlap
3 2 1 0 -1
1534 1537 1540 1543 1546 1549 1552 1555 1558 1561 1564 1567 1570 1573 1576 1579
1535 1538 1541 1544 1547 1550 1553 1556 1559 1562 1565 1568 1571 1574 1577 1580
1536 1539 1542 1545 1548 1551 1554 1557 1560 1563 1566 1569 1572 1575 1578 1581
1700 K 1705 K 1710 K 1715 K 1720 K 1725 K 1730 K 1735 K 1740 K 1745 K 1750 K
SE15_1st
SE15_2nd
Overlap
1582 1585 1588 1591 1594 1597 1600 1603 1606 1609 1612 1615 1618 1621 1624 1627 1630
1583 1586 1589 1592 1595 1598 1601 1604 1607 1610 1613 1616 1619 1622 1625 1628 1631
1584 1587 1590 1593 1596 1599 1602 1605 1608 1611 1614 1617 1620 1623 1626 1629 1632
1750 K 1755 K 1760 K 1765 K 1770 K 1775 K 1780 K 1785 K 1790 K 1795 K 1800 K
SE15_1st
SE15_2nd
Overlap
1636 1639 1642 1645 1648 1651 1654 1657 1660 1663 1666 1669 1672 1675 1678 1681 1684
1634 1637 1640 1643 1646 1649 1652 1655 1658 1661 1664 1667 1670 1673 1676 1679 1682 1685
1635 1638 1641 1644 1647 1650 1653 1656 1659 1662 1665 1668 1671 1674 1677 1680 1683

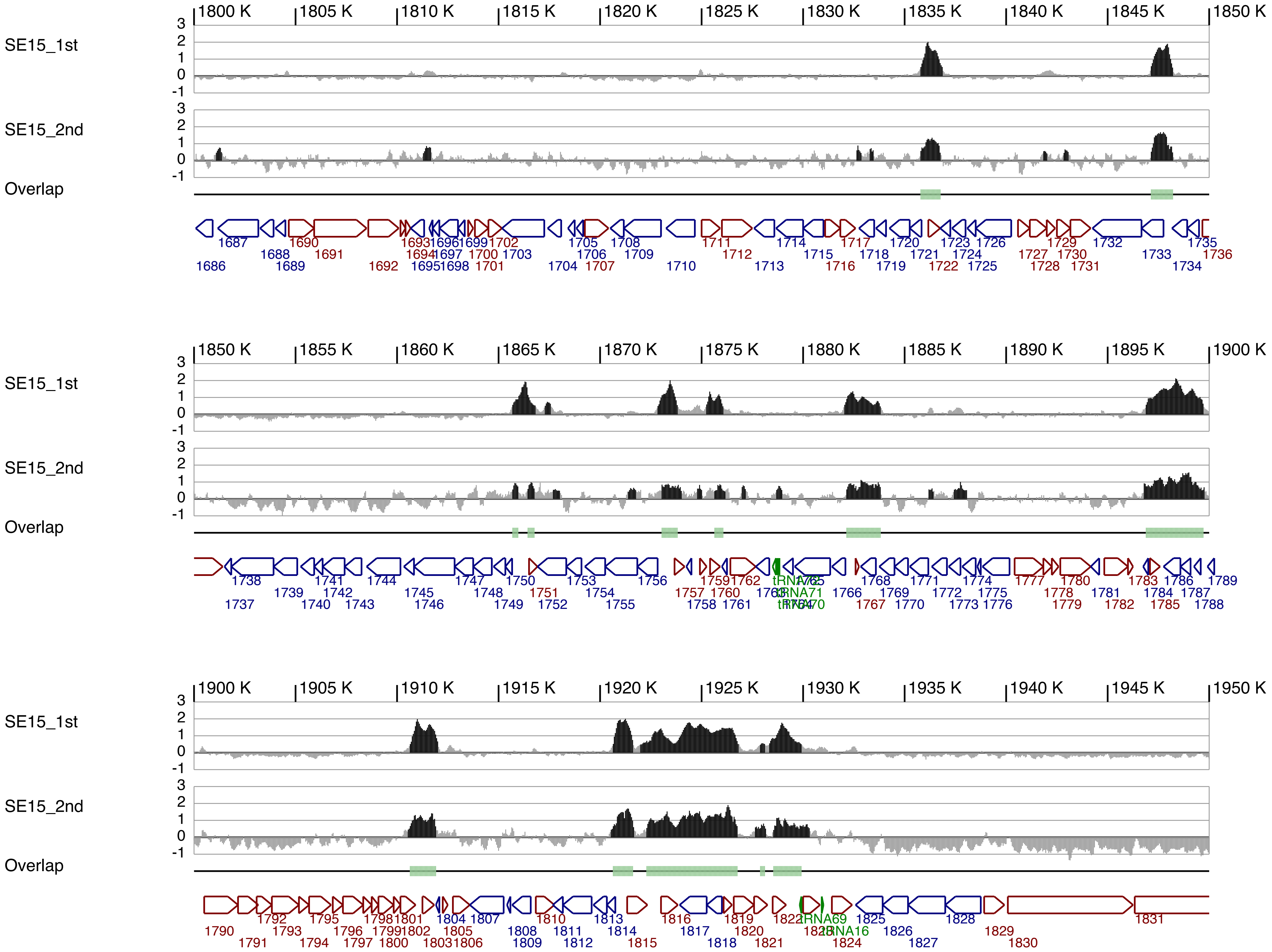

1800 K
1805 K
1810 K
1815 K
1820 K
1825 K
1830 K
1835 K
1840 K
1845 K
1850 K
SE15_1st
SE15_2nd
Overlap
3
2
1
0
-1
1687 1690 1702 1705 1708 1711 1714 1717 1720 1723 1726 1729 1732 1735
1688 1691 1700 1703 1706 1709 1712 1715 1718 1721 1724 1727 1730 1733 1736
1686 1689 1692 1701 1704 1707 1710 1713 1716 1719 1722 1725 1728 1731 1734
1855 K
1860 K
1865 K
1870 K
1875 K
1880 K
1885 K
1890 K
1895 K
1900 K
1738 1741 1744 1747 1750 1753 1756 1768 1771 1774 1777 1780 1783 1786 1789
1739 1742 1745 1748 1751 1754 1757 1760 1766 1769 1772 1775 1778 1781 1784 1787
1737 1740 1743 1746 1749 1752 1755 1758 1761 1767 1770 1773 1776 1779 1782 1785 1788
1905 K
1910 K
1915 K
1920 K
1925 K
1930 K
1935 K
1940 K
1945 K
1950 K
1792 1795 1804 1807 1810 1813 1816 1819 1822 tRNA69 1825 1828 1831
1790 1793 1796 1805 1808 1811 1814 1817 1820 tRNA16 1826 1829
1791 1794 1797 1800 1809 1812 1815 1818 1821 1824 1827 1830

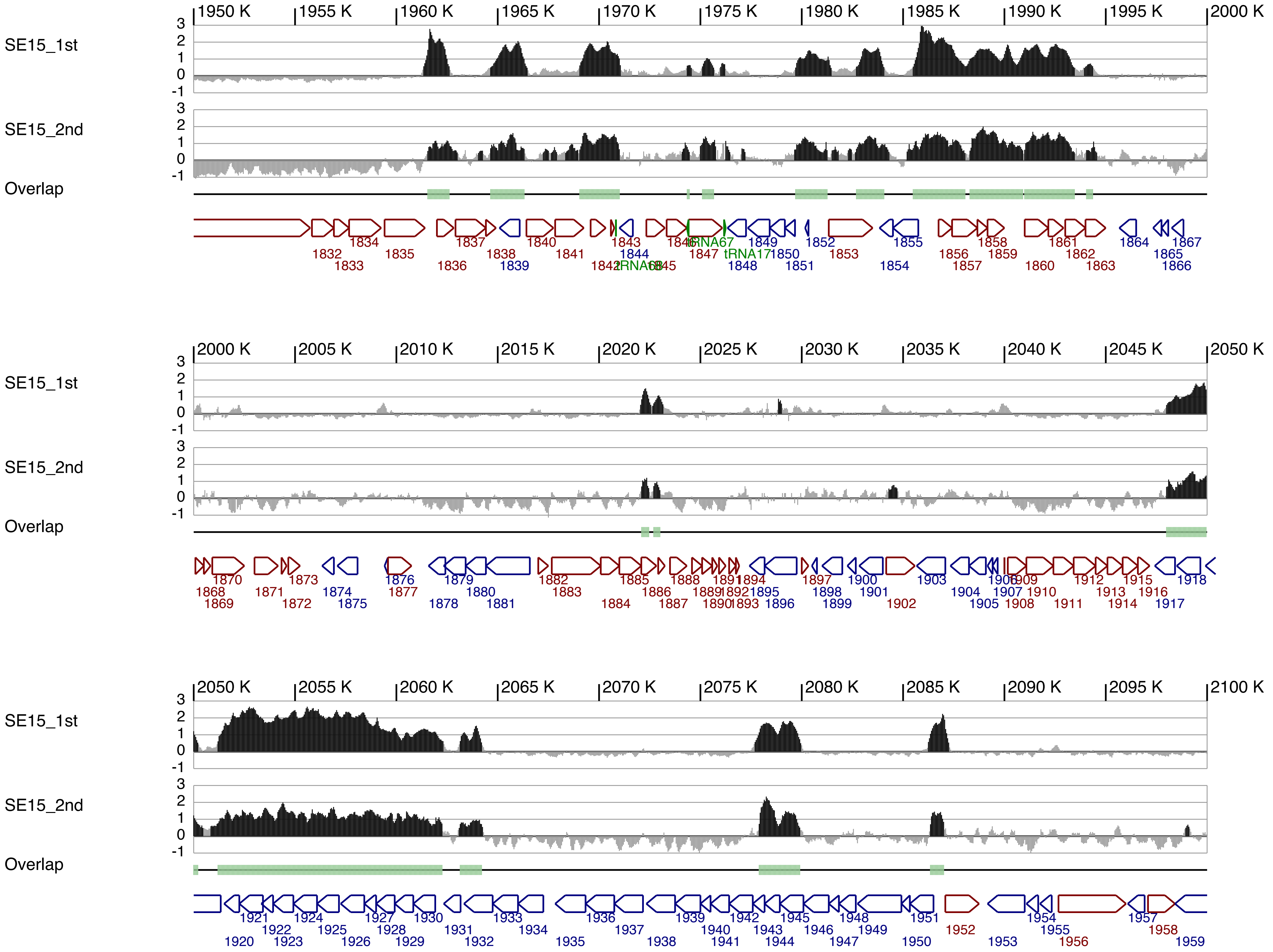
1950 K
1955 K
1960 K
1965 K
1970 K
1975 K
1980 K
1985 K
1990 K
1995 K
2000 K
SE15_1st
SE15_2nd
Overlap
3
2
1
0
-1
1832
1833
1834
1835
1836
1837
1838
1839
1840
1841
1842
1843
1844
1845
1847
1848
1849
1850
1851
1852
1853
1854
1855
1856
1857
1858
1859
1860
1861
1862
1863
1864
1865
1866
1867
tRNA67
tRNA17
2000 K
2005 K
2010 K
2015 K
2020 K
2025 K
2030 K
2035 K
2040 K
2045 K
2050 K
SE15_1st
SE15_2nd
Overlap
1868
1869
1870
1871
1872
1873
1874
1875
1876
1877
1878
1879
1880
1881
1882
1883
1884
1885
1886
1887
1888
1889
1890
1893
1895
1896
1897
1898
1899
1900
1901
1902
1903
1904
1905
1907
1908
1910
1911
1912
1913
1914
1915
1916
1917
1918
2050 K
2055 K
2060 K
2065 K
2070 K
2075 K
2080 K
2085 K
2090 K
2095 K
2100 K
SE15_1st
SE15_2nd
Overlap
1920
1921
1922
1923
1924
1925
1926
1927
1928
1929
1930
1931
1932
1933
1934
1935
1936
1937
1938
1939
1940
1941
1942
1943
1944
1945
1946
1947
1948
1949
1950
1951
1952
1953
1954
1955
1956
1957
1958
1959

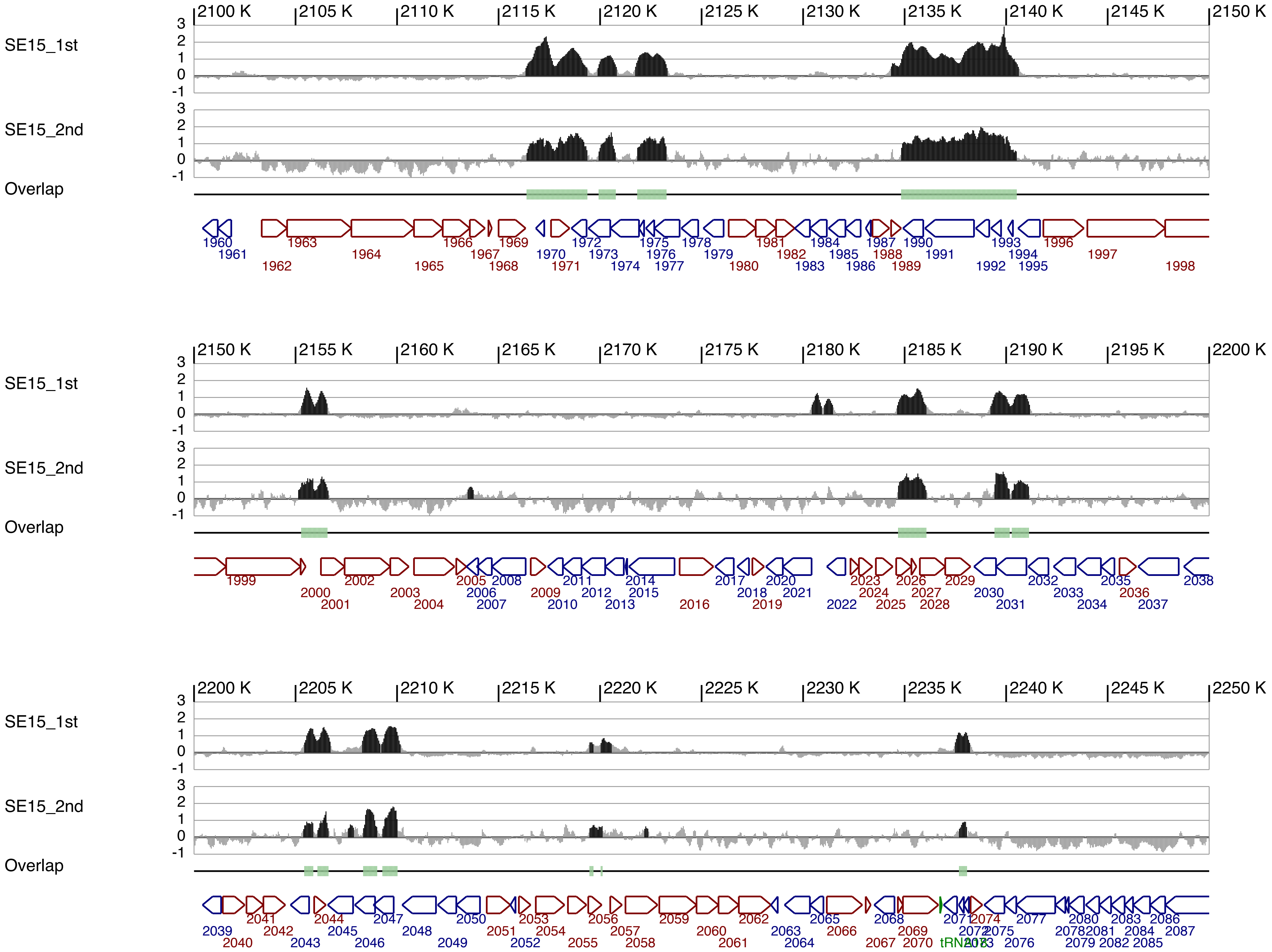

2100 K 2105 K 2110 K 2115 K 2120 K 2125 K 2130 K 2135 K 2140 K 2145 K 2150 K
SE15_1st
SE15_2nd
Overlap
3 2 1 0 -1
1960 1963 1966 1969 1972 1975 1978 1981 1984 1987 1990 1993 1996
1961 1964 1967 1970 1973 1976 1979 1982 1985 1988 1991 1994 1997
1962 1965 1968 1971 1974 1977 1980 1983 1986 1989 1992 1995 1998
2150 K 2155 K 2160 K 2165 K 2170 K 2175 K 2180 K 2185 K 2190 K 2195 K 2200 K
SE15_1st
SE15_2nd
Overlap
1999 2002 2005 2008 2011 2014 2017 2020 2023 2026 2029 2032 2035 2038
2000 2003 2006 2009 2012 2015 2018 2021 2024 2027 2030 2033 2036
2001 2004 2007 2010 2013 2016 2019 2022 2025 2028 2031 2034 2037
2200 K 2205 K 2210 K 2215 K 2220 K 2225 K 2230 K 2235 K 2240 K 2245 K 2250 K
SE15_1st
SE15_2nd
Overlap
2041 2044 2047 2050 2053 2056 2059 2062 2065 2068 2071 2074 2077 2080 2083 2086
2039 2042 2045 2048 2051 2054 2057 2060 2063 2066 2069 2072 2075 2078 2081 2084 2087
2040 2043 2046 2049 2052 2055 2058 2061 2064 2067 2070 tRNA 2073 2076 2079 2082 2085

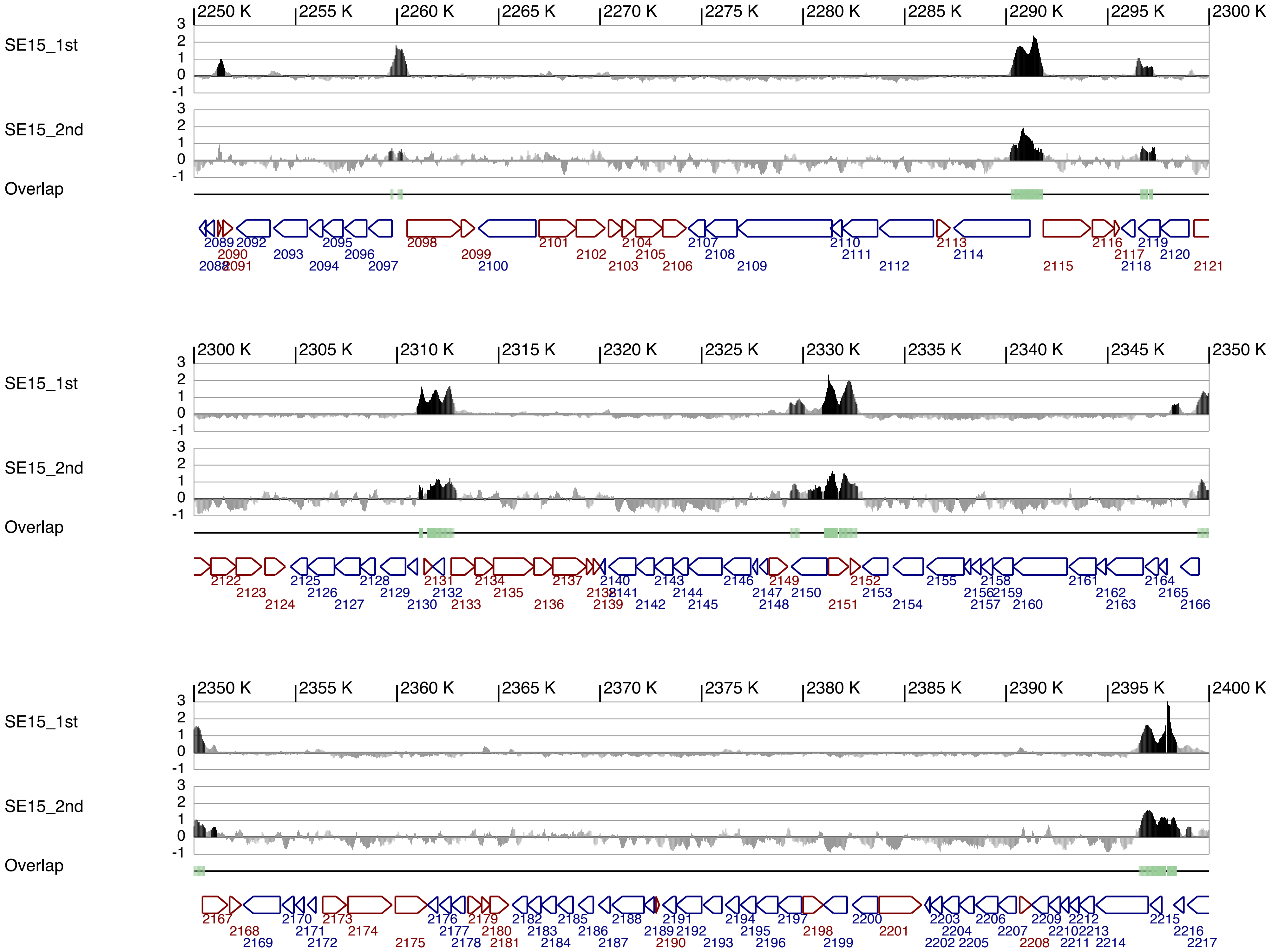

2250 K
2255 K
2260 K
2265 K
2270 K
2275 K
2280 K
2285 K
2290 K
2295 K
2300 K
SE15_1st
SE15_2nd
Overlap
2300 K
2305 K
2310 K
2315 K
2320 K
2325 K
2330 K
2335 K
2340 K
2345 K
2350 K
SE15_1st
SE15_2nd
Overlap
2350 K
2355 K
2360 K
2365 K
2370 K
2375 K
2380 K
2385 K
2390 K
2395 K
2400 K
SE15_1st
SE15_2nd
Overlap

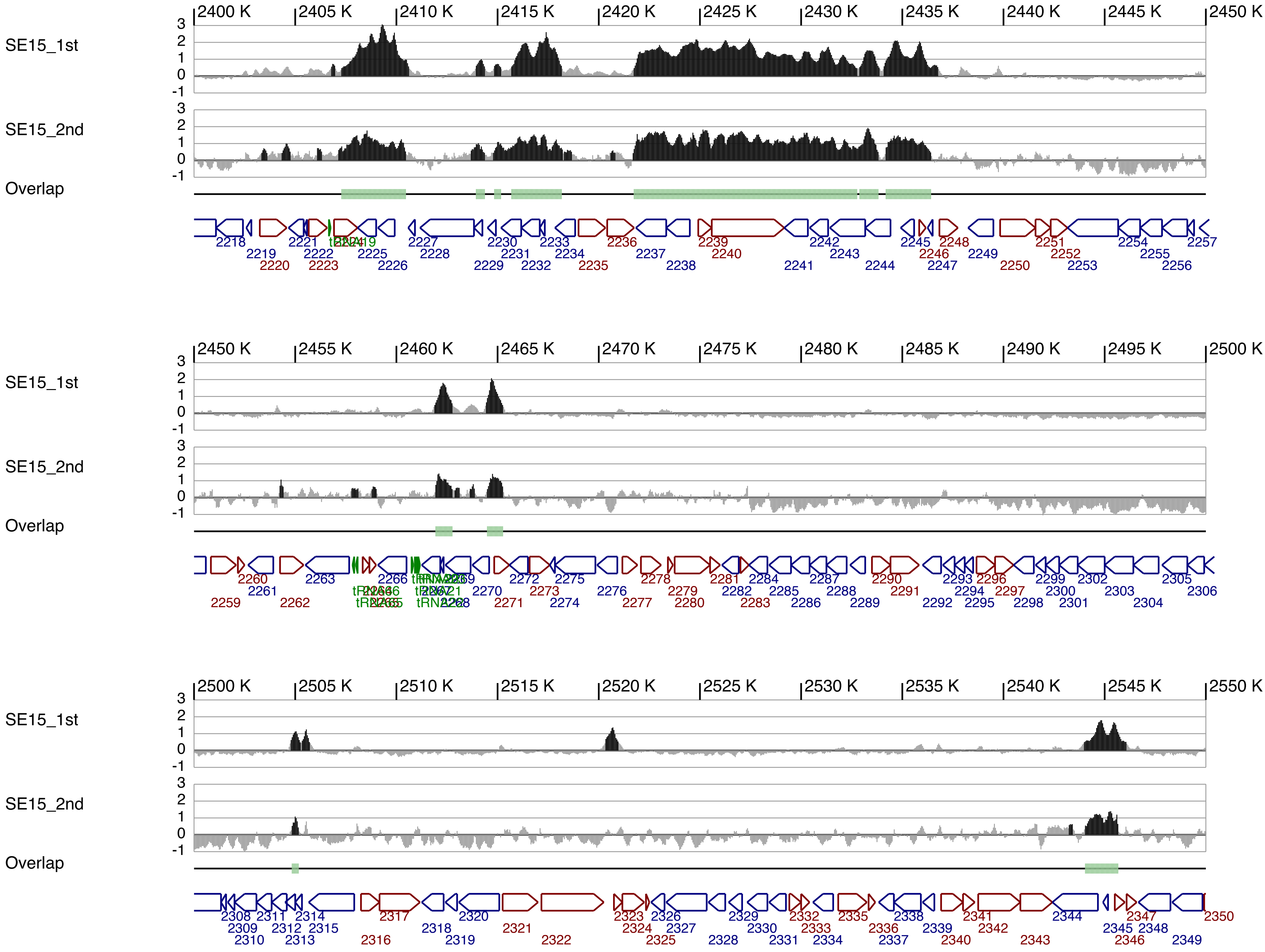
2400 K
2405 K
2410 K
2415 K
2420 K
2425 K
2430 K
2435 K
2440 K
2445 K
2450 K
SE15_1st
SE15_2nd
Overlap
3
2
1
0
-1
2218 2219 2220 2221 2222 2223 2225 2226 2227 2228 2229 2230 2231 2232 2233 2234 2235 2236 2237 2238 2239 2240 2241 2242 2243 2244 2245 2246 2247 2248 2249 2250 2251 2252 2253 2254 2255 2256 2257
2450 K
2455 K
2460 K
2465 K
2470 K
2475 K
2480 K
2485 K
2490 K
2495 K
2500 K
SE15_1st
SE15_2nd
Overlap
2259 2260 2261 2262 2263 2266 2270 2271 2272 2273 2274 2275 2276 2277 2278 2279 2280 2281 2282 2283 2284 2285 2286 2287 2288 2289 2290 2291 2292 2293 2294 2295 2296 2297 2298 2299 2300 2301 2302 2303 2304 2305 2306
2500 K
2505 K
2510 K
2515 K
2520 K
2525 K
2530 K
2535 K
2540 K
2545 K
2550 K
SE15_1st
SE15_2nd
Overlap
2308 2309 2310 2311 2312 2313 2314 2315 2316 2317 2318 2319 2320 2321 2322 2323 2324 2325 2326 2327 2328 2329 2330 2331 2332 2333 2334 2335 2336 2337 2338 2339 2340 2341 2342 2343 2344 2345 2346 2347 2348 2349 2350

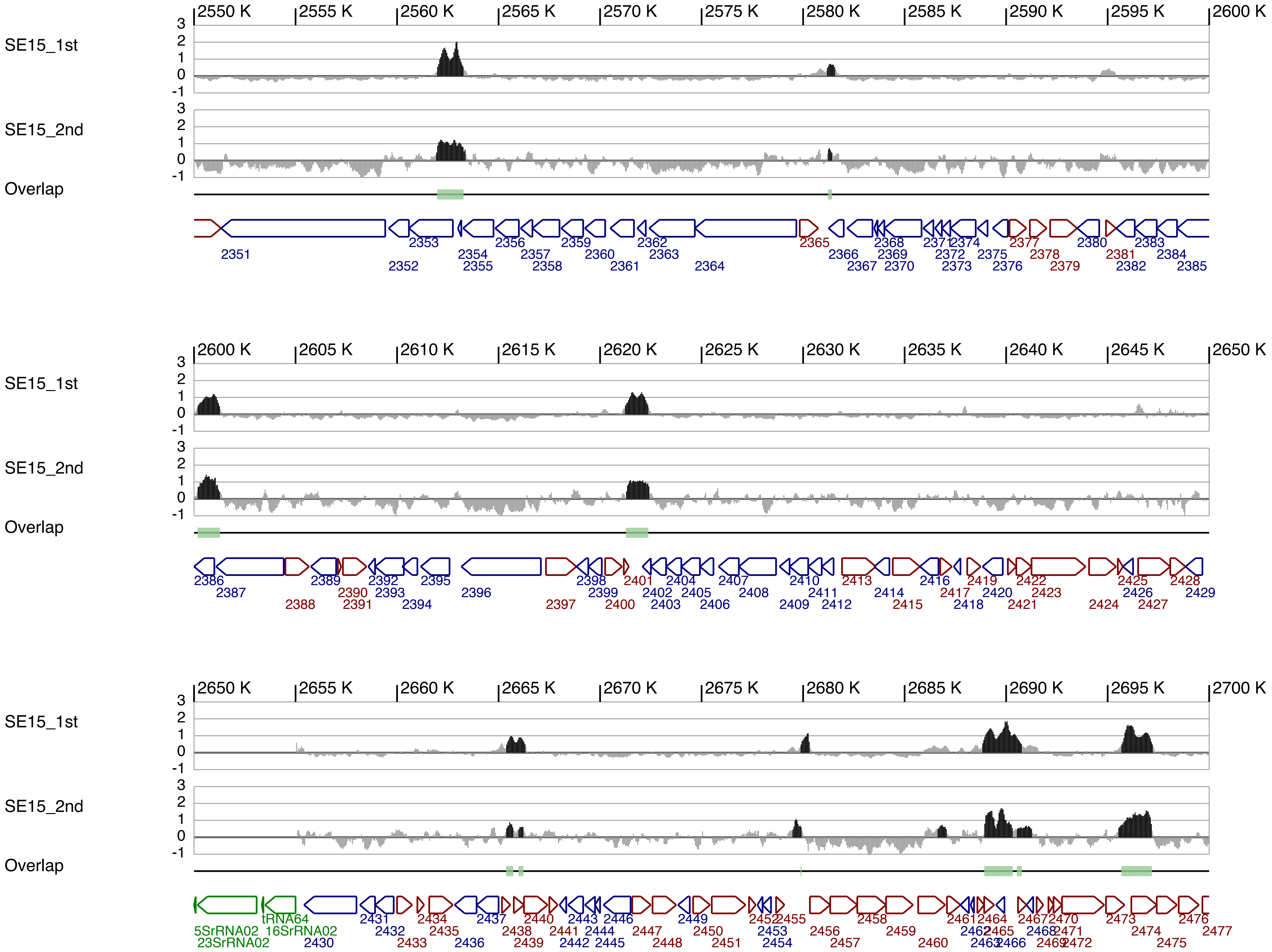
2550 K 2555 K 2560 K 2565 K 2570 K 2575 K 2580 K 2585 K 2590 K 2595 K 2600 K
SE15_1st
SE15_2nd
Overlap
3 2 1 0 -1
2353 2356 2359 2362 2365 2368 2371 2374 2377 2380 2383
2351 2354 2357 2360 2363 2366 2369 2372 2375 2378 2381 2384
2352 2355 2358 2361 2364 2367 2370 2373 2376 2379 2382 2385
2600 K 2605 K 2610 K 2615 K 2620 K 2625 K 2630 K 2635 K 2640 K 2645 K 2650 K
SE15_1st
SE15_2nd
Overlap
2386 2389 2392 2395 2398 2401 2404 2407 2410 2413 2416 2419 2422 2425 2428
2387 2390 2393 2396 2399 2402 2405 2408 2411 2414 2417 2420 2423 2426 2429
2388 2391 2394 2397 2400 2403 2406 2409 2412 2415 2418 2421 2424 2427
2650 K 2655 K 2660 K 2665 K 2670 K 2675 K 2680 K 2685 K 2690 K 2695 K 2700 K
SE15_1st
SE15_2nd
Overlap
tRNA64 2431 2434 2437 2440 2443 2446 2449 2452 2455 2458 2461 2464 2467 2470 2473 2476
5SrRNA02 16SrRNA02 2432 2435 2438 2441 2444 2447 2450 2453 2456 2459 2462 2465 2468 2471 2474 2477
23SrRNA02 2430 2433 2436 2439 2442 2445 2448 2451 2454 2457 2460 2463 2466 2469 2472 2475

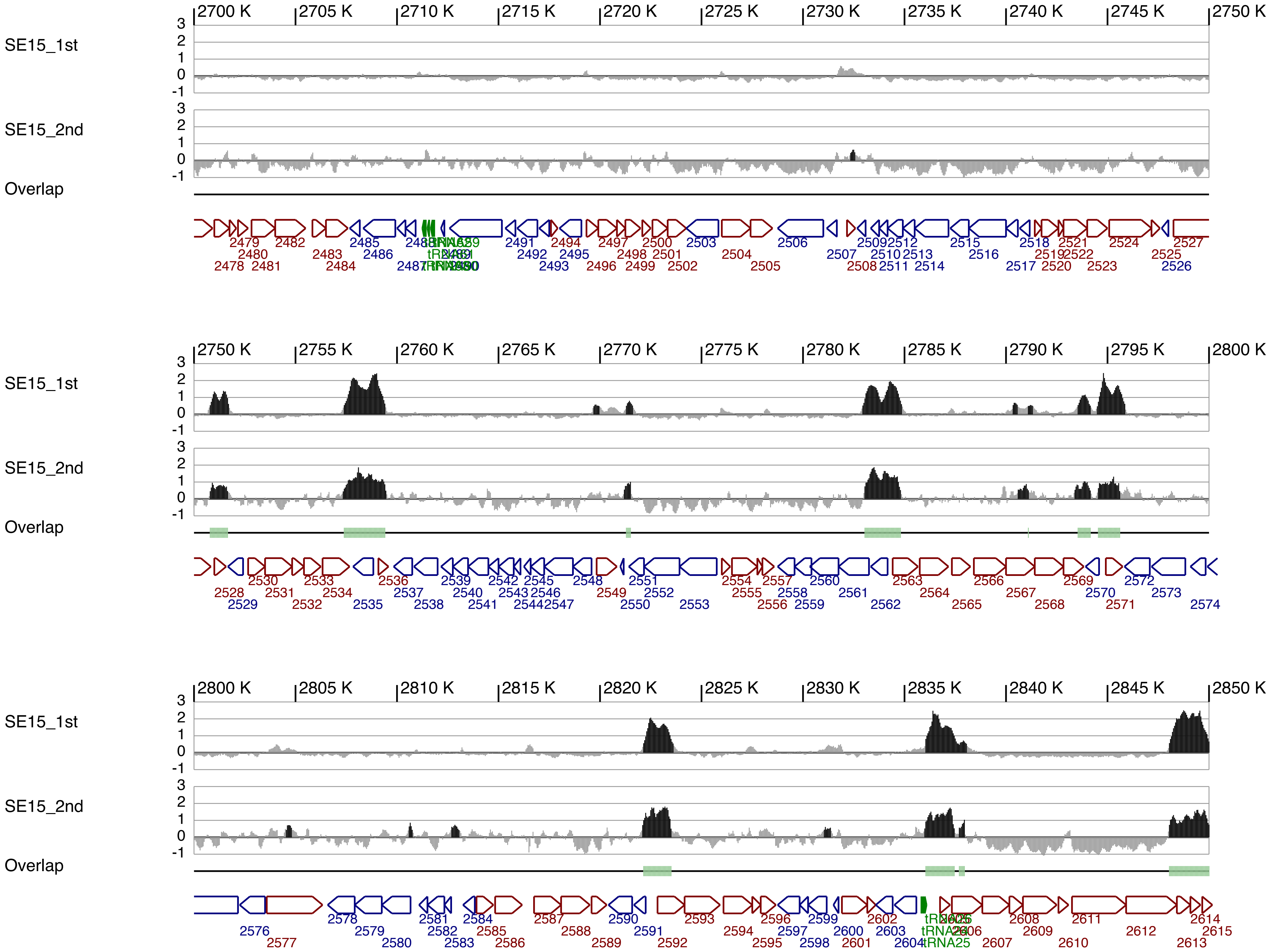

2700 K
2705 K
2710 K
2715 K
2720 K
2725 K
2730 K
2735 K
2740 K
2745 K
2750 K
SE15_1st
SE15_2nd
Overlap
3
2
1
0
-1
2479 2482 2485 2491 2494 2497 2500 2503 2506 2509 2512 2515 2518 2521 2524 2527
2480 2483 2486 2492 2495 2498 2501 2504 2507 2510 2513 2516 2519 2522 2525
2478 2481 2484 2487 2493 2496 2499 2502 2505 2508 2511 2514 2517 2520 2523 2526
2755 K
2760 K
2765 K
2770 K
2775 K
2780 K
2785 K
2790 K
2795 K
2800 K
2530 2533 2536 2539 2542 2545 2548 2551 2554 2557 2560 2563 2566 2569 2572
2528 2531 2534 2537 2540 2543 2546 2549 2552 2555 2558 2561 2564 2567 2570 2573
2529 2532 2535 2538 2541 2544 2547 2550 2553 2556 2559 2562 2565 2568 2571 2574
2805 K
2810 K
2815 K
2820 K
2825 K
2830 K
2835 K
2840 K
2845 K
2850 K
2578 2581 2584 2587 2590 2593 2596 2599 2602 2608 2611 2614
2576 2579 2582 2585 2588 2591 2594 2597 2600 2603 2606 2609 2612 2615
2577 2580 2583 2586 2589 2592 2595 2598 2601 2604 tRNA25 2607 2610 2613

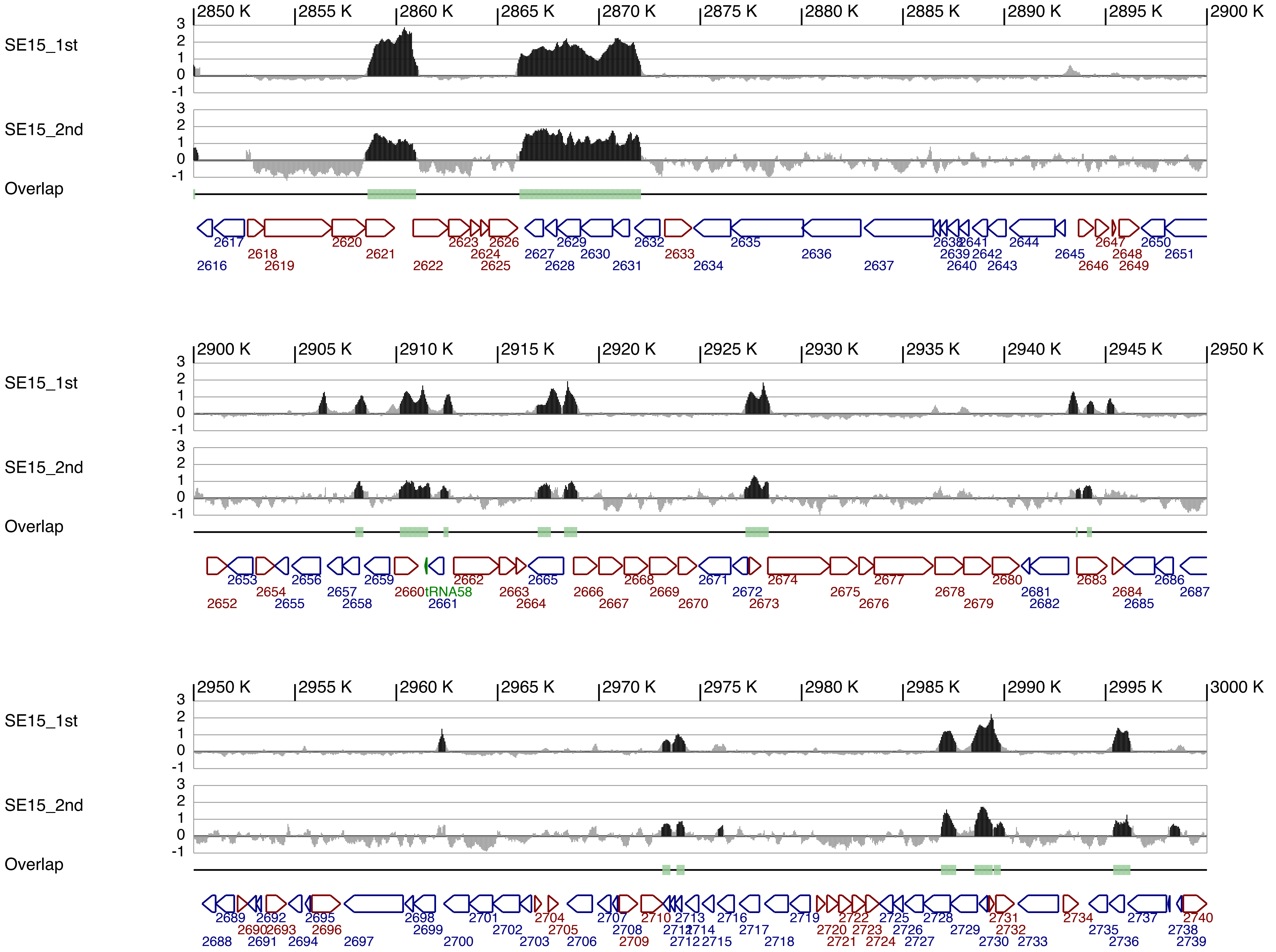

2850 K
2855 K
2860 K
2865 K
2870 K
2875 K
2880 K
2885 K
2890 K
2895 K
2900 K
SE15_1st
SE15_2nd
Overlap
3
2
1
0
-1
2616 2617 2618 2619 2620 2621 2622 2623 2624 2625 2626 2627 2628 2629 2630 2631 2632 2633 2634 2635 2636 2637 2638 2639 2640 2641 2642 2643 2644 2645 2646 2647 2648 2649 2650 2651
2905 K
2910 K
2915 K
2920 K
2925 K
2930 K
2935 K
2940 K
2945 K
2950 K
2652 2653 2654 2655 2656 2657 2658 2659 2660 tRNA58 2661 2662 2663 2664 2665 2666 2667 2668 2669 2670 2671 2672 2673 2674 2675 2676 2677 2678 2679 2680 2681 2682 2683 2684 2685 2686 2687
2955 K
2960 K
2965 K
2970 K
2975 K
2980 K
2985 K
2990 K
2995 K
3000 K
2688 2689 2690 2691 2692 2693 2694 2695 2696 2697 2698 2699 2700 2701 2702 2703 2704 2705 2706 2707 2708 2709 2710 2711 2712 2713 2714 2715 2716 2717 2718 2719 2720 2721 2722 2723 2724 2725 2726 2727 2728 2729 2730 2731 2732 2733 2734 2735 2736 2737 2738 2739 2740

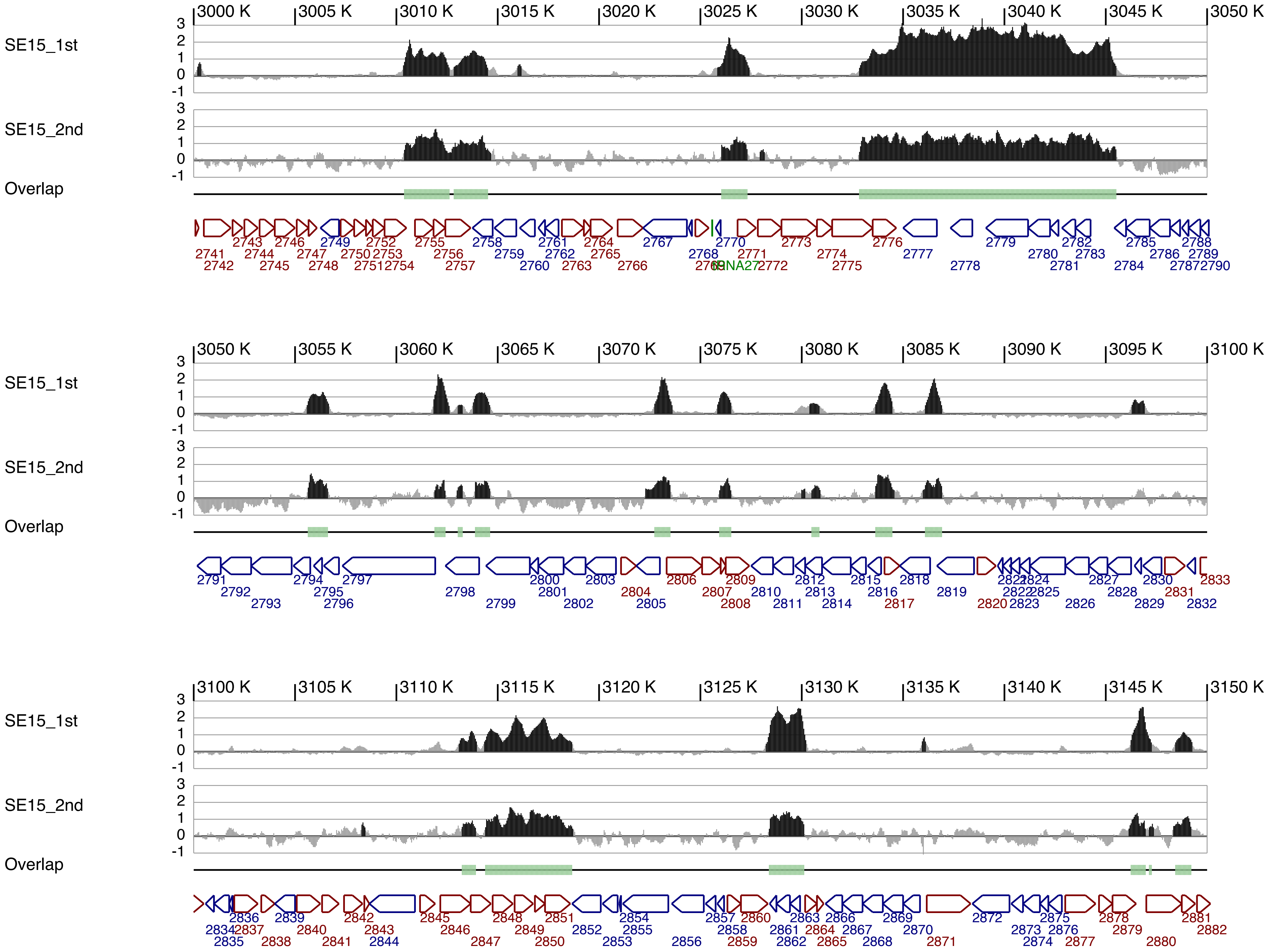
3000 K
3005 K
3010 K
3015 K
3020 K
3025 K
3030 K
3035 K
3040 K
3045 K
3050 K
SE15_1st
SE15_2nd
Overlap
2741
2742
2743
2744
2745
2746
2747
2748
2749
2750
2751
2752
2753
2754
2755
2756
2757
2758
2759
2760
2761
2762
2763
2764
2765
2766
2767
2768
2770
2771
2772
2773
2774
2775
2776
2777
2778
2779
2780
2781
2782
2783
2784
2785
2786
2787
2788
2789
2790
3055 K
3060 K
3065 K
3070 K
3075 K
3080 K
3085 K
3090 K
3095 K
3100 K
2791
2792
2793
2794
2795
2796
2797
2798
2799
2800
2801
2802
2803
2804
2805
2806
2807
2808
2809
2810
2811
2812
2813
2814
2815
2816
2817
2818
2819
2820
2823
2824
2825
2826
2827
2828
2829
2830
2831
2832
2833
3105 K
3110 K
3115 K
3120 K
3125 K
3130 K
3135 K
3140 K
3145 K
3150 K
2834
2835
2836
2837
2838
2839
2840
2841
2842
2843
2844
2845
2846
2847
2848
2849
2850
2851
2852
2853
2854
2855
2856
2857
2858
2859
2860
2861
2862
2863
2864
2865
2866
2867
2868
2869
2870
2871
2872
2873
2874
2875
2876
2877
2878
2879
2880
2881
2882

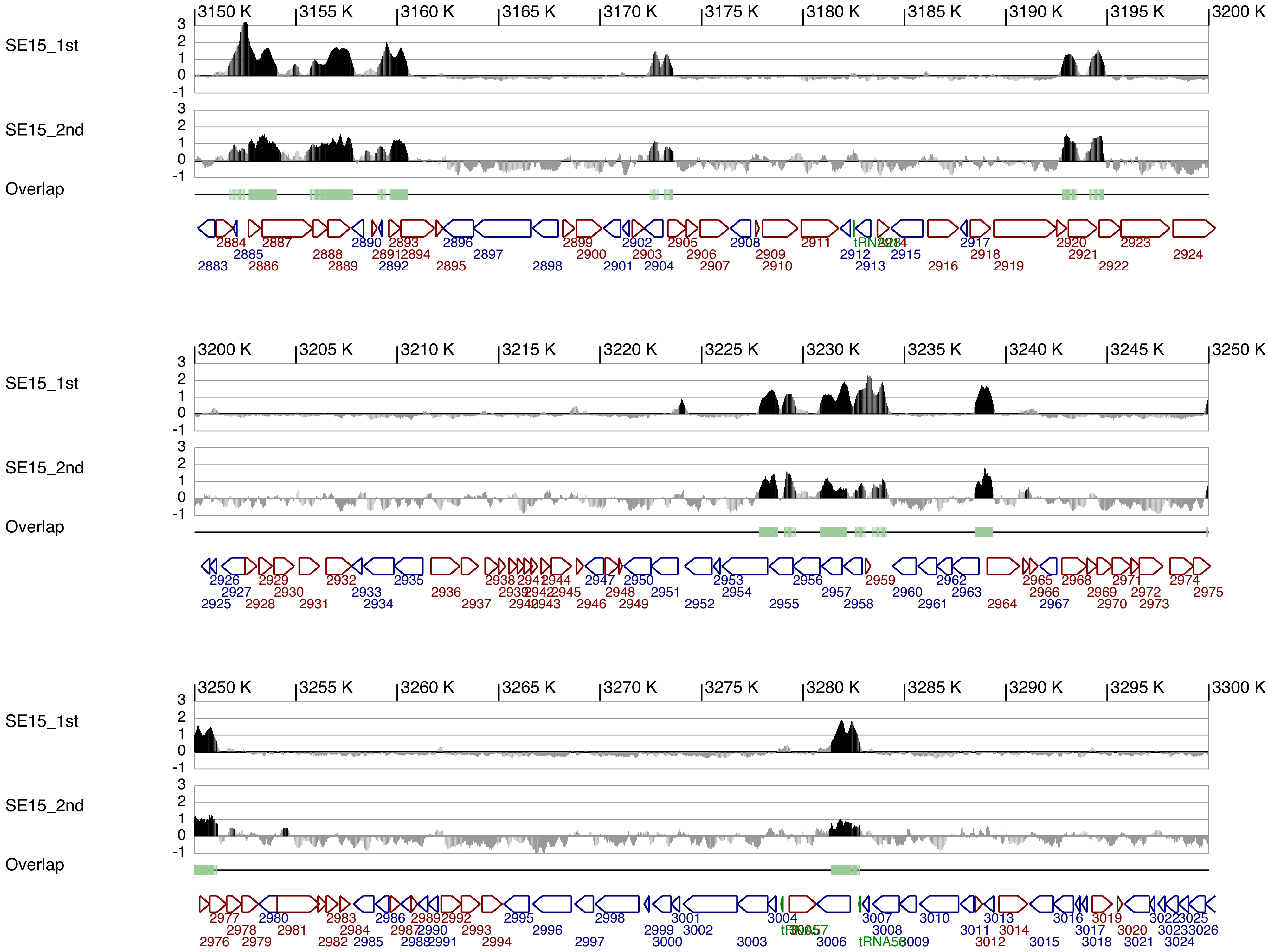

3150 K 3155 K 3160 K 3165 K 3170 K 3175 K 3180 K 3185 K 3190 K 3195 K 3200 K
SE15_1st
SE15_2nd
Overlap
2883 2884 2885 2886 2887 2888 2889 2890 2891 2892 2893 2894 2895 2896 2897 2898 2899 2900 2901 2902 2903 2904 2905 2906 2907 2908 2909 2910 2911 2912 2913 2915 2916 2917 2918 2919 2920 2921 2922 2923 2924
3200 K 3205 K 3210 K 3215 K 3220 K 3225 K 3230 K 3235 K 3240 K 3245 K 3250 K
SE15_1st
SE15_2nd
Overlap
2925 2926 2927 2928 2929 2930 2931 2932 2933 2934 2935 2936 2937 2938 2939 2944 2945 2946 2947 2948 2949 2950 2951 2952 2953 2954 2955 2956 2957 2958 2959 2960 2961 2962 2963 2964 2965 2966 2967 2968 2969 2970 2971 2972 2973 2974 2975
3250 K 3255 K 3260 K 3265 K 3270 K 3275 K 3280 K 3285 K 3290 K 3295 K 3300 K
SE15_1st
SE15_2nd
Overlap
2976 2977 2978 2979 2980 2981 2982 2983 2984 2985 2986 2987 2990 2991 2993 2994 2995 2996 2997 2998 2999 3000 3001 3002 3003 3004 3006 3007 3008 3010 3011 3012 3013 3014 3015 3016 3017 3018 3019 3020 3021 3024 3025

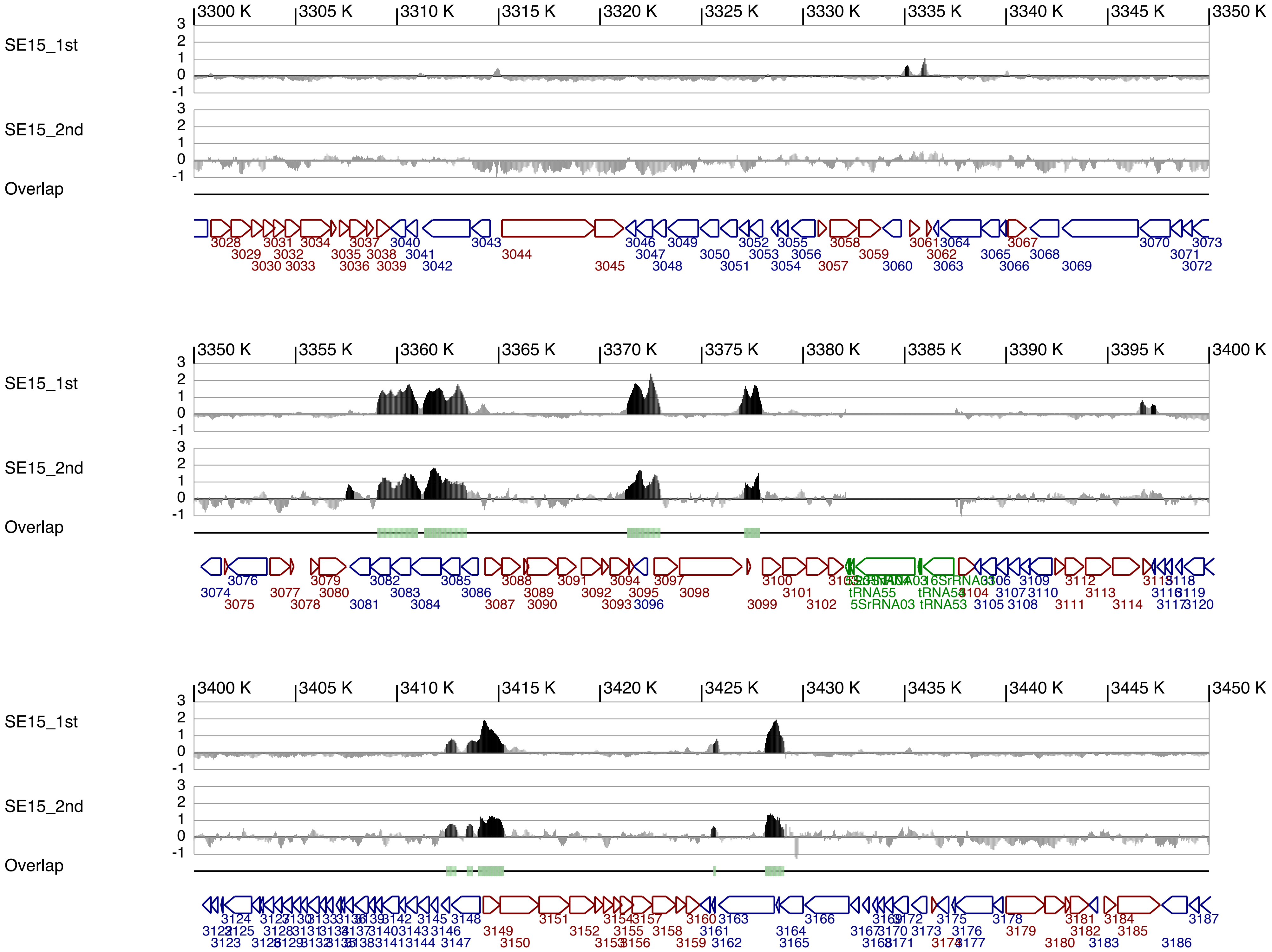
3300 K
3305 K
3310 K
3315 K
3320 K
3325 K
3330 K
3335 K
3340 K
3345 K
3350 K
SE15_1st
SE15_2nd
Overlap
3028 3031 3034 3037 3040 3043 3046 3049 3052 3055 3058 3061 3064 3067 3070 3073
3029 3032 3035 3038 3041 3044 3047 3050 3053 3056 3059 3062 3065 3068 3071
3030 3033 3036 3039 3042 3045 3048 3051 3054 3057 3060 3063 3066 3069 3072
3350 K
3355 K
3360 K
3365 K
3370 K
3375 K
3380 K
3385 K
3390 K
3395 K
3400 K
SE15_1st
SE15_2nd
Overlap
3076 3079 3082 3085 3088 3091 3094 3097 3100 3109 3112 3118
3074 3077 3080 3083 3086 3089 3092 3095 3098 3101 tRNA55 tRNA54 3104 3107 3110 3113 3119
3075 3078 3081 3084 3087 3090 3093 3096 3099 3102 5SrRNA03 tRNA53 3105 3108 3111 3114 3120
3400 K
3405 K
3410 K
3415 K
3420 K
3425 K
3430 K
3435 K
3440 K
3445 K
3450 K
SE15_1st
SE15_2nd
Overlap
3124 3142 3145 3148 3151 3157 3160 3163 3166 3172 3175 3178 3181 3184 3187
3125 3128 3143 3146 3149 3152 3155 3158 3161 3164 3170 3173 3176 3179 3182 3185
3123 3144 3147 3150 3156 3159 3162 3165 3171 3177 3180 3183 3186

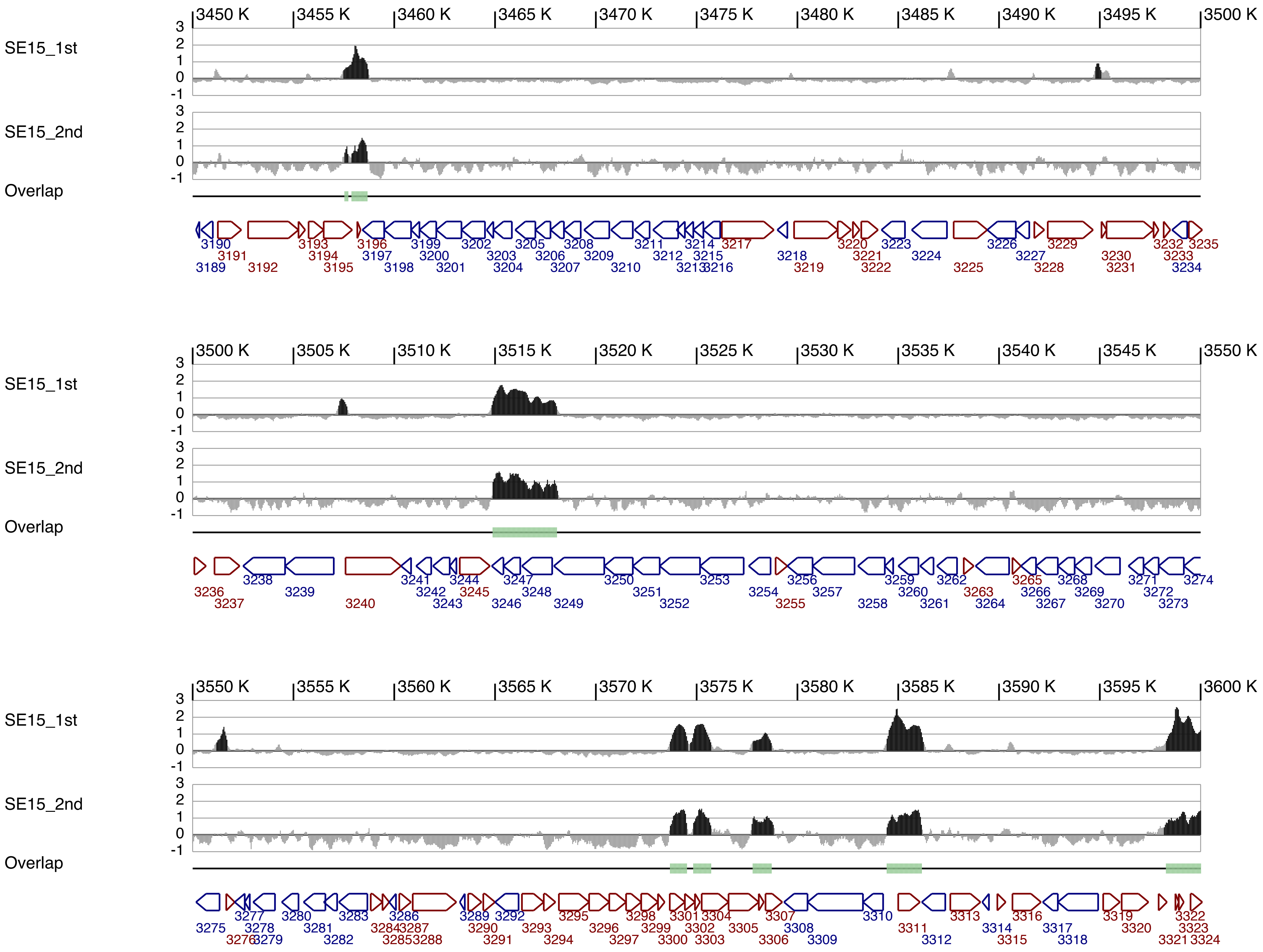

3450 K 3455 K 3460 K 3465 K 3470 K 3475 K 3480 K 3485 K 3490 K 3495 K 3500 K
SE15_1st
SE15_2nd
Overlap
3 2 1 0 -1
3189 3190 3191 3192 3193 3194 3195 3196 3197 3198 3199 3200 3201 3202 3203 3204 3205 3206 3207 3208 3209 3210 3211 3212 3213 3214 3215 3216 3217 3218 3219 3220 3221 3222 3223 3224 3225 3226 3227 3228 3229 3230 3231 3232 3233 3234 3235
3500 K 3505 K 3510 K 3515 K 3520 K 3525 K 3530 K 3535 K 3540 K 3545 K 3550 K
SE15_1st
SE15_2nd
Overlap
3236 3237 3238 3239 3240 3241 3242 3243 3244 3245 3246 3247 3248 3249 3250 3251 3252 3253 3254 3255 3256 3257 3258 3259 3260 3261 3262 3263 3264 3265 3266 3267 3268 3269 3270 3271 3272 3273 3274
3550 K 3555 K 3560 K 3565 K 3570 K 3575 K 3580 K 3585 K 3590 K 3595 K 3600 K
SE15_1st
SE15_2nd
Overlap
3275 3276 3277 3278 3279 3280 3281 3282 3283 3284 3285 3286 3287 3288 3289 3290 3291 3292 3293 3294 3295 3296 3297 3298 3299 3300 3301 3302 3303 3304 3305 3306 3307 3308 3309 3310 3311 3312 3313 3314 3315 3316 3317 3318 3319 3320 3321 3322 3323 3324

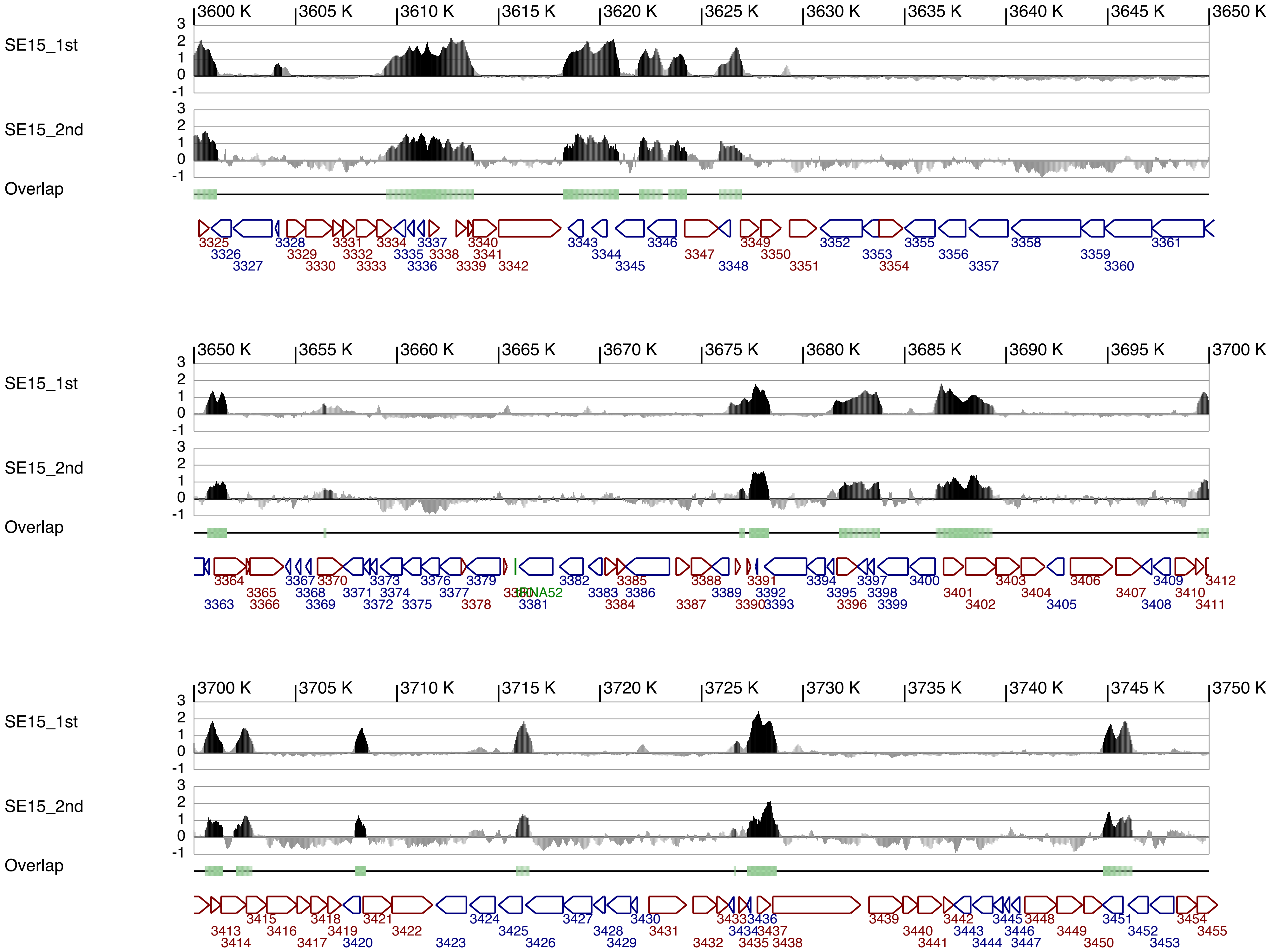

3600 K
3605 K
3610 K
3615 K
3620 K
3625 K
3630 K
3635 K
3640 K
3645 K
3650 K
SE15_1st
SE15_2nd
Overlap
3325 3328 3331 3334 3337 3340 3343 3346 3349 3352 3355 3358 3361
3326 3329 3332 3335 3338 3341 3344 3347 3350 3353 3356 3359
3327 3330 3333 3336 3339 3342 3345 3348 3351 3354 3357 3360
3650 K
3655 K
3660 K
3665 K
3670 K
3675 K
3680 K
3685 K
3690 K
3695 K
3700 K
SE15_1st
SE15_2nd
Overlap
3364 3367 3370 3373 3376 3379 3382 3385 3388 3391 3394 3397 3400 3403 3406 3409 3412
3365 3368 3371 3374 3377 3380 tRNA52 3383 3386 3389 3392 3395 3398 3401 3404 3407 3410
3363 3366 3369 3372 3375 3378 3381 3384 3387 3390 3393 3396 3399 3402 3405 3408 3411
3700 K
3705 K
3710 K
3715 K
3720 K
3725 K
3730 K
3735 K
3740 K
3745 K
3750 K
SE15_1st
SE15_2nd
Overlap
3415 3418 3421 3424 3427 3430 3433 3436 3439 3442 3445 3448 3451 3454
3413 3416 3419 3422 3425 3428 3431 3434 3437 3440 3443 3446 3449 3452 3455
3414 3417 3420 3423 3426 3429 3432 3435 3438 3441 3444 3447 3450 3453

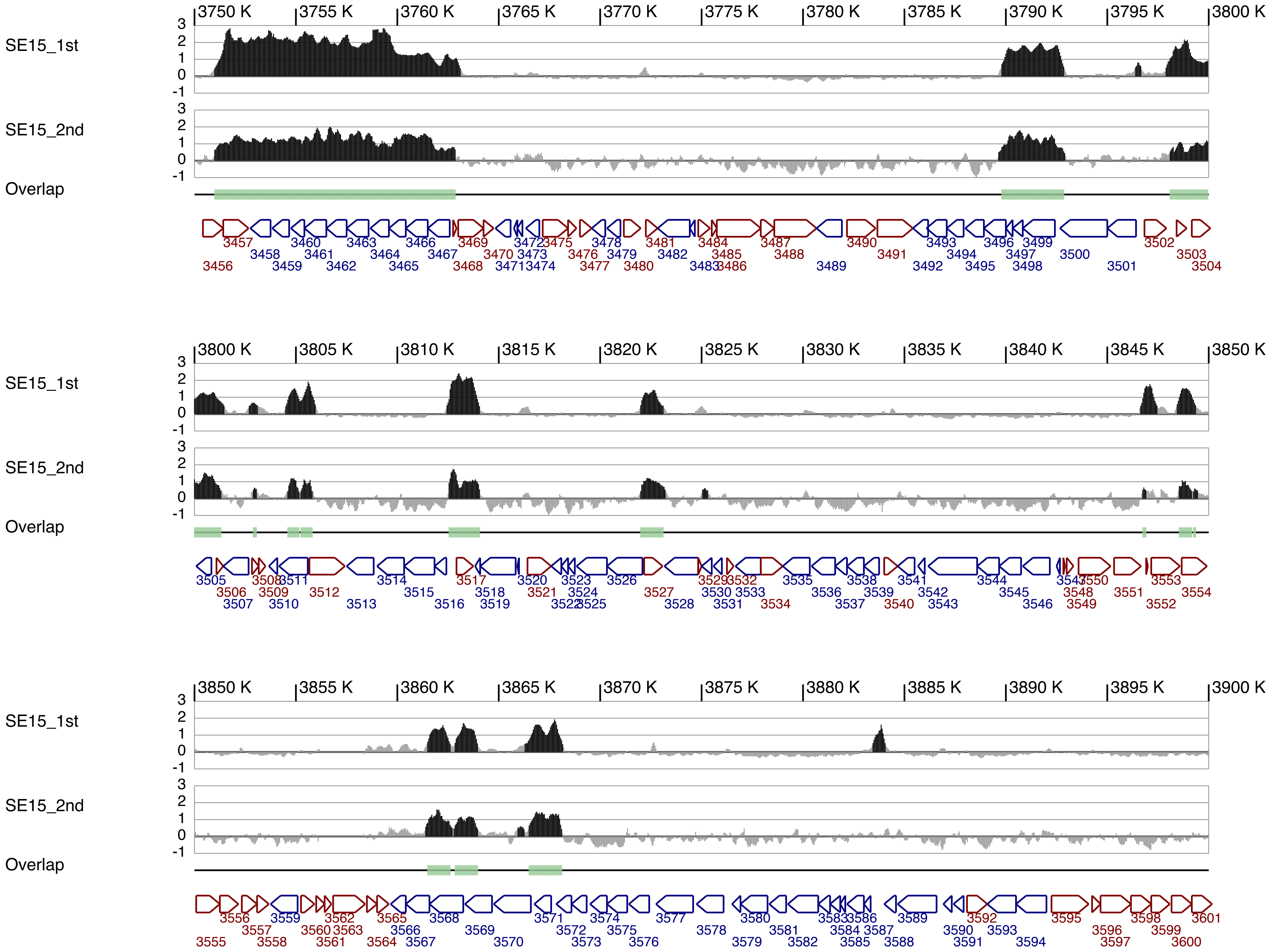

3750 K
3755 K
3760 K
3765 K
3770 K
3775 K
3780 K
3785 K
3790 K
3795 K
3800 K
SE15_1st
SE15_2nd
Overlap
3 2 1 0 -1
3456 3457 3458 3459 3460 3461 3462 3463 3464 3465 3466 3467 3468 3469 3470 3471 3472 3473 3474 3475 3476 3477 3478 3479 3480 3481 3482 3483 3484 3485 3486 3487 3488 3489 3490 3491 3492 3493 3494 3495 3496 3497 3498 3499 3500 3501 3502 3503 3504
3800 K
3805 K
3810 K
3815 K
3820 K
3825 K
3830 K
3835 K
3840 K
3845 K
3850 K
SE15_1st
SE15_2nd
Overlap
3505 3506 3507 3508 3509 3510 3511 3512 3513 3514 3515 3516 3517 3518 3519 3520 3521 3522 3523 3524 3525 3526 3527 3528 3529 3530 3531 3532 3533 3534 3535 3536 3537 3538 3539 3540 3541 3542 3543 3544 3545 3546 3547 3548 3549 3550 3551 3552 3553 3554
3850 K
3855 K
3860 K
3865 K
3870 K
3875 K
3880 K
3885 K
3890 K
3895 K
3900 K
SE15_1st
SE15_2nd
Overlap
3555 3556 3557 3558 3559 3560 3561 3562 3563 3564 3565 3566 3567 3568 3569 3570 3571 3572 3573 3574 3575 3576 3577 3578 3579 3580 3581 3582 3583 3584 3585 3586 3587 3588 3589 3590 3591 3592 3593 3594 3595 3596 3597 3598 3599 3600 3601

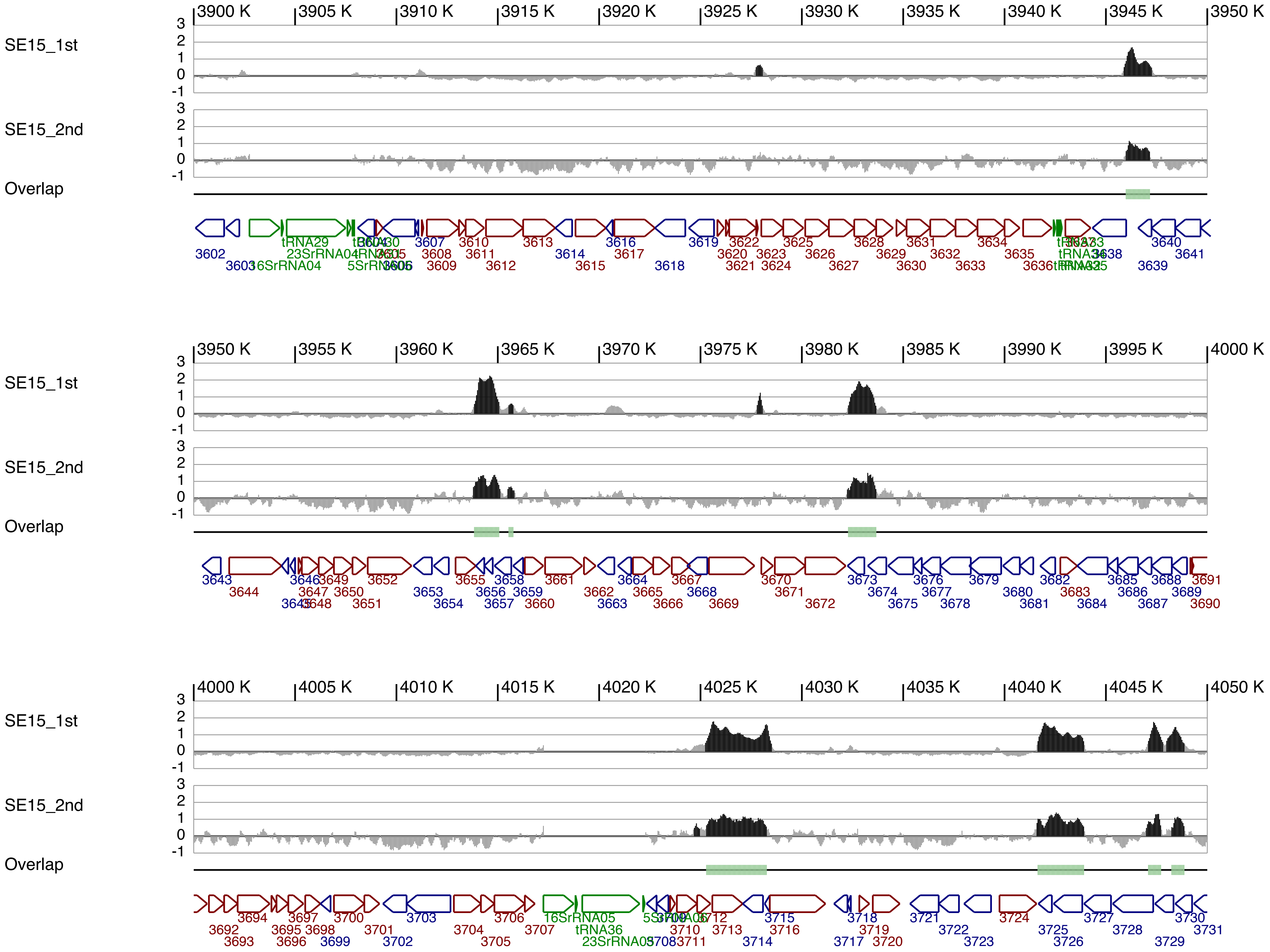

3900 K
3905 K
3910 K
3915 K
3920 K
3925 K
3930 K
3935 K
3940 K
3945 K
3950 K
SE15_1st
SE15_2nd
Overlap
3
2
1
0
-1
3602
16SrRNA04
tRNA29
23SrRNA04
5SrRNA05
3607
3608
3609
3610
3611
3612
3613
3614
3615
3616
3617
3618
3619
3620
3621
3622
3623
3624
3625
3626
3627
3628
3629
3630
3631
3632
3633
3634
3635
3638
3639
3640
3641
3955 K
3960 K
3965 K
3970 K
3975 K
3980 K
3985 K
3990 K
3995 K
4000 K
3643
3644
3646
3647
3649
3650
3651
3652
3653
3654
3655
3656
3657
3658
3659
3660
3661
3662
3663
3664
3665
3666
3667
3668
3669
3670
3671
3672
3673
3674
3675
3676
3677
3678
3679
3680
3681
3682
3683
3684
3685
3686
3687
3688
3689
3690
3691
4005 K
4010 K
4015 K
4020 K
4025 K
4030 K
4035 K
4040 K
4045 K
4050 K
3692
3693
3694
3695
3696
3697
3698
3699
3700
3701
3702
3703
3704
3705
3706
3707
16SrRNA05
tRNA36
23SrRNA05
3710
3711
3713
3714
3715
3716
3717
3718
3719
3720
3721
3722
3723
3724
3725
3726
3727
3728
3729
3730
3731

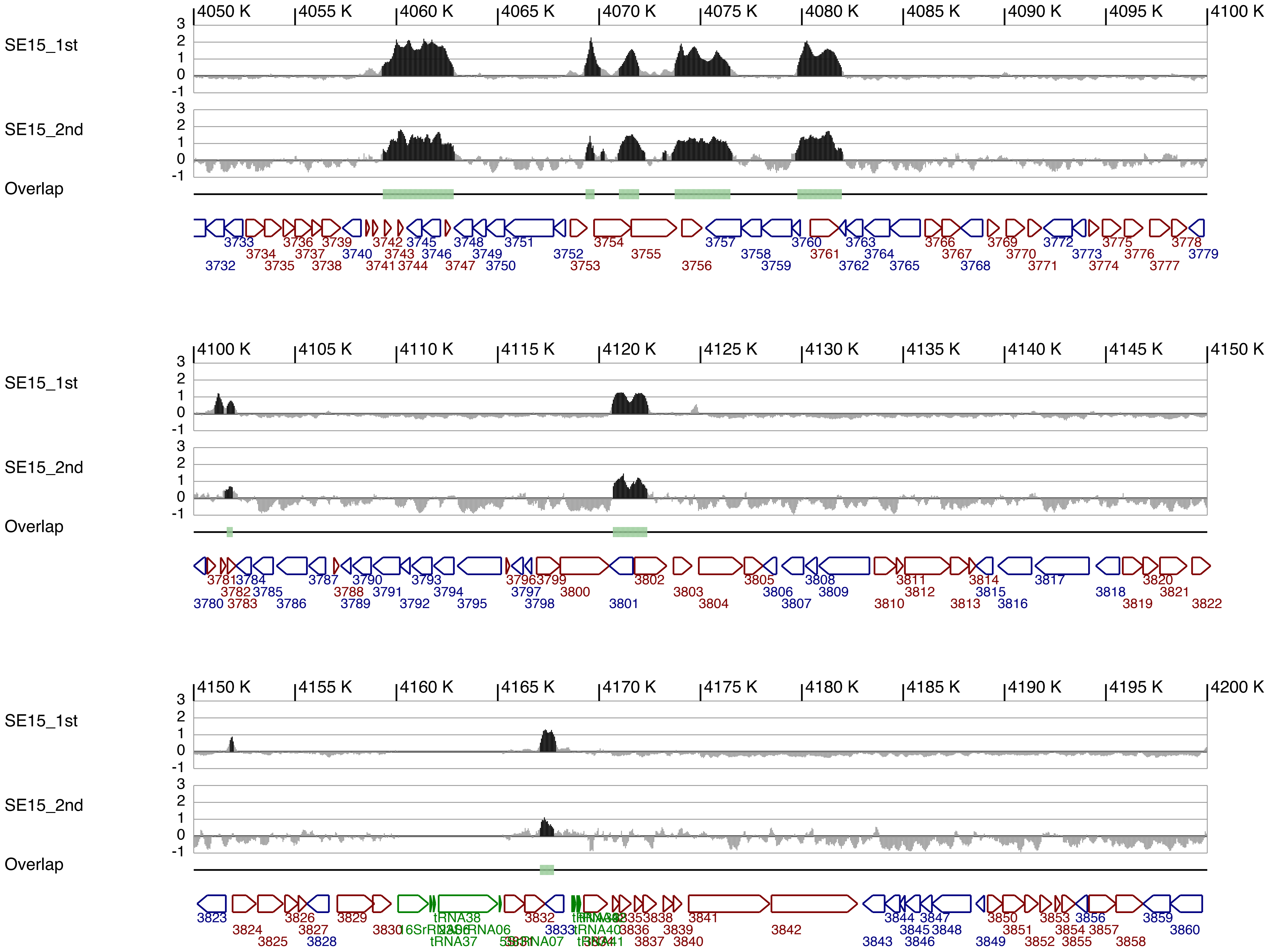

4050 K
4055 K
4060 K
4065 K
4070 K
4075 K
4080 K
4085 K
4090 K
4095 K
4100 K
SE15_1st
SE15_2nd
Overlap
3732 3733 3734 3735 3736 3737 3738 3739 3740 3741 3742 3743 3744 3745 3746 3747 3748 3749 3750 3751 3752 3753 3754 3755 3756 3757 3758 3759 3760 3761 3762 3763 3764 3765 3766 3767 3768 3769 3770 3771 3772 3773 3774 3775 3776 3777 3778 3779
4105 K
4110 K
4115 K
4120 K
4125 K
4130 K
4135 K
4140 K
4145 K
4150 K
3780 3782 3783 3784 3785 3786 3787 3788 3789 3790 3791 3792 3793 3794 3795 3797 3798 3800 3801 3802 3803 3804 3805 3806 3807 3808 3809 3810 3811 3812 3813 3814 3815 3816 3817 3818 3819 3820 3821 3822
4155 K
4160 K
4165 K
4170 K
4175 K
4180 K
4185 K
4190 K
4195 K
4200 K
3823 3824 3825 3826 3827 3828 3829 3830 3832 3833 3836 3837 3838 3839 3840 3841 3842 3843 3844 3845 3846 3847 3848 3849 3850 3851 3852 3853 3854 3855 3856 3857 3858 3859 3860
tRNA38 tRNA37 tRNA40

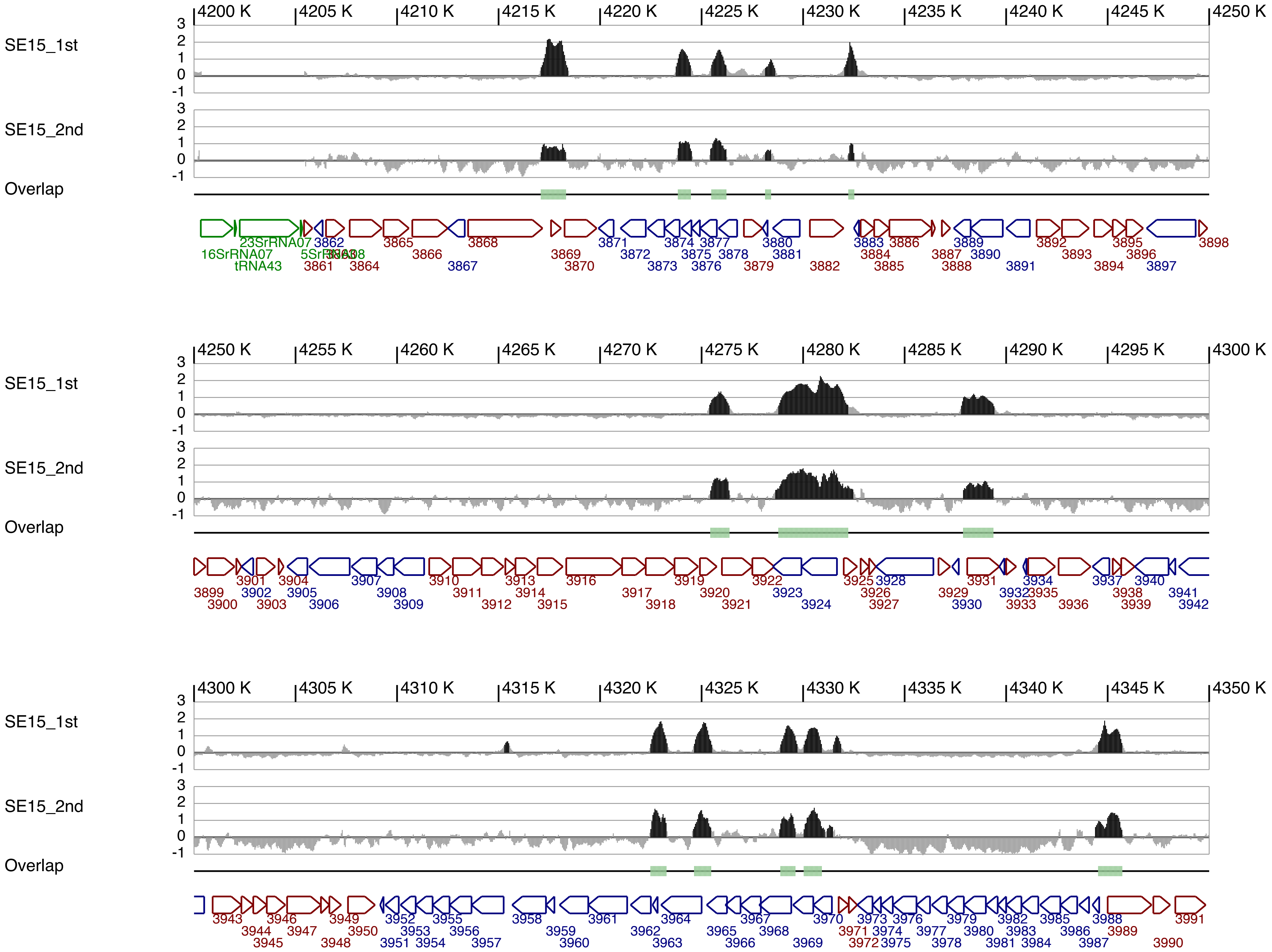

SE15_1st
SE15_2nd
Overlap
4200 K
4205 K
4210 K
4215 K
4220 K
4225 K
4230 K
4235 K
4240 K
4245 K
4250 K
4255 K
4260 K
4265 K
4270 K
4275 K
4280 K
4285 K
4290 K
4295 K
4300 K
4305 K
4310 K
4315 K
4320 K
4325 K
4330 K
4335 K
4340 K
4345 K
4350 K
16SrRNA07
23SrRNA07
tRNA43
5SrRNA08

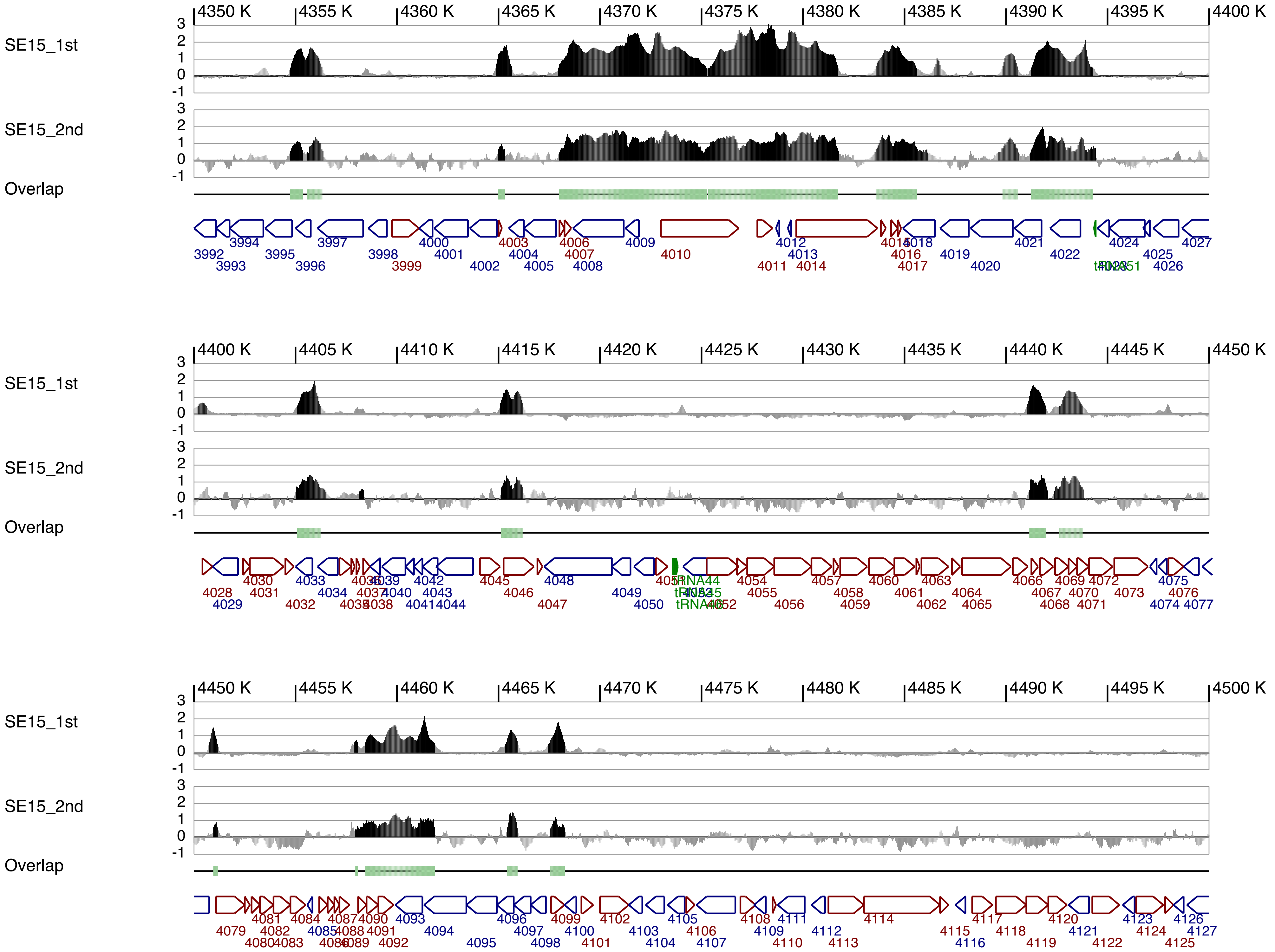
4350 K
4355 K
4360 K
4365 K
4370 K
4375 K
4380 K
4385 K
4390 K
4395 K
4400 K
SE15_1st
SE15_2nd
Overlap
3
2
1
0
-1
3994 3997 4000 4003 4006 4009 4012 4018 4021 4024 4027
3992 3995 3998 4001 4004 4007 4010 4013 4016 4019 4022 4025
3993 3996 3999 4002 4005 4008 4011 4014 4017 4020 4026
4400 K
4405 K
4410 K
4415 K
4420 K
4425 K
4430 K
4435 K
4440 K
4445 K
4450 K
SE15_1st
SE15_2nd
Overlap
4030 4033 4039 4042 4045 4048 4054 4057 4060 4063 4066 4069 4072 4075
4028 4031 4034 4040 4043 4046 4049 4055 4058 4061 4064 4067 4070 4073 4076
4029 4032 4038 4044 4047 4050 4056 4059 4062 4065 4068 4071 4074 4077
4450 K
4455 K
4460 K
4465 K
4470 K
4475 K
4480 K
4485 K
4490 K
4495 K
4500 K
SE15_1st
SE15_2nd
Overlap
4081 4084 4087 4090 4093 4096 4099 4102 4105 4108 4111 4114 4117 4120 4123 4126
4079 4082 4085 4088 4091 4094 4097 4100 4103 4106 4109 4112 4115 4118 4121 4124 4127
4092 4095 4098 4101 4104 4107 4110 4113 4116 4119 4122 4125

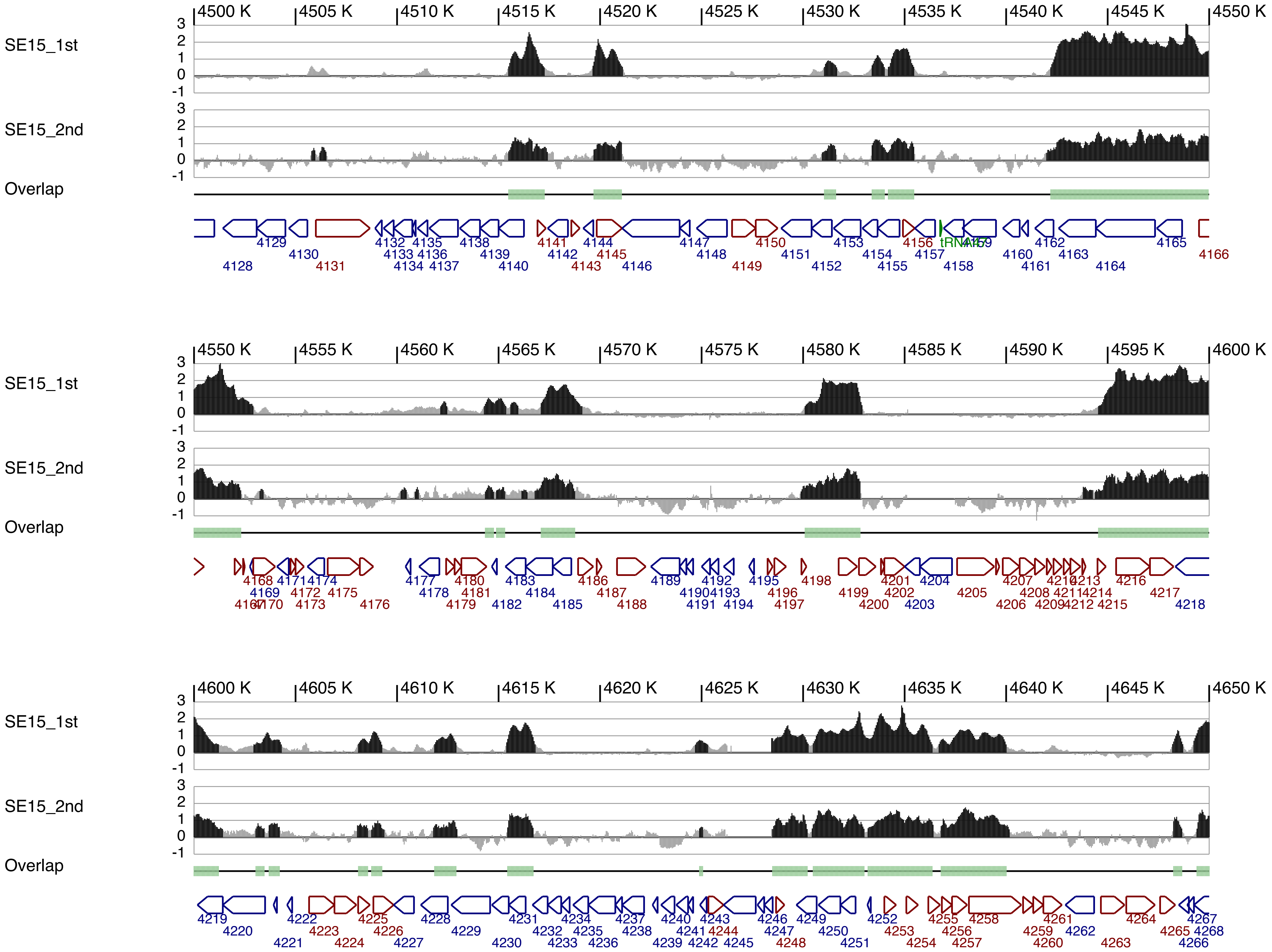

4500 K 4505 K 4510 K 4515 K 4520 K 4525 K 4530 K 4535 K 4540 K 4545 K 4550 K
SE15_1st
SE15_2nd
Overlap
3 2 1 0 -1
4129 4132 4135 4138 4141 4144 4147 4150 4153 4156 tRNA4159 4162 4165
4130 4133 4136 4139 4142 4145 4148 4151 4154 4157 4160 4163 4166
4128 4131 4134 4137 4140 4143 4146 4149 4152 4155 4158 4161 4164
4550 K 4555 K 4560 K 4565 K 4570 K 4575 K 4580 K 4585 K 4590 K 4595 K 4600 K
SE15_1st
SE15_2nd
Overlap
3 2 1 0 -1
4168 41714174 4177 4180 4183 4186 4189 4192 4195 4198 4201 4204 4207 4210 4213 4216
4169 4172 4175 4178 4181 4184 4187 41904193 4196 4199 4202 4205 4208 42114214 4217
4167 4170 4173 4176 4179 4182 4185 4188 4191 4194 4197 4200 4203 4206 4209 4212 4215 4218
4600 K 4605 K 4610 K 4615 K 4620 K 4625 K 4630 K 4635 K 4640 K 4645 K 4650 K
SE15_1st
SE15_2nd
Overlap
3 2 1 0 -1
4219 4222 4225 4228 4231 4234 4237 4240 4243 4246 4249 4252 4255 4258 4261 4264 4267
4220 4223 4226 4229 4232 4235 4238 4241 4244 4247 4250 4253 4256 4259 4262 4265 4268
4221 4224 4227 4230 4233 4236 4239 4242 4245 4248 4251 4254 4257 4260 4263 4266

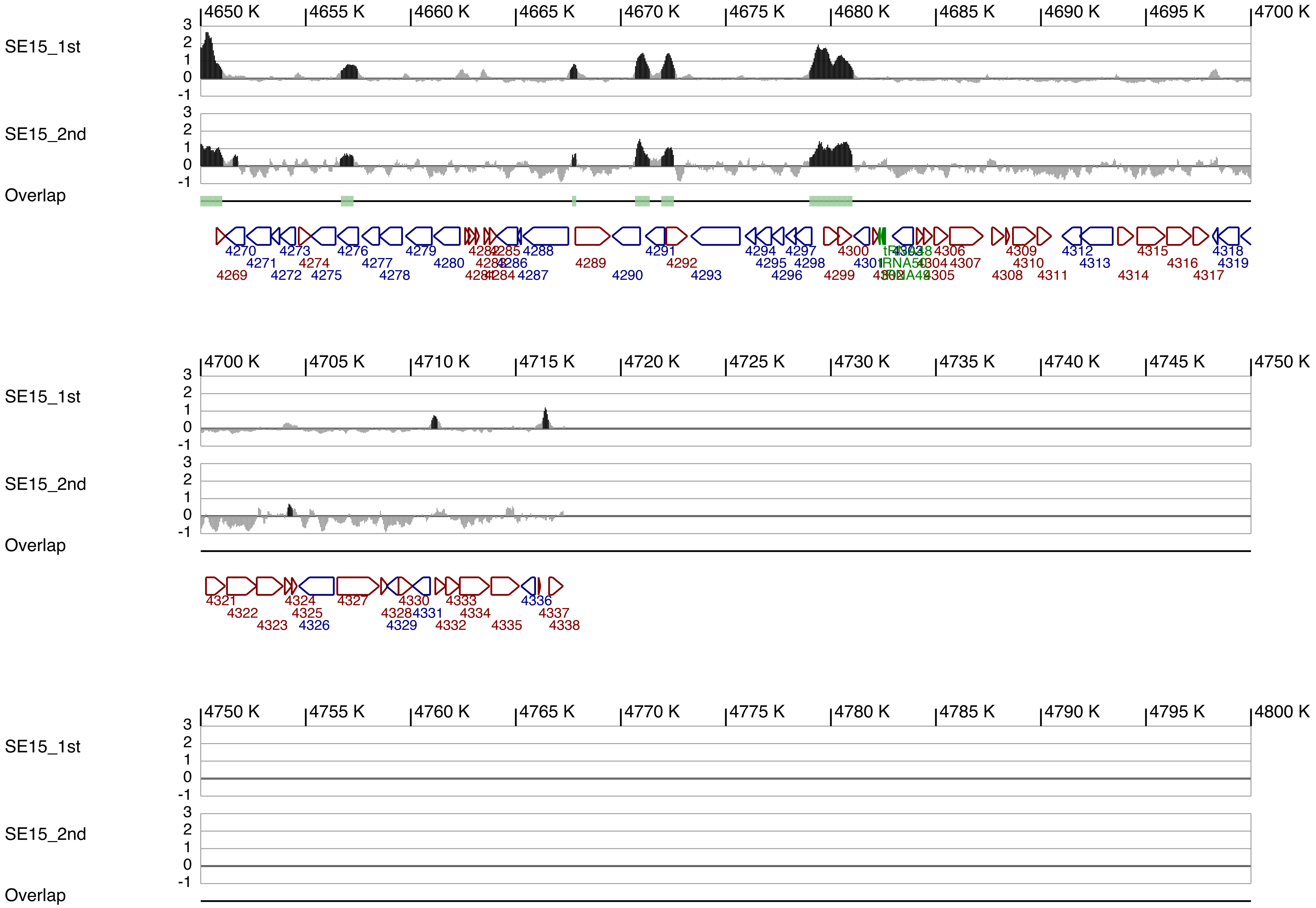

4650 K
4655 K
4660 K
4665 K
4670 K
4675 K
4680 K
4685 K
4690 K
4695 K
4700 K
SE15_1st
SE15_2nd
Overlap
3
2
1
0
-1
4270
4271
4269
4273
4272
4274
4275
4276
4277
4278
4279
4280
4288
4286
4287
4289
4290
4291
4292
4293
4294
4295
4296
4297
4298
4299
4300
4301
4306
4307
4305
4309
4310
4308
4311
4312
4313
4315
4314
4316
4317
4318
4319
4700 K
4705 K
4710 K
4715 K
4720 K
4725 K
4730 K
4735 K
4740 K
4745 K
4750 K
SE15_1st
SE15_2nd
Overlap
4321
4322
4323
4324
4325
4326
4327
4330
4331
4329
4333
4334
4332
4335
4336
4337
4338
4750 K
4755 K
4760 K
4765 K
4770 K
4775 K
4780 K
4785 K
4790 K
4795 K
4800 K
SE15_1st
SE15_2nd
Overlap

H-NS binding profiles in *Escherichia coli* K-12 (W3110)

(1st experiment and 2nd experiment)

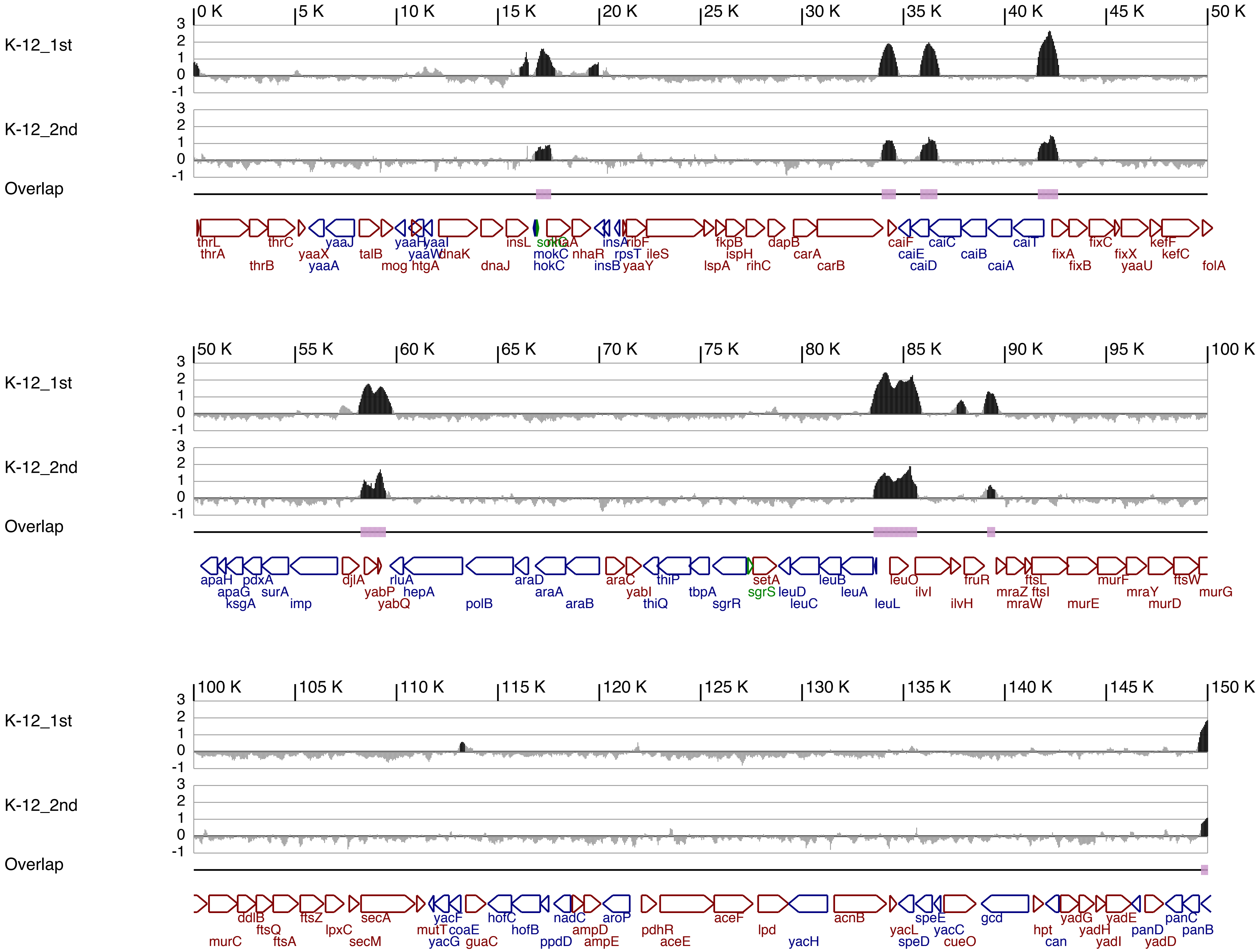
K-12_1st
K-12_2nd
Overlap
0 K
5 K
10 K
15 K
20 K
25 K
30 K
35 K
40 K
45 K
50 K
thrL thrA thrB thrC yaaX yaaA yaaJ talB mog htgA yaaW dnaK dnaJ insL mokC hokC nhaR insB rpsT ribF ileS yaaY lspA fkpB ispH rihC dapB carA carB caiF caiE caiD caiC caiB caiA caiT fixA fixB fixC fixX yaaU kefF kefC folA
55 K
60 K
65 K
70 K
75 K
80 K
85 K
90 K
95 K
100 K
apaH apaG ksgA pdxA surA imp djlA yabP yabQ rluA hepA polB araD araA araB araC yabI thiP thiQ tbpA sgrR setA sgrS leuD leuC leuB leuA leuL leuO ilvI ilvH fruR mraZ mraW ftsL ftsI murE murF mraY murD ftsW murG
105 K
110 K
115 K
120 K
125 K
130 K
135 K
140 K
145 K
150 K
murC ddlB ftsQ ftsA ftsZ lpxC secA secM yacF mutT yacG coaE guaC hofC hofB nadC ppdD ampD ampE aroP pdhR aceE aceF lpd yacH acnB yacL speD speE yacC cueO gcd hpt can yadG yadH yadI yadE panD yadD panC panB
3
2
1
0
-1

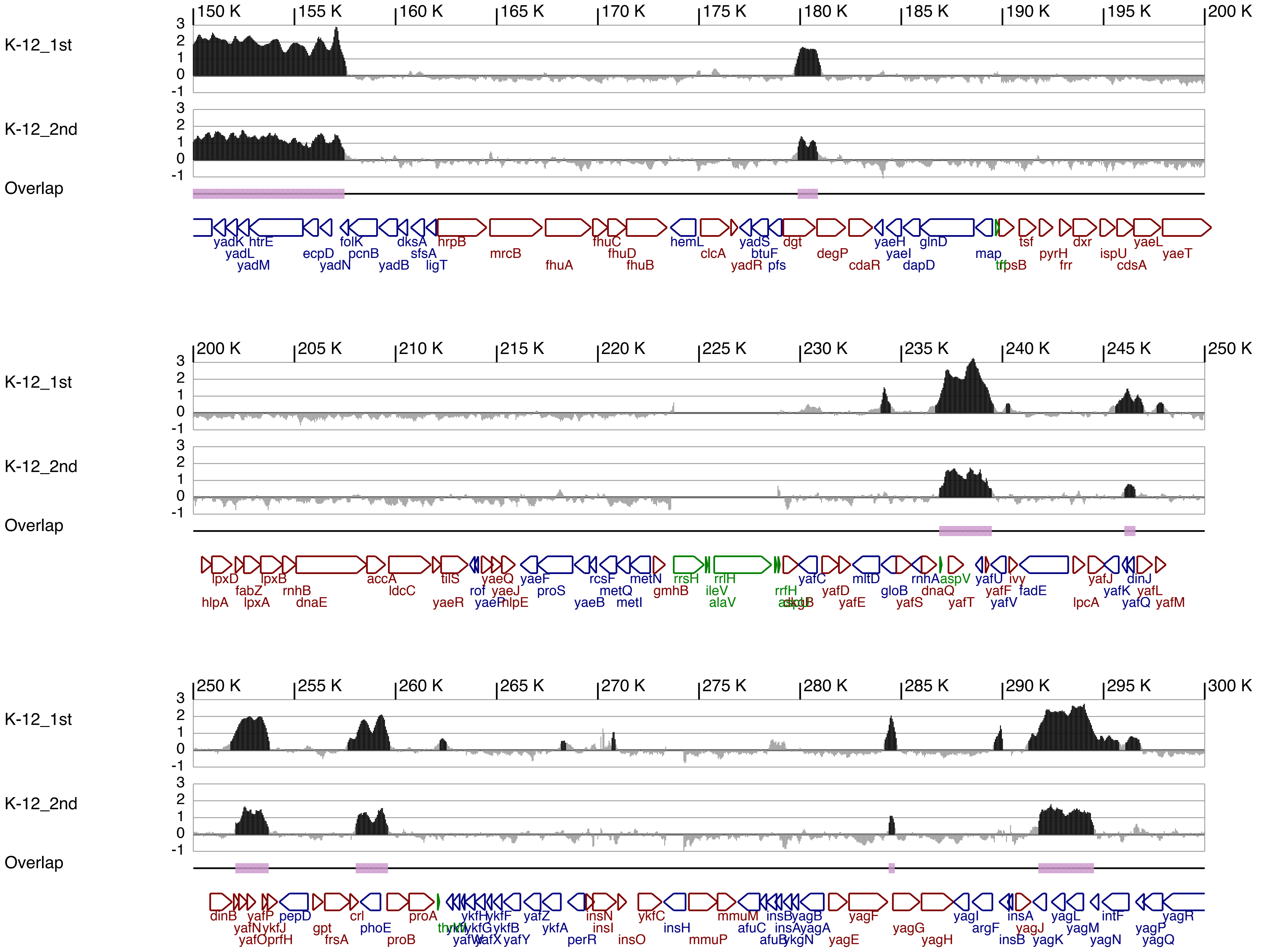
150 K 155 K 160 K 165 K 170 K 175 K 180 K 185 K 190 K 195 K 200 K
K-12_1st
K-12_2nd
Overlap
3 2 1 0 -1
yadK htrE yadL yadM ecpD yadN folK pcnB yadB dksA sfsA ligT hrpB mrcB fhuA fhuC fhuD fhuB hemL clcA yadS btuF yadR pfs dgt degP cdaR yaeH yaeI glnD dapD map tsf pyrH frr dxr ispU cdsA yaeL yaeT
200 K 205 K 210 K 215 K 220 K 225 K 230 K 235 K 240 K 245 K 250 K
K-12_1st
K-12_2nd
Overlap
lpxD fabZ lpxB hlpA lpxA rnhB dnaE accA ldcC tilS yaeR rof yaeQ yaeJ yaeF proS rcsF metQ yaeB metI metN gmhB rrsH ileV alaV rrlH rrfH yafC yafD yafE mltD gloB yafS rnhA dnaQ aspV yafT yafU yafF yafV ivy fadE yafJ lpcA yafK dinJ yafL yafQ yafM
250 K 255 K 260 K 265 K 270 K 275 K 280 K 285 K 290 K 295 K 300 K
K-12_1st
K-12_2nd
Overlap
dinB yafP yafN ykfJ yafO prfH pepD gpt frsA crl phoE proA proB ykfH ykfF ykfG ykfB yafW yafX yafY yafZ ykfA insN insI perR insO ykfC insH mmuM mmuP afuC insB yagB insA yagA afuB ykgN yagF yagE yagG yagH yagI argF insA insB yagJ yagK yagL yagM yagN intF yagP yagQ yagR

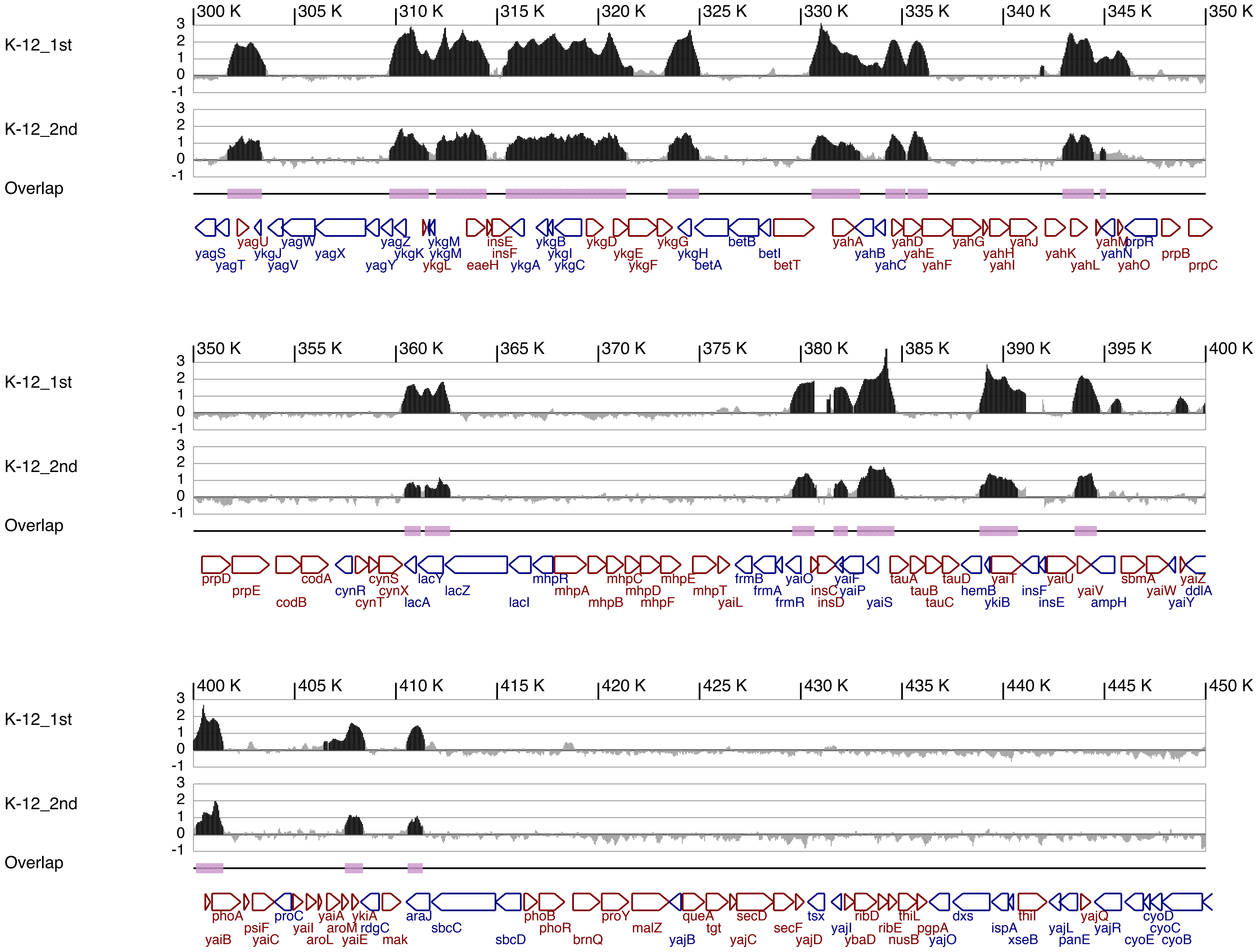

300 K
305 K
310 K
315 K
320 K
325 K
330 K
335 K
340 K
345 K
350 K
K-12_1st
K-12_2nd
Overlap
yagS yagT yagU ykgJ yagV yagW yagX yagY yagZ ykgK ykgL ykgM ykgM insE insF eaeH ykgA ykgB ykgI ykgC ykgD ykgE ykgF ykgG ykgH betA betB betI betT yahA yahB yahC yahD yahE yahF yahG yahH yahI yahJ yahK yahL yahM yahN prpR yahO prpB prpC
355 K
360 K
365 K
370 K
375 K
380 K
385 K
390 K
395 K
400 K
prpD prpE codA codB cynR cynS cynX cynT lacY lacA lacZ lacI mhpR mhpA mhpC mhpB mhpD mhpE mhpF mhpT yaiL frmB frmA frmR yaiO insC insD yaiF yaiP yaiS tauA tauB tauC tauD hemB ykiB yaiT insF insE yaiU yaiV ampH sbmA yaiW yaiY yaiZ ddlA
405 K
410 K
415 K
420 K
425 K
430 K
435 K
440 K
445 K
450 K
phoA yaiB psiF yaiC proC yaiI aroL yaiA aroM yaiE ykiA rdgC mak araJ sbcC sbcD phoB phoR proY brnQ malZ queA yajB tgt secD yajC secF yajD tsx yajI ribD ybaD ribE nusB thiL pgpA yajO dxs ispA xseB thiI yajL panE yajQ yajR cyoD cyoC cyoE cyoB

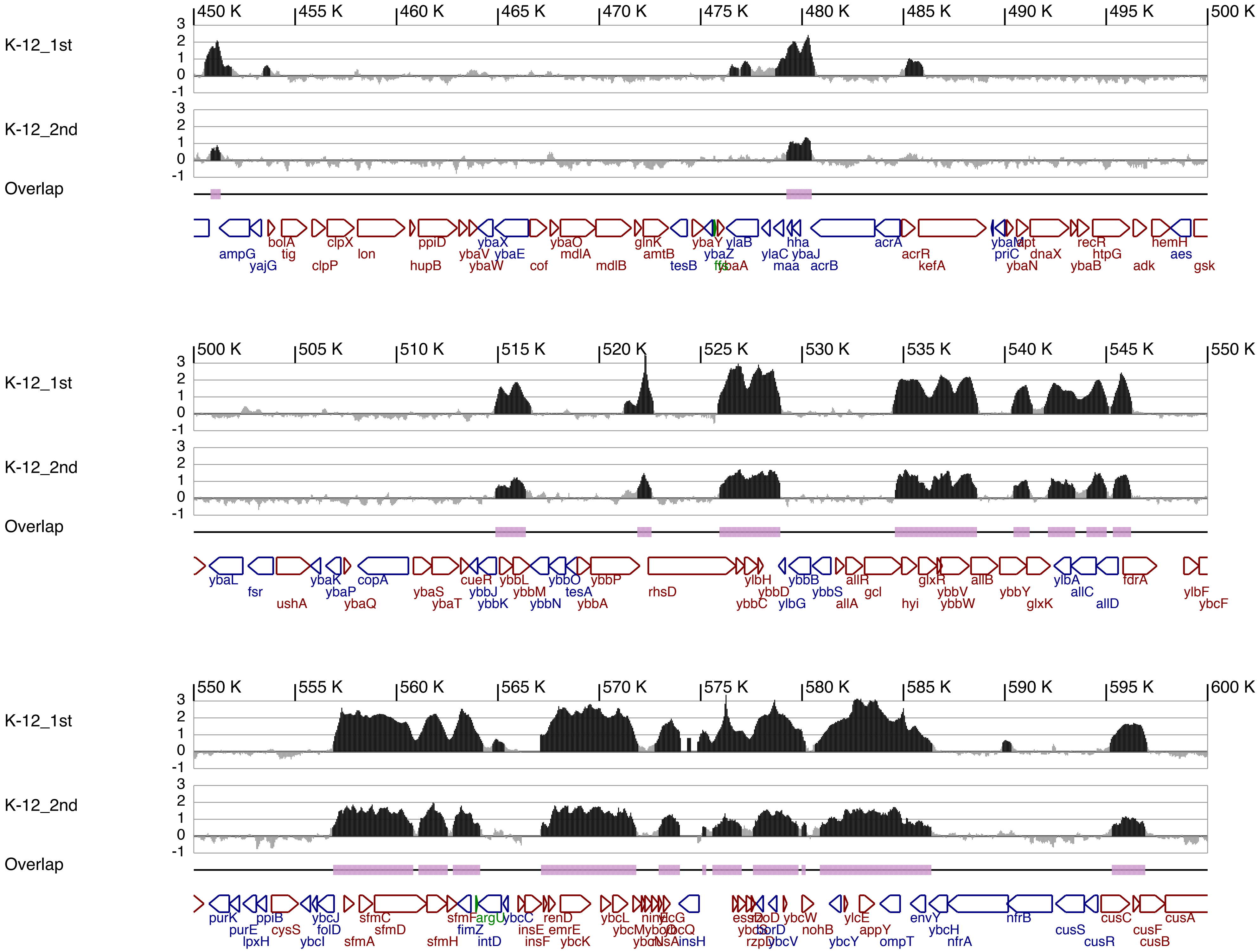

450 K
455 K
460 K
465 K
470 K
475 K
480 K
485 K
490 K
495 K
500 K
K-12_1st
K-12_2nd
Overlap
ampG
yajG
bolA
tig
clpX
clpP
lon
ppiD
hupB
ybaV
ybaW
ybaX
ybaE
cof
ybaO
mdlA
mdlB
glnK
amtB
tesB
ybaY
ybaZ
ylaB
ylaC
ybaJ
hha
maa
acrB
acrA
acrR
kefA
priC
ybaN
dnaX
recR
htpG
ybaB
adk
hemH
aes
gsk
505 K
510 K
515 K
520 K
525 K
530 K
535 K
540 K
545 K
550 K
ybaL
fsr
ushA
ybaK
ybaP
ybaQ
copA
ybaS
ybaT
cueR
ybbJ
ybbK
ybbL
ybbM
ybbN
ybbO
tesA
ybbA
ybbP
rhsD
ylbH
ybbD
ybbC
ylbG
ybbB
ybbS
allR
allA
gcl
glxR
hyi
ybbV
ybbW
allB
ybbY
glxK
ylbA
allC
allD
fdrA
ylbF
ybcF
555 K
560 K
565 K
570 K
575 K
580 K
585 K
590 K
595 K
600 K
purK
purE
lpxH
ppiB
cysS
ybcI
ybcJ
folD
sfmC
sfmA
sfmD
sfmH
fimZ
intD
ybcC
insE
insF
renD
emrE
ybcK
ybcL
ybcW
nohB
ylcE
appY
ompT
ybcY
envY
ybcH
nfrA
nfrB
cusS
cusR
cusC
cusF
cusB
cusA

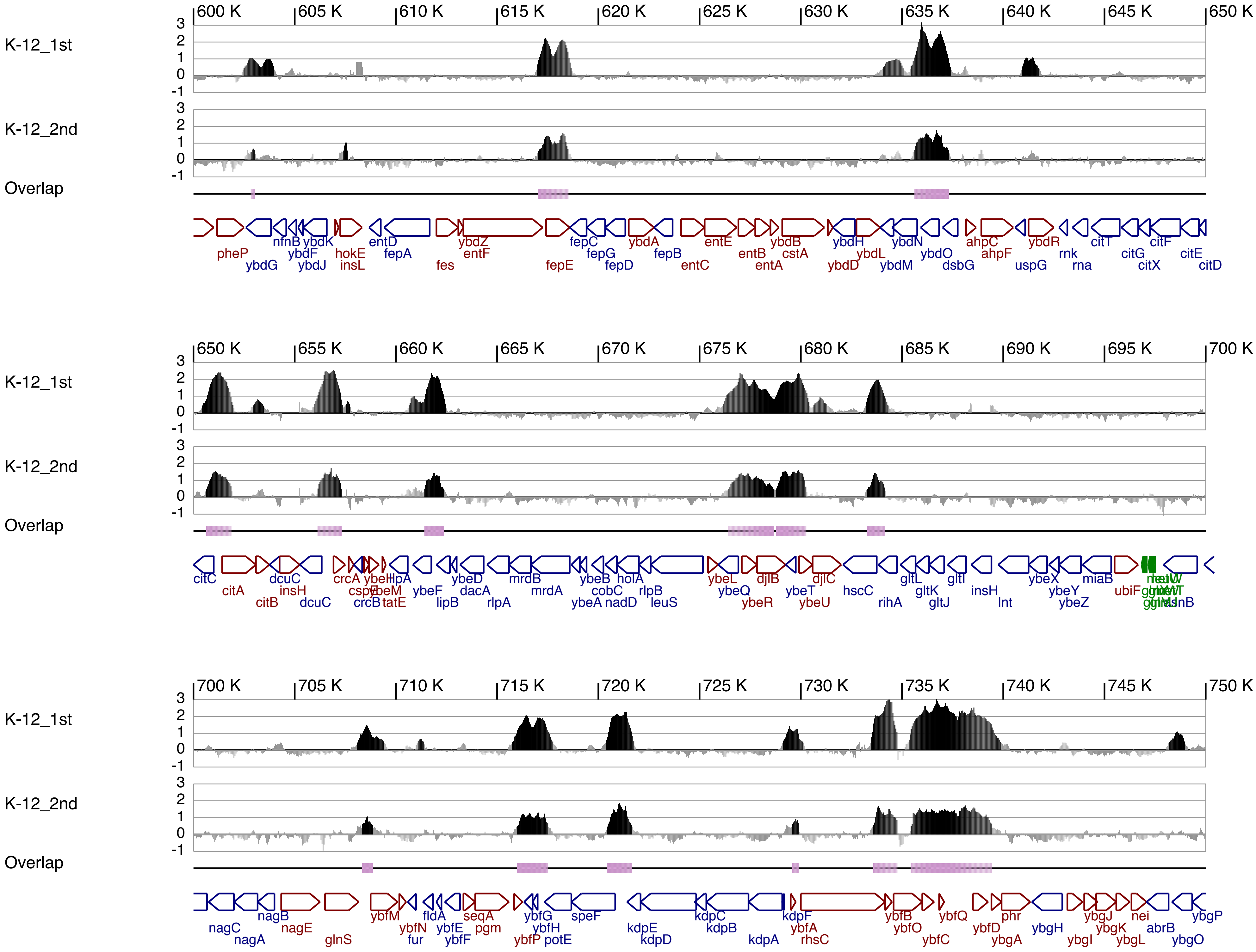

600 K
605 K
610 K
615 K
620 K
625 K
630 K
635 K
640 K
645 K
650 K
K-12_1st
K-12_2nd
Overlap
3
2
1
0
-1
nfnB ybdK
pheP
ybdF
ybdG
ybdJ
hokE
insL
entD
fepA
fes
ybdZ
entF
fepE
fepC
fepG
fepD
ybdA
fepB
entE
entC
entB
entA
ybdB
cstA
ybdH
ybdD
ybdL
ybdM
ybdN
ybdO
dsbG
ahpC
ahpF
uspG
ybdR
rnk
rna
citT
citG
citX
citF
citE
citD
650 K
655 K
660 K
665 K
670 K
675 K
680 K
685 K
690 K
695 K
700 K
citC
citA
citB
dcuC
insH
dcuC
crcA
crcB
tatE
ybeF
lipB
ybeD
dacA
rlpA
mrdB
mrdA
ybeB
ybeA
cobC
nadD
holA
rlpB
leuS
ybeL
ybeQ
ybeR
djlB
ybeT
ybeU
djlC
hscC
rihA
gltL
gltK
gltJ
gltI
insH
Int
ybeX
ybeY
ybeZ
miaB
ubiF
700 K
705 K
710 K
715 K
720 K
725 K
730 K
735 K
740 K
745 K
750 K
nagC
nagA
nagB
nagE
glnS
ybfM
ybfN
fur
fldA
ybfE
ybfF
seqA
pgm
ybfG
ybfH
ybfP
potE
speF
kdpE
kdpD
kdpC
kdpB
kdpA
kdpF
ybfA
rhsC
ybfB
ybfO
ybfC
ybfQ
ybfD
phr
ybgA
ybgH
ybgJ
ybgI
ybgK
ybgL
nei
abrB
ybgO
ybgP

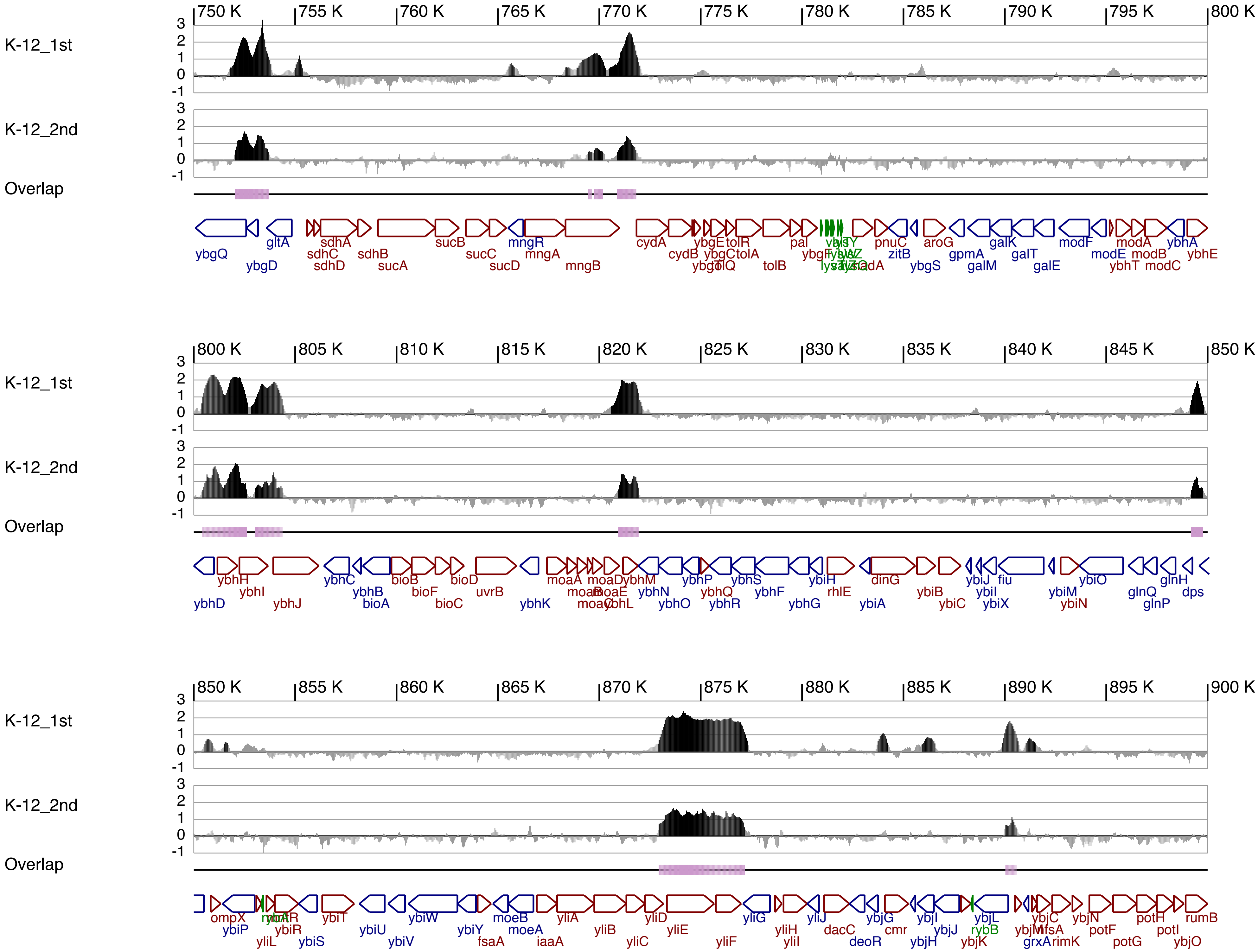
750 K
755 K
760 K
765 K
770 K
775 K
780 K
785 K
790 K
795 K
800 K
K-12_1st
K-12_2nd
Overlap
3
2
1
0
-1
ybgQ
gltA
ybgD
sdhA
sdhC
sdhD
sdhB
sucA
sucB
sucC
sucD
mngR
mngA
mngB
cydA
cydB
ybgE
tolR
tolA
tolB
pal
pnuC
zitB
aroG
ybgS
gpmA
galM
galK
galT
galE
modF
modE
modA
modB
modC
ybhT
ybhA
ybhE
805 K
810 K
815 K
820 K
825 K
830 K
835 K
840 K
845 K
850 K
ybhD
ybhH
ybhI
ybhJ
ybhC
ybhB
bioA
bioB
bioF
bioC
bioD
uvrB
ybhK
moaA
moaD
ybhM
moaE
ybhL
ybhN
ybhO
ybhP
ybhQ
ybhR
ybhS
ybhF
ybhG
ybiH
rhlE
ybiA
dinG
ybiB
ybiC
ybiJ
fiu
ybiI
ybiX
ybiM
ybiN
ybiO
glnQ
glnP
glnH
dps
855 K
860 K
865 K
870 K
875 K
880 K
885 K
890 K
895 K
900 K
ompX
ybiP
yliL
ybiR
ybiS
ybiT
ybiU
ybiV
ybiW
ybiY
fsaA
moeB
moeA
iaaA
yliA
yliB
yliC
yliD
yliE
yliF
yliG
yliH
yliI
yliJ
dacC
deoR
ybjG
cmr
ybjI
ybjJ
ybjH
ybjL
rybB
ybjK
ybjC
grxA
rimK
ybjN
potF
potG
potH
potI
rumB
ybjO

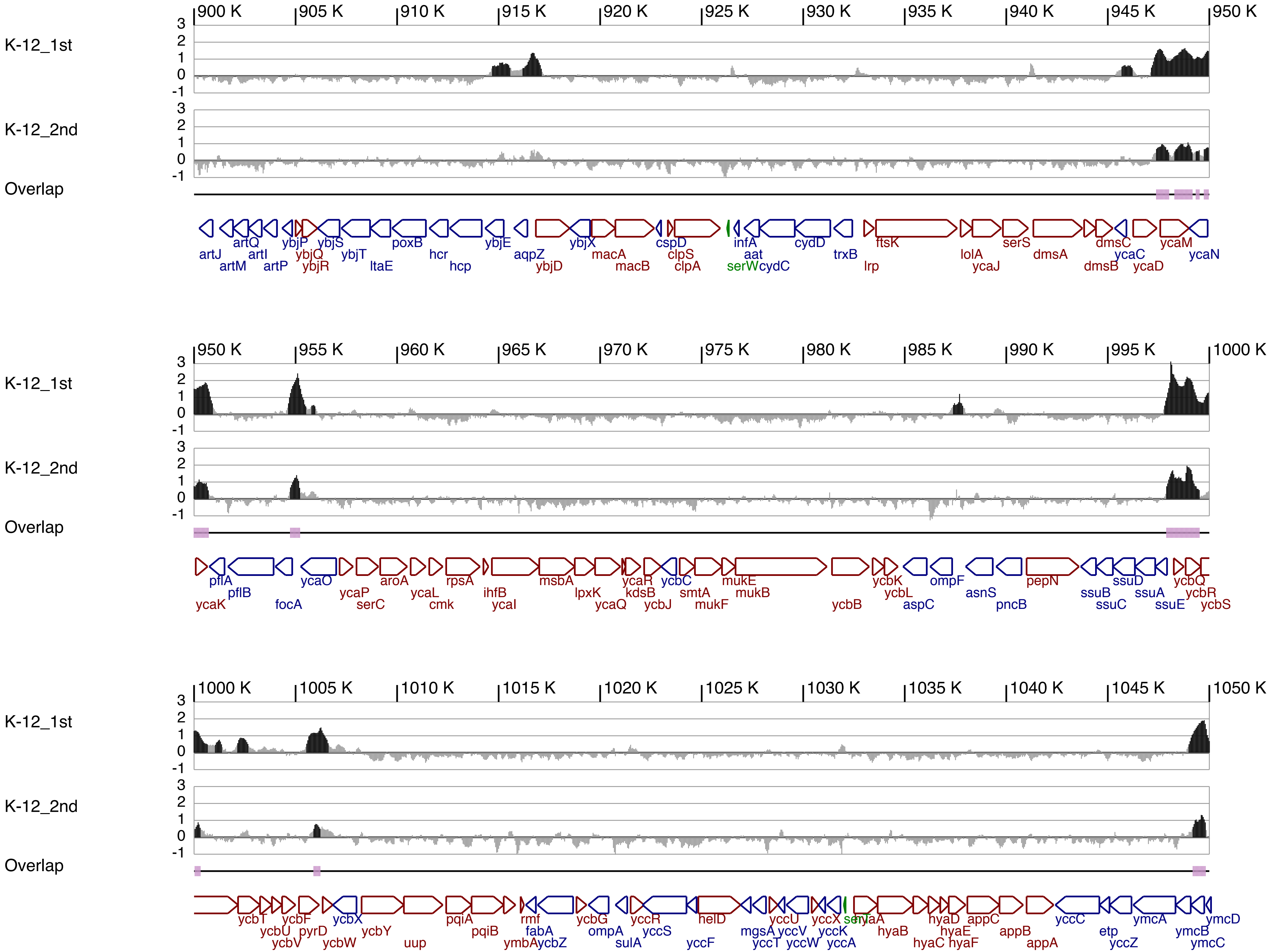

900 K
905 K
910 K
915 K
920 K
925 K
930 K
935 K
940 K
945 K
950 K
K-12_1st
K-12_2nd
Overlap
3
2
1
0
-1
artJ artM artQ artI artP ybjP ybjQ ybjR ybjS ybjT ltaE poxB hcr hcp ybjE aqpZ ybjD ybjX macA macB cspD clpS clpA infA aat serW cydC cydD trxB lrp ftsK lolA ycaJ serS dmsA dmsC dmsB ycaC ycaD ycaM ycaN
950 K
955 K
960 K
965 K
970 K
975 K
980 K
985 K
990 K
995 K
1000 K
ycaK pflA pflB focA ycaO ycaP serC aroA ycaL cmk rpsA ihfB ycaI msbA lpxK ycaQ ycaR kdsB ycbJ ycbC smtA mukF mukE mukB ycbB ycbK ycbL aspC ompF asnS pncB pepN ssuB ssuC ssuD ssuA ssuE ycbQ ycbR ycbS
1000 K
1005 K
1010 K
1015 K
1020 K
1025 K
1030 K
1035 K
1040 K
1045 K
1050 K
ycbT ycbU ycbV ycbF pyrD ycbW ycbX ycbY uup pqiA pqiB rmf ymbA fabA ycbZ ycbG ompA sulA yccR yccS helD yccF mgsA yccT yccU yccV yccW yccX yccK yccA hyaA hyaB hyaC hyaD hyaE hyaF appC appB appA yccC etp yccZ ymcA ymcB ymcC ymcD

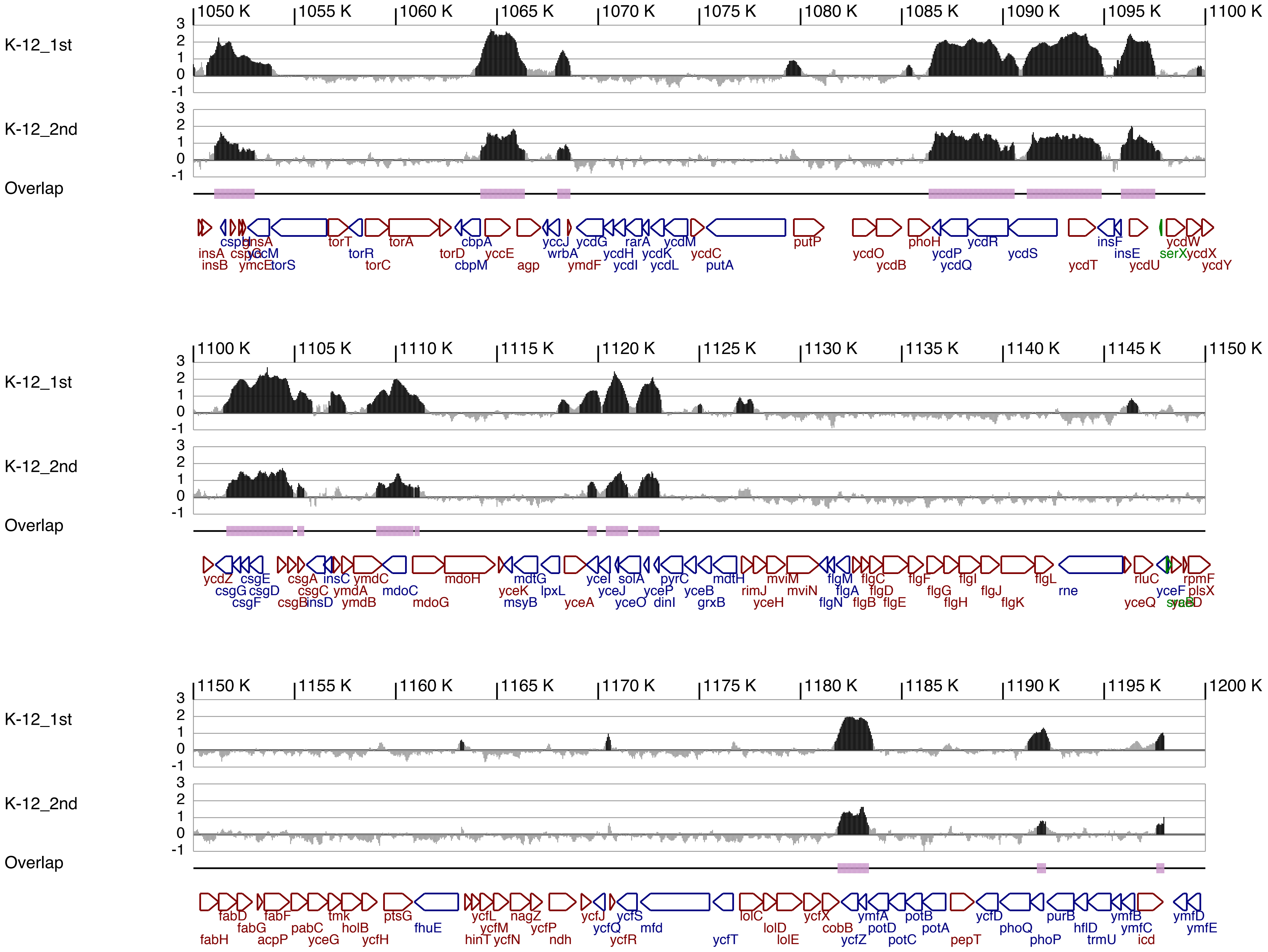

1050 K
1055 K
1060 K
1065 K
1070 K
1075 K
1080 K
1085 K
1090 K
1095 K
1100 K
K-12_1st
K-12_2nd
Overlap
3
2
1
0
-1
insA
insB
torT
torR
torC
torA
torD
cbpA
cbpM
yccE
agp
yccJ
wrbA
ymdF
ycdG
ycdH
ycdI
rarA
ycdK
ycdL
ycdM
ycdC
putA
putP
ycdO
ycdB
phoH
ycdP
ycdQ
ycdR
ycdS
ycdT
insF
insE
ycdU
serX
ycdW
ycdX
ycdY
1105 K
1110 K
1115 K
1120 K
1125 K
1130 K
1135 K
1140 K
1145 K
1150 K
ycdZ
csgG
csgF
csgE
csgD
csgA
csgC
csgB
insC
insD
ymdA
ymdB
ymdC
mdoC
mdoH
mdoG
yceK
msyB
mdtG
lpxL
yceA
yceI
yceJ
yceO
solA
yceP
dinI
pyrC
yceB
grxB
mdtH
rimJ
yceH
mviM
mviN
flgM
flgN
flgA
flgB
flgC
flgD
flgE
flgF
flgG
flgH
flgI
flgJ
flgK
flgL
rne
yceQ
rluC
yceF
rpmF
plsX
1155 K
1160 K
1165 K
1170 K
1175 K
1180 K
1185 K
1190 K
1195 K
1200 K
fabH
fabD
fabG
acpP
fabF
pabC
yceG
tmk
holB
ycfH
ptsG
fhuE
hinT
ycfL
ycfM
ycfN
nagZ
ycfP
ndh
ycfJ
ycfQ
ycfR
ycfS
mfd
ycfT
lolC
lolD
lolE
ycfX
cobB
ycfZ
ymfA
potD
potC
potB
potA
pepT
ycfD
phoQ
phoP
purB
hflD
trmU
ymfB
ymfC
icd
ymfD
ymfE

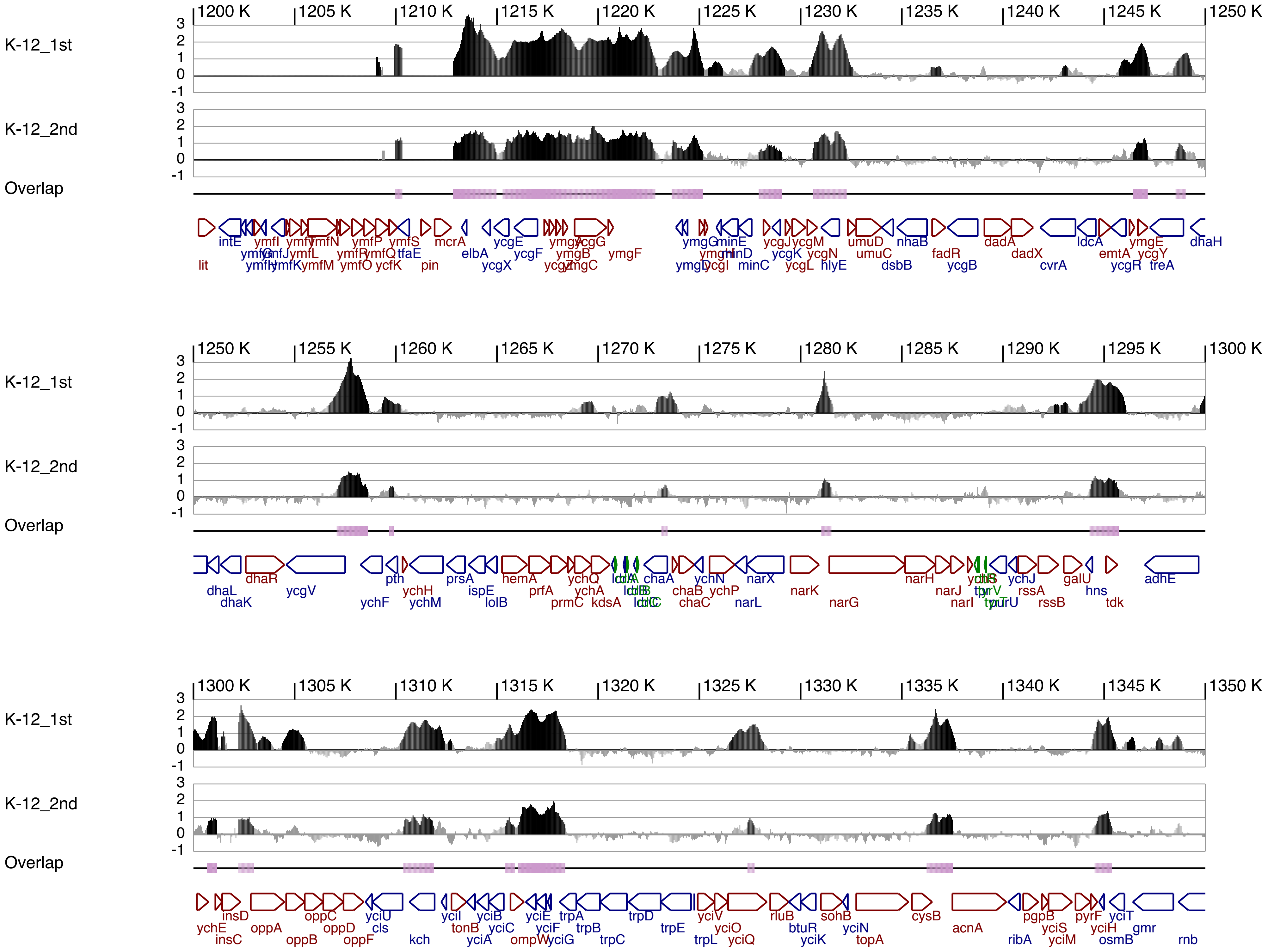

1200 K
1205 K
1210 K
1215 K
1220 K
1225 K
1230 K
1235 K
1240 K
1245 K
1250 K
K-12_1st
K-12_2nd
Overlap
3
2
1
0
-1
lit
intE
ymfI
ymfN
ymfP
ymfS
tfaE
ycfK
pin
mcrA
elbA
ycgX
ycgE
ycgF
ymgB
ycgG
ymgF
ymgG
minE
minD
minC
ycgJ
ycgM
ycgK
ycgN
ycgL
hlyE
umuD
umuC
nhaB
dsbB
fadR
ycgB
dadA
dadX
cvrA
ldcA
emtA
ymgE
ycgY
ycgR
treA
dhaH
1255 K
1260 K
1265 K
1270 K
1275 K
1280 K
1285 K
1290 K
1295 K
1300 K
dhaL
dhaK
dhaR
ycgV
ychF
pth
ychH
ychM
prsA
ispE
lolB
hemA
prfA
prmC
ychQ
ychA
kdsA
chaA
chaB
chaC
ychN
ychP
narX
narL
narK
narG
narH
narJ
narI
ychJ
rssA
rssB
galU
hns
tdk
adhE
1305 K
1310 K
1315 K
1320 K
1325 K
1330 K
1335 K
1340 K
1345 K
1350 K
ychE
insD
insC
oppA
oppB
oppC
oppD
oppF
yciU
cls
kch
yciI
tonB
yciA
yciB
yciC
ompW
yciE
yciF
yciG
trpA
trpB
trpC
trpD
trpE
yciV
trpL
yciO
yciQ
rluB
btuR
yciK
sohB
yciN
topA
cysB
acnA
ribA
pgpB
yciS
yciM
pyrF
yciH
osmB
yciT
gmr
rnb

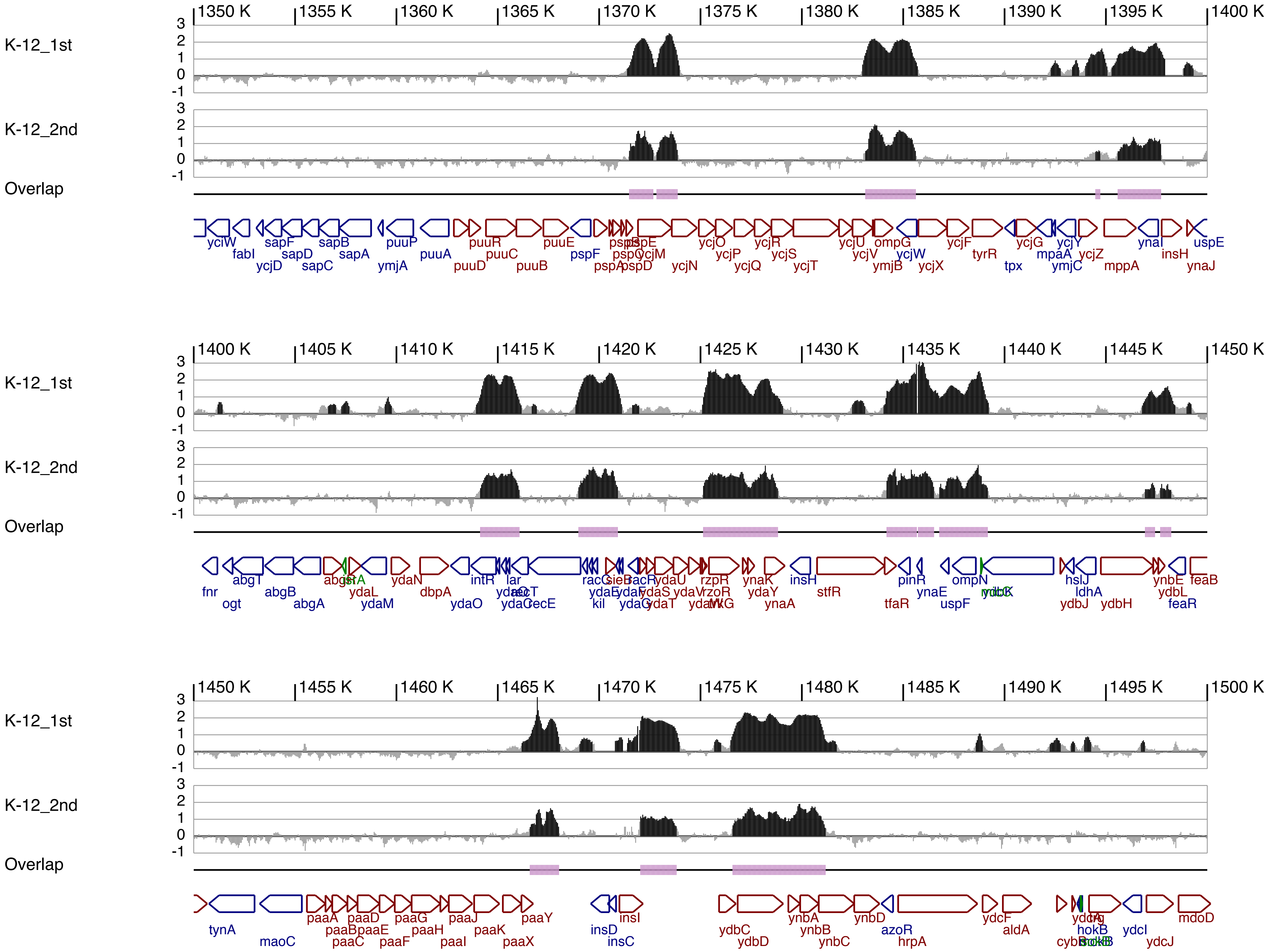

1350 K 1355 K 1360 K 1365 K 1370 K 1375 K 1380 K 1385 K 1390 K 1395 K 1400 K
K-12_1st
K-12_2nd
Overlap
yciW fabI ycjD sapF sapD sapC sapB sapA ymjA puuP puuA puuR puuD puuC puuB puuE pspF pspA pspD pspE ycjM ycjN ycjO ycjP ycjQ ycjR ycjS ycjT ycjU ycjV ompG ymjB ycjW ycjX ycjF tyrR tpx ycjG mpaA ymjC ycjY ycjZ mppA ynaI insH ynaJ uspE
1400 K 1405 K 1410 K 1415 K 1420 K 1425 K 1430 K 1435 K 1440 K 1445 K 1450 K
K-12_1st
K-12_2nd
Overlap
fnr ogt abgT abgB abgA abgR ydaL ydaM ydaN dbpA ydaO intR lar recT recE racC kil sieB racR ydaU ydaS ydaT rzpR rzoR ynaK ydaY ynaA insH stfR tfaR pinR ynaE uspF ompN hslJ ldhA ydbJ ydbH ynbE ydbL feaB feaR
1450 K 1455 K 1460 K 1465 K 1470 K 1475 K 1480 K 1485 K 1490 K 1495 K 1500 K
K-12_1st
K-12_2nd
Overlap
tynA maoC paaA paaB paaC paaD paaE paaF paaG paaH paaI paaJ paaK paaX paaY insD insI insC ydbC ydbD ynbA ynbB ynbC ynbD azoR hrpA ydcF aldA hokB ydcI ydcJ mdoD

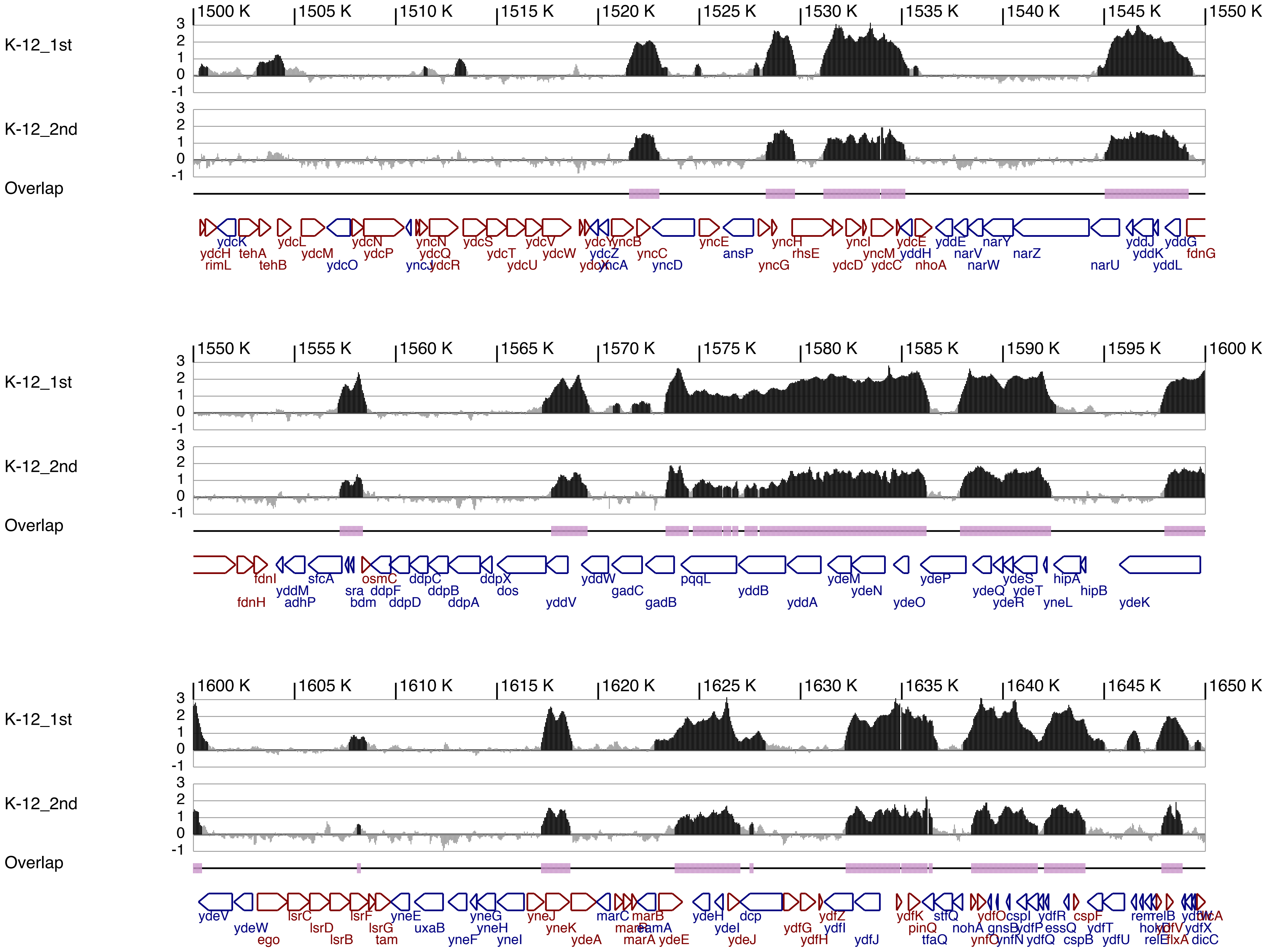

1500 K
1505 K
1510 K
1515 K
1520 K
1525 K
1530 K
1535 K
1540 K
1545 K
1550 K
3
2
1
0
-1
K-12_1st
K-12_2nd
Overlap
ydcK
ydcH
tehA
rimL
tehB
ydcL
ydcM
ydcO
ydcN
ydcP
yncN
ydcQ
ydcS
ydcT
ydcU
ydcV
ydcW
ydcZ
yncC
yncD
yncE
ansP
yncH
rhsE
yncG
yncI
yncM
ydcD
ydcC
ydcE
yddH
nhoA
yddE
narY
narV
narW
narZ
narU
yddJ
yddK
yddL
yddG
fdnG
1555 K
1560 K
1565 K
1570 K
1575 K
1580 K
1585 K
1590 K
1595 K
1600 K
fdnI
fdnH
yddM
adhP
sfcA
sra
bdm
osmC
ddpF
ddpD
ddpC
ddpB
ddpA
ddpX
dos
yddV
yddW
gadC
gadB
pqqL
yddB
yddA
ydeM
ydeN
ydeO
ydeP
ydeQ
ydeR
ydeS
ydeT
yneL
hipA
hipB
ydeK
1605 K
1610 K
1615 K
1620 K
1625 K
1630 K
1635 K
1640 K
1645 K
1650 K
ydeV
ydeW
ego
lsrC
lsrD
lsrB
lsrF
lsrG
tam
yneE
uxaB
yneF
yneG
yneH
yneI
yneJ
yneK
ydeA
marC
marB
marA
ydeE
ydeH
ydeI
ydeJ
dcp
ydfG
ydfH
ydfZ
ydfI
ydfJ
ydfK
pinQ
tfaQ
stfQ
nohA
cspI
ydfR
ydfQ
essQ
cspB
cspF
ydfT
ydfU
relB
dicC

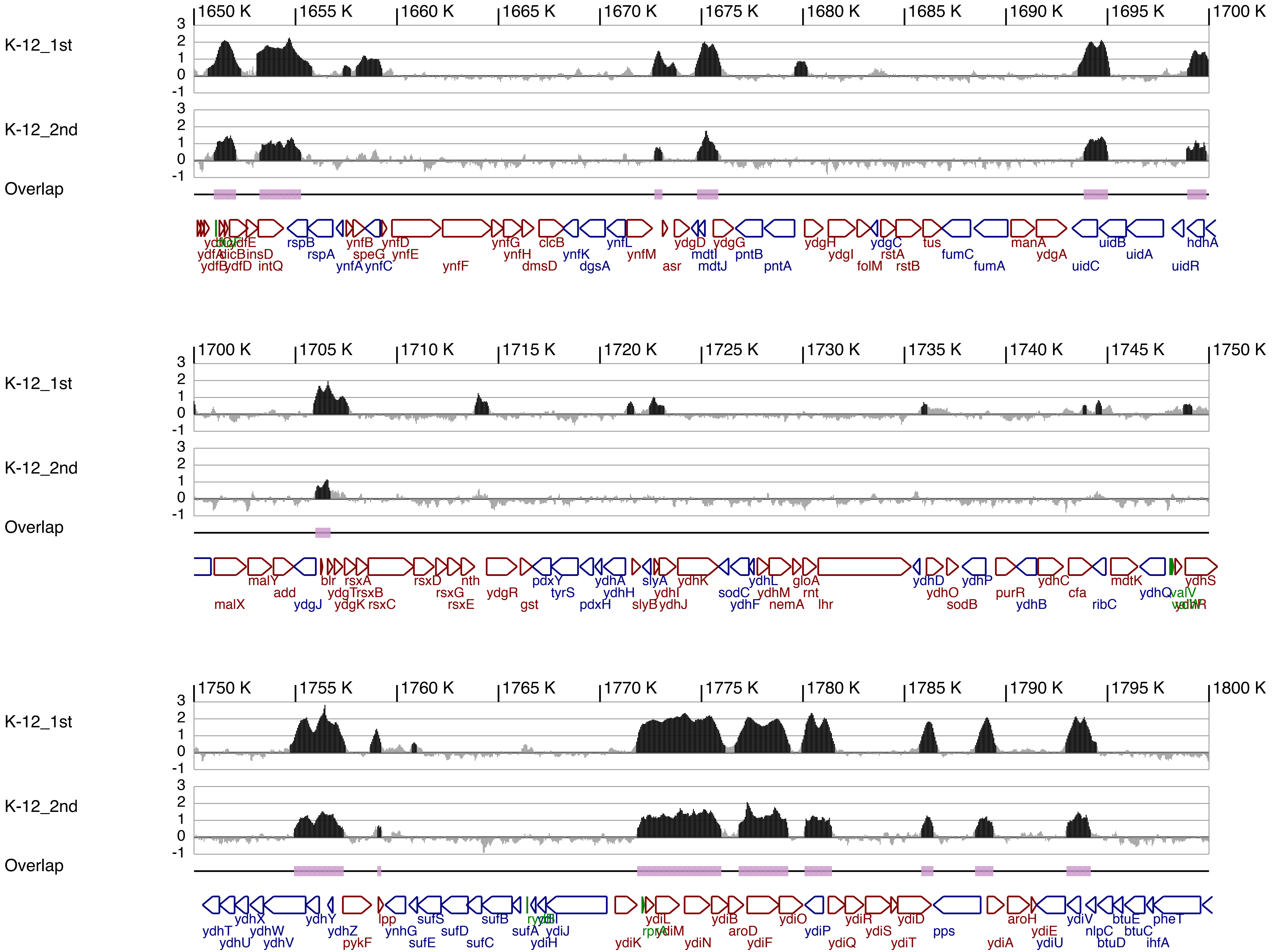

1650 K
1655 K
1660 K
1665 K
1670 K
1675 K
1680 K
1685 K
1690 K
1695 K
1700 K
K-12_1st
K-12_2nd
Overlap
ydfA dicB insD
ydfB ydfD intQ
rspB
rspA
ynfB ynfD
speG ynfE
ynfA ynfC
ynfF
ynfG
ynfH
clcB
dmsD
ynfK
dgsA
ynfL
ynfM
asr
ydgD ydgG
mdtI
mdtJ
pntB
pntA
ydgH
ydgI
folM
ydgC
rstA
rstB
tus
fumC
fumA
manA
ydgA
uidB
uidC
uidA
hdhA
uidR
1705 K
1710 K
1715 K
1720 K
1725 K
1730 K
1735 K
1740 K
1745 K
1750 K
malY
malX
add
ydgJ
blr rsxA
ydgT rsxB
ydgK rsxC
rsxD
rsxG
rsxE
nth
ydgR
gst
pdxY
tyrS
pdxH
ydhA
ydhH
slyA
ydhI
slyB ydhJ
ydhK
sodC
ydhF
ydhL
ydhM
nemA
gloA
rnt
lhr
ydhD
ydhO
sodB
ydhP
purR
ydhB
ydhC
cfa
ribC
mdtK
ydhQ
valV
ydhS
1755 K
1760 K
1765 K
1770 K
1775 K
1780 K
1785 K
1790 K
1795 K
1800 K
ydhX
ydhT
ydhU
ydhW
ydhV
ydhY
ydhZ
pykF
lpp
ynhG
sufS
sufE
sufD
sufC
sufB
sufA
ydiJ
ydiH
ydiK
ydiL
ydiM
ydiN
ydiB
aroD
ydiF
ydiO
ydiP
ydiQ
ydiR
ydiS
ydiT
ydiD
pps
ydiA
aroH
ydiE
ydiU
ydiV
nlpC
btuE
btuD
btuC
pheT
ihfA

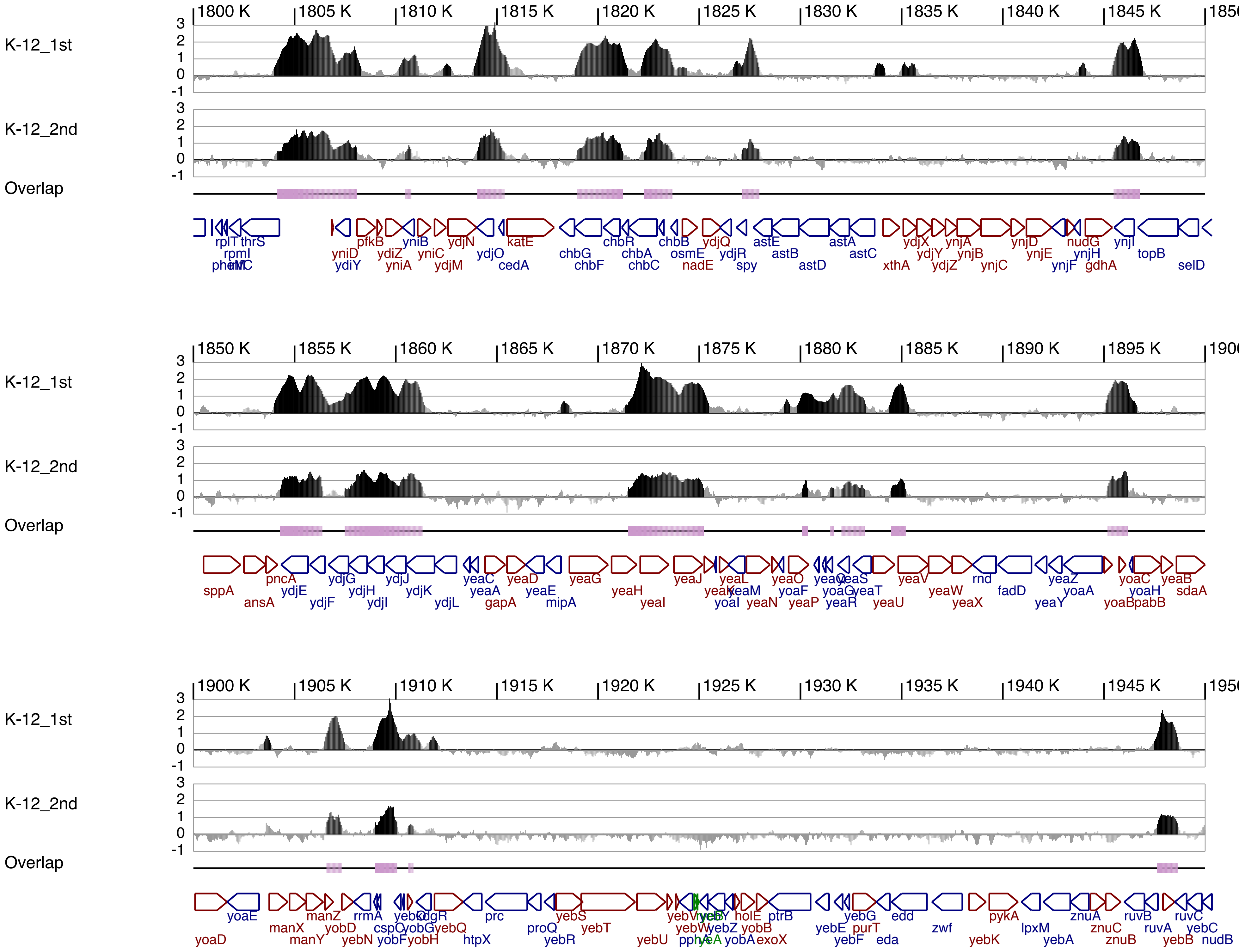

1800 K
1805 K
1810 K
1815 K
1820 K
1825 K
1830 K
1835 K
1840 K
1845 K
1850 K
K-12_1st
K-12_2nd
Overlap
3
2
1
0
-1
rplT thrS
rpmI
pheMC
yniD
ydiY
pfkB
ydiZ
yniA
yniB
yniC
ydjM
ydjN
ydjO
katE
cedA
chbG
chbF
chbR
chbA
chbC
chbB
osmE
nadE
ydjQ
ydjR
spy
astE
astB
astD
astA
astC
xthA
ydjX
ydjY
ydjZ
ynjA
ynjB
ynjC
ynjD
ynjE
ynjF
nudG
ynjH
gdhA
ynjI
topB
selD
1850 K
1855 K
1860 K
1865 K
1870 K
1875 K
1880 K
1885 K
1890 K
1895 K
1900 K
K-12_1st
K-12_2nd
Overlap
sppA
ansA
pncA
ydjE
ydjF
ydjG
ydjH
ydjI
ydjJ
ydjK
ydjL
yeaC
yeaA
gapA
yeaD
yeaE
mipA
yeaG
yeaH
yeaI
yeaJ
yeaL
yeaM
yeaN
yoaI
yeaO
yoaF
yeaP
yeaR
yeaT
yeaU
yeaV
yeaW
yeaX
rnd
fadD
yeaZ
yeaY
yoaA
yoaC
yoaH
yoaB
pabB
yeaB
sdaA
1900 K
1905 K
1910 K
1915 K
1920 K
1925 K
1930 K
1935 K
1940 K
1945 K
1950 K
K-12_1st
K-12_2nd
Overlap
yoaD
yoaE
manX
manY
manZ
yobD
yebN
rrmA
cspC
yobF
yobG
yobH
yebQ
htpX
prc
proQ
yebR
yebS
yebT
yebU
yebV
yebW
yebY
yebZ
yobB
yobA
holE
exoX
ptrB
yebE
yebF
yebG
purT
eda
edd
zwf
yebK
pykA
lpxM
yebA
znuA
znuC
znuB
ruvB
ruvA
yebB
ruvC
yebC
nudB

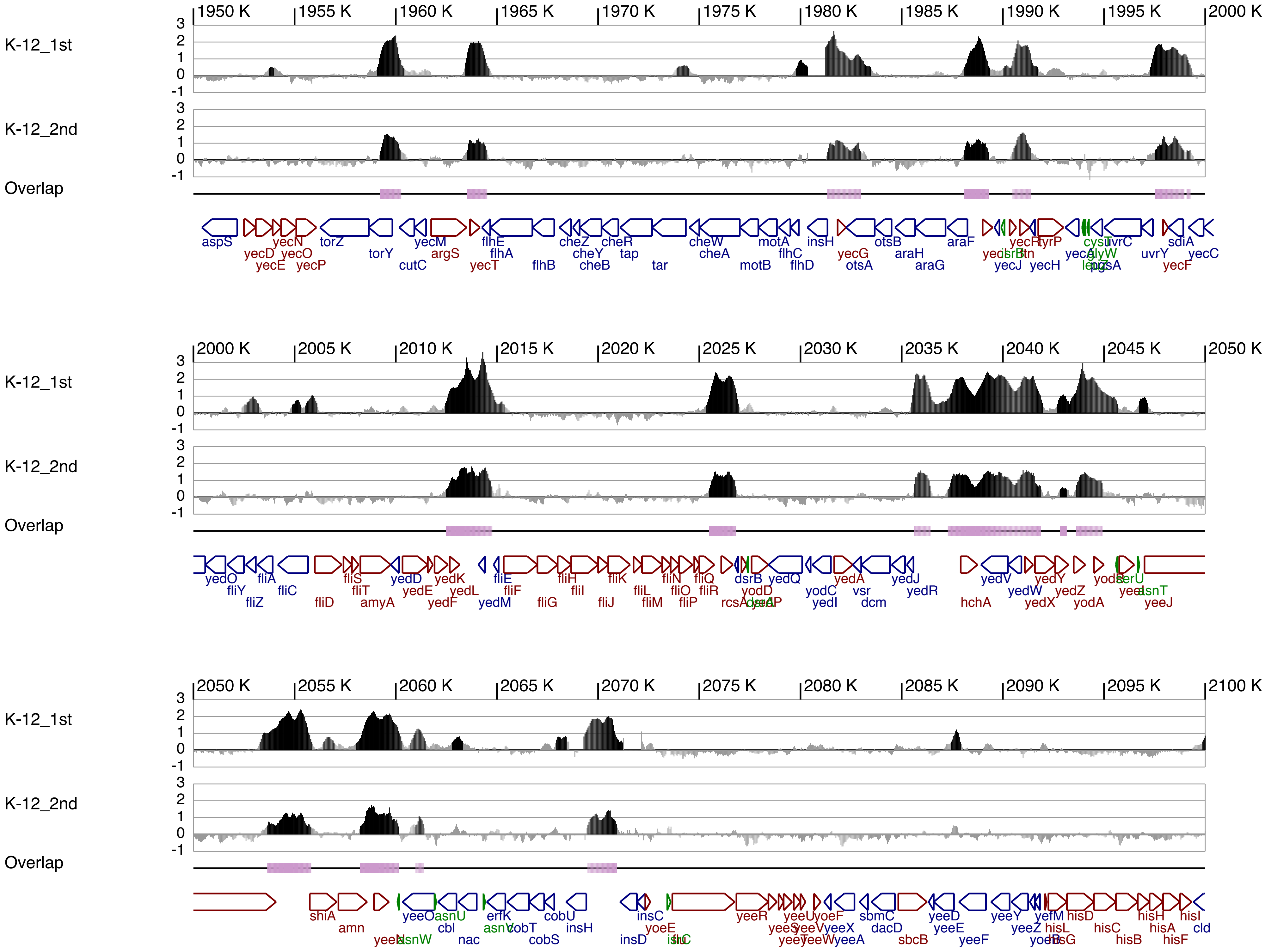
1950 K
1955 K
1960 K
1965 K
1970 K
1975 K
1980 K
1985 K
1990 K
1995 K
2000 K
K-12_1st
K-12_2nd
Overlap
3
2
1
0
-1
aspS
yecN
torZ
yecD
yecO
yecE
yecP
torY
cutC
yecM
argS
yecT
flhE
flhA
flhB
cheZ
cheR
cheY
tap
cheB
tar
cheW
cheA
motA
motB
flhC
flhD
insH
yecG
otsA
otsB
araH
araG
araF
yecJ
yecH
tyrP
uvrC
pgsA
uvrY
sdiA
yecC
yecF
2005 K
2010 K
2015 K
2020 K
2025 K
2030 K
2035 K
2040 K
2045 K
2050 K
yedO
fliY
fliZ
fliA
fliC
fliD
fliS
fliT
amyA
yedD
yedE
yedF
yedK
yedL
yedM
fliE
fliF
fliG
fliH
fliI
fliJ
fliK
fliL
fliM
fliN
fliO
fliP
fliQ
fliR
dsrB
yedQ
yodD
yodC
yedI
vsr
dcm
yedA
yedJ
yedR
hchA
yedV
yedW
yedX
yedY
yedZ
yodA
yeeJ
2055 K
2060 K
2065 K
2070 K
2075 K
2080 K
2085 K
2090 K
2095 K
2100 K
shiA
amn
cbl
nac
asnU
asnW
erfK
cobT
cobS
cobU
insH
insC
insD
yoeE
yeeR
yoeF
yeeX
yeeA
sbmC
dacD
sbcB
yeeD
yeeE
yeeF
yeeY
yeeZ
yefM
hisL
hisD
hisG
hisC
hisB
hisH
hisA
hisF
hisI
cld

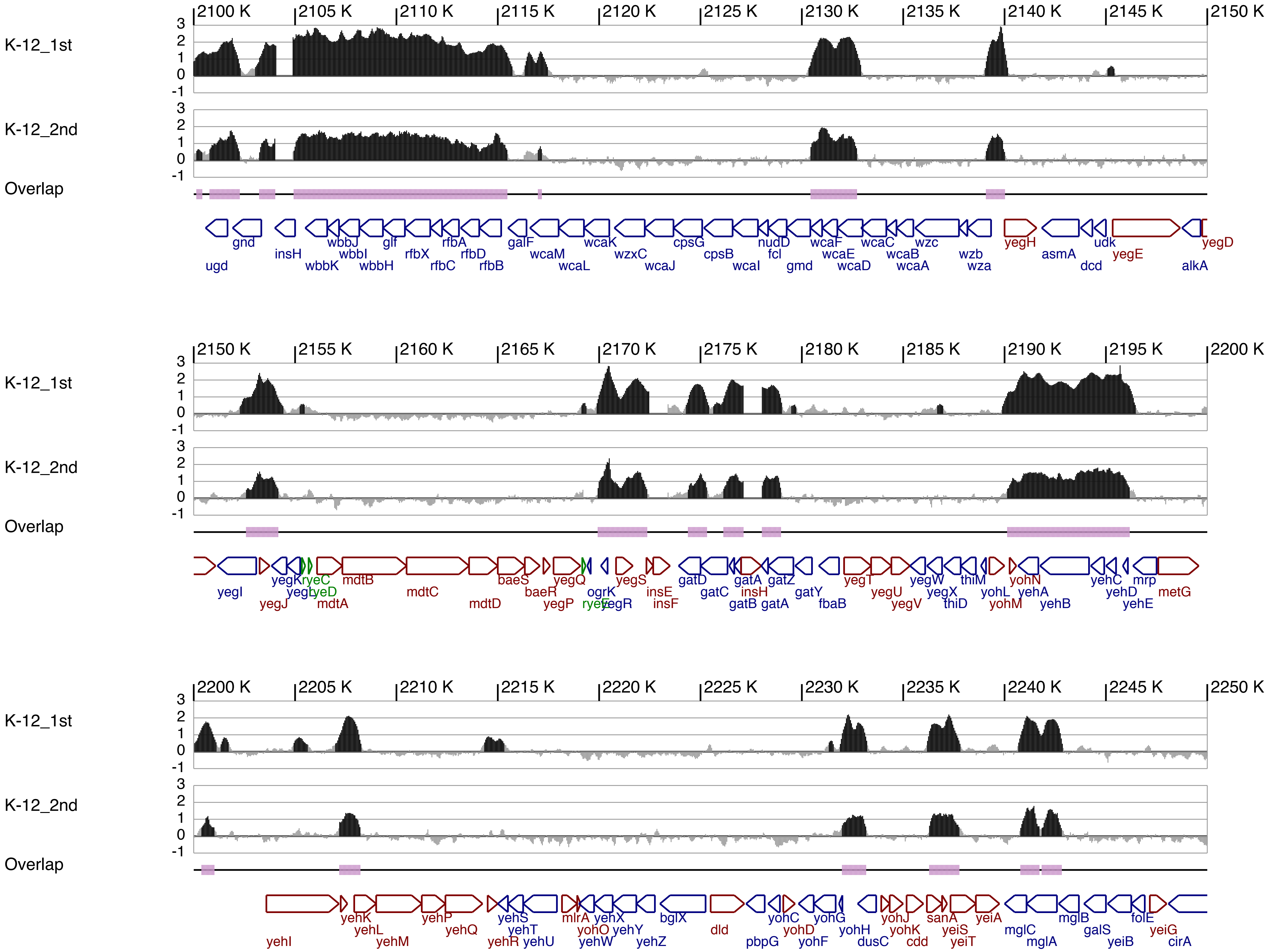

2100 K
2105 K
2110 K
2115 K
2120 K
2125 K
2130 K
2135 K
2140 K
2145 K
2150 K
K-12_1st
K-12_2nd
Overlap
3
2
1
0
-1
gnd
ugd
insH
wbbJ
wbbI
wbbK
wbbH
glf
rfbX
rfbA
rfbC
rfbD
rfbB
galF
wcaM
wcaK
wcaL
wzxC
wcaJ
cpsG
cpsB
wcaI
nudD
fcl
gmd
wcaF
wcaE
wcaD
wcaC
wcaB
wcaA
wzc
wzb
wza
yegH
asmA
udk
dcd
yegE
yegD
alkA
2155 K
2160 K
2165 K
2170 K
2175 K
2180 K
2185 K
2190 K
2195 K
2200 K
yegI
yegJ
yegK
yegL
ryeC
yeeD
mdtA
mdtB
mdtC
mdtD
baeS
baeR
yegP
yegQ
ogrK
yegS
yegR
insE
insF
gatD
gatC
gatB
gatA
insH
gatZ
gatA
gatY
fbaB
yegT
yegU
yegV
yegW
yegX
thiM
thiD
yohL
yohM
yohN
yehA
yehB
yehC
yehD
yehE
mrp
metG
2205 K
2210 K
2215 K
2220 K
2225 K
2230 K
2235 K
2240 K
2245 K
2250 K
yehI
yehK
yehL
yehM
yehP
yehQ
yehS
yehT
yehR
yehU
mlrA
yehX
yohO
yehY
yehW
yehZ
bglX
dld
pbpG
yohC
yohD
yohF
yohG
yohH
dusC
yohJ
yohK
cdd
sanA
yeiS
yeiT
yeiA
mglC
mglA
mglB
galS
yeiB
folE
yeiG
cirA

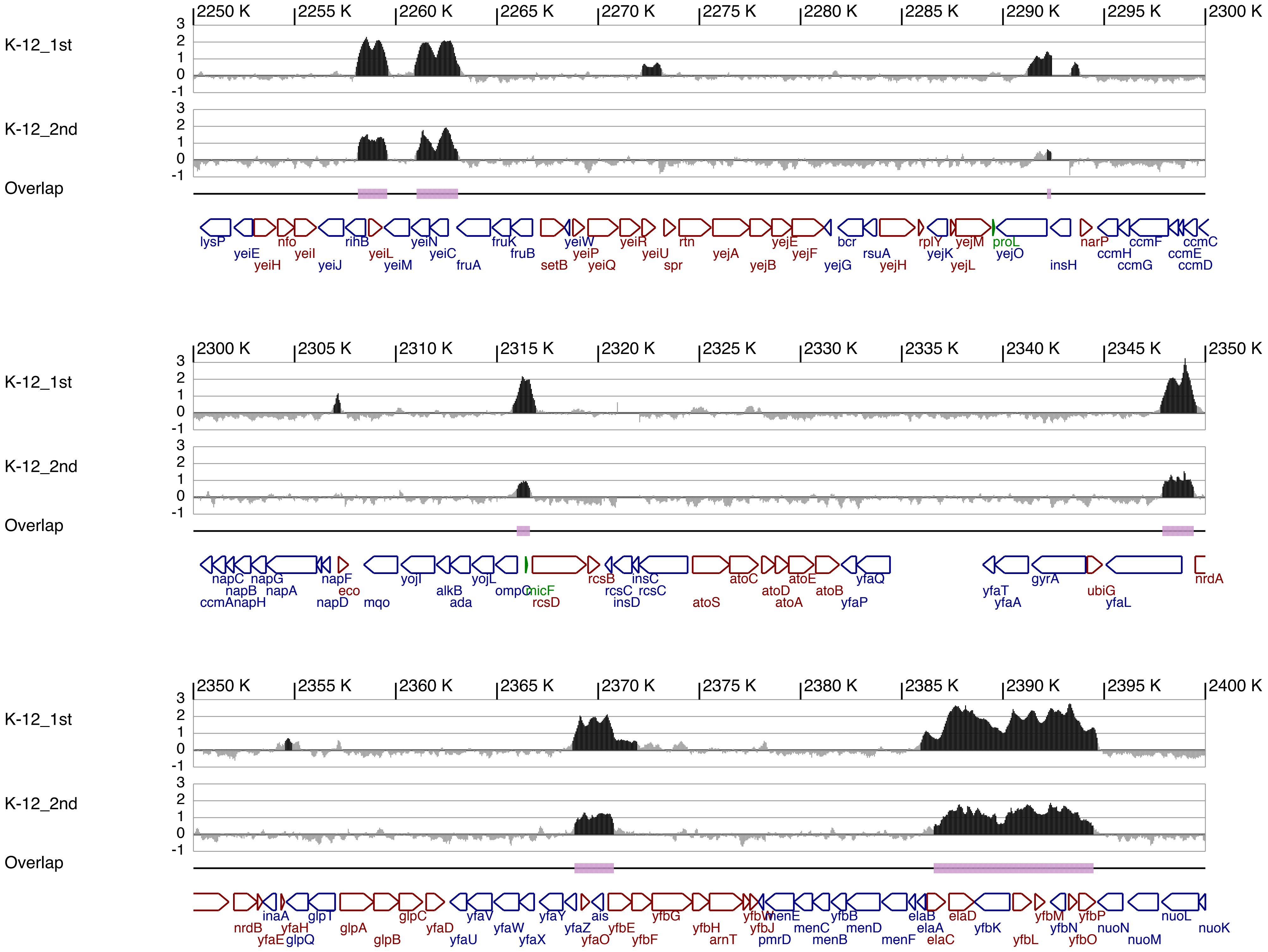

2250 K 2255 K 2260 K 2265 K 2270 K 2275 K 2280 K 2285 K 2290 K 2295 K 2300 K
K-12_1st
K-12_2nd
Overlap
lysP yeiE yeiH nfo yeiI yeiJ rihB yeiL yeiM yeiN yeiC fruA fruK fruB setB yeiW yeiP yeiQ yeiR yeiU spr rtn yejA yejB yejE yejF yejG bcr rsuA yejH rplY yejK yejL yejM proL yejO insH narP ccmH ccmG ccmF ccmE ccmD ccmC
2300 K 2305 K 2310 K 2315 K 2320 K 2325 K 2330 K 2335 K 2340 K 2345 K 2350 K
K-12_1st
K-12_2nd
Overlap
napC napB ccmAnapH napG napA napF eco napD mqo yojI alkB ada yojL ompC micF rcsD rcsB rcsC insD insC rcsC atoS atoC atoD atoA atoE atoB yfaQ yfaP yfaT yfaA gyrA ubiG yfaL nrdA
2350 K 2355 K 2360 K 2365 K 2370 K 2375 K 2380 K 2385 K 2390 K 2395 K 2400 K
K-12_1st
K-12_2nd
Overlap
nrdB yfaE inaA yfaH glpQ glpT glpA glpB glpC yfaD yfaU yfaV yfaW yfaX yfaY yfaZ yfaO ais yfbE yfbF yfbG yfbH arnT yfbW yfbJ pmrD menE menC menB yfbB menD menF elaB elaA elaC elaD yfbK yfbL yfbM yfbN yfbO yfbP nuoN nuoM nuoL nuoK

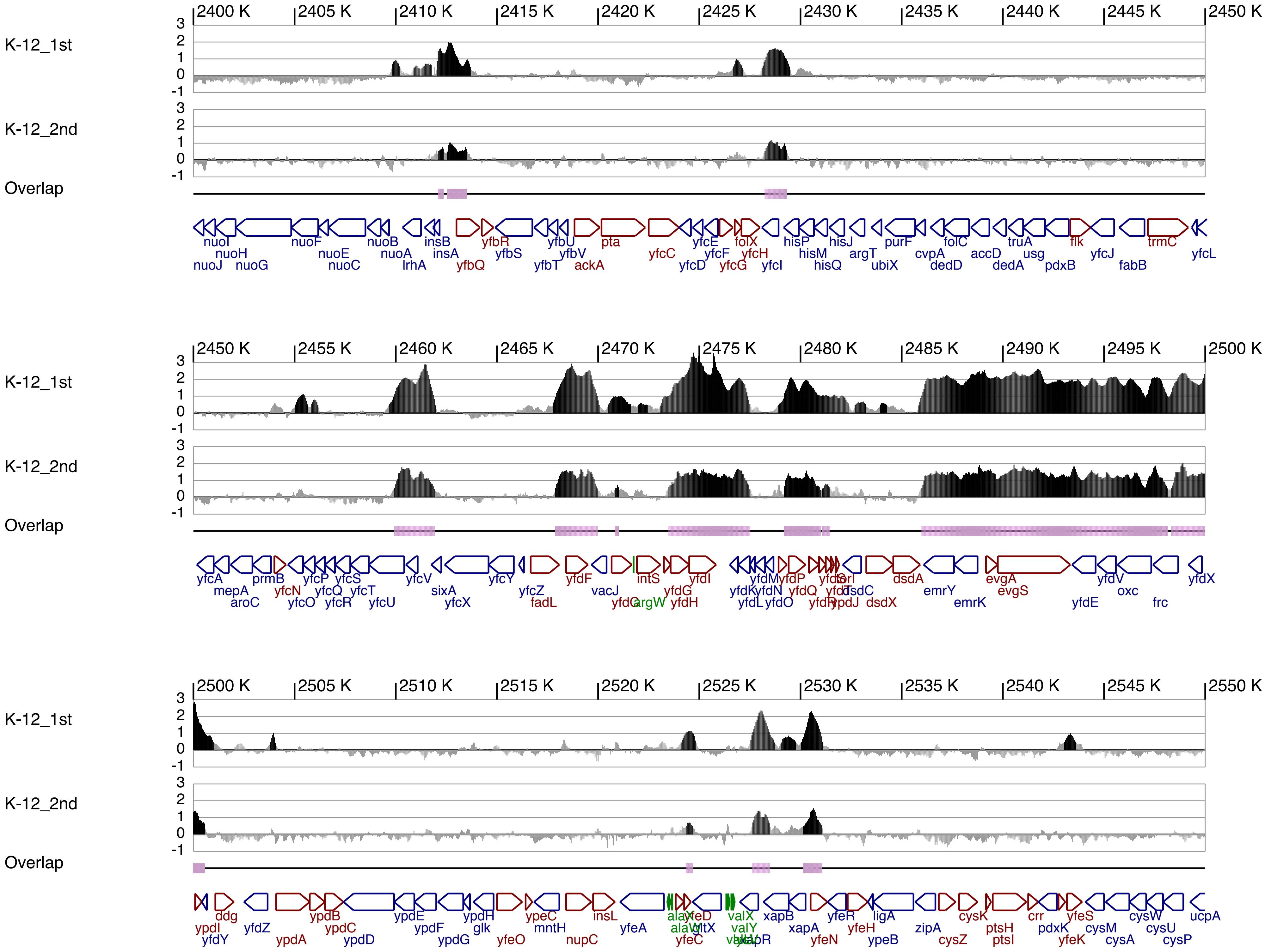
2400 K
2405 K
2410 K
2415 K
2420 K
2425 K
2430 K
2435 K
2440 K
2445 K
2450 K
K-12_1st
K-12_2nd
Overlap
3
2
1
0
-1
nuoI nuoH nuoJ nuoG nuoF nuoE nuoC nuoB nuoA lrhA insB insA yfbR yfbQ yfbS yfbU yfbT yfbV pta ackA yfcC yfcE yfcD yfcF folX yfcG yfcH yfcI hisP hisM hisJ hisQ argT purF ubiX cvpA dedD folC accD truA dedA usg flk pdxB yfcJ trmC fabB yfcL
2455 K
2460 K
2465 K
2470 K
2475 K
2480 K
2485 K
2490 K
2495 K
2500 K
yfcA mepA aroC prmB yfcN yfcO yfcP yfcQ yfcR yfcS yfcT yfcU yfcV sixA yfcX yfcY yfcZ fadL yfdF vacJ yfdC argW intS yfdG yfdH yfdI yfdK yfdL yfdM yfdN yfdO yfdP yfdQ yfdR ypdJ dsdC dsdX dsdA emrY emrK evgA evgS yfdV yfdE oxc frc yfdX
2505 K
2510 K
2515 K
2520 K
2525 K
2530 K
2535 K
2540 K
2545 K
2550 K
ypdI yfdY ddg yfdZ ypdA ypdB ypdC ypdD ypdE ypdF ypdG ypdH glk yfeO ypeC mntH insL nupC yfeA alaX alaW yfeD yfeC gltX valX valY xapB xapA xapR yfeR yfeH yfeN ligA ypeB zipA cysK cysZ ptsH ptsI crr pdxK yfeS yfeK cysM cysA cysW cysU cysP ucpA

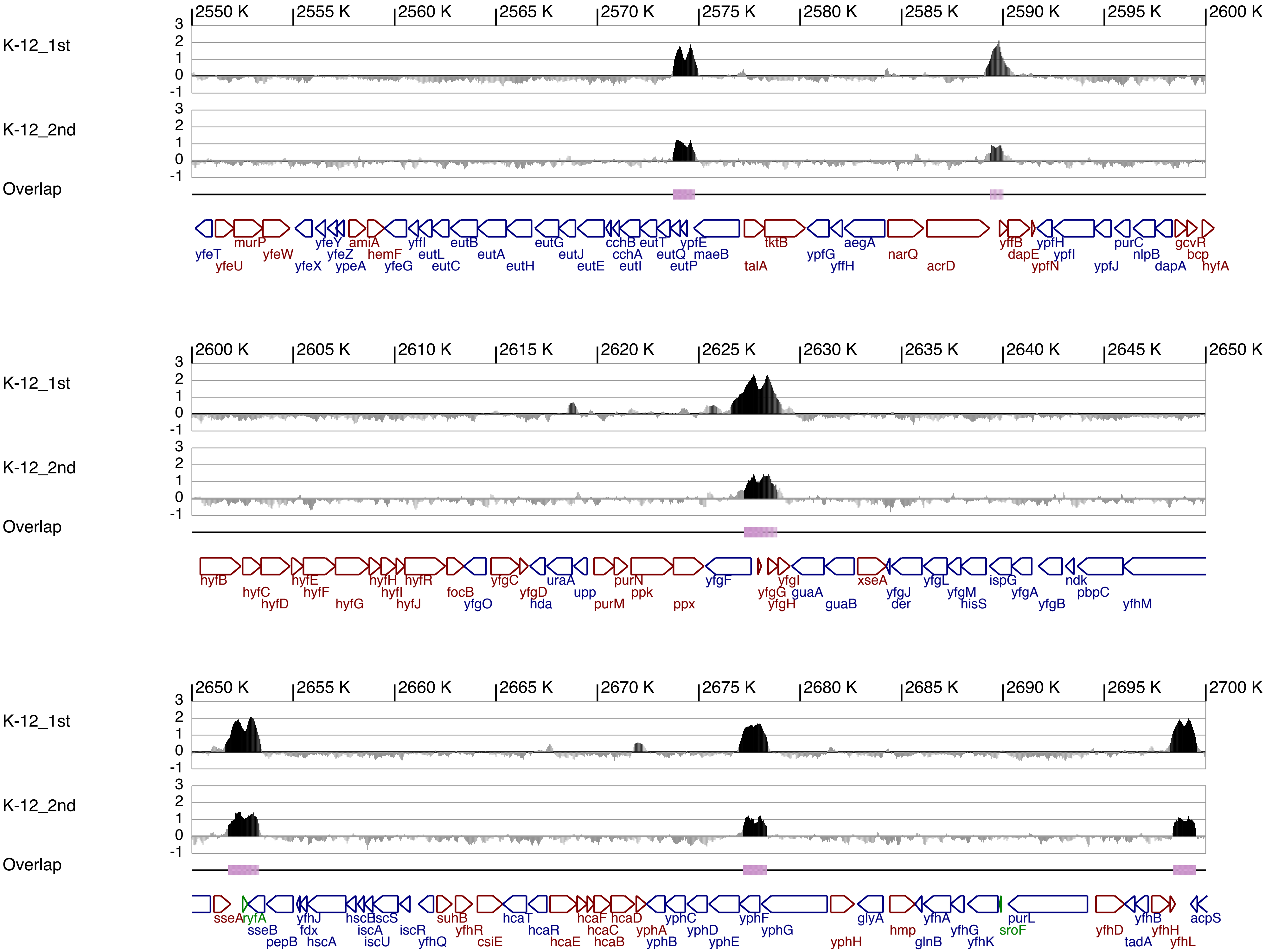

2550 K
2555 K
2560 K
2565 K
2570 K
2575 K
2580 K
2585 K
2590 K
2595 K
2600 K
K-12_1st
K-12_2nd
Overlap
3
2
1
0
-1
murP amiA yffI eutB eutG cchB eutT ypfE tktB aegA yffB ypfH purC gcvR
yfeY yfeZ hemF eutL eutA eutJ cchA eutQ maeB ypfG narQ dapE ypfI nlpB bcp
yfeT yfeW yfeU yfeX ypeA yfeG eutC eutH eutE eutI eutP talA yffH acrD ypfN ypfJ dapA hyfA
2605 K
2610 K
2615 K
2620 K
2625 K
2630 K
2635 K
2640 K
2645 K
2650 K
hyfB hyfE hyfH hyfR yfgC uraA purN yfgF yfgI xseA yfgL ispG ndk
hyfC hyfF hyfI focB yfgD upp ppk yfgG guaA yfgJ yfgM yfgA pbpC
hyfD hyfG hyfJ yfgO hda purM ppx yfgH guaB der hisS yfgB yfhM
2655 K
2660 K
2665 K
2670 K
2675 K
2680 K
2685 K
2690 K
2695 K
2700 K
sseA ryfA yfhJ hscB scS suhB hcaT hcaF hcaD yphC yphF glyA yfhA purL yfhB acpS
sseB fdx iscA iscR yfhR hcaR hcaC yphA yphD yphG hmp yfhG sroF yfhD yfhH
pepB hscA iscU yfhQ csiE hcaE hcaB yphB yphE yphH glnB yfhK tadA yfhL

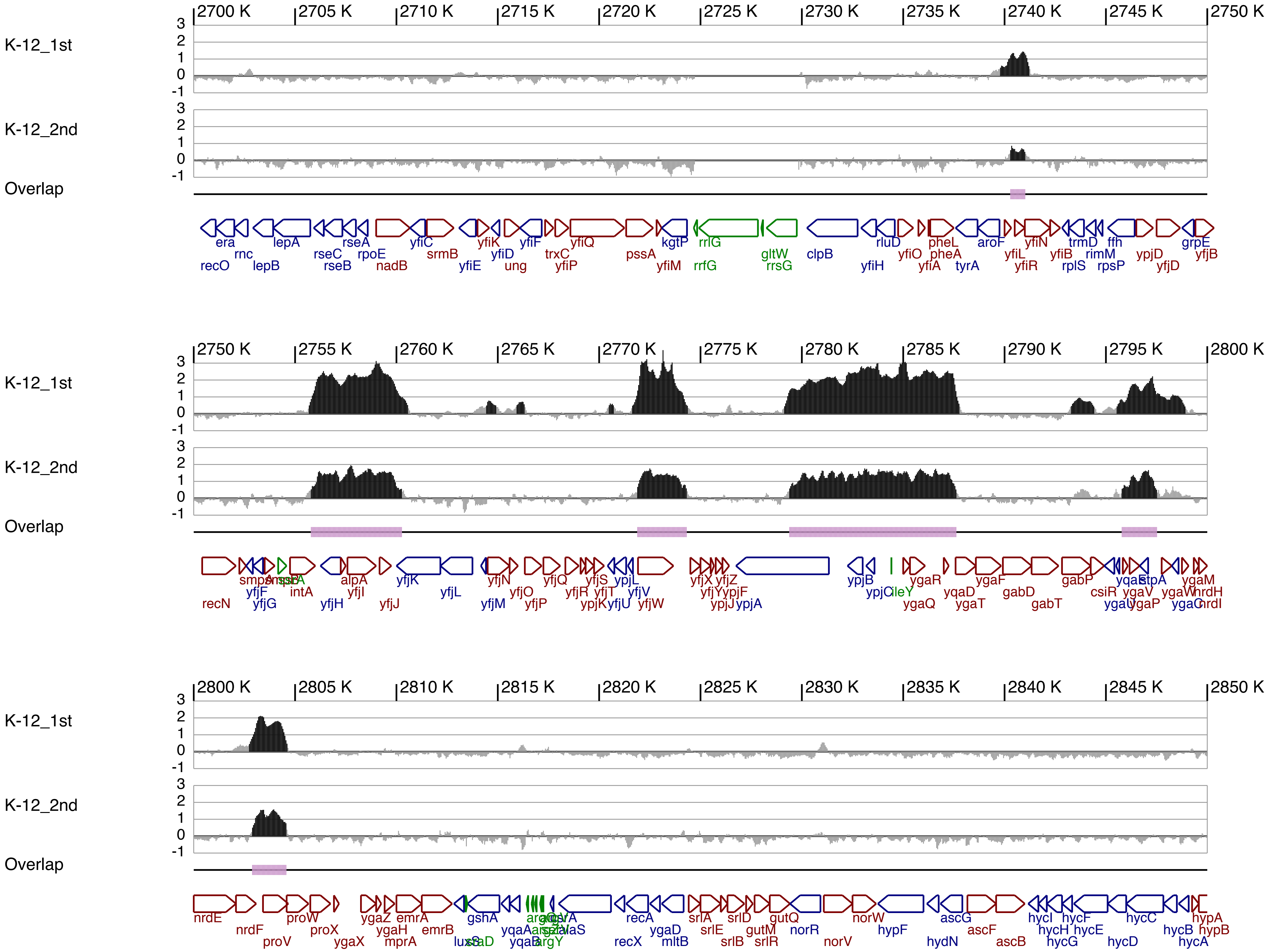

2700 K
2705 K
2710 K
2715 K
2720 K
2725 K
2730 K
2735 K
2740 K
2745 K
2750 K
K-12_1st
K-12_2nd
Overlap
3
2
1
0
-1
era
rnc
recO
lepA
lepB
rseA
rseC
rseB
rpoE
nadB
yfiC
srmB
yfiE
yfiK
yfiD
ung
yfiF
trxC
yfiP
yfiQ
pssA
kgtP
yfiM
rrlG
rrfG
gltW
rrsG
clpB
rluD
yfiH
yfiO
yfiA
pheL
pheA
tyrA
aroF
yfiL
yfiR
yfiN
yfiB
rplS
trmD
rimM
rpsP
ffh
ypjD
yfjD
grpE
yfjB
2750 K
2755 K
2760 K
2765 K
2770 K
2775 K
2780 K
2785 K
2790 K
2795 K
2800 K
K-12_1st
K-12_2nd
Overlap
recN
yfjF
yfjG
intA
alpA
yfjI
yfjH
yfjJ
yfjK
yfjL
yfjN
yfjM
yfjO
yfjP
yfjQ
yfjR
yfjS
yfjT
ypjK
yfjU
ypjL
yfjV
yfjW
yfjX
yfjZ
ypjF
ypjJ
ypjA
ypjB
ypjC
ileY
ygaR
ygaQ
yqaD
ygaT
ygaF
gabD
gabT
gabP
csiR
ygaU
ygaV
ygaP
stpA
ygaW
ygaC
ygaM
nrdH
nrdI
2800 K
2805 K
2810 K
2815 K
2820 K
2825 K
2830 K
2835 K
2840 K
2845 K
2850 K
K-12_1st
K-12_2nd
Overlap
nrdE
nrdF
proV
proW
proX
ygaX
ygaZ
ygaH
mprA
emrA
emrB
gshA
yqaA
yqaB
argY
alaS
recA
recX
ygaD
mltB
srlA
srlE
srlB
srlD
gutM
srlR
gutQ
norR
norV
norW
hypF
ascG
hydN
ascF
ascB
hycI
hycH
hycG
hycF
hycE
hycD
hycC
hycB
hycA
hypA
hypB

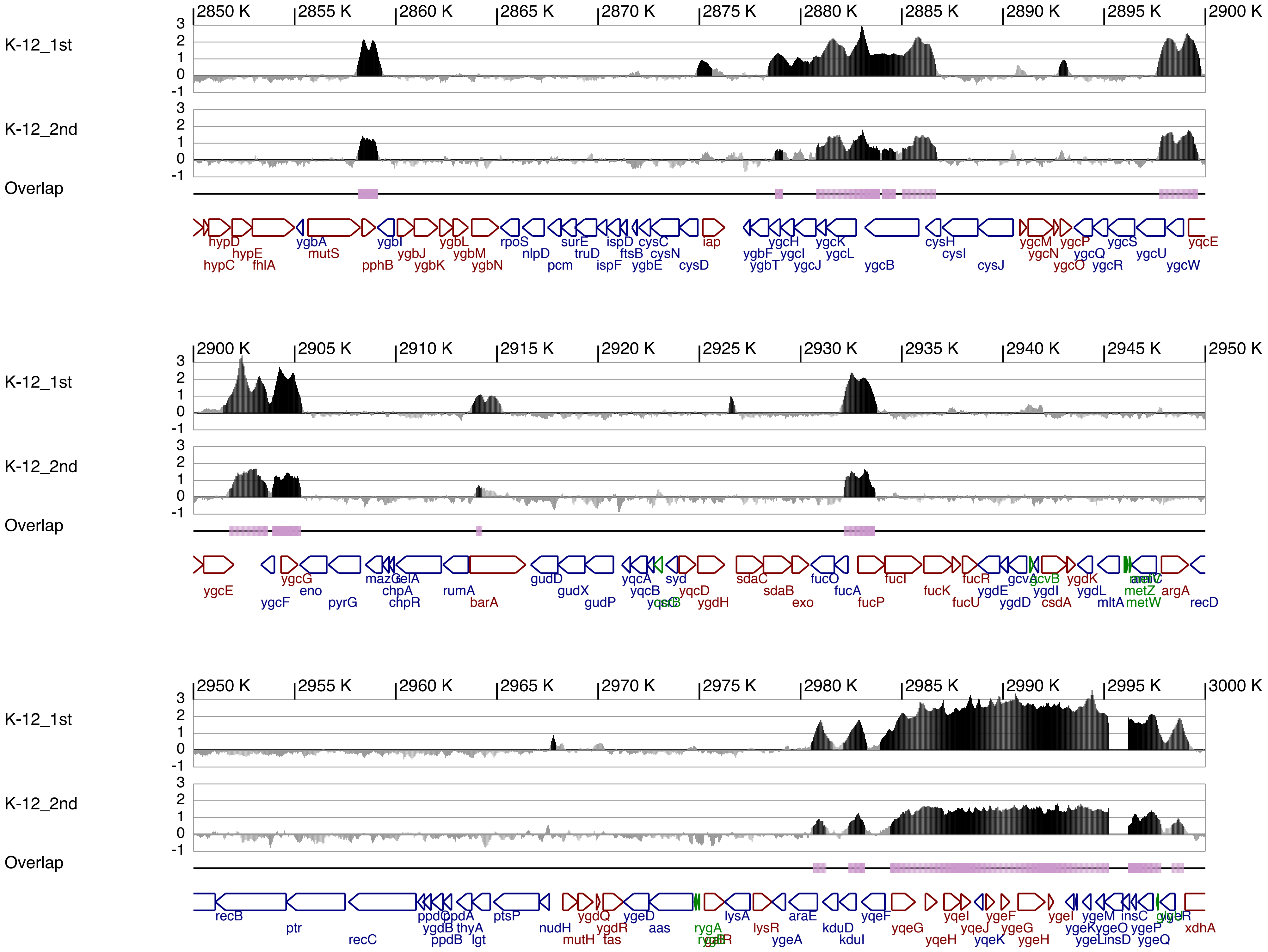

2850 K
2855 K
2860 K
2865 K
2870 K
2875 K
2880 K
2885 K
2890 K
2895 K
2900 K
K-12_1st
K-12_2nd
Overlap
hypD hypE hypC fhlA ygbA mutS ygbI pphB ygbJ ygbK ygbL ygbM ygbN rpoS nlpD pcm surE truD ispF ispD ftsB cysC ygbE cysN cysD iap ygcH ygbF ygcI ygbT ygcJ ygcK ygcL ygcB cysH cysI cysJ ygcM ygcN ygcO ygcP ygcQ ygcR ygcS ygcU ygcW yqcE
2905 K
2910 K
2915 K
2920 K
2925 K
2930 K
2935 K
2940 K
2945 K
2950 K
ygcE ygcF ygcG eno pyrG mazG relA chpA chpR rumA barA gudD gudX gudP yqcA yqcB syd yqcD ygdH sdaC sdaB exo fucO fucA fucP fucI fucK fucR fucU ygdE ygdD ygdI csdA ygdK ygdL mltA metZ metW argA recD
2955 K
2960 K
2965 K
2970 K
2975 K
2980 K
2985 K
2990 K
2995 K
3000 K
recB ptr recC ppdC ppdA ygdB thyA ppdB lgt ptsP nudH ygdQ ygdR mutH tas ygeD aas rygA lysA lysR ygeA araE kduD kduI yqeF yqeG yqeH yqeI yqeJ yqeK ygeF ygeG ygeH ygeI ygeM ygeK ygeO ygeL insD insC ygeP ygeQ xdhA

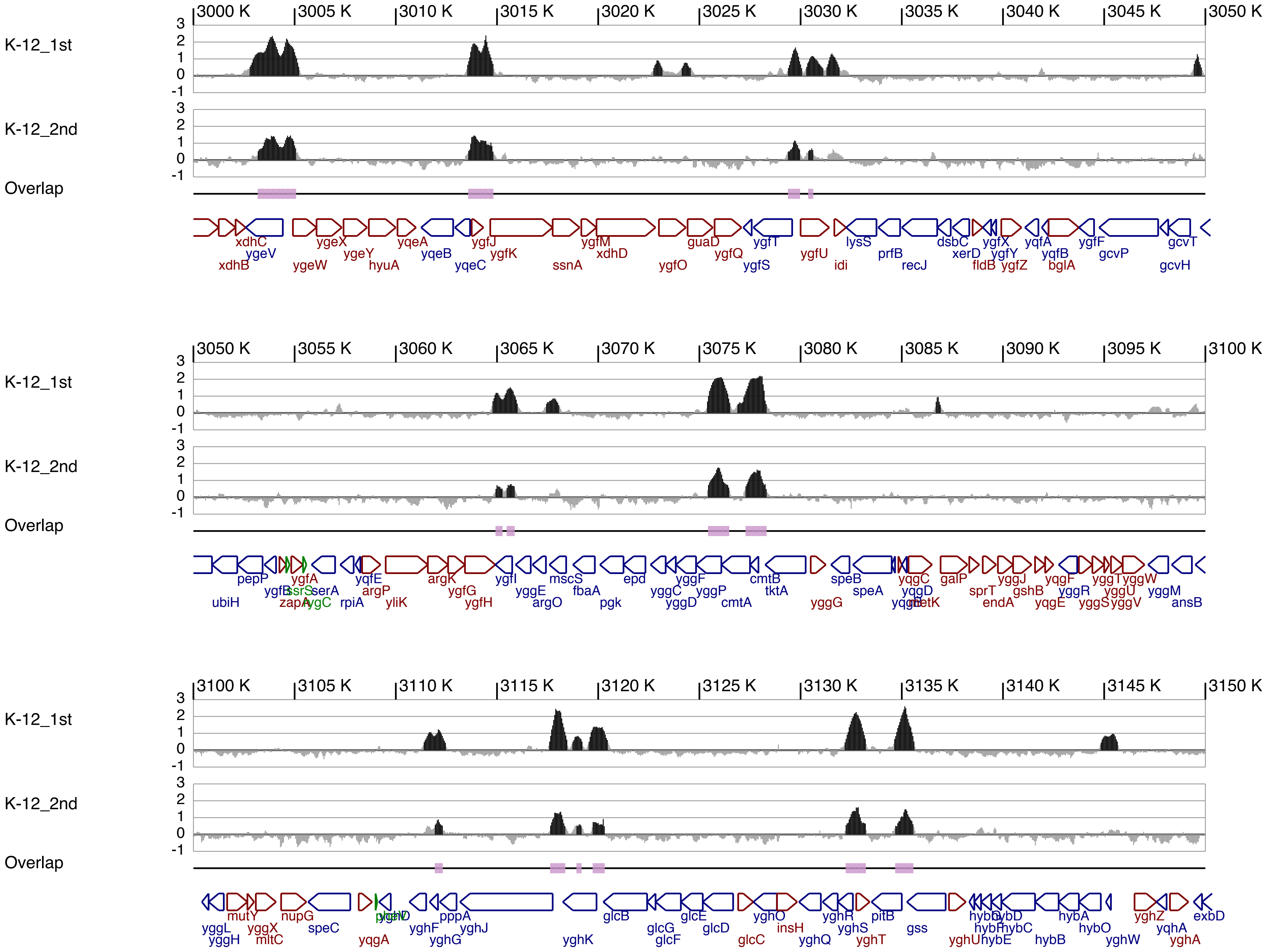

3000 K
3005 K
3010 K
3015 K
3020 K
3025 K
3030 K
3035 K
3040 K
3045 K
3050 K
K-12_1st
K-12_2nd
Overlap
3
2
1
0
-1
xdhC ygeV xdhB ygeX ygeW ygeY hyuA yqeA yqeB yqeC ygfJ ygfK ssnA ygfM xdhD guaD ygfO ygfQ ygfT ygfS ygfU lysS idi prfB recJ dsbC xerD ygfX ygfY fldB ygfZ yqfA yqfB bglA ygfF gcvP gcvT gcvH
3055 K
3060 K
3065 K
3070 K
3075 K
3080 K
3085 K
3090 K
3095 K
3100 K
pepP ubiH ygfB ssrS ygfA serA yqfE rpiA argP yliK argK ygfG ygfH ygfI yggE argO mscS fbaA pgk epd yggC yggF yggD yggP cmtB cmtA tktA yggG speB speA yqgC yqgD galP sprT endA yggJ gshB yqgE yqgF yggR yggS yggT yggU yggV yggW yggM ansB
3105 K
3110 K
3115 K
3120 K
3125 K
3130 K
3135 K
3140 K
3145 K
3150 K
yggL yggH mutY yggX mltC nupG speC yqgA yghF yghG pppA yghJ yghK glcB glcG glcF glcE glcD glcC yghO insH yghQ yghR yghS yghT pitB gss yghU hybE hybB hybA hybO yghW yghZ yqhA yghA exbD

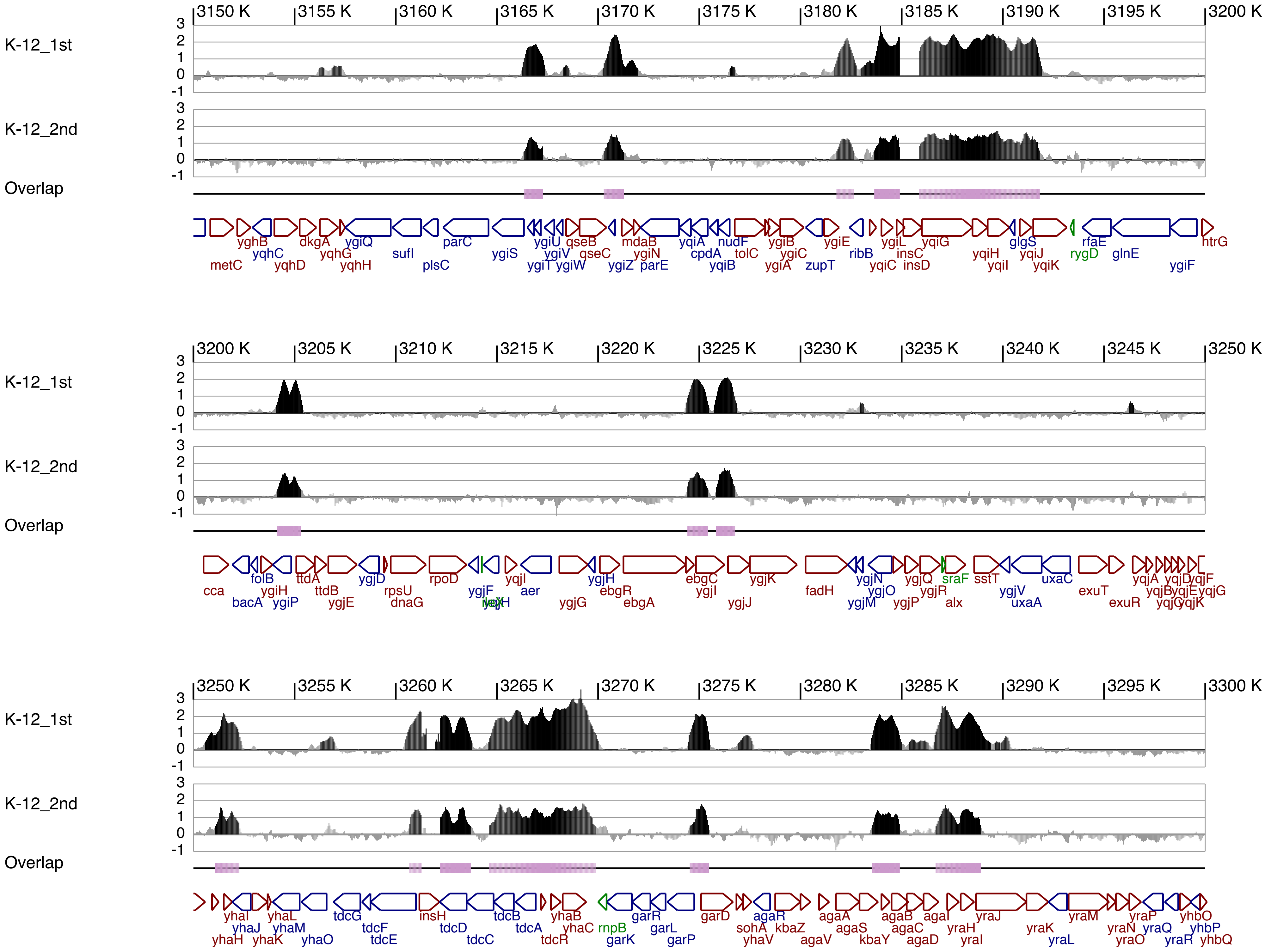

3150 K 3155 K 3160 K 3165 K 3170 K 3175 K 3180 K 3185 K 3190 K 3195 K 3200 K
K-12_1st
K-12_2nd
Overlap
3 2 1 0 -1
yghB dkgA ygiQ parC ygiU qseB mdaB yqiA nudF ygiB ygiE ygiL yqiG glgS rfaE htrG
yqhC yqhG sufI ygiS ygiV qseC ygiN cpdA tolC ygiC ribB insC yqiH yqiJ rygD glnE
metC yqhD yqhH plsC ygiT ygiW ygiZ parE yqiB ygiA zupT yqiC insD yqiI yqiK ygiF
3200 K 3205 K 3210 K 3215 K 3220 K 3225 K 3230 K 3235 K 3240 K 3245 K 3250 K
K-12_1st
K-12_2nd
Overlap
folB ttdA ygjD rpoD yqjI ygjH ebgC ygjK ygjN ygjQ sraF sstT uxaC yqjA yqjD yqjF
cca ygiH ttdB rpsU ygjF aer ebgR ygjI fadH ygjO ygjR ygjV exuT yqjB yqjE yqjG
bacA ygiP ygjE dnaG ygjG ebgA ygjJ ygjM ygjP alx uxaA exuR yqjC yqjK
3250 K 3255 K 3260 K 3265 K 3270 K 3275 K 3280 K 3285 K 3290 K 3295 K 3300 K
K-12_1st
K-12_2nd
Overlap
yhaI yhaL tdcG insH tdcB yhaB garR garD agaR agaA agaB agaI yraJ yraM yraP yhbO
yhaJ yhaM tdcF tdcD tdcA yhaC rnpB garL sohA kbaZ agaS agaC yraH yraK yraN yraQ yhbP
yhaH yhaK yhaO tdcE tdcC tdcR garK garP yhaV agaV kbaY agaD yraI yraL yraO yraR yhbQ

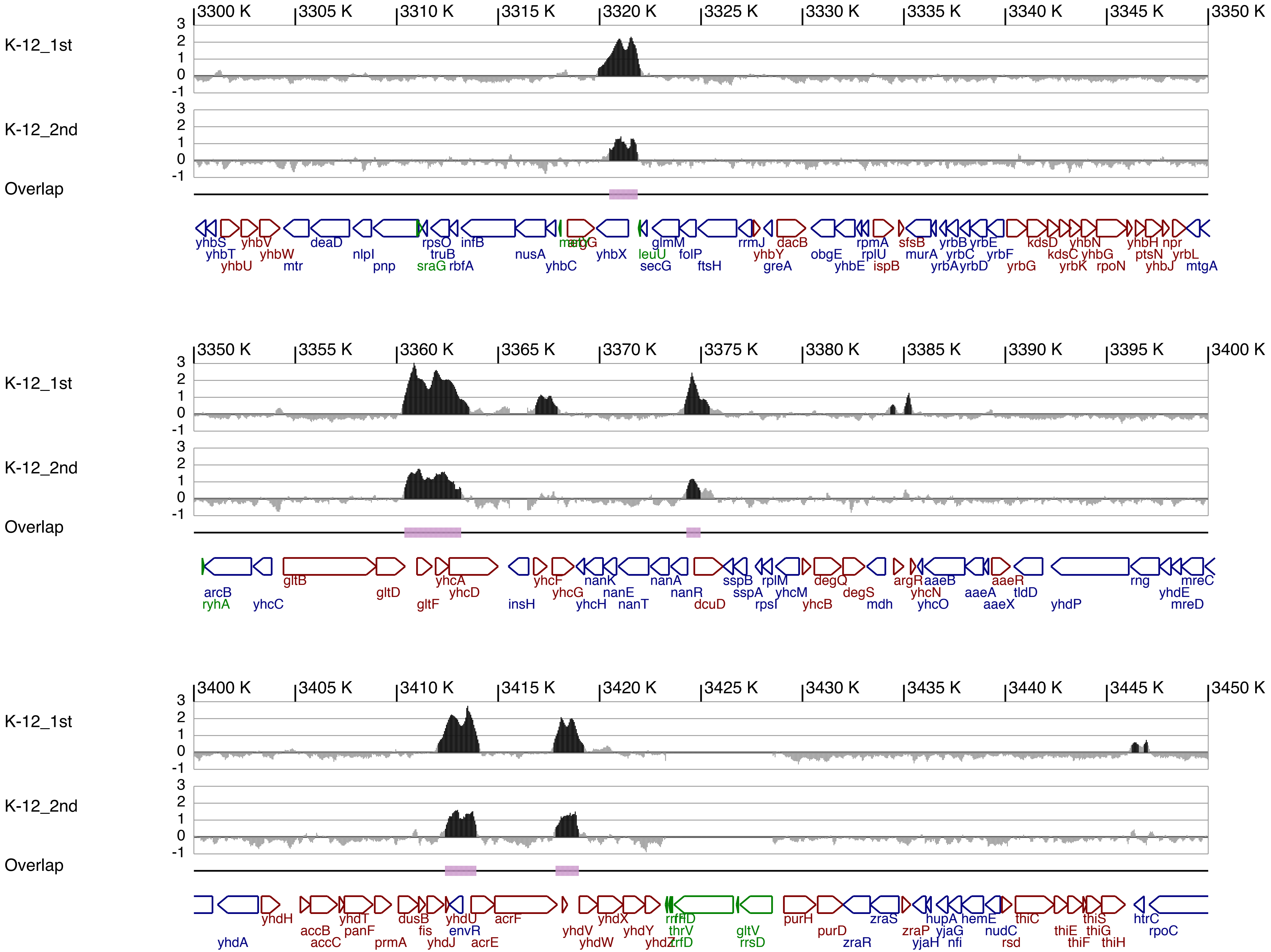

3300 K
3305 K
3310 K
3315 K
3320 K
3325 K
3330 K
3335 K
3340 K
3345 K
3350 K
K-12_1st
K-12_2nd
Overlap
3
2
1
0
-1
yhbS yhbT yhbU yhbV yhbW mtr deaD nlpI pnp rpsO truB sraG rbfA infB nusA yhbC yhbX leuU secG glmM folP ftsH rrmJ ybhY greA dacB obgE yhbE rpmA rplU ispB sfsB murA yrbA yrbB yrbC yrbD yrbE yrbF yrbG kdsD kdsC yrbK yhbN yhbG rpoN yhbH ptsN yhbJ npr yrbL mtgA
3350 K
3355 K
3360 K
3365 K
3370 K
3375 K
3380 K
3385 K
3390 K
3395 K
3400 K
arcB ryhA yhcC gltB gltD gltF yhcA yhcD insH yhcF yhcG yhcH nanK nanE nanT nanA nanR dcuD sspB sspA rpsI rplM yhcM yhcB degQ degS mdh argR yhcN yhcO aaeB aaeA aaeX aaeR tldD yhdP rng yhdE mreC mreD
3400 K
3405 K
3410 K
3415 K
3420 K
3425 K
3430 K
3435 K
3440 K
3445 K
3450 K
yhdA yhdH accB accC yhdT panF prmA dusB fis yhdJ yhdU envR acrE acrF yhdV yhdW yhdX yhdY yhdZ thrV rrfD gltV rrsD purH purD zraS zraR zraP yjaH hupA yjaG nfi hemE nudC rsd thiC thiE thiF thiS thiG thiH htrC rpoC

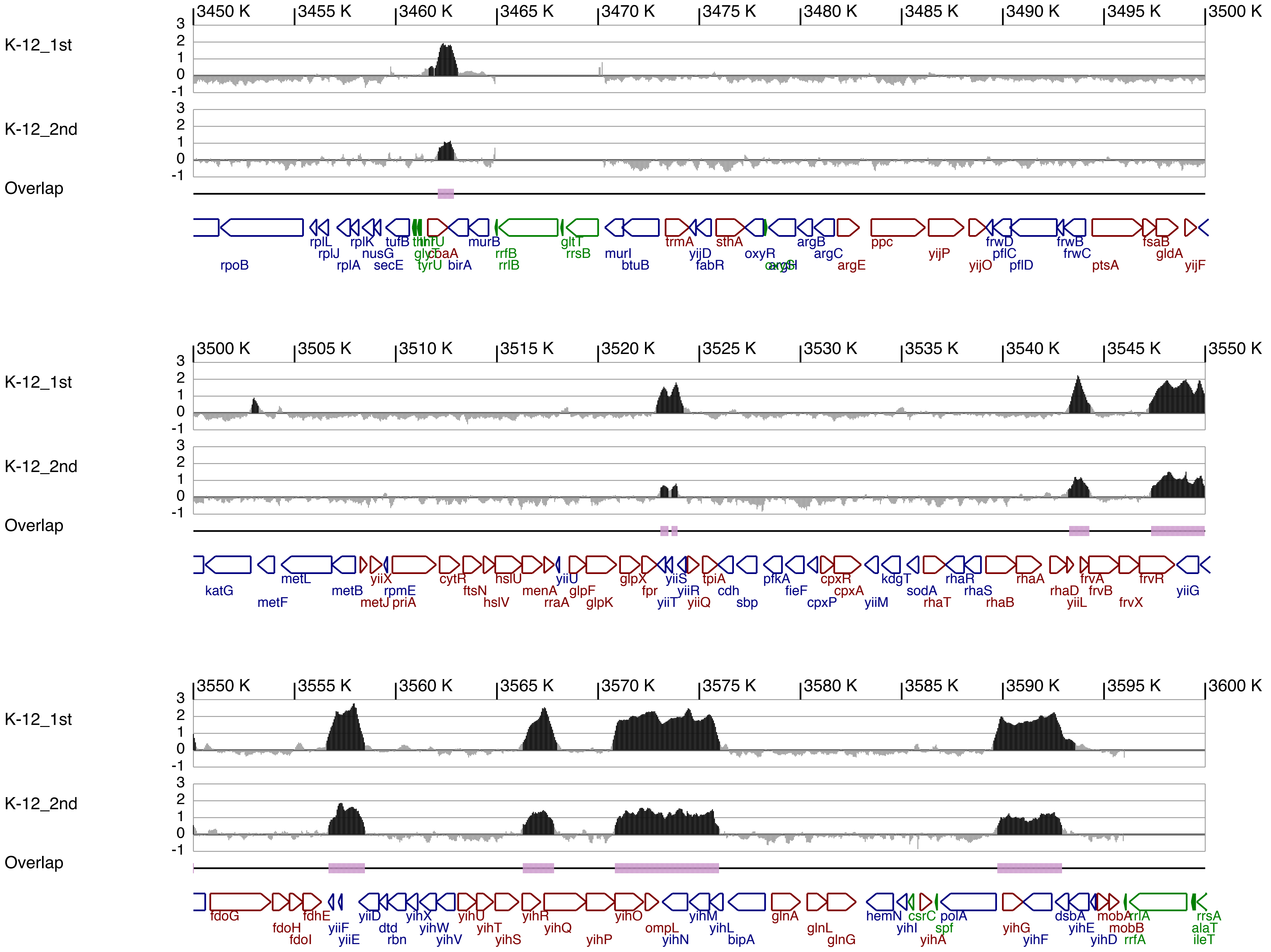

3450 K
3455 K
3460 K
3465 K
3470 K
3475 K
3480 K
3485 K
3490 K
3495 K
3500 K
K-12_1st
K-12_2nd
Overlap
rpoB
rplL
rplJ
rplK
rplA
nusG
secE
tufB
thrU
glyT
tyrU
coaA
birA
murB
rrfB
rrlB
gltT
rrsB
murI
btuB
trmA
yijD
fabR
sthA
oxyR
argB
argC
argE
ppc
yijP
frwD
pflC
yijO
pflD
frwB
frwC
ptsA
fsaB
gldA
yijF
3505 K
3510 K
3515 K
3520 K
3525 K
3530 K
3535 K
3540 K
3545 K
3550 K
katG
metF
metL
metB
yiiX
rpmE
metJ
priA
cytR
ftsN
hslU
hslV
menA
rraA
yiiU
glpF
glpK
glpX
fpr
yiiS
yiiT
yiiR
yiiQ
tpiA
cdh
sbp
pfkA
fieF
cpxR
cpxA
cpxP
yiiM
kdgT
sodA
rhaT
rhaR
rhaS
rhaB
rhaA
rhaD
yiiL
frvA
frvB
frvX
frvR
yiiG
3555 K
3560 K
3565 K
3570 K
3575 K
3580 K
3585 K
3590 K
3595 K
3600 K
fdoG
fdoH
fdoI
fdhE
yiiF
yiiE
yiiD
dtd
rbn
yihX
yihW
yihV
yihU
yihT
yihS
yihR
yihQ
yihP
yihO
ompL
yihN
yihM
yihL
bipA
glnA
glnL
glnG
hemN
yihI
csrC
spf
polA
yihA
yihG
yihF
dsbA
yihE
yihD
mobA
mobB
rrlA
rrfA
rrsA
alaT
ileT

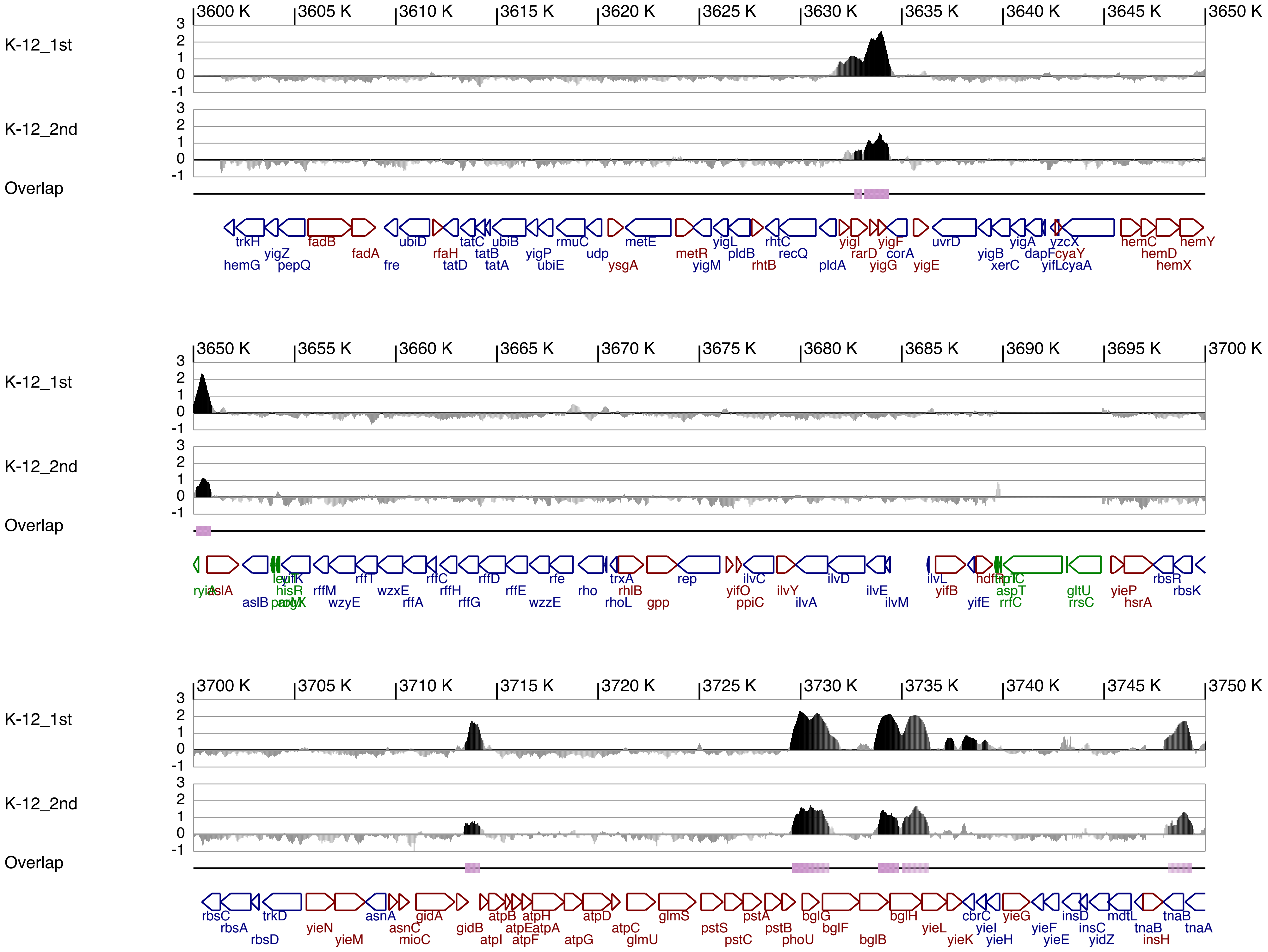

3600 K
3605 K
3610 K
3615 K
3620 K
3625 K
3630 K
3635 K
3640 K
3645 K
3650 K
K-12_1st
K-12_2nd
Overlap
3
2
1
0
-1
trkH
hemG
yigZ
pepQ
fadB
fadA
fre
ubiD
rfaH
tatD
tatC
tatB
tatA
ubiB
yigP
ubiE
rmuC
udp
ysgA
metE
metR
yigM
yigL
pldB
rhtB
rhtC
recQ
pldA
yigI
rarD
yigG
yigF
corA
yigE
uvrD
yigB
xerC
yigA
dapF
yzcX
cyaY
yifL
cyaA
hemC
hemD
hemX
hemY
3650 K
3655 K
3660 K
3665 K
3670 K
3675 K
3680 K
3685 K
3690 K
3695 K
3700 K
aslA
aslB
hisR
rffM
wzyE
rffT
wzxE
rffA
rffC
rffH
rffG
rffD
rffE
wzzE
rfe
rho
rhoL
trxA
rhlB
gpp
rep
yifO
ppiC
ilvC
ilvY
ilvA
ilvD
ilvE
ilvM
ilvL
yifB
yifE
aspT
rrfC
gltU
rrsC
yieP
hsrA
rbsR
rbsK
3700 K
3705 K
3710 K
3715 K
3720 K
3725 K
3730 K
3735 K
3740 K
3745 K
3750 K
rbsC
rbsA
rbsD
trkD
yieN
yieM
asnA
asnC
mioC
gidA
gidB
atpB
atpI
atpH
atpF
atpD
atpG
atpC
glmU
glmS
pstS
pstC
pstA
pstB
phoU
bglG
bglF
bglB
bglH
yieL
yieK
cbrC
yieI
yieH
yieG
yieF
yieE
insD
insC
yidZ
mdtL
tnaB
insH
tnaA

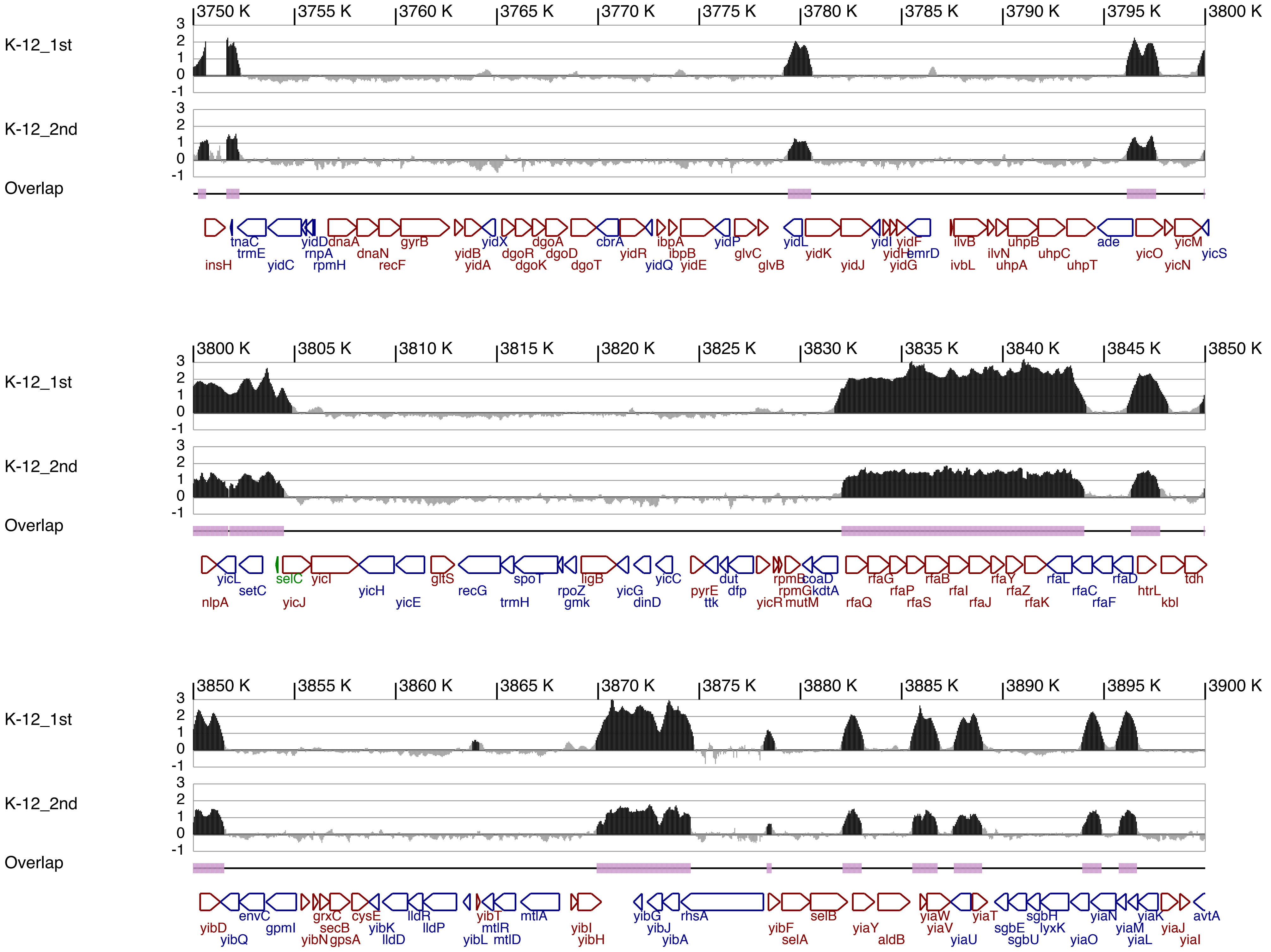
3750 K
3755 K
3760 K
3765 K
3770 K
3775 K
3780 K
3785 K
3790 K
3795 K
3800 K
K-12_1st
K-12_2nd
Overlap
3
2
1
0
-1
tnaC
trmE
insH
yidC
yidD
rnpA
rpmH
dnaA
dnaN
recF
gyrB
yidB
yidA
yidX
dgoR
dgoK
dgoA
dgoD
dgoT
cbrA
yidR
ibpA
ibpB
yidQ
yidE
yidP
glvC
glvB
yidL
yidK
yidJ
yidI
yidH
yidF
emrD
yidG
ilvB
ivbL
ilvN
uhpA
uhpB
uhpC
uhpT
ade
yicO
yicN
yicM
yicS
3805 K
3810 K
3815 K
3820 K
3825 K
3830 K
3835 K
3840 K
3845 K
3850 K
yicL
setC
nlpA
selC
yicI
yicJ
yicH
yicE
gltS
recG
spoT
trmH
rpoZ
gmk
ligB
yicG
dinD
yicC
pyrE
ttk
dut
dfp
yicR
rpmB
rpmG
mutM
coaD
kdtA
rfaG
rfaQ
rfaP
rfaS
rfaB
rfaI
rfaJ
rfaY
rfaZ
rfaK
rfaL
rfaC
rfaF
rfaD
htrL
kbl
tdh
3855 K
3860 K
3865 K
3870 K
3875 K
3880 K
3885 K
3890 K
3895 K
3900 K
yibD
yibQ
envC
gpmI
grxC
secB
yibN
gpsA
cysE
yibK
lldD
lldR
lldP
yibT
mtlR
yibL
mtlD
mtlA
yibI
yibH
yibG
yibJ
yibA
rhsA
yibF
selA
selB
yiaY
aldB
yiaW
yiaV
yiaU
yiaT
sgbE
sgbU
sgbH
lyxK
yiaO
yiaN
yiaM
yiaL
yiaK
yiaJ
yiaI
avtA

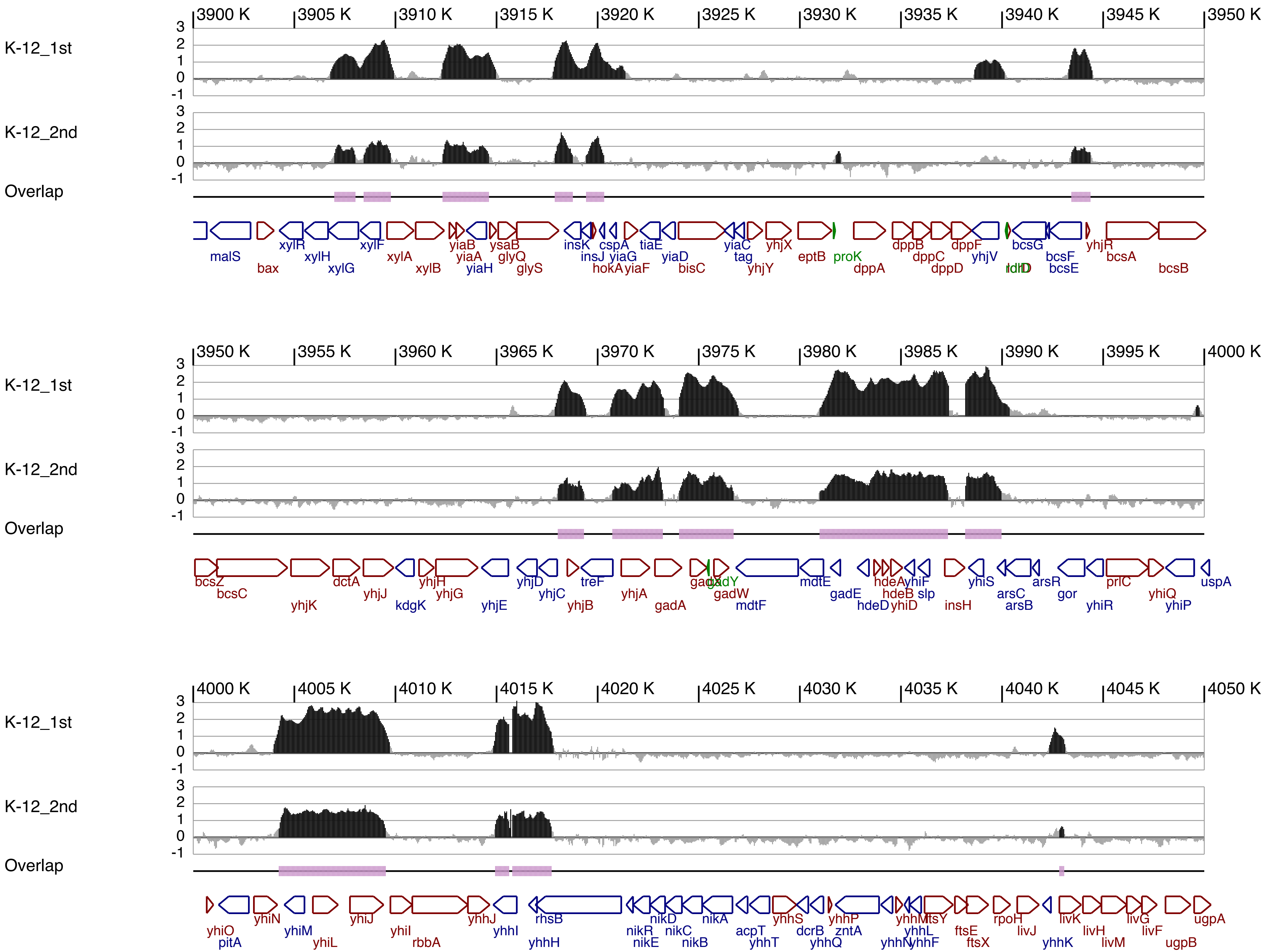

3900 K
3905 K
3910 K
3915 K
3920 K
3925 K
3930 K
3935 K
3940 K
3945 K
3950 K
K-12_1st
K-12_2nd
Overlap
malS
bax
xylR
xylH
xylG
xylF
xylA
xylB
yiaB
yiaA
yiaH
ysaB
glyQ
glyS
insK
insJ
hokA
cspA
yiaG
yiaF
tiaE
yiaD
bisC
yiaC
tag
yhjY
yhjX
eptB
proK
dppA
dppB
dppC
dppD
dppF
yhjV
bcsG
bcsF
bcsE
yhjR
bcsA
bcsB
3955 K
3960 K
3965 K
3970 K
3975 K
3980 K
3985 K
3990 K
3995 K
4000 K
bcsZ
bcsC
yhjK
dctA
yhjJ
kdgK
yhjH
yhjG
yhjE
yhjD
yhjC
treF
yhjB
yhjA
gadA
gadY
gadW
mdtF
mdtE
gadE
hdeD
hdeA
hdeB
yhiF
yhiD
slp
insH
yhiS
arsC
arsB
arsR
gor
yhiR
prlC
yhiQ
yhiP
uspA
4005 K
4010 K
4015 K
4020 K
4025 K
4030 K
4035 K
4040 K
4045 K
4050 K
yhiO
pitA
yhiN
yhiM
yhiL
yhiJ
yhiI
rbbA
yhhJ
yhhI
rhsB
yhhH
nikR
nikE
nikD
nikC
nikB
nikA
acpT
yhhT
yhhS
dcrB
yhhQ
yhhP
zntA
yhhM
yhhL
tsY
yhhF
ftsE
ftsX
rpoH
livJ
yhhK
livK
livH
livM
livG
livF
ugpB
ugpA

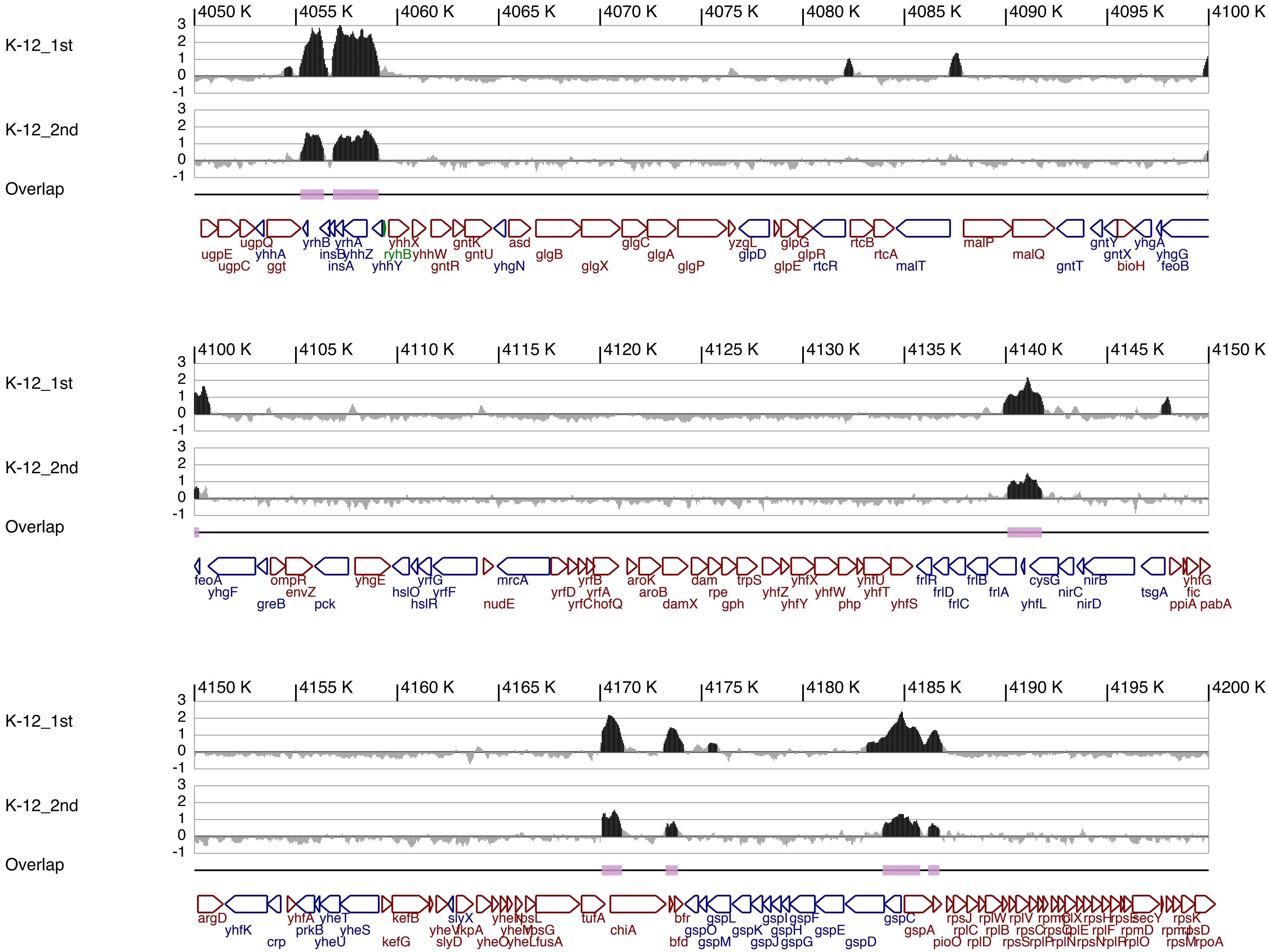

4050 K
4055 K
4060 K
4065 K
4070 K
4075 K
4080 K
4085 K
4090 K
4095 K
4100 K
K-12_1st
K-12_2nd
Overlap
3
2
1
0
-1
ugpE ugpC ugpQ yhhA ggt yrhB yrhA insA ryhB yhhX yhhW yhhY gntK gntR gntU asd yhgN glgB glgX glgC glgA glgP yzgL glpD glpG glpR glpE rtcR rtcB rtcA malT malP malQ gntT gntY gntX bioH yhgA yhgG feoB
4100 K
4105 K
4110 K
4115 K
4120 K
4125 K
4130 K
4135 K
4140 K
4145 K
4150 K
K-12_1st
K-12_2nd
Overlap
feoA yhgF ompR envZ greB pck yhgE hslO yrfG hslR yrfF mrcA nudE yrfD yrfB yrfA aroK aroB dam damX rpe gph trpS yhfZ yhfY yhfX yhfW php yhfU yhfT yhfS frlR frlD frlC frlB frlA cysG yhfL nirC nirB nirD tsgA yhfG fic
4150 K
4155 K
4160 K
4165 K
4170 K
4175 K
4180 K
4185 K
4190 K
4195 K
4200 K
K-12_1st
K-12_2nd
Overlap
argD yhfK crp yhfA prkB yheT yheU yheS kefB kefG slyX slyD fusA tufA chiA bfr bfd gspO gspL gspM gspK gspJ gspH gspG gspF gspE gspD gspC gspA pioO rpsJ rplC rplD rplB rplW rplV rplF rpmD rplO rpsK rpoA

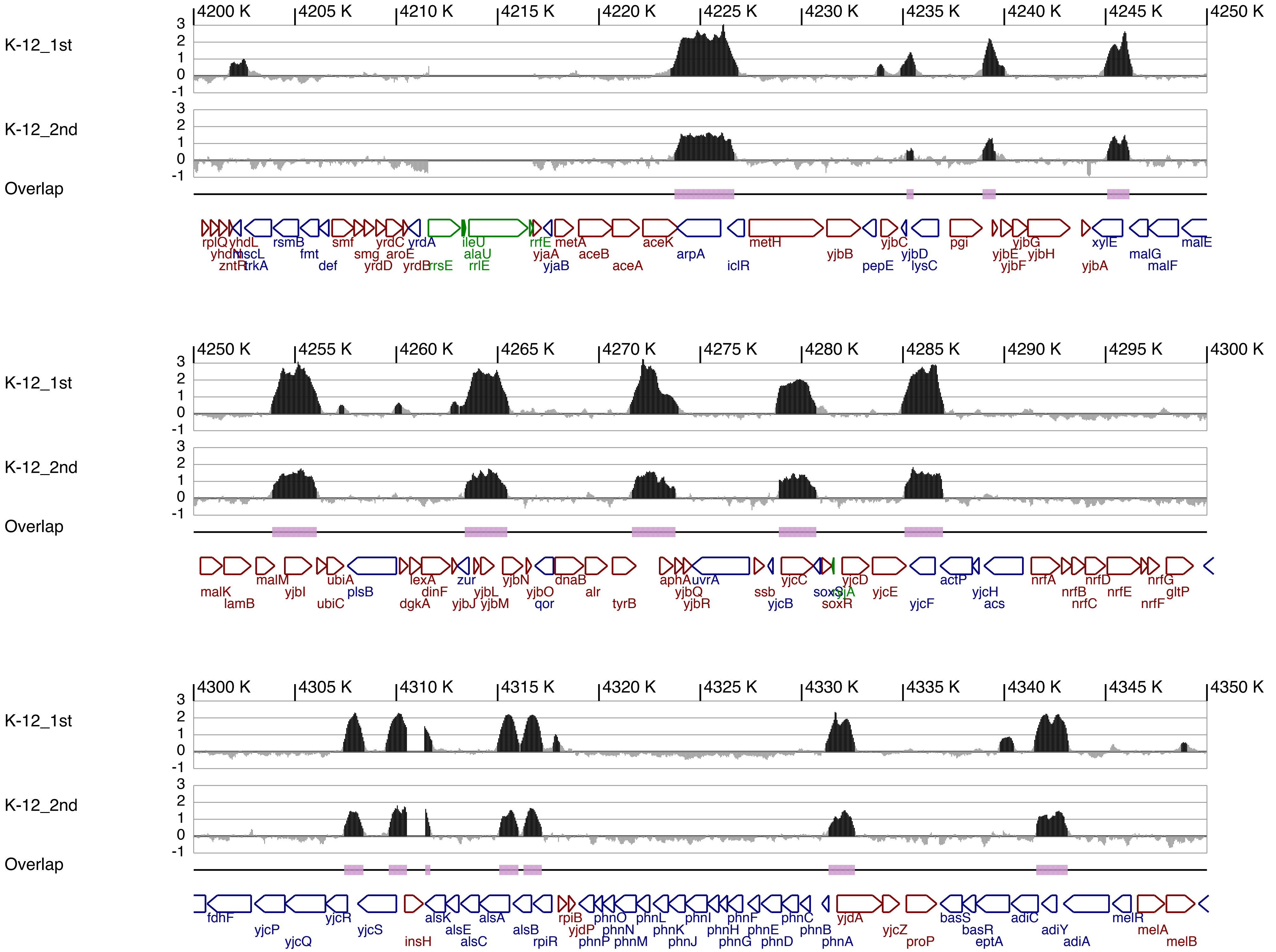

4200 K
4205 K
4210 K
4215 K
4220 K
4225 K
4230 K
4235 K
4240 K
4245 K
4250 K
K-12_1st
K-12_2nd
Overlap
3
2
1
0
-1
rplQ yhdL rsmB smf yrdC yrdA ileU rrfE metA aceK arpA metH yjbC pgi yjbG xylE malE
yhdN mscL fmt smg aroE alaU yjaA aceB yjbB yjbD yjbE yjbH malG
zntR trkA def yrdD yrdB rrsE rrlE yjaB aceA iclR pepE lysC yjbF yjbA malF
4250 K
4255 K
4260 K
4265 K
4270 K
4275 K
4280 K
4285 K
4290 K
4295 K
4300 K
malM ubiA lexA zur yjbN dnaB aphA uvrA yjcC yjcD actP nrfA nrfD nrfG
malK yjbI plsB dinF yjbL yjbO alr yjbQ ssb soxS yjjA yjcE yjcH nrfB nrfE gltP
lamB ubiC dgkA yjbJ yjbM qor tyrB yjbR yjcB soxR yjcF acs nrfC nrfF
4300 K
4305 K
4310 K
4315 K
4320 K
4325 K
4330 K
4335 K
4340 K
4345 K
4350 K
fdhF yjcR alsK alsA rpiB phnO phnL phnI phnF phnC yjdA basS adiC melR
yjcP yjcS alsE alsB yjdP phnN phnK phnH phnE phnB yjcZ basR adiY melA
yjcQ insH alsC rpiR phnP phnM phnJ phnG phnD phnA proP eptA adiA melB

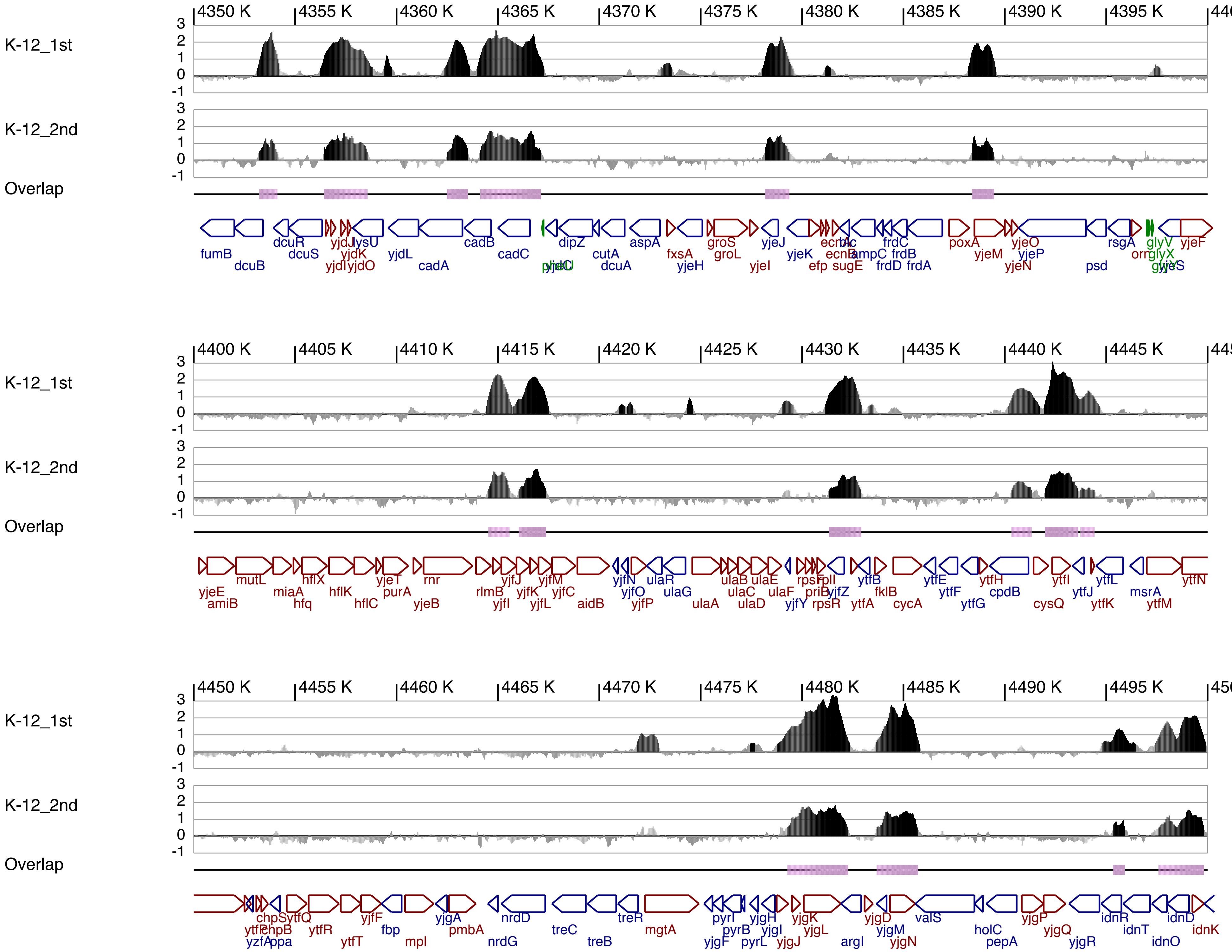

4350 K
4355 K
4360 K
4365 K
4370 K
4375 K
4380 K
4385 K
4390 K
4395 K
4400 K
K-12_1st
K-12_2nd
Overlap
fumB
dcuB
dcuR
dcuS
yjdJ
yjdK
yjdI
yjdO
lysU
yjdL
cadA
cadB
cadC
dipZ
cutA
dcuA
aspA
fxsA
yjeH
groS
groL
yjeI
yjeJ
yjeK
efp
sugE
ampC
frdC
frdB
frdD
frdA
poxA
yjeM
yjeO
yjeP
yjeN
psd
rsgA
glyV
glyX
yjeF
4405 K
4410 K
4415 K
4420 K
4425 K
4430 K
4435 K
4440 K
4445 K
4450 K
yjeE
amiB
mutL
miaA
hfq
hflX
hflK
hflC
yjeT
purA
yjeB
rnr
rlmB
yjfI
yjfJ
yjfK
yjfL
yjfM
yjfC
aidB
yjfN
yjfO
yjfP
ulaR
ulaG
ulaA
ulaB
ulaC
ulaD
ulaE
ulaF
yjfY
rpsR
priB
yjfZ
ytfA
ytfB
fklB
cycA
ytfE
ytfF
ytfG
ytfH
cpdB
cysQ
ytfI
ytfJ
ytfK
ytfL
msrA
ytfM
ytfN
4455 K
4460 K
4465 K
4470 K
4475 K
4480 K
4485 K
4490 K
4495 K
4500 K
chpB
ytfR
ytfT
yjfF
fbp
mpl
yjgA
pmbA
nrdD
nrdG
treC
treB
treR
mgtA
pyrI
pyrB
pyrL
yjgF
yjgH
yjgI
yjgJ
yjgK
yjgL
argI
yjgD
yjgM
yjgN
valS
holC
pepA
yjgP
yjgQ
yjgR
idnR
idnT
idnO
idnD
idnK

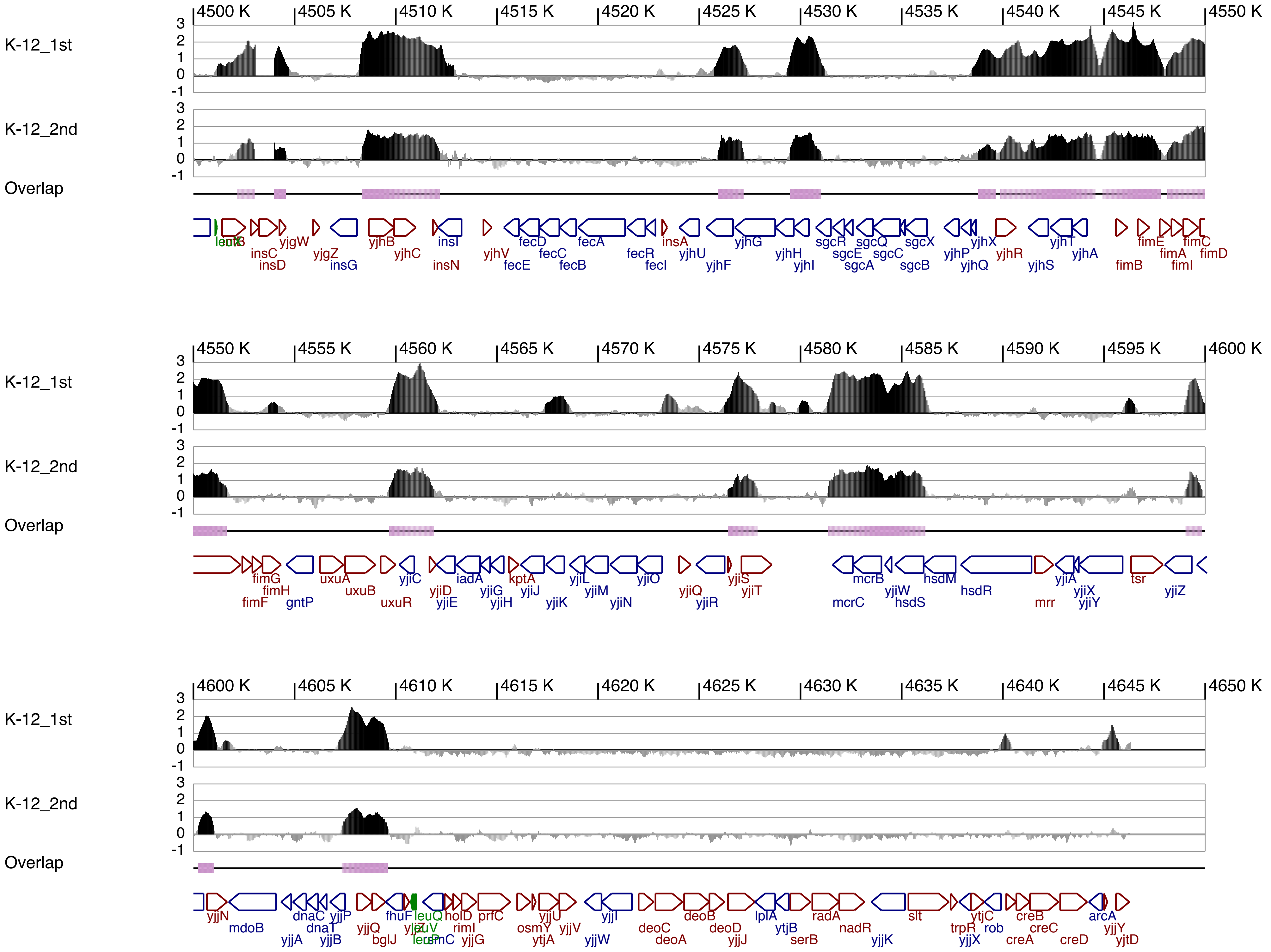

4500 K
4505 K
4510 K
4515 K
4520 K
4525 K
4530 K
4535 K
4540 K
4545 K
4550 K
K-12_1st
K-12_2nd
Overlap
3
2
1
0
-1
insC
insD
yjgW
yjgZ
insG
yjhB
yjhC
insI
insN
yjhV
fecD
fecE
fecC
fecB
fecA
fecR
fecI
insA
yjhU
yjhF
yjhG
yjhH
yjhI
sgcR
sgcE
sgcA
sgcQ
sgcC
sgcX
sgcB
yjhP
yjhQ
yjhX
yjhR
yjhS
yjhT
yjhA
fimB
fimE
fimA
fimI
fimC
fimD
4550 K
4555 K
4560 K
4565 K
4570 K
4575 K
4580 K
4585 K
4590 K
4595 K
4600 K
fimF
fimG
fimH
gntP
uxuA
uxuB
uxuR
yjiC
yjiD
yjiE
iadA
yjiG
yjiH
kptA
yjiJ
yjiK
yjiL
yjiM
yjiN
yjiO
yjiQ
yjiR
yjiS
yjiT
mcrC
mcrB
yjiW
hsdS
hsdM
hsdR
mrr
yjiA
yjiX
yjiY
tsr
yjiZ
4600 K
4605 K
4610 K
4615 K
4620 K
4625 K
4630 K
4635 K
4640 K
4645 K
4650 K
yjjN
mdoB
yjjA
dnaC
dnaT
yjjB
yjjP
yjjQ
bglJ
fhuF
leuQ
holD
rimI
yjjG
prfC
osmY
yjjU
ytjA
yjjV
yjjW
yjjI
deoC
deoA
deoB
deoD
yjjJ
lplA
ytjB
serB
radA
nadR
yjjK
slt
trpR
yjjX
ytjC
rob
creA
creB
creC
creD
arcA
yjjY
yjtD
