# Supplementary material for: H-NS Facilitates Sequence Diversification of Horizontally Transferred DNAs during Their Integration in Host Chromosomes
Source: PLoS Genet. 2016 Jan 20;12(1):e1005796. doi: 10.1371/journal.pgen.1005796 (PMC4720273; doi:10.1371/journal.pgen.1005796)
Supplement: S7 Fig — The H-NS binding profiles on the connected “common” segments in SE11, SE15, and K-12 are shown as for Fig 1C–1F. (PDF) [file pgen.1005796.s007.pdf]

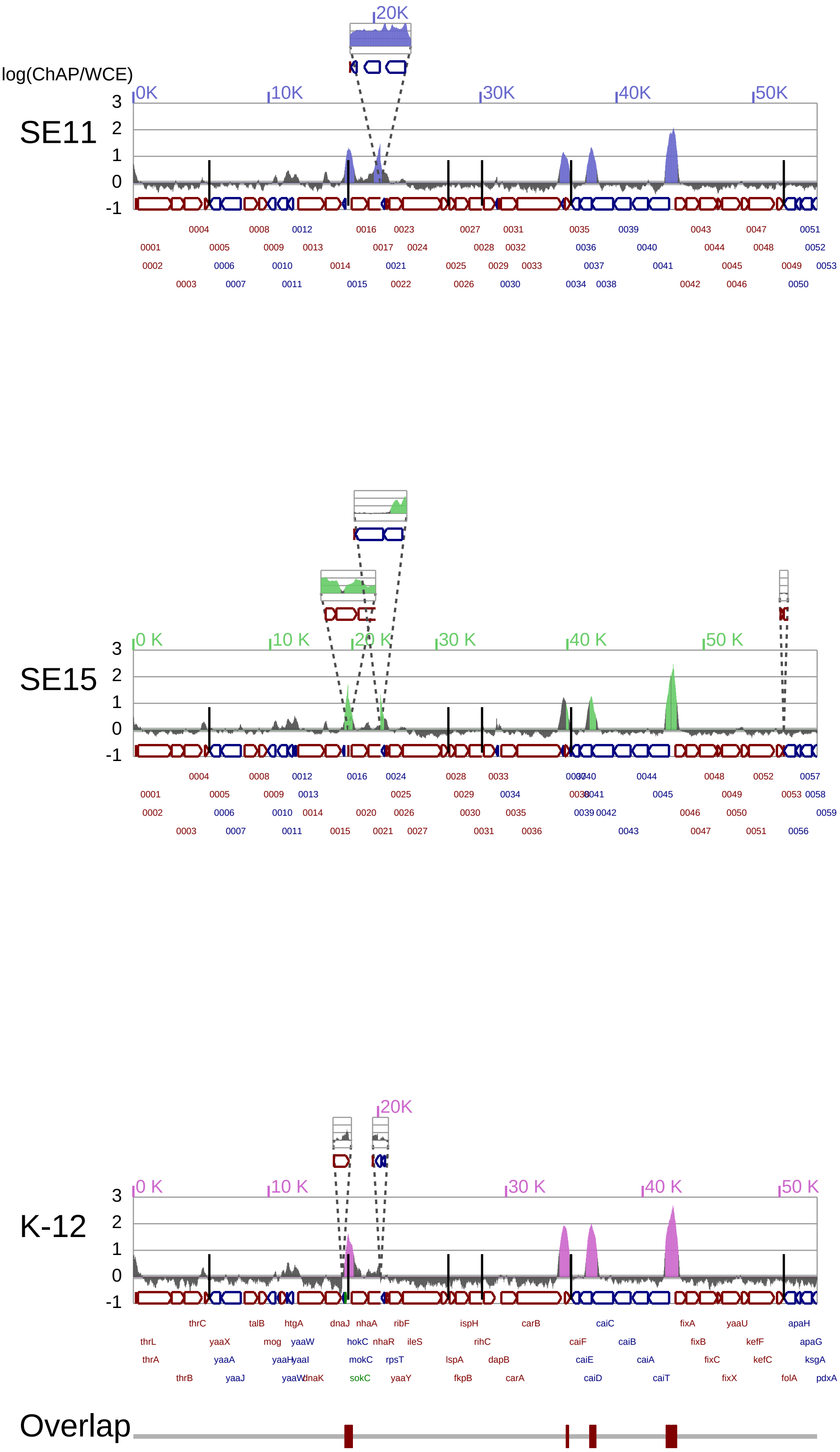
log(ChAP/WCE)
SE11
3
2
1
0
-1
0K
10K
20K
30K
40K
50K
0004 0008 0012 0016 0023 0027 0031 0035 0039 0043 0047 0051
0001 0005 0009 0013 0017 0024 0028 0032 0036 0040 0044 0048 0052
0002 0006 0010 0014 0021 0025 0029 0033 0037 0041 0045 0049 0053
0003 0007 0011 0015 0022 0026 0030 0034 0038 0042 0046 0050
SE15
3
2
1
0
-1
0 K
10 K
20 K
30 K
40 K
50 K
0004 0008 0012 0016 0024 0028 0033 0037 0040 0044 0048 0052 0057
0001 0005 0009 0013 0025 0029 0034 0038 0041 0045 0049 0053 0058
0002 0006 0010 0014 0020 0026 0030 0035 0039 0042 0046 0050 0059
0003 0007 0011 0015 0021 0027 0031 0036 0043 0047 0051 0056
K-12
3
2
1
0
-1
20K
0 K
10 K
30 K
40 K
50 K
thrC talB htgA dnaJ nhaA ribF ispH carB caiC fixA yaaU apaH
thrL yaaX mog yaaW hokC nhaR ileS rihC caiF caiB fixB kefF apaG
thrA yaaA yaaH yaaI mokC rpsT lspA dapB caiE caiA fixC kefC ksgA
thrB yaaJ yaaW dnaK sokC yaaY fkpB carA caiD caiT fixX folA pdxA
Overlap

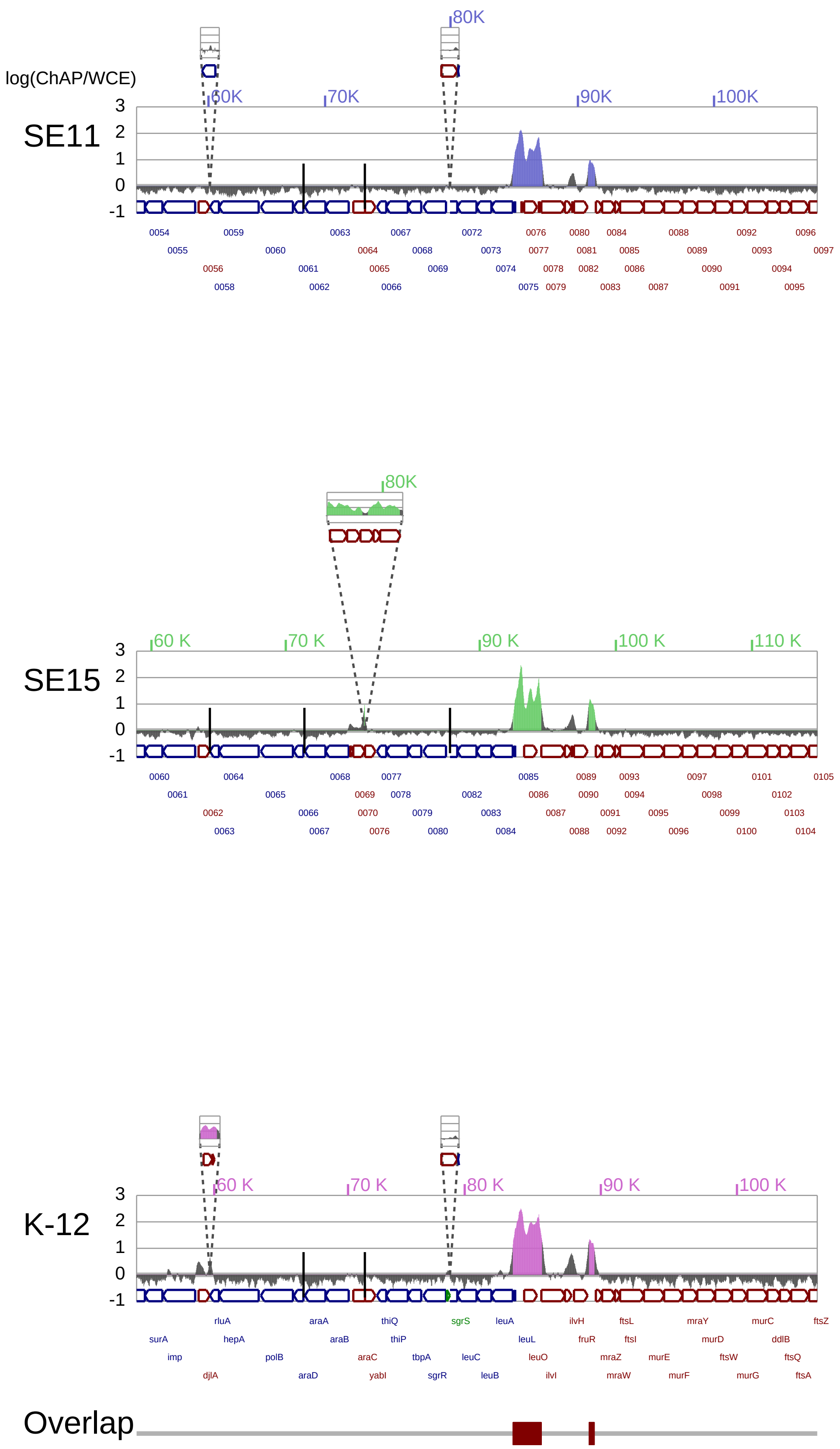
log(ChAP/WCE)
SE11
80K
60K
70K
90K
100K
3
2
1
0
-1
0054 0055 0056 0058 0059 0060 0061 0062 0063 0064 0065 0066 0067 0068 0069 0072 0073 0074 0075 0076 0077 0078 0079 0080 0081 0082 0083 0084 0085 0086 0087 0088 0089 0090 0091 0092 0093 0094 0095 0096 0097
SE15
80K
60 K
70 K
90 K
100 K
110 K
0060 0061 0062 0063 0064 0065 0066 0067 0068 0069 0070 0076 0077 0078 0079 0080 0082 0083 0084 0085 0086 0087 0088 0089 0090 0091 0092 0093 0094 0095 0096 0097 0098 0099 0100 0101 0102 0103 0104 0105
K-12
60 K
70 K
80 K
90 K
100 K
surA imp djlA rluA hepA polB araA araB araD araC yabI thiQ thiP tbpA sgrR sgrS leuC leuA leuL leuB leuO ilvI ilvH fruR mraZ mraW ftsL ftsI murE murF mraY murD ftsW murG murC ddlB ftsQ ftsA ftsZ
Overlap

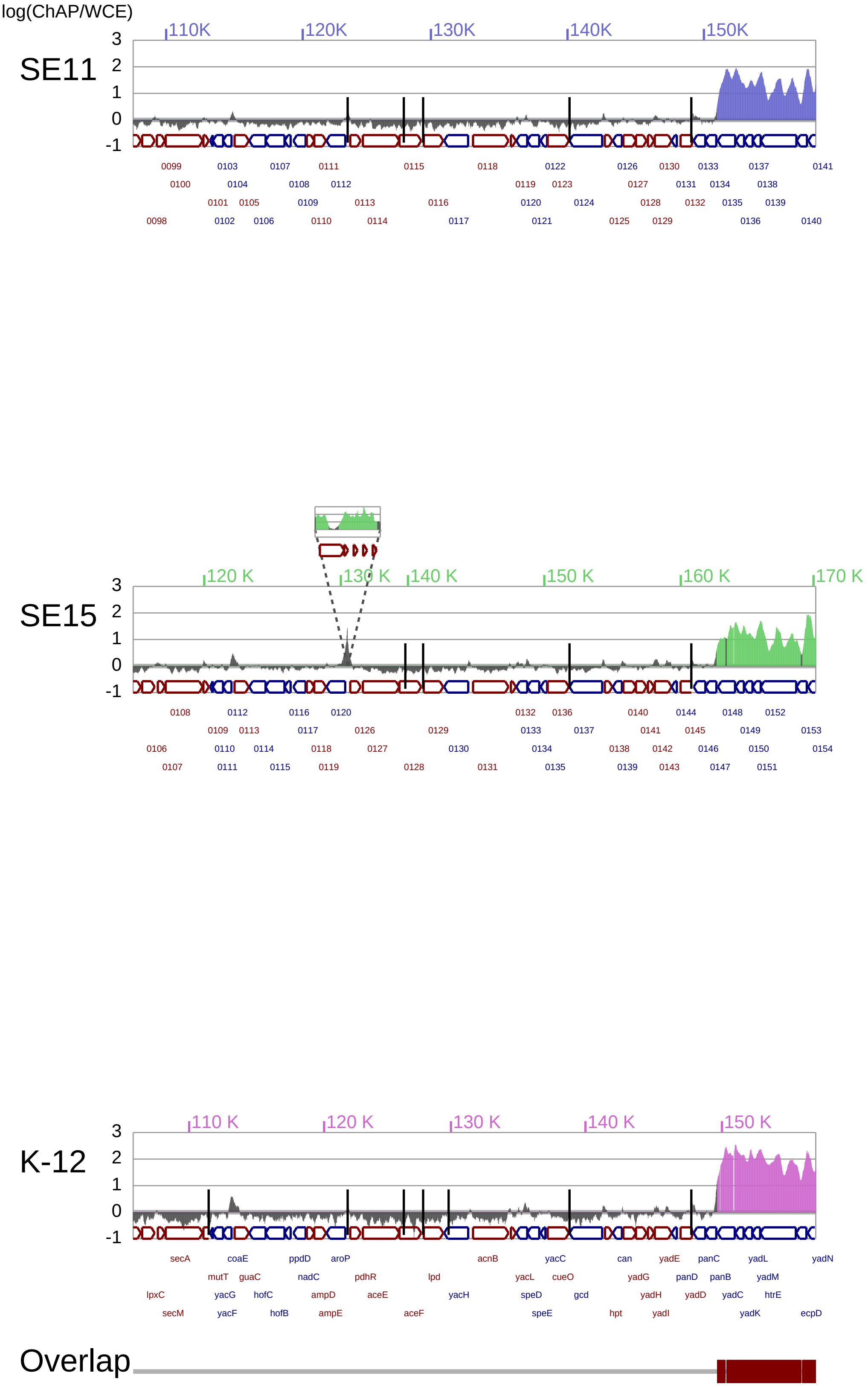

log(ChAP/WCE)
SE11
110K
120K
130K
140K
150K
3
2
1
0
-1
0099 0103 0107 0111 0115 0118 0122 0126 0130 0133 0137 0141
0100 0104 0108 0112 0119 0123 0127 0131 0134 0138
0101 0105 0109 0113 0116 0120 0124 0128 0132 0135 0139
0098 0102 0106 0110 0114 0117 0121 0125 0129 0136 0140
SE15
120 K
130 K
140 K
150 K
160 K
170 K
3
2
1
0
-1
0108 0112 0116 0120 0132 0136 0140 0144 0148 0152
0109 0113 0117 0126 0129 0133 0137 0141 0145 0149 0153
0106 0110 0114 0118 0127 0130 0134 0138 0142 0146 0150 0154
0107 0111 0115 0119 0128 0131 0135 0139 0143 0147 0151
K-12
110 K
120 K
130 K
140 K
150 K
3
2
1
0
-1
secA coaE ppdD aroP acnB yacC can yadE panC yadL yadN
mutT guaC nadC pdhR lpd yacL cueO yadG panD panB yadM
lpxC yacG hofC ampD aceE yacH speD gcd yadH yadD yadC htrE
secM yacF hofB ampE aceF speE hpt yadI yadK ecpD
Overlap

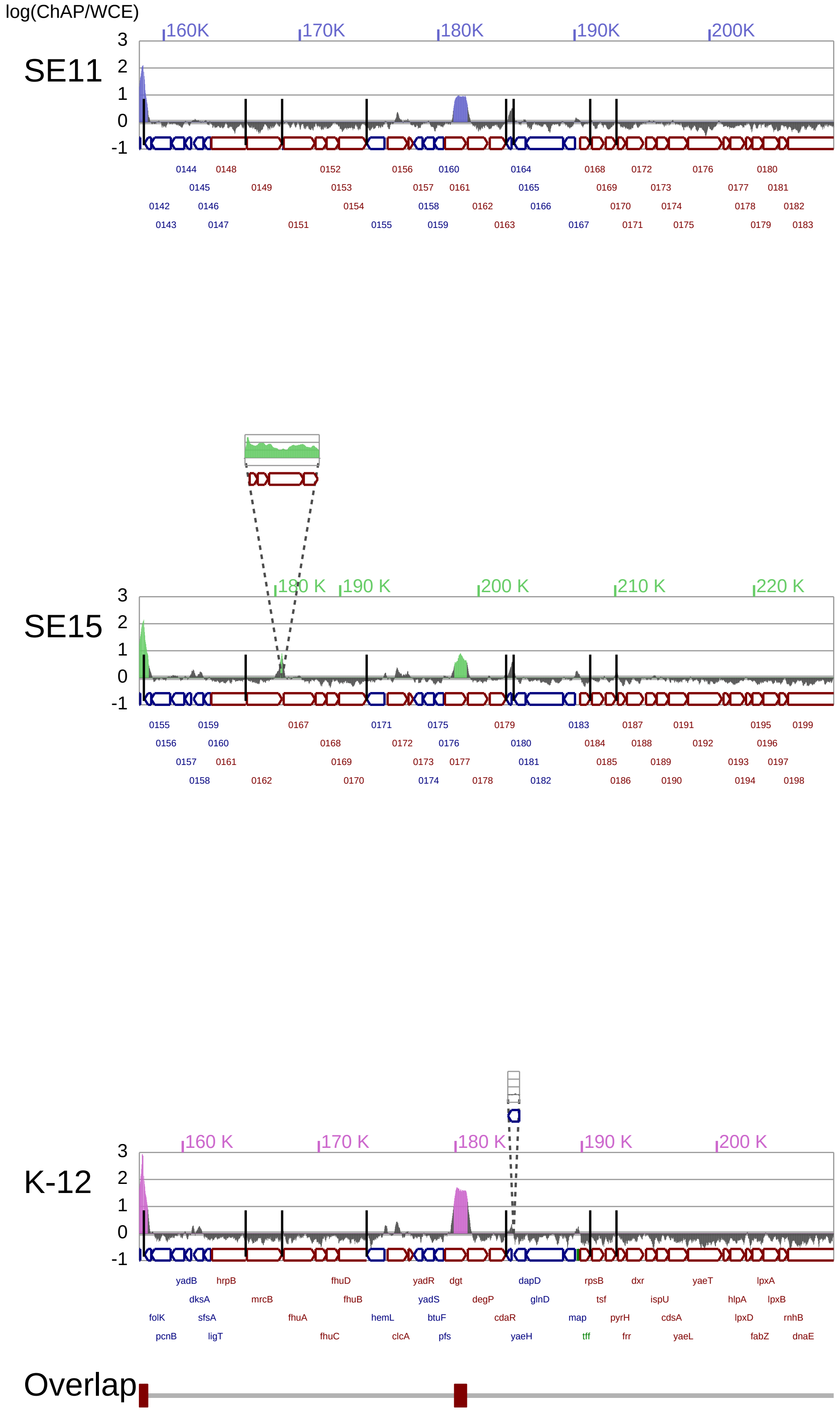
log(ChAP/WCE)
SE11
160K
170K
180K
190K
200K
3
2
1
0
-1
0144 0148 0152 0156 0160 0164 0168 0172 0176 0180
0145 0149 0153 0157 0161 0165 0169 0173 0177 0181
0142 0146 0154 0158 0162 0166 0170 0174 0178 0182
0143 0147 0151 0155 0159 0163 0167 0171 0175 0179 0183
SE15
180 K
190 K
200 K
210 K
220 K
0155 0159 0167 0171 0175 0179 0183 0187 0191 0195 0199
0156 0160 0168 0172 0176 0180 0184 0188 0192 0196
0157 0161 0169 0173 0177 0181 0185 0189 0193 0197
0158 0162 0170 0174 0178 0182 0186 0190 0194 0198
K-12
160 K
170 K
180 K
190 K
200 K
yadB hrpB fhuD yadR dgt dapD rpsB dxr yaeT lpxA
dksA mrcB fhuB yadS degP glnD tsf ispU hlpA lpxB
folK sfsA fhuA hemL btuF cdaR map pyrH cdsA lpxD rnhB
pcnB ligT fhuC clcA pfs yaeH tff frr yaeL fabZ dnaE
Overlap

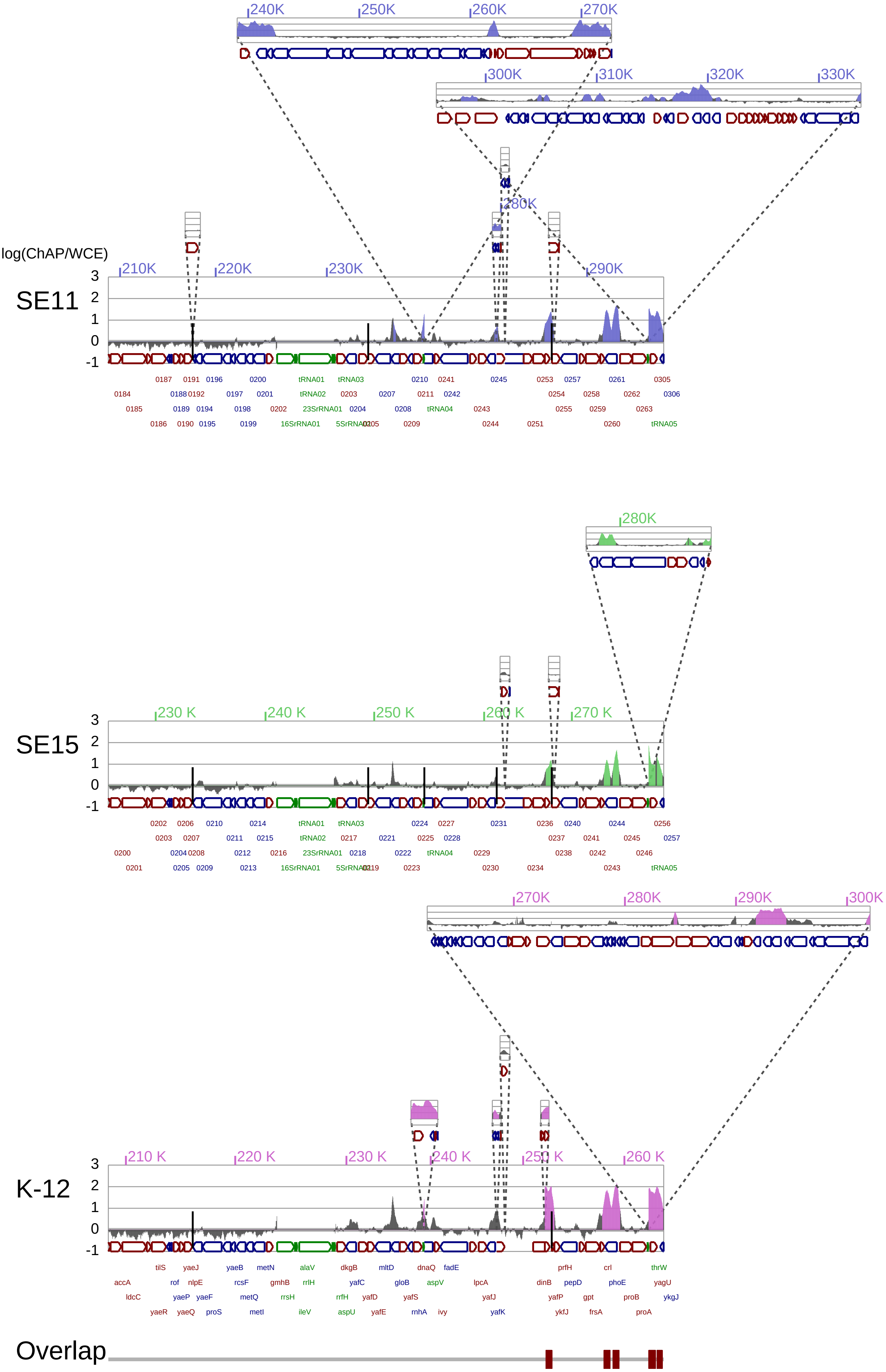
240K
250K
260K
270K
300K
310K
320K
330K
280K
log(ChAP/WCE)
210K
220K
230K
290K
SE11
3
2
1
0
-1
0187 0191 0196 0200 tRNA01 tRNA03 0210 0241 0245 0253 0257 0261 0305
0184 0188 0192 0197 0201 tRNA02 0203 0207 0211 0242 0254 0258 0262 0306
0185 0189 0194 0198 0202 23SrRNA01 0204 0208 tRNA04 0243 0255 0259 0263
0186 0190 0195 0199 16SrRNA01 5SrRNA01 0205 0209 0244 0251 0260 tRNA05
280K
230 K
240 K
250 K
260 K
270 K
SE15
3
2
1
0
-1
0202 0206 0210 0214 tRNA01 tRNA03 0224 0227 0231 0236 0240 0244 0256
0203 0207 0211 0215 tRNA02 0217 0221 0225 0228 0237 0241 0245 0257
0200 0204 0208 0212 0216 23SrRNA01 0218 0222 tRNA04 0229 0238 0242 0246
0201 0205 0209 0213 16SrRNA01 5SrRNA01 0219 0223 0230 0234 0243 tRNA05
270K
280K
290K
300K
210 K
220 K
230 K
240 K
250 K
260 K
K-12
3
2
1
0
-1
tilS yaeJ yaeB metN alaV dkgB mltD dnaQ fadE prfH crl thrW
accA rof nlpE rcsF gmhB rrlH yafC gloB aspV lpcA dinB pepD phoE yagU
ldcC yaeP yaeF metQ rrsH rrfH yafD yafS yafJ yafP gpt proB ykgJ
yaeR yaeQ proS metI ileV aspU yafE rnhA ivy yafK ykfJ frsA proA
Overlap

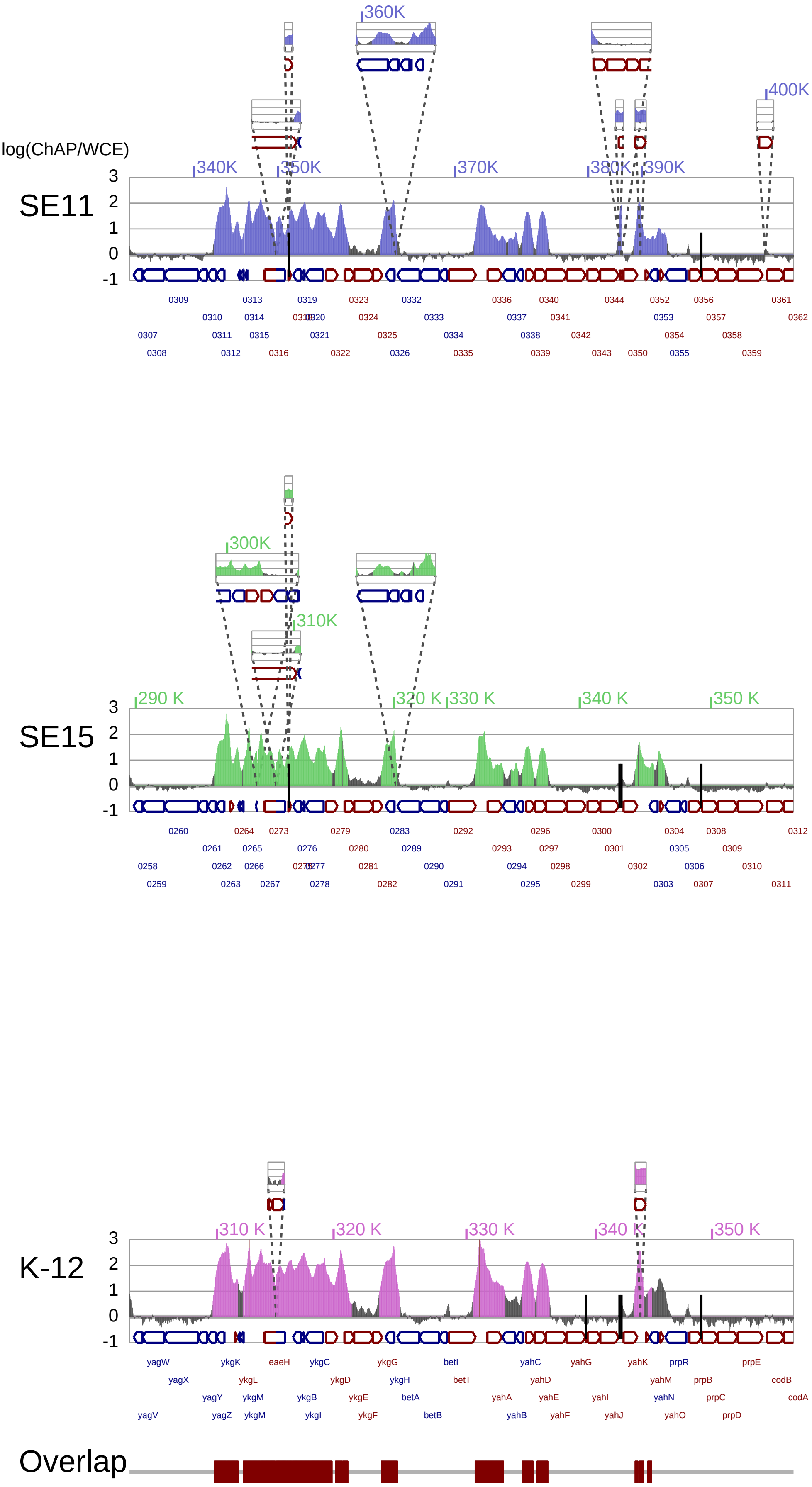

log(ChAP/WCE)
SE11
340K
350K
360K
370K
380K
390K
400K
3
2
1
0
-1
0309 0313 0319 0323 0332 0336 0340 0344 0352 0356 0361
0310 0314 0318 0320 0324 0333 0337 0341 0353 0357 0362
0307 0311 0315 0321 0325 0334 0338 0342 0354 0358
0308 0312 0316 0322 0326 0335 0339 0343 0350 0355 0359
SE15
290 K
300K
310K
320 K
330 K
340 K
350 K
0260 0264 0273 0279 0283 0292 0296 0300 0304 0308 0312
0261 0265 0276 0280 0289 0293 0297 0301 0305 0309
0258 0262 0266 0277 0281 0290 0294 0298 0302 0306 0310
0259 0263 0267 0278 0282 0291 0295 0299 0303 0307 0311
K-12
310 K
320 K
330 K
340 K
350 K
yagW ykgK eaeH ykgC ykgG betI yahC yahG yahK prpR prpE
yagX ykgL ykgD ykgH betT yahD yahM prpB codB
yagY ykgM ykgB ykgE betA yahA yahE yahI yahN prpC codA
yagV yagZ ykgM ykgI ykgF betB yahB yahF yahJ yahO prpD
Overlap

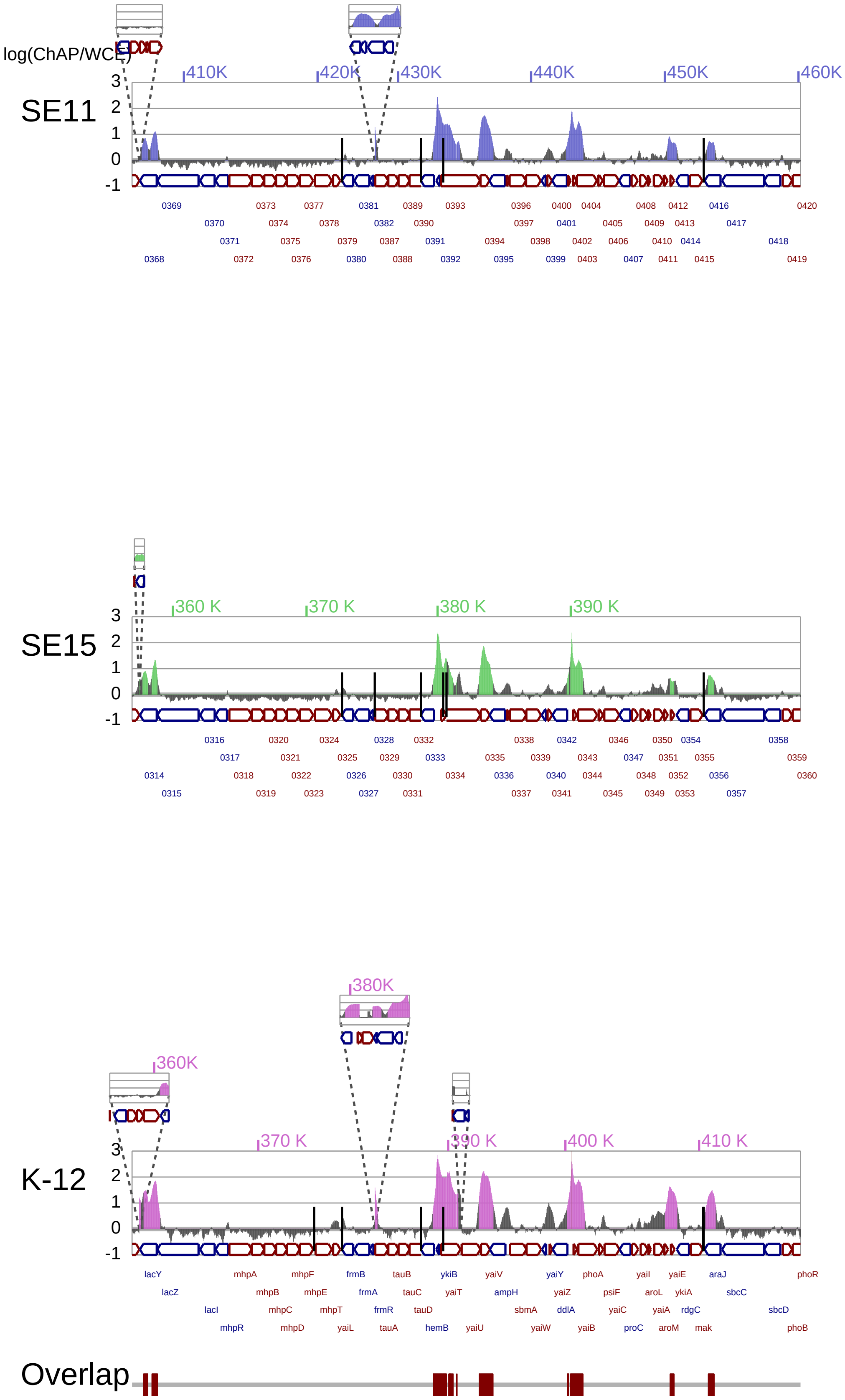

log(ChAP/WCE)
SE11
410K
420K
430K
440K
450K
460K
3
2
1
0
-1
0368 0369 0370 0371 0372 0373 0374 0375 0376 0377 0378 0379 0380 0381 0382 0387 0388 0389 0390 0391 0392 0393 0394 0395 0396 0397 0398 0399 0400 0401 0402 0403 0404 0405 0406 0407 0408 0409 0410 0411 0412 0413 0414 0415 0416 0417 0418 0419 0420
SE15
360 K
370 K
380 K
390 K
0314 0315 0316 0317 0318 0319 0320 0321 0322 0323 0324 0325 0326 0327 0328 0329 0330 0331 0332 0333 0334 0336 0337 0338 0339 0340 0341 0342 0343 0344 0345 0346 0347 0348 0349 0350 0351 0352 0353 0354 0355 0356 0357 0358 0359 0360
K-12
360K
380K
370 K
390 K
400 K
410 K
lacY lacZ lacI mhpR mhpA mhpB mhpC mhpD mhpF mhpE mhpT yaiL frmB frmA frmR tauA tauB tauC tauD hemB ykiB yaiT yaiU yaiV ampH sbmA yaiW yaiY yaiZ ddlA yaiB phoA psiF yaiC proC yaiI aroL yaiA aroM yaiE ykiA rdgC mak araJ sbcC sbcD phoB phoR
Overlap

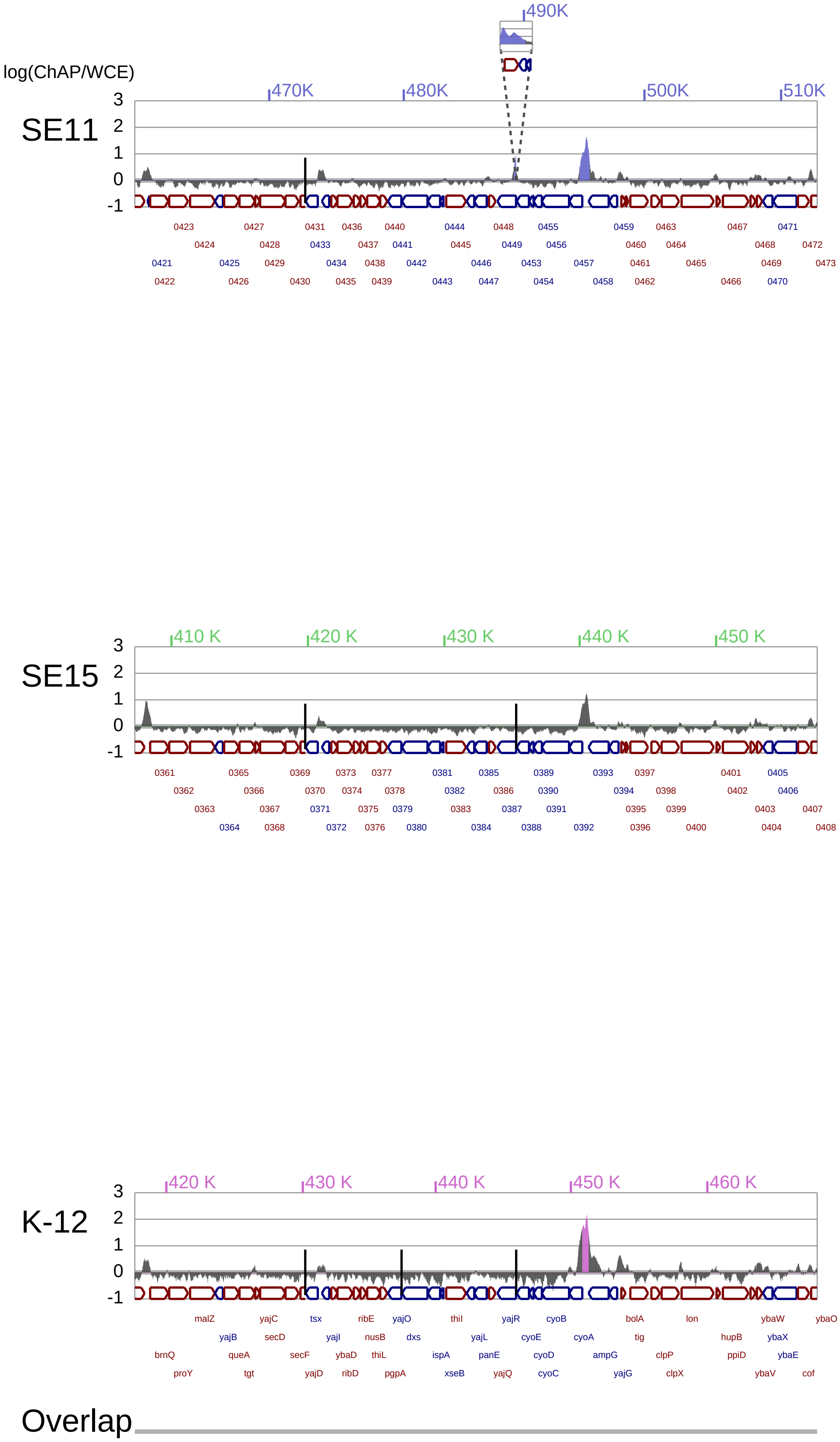

log(ChAP/WCE)
SE11
3
2
1
0
-1
470K
480K
490K
500K
510K
0423 0427 0431 0436 0440 0444 0448 0455 0459 0463 0467 0471
0424 0428 0433 0437 0441 0445 0449 0456 0460 0464 0468 0472
0421 0425 0429 0434 0438 0442 0446 0453 0457 0461 0465 0469 0473
0422 0426 0430 0435 0439 0443 0447 0454 0458 0462 0466 0470
SE15
3
2
1
0
-1
410 K
420 K
430 K
440 K
450 K
0361 0365 0369 0373 0377 0381 0385 0389 0393 0397 0401 0405
0362 0366 0370 0374 0378 0382 0386 0390 0394 0398 0402 0406
0363 0367 0371 0375 0379 0383 0387 0391 0395 0399 0403 0407
0364 0368 0372 0376 0380 0384 0388 0392 0396 0400 0404 0408
K-12
3
2
1
0
-1
420 K
430 K
440 K
450 K
460 K
malZ yajC tsx ribE yajO thiI yajR cyoB bolA lon ybaW ybaO
yajB secD yajI nusB dxs yajL cyoE cyoA tig hupB ybaX
brnQ queA secF ybaD thiL ispA panE cyoD ampG clpP ppiD ybaE
proY tgt yajD ribD pgpA xseB yajQ cyoC yajG clpX ybaV cof
Overlap

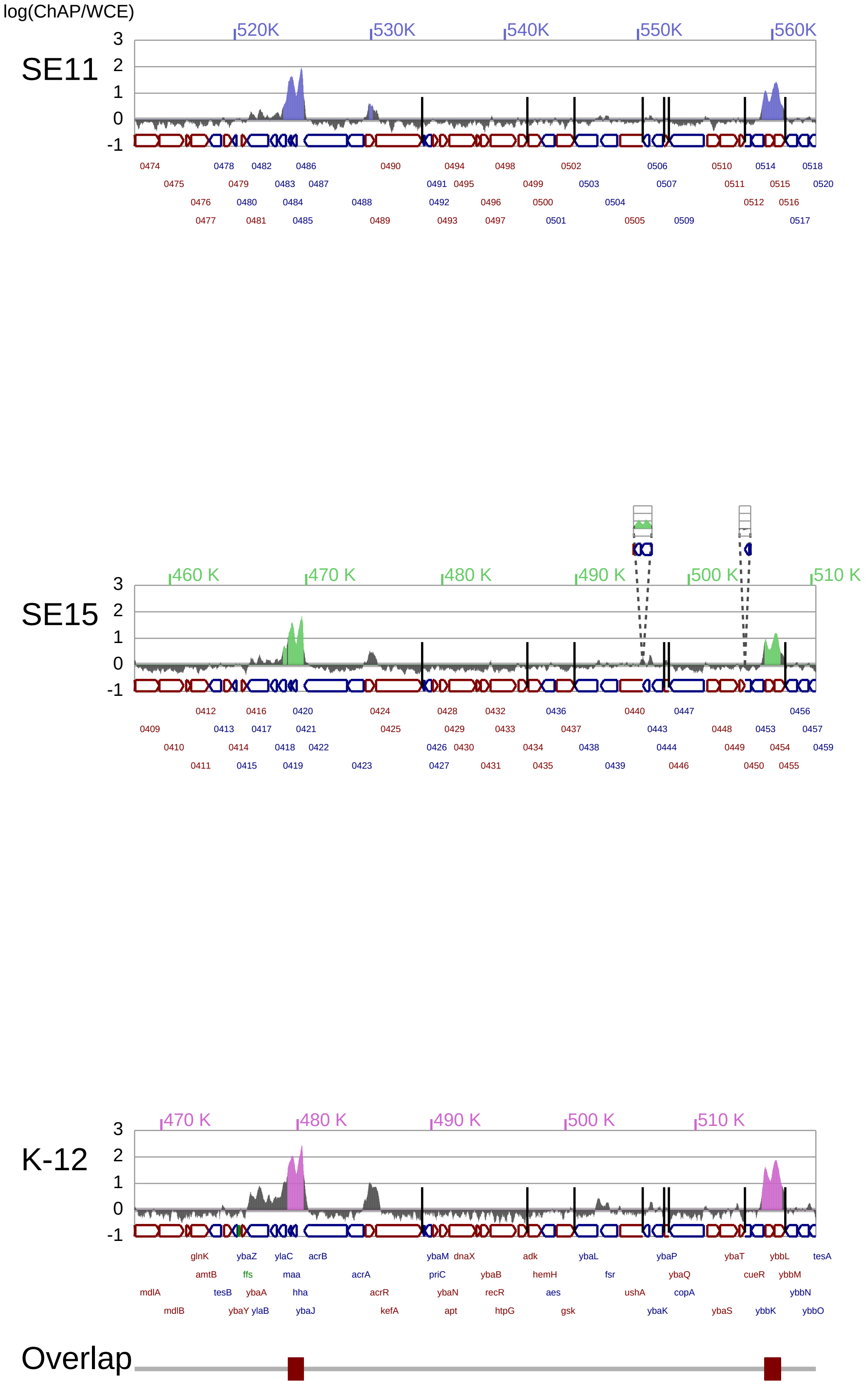
log(ChAP/WCE)
SE11
520K
530K
540K
550K
560K
3
2
1
0
-1
0474 0478 0482 0486 0490 0494 0498 0502 0506 0510 0514 0518
0475 0479 0483 0487 0491 0495 0499 0503 0507 0511 0515 0520
0476 0480 0484 0488 0492 0496 0500 0504 0512 0516
0477 0481 0485 0489 0493 0497 0501 0505 0509 0517
SE15
460 K
470 K
480 K
490 K
500 K
510 K
3
2
1
0
-1
0412 0416 0420 0424 0428 0432 0436 0440 0447 0456
0409 0413 0417 0421 0425 0429 0433 0437 0443 0448 0453 0457
0410 0414 0418 0422 0426 0430 0434 0438 0444 0449 0454 0459
0411 0415 0419 0423 0427 0431 0435 0439 0446 0450 0455
K-12
470 K
480 K
490 K
500 K
510 K
3
2
1
0
-1
glnK ybaZ ylaC acrB ybaM dnaX adk ybaL ybaP ybaT ybbL tesA
amtB ffs maa acrA priC ybaB hemH fsr ybaQ cueR ybbM
mdlA tesB ybaA hha acrR ybaN recR aes ushA copA ybbN
mdlB ybaY ylaB ybaJ kefA apt htpG gsk ybaK ybaS ybbK ybbO
Overlap

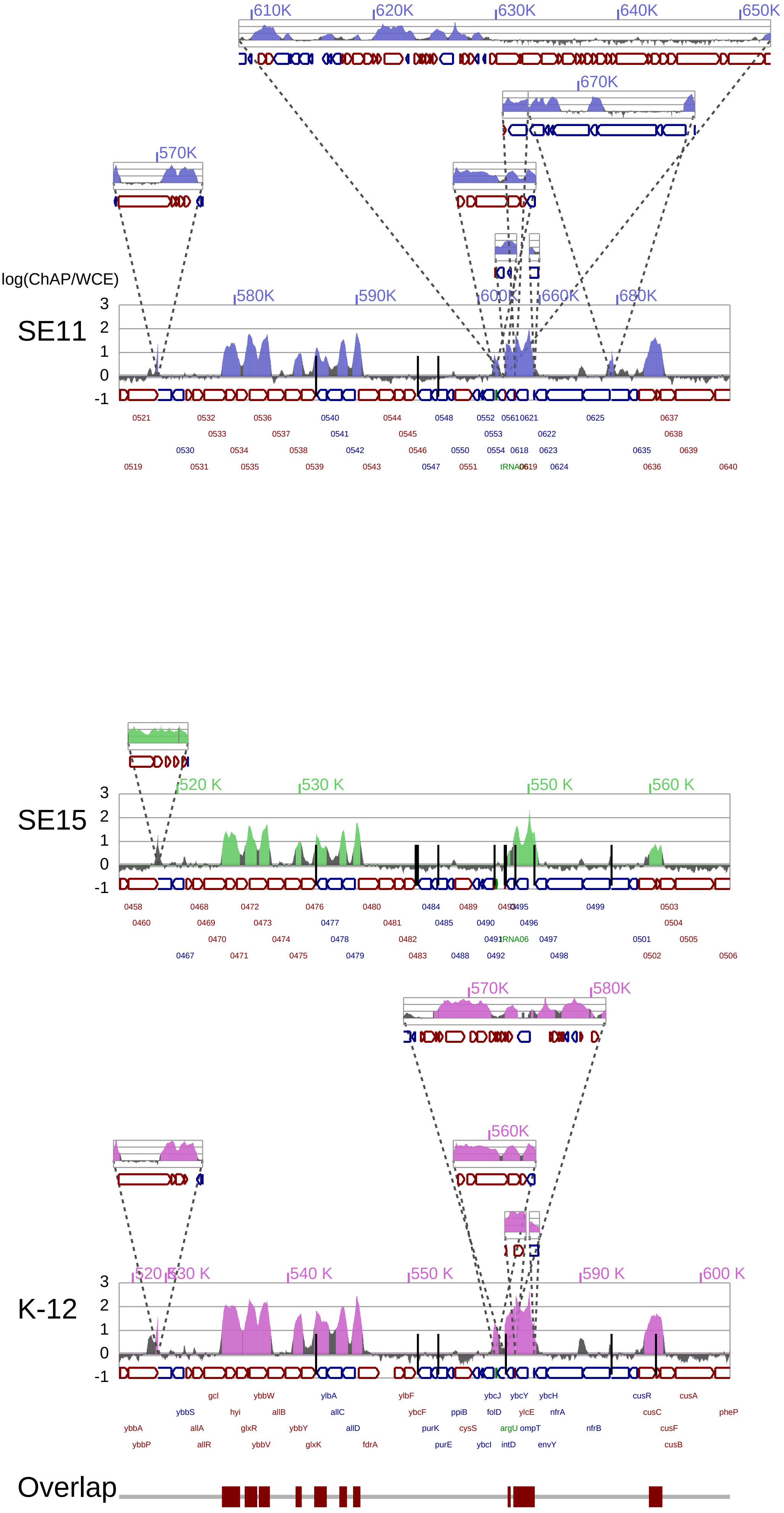

log(ChAP/WCE)
SE11
610K
620K
630K
640K
650K
670K
570K
580K
590K
600K
660K
680K
3
2
1
0
-1
0521
0532
0536
0540
0544
0548
0552
0561
0621
0625
0637
0533
0537
0541
0545
0553
0622
0638
0530
0534
0538
0542
0546
0550
0554
0618
0623
0635
0639
0519
0531
0535
0539
0543
0547
0551
tRNA06
0619
0624
0636
0640
SE15
520 K
530 K
550 K
560 K
0458
0468
0472
0476
0480
0484
0489
0493
0495
0499
0503
0460
0469
0473
0477
0481
0485
0490
0496
0504
0470
0474
0478
0482
0491
tRNA06
0497
0501
0505
0467
0471
0475
0479
0483
0488
0492
0498
0502
0506
K-12
570K
580K
560K
520 K
530 K
540 K
550 K
590 K
600 K
gcl
ybbW
ylbA
ylbF
ybcJ
ybcY
ybcH
cusR
cusA
ybbS
hyi
allB
allC
ybcF
ppiB
folD
ylcE
nfrA
cusC
pheP
ybbA
allA
glxR
ybbY
allD
purK
cysS
argU
ompT
nfrB
cusF
ybbP
allR
ybbV
glxK
fdrA
purE
ybcI
intD
envY
cusB
Overlap

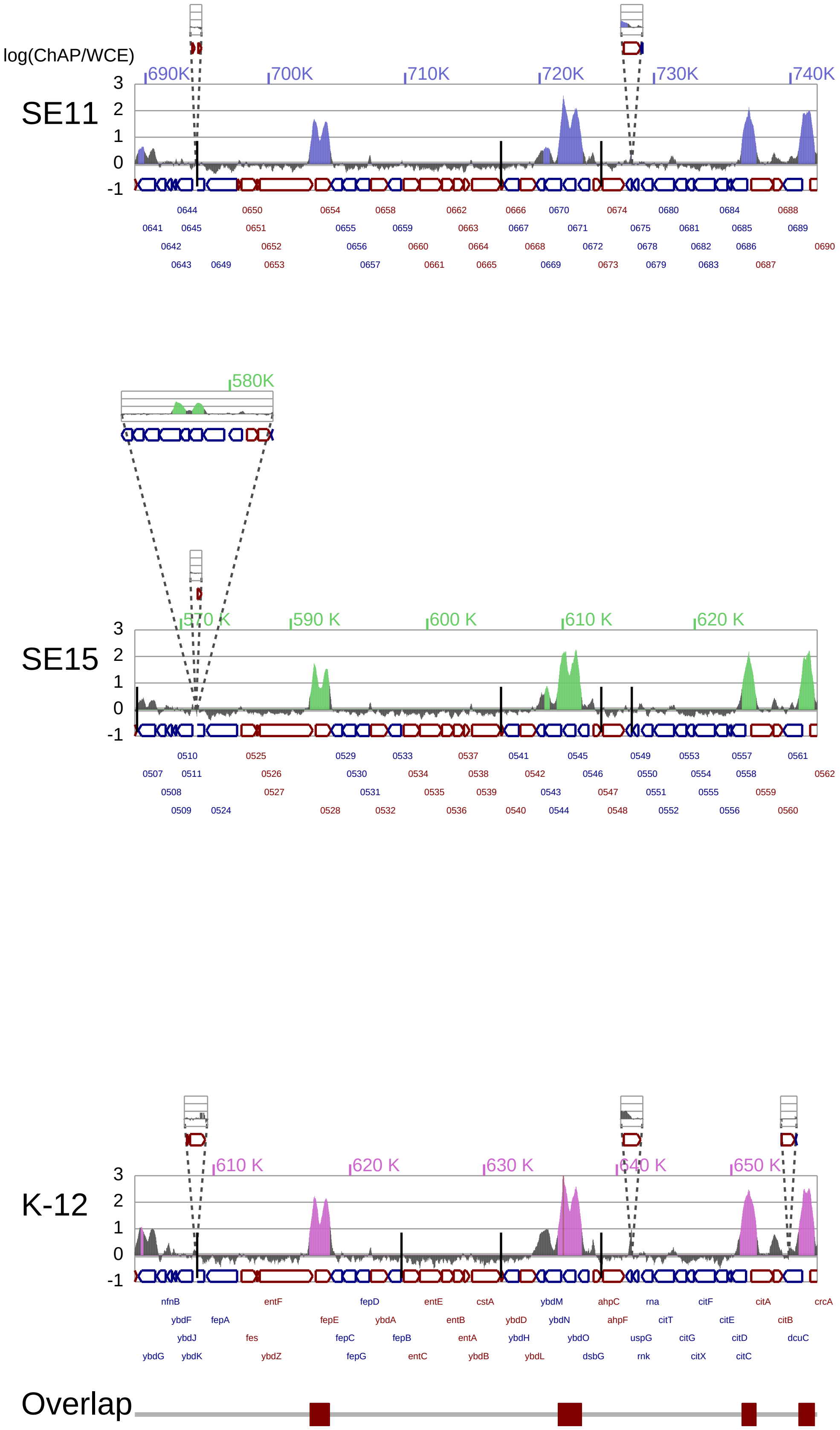
log(ChAP/WCE)
SE11
690K
700K
710K
720K
730K
740K
3
2
1
0
-1
0644 0650 0654 0658 0662 0666 0670 0674 0680 0684 0688
0641 0645 0651 0655 0659 0663 0667 0671 0675 0681 0685 0689
0642 0652 0656 0660 0664 0668 0672 0678 0682 0686 0690
0643 0649 0653 0657 0661 0665 0669 0673 0679 0683 0687
580K
SE15
570 K
590 K
600 K
610 K
620 K
0510 0525 0529 0533 0537 0541 0545 0549 0553 0557 0561
0507 0511 0526 0530 0534 0538 0542 0546 0550 0554 0558 0562
0508 0527 0531 0535 0539 0543 0547 0551 0555 0559
0509 0524 0528 0532 0536 0540 0544 0548 0552 0556 0560
K-12
610 K
620 K
630 K
640 K
650 K
nfnB entF fepD entE cstA ybdM ahpC rna citF citA crcA
ybdF fepA fepE ybdA entB ybdD ybdN ahpF citT citE citB
ybdJ fes fepC fepB entA ybdH ybdO uspG citG citD dcuC
ybdG ybdK ybdZ fepG entC ybdB ybdL dsbG rnk citX citC
Overlap

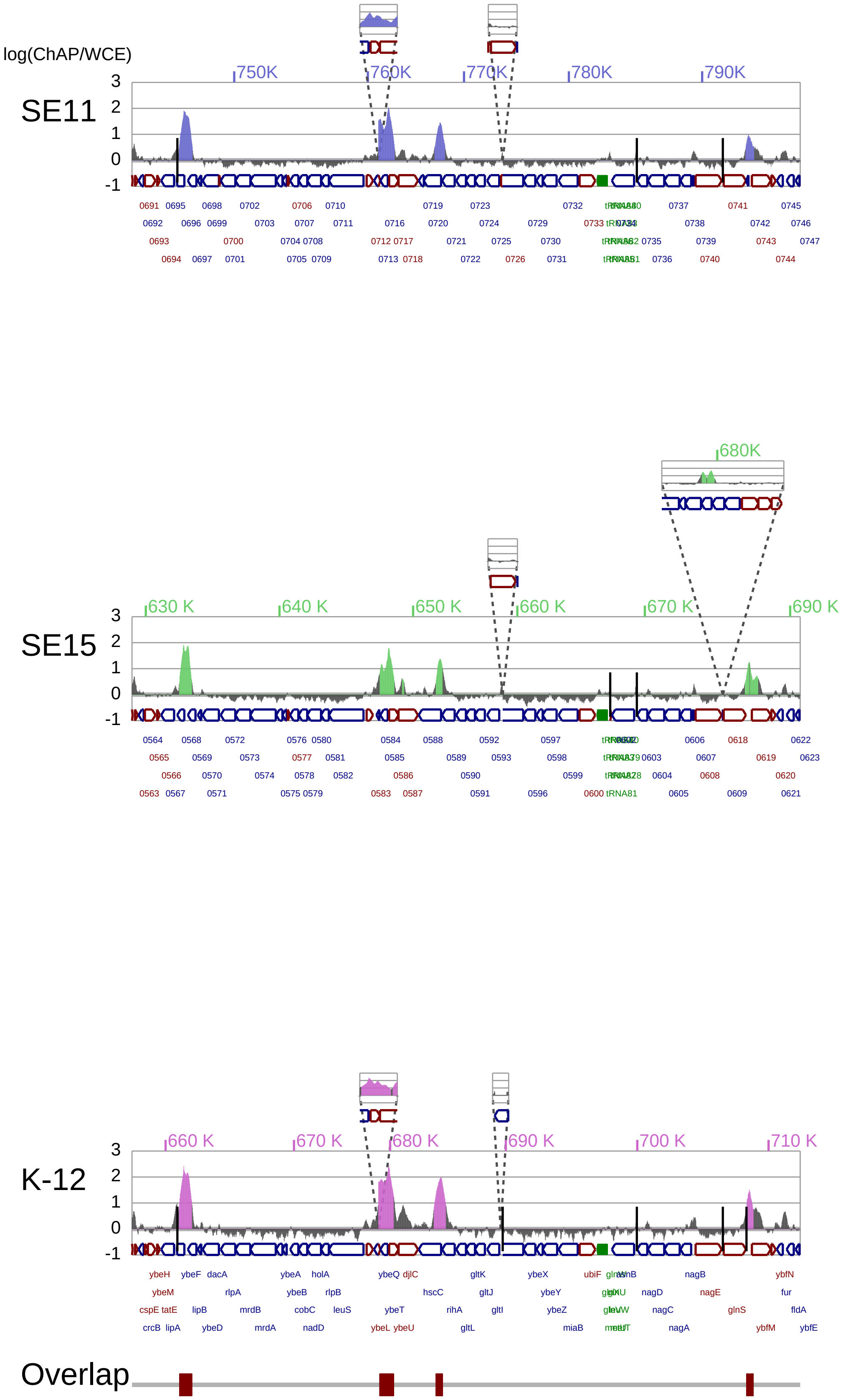

log(ChAP/WCE)
SE11
750K
760K
770K
780K
790K
3
2
1
0
-1
0691 0695 0698 0702 0706 0710 0719 0723 0732 0737 0741 0745
0692 0696 0699 0703 0707 0711 0716 0720 0724 0729 0733 0738 0742 0746
0693 0700 0704 0708 0712 0717 0721 0725 0730 0735 0739 0743 0747
0694 0697 0701 0705 0709 0713 0718 0722 0726 0731 0736 0740 0744
SE15
680K
630 K
640 K
650 K
660 K
670 K
690 K
0564 0568 0572 0576 0580 0584 0588 0592 0597 0606 0618 0622
0565 0569 0573 0577 0581 0585 0589 0593 0598 0603 0607 0619 0623
0566 0570 0574 0578 0582 0586 0590 0599 0604 0608 0620
0563 0567 0571 0575 0579 0583 0587 0591 0596 0600 tRNA81 0605 0609 0621
K-12
660 K
670 K
680 K
690 K
700 K
710 K
ybeH ybeF dacA ybeA holA ybeQ djlC gltK ybeX ubiF nagB ybfN
ybeM rlpA ybeB rlpB hscC gltJ ybeY nagD nagE fur
cspE tatE lipB mrdB cobC leuS ybeT rihA gltI ybeZ nagC glnS fldA
crcB lipA ybeD mrdA nadD ybeL ybeU gltL miaB nagA ybfM ybfE
Overlap

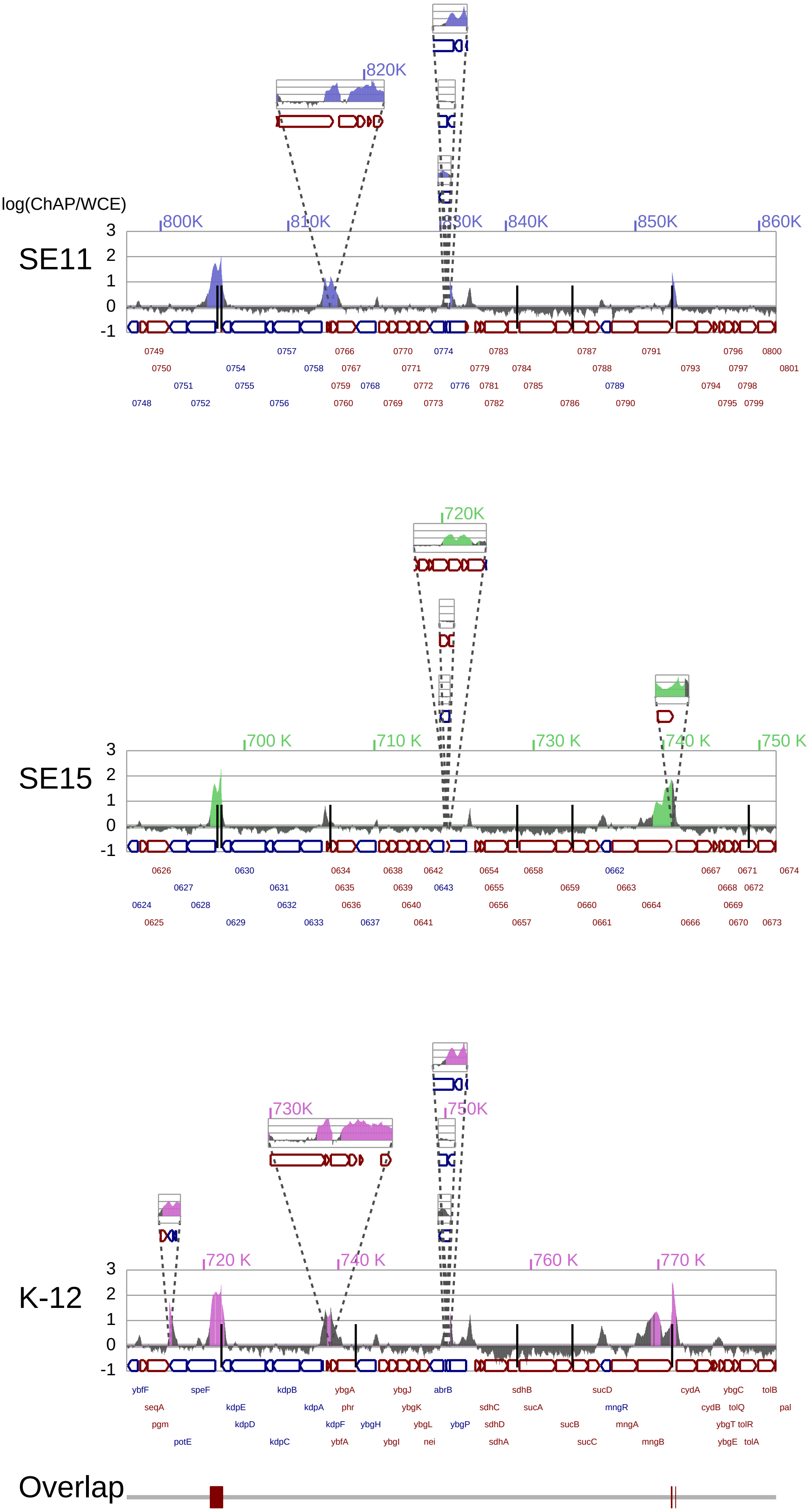

log(ChAP/WCE)
SE11
800K
810K
820K
830K
840K
850K
860K
0749 0750 0751 0748 0752 0754 0755 0756 0757 0758 0759 0760 0766 0767 0768 0769 0770 0771 0772 0773 0774 0776 0779 0781 0782 0783 0784 0785 0786 0787 0788 0789 0790 0791 0793 0794 0795 0796 0797 0798 0799 0800 0801
SE15
700 K
710 K
720K
730 K
740 K
750 K
0624 0625 0626 0627 0628 0629 0630 0631 0632 0633 0634 0635 0636 0637 0638 0639 0640 0641 0642 0643 0654 0655 0656 0657 0658 0659 0660 0661 0662 0663 0664 0666 0667 0668 0669 0670 0671 0672 0673 0674
K-12
720 K
730K
740 K
750K
760 K
770 K
ybfF seqA pgm potE speF kdpE kdpD kdpB kdpC kdpA kdpF ybfA ybgA phr ybgH ybgJ ybgK ybgI ybgL nei abrB ybgP sdhC sdhD sdhA sdhB sucA sucB sucC sucD mngR mngA mngB cydA cydB ybgC tolQ ybgT tolR ybgE tolA tolB pal
Overlap

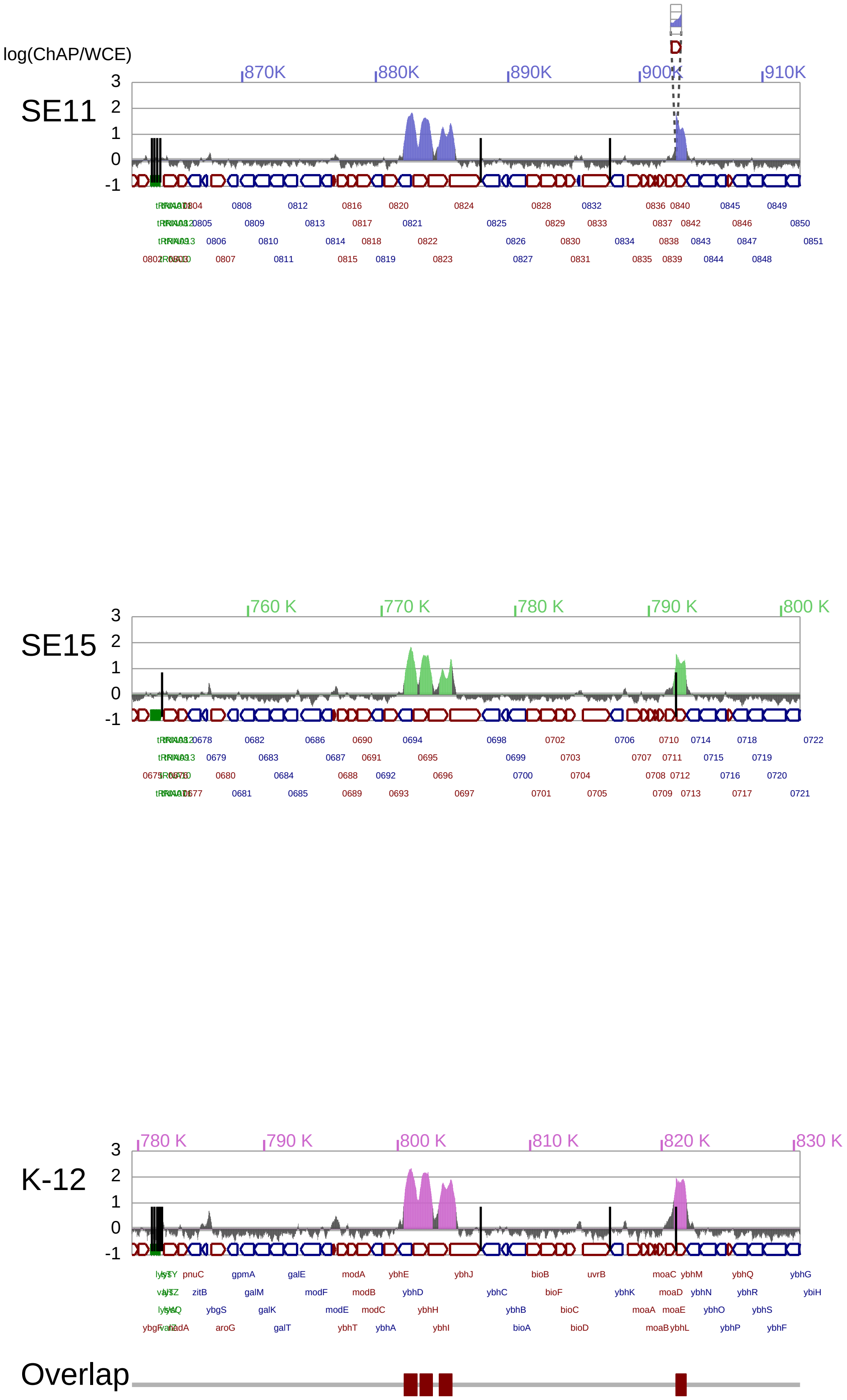

log(ChAP/WCE)
SE11
3
2
1
0
-1
870K
880K
890K
900K
910K
0808 0812 0816 0820 0824 0828 0832 0836 0840 0845 0849
0805 0809 0813 0817 0821 0825 0829 0833 0837 0842 0846 0850
0806 0810 0814 0818 0822 0826 0830 0834 0838 0843 0847 0851
0807 0811 0815 0819 0823 0827 0831 0835 0839 0844 0848
SE15
760 K
770 K
780 K
790 K
800 K
0678 0682 0686 0690 0694 0698 0702 0706 0710 0714 0718 0722
0679 0683 0687 0691 0695 0699 0703 0707 0711 0715 0719
0680 0684 0688 0692 0696 0700 0704 0708 0712 0716 0720
0677 0681 0685 0689 0693 0697 0701 0705 0709 0713 0717 0721
K-12
780 K
790 K
800 K
810 K
820 K
830 K
pnuC gpmA galE modA ybhE ybhJ bioB uvrB moaC ybhM ybhQ ybhG
zitB galM modF modB ybhD ybhC bioF ybhK moaD ybhN ybhR ybiH
ybgS galK modE modC ybhH ybhB bioC moaA moaE ybhO ybhS
nadA aroG galT ybhT ybhA ybhI bioA bioD moaB ybhL ybhP ybhF
Overlap

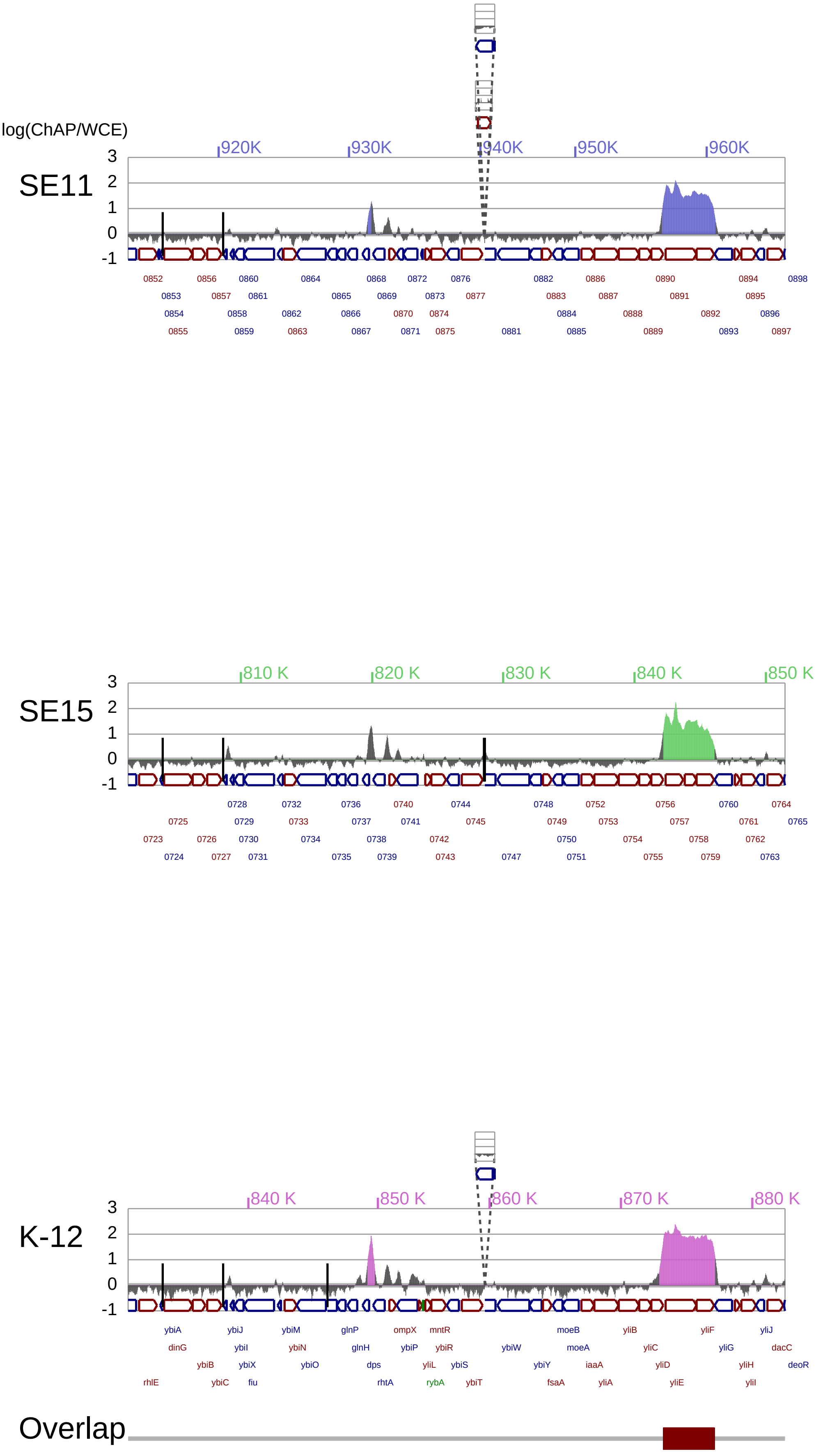

log(ChAP/WCE)
SE11
920K
930K
940K
950K
960K
3
2
1
0
-1
0852 0856 0860 0864 0868 0872 0876 0882 0886 0890 0894 0898
0853 0857 0861 0865 0869 0873 0877 0883 0887 0891 0895
0854 0858 0862 0866 0870 0874 0884 0888 0892 0896
0855 0859 0863 0867 0871 0875 0881 0885 0889 0893 0897
SE15
810 K
820 K
830 K
840 K
850 K
3
2
1
0
-1
0728 0732 0736 0740 0744 0748 0752 0756 0760 0764
0725 0729 0733 0737 0741 0745 0749 0753 0757 0761 0765
0723 0726 0730 0734 0738 0742 0750 0754 0758 0762
0724 0727 0731 0735 0739 0743 0747 0751 0755 0759 0763
K-12
840 K
850 K
860 K
870 K
880 K
3
2
1
0
-1
ybiA ybiJ ybiM glnP ompX mntR moeB yliB yliF yliJ
dinG ybiI ybiN glnH ybiP ybiR ybiW moeA yliC yliG dacC
ybiB ybiX ybiO dps yliL ybiS ybiY iaaA yliD yliH deoR
rhlE ybiC fiu rhtA rybA ybiT fsaA yliA yliE yliI
Overlap

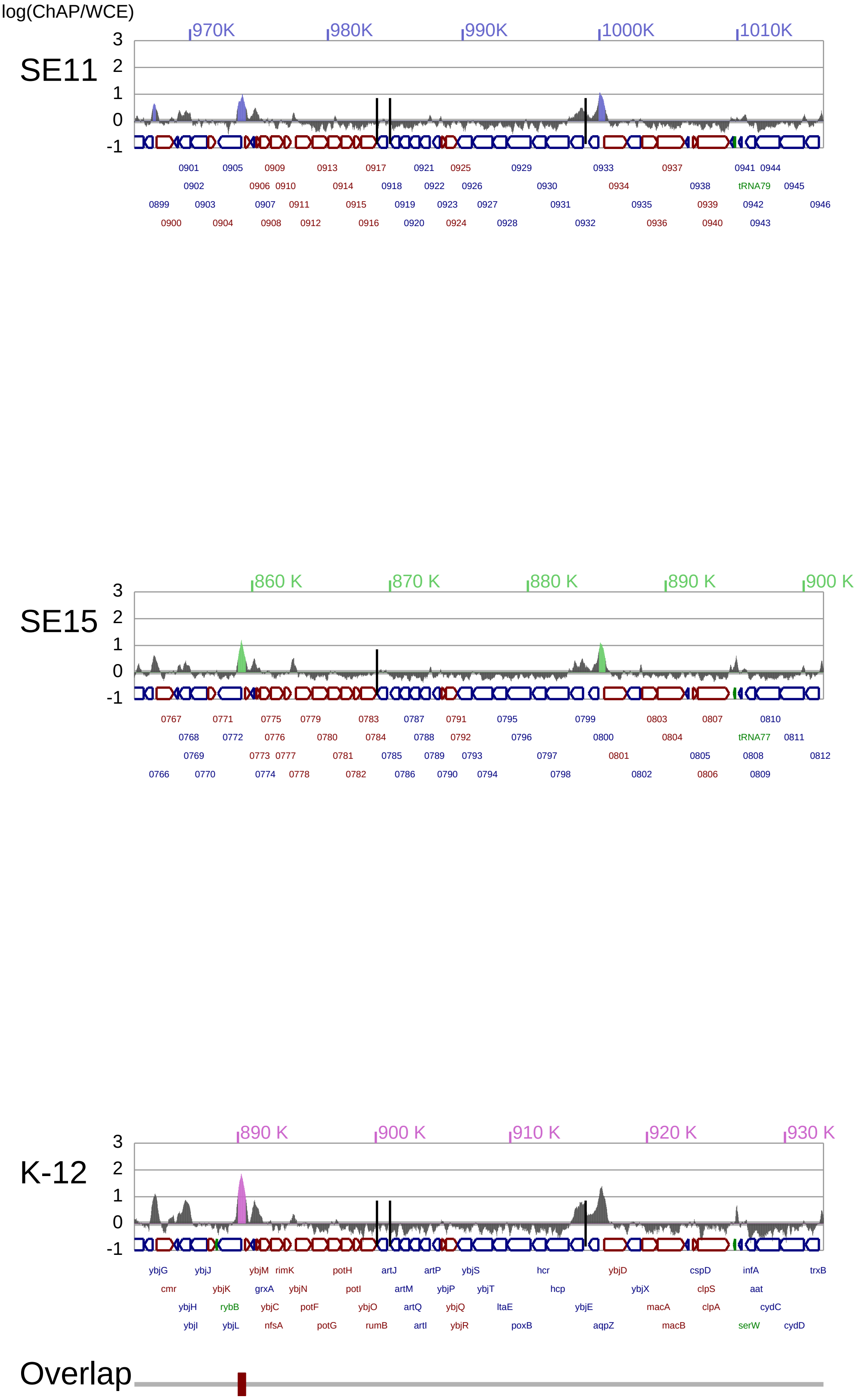
log(ChAP/WCE)
SE11
970K
980K
990K
1000K
1010K
3
2
1
0
-1
0901 0905 0909 0913 0917 0921 0925 0929 0933 0937 0941 0944
0902 0906 0910 0914 0918 0922 0926 0930 0934 0938 tRNA79 0945
0899 0903 0907 0911 0915 0919 0923 0927 0931 0935 0939 0942 0946
0900 0904 0908 0912 0916 0920 0924 0928 0932 0936 0940 0943
SE15
860 K
870 K
880 K
890 K
900 K
3
2
1
0
-1
0767 0771 0775 0779 0783 0787 0791 0795 0799 0803 0807 0810
0768 0772 0776 0780 0784 0788 0792 0796 0800 0804 tRNA77 0811
0769 0773 0777 0781 0785 0789 0793 0797 0801 0805 0808 0812
0766 0770 0774 0778 0782 0786 0790 0794 0798 0802 0806 0809
K-12
890 K
900 K
910 K
920 K
930 K
3
2
1
0
-1
ybjG ybjJ ybjM rimK potH artJ artP ybjS hcr ybjD cspD infA trxB
cmr ybjK grxA ybjN potI artM ybjP ybjT hcp ybjX clpS aat
ybjH rybB ybjC potF ybjO artQ ybjQ ltaE ybjE macA clpA cydC
ybjI ybjL nfsA potG rumB artI ybjR poxB aqpZ macB serW cydD
Overlap

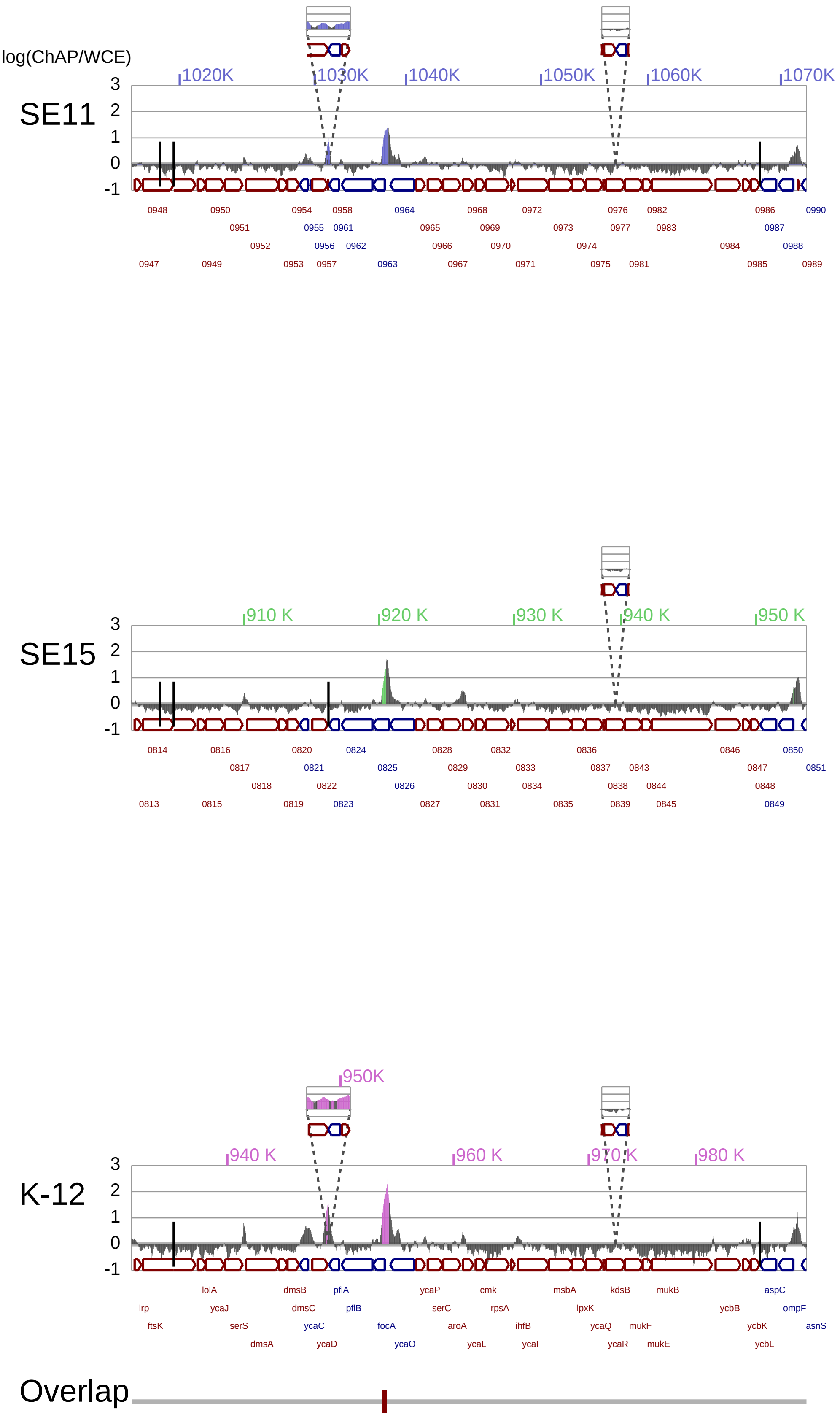

log(ChAP/WCE)
SE11
1020K
1030K
1040K
1050K
1060K
1070K
3
2
1
0
-1
0948 0950 0954 0958 0964 0968 0972 0976 0982 0986 0990
0951 0955 0961 0965 0969 0973 0977 0983 0987
0952 0956 0962 0966 0970 0974 0984 0988
0947 0949 0953 0957 0963 0967 0971 0975 0981 0985 0989
SE15
910 K
920 K
930 K
940 K
950 K
3
2
1
0
-1
0814 0816 0820 0824 0828 0832 0836 0846 0850
0817 0821 0825 0829 0833 0837 0843 0847 0851
0818 0822 0826 0830 0834 0838 0844 0848
0813 0815 0819 0823 0827 0831 0835 0839 0845 0849
K-12
950K
940 K
960 K
970 K
980 K
3
2
1
0
-1
lolA dmsB pflA ycaP cmk msbA kdsB mukB aspC
lrp ycaJ dmsC pflB serC rpsA lpxK ycbB ompF
ftsK serS ycaC focA aroA ihfB ycaQ mukF ycbK asnS
dmsA ycaD ycaO ycaL ycaI ycaR mukE ycbL
Overlap

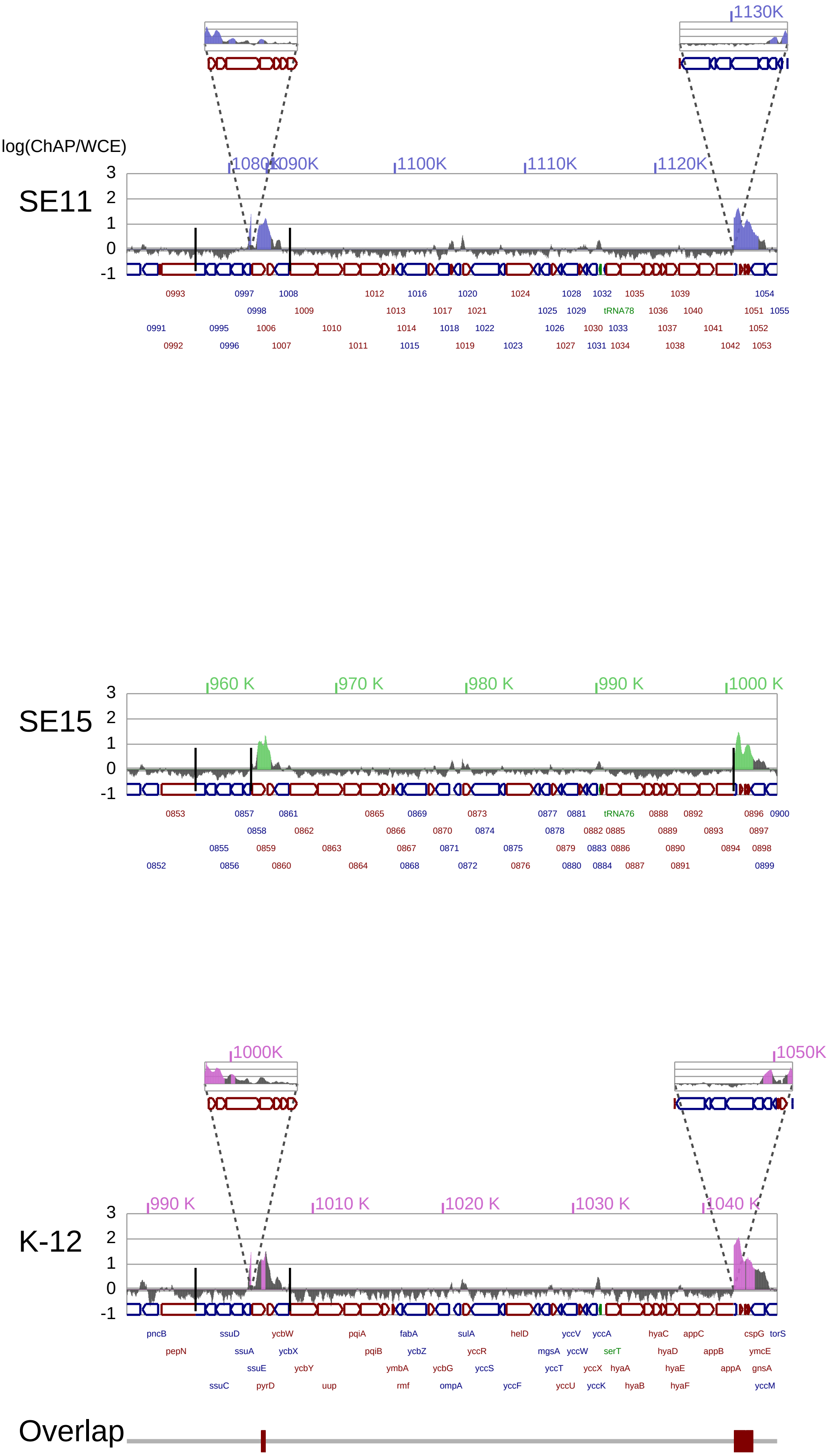

log(ChAP/WCE)
SE11
1080K
1090K
1100K
1110K
1120K
1130K
3
2
1
0
-1
0993 0997 1008 1012 1016 1020 1024 1028 1032 1035 1039 1054
0998 1009 1013 1017 1021 1025 1029 tRNA78 1036 1040 1051 1055
0991 0995 1006 1010 1014 1018 1022 1026 1030 1033 1037 1041 1052
0992 0996 1007 1011 1015 1019 1023 1027 1031 1034 1038 1042 1053
SE15
960 K
970 K
980 K
990 K
1000 K
0853 0857 0861 0865 0869 0873 0877 0881 tRNA76 0888 0892 0896 0900
0858 0862 0866 0870 0874 0878 0882 0885 0889 0893 0897
0855 0859 0863 0867 0871 0875 0879 0883 0886 0890 0894 0898
0852 0856 0860 0864 0868 0872 0876 0880 0884 0887 0891 0899
K-12
1000K
1050K
990 K
1010 K
1020 K
1030 K
1040 K
pncB ssuD ycbW pqiA fabA sulA helD yccV yccA hyaC appC cspG torS
pepN ssuA ycbX pqiB ycbZ yccR mgsA yccW serT hyaD appB ymcE
ssuE ycbY ymbA ycbG yccS yccT yccX hyaA hyaE appA gnsA
ssuC pyrD uup rmf ompA yccF yccU yccK hyaB hyaF yccM
Overlap

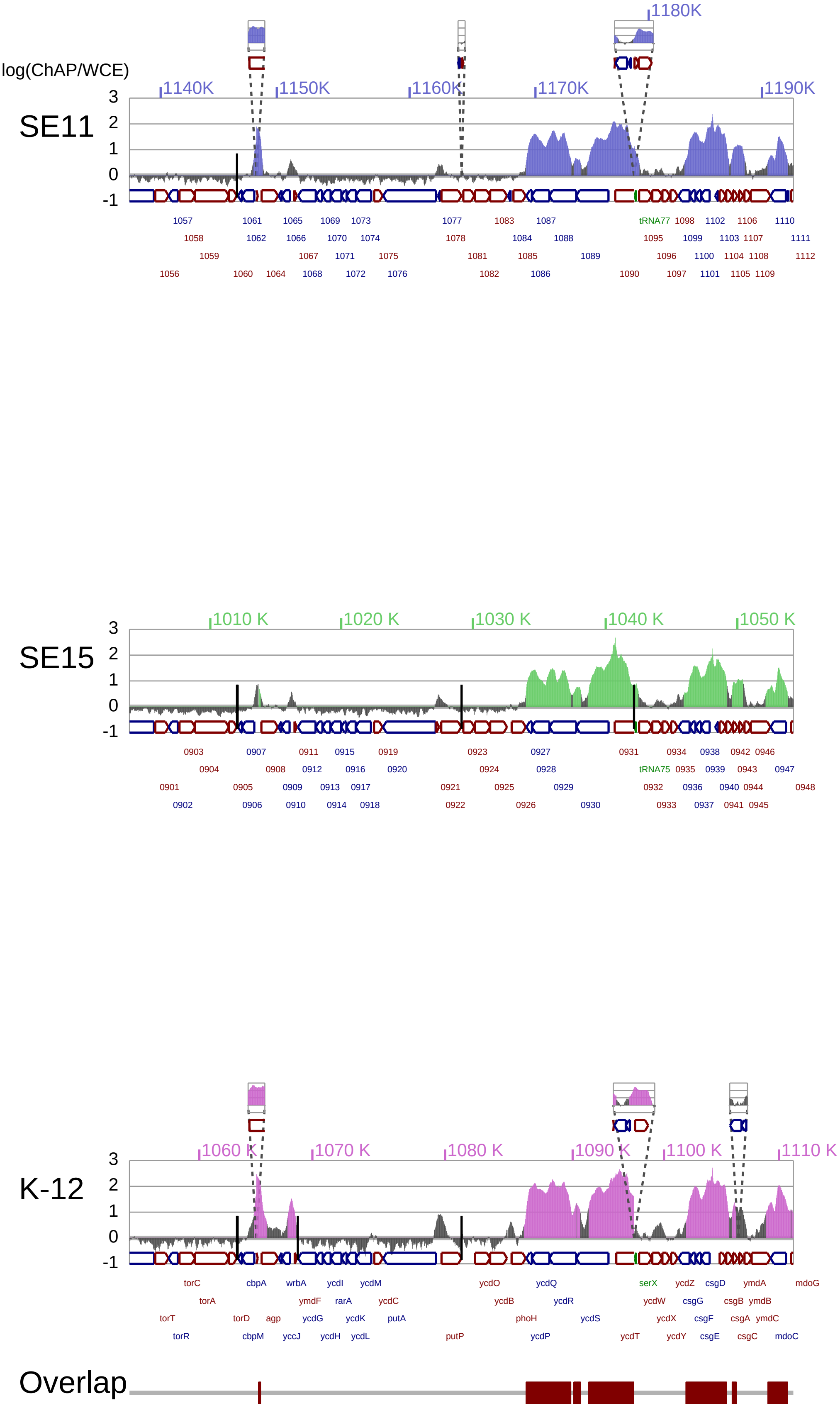

log(ChAP/WCE)
SE11
3
2
1
0
-1
1140K
1150K
1160K
1170K
1180K
1190K
1057 1061 1065 1069 1073 1077 1083 1087 tRNA77 1098 1102 1106 1110
1058 1062 1066 1070 1074 1078 1084 1088 1095 1099 1103 1107 1111
1059 1067 1071 1075 1081 1085 1089 1096 1100 1104 1108 1112
1056 1060 1064 1068 1072 1076 1082 1086 1090 1097 1101 1105 1109
SE15
1010 K
1020 K
1030 K
1040 K
1050 K
0903 0907 0911 0915 0919 0923 0927 0931 0934 0938 0942 0946
0904 0908 0912 0916 0920 0924 0928 tRNA75 0935 0939 0943 0947
0901 0905 0909 0913 0917 0921 0925 0929 0932 0936 0940 0944 0948
0902 0906 0910 0914 0918 0922 0926 0930 0933 0937 0941 0945
K-12
1060 K
1070 K
1080 K
1090 K
1100 K
1110 K
torC cbpA wrbA ycdI ycdM ycdO ycdQ serX ycdZ csgD ymdA mdoG
torA ymdF rarA ycdC ycdB ycdR ycdW csgG csgB ymdB
torT torD agp ycdG ycdK putA phoH ycdS ycdX csgF csgA ymdC
torR cbpM yccJ ycdH ycdL putP ycdP ycdT ycdY csgE csgC mdoC
Overlap

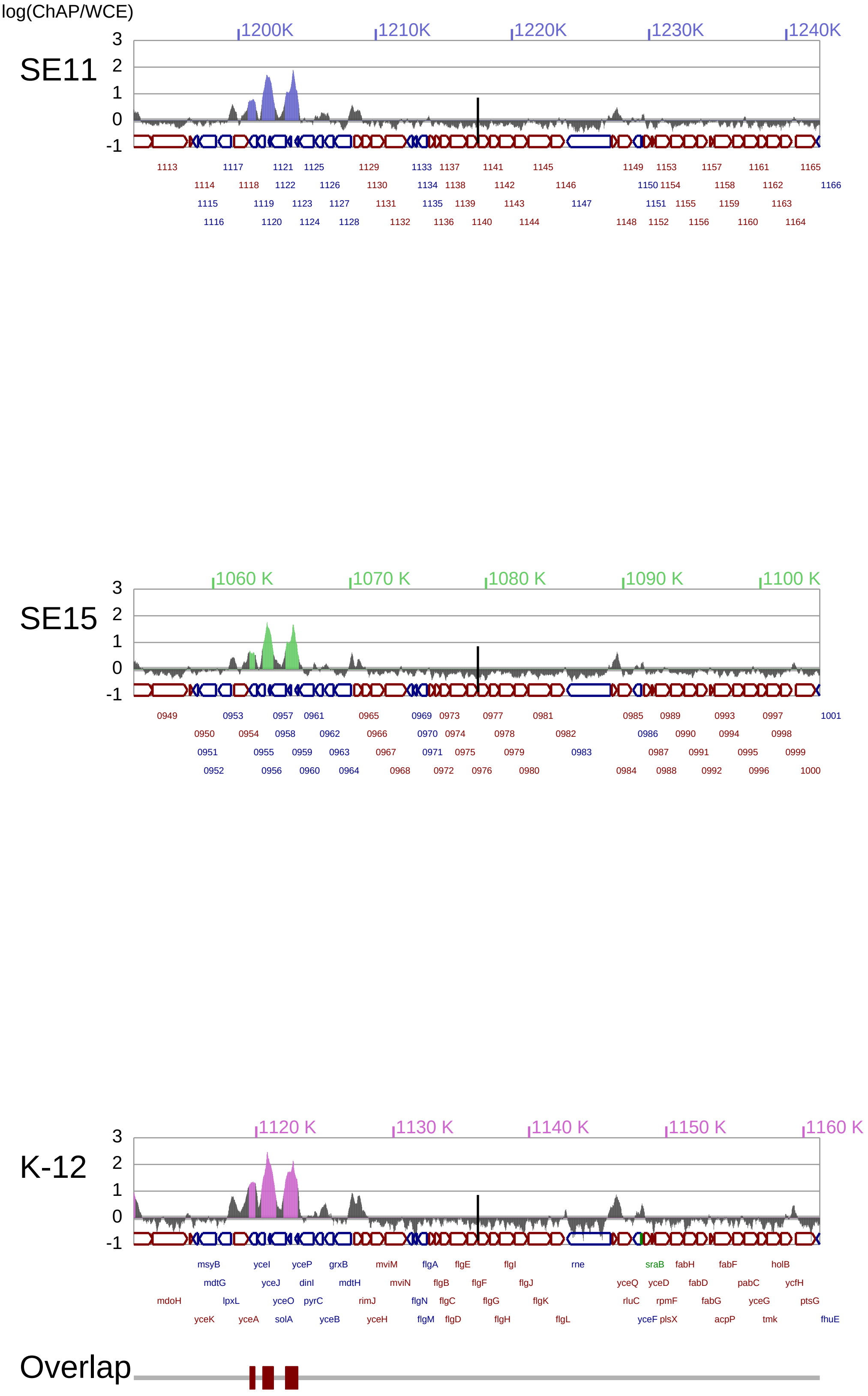

log(ChAP/WCE)
SE11
1200K
1210K
1220K
1230K
1240K
3
2
1
0
-1
1113 1117 1121 1125 1129 1133 1137 1141 1145 1149 1153 1157 1161 1165
1114 1118 1122 1126 1130 1134 1138 1142 1146 1150 1154 1158 1162 1166
1115 1119 1123 1127 1131 1135 1139 1143 1147 1151 1155 1159 1163
1116 1120 1124 1128 1132 1136 1140 1144 1148 1152 1156 1160 1164
SE15
1060 K
1070 K
1080 K
1090 K
1100 K
3
2
1
0
-1
0949 0953 0957 0961 0965 0969 0973 0977 0981 0985 0989 0993 0997 1001
0950 0954 0958 0962 0966 0970 0974 0978 0982 0986 0990 0994 0998
0951 0955 0959 0963 0967 0971 0975 0979 0983 0987 0991 0995 0999
0952 0956 0960 0964 0968 0972 0976 0980 0984 0988 0992 0996 1000
K-12
1120 K
1130 K
1140 K
1150 K
1160 K
3
2
1
0
-1
msyB yceI yceP grxB mviM flgA flgE flgI rne sraB fabH fabF holB
mdtG yceJ dinI mdtH mviN flgB flgF flgJ yceQ yceD fabD pabC ycfH
mdoH lpxL yceO pyrC rimJ flgN flgC flgG flgK rluC rpmF fabG yceG ptsG
yceK yceA solA yceB yceH flgM flgD flgH flgL yceF plsX acpP tmk fhuE
Overlap

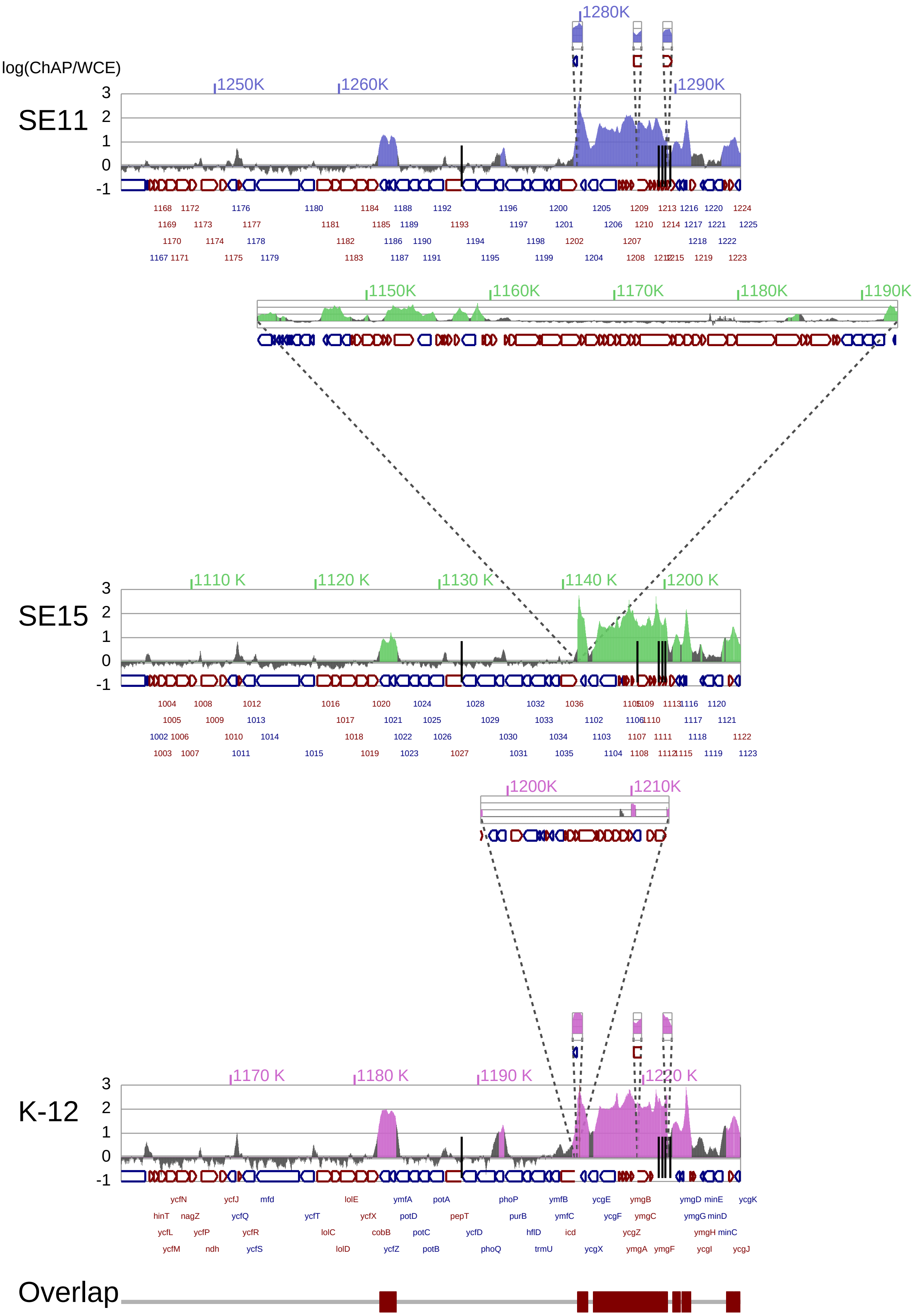

log(ChAP/WCE)
SE11
1250K
1260K
1280K
1290K
3
2
1
0
-1
1168 1172 1176 1180 1184 1188 1192 1196 1200 1205 1209 1213 1216 1220 1224
1169 1173 1177 1181 1185 1189 1193 1197 1201 1206 1210 1214 1217 1221 1225
1170 1174 1178 1182 1186 1190 1194 1198 1202 1207 1218 1222
1167 1171 1175 1179 1183 1187 1191 1195 1199 1204 1208 1212 1215 1219 1223
1150K
1160K
1170K
1180K
1190K
SE15
1110 K
1120 K
1130 K
1140 K
1200 K
3
2
1
0
-1
1004 1008 1012 1016 1020 1024 1028 1032 1036 1105 1109 1113 1116 1120
1005 1009 1013 1017 1021 1025 1029 1033 1102 1106 1110 1117 1121
1002 1006 1010 1014 1018 1022 1026 1030 1034 1103 1107 1111 1118 1122
1003 1007 1011 1015 1019 1023 1027 1031 1035 1104 1108 1112 1115 1119 1123
1200K
1210K
K-12
1170 K
1180 K
1190 K
1220 K
3
2
1
0
-1
ycfN ycfJ mfd lolE ymfA potA phoP ymfB ycgE ymgB ymgD minE ycgK
hinT nagZ ycfQ ycfT ycfX potD pepT purB ymfC ycgF ymgC ymgG minD
ycfL ycfP ycfR lolC cobB potC ycfD hflD icd ycgZ ymgH minC
ycfM ndh ycfS lolD ycfZ potB phoQ trmU ycgX ymgA ymgF ycgI ycgJ
Overlap

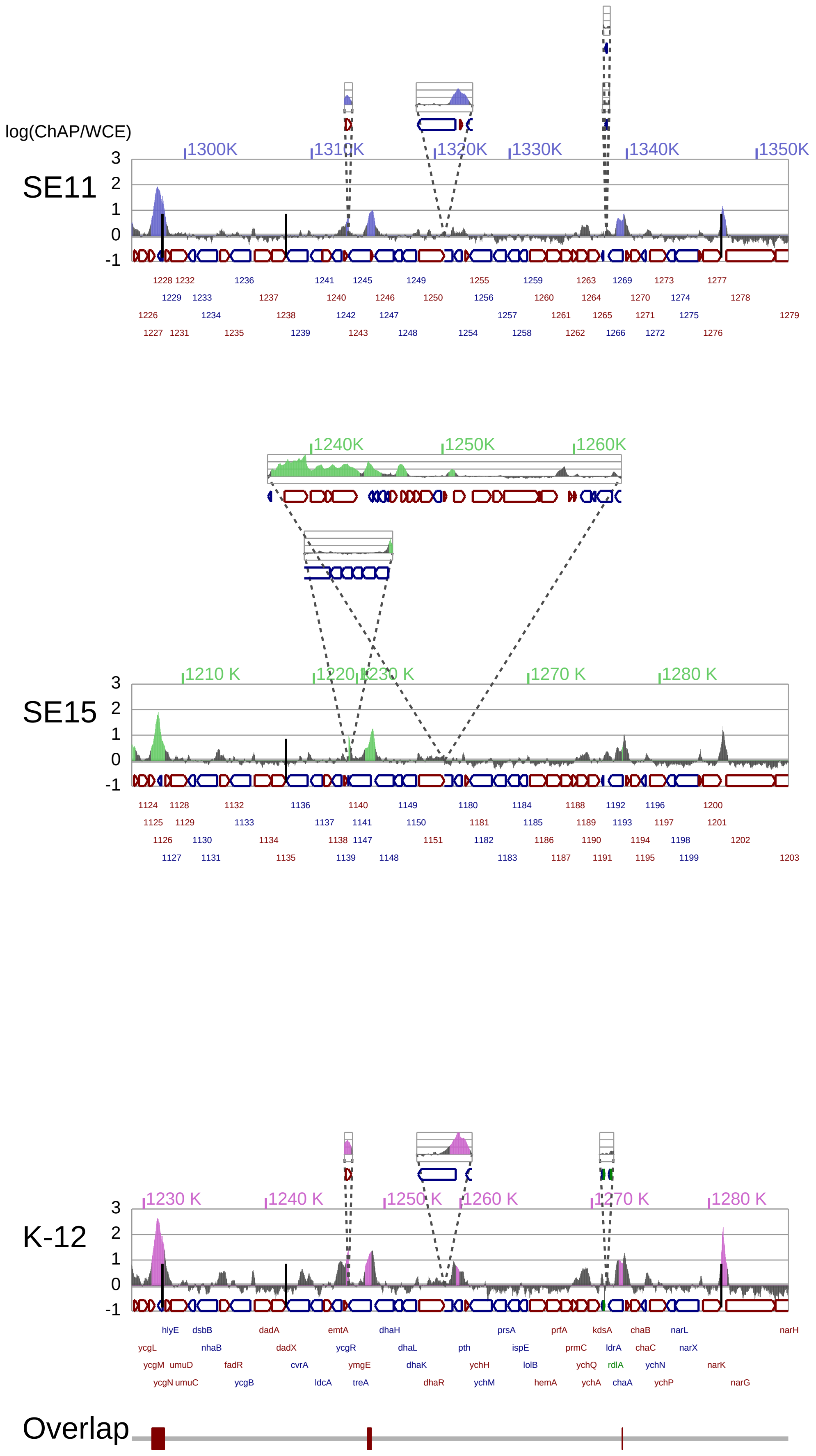

log(ChAP/WCE)
SE11
1300K
1310K
1320K
1330K
1340K
1350K
3
2
1
0
-1
1228 1232
1236
1241
1245
1249
1255
1259
1263
1269
1273
1277
1229
1233
1237
1240
1246
1250
1256
1260
1264
1270
1274
1278
1226
1234
1238
1242
1247
1257
1261
1265
1271
1275
1279
1227
1231
1235
1239
1243
1248
1254
1258
1262
1266
1272
1276
SE15
1240K
1250K
1260K
1210 K
1220 K
1230 K
1270 K
1280 K
1124
1128
1132
1136
1140
1149
1180
1184
1188
1192
1196
1200
1125
1129
1133
1137
1141
1150
1181
1185
1189
1193
1197
1201
1126
1130
1134
1138
1147
1151
1182
1186
1190
1194
1198
1202
1127
1131
1135
1139
1148
1183
1187
1191
1195
1199
1203
K-12
1230 K
1240 K
1250 K
1260 K
1270 K
1280 K
hlyE
dsbB
dadA
emtA
dhaH
prsA
prfA
kdsA
chaB
narL
narH
ycgL
nhaB
dadX
ycgR
dhaL
pth
ispE
prmC
ldrA
chaC
narX
ycgM
umuD
fadR
cvrA
ymgE
dhaK
ychH
lolB
ychQ
rdlA
ychN
narK
ycgN
umuC
ycgB
ldcA
treA
dhaR
ychM
hemA
ychA
chaA
ychP
narG
Overlap

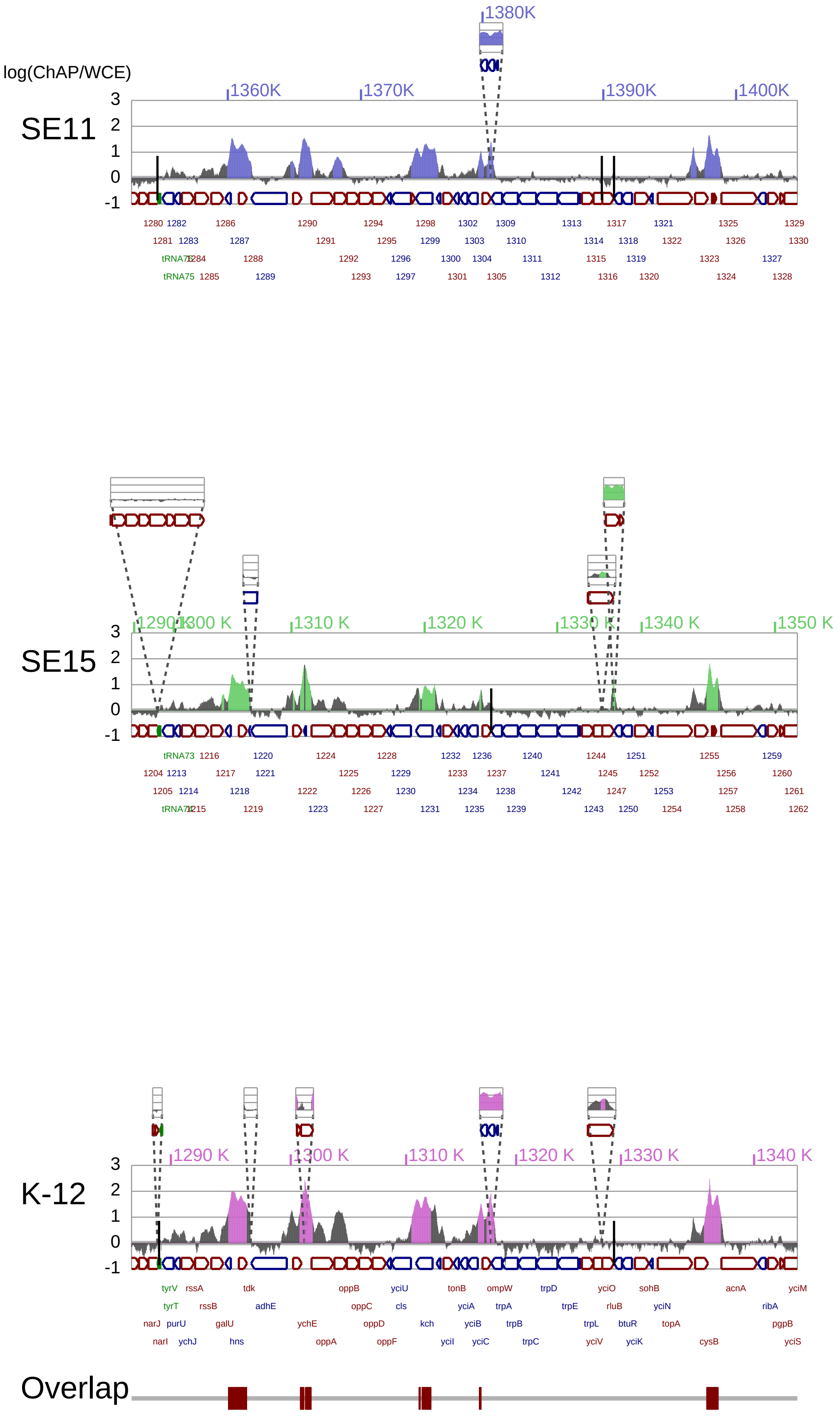
log(ChAP/WCE)
SE11
1360K
1370K
1380K
1390K
1400K
SE15
1290 K
1300 K
1310 K
1320 K
1330 K
1340 K
1350 K
K-12
1290 K
1300 K
1310 K
1320 K
1330 K
1340 K
Overlap

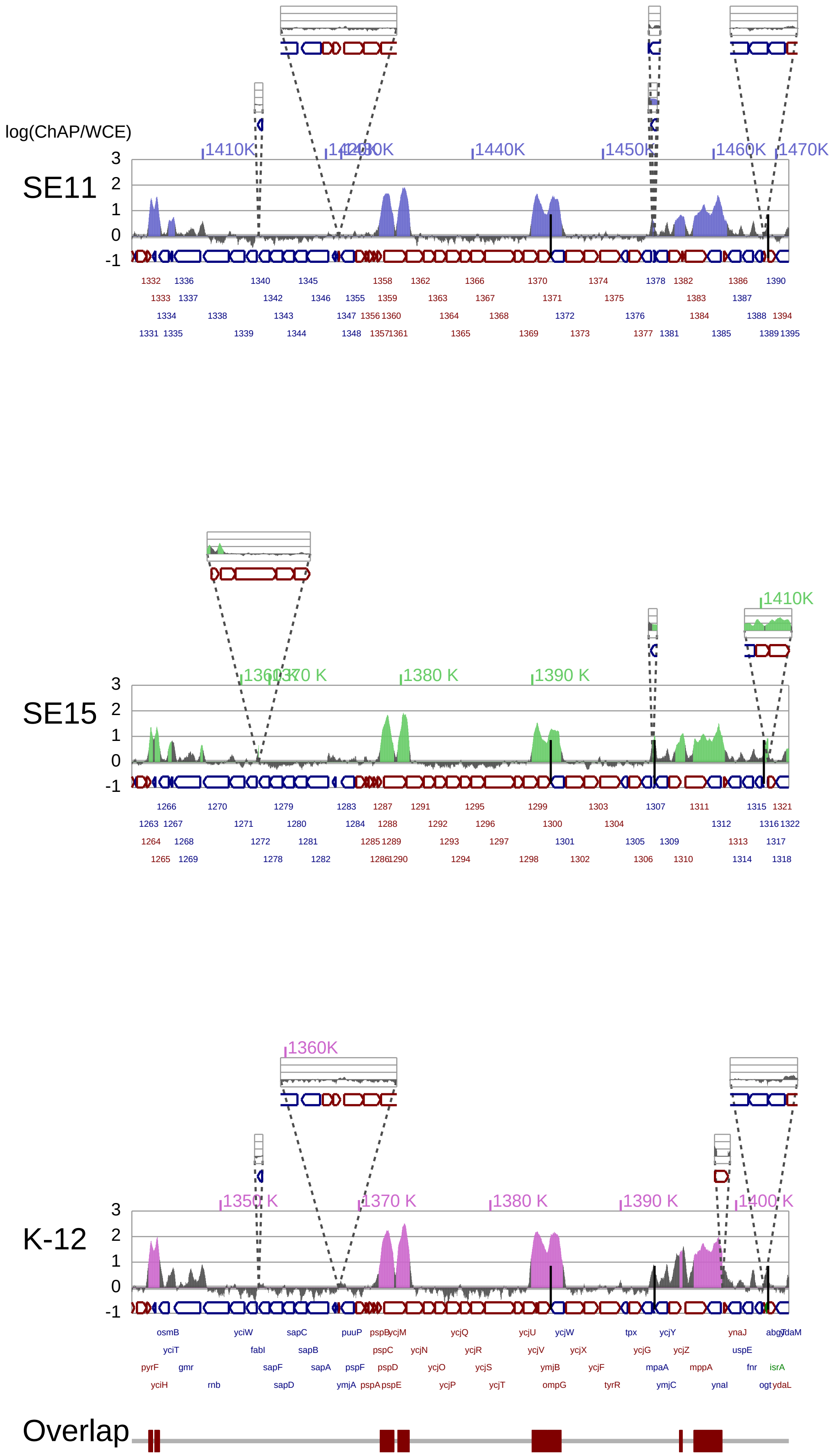

log(ChAP/WCE)
SE11
1410K
1440K
1450K
1460K
1470K
3
2
1
0
-1
1332 1336 1340 1345 1358 1362 1366 1370 1374 1378 1382 1386 1390
1333 1337 1342 1346 1355 1359 1363 1367 1371 1375 1383 1387
1334 1338 1343 1347 1356 1360 1364 1368 1372 1376 1384 1388 1394
1331 1335 1339 1344 1348 1357 1361 1365 1369 1373 1377 1381 1385 1389 1395
SE15
1410K
1380 K
1390 K
1266 1270 1279 1283 1287 1291 1295 1299 1303 1307 1311 1315 1321
1263 1267 1271 1280 1284 1288 1292 1296 1300 1304 1312 1316 1322
1264 1268 1272 1281 1285 1289 1293 1297 1301 1305 1309 1313 1317
1265 1269 1278 1282 1286 1290 1294 1298 1302 1306 1310 1314 1318
K-12
1360K
1370 K
1380 K
1390 K
osmB yciW sapC puuP pspB ycjM ycjQ ycjU ycjW tpx ycjY ynaJ
yciT fabI sapB pspC ycjN ycjR ycjV ycjX ycjG ycjZ uspE
pyrF gmr sapF sapA pspF pspD ycjO ycjS ymjB ycjF mpaA mppA fnr isrA
yciH rnb sapD ymjA pspA pspE ycjP ycjT ompG tyrR ymjC ynaI ogt ydaL
Overlap

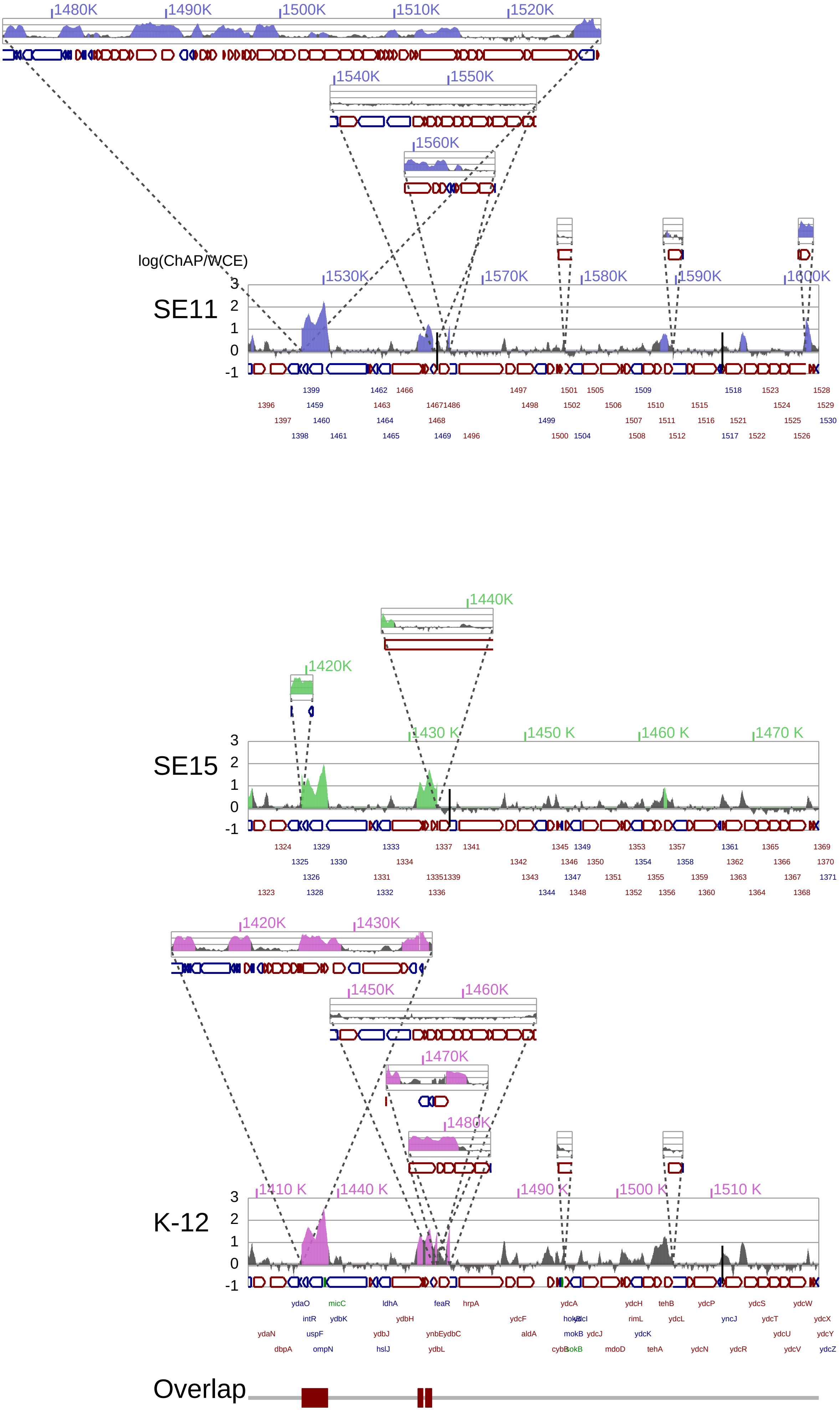

log(ChAP/WCE)
SE11
1480K
1490K
1500K
1510K
1520K
1540K
1550K
1560K
1530K
1570K
1580K
1590K
1600K
3
2
1
0
-1
1399 1462 1466 1497 1501 1505 1509 1518 1523 1528
1396 1459 1463 14671486 1498 1502 1506 1510 1515 1524 1529
1397 1460 1464 1468 1499 1507 1511 1516 1521 1525 1530
1398 1461 1465 1469 1496 1500 1504 1508 1512 1517 1522 1526
SE15
1440K
1420K
1430 K
1450 K
1460 K
1470 K
1324 1329 1333 1337 1341 1345 1349 1353 1357 1361 1365 1369
1325 1330 1334 1342 1346 1350 1354 1358 1362 1366 1370
1326 1331 13351339 1343 1347 1351 1355 1359 1363 1367 1371
1323 1328 1332 1336 1344 1348 1352 1356 1360 1364 1368
K-12
1420K
1430K
1450K
1460K
1470K
1480K
1410 K
1440 K
1490 K
1500 K
1510 K
ydaO micC ldhA feaR hrpA ydcA ydcH tehB ydcP ydcS ydcW
intR ydbK ydbH ydcF hokB ydcI rimL ydcL yncJ ydcT ydcX
ydaN uspF ydbJ ynbEydbC aldA mokB ydcJ ydcK ydcU ydcY
dbpA ompN hslJ ydbL cybB sokB mdoD tehA ydcN ydcR ydcV ydcZ
Overlap

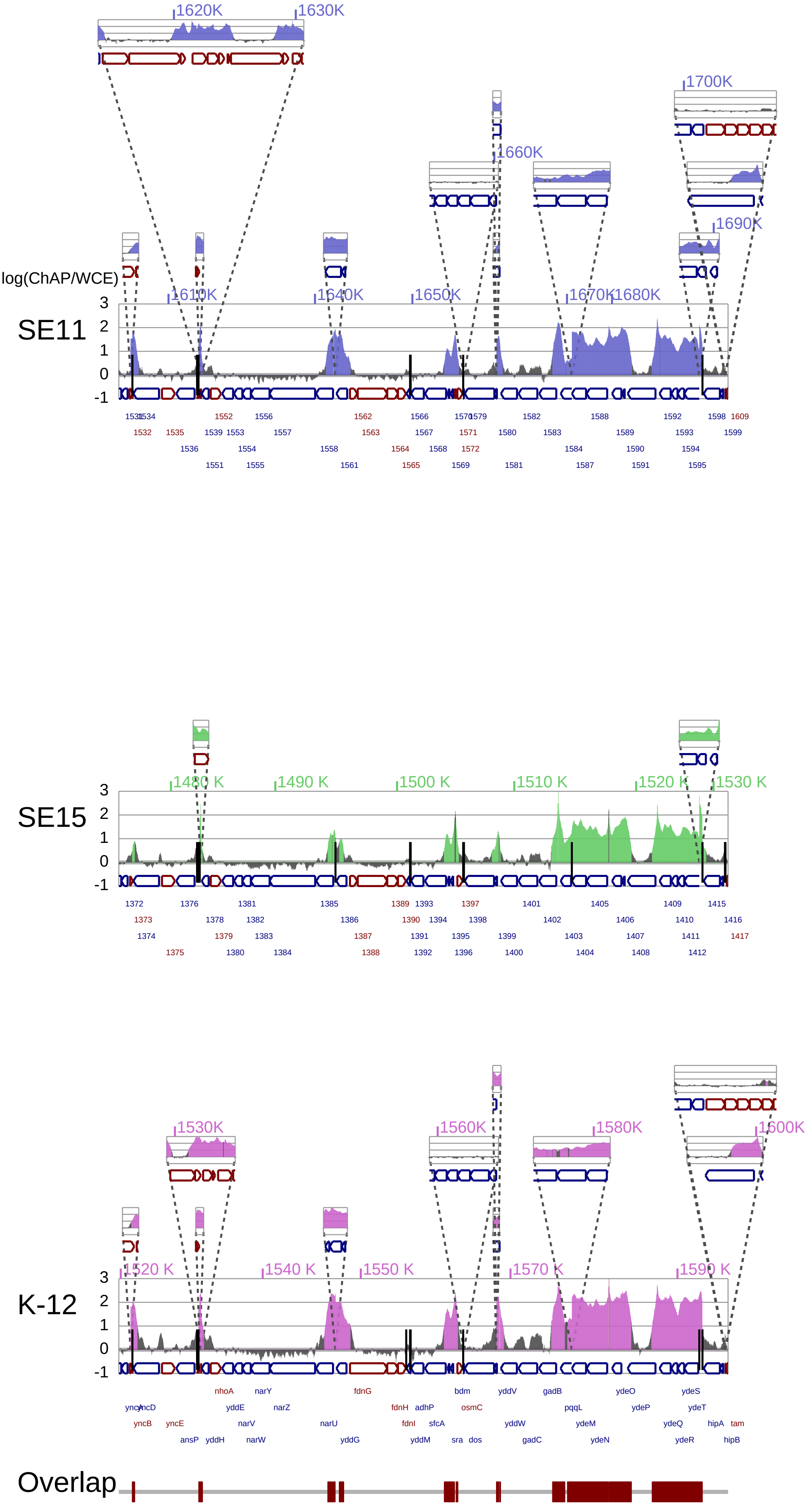

1620K
1630K
1700K
1660K
1690K
log(ChAP/WCE)
1610K
1640K
1650K
1670K
1680K
SE11
3
2
1
0
-1
1531 1534 1552 1556 1562 1566 1570 1579 1582 1588 1592 1598 1609
1532 1535 1539 1553 1557 1563 1567 1571 1580 1583 1589 1593 1599
1536 1554 1558 1564 1568 1572 1584 1590 1594
1551 1555 1561 1565 1569 1581 1587 1591 1595
SE15
1480 K
1490 K
1500 K
1510 K
1520 K
1530 K
1372 1376 1381 1385 1389 1393 1397 1401 1405 1409 1415
1373 1378 1382 1386 1390 1394 1398 1402 1406 1410 1416
1374 1379 1383 1387 1391 1395 1399 1403 1407 1411 1417
1375 1380 1384 1388 1392 1396 1400 1404 1408 1412
K-12
1530K
1560K
1580K
1600K
1520 K
1540 K
1550 K
1570 K
1590 K
nhoA narY fdnG bdm yddV gadB ydeO ydeS
yncD yddE narZ fdnH adhP osmC pqqL ydeP ydeT
yncB yncE narV narU fdnI sfcA yddW ydeM ydeQ hipA tam
ansP yddH narW yddG yddM sra dos gadC ydeN ydeR hipB
Overlap

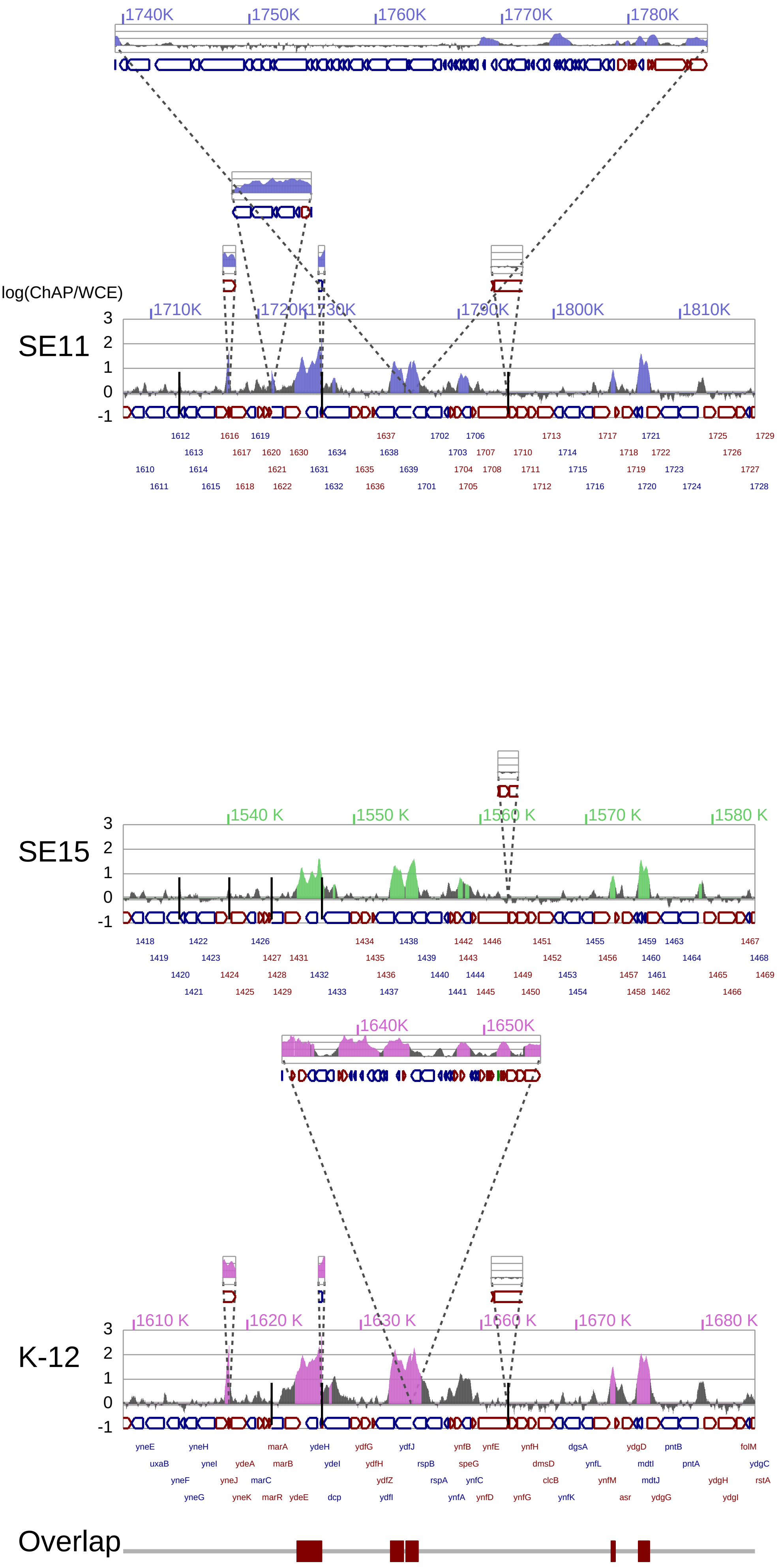

1740K
1750K
1760K
1770K
1780K
log(ChAP/WCE)
SE11
3
2
1
0
-1
1710K
1720K
1730K
1790K
1800K
1810K
1612 1616 1619 1637 1702 1706 1713 1717 1721 1725 1729
1613 1617 1620 1630 1634 1638 1703 1707 1710 1714 1718 1722 1726
1610 1614 1621 1631 1635 1639 1704 1708 1711 1715 1719 1723 1727
1611 1615 1618 1622 1632 1636 1701 1705 1712 1716 1720 1724 1728
SE15
1540 K
1550 K
1560 K
1570 K
1580 K
1418 1422 1426 1434 1438 1442 1446 1451 1455 1459 1463 1467
1419 1423 1427 1431 1435 1439 1443 1452 1456 1460 1464 1468
1420 1424 1428 1432 1436 1440 1444 1449 1453 1457 1461 1465 1469
1421 1425 1429 1433 1437 1441 1445 1450 1454 1458 1462 1466
1640K
1650K
K-12
1610 K
1620 K
1630 K
1660 K
1670 K
1680 K
yneE yneH marA ydeH ydfG ydfJ ynfB ynfE ynfH dgsA ydgD pntB folM
uxaB yneI ydeA marB ydeI ydfH rspB speG dmsD ynfL mdtI pntA ydgC
yneF yneJ marC ydfZ rspA ynfC clcB ynfM mdtJ ydgH rstA
yneG yneK marR ydeE dcp ydfI ynfA ynfD ynfG ynfK asr ydgG ydgI
Overlap

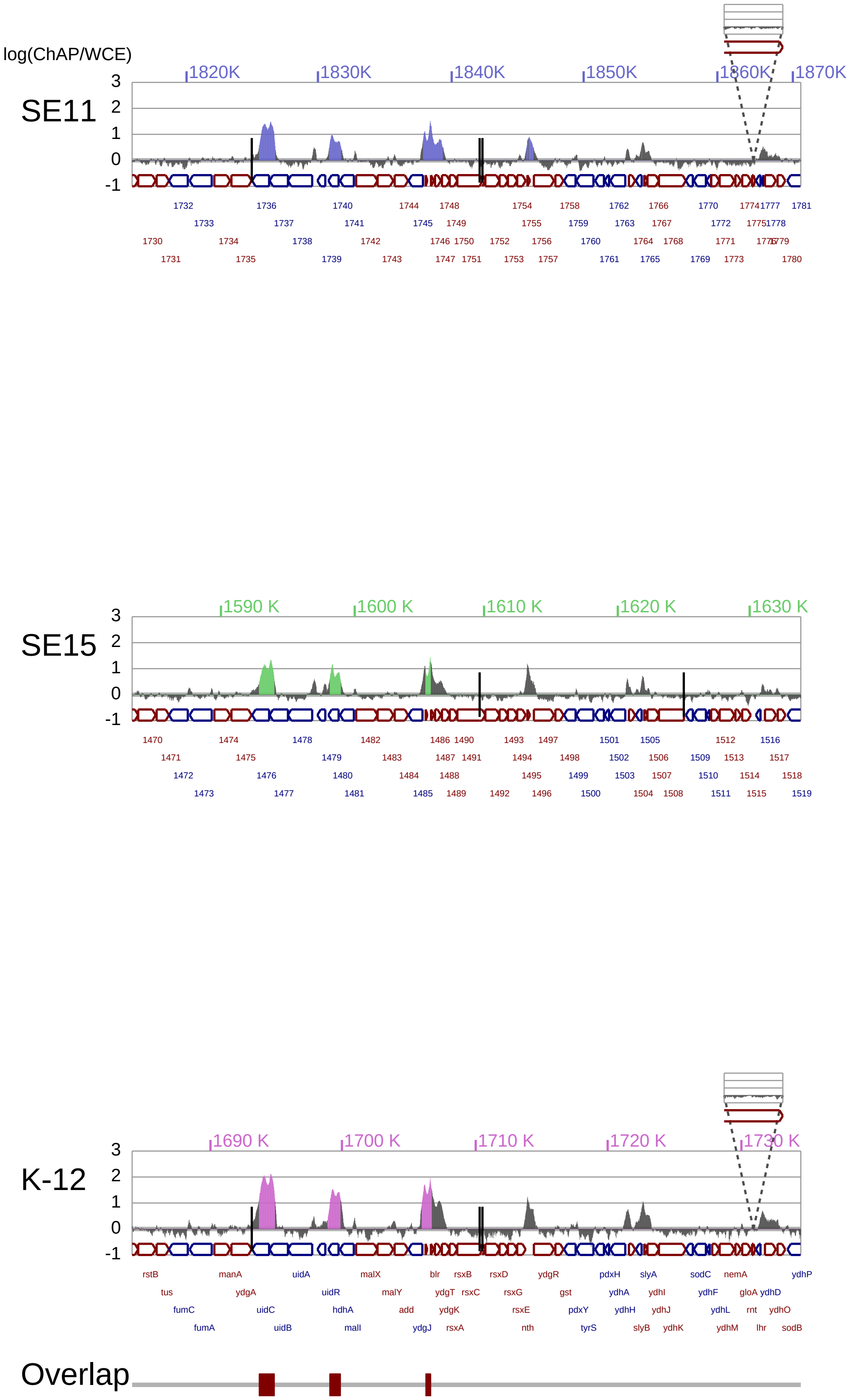

log(ChAP/WCE)
SE11
3
2
1
0
-1
1820K
1830K
1840K
1850K
1860K
1870K
1732 1736 1740 1744 1748 1754 1758 1762 1766 1770 1774 1777 1781
1733 1737 1741 1745 1749 1755 1759 1763 1767 1772 1775 1778
1730 1734 1738 1742 1746 1750 1752 1756 1760 1764 1768 1771 1776 1779
1731 1735 1739 1743 1747 1751 1753 1757 1761 1765 1769 1773 1780
SE15
3
2
1
0
-1
1590 K
1600 K
1610 K
1620 K
1630 K
1470 1474 1478 1482 1486 1490 1493 1497 1501 1505 1512 1516
1471 1475 1479 1483 1487 1491 1494 1498 1502 1506 1509 1513 1517
1472 1476 1480 1484 1488 1495 1499 1503 1507 1510 1514 1518
1473 1477 1481 1485 1489 1492 1496 1500 1504 1508 1511 1515 1519
K-12
3
2
1
0
-1
1690 K
1700 K
1710 K
1720 K
1730 K
rstB manA uidA malX blr rsxB rsxD ydgR pdxH slyA sodC nemA ydhP
tus ydgA uidR malY ydgT rsxC rsxG gst ydhA ydhI ydhF gloA ydhD
fumC uidC hdhA add ydgK rsxE pdxY ydhH ydhJ ydhL rnt ydhO
fumA uidB malI ydgJ rsxA nth tyrS slyB ydhK ydhM lhr sodB
Overlap

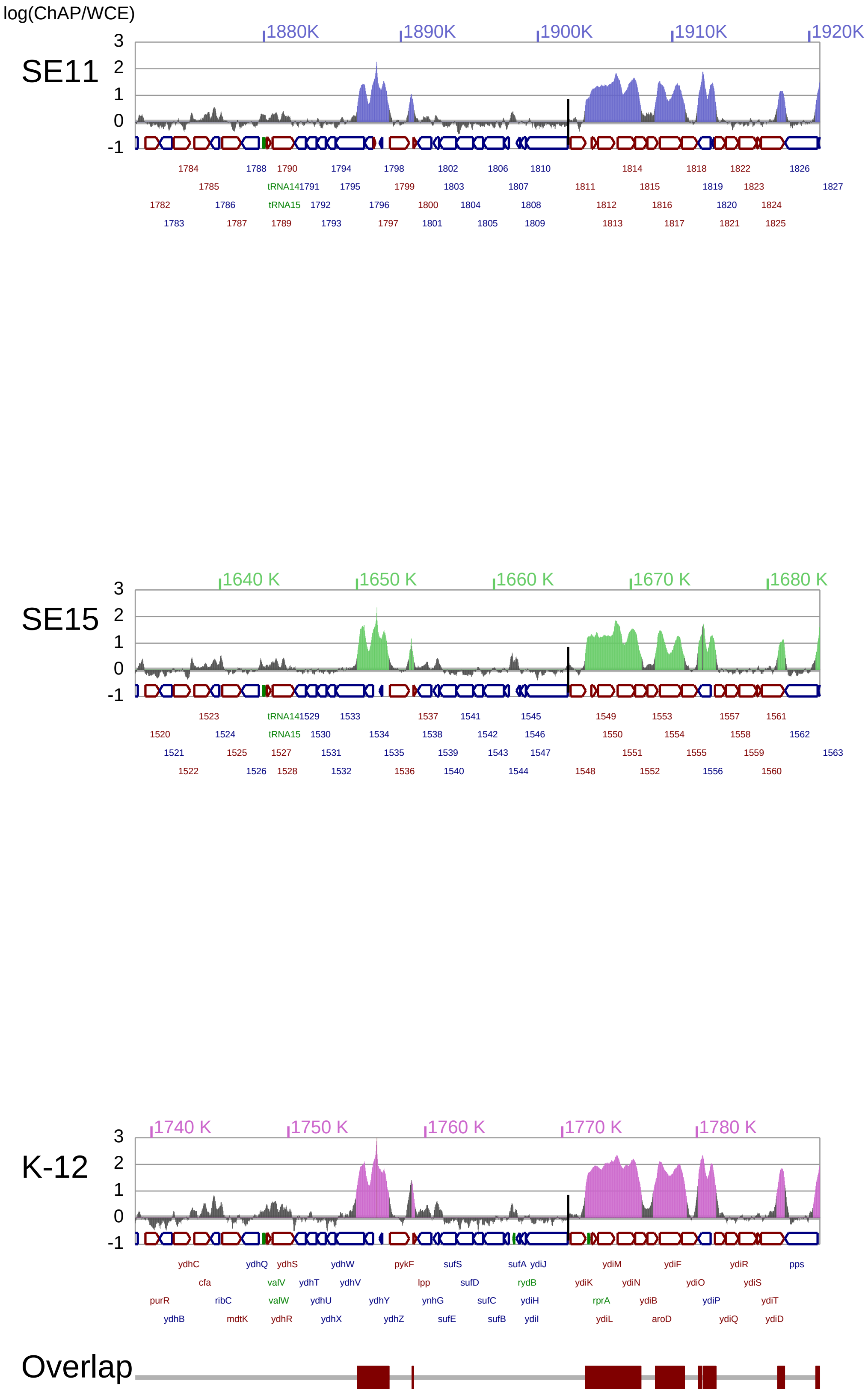
log(ChAP/WCE)
SE11
1880K
1890K
1900K
1910K
1920K
3
2
1
0
-1
1784 1788 1790 1794 1798 1802 1806 1810 1814 1818 1822 1826
1785 tRNA14 1791 1795 1799 1803 1807 1811 1815 1819 1823 1827
1782 1786 tRNA15 1792 1796 1800 1804 1808 1812 1816 1820 1824
1783 1787 1789 1793 1797 1801 1805 1809 1813 1817 1821 1825
SE15
1640 K
1650 K
1660 K
1670 K
1680 K
3
2
1
0
-1
1523 tRNA14 1529 1533 1537 1541 1545 1549 1553 1557 1561
1520 1524 tRNA15 1530 1534 1538 1542 1546 1550 1554 1558 1562
1521 1525 1527 1531 1535 1539 1543 1547 1551 1555 1559 1563
1522 1526 1528 1532 1536 1540 1544 1548 1552 1556 1560
K-12
1740 K
1750 K
1760 K
1770 K
1780 K
3
2
1
0
-1
ydhC ydhQ ydhS ydhW pykF sufS sufA ydiJ ydiM ydiF ydiR pps
cfa valV ydhT ydhV lpp sufD rydB ydiK ydiN ydiO ydiS
purR ribC valW ydhU ydhY ynhG sufC ydiH rprA ydiB ydiP ydiT
ydhB mdtK ydhR ydhX ydhZ sufE sufB ydiI ydiL aroD ydiQ ydiD
Overlap

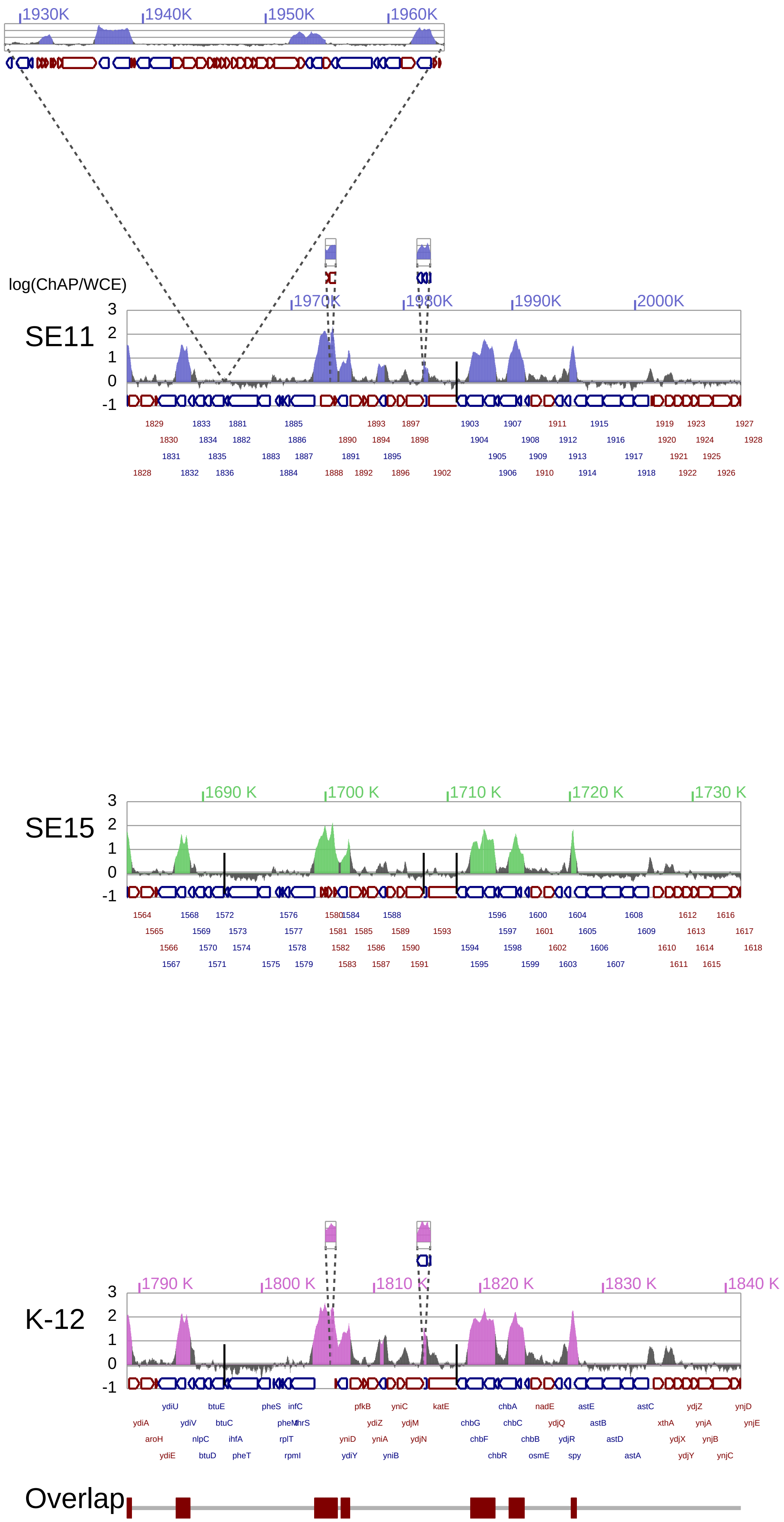
1930K
1940K
1950K
1960K
log(ChAP/WCE)
SE11
1970K
1980K
1990K
2000K
3
2
1
0
-1
1829 1833 1881 1885 1893 1897 1903 1907 1911 1915 1919 1923 1927
1830 1834 1882 1886 1890 1894 1898 1904 1908 1912 1916 1920 1924 1928
1831 1835 1883 1887 1891 1895 1905 1909 1913 1917 1921 1925
1828 1832 1836 1884 1888 1892 1896 1902 1906 1910 1914 1918 1922 1926
SE15
1690 K
1700 K
1710 K
1720 K
1730 K
1564 1568 1572 1576 1580 1584 1588 1596 1600 1604 1608 1612 1616
1565 1569 1573 1577 1581 1585 1589 1593 1597 1601 1605 1609 1613 1617
1566 1570 1574 1578 1582 1586 1590 1594 1598 1602 1606 1610 1614 1618
1567 1571 1575 1579 1583 1587 1591 1595 1599 1603 1607 1611 1615
K-12
1790 K
1800 K
1810 K
1820 K
1830 K
1840 K
ydiU btuE pheS infC pfkB yniC katE chbA nadE astE astC ydjZ ynjD
ydiA ydiV btuC pheM thrS ydiZ ydjM chbG chbC ydjQ astB xthA ynjA ynjE
aroH nlpC ihfA rplT yniD yniA ydjN chbF chbB ydjR astD ydjX ynjB
ydiE btuD pheT rpmI ydiY yniB chbR osmE spy astA ydjY ynjC
Overlap

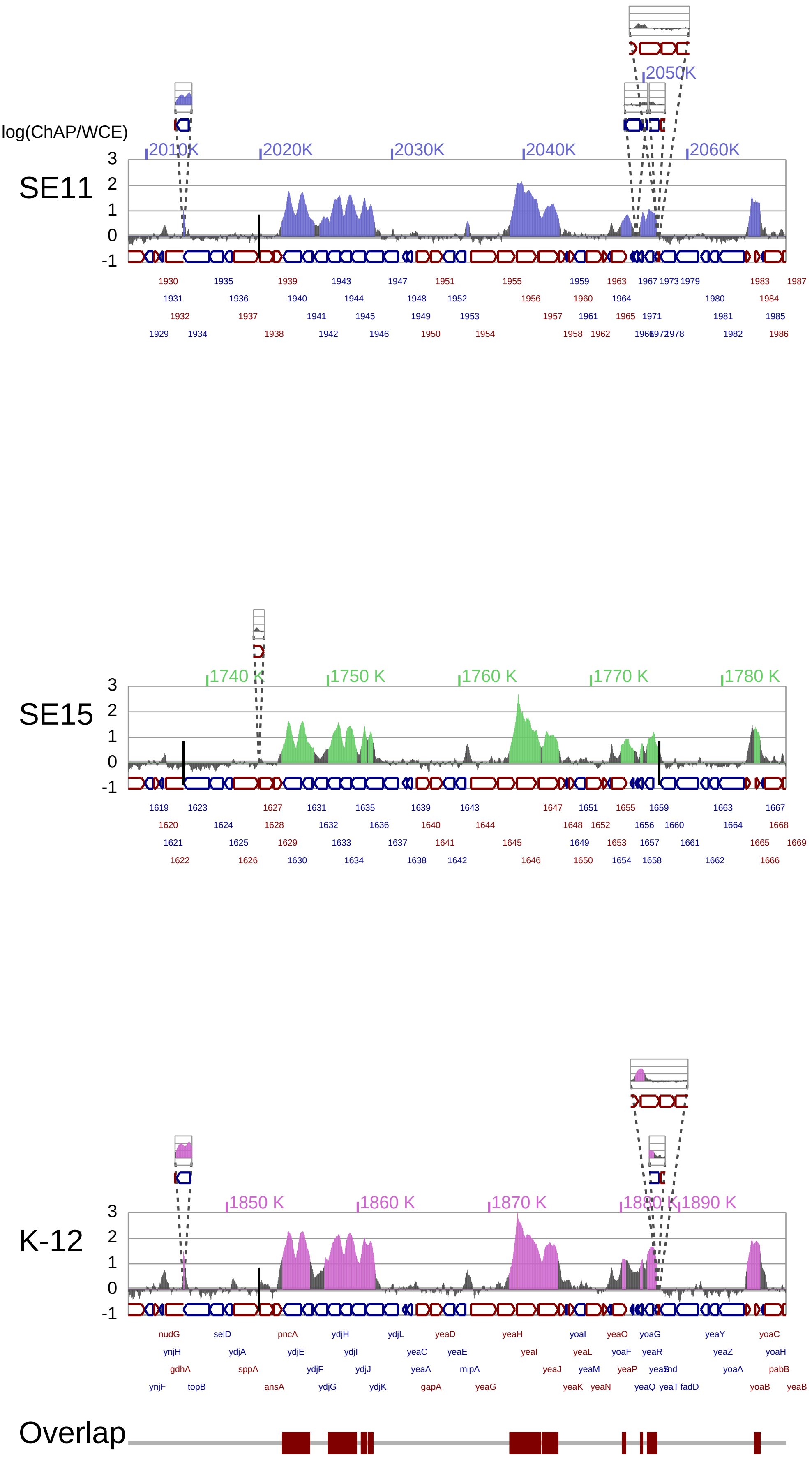
log(ChAP/WCE)
SE11
2010K
2020K
2030K
2040K
2050K
2060K
SE15
1740 K
1750 K
1760 K
1770 K
1780 K
K-12
1850 K
1860 K
1870 K
1880 K
1890 K
nudG ynjH gdhA ynjF selD topB ydjA sppA ansA pncA ydjE ydjF ydjG ydjH ydjI ydjJ ydjK ydjL yeaC yeaA gapA yeaD yeaE mipA yeaG yeaH yeaI yeaJ yeaK yoaI yeaL yeaM yeaN yoaO yoaF yeaP yeaQ yoaG yeaR yeaS yeaT fadD yeaY yeaZ yoaA yoaB yoaC yoaH pabB yeaB
Overlap

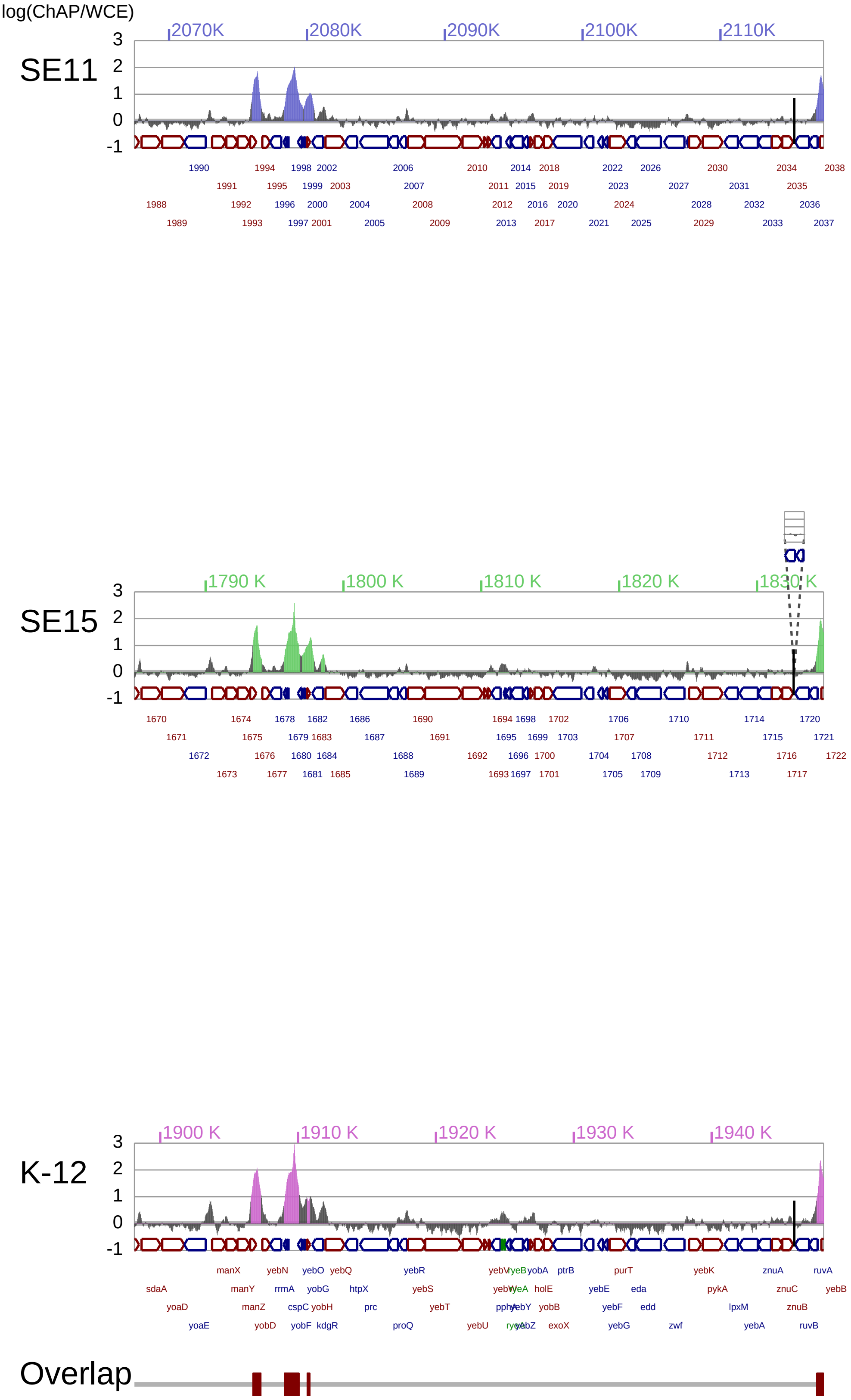
log(ChAP/WCE)
SE11
2070K
2080K
2090K
2100K
2110K
3
2
1
0
-1
1990 1994 1998 2002 2006 2010 2014 2018 2022 2026 2030 2034 2038
1991 1995 1999 2003 2007 2011 2015 2019 2023 2027 2031 2035
1988 1992 1996 2000 2004 2008 2012 2016 2020 2024 2028 2032 2036
1989 1993 1997 2001 2005 2009 2013 2017 2021 2025 2029 2033 2037
SE15
1790 K
1800 K
1810 K
1820 K
1830 K
1670 1674 1678 1682 1686 1690 1694 1698 1702 1706 1710 1714 1720
1671 1675 1679 1683 1687 1691 1695 1699 1703 1707 1711 1715 1721
1672 1676 1680 1684 1688 1692 1696 1700 1704 1708 1712 1716 1722
1673 1677 1681 1685 1689 1693 1697 1701 1705 1709 1713 1717
K-12
1900 K
1910 K
1920 K
1930 K
1940 K
manX yebN yebO yebQ yebR yebV ryeB yobA ptrB purT yebK znuA ruvA
sdaA manY rrmA yobG htpX yebS yebW ryeA holE yebE eda pykA znuC yebB
yoaD manZ cspC yobH prc yebT pphA yebY yobB yebF edd lpxM znuB
yoaE yobD yobF kdgR proQ yebU ryeA yebZ exoX yebG zwf yebA ruvB
Overlap

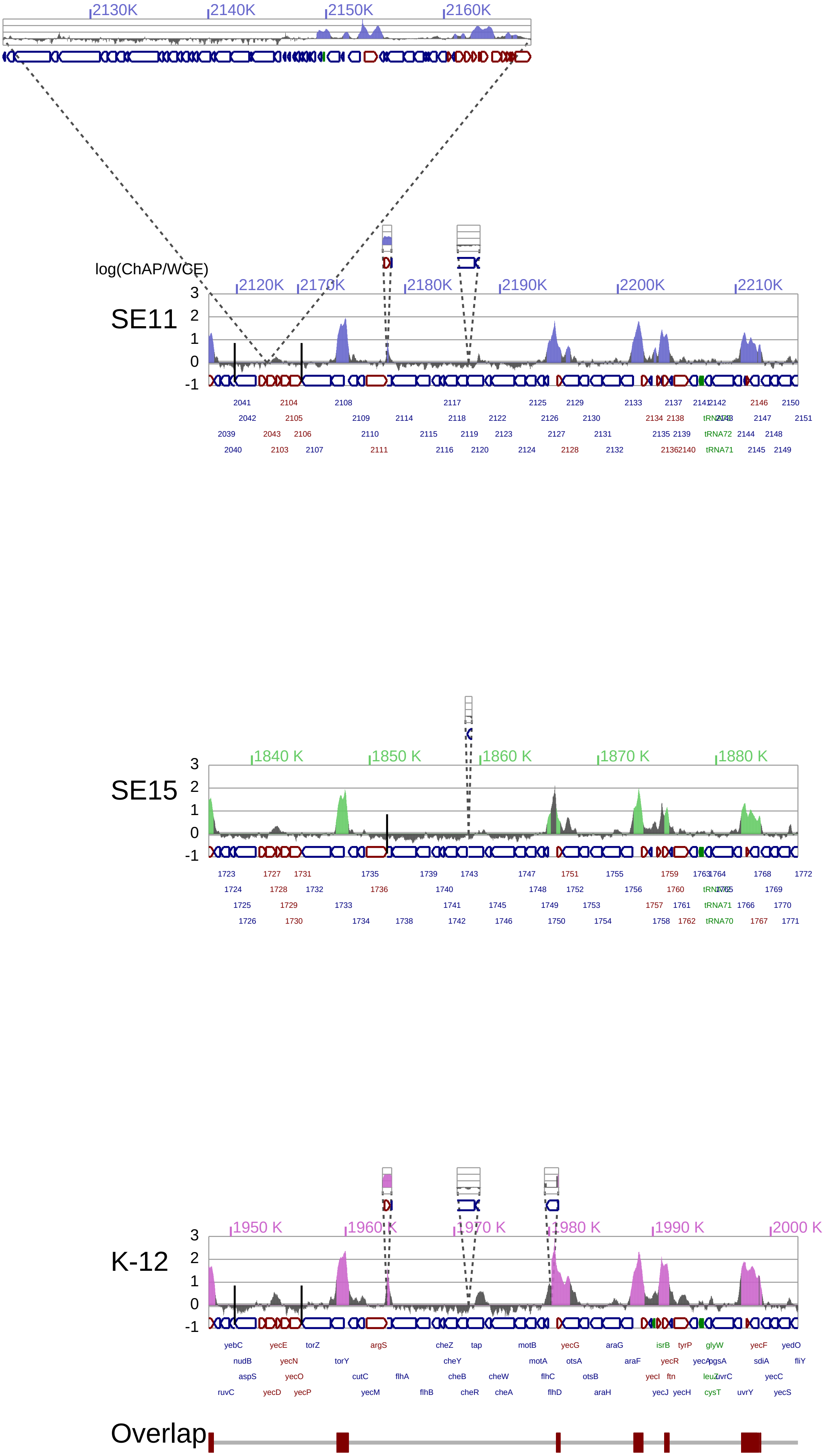

2130K
2140K
2150K
2160K
log(ChAP/WCE)
SE11
2120K
2170K
2180K
2190K
2200K
2210K
SE15
1840 K
1850 K
1860 K
1870 K
1880 K
K-12
1950 K
1960 K
1970 K
1980 K
1990 K
2000 K
3
2
1
0
-1
yebC
yecE
torZ
argS
cheZ
tap
motB
yecG
araG
isrB
tyrP
glyW
yecF
yedO
nudB
yecN
torY
cheY
motA
otsA
araF
yecR
sdiA
fliY
aspS
yecO
cutC
flhA
cheB
cheW
flhC
otsB
yecI
ftn
yecC
ruvC
yecD
yecP
yecM
flhB
cheR
cheA
flhD
araH
yecJ
yecH
cysT
uvrY
yecS
Overlap

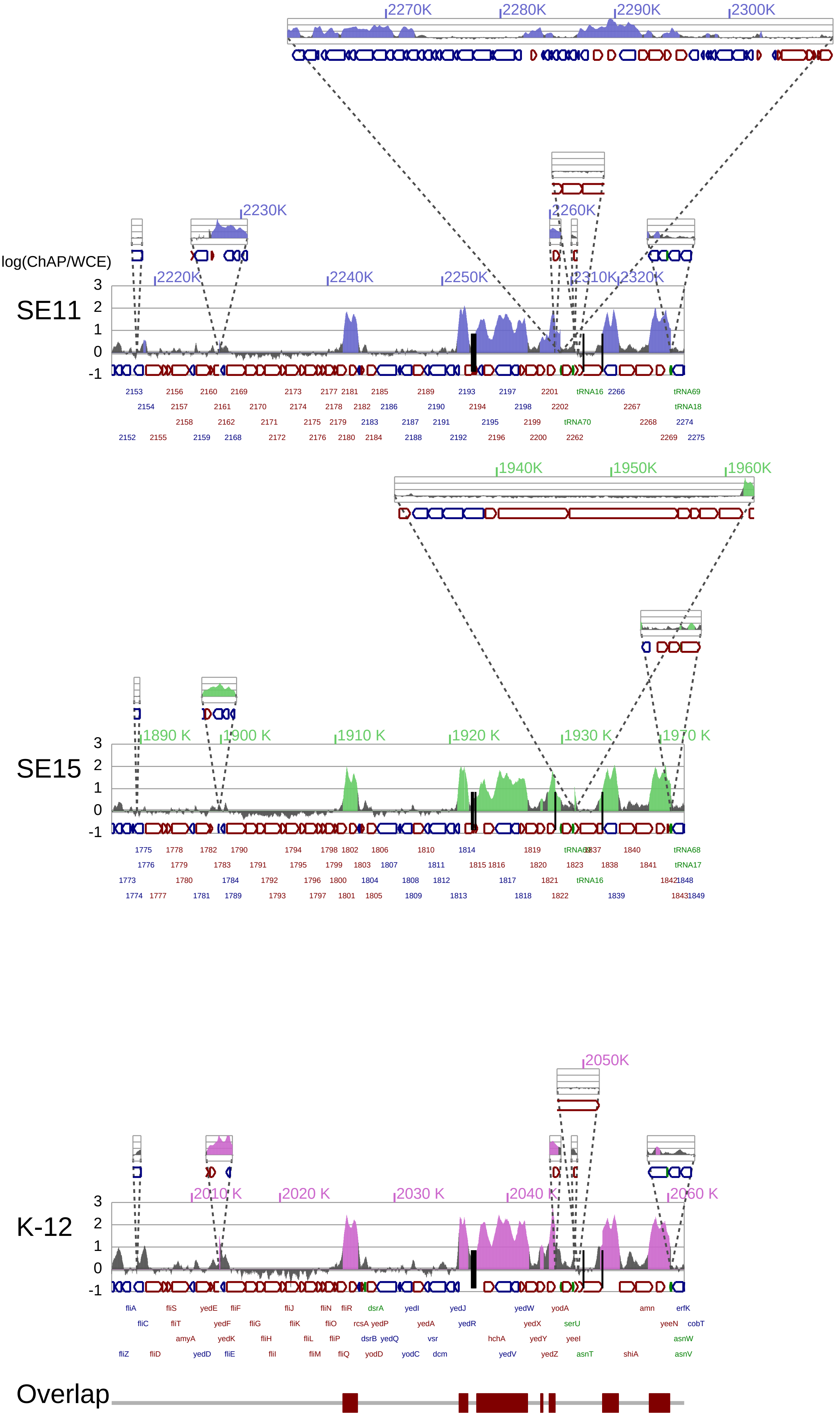

log(ChAP/WCE)
SE11
SE15
K-12
Overlap
2220K
2230K
2240K
2250K
2260K
2270K
2280K
2290K
2300K
2310K
2320K
1890 K
1900 K
1910 K
1920 K
1930 K
1940K
1950K
1960K
1970 K
2010 K
2020 K
2030 K
2040 K
2050K
2060 K
3
2
1
0
-1
2153 2156 2160 2169 2173 2177 2181 2185 2189 2193 2197 2201 tRNA16 2266 tRNA69
2154 2157 2161 2170 2174 2178 2182 2186 2190 2194 2198 2202 2267 tRNA18
2158 2162 2171 2175 2179 2183 2187 2191 2195 2199 tRNA70 2268 2274
2152 2155 2159 2168 2172 2176 2180 2184 2188 2192 2196 2200 2262 2269 2275
1775 1778 1782 1790 1794 1798 1802 1806 1810 1814 1819 tRNA69 1837 1840 tRNA68
1776 1779 1783 1791 1795 1799 1803 1807 1811 1815 1816 1820 1823 1838 1841 tRNA17
1773 1780 1784 1792 1796 1800 1804 1808 1812 1817 1821 tRNA16 1842 1848
1774 1777 1781 1789 1793 1797 1801 1805 1809 1813 1818 1822 1839 1843 1849
fliA fliS yedE fliF fliJ fliN fliR dsrA yedI yedJ yedW yodA amn erfK
fliC fliT yedF fliG fliK fliO rcsA yedP yedA yedR yedX serU yeeN cobT
amyA yedK fliH fliL fliP dsrB yedQ vsr hchA yedY yeeI asnW
fliZ fliD yedD fliE fliI fliM fliQ yodD yodC dcm yedV yedZ asnT shiA asnV

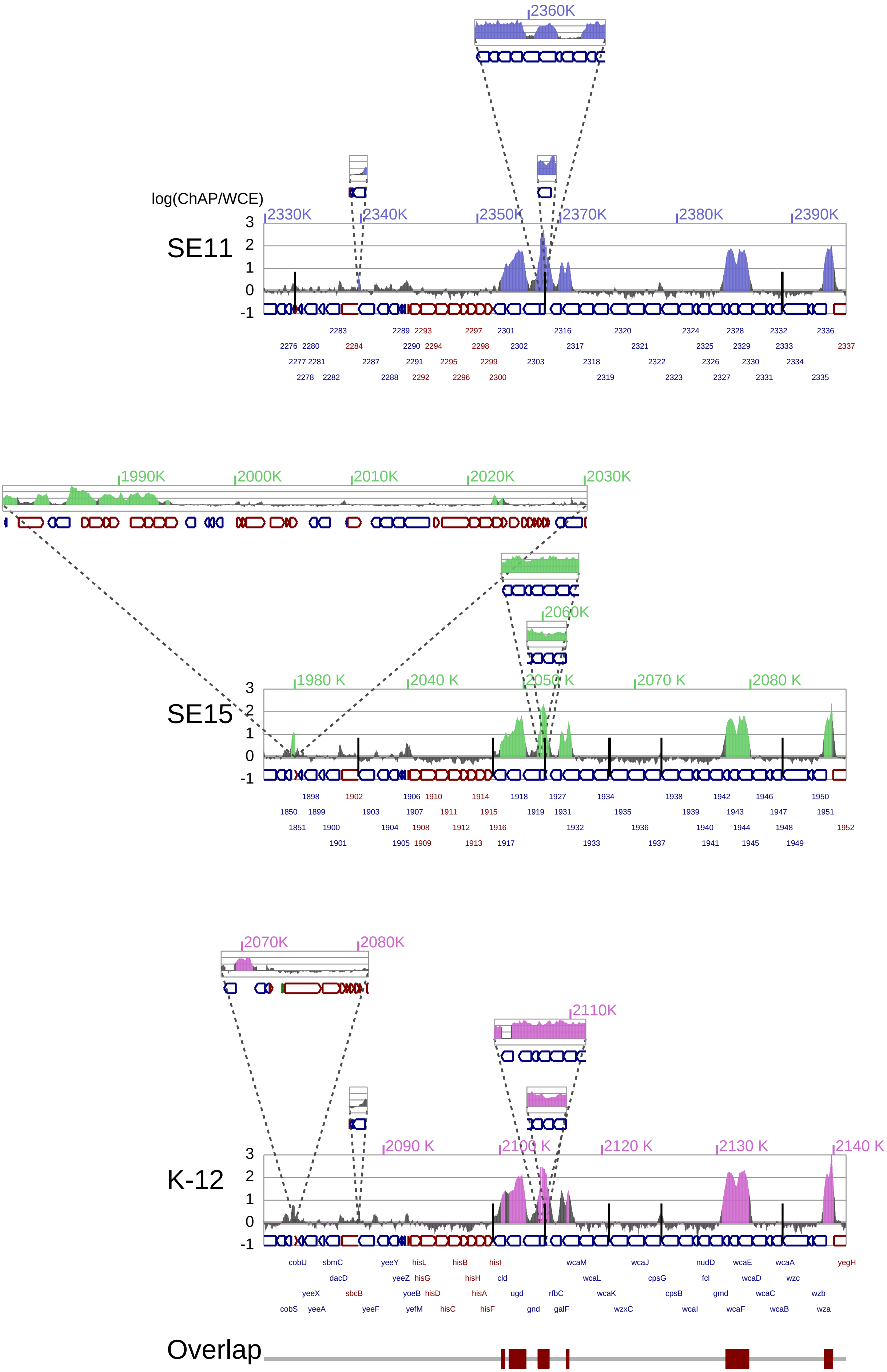

log(ChAP/WCE)
SE11
SE15
K-12
Overlap
2330K
2340K
2350K
2360K
2370K
2380K
2390K
1990K
2000K
2010K
2020K
2030K
1980 K
2040 K
2050 K
2060K
2070 K
2080 K
2070K
2080K
2090 K
2100 K
2110K
2120 K
2130 K
2140 K
cobU sbmC yeeY hisL hisB hisI wcaM wcaJ nudD wcaE wcaA yegH
dacD yeeZ hisG hisH cld wcaL cpsG fcl wcaD wzc
yeeX sbcB yoeB hisD hisA ugd rfbC wcaK cpsB gmd wcaC wzb
cobS yeeA yeeF yefM hisC hisF gnd galF wzxC wcaI wcaF wcaB wza

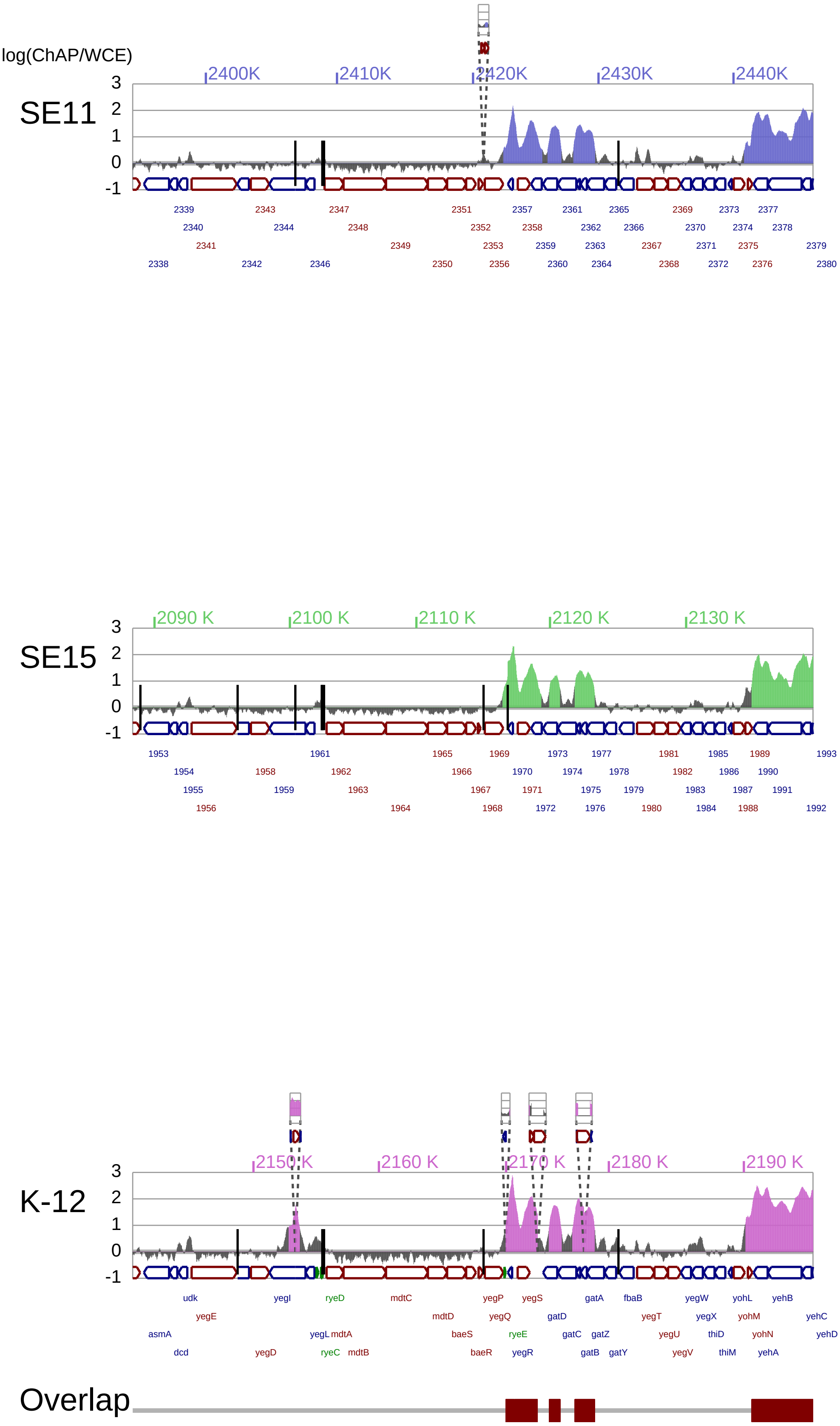
log(ChAP/WCE)
SE11
2400K
2410K
2420K
2430K
2440K
3
2
1
0
-1
2339 2343 2347 2351 2357 2361 2365 2369 2373 2377
2340 2344 2348 2352 2358 2362 2366 2370 2374 2378
2341 2349 2353 2359 2363 2367 2371 2375 2379
2338 2342 2346 2350 2356 2360 2364 2368 2372 2376 2380
SE15
2090 K
2100 K
2110 K
2120 K
2130 K
3
2
1
0
-1
1953 1961 1965 1969 1973 1977 1981 1985 1989 1993
1954 1958 1962 1966 1970 1974 1978 1982 1986 1990
1955 1959 1963 1967 1971 1975 1979 1983 1987 1991
1956 1964 1968 1972 1976 1980 1984 1988 1992
K-12
2150 K
2160 K
2170 K
2180 K
2190 K
3
2
1
0
-1
udk yegI ryeD mdtC yegP yegS gatA fbaB yegW yohL yehB
yegE mdtD yegQ gatD yegT yegX yohM yehC
asmA yegL mdtA baeS ryeE gatC gatZ yegU thiD yohN yehD
dcd yegD ryeC mdtB baeR yegR gatB gatY yegV thiM yehA
Overlap

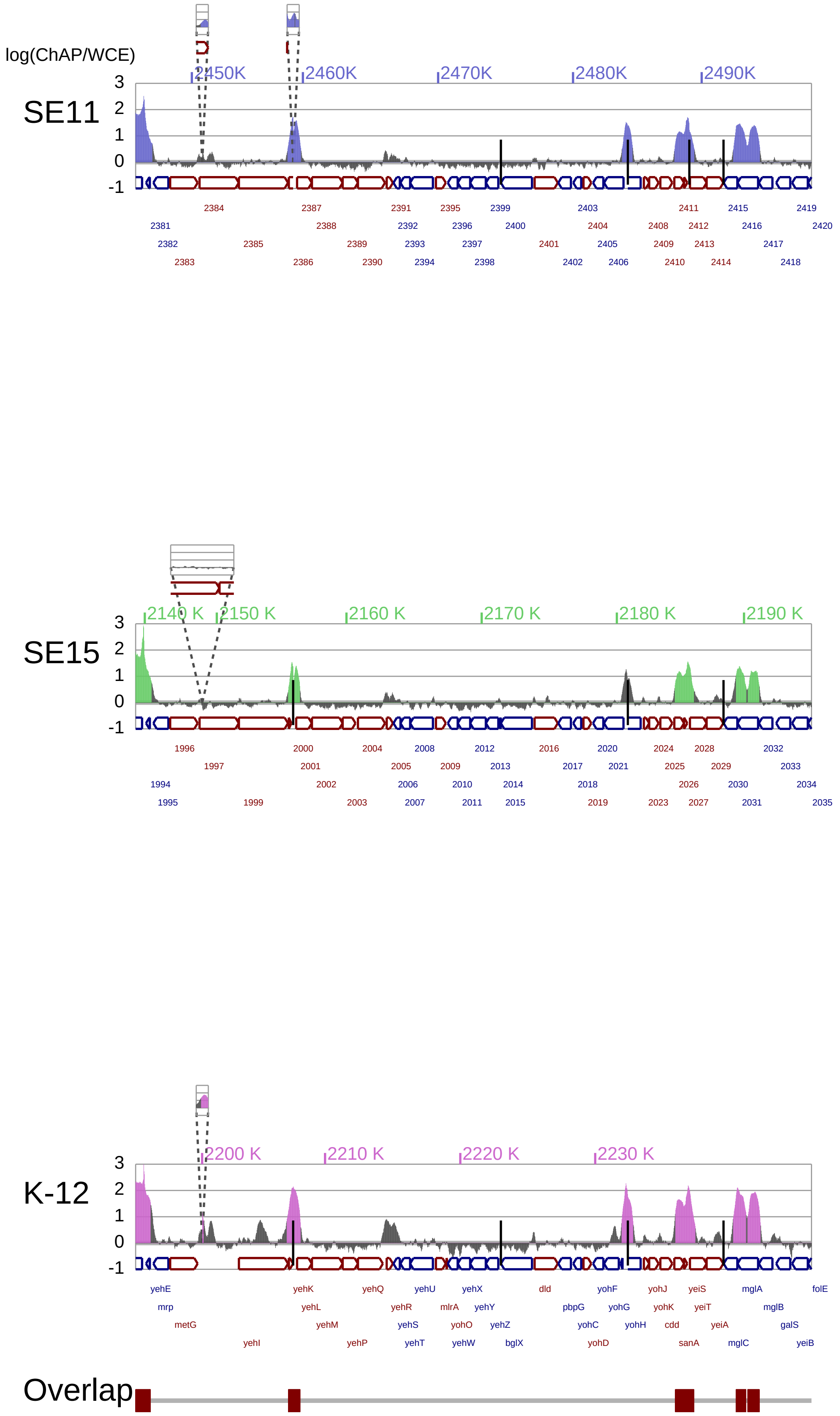

log(ChAP/WCE)
SE11
3
2
1
0
-1
2450K
2460K
2470K
2480K
2490K
2384 2387 2391 2395 2399 2403 2411 2415 2419
2381 2388 2392 2396 2400 2404 2408 2412 2416 2420
2382 2385 2389 2393 2397 2401 2405 2409 2413 2417
2383 2386 2390 2394 2398 2402 2406 2410 2414 2418
SE15
3
2
1
0
-1
2140 K
2150 K
2160 K
2170 K
2180 K
2190 K
1996 2000 2004 2008 2012 2016 2020 2024 2028 2032
1997 2001 2005 2009 2013 2017 2021 2025 2029 2033
1994 2002 2006 2010 2014 2018 2026 2030 2034
1995 1999 2003 2007 2011 2015 2019 2023 2027 2031 2035
K-12
3
2
1
0
-1
2200 K
2210 K
2220 K
2230 K
yehE yehK yehQ yehU yehX dld yohF yohJ yeiS mglA folE
mrp yehL yehR mlrA yehY pbpG yohG yohK yeiT mglB
metG yehM yehS yohO yehZ yohC yohH cdd yeiA galS
yehI yehP yehT yehW bglX yohD sanA mglC yeiB
Overlap

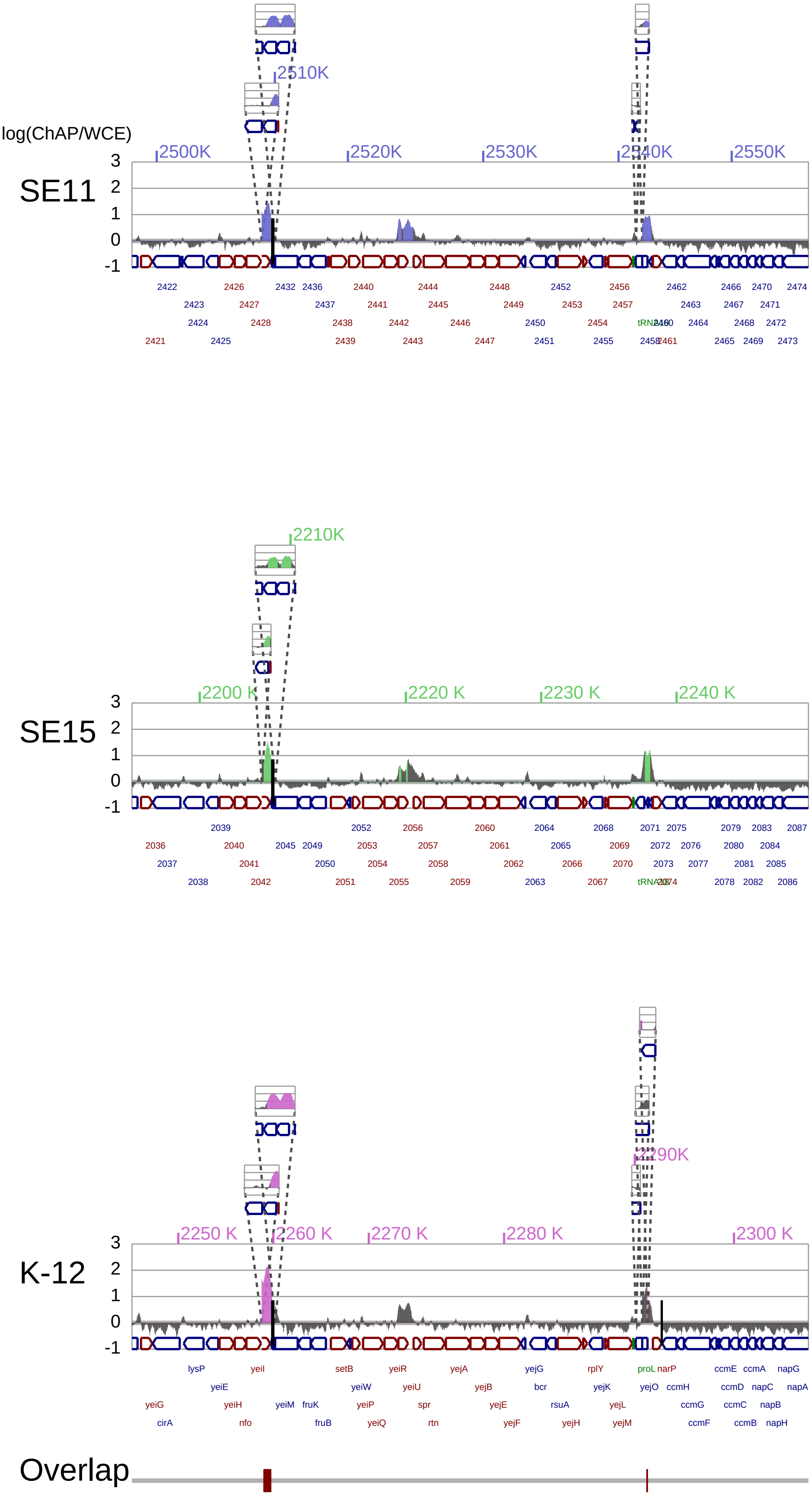

log(ChAP/WCE)
SE11
2510K
2500K
2520K
2530K
2540K
2550K
3
2
1
0
-1
2422 2426 2432 2436 2440 2444 2448 2452 2456 2462 2466 2470 2474
2423 2427 2437 2441 2445 2449 2453 2457 2463 2467 2471
2424 2428 2438 2442 2446 2450 2454 2464 2468 2472
2421 2425 2439 2443 2447 2451 2455 2461 2465 2469 2473
SE15
2210K
2200 K
2220 K
2230 K
2240 K
2039 2052 2056 2060 2064 2068 2071 2075 2079 2083 2087
2036 2040 2045 2049 2053 2057 2061 2065 2069 2072 2076 2080 2084
2037 2041 2050 2054 2058 2062 2066 2070 2073 2077 2081 2085
2038 2042 2051 2055 2059 2063 2067 2078 2082 2086
K-12
2290K
2250 K
2260 K
2270 K
2280 K
2300 K
lysP yeiI setB yeiR yejA yejG rplY proL narP ccmE ccmA napG
yeiE yeiW yeiU yejB bcr yejK yejO ccmH ccmD napC napA
yeiG yeiH yeiM fruK yeiP spr yejE rsuA yejL ccmG ccmC napB
cirA nfo fruB yeiQ rtn yejF yejH yejM ccmF ccmB napH
Overlap

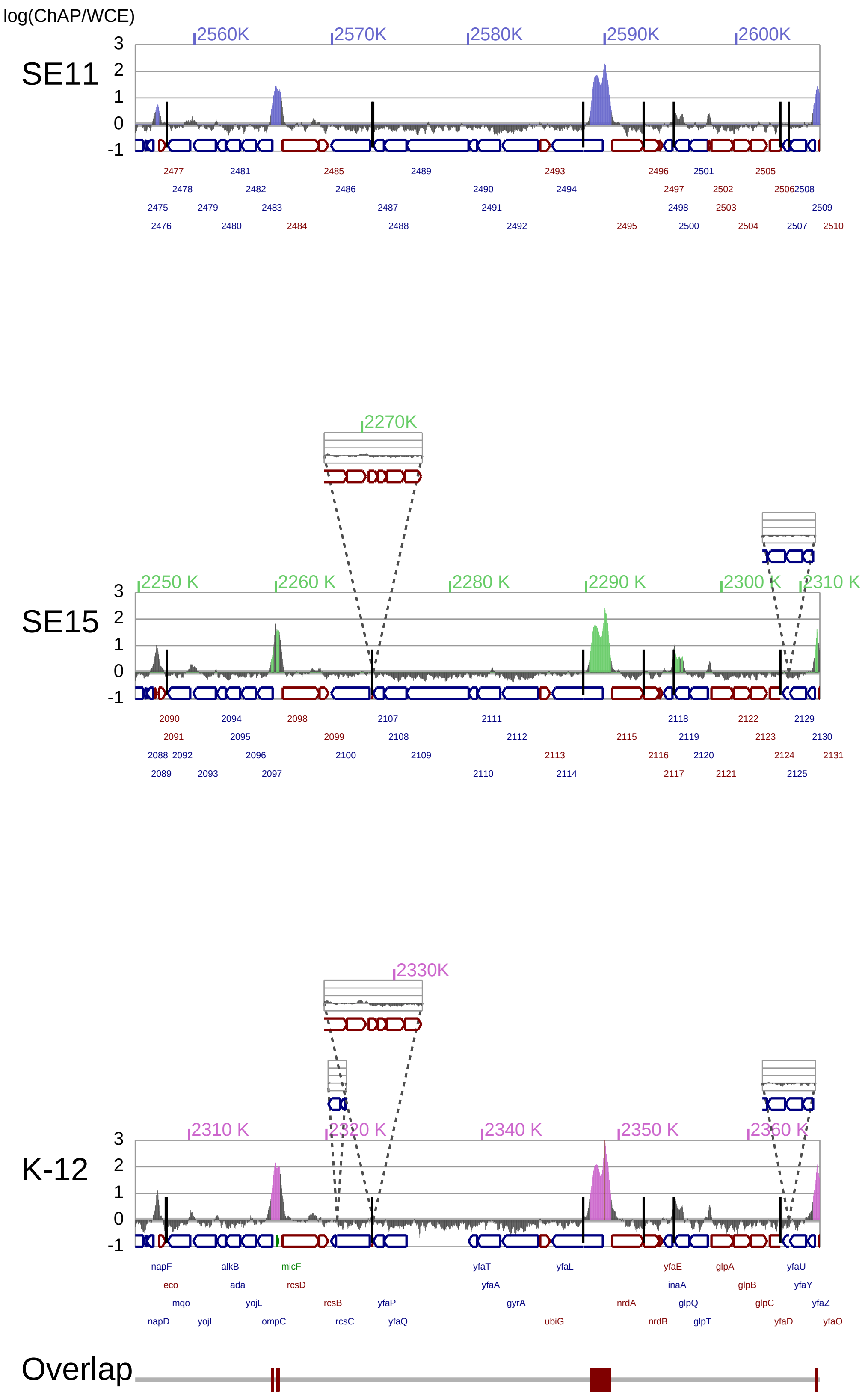
log(ChAP/WCE)
SE11
2560K
2570K
2580K
2590K
2600K
SE15
2250 K
2260 K
2270K
2280 K
2290 K
2300 K
2310 K
K-12
2310 K
2320 K
2330K
2340 K
2350 K
2360 K
napF
eco
mqo
napD
yojI
alkB
ada
yojL
ompC
micF
rcsD
rcsB
rcsC
yfaP
yfaQ
yfaT
yfaA
gyrA
yfaL
ubiG
nrdA
nrdB
yfaE
inaA
glpQ
glpT
glpA
glpB
glpC
yfaD
yfaU
yfaY
yfaZ
yfaO
Overlap

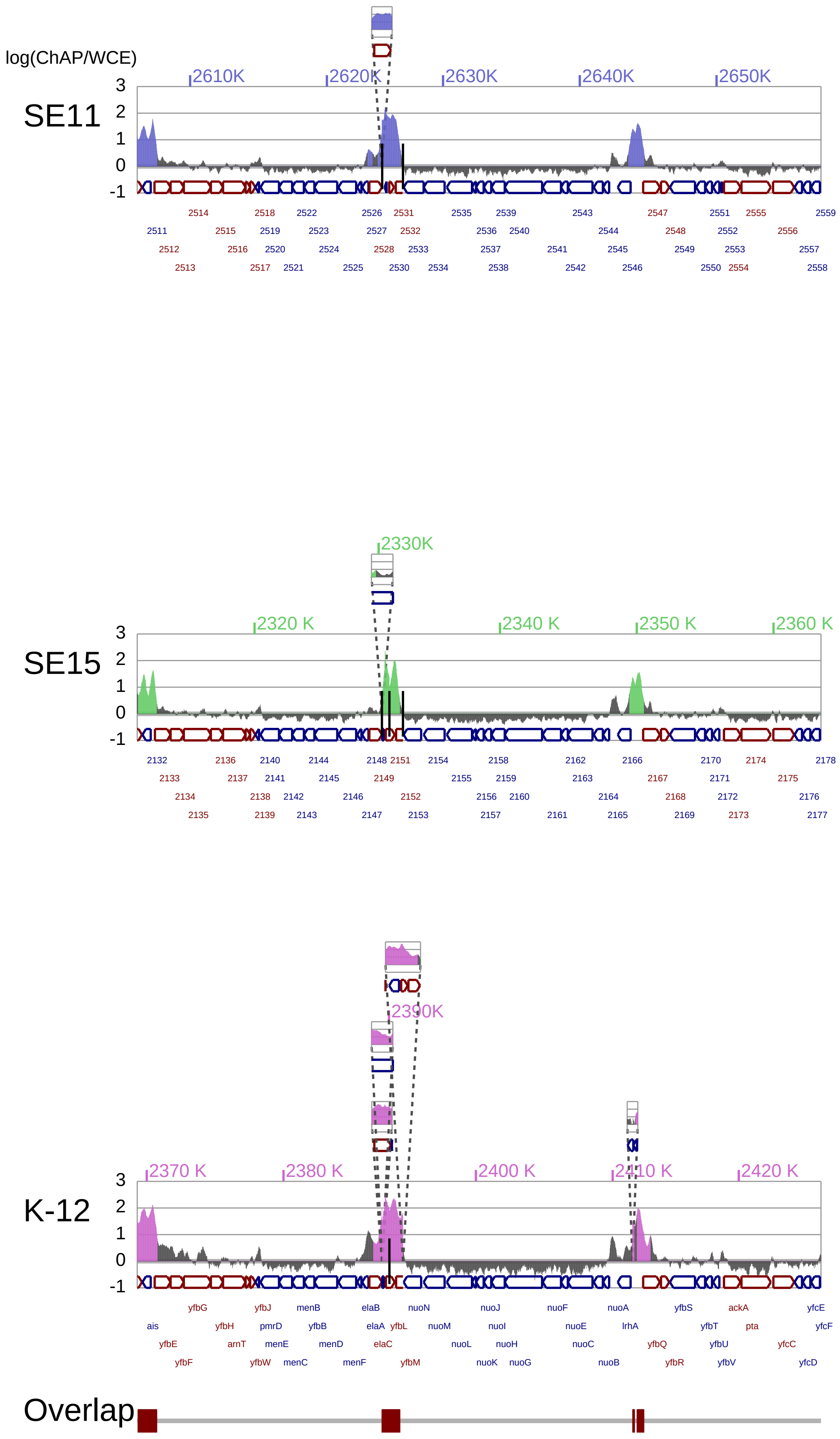
log(ChAP/WCE)
SE11
2610K
2620K
2630K
2640K
2650K
SE15
2320 K
2330K
2340 K
2350 K
2360 K
K-12
2370 K
2380 K
2390K
2400 K
2410 K
2420 K
yfbG yfbJ menB elaB nuoN nuoJ nuoF nuoA yfbS ackA yfcE
ais yfbH pmrD yfbB elaA yfbL nuoM nuoI nuoE lrhA yfbT pta yfcF
yfbE arnT menE menD elaC nuoL nuoH nuoC yfbQ yfbU yfcC
yfbF yfbW menC menF yfbM nuoK nuoG nuoB yfbR yfbV yfcD
Overlap

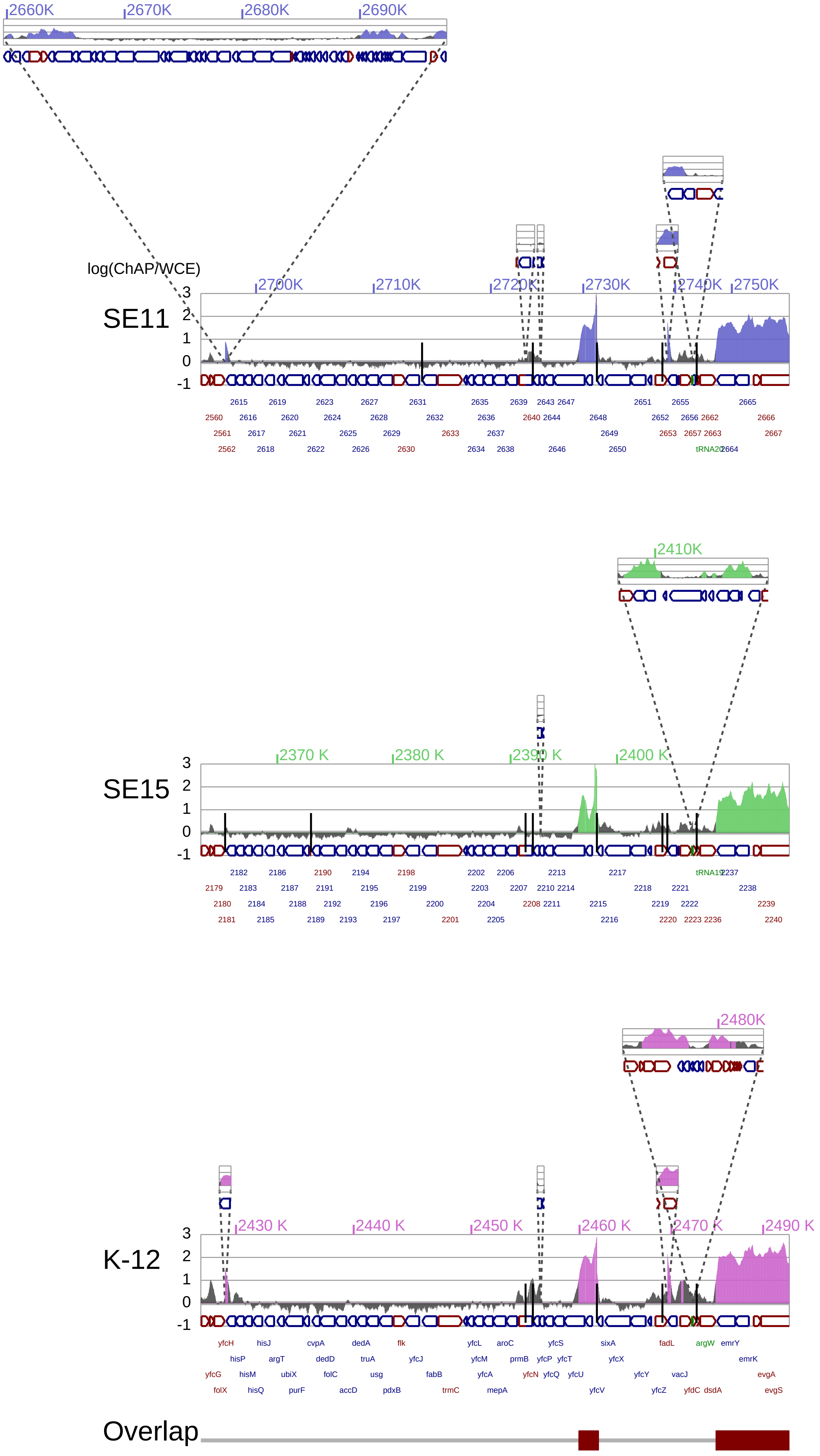

2660K
2670K
2680K
2690K
log(ChAP/WCE)
SE11
2700K
2710K
2720K
2730K
2740K
2750K
3
2
1
0
-1
2615 2619 2623 2627 2631 2635 2639 2643 2647 2651 2655 2665
2560 2616 2620 2624 2628 2632 2636 2640 2644 2648 2652 2656 2662 2666
2561 2617 2621 2625 2629 2633 2637 2649 2653 2657 2663 2667
2562 2618 2622 2626 2630 2634 2638 2646 2650 tRNA20 2664
2410K
SE15
2370 K
2380 K
2390 K
2400 K
3
2
1
0
-1
2182 2186 2190 2194 2198 2202 2206 2213 2217 tRNA19 2237
2179 2183 2187 2191 2195 2199 2203 2207 2210 2214 2218 2221 2238
2180 2184 2188 2192 2196 2200 2204 2208 2211 2215 2219 2222 2239
2181 2185 2189 2193 2197 2201 2205 2216 2220 2223 2236 2240
2480K
K-12
2430 K
2440 K
2450 K
2460 K
2470 K
2490 K
3
2
1
0
-1
yfcH hisJ cvpA dedA flk yfcL aroC yfcS sixA fadL argW emrY
hisP argT dedD truA yfcJ yfcM prmB yfcP yfcT yfcX emrK
yfcG hisM ubiX folC usg fabB yfcA yfcN yfcQ yfcU yfcY vacJ evgA
folX hisQ purF accD pdxB trmC mepA yfcV yfcZ yfdC dsdA evgS
Overlap

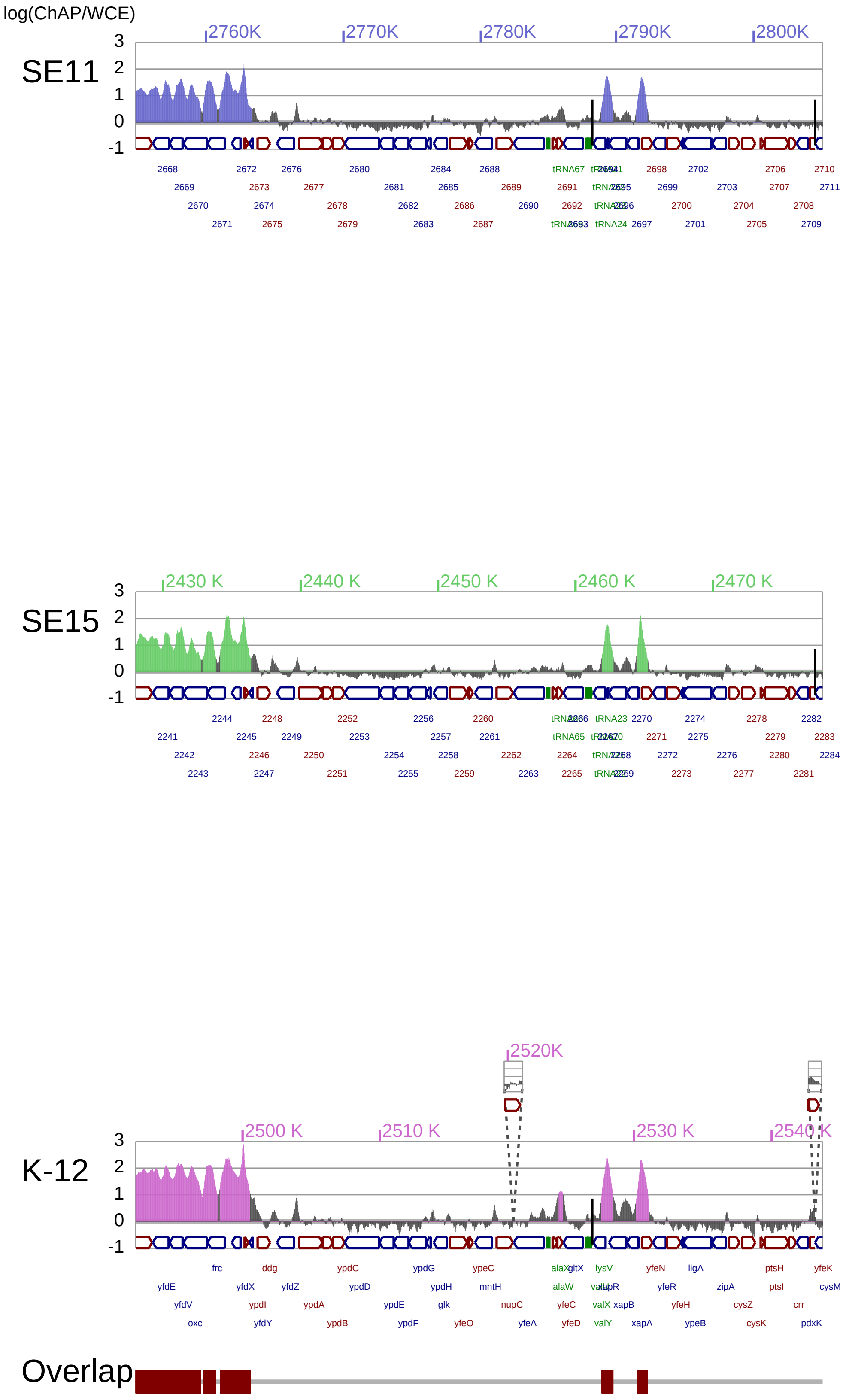

log(ChAP/WCE)
SE11
2760K
2770K
2780K
2790K
2800K
3
2
1
0
-1
2668 2669 2670 2671 2672 2673 2674 2675 2676 2677 2678 2679 2680 2681 2682 2683 2684 2685 2686 2687 2688 2689 2690 2691 2692 2697 2698 2699 2700 2701 2702 2703 2704 2705 2706 2707 2708 2709 2710 2711
tRNA67 tRNA24
SE15
2430 K
2440 K
2450 K
2460 K
2470 K
2241 2242 2243 2244 2245 2246 2247 2248 2249 2250 2251 2252 2253 2254 2255 2256 2257 2258 2259 2260 2261 2262 2263 2264 2265 2270 2271 2272 2273 2274 2275 2276 2277 2278 2279 2280 2281 2282 2283 2284
tRNA23 tRNA65
K-12
2500 K
2510 K
2520K
2530 K
2540 K
frc ddg ypdC ypdG ypeC lysV yfeN ligA ptsH yfeK
yfdE yfdX yfdZ ypdD ypdH mntH alaW yfeR zipA ptsI cysM
yfdV ypdI ypdA ypdE glk nupC yfeC valX xapB yfeH cysZ crr
oxc yfdY ypdB ypdF yfeO yfeA yfeD valY xapA ypeB cysK pdxK
Overlap

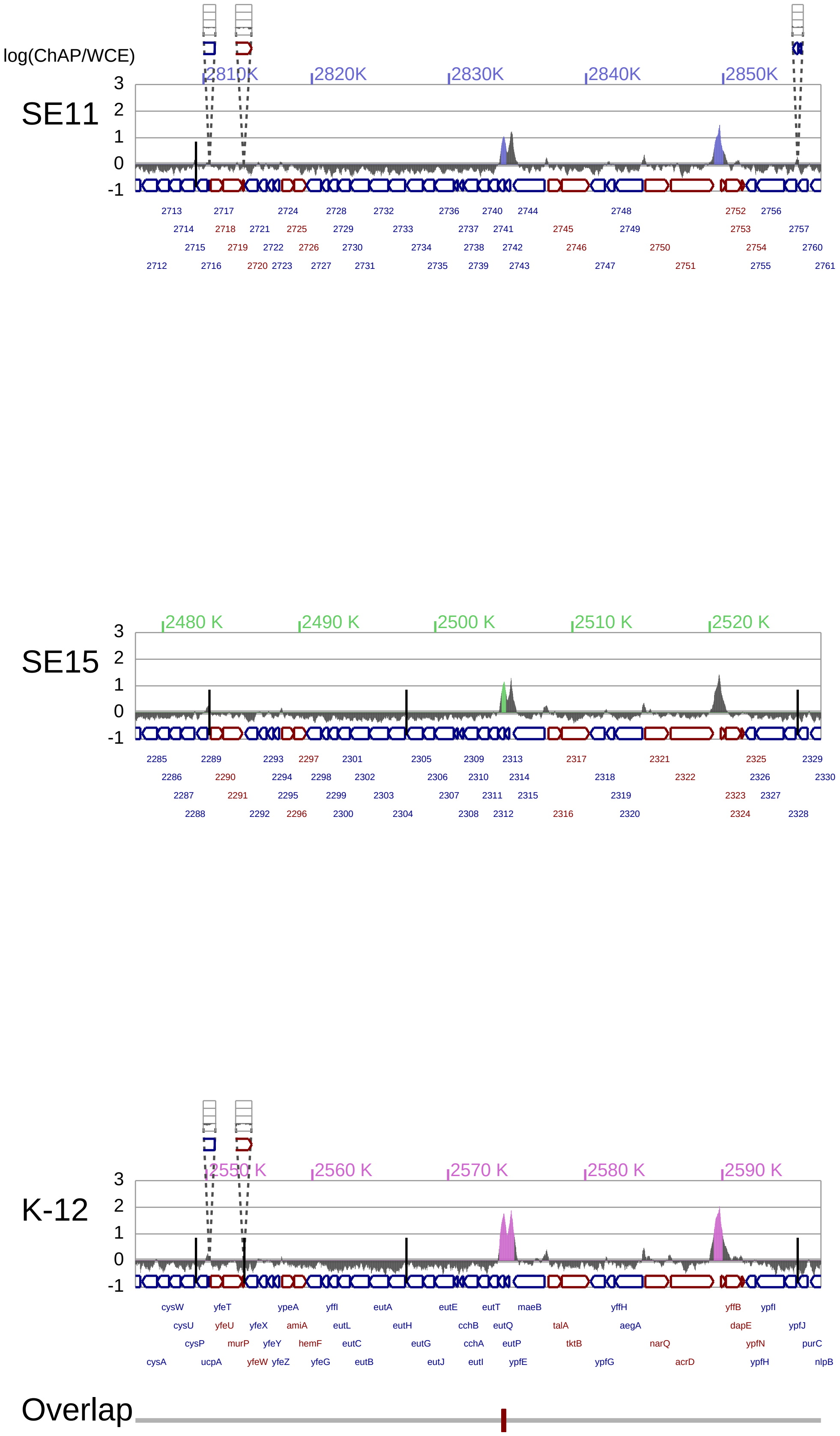
log(ChAP/WCE)
SE11
2810K
2820K
2830K
2840K
2850K
3
2
1
0
-1
2713 2717 2724 2728 2732 2736 2740 2744 2748 2752 2756
2714 2718 2721 2725 2729 2733 2737 2741 2745 2749 2753 2757
2715 2719 2722 2726 2730 2734 2738 2742 2746 2750 2754 2760
2712 2716 2720 2723 2727 2731 2735 2739 2743 2747 2751 2755 2761
SE15
2480 K
2490 K
2500 K
2510 K
2520 K
3
2
1
0
-1
2285 2289 2293 2297 2301 2305 2309 2313 2317 2321 2325 2329
2286 2290 2294 2298 2302 2306 2310 2314 2318 2322 2326 2330
2287 2291 2295 2299 2303 2307 2311 2315 2319 2323 2327
2288 2292 2296 2300 2304 2308 2312 2316 2320 2324 2328
K-12
2550 K
2560 K
2570 K
2580 K
2590 K
3
2
1
0
-1
cysW yfeT ypeA yffI eutA eutE eutT maeB yffH yffB ypfI
cysU yfeU yfeX amiA eutL eutH cchB eutQ talA aegA dapE ypfJ
cysP murP yfeY hemF eutC eutG cchA eutP tktB narQ ypfN purC
cysA ucpA yfeW yfeZ yfeG eutB eutJ eutI ypfE ypfG acrD ypfH nlpB
Overlap

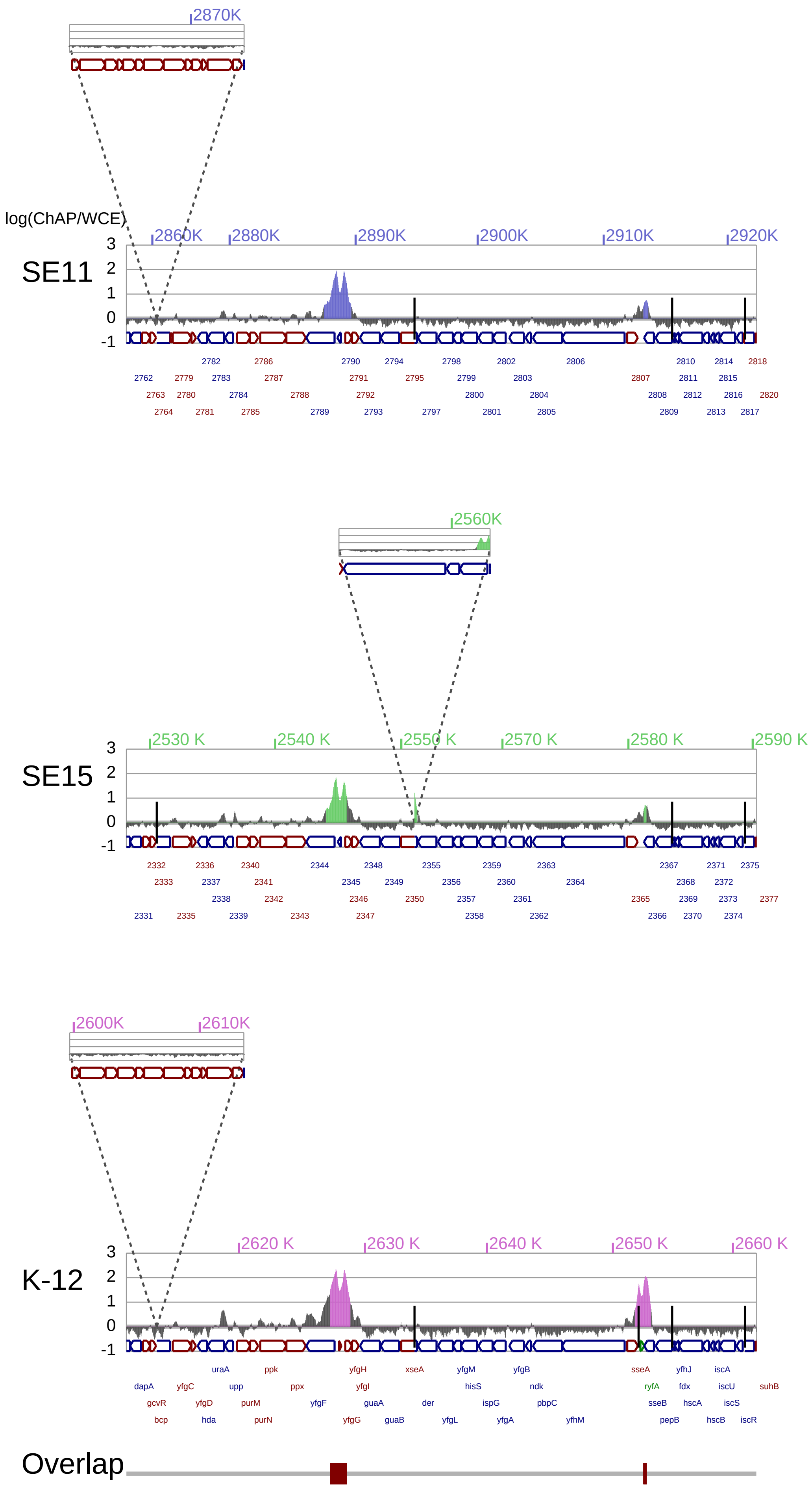
2870K
log(ChAP/WCE)
SE11
3
2
1
0
-1
2860K
2880K
2890K
2900K
2910K
2920K
2782 2786 2790 2794 2798 2802 2806 2810 2814 2818
2762 2779 2783 2787 2791 2795 2799 2803 2807 2811 2815
2763 2780 2784 2788 2792 2800 2804 2808 2812 2816 2820
2764 2781 2785 2789 2793 2797 2801 2805 2809 2813 2817
2560K
SE15
2530 K
2540 K
2550 K
2570 K
2580 K
2590 K
2332 2336 2340 2344 2348 2355 2359 2363 2367 2371 2375
2333 2337 2341 2345 2349 2356 2360 2364 2368 2372
2338 2342 2346 2350 2357 2361 2365 2369 2373 2377
2331 2335 2339 2343 2347 2358 2362 2366 2370 2374
2600K
2610K
K-12
2620 K
2630 K
2640 K
2650 K
2660 K
uraA ppk yfgH xseA yfgM yfgB sseA yfhJ iscA
dapA yfgC upp ppx yfgI hisS ndk ryfA fdx iscU suhB
gcvR yfgD purM yfgF guaA der ispG pbpC sseB hscA iscS
bcp hda purN yfgG guaB yfgL yfgA yfhM pepB hscB iscR
Overlap

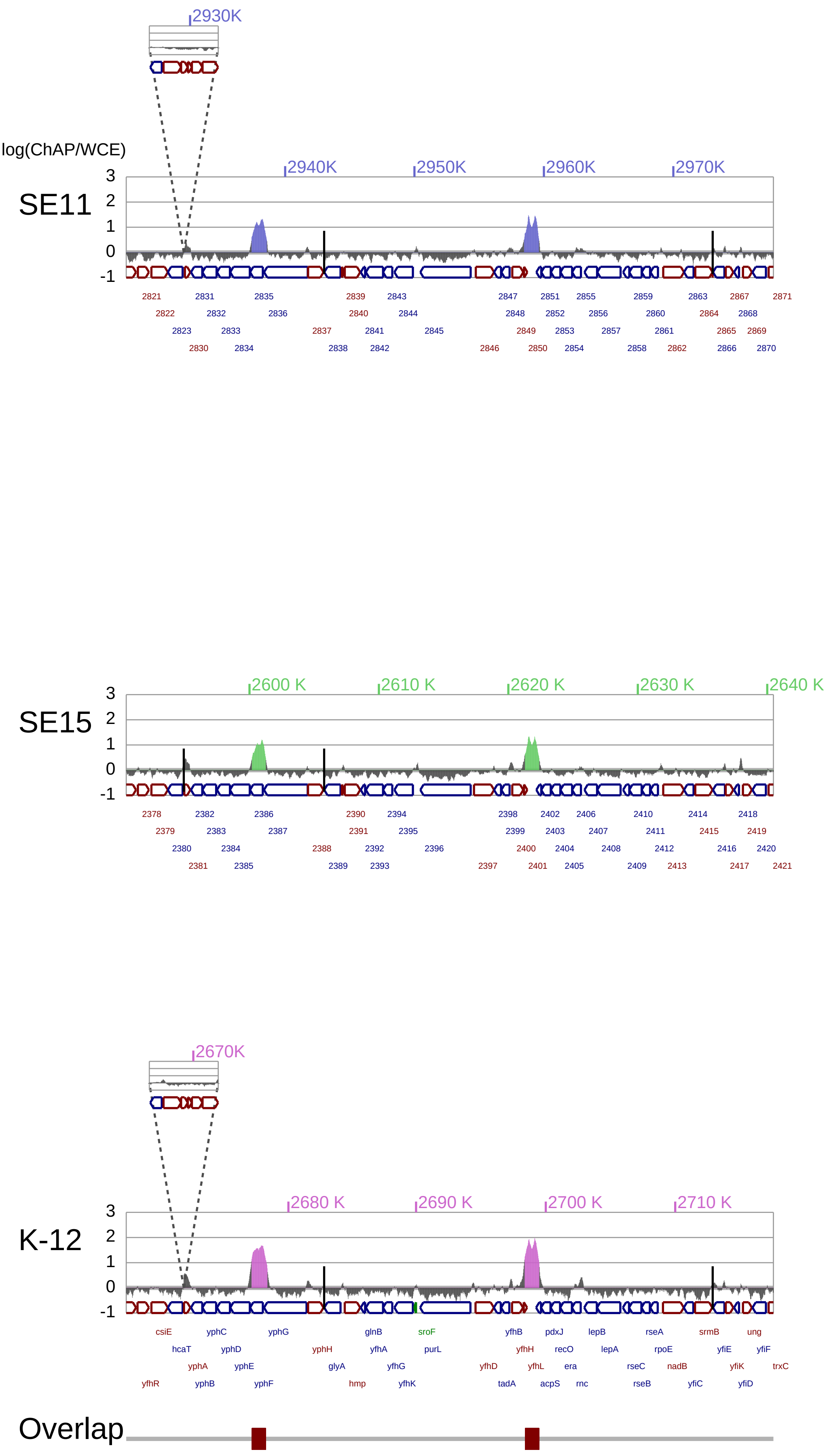

2930K
log(ChAP/WCE)
SE11
2940K
2950K
2960K
2970K
3
2
1
0
-1
2821 2831 2835 2839 2843 2847 2851 2855 2859 2863 2867 2871
2822 2832 2836 2840 2844 2848 2852 2856 2860 2864 2868
2823 2833 2837 2841 2845 2849 2853 2857 2861 2865 2869
2830 2834 2838 2842 2846 2850 2854 2858 2862 2866 2870
SE15
2600 K
2610 K
2620 K
2630 K
2640 K
2378 2382 2386 2390 2394 2398 2402 2406 2410 2414 2418
2379 2383 2387 2391 2395 2399 2403 2407 2411 2415 2419
2380 2384 2388 2392 2396 2400 2404 2408 2412 2416 2420
2381 2385 2389 2393 2397 2401 2405 2409 2413 2417 2421
2670K
K-12
2680 K
2690 K
2700 K
2710 K
csiE yphC yphG glnB sroF yfhB pdxJ lepB rseA srmB ung
hcaT yphD yphH yfhA purL yfhH recO lepA rpoE yfiE yfiF
yphA yphE glyA yfhG yfhD yfhL era rseC nadB yfiK trxC
yfhR yphB yphF hmp yfhK tadA acpS rnc rseB yfiC yfiD
Overlap

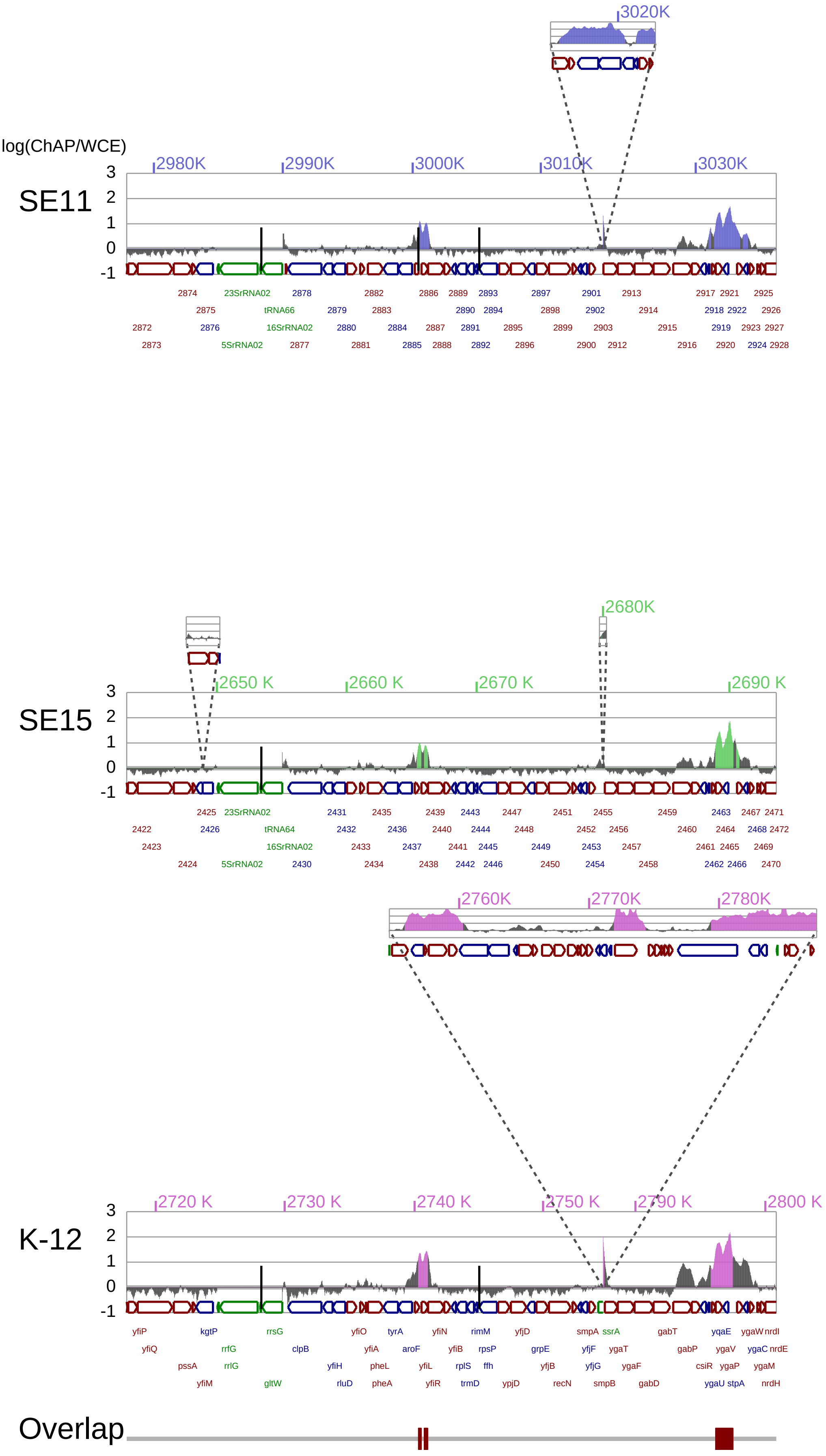
log(ChAP/WCE)
SE11
SE15
K-12
Overlap
3020K
2980K
2990K
3000K
3010K
3030K
2650 K
2660 K
2670 K
2680K
2690 K
2720 K
2730 K
2740 K
2750 K
2760K
2770K
2780K
2790 K
2800 K
23SrRNA02
tRNA66
16SrRNA02
5SrRNA02
tRNA64
yfiP yfiQ pssA yfiM kgtP rrfG rrlG rrsG gltW clpB yfiH rluD yfiO yfiA pheL pheA tyrA aroF yfiL yfiR yfiN yfiB rplS trmD rimM rpsP ffh ypjD yfjD grpE yfjB recN smpA yfjF yfjG smpB ssrA ygaT ygaF gabD gabT gabP csiR ygaU ygaE ygaV ygaP stpA ygaW nrdI ygaC nrdE ygaM nrdH

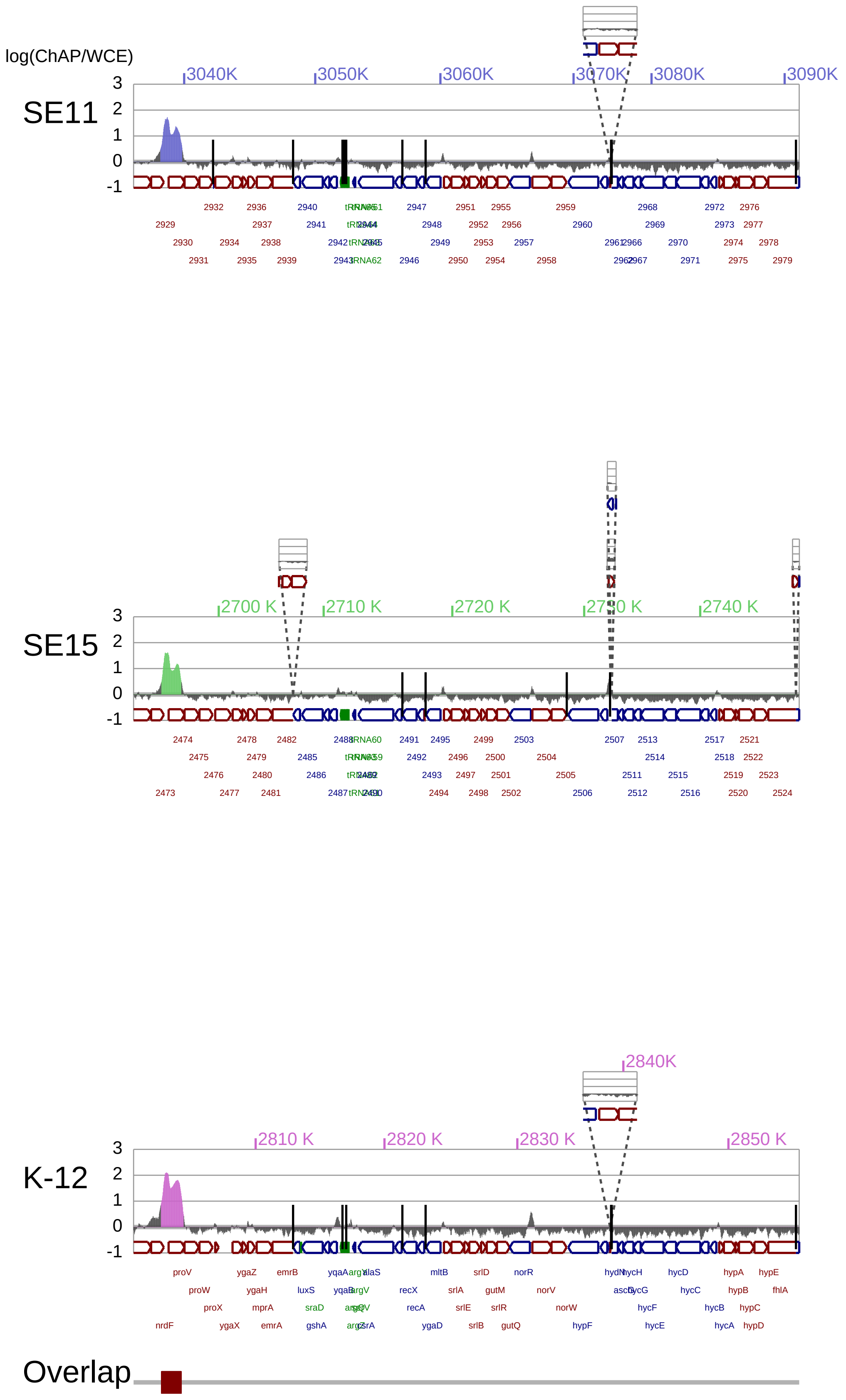

log(ChAP/WCE)
SE11
3
2
1
0
-1
3040K
3050K
3060K
3070K
3080K
3090K
2929
2930
2931
2932
2934
2935
2936
2937
2938
2939
2940
2941
2942
2943
tRNA62
2946
2947
2948
2949
2950
2951
2952
2953
2954
2955
2956
2957
2958
2959
2960
2961
2966
2967
2968
2969
2970
2971
2972
2973
2974
2975
2976
2977
2978
2979
SE15
2700 K
2710 K
2720 K
2740 K
2473
2474
2475
2476
2477
2478
2479
2480
2481
2482
2485
2486
2487
2488
tRNA60
2491
2492
2493
2494
2495
2496
2497
2498
2499
2500
2501
2502
2503
2504
2505
2506
2507
2511
2512
2513
2514
2515
2516
2517
2518
2519
2520
2521
2522
2523
2524
K-12
2810 K
2820 K
2830 K
2840K
2850 K
nrdF
proV
proW
proX
ygaZ
ygaH
ygaX
emrB
mprA
emrA
luxS
sraD
gshA
yqaA
yqaB
argV
alaS
recX
recA
ygaD
mltB
srlA
srlE
srlB
srlD
gutM
srlR
gutQ
norR
norV
norW
hypF
hycH
hycG
hycF
hycE
hycD
hycC
hycB
hycA
hypA
hypB
hypC
hypD
hypE
fhlA
Overlap

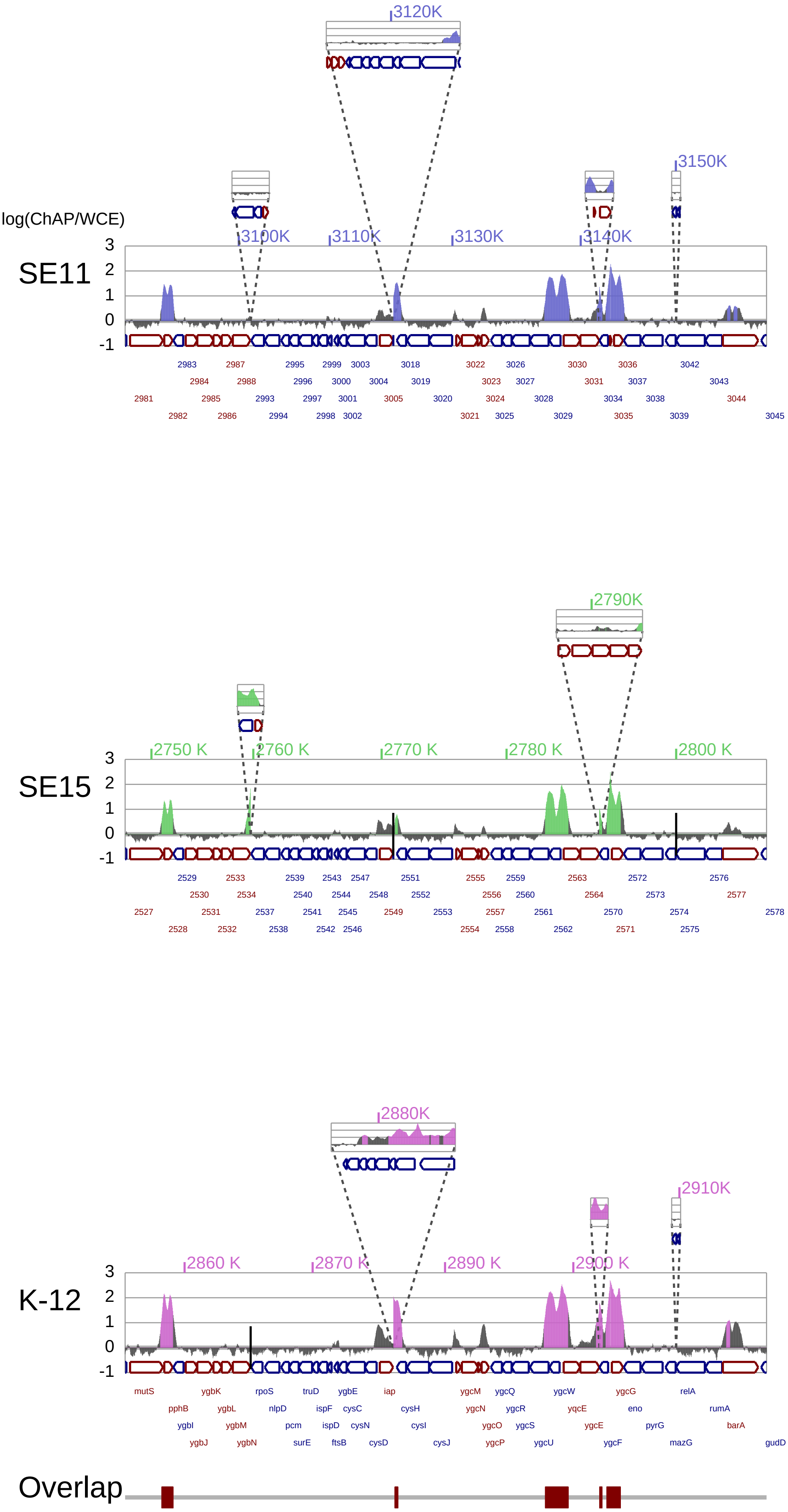

log(ChAP/WCE)
SE11
3120K
3150K
3100K
3110K
3130K
3140K
3
2
1
0
-1
2983 2987 2995 2999 3003 3018 3022 3026 3030 3036 3042
2984 2988 2996 3000 3004 3019 3023 3027 3031 3037 3043
2981 2985 2993 2997 3001 3005 3020 3024 3028 3034 3038 3044
2982 2986 2994 2998 3002 3021 3025 3029 3035 3039 3045
SE15
2790K
2750 K
2760 K
2770 K
2780 K
2800 K
2529 2533 2539 2543 2547 2551 2555 2559 2563 2572 2576
2530 2534 2540 2544 2548 2552 2556 2560 2564 2573 2577
2527 2531 2537 2541 2545 2549 2553 2557 2561 2570 2574 2578
2528 2532 2538 2542 2546 2554 2558 2562 2571 2575
K-12
2880K
2910K
2860 K
2870 K
2890 K
2900 K
mutS ygbK rpoS truD ygbE iap ygcM ygcQ ygcW ygcG relA
pphB ygbL nlpD ispF cysC cysH ygcN ygcR yqcE eno rumA
ygbI ygbM pcm ispD cysN cysI ygcO ygcS ygcE pyrG barA
ygbJ ygbN surE ftsB cysD cysJ ygcP ygcU ygcF mazG gudD
Overlap

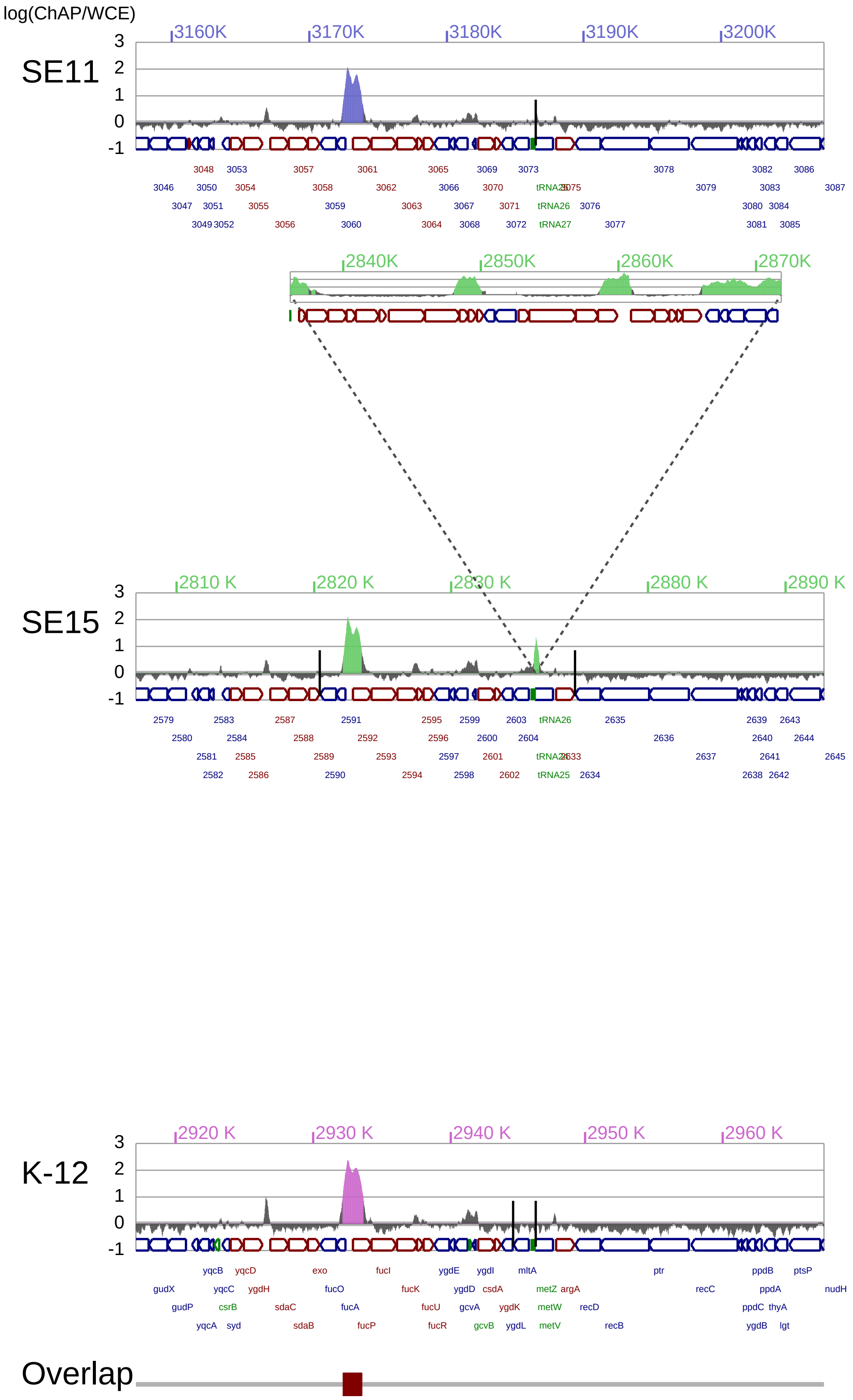
log(ChAP/WCE)
SE11
3160K
3170K
3180K
3190K
3200K
3
2
1
0
-1
3048 3053 3057 3061 3065 3069 3073 3078 3082 3086
3046 3050 3054 3058 3062 3066 3070 tRNA25 3075 3079 3083 3087
3047 3051 3055 3059 3063 3067 3071 tRNA26 3076 3080 3084
3049 3052 3056 3060 3064 3068 3072 tRNA27 3077 3081 3085
2840K
2850K
2860K
2870K
SE15
2810 K
2820 K
2830 K
2880 K
2890 K
3
2
1
0
-1
2579 2583 2587 2591 2595 2599 2603 tRNA26 2635 2639 2643
2580 2584 2588 2592 2596 2600 2604 2636 2640 2644
2581 2585 2589 2593 2597 2601 tRNA24 2633 2637 2641 2645
2582 2586 2590 2594 2598 2602 tRNA25 2634 2638 2642
K-12
2920 K
2930 K
2940 K
2950 K
2960 K
3
2
1
0
-1
yqcB yqcD exo fucI ygdE ygdI mltA ptr ppdB ptsP
gudX yqcC ygdH fucO fucK ygdD csdA metZ argA recC ppdA nudH
gudP csrB sdaC fucA fucU gcvA ygdK metW recD ppdC thyA
yqcA syd sdaB fucP fucR gcvB ygdL metV recB ygdB lgt
Overlap

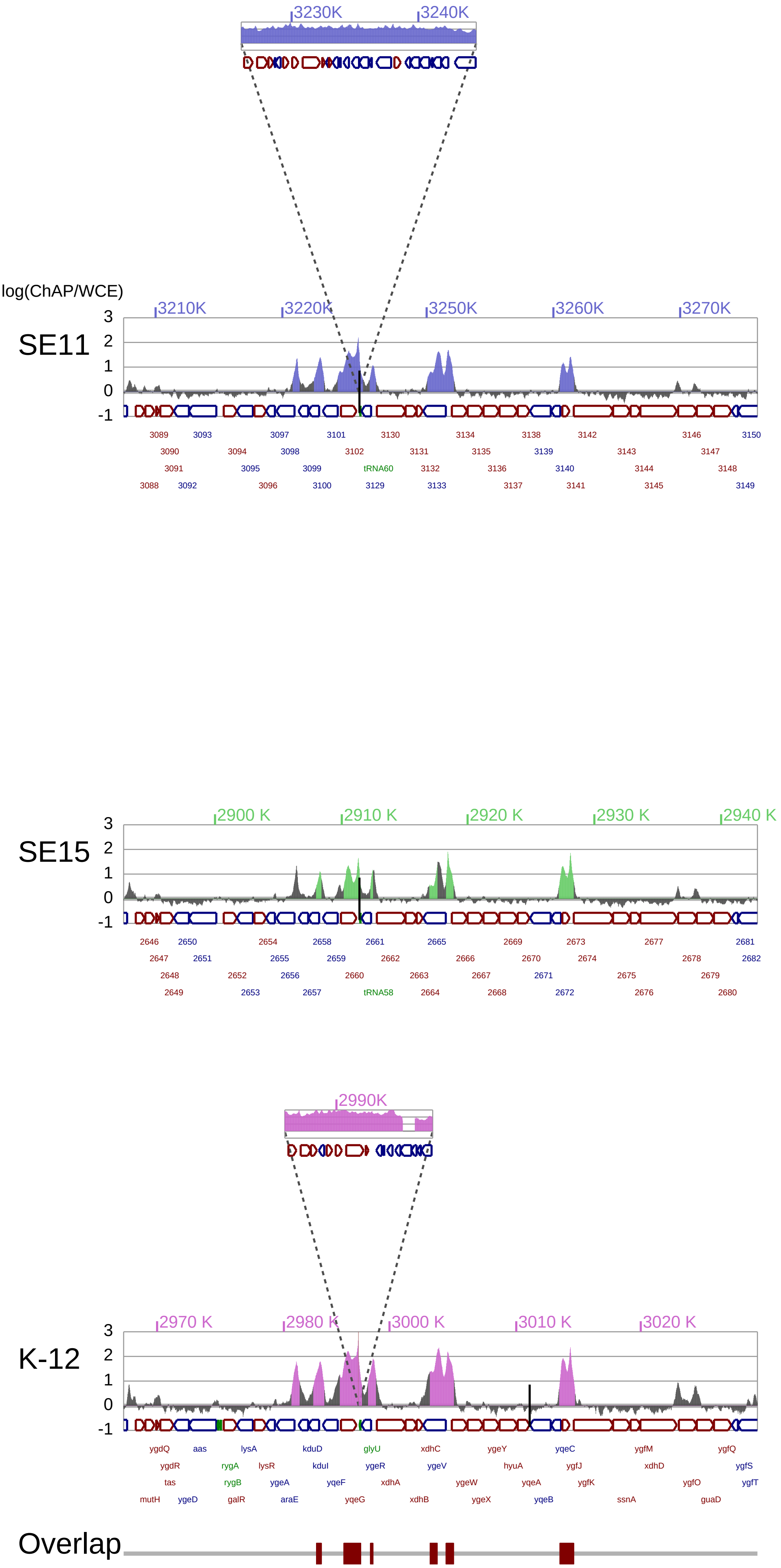
3230K
3240K
log(ChAP/WCE)
3210K
3220K
3250K
3260K
3270K
SE11
3
2
1
0
-1
3089 3093 3097 3101 3130 3134 3138 3142 3146 3150
3090 3094 3098 3102 3131 3135 3139 3143 3147
3091 3095 3099 tRNA60 3132 3136 3140 3144 3148
3088 3092 3096 3100 3129 3133 3137 3141 3145 3149
2900 K
2910 K
2920 K
2930 K
2940 K
SE15
2646 2650 2654 2658 2661 2665 2669 2673 2677 2681
2647 2651 2655 2659 2662 2666 2670 2674 2678 2682
2648 2652 2656 2660 2663 2667 2671 2675 2679
2649 2653 2657 tRNA58 2664 2668 2672 2676 2680
2990K
2970 K
2980 K
3000 K
3010 K
3020 K
K-12
ygdQ aas lysA kduD glyU xdhC ygeY yqeC ygfM ygfQ
ygdR rygA lysR kduI ygeR ygeV hyuA ygfJ xdhD ygfS
tas rygB ygeA yqeF xdhA ygeW yqeA ygfK ygfO ygfT
mutH ygeD galR araE yqeG xdhB ygeX yqeB ssnA guaD
Overlap

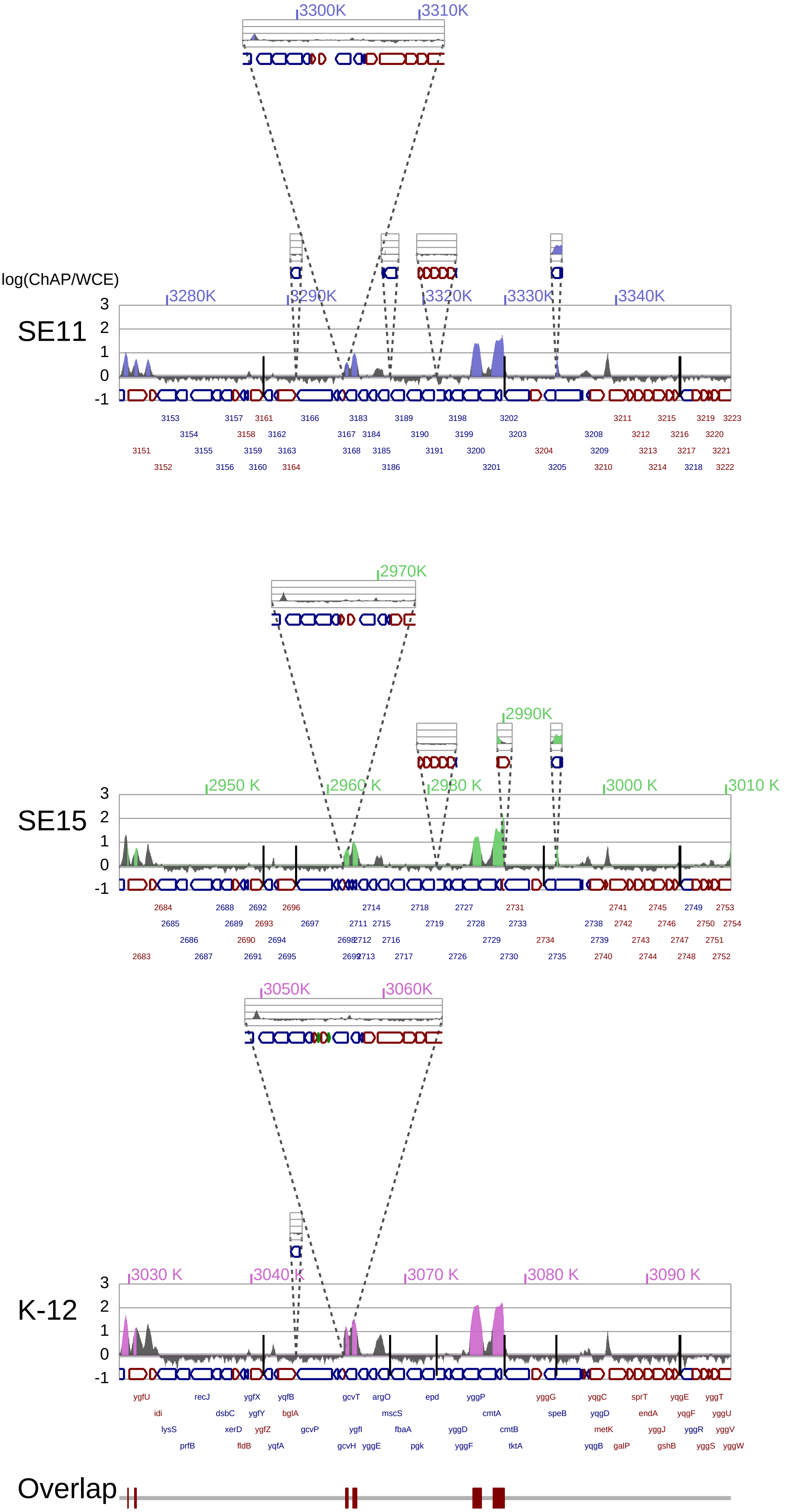

3300K
3310K
log(ChAP/WCE)
SE11
3280K
3290K
3320K
3330K
3340K
3
2
1
0
-1
3153 3157 3161 3166 3183 3189 3198 3202 3211 3215 3219 3223
3154 3158 3162 3167 3184 3190 3199 3203 3208 3212 3216 3220
3151 3155 3159 3163 3168 3185 3191 3200 3204 3209 3213 3217 3221
3152 3156 3160 3164 3186 3201 3205 3210 3214 3218 3222
2970K
2990K
SE15
2950 K
2960 K
2980 K
3000 K
3010 K
3
2
1
0
-1
2684 2688 2692 2696 2714 2718 2727 2731 2741 2745 2749 2753
2685 2689 2693 2697 2711 2715 2719 2728 2733 2738 2742 2746 2750 2754
2686 2690 2694 2698 2712 2716 2729 2734 2739 2743 2747 2751
2683 2687 2691 2695 2699 2713 2717 2726 2730 2735 2740 2744 2748 2752
3050K
3060K
K-12
3030 K
3040 K
3070 K
3080 K
3090 K
3
2
1
0
-1
ygfU recJ ygfX yqfB gcvT argO epd yggP yggG yqgC sprT yqgE yggT
idi dsbC ygfY bglA mscS cmtA speB yqgD endA yqgF yggU
lysS xerD ygfZ gcvP ygfI fbaA yggD cmtB metK yggJ yggR yggV
prfB fldB yqfA gcvH yggE pgk yggF tktA yqgB galP gshB yggS yggW
Overlap

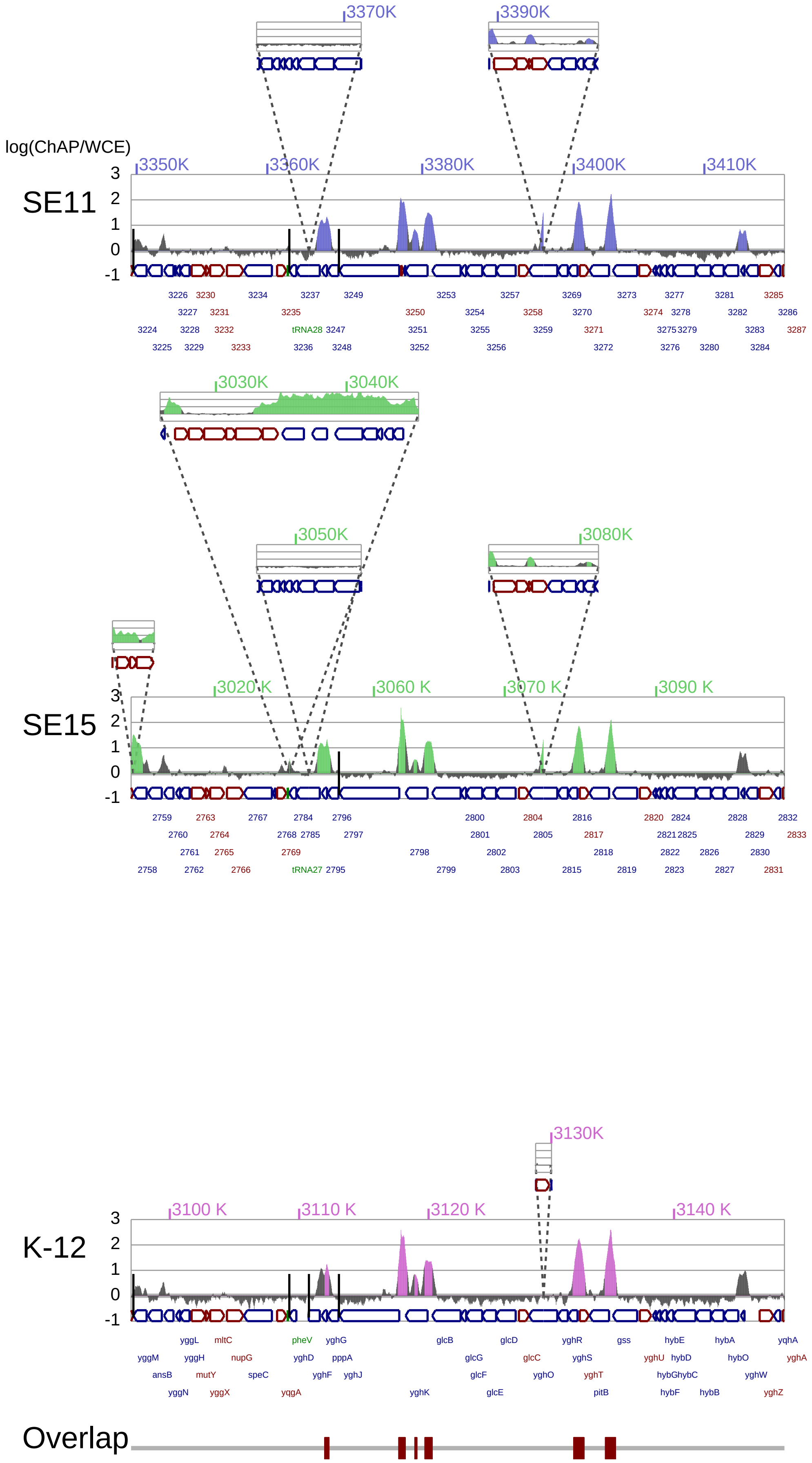

log(ChAP/WCE)
SE11
SE15
K-12
Overlap
3
2
1
0
-1
3370K
3390K
3350K
3360K
3380K
3400K
3410K
3226 3230 3234 3237 3249 3253 3257 3269 3273 3277 3281 3285
3227 3231 3235 3250 3254 3258 3270 3274 3278 3282 3286
3224 3228 3232 tRNA28 3247 3251 3255 3259 3271 3275 3279 3283 3287
3225 3229 3233 3236 3248 3252 3256 3272 3276 3280 3284
3030K
3040K
3050K
3080K
3020 K
3060 K
3070 K
3090 K
2759 2763 2767 2784 2796 2800 2804 2816 2820 2824 2828 2832
2760 2764 2768 2785 2797 2801 2805 2817 2821 2825 2829 2833
2761 2765 2769 2798 2802 2818 2822 2826 2830
2758 2762 2766 tRNA27 2795 2799 2803 2815 2819 2823 2827 2831
3130K
3100 K
3110 K
3120 K
3140 K
yggL mltC pheV yghG glcB glcD yghR gss hybE hybA yqhA
yggM yggH nupG yghD pppA glcG glcC yghS yghU hybD hybO yghA
ansB mutY speC yghF yghJ glcF yghO yghT hybGhybC yghW
yggN yggX yqgA yghK glcE pitB hybF hybB yghZ

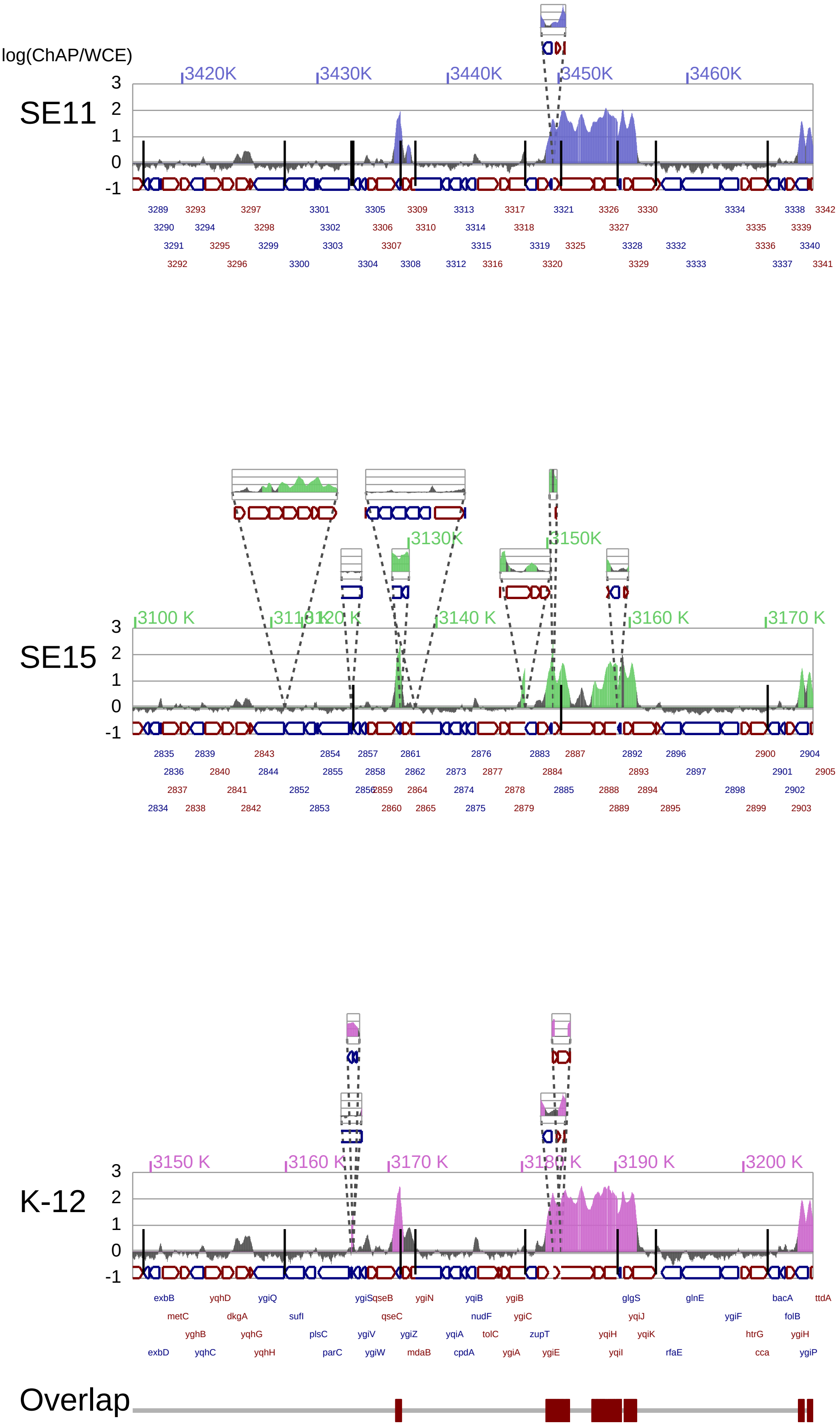
log(ChAP/WCE)
SE11
SE15
K-12
Overlap
3420K
3430K
3440K
3450K
3460K
3100 K
3110 K
3120 K
3130 K
3140 K
3150 K
3160 K
3170 K
3180 K
3190 K
3200 K
exbB
yqhD
ygiQ
ygiS
qseB
ygiN
yqiB
ygiB
glgS
glnE
bacA
ttdA
metC
dkgA
sufI
qseC
nudF
ygiC
yqiJ
ygiF
folB
yghB
yqhG
plsC
ygiV
ygiZ
yqiA
tolC
zupT
yqiH
yqiK
htrG
ygiH
exbD
yqhC
yqhH
parC
ygiW
mdaB
cpdA
ygiA
ygiE
yqiI
rfaE
cca
ygiP

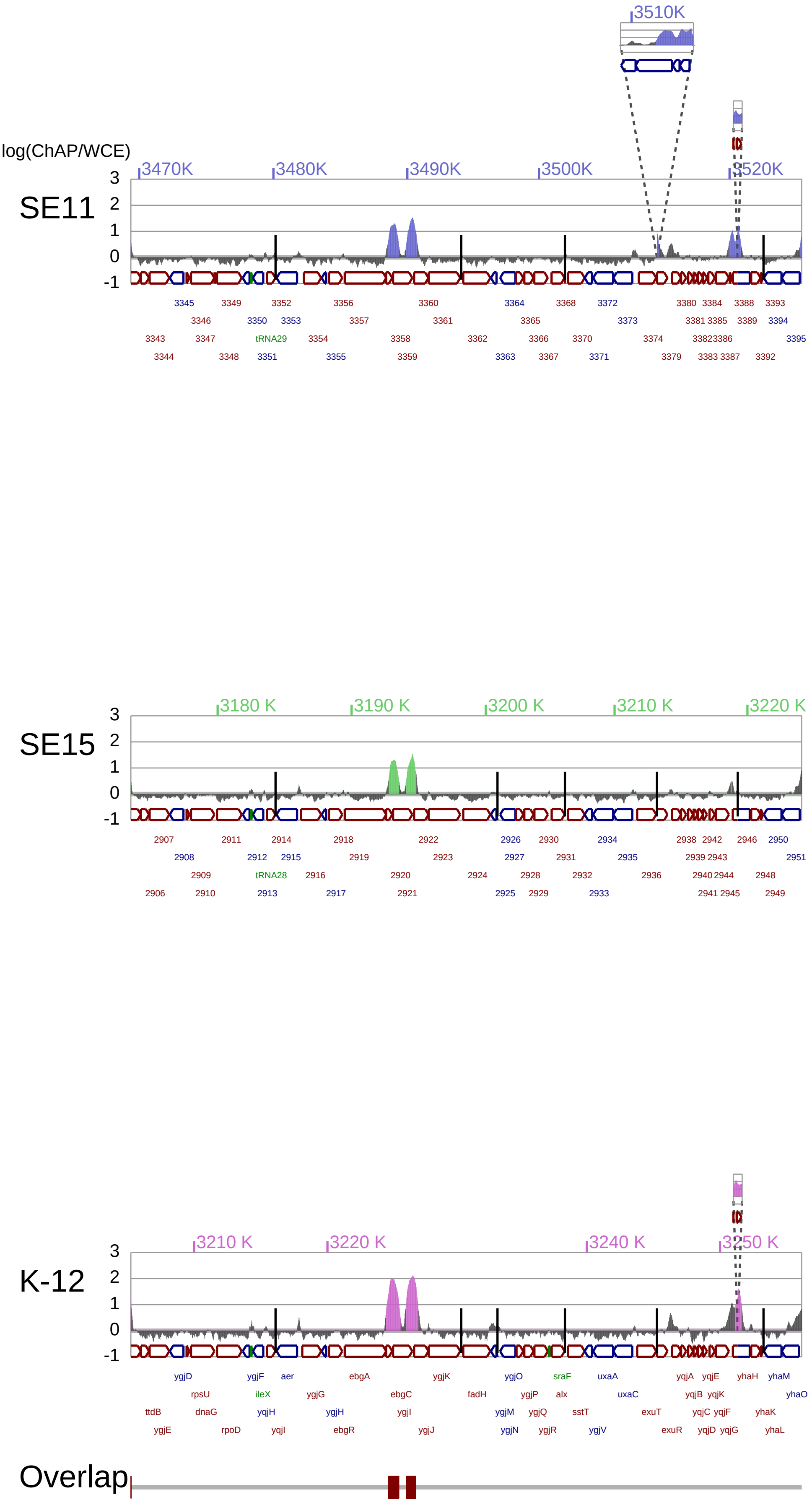
log(ChAP/WCE)
SE11
3510K
3470K
3480K
3490K
3500K
3520K
3
2
1
0
-1
3345 3349 3352 3356 3360 3364 3368 3372 3380 3384 3388 3393
3346 3350 3353 3357 3361 3365 3373 3381 3385 3389 3394
3343 3347 tRNA29 3354 3358 3362 3366 3370 3374 33823386 3395
3344 3348 3351 3355 3359 3363 3367 3371 3379 3383 3387 3392
SE15
3180 K
3190 K
3200 K
3210 K
3220 K
3
2
1
0
-1
2907 2911 2914 2918 2922 2926 2930 2934 2938 2942 2946 2950
2908 2912 2915 2919 2923 2927 2931 2935 2939 2943 2951
2909 tRNA28 2916 2920 2924 2928 2932 2936 2940 2944 2948
2906 2910 2913 2917 2921 2925 2929 2933 2941 2945 2949
K-12
3210 K
3220 K
3240 K
3250 K
3
2
1
0
-1
ygjD ygjF aer ebgA ygjK ygjO sraF uxaA yqjA yqjE yhaH yhaM
rpsU ygjG ebgC fadH ygjP alx uxaC yqjB yqjK yhaO
ttdB dnaG ileX yqjH ygjH ygjI ygjM ygjQ sstT exuT yqjC yqjF yhaK
ygjE rpoD yqjI ebgR ygjJ ygjN ygjR ygjV exuR yqjD yqjG yhaL
Overlap

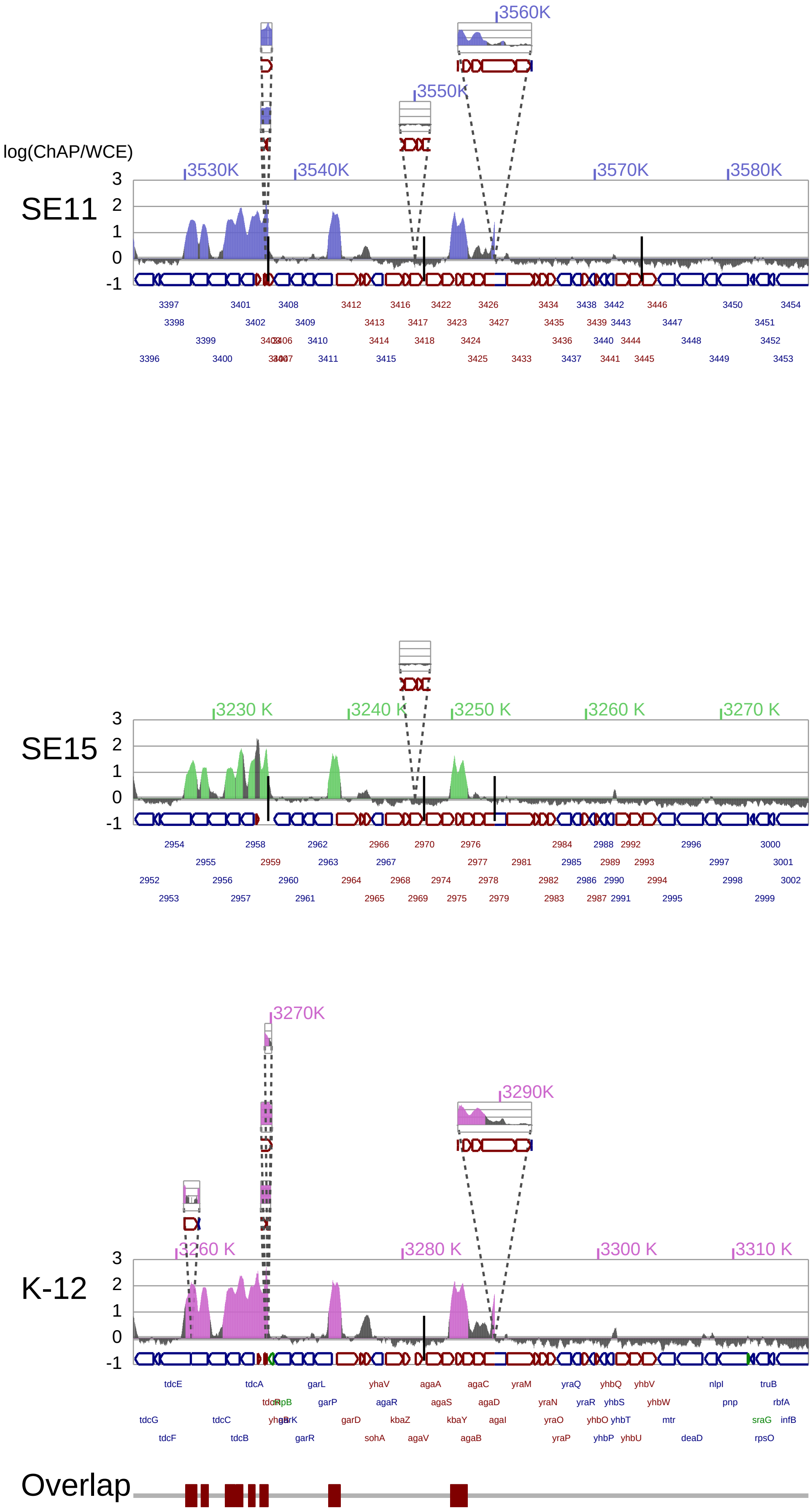
log(ChAP/WCE)
SE11
3530K
3540K
3550K
3560K
3570K
3580K
3
2
1
0
-1
3397 3401 3408 3412 3416 3422 3426 3434 3438 3442 3446 3450 3454
3398 3402 3409 3413 3417 3423 3427 3435 3439 3443 3447 3451
3399 3410 3414 3418 3424 3436 3440 3444 3448 3452
3396 3400 3411 3415 3425 3433 3437 3441 3445 3449 3453
SE15
3230 K
3240 K
3250 K
3260 K
3270 K
2954 2958 2962 2966 2970 2976 2984 2988 2992 2996 3000
2955 2959 2963 2967 2977 2981 2985 2989 2993 2997 3001
2952 2956 2960 2964 2968 2974 2978 2982 2986 2990 2994 2998 3002
2953 2957 2961 2965 2969 2975 2979 2983 2987 2991 2995 2999
K-12
3260 K
3270K
3280 K
3290K
3300 K
3310 K
tdcE tdcA garL yhaV agaA agaC yraM yraQ yhbQ yhbV nlpI truB
garP agaR agaS agaD yraN yraR yhbS yhbW pnp rbfA
tdcG tdcC garK garD kbaZ kbaY agaI yraO yhbO yhbT mtr sraG infB
tdcF tdcB garR sohA agaV agaB yraP yhbP yhbU deaD rpsO
Overlap

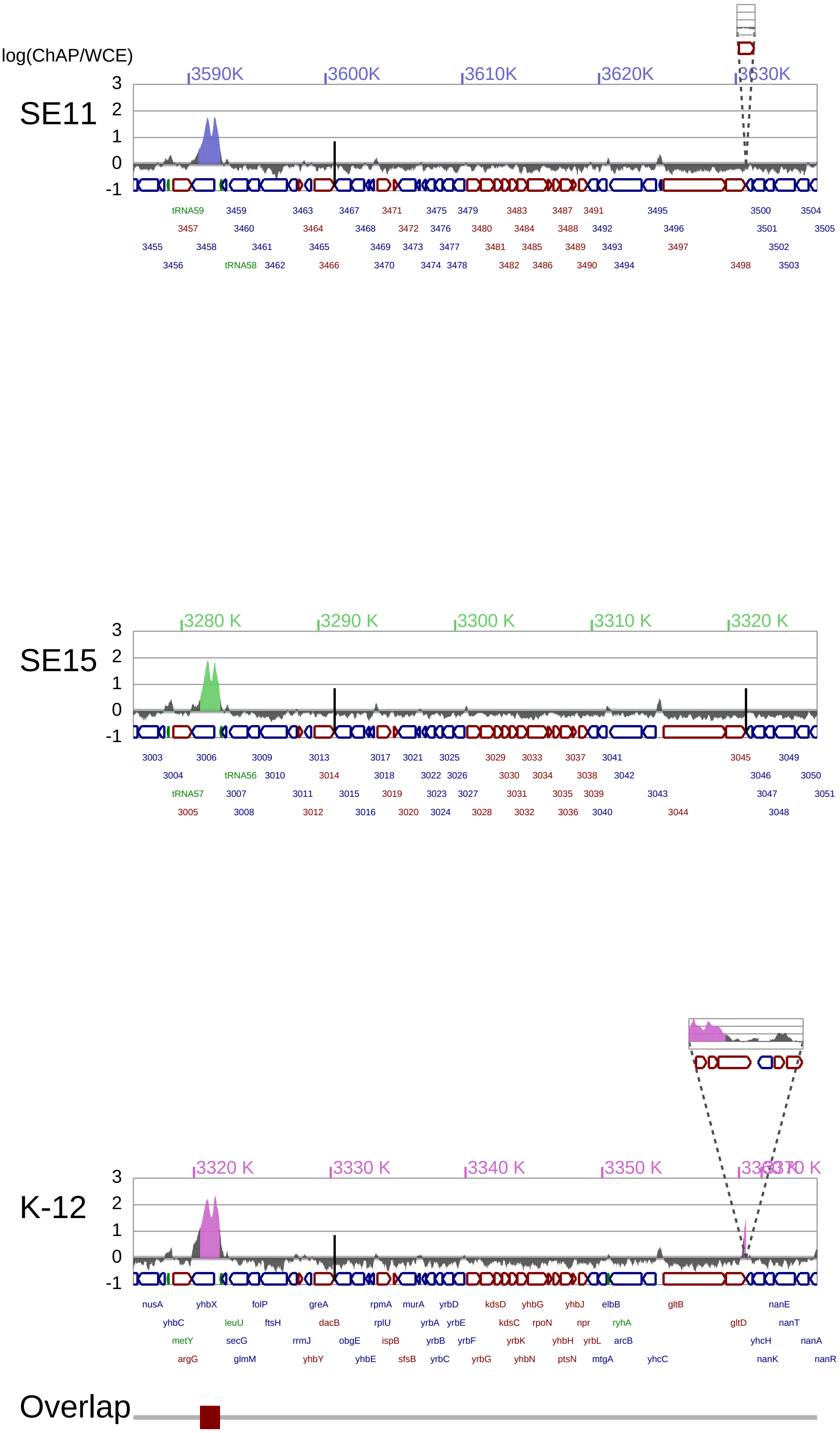

log(ChAP/WCE)
SE11
3590K
3600K
3610K
3620K
3630K
3
2
1
0
-1
tRNA59 3459 3463 3467 3471 3475 3479 3483 3487 3491 3495 3500 3504
3457 3460 3464 3468 3472 3476 3480 3484 3488 3492 3496 3501 3505
3455 3458 3461 3465 3469 3473 3477 3481 3485 3489 3493 3497 3502
3456 tRNA58 3462 3466 3470 3474 3478 3482 3486 3490 3494 3498 3503
SE15
3280 K
3290 K
3300 K
3310 K
3320 K
3
2
1
0
-1
3003 3006 3009 3013 3017 3021 3025 3029 3033 3037 3041 3045 3049
3004 tRNA56 3010 3014 3018 3022 3026 3030 3034 3038 3042 3046 3050
tRNA57 3007 3011 3015 3019 3023 3027 3031 3035 3039 3043 3047 3051
3005 3008 3012 3016 3020 3024 3028 3032 3036 3040 3044 3048
K-12
3320 K
3330 K
3340 K
3350 K
3360 K
3370 K
3
2
1
0
-1
nusA yhbX folP greA rpmA murA yrbD kdsD yhbG yhbJ elbB gltB nanE
yhbC leuU ftsH dacB rplU yrbA yrbE kdsC rpoN npr ryhA gltD nanT
metY secG rrmJ obgE ispB yrbB yrbF yrbK yhbH yrbL arcB yhcH nanA
argG glmM yhbY yhbE sfsB yrbC yrbG yhbN ptsN mtgA yhcC nanK nanR
Overlap

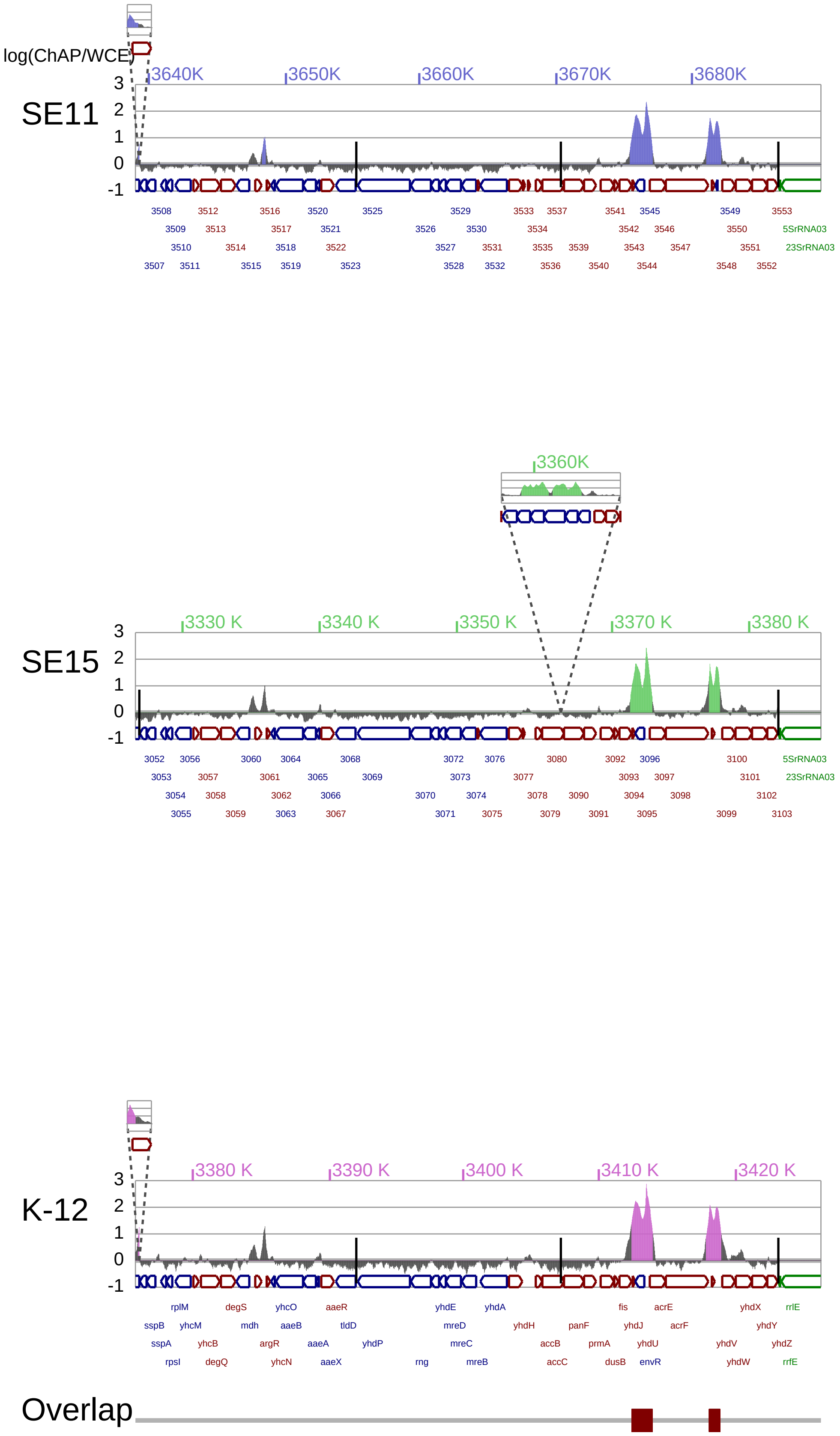

log(ChAP/WCE)
SE11
3640K
3650K
3660K
3670K
3680K
3
2
1
0
-1
3508 3512 3516 3520 3525 3529 3533 3537 3541 3545 3549 3553
3509 3513 3517 3521 3526 3530 3534 3542 3546 3550 5SrRNA03
3510 3514 3518 3522 3527 3531 3535 3539 3543 3547 3551 23SrRNA03
3507 3511 3515 3519 3523 3528 3532 3536 3540 3544 3548 3552
3360K
SE15
3330 K
3340 K
3350 K
3370 K
3380 K
3
2
1
0
-1
3052 3056 3060 3064 3068 3072 3076 3080 3092 3096 3100 5SrRNA03
3053 3057 3061 3065 3069 3073 3077 3093 3097 3101 23SrRNA03
3054 3058 3062 3066 3070 3074 3078 3090 3094 3098 3102
3055 3059 3063 3067 3071 3075 3079 3091 3095 3099 3103
K-12
3380 K
3390 K
3400 K
3410 K
3420 K
3
2
1
0
-1
rplM degS yhcO aaeR yhdE yhdA fis acrE yhdX rrlE
sspB yhcM mdh aaeB tldD mreD yhdH panF yhdJ acrF yhdY
sspA yhcB argR aaeA yhdP mreC accB prmA yhdU yhdV yhdZ
rpsI degQ yhcN aaeX rng mreB accC dusB envR yhdW rrfE
Overlap

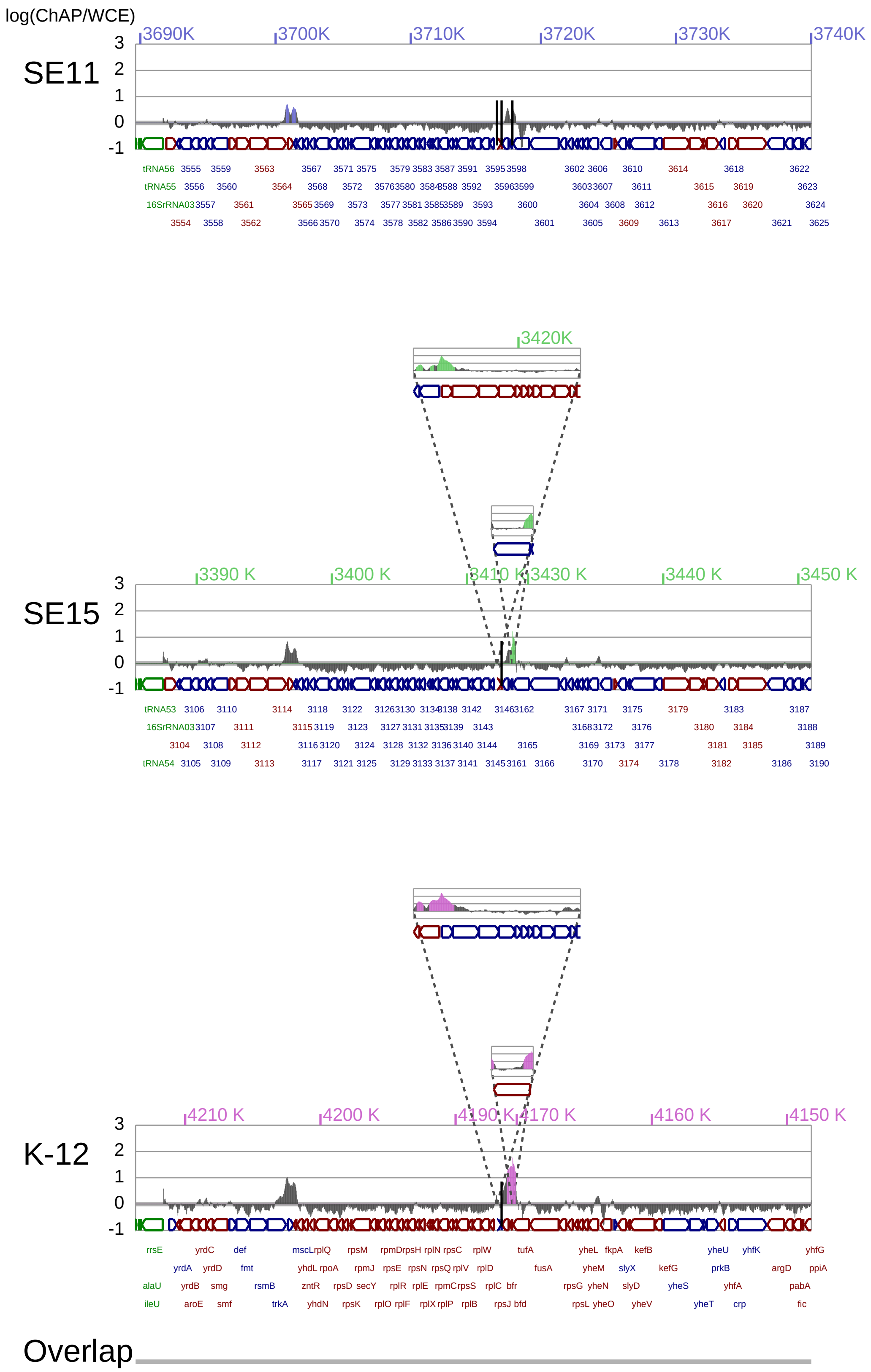
log(ChAP/WCE)
SE11
3690K
3700K
3710K
3720K
3730K
3740K
3
2
1
0
-1
tRNA56 3555 3559 3563 3567 3571 3575 3579 3583 3587 3591 3595 3598 3602 3606 3610 3614 3618 3622
tRNA55 3556 3560 3564 3568 3572 3576 3580 3584 3588 3592 3596 3599 3603 3607 3611 3615 3619 3623
16SrRNA03 3557 3561 3565 3569 3573 3577 3581 3585 3589 3593 3600 3604 3608 3612 3616 3620 3624
3554 3558 3562 3566 3570 3574 3578 3582 3586 3590 3594 3601 3605 3609 3613 3617 3621 3625
3420K
SE15
3390 K
3400 K
3410 K
3430 K
3440 K
3450 K
tRNA53 3106 3110 3114 3118 3122 3126 3130 3134 3138 3142 3146 3162 3167 3171 3175 3179 3183 3187
16SrRNA03 3107 3111 3115 3119 3123 3127 3131 3135 3139 3143 3168 3172 3176 3180 3184 3188
3104 3108 3112 3116 3120 3124 3128 3132 3136 3140 3144 3165 3169 3173 3177 3181 3185 3189
tRNA54 3105 3109 3113 3117 3121 3125 3129 3133 3137 3141 3145 3161 3166 3170 3174 3178 3182 3186 3190
K-12
4210 K
4200 K
4190 K
4170 K
4160 K
4150 K
rrsE yrdC def mscL rplQ rpsM rpmD rpsH rplN rpsC rplW tufA yheL fkpA kefB yheU yhfK yhfG
yrdA yrdD fmt yhdL rpoA rpmJ rpsE rpsN rpsQ rplV rplD fusA yheM slyX kefG prkB argD ppiA
alaU yrdB smg rsmB zntR rpsD secY rplR rplE rpmC rpsS rplC bfr rpsG yheN slyD yheS yhfA pabA
ileU aroE smf trkA yhdN rpsK rplO rplF rplX rplP rplB rpsJ bfd rpsL yheO yheV yheT crp fic
Overlap

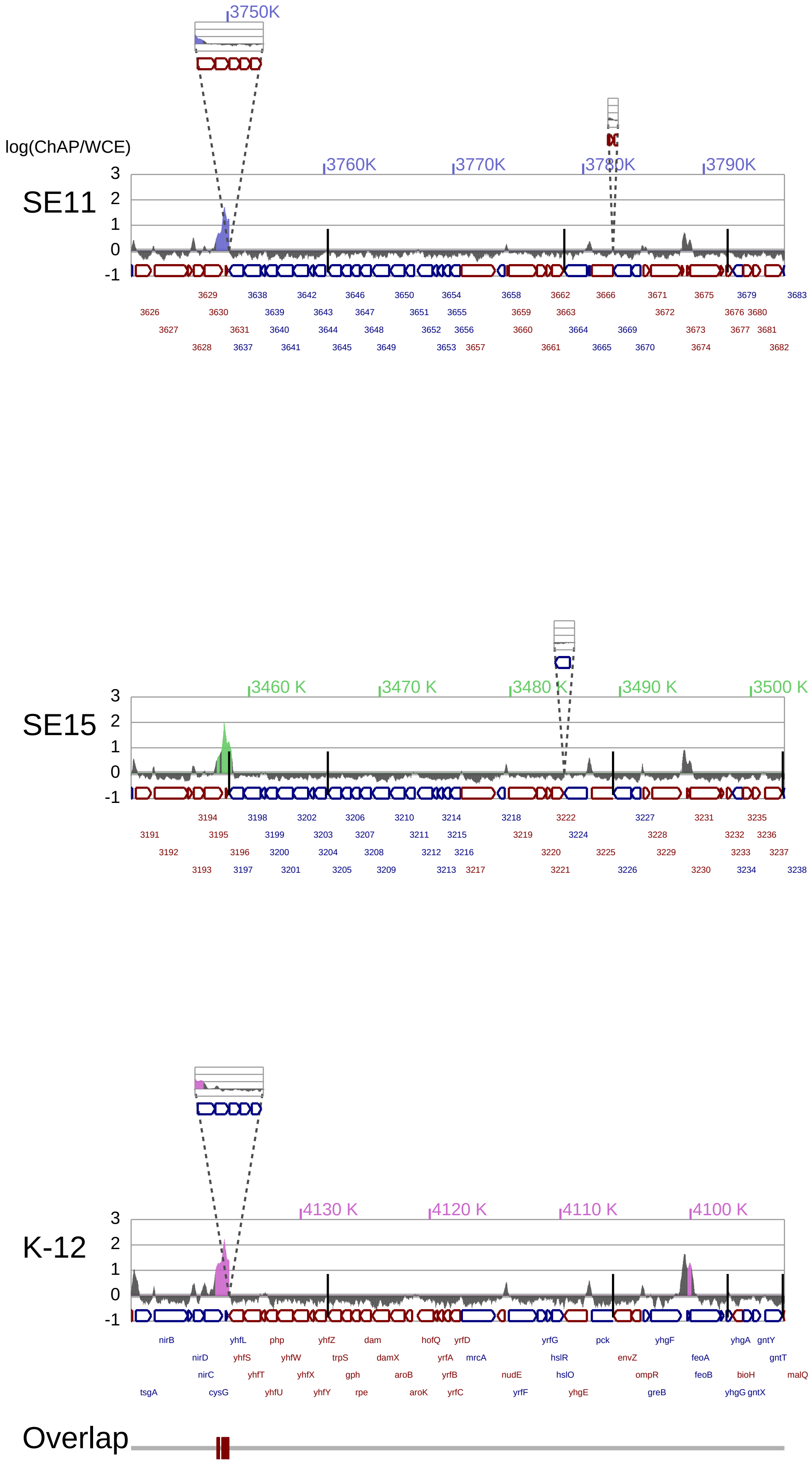

log(ChAP/WCE)
SE11
3750K
3760K
3770K
3780K
3790K
3626 3627 3628 3629 3630 3631 3637 3638 3639 3640 3641 3642 3643 3644 3645 3646 3647 3648 3649 3650 3651 3652 3653 3654 3655 3656 3657 3658 3659 3660 3661 3662 3663 3664 3665 3666 3669 3670 3671 3672 3673 3674 3675 3676 3677 3679 3680 3681 3682 3683
SE15
3460 K
3470 K
3480 K
3490 K
3500 K
3191 3192 3193 3194 3195 3196 3197 3198 3199 3200 3201 3202 3203 3204 3205 3206 3207 3208 3209 3210 3211 3212 3213 3214 3215 3216 3217 3218 3219 3220 3221 3222 3224 3225 3226 3227 3228 3229 3230 3231 3232 3233 3234 3235 3236 3237 3238
K-12
4130 K
4120 K
4110 K
4100 K
tsgA nirB nirD nirC cysG yhfL yhfS yhfT yhfU php yhfW yhfX yhfY yhfZ trpS gph rpe dam damX aroB aroK hofQ yrfA yrfB yrfC yrfD mrcA nudE yrfF yrfG hslR hslO yhgE pck envZ ompR greB yhgF feoA feoB bioH yhgA gntY gntT yhgG gntX malQ
Overlap

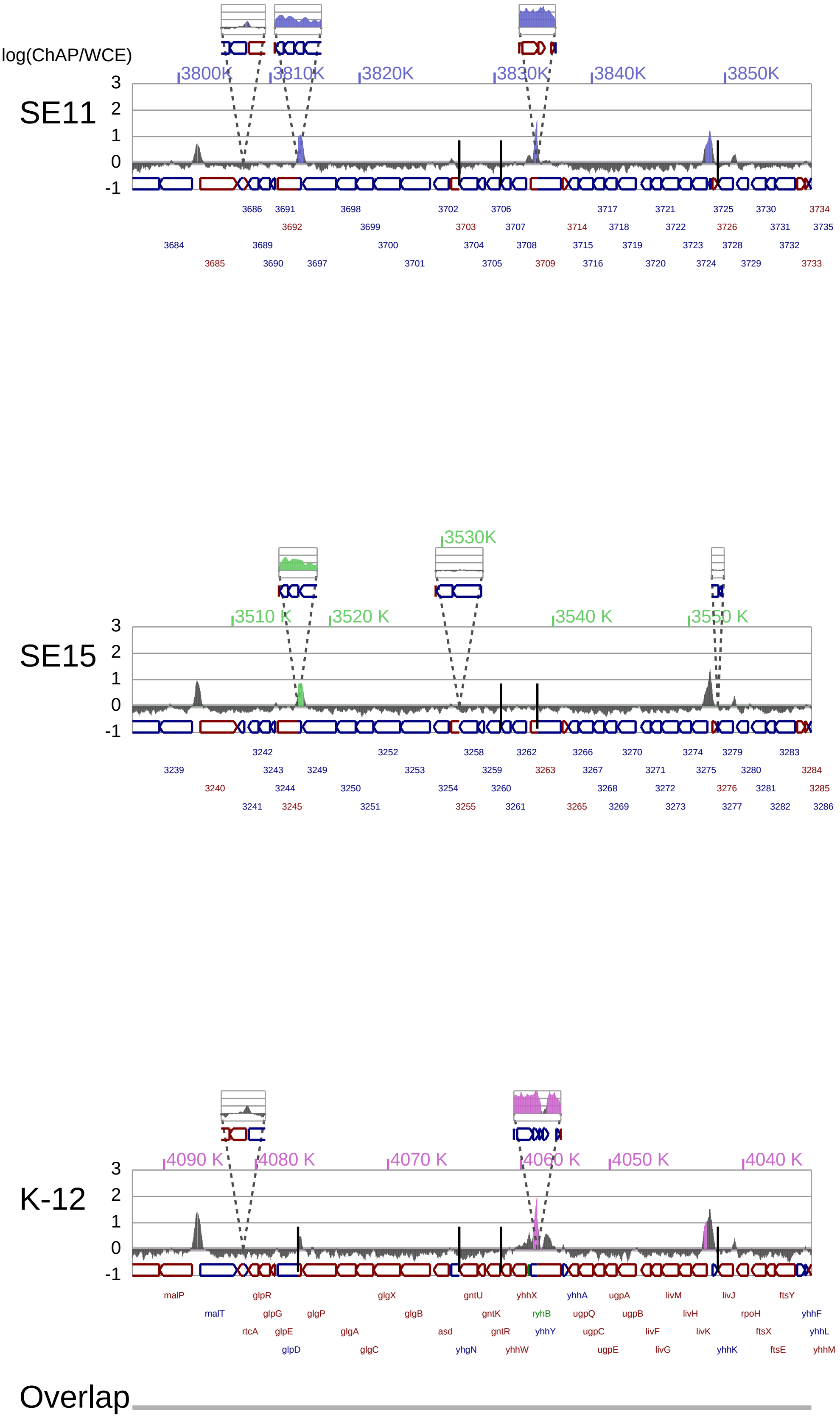
log(ChAP/WCE)
SE11
3800K
3810K
3820K
3830K
3840K
3850K
3684 3685 3686 3689 3690 3691 3692 3697 3698 3699 3700 3701 3702 3703 3704 3705 3706 3707 3708 3709 3714 3715 3716 3717 3718 3719 3720 3721 3722 3723 3724 3725 3726 3728 3729 3730 3731 3732 3733 3734 3735
SE15
3510 K
3520 K
3530K
3540 K
3550 K
3239 3240 3241 3242 3243 3244 3245 3249 3250 3251 3252 3253 3254 3255 3258 3259 3260 3261 3262 3263 3265 3266 3267 3268 3269 3270 3271 3272 3273 3274 3275 3276 3277 3279 3280 3281 3282 3283 3284 3285 3286
K-12
4090 K
4080 K
4070 K
4060 K
4050 K
4040 K
malP malT rtcA glpR glpG glpE glpD glpP glgA glgC glgX glgB asd yhgN gntU gntK gntR yhhW yhhX ryhB yhhY yhhA ugpQ ugpC ugpE ugpA ugpB livF livG livM livH livK livJ yhhK rpoH ftsX ftsE ftsY yhhF yhhL yhhM
Overlap

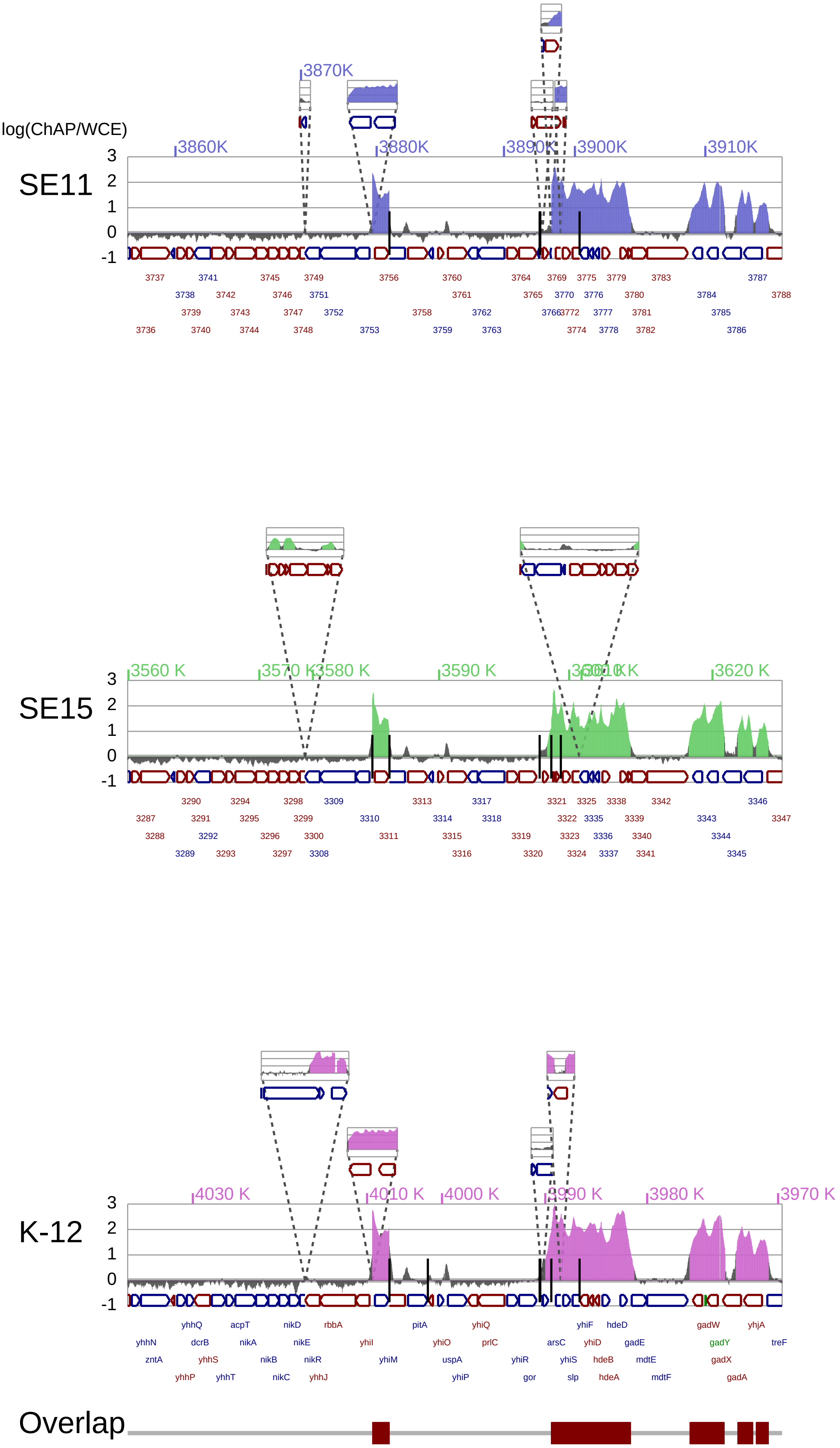

log(ChAP/WCE)
SE11
3860K
3870K
3880K
3890K
3900K
3910K
3 2 1 0 -1
3737 3741 3745 3749 3756 3760 3764 3769 3775 3779 3783 3787
3738 3742 3746 3751 3761 3765 3770 3776 3780 3784 3788
3739 3743 3747 3752 3758 3762 3766 3772 3777 3781 3785
3736 3740 3744 3748 3753 3759 3763 3774 3778 3782 3786
SE15
3560 K
3570 K
3580 K
3590 K
3610 K
3620 K
3 2 1 0 -1
3290 3294 3298 3309 3313 3317 3321 3325 3338 3342 3346
3287 3291 3295 3299 3310 3314 3318 3322 3335 3339 3343 3347
3288 3292 3296 3300 3311 3315 3319 3323 3336 3340 3344
3289 3293 3297 3308 3316 3320 3324 3337 3341 3345
K-12
4030 K
4010 K
4000 K
3990 K
3980 K
3970 K
3 2 1 0 -1
yhhQ acpT nikD rbbA pitA yhiQ yhiF hdeD gadW yhjA
yhhN dcrB nikA nikE yhiI yhiO prlC arsC yhiD gadE gadY treF
zntA yhhS nikB nikR yhiM uspA yhiR yhiS hdeB mdtE gadX
yhhP yhhT nikC yhhJ yhiP gor slp hdeA mdtF gadA
Overlap

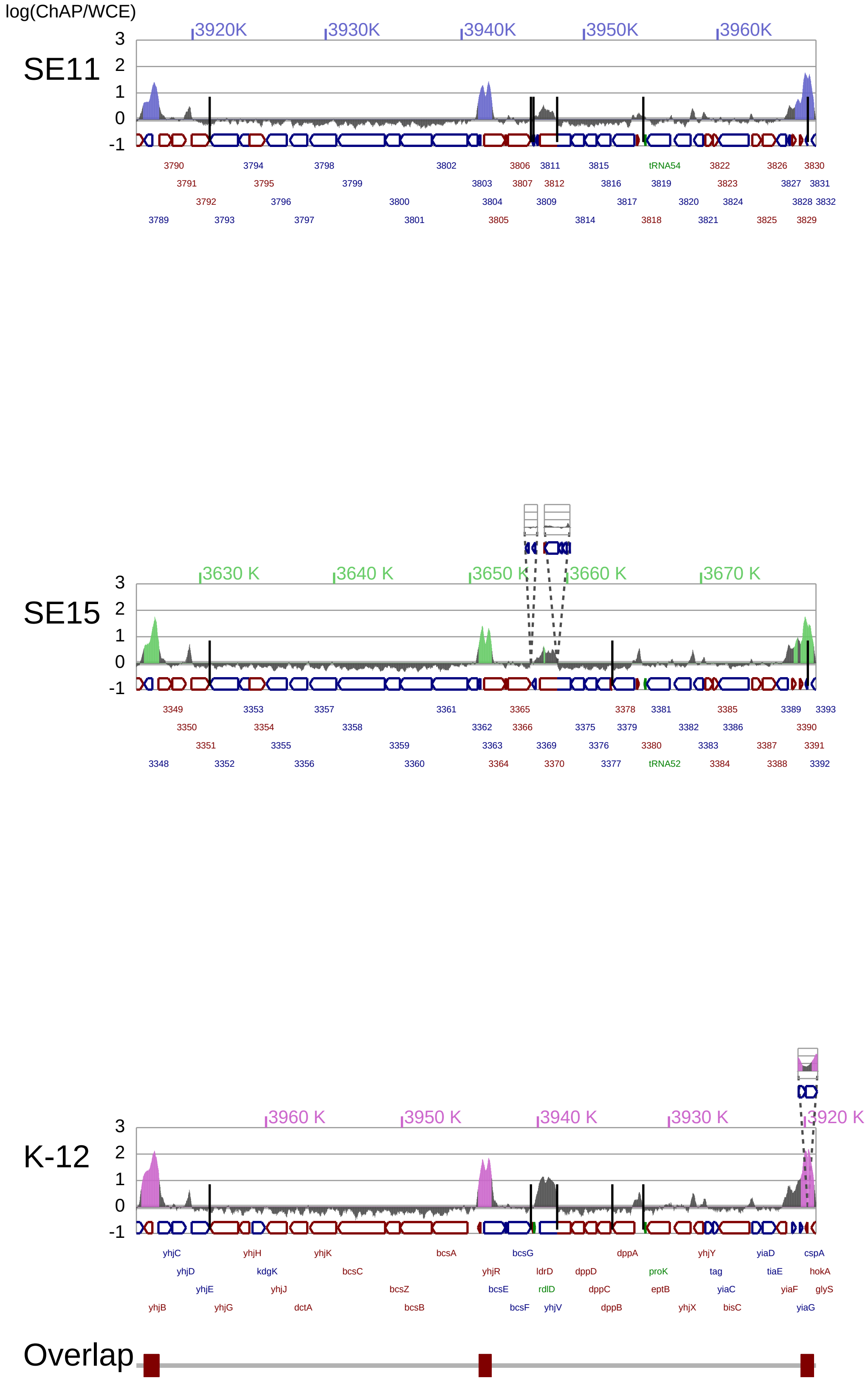

log(ChAP/WCE)
SE11
3
2
1
0
-1
3920K
3930K
3940K
3950K
3960K
3790 3794 3798 3802 3806 3811 3815 tRNA54 3822 3826 3830
3791 3795 3799 3803 3807 3812 3816 3819 3823 3827 3831
3792 3796 3800 3804 3809 3817 3820 3824 3828 3832
3789 3793 3797 3801 3805 3814 3818 3821 3825 3829
SE15
3630 K
3640 K
3650 K
3660 K
3670 K
3349 3353 3357 3361 3365 3378 3381 3385 3389 3393
3350 3354 3358 3362 3366 3375 3379 3382 3386 3390
3351 3355 3359 3363 3369 3376 3380 3383 3387 3391
3348 3352 3356 3360 3364 3370 3377 tRNA52 3384 3388 3392
K-12
3960 K
3950 K
3940 K
3930 K
3920 K
yhjC yhjH yhjK bcsA bcsG dppA yhjY yiaD cspA
yhjD kdgK bcsC yhjR ldrD dppD proK tag tiaE hokA
yhjE yhjJ bcsZ bcsE rdlD dppC eptB yiaC yiaF glyS
yhjB yhjG dctA bcsB bcsF yhjV dppB yhjX bisC yiaG
Overlap

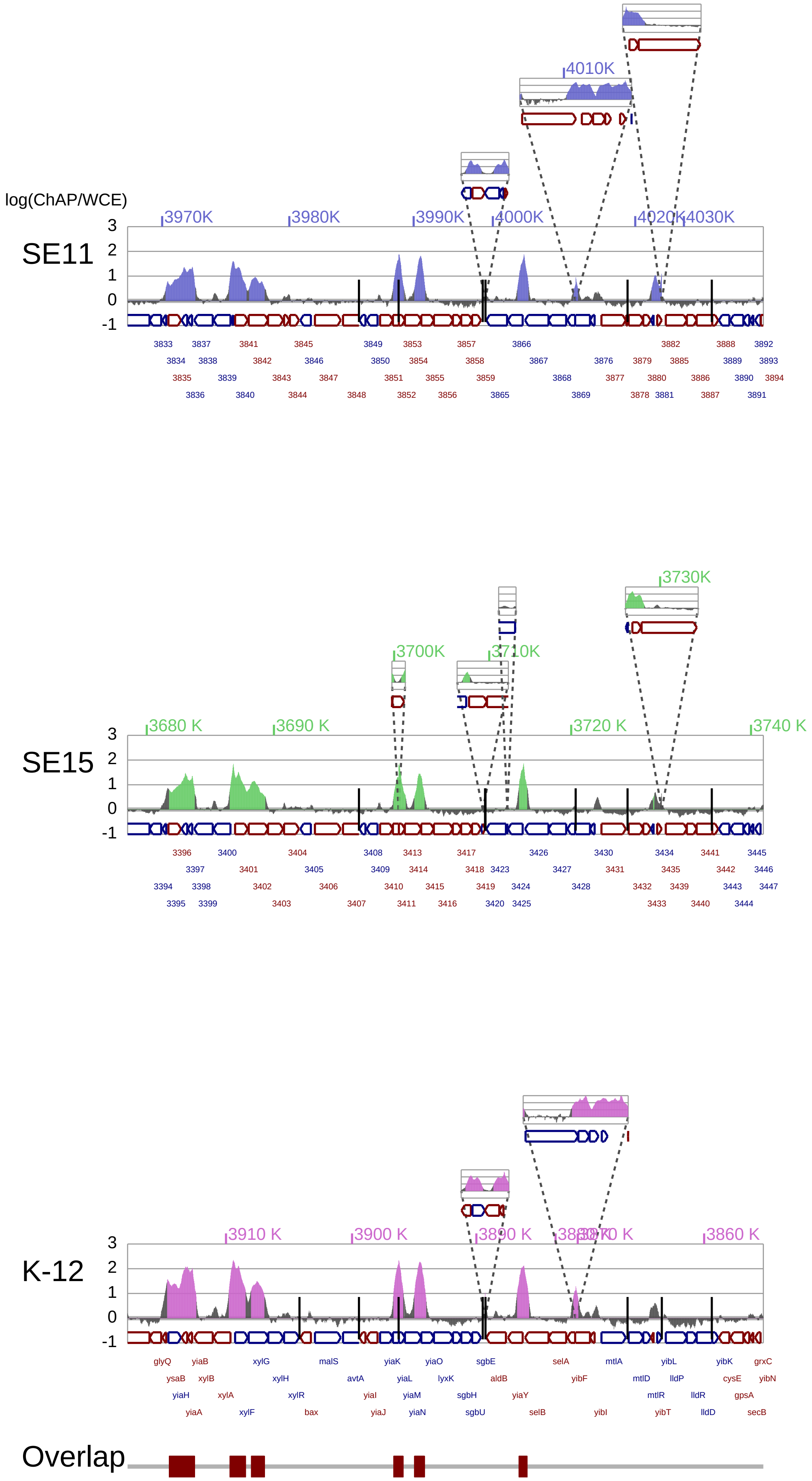

log(ChAP/WCE)
SE11
3970K
3980K
3990K
4000K
4010K
4020K
4030K
3
2
1
0
-1
3833 3837 3841 3845 3849 3853 3857 3866 3882 3888 3892
3834 3838 3842 3846 3850 3854 3858 3867 3876 3879 3885 3889 3893
3835 3839 3843 3847 3851 3855 3859 3868 3877 3880 3886 3890 3894
3836 3840 3844 3848 3852 3856 3865 3869 3878 3881 3887 3891
SE15
3680 K
3690 K
3700K
3710K
3720 K
3730K
3740 K
3
2
1
0
-1
3396 3400 3404 3408 3413 3417 3426 3430 3434 3441 3445
3397 3401 3405 3409 3414 3418 3423 3427 3431 3435 3442 3446
3394 3398 3402 3406 3410 3415 3419 3424 3428 3432 3439 3443 3447
3395 3399 3403 3407 3411 3416 3420 3425 3433 3440 3444
K-12
3910 K
3900 K
3890 K
3880 K
3870 K
3860 K
3
2
1
0
-1
glyQ yiaB xylG malS yiaK yiaO sgbE selA mtlA yibL yibK grxC
ysaB xylB xylH avtA yiaL lyxK aldB yibF mtlD lldP cysE yibN
yiaH xylA xylR yiaI yiaM sgbH yiaY mtlR lldR gpsA
yiaA xylF bax yiaJ yiaN sgbU selB yibI yibT lldD secB
Overlap

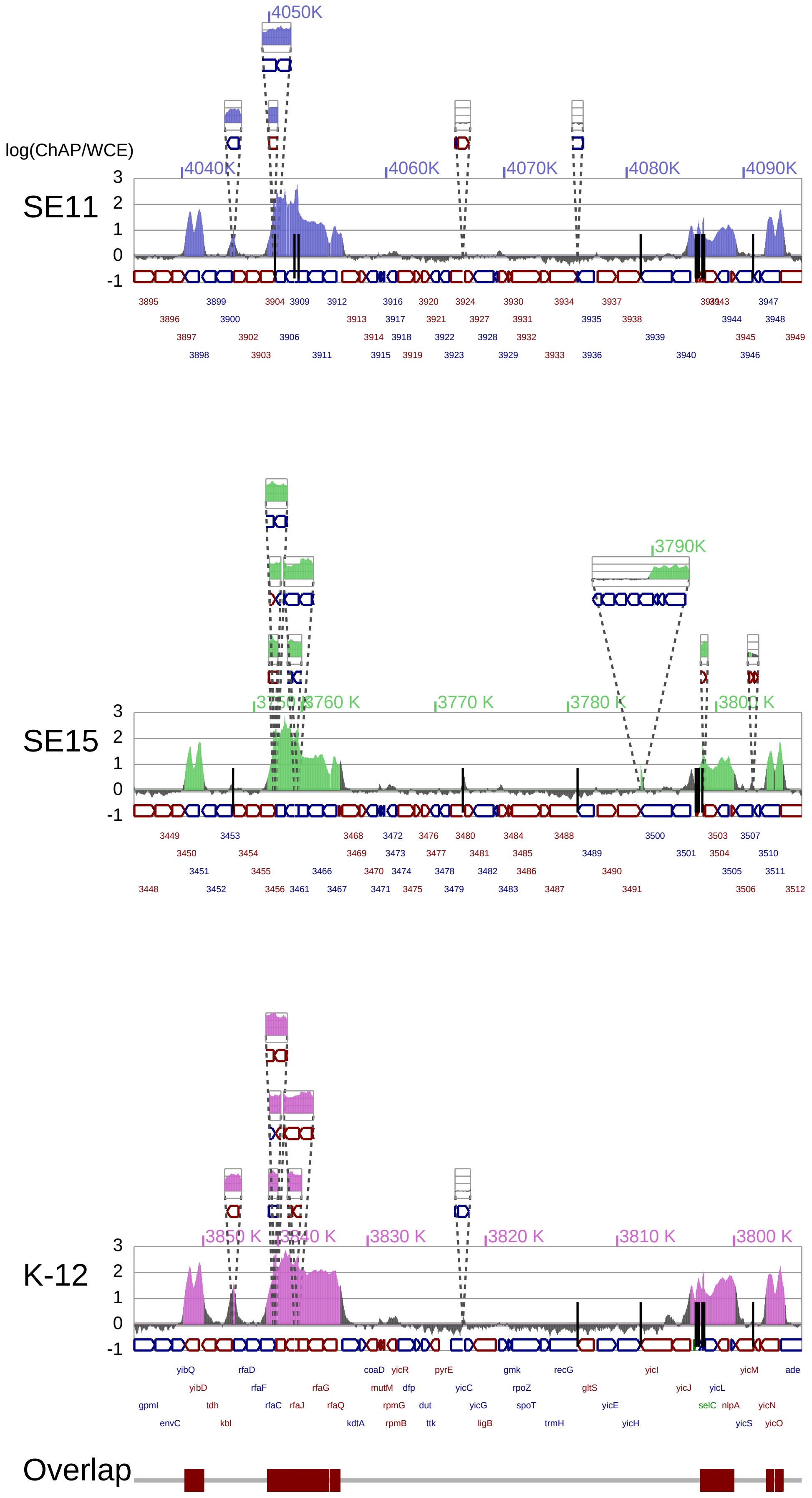

log(ChAP/WCE)
SE11
3
2
1
0
-1
4040K
4050K
4060K
4070K
4080K
4090K
3895 3896 3897 3898 3899 3900 3902 3903 3904 3906 3909 3911 3912 3913 3914 3915 3916 3917 3918 3919 3920 3921 3922 3923 3924 3927 3928 3929 3930 3931 3932 3933 3934 3935 3936 3937 3938 3939 3940 3944 3945 3946 3947 3948 3949
SE15
3750
3760 K
3770 K
3780 K
3790K
3800 K
3448 3449 3450 3451 3452 3453 3454 3455 3456 3461 3466 3467 3468 3469 3470 3471 3472 3473 3474 3475 3476 3477 3478 3479 3480 3481 3482 3483 3484 3485 3486 3487 3488 3489 3490 3491 3500 3501 3503 3504 3505 3506 3507 3510 3511 3512
K-12
3850 K
3840 K
3830 K
3820 K
3810 K
3800 K
yibQ yibD gpmI envC tdh kbl rfaD rfaF rfaC rfaJ rfaG rfaQ kdtA coaD yicR mutM dfp rpmG rpmB dut ttk pyrE yicC yicG ligB gmk rpoZ spoT trmH recG gltS yicE yicH yicI yicJ yicL selC nlpA yicM yicN yicS yicO ade
Overlap

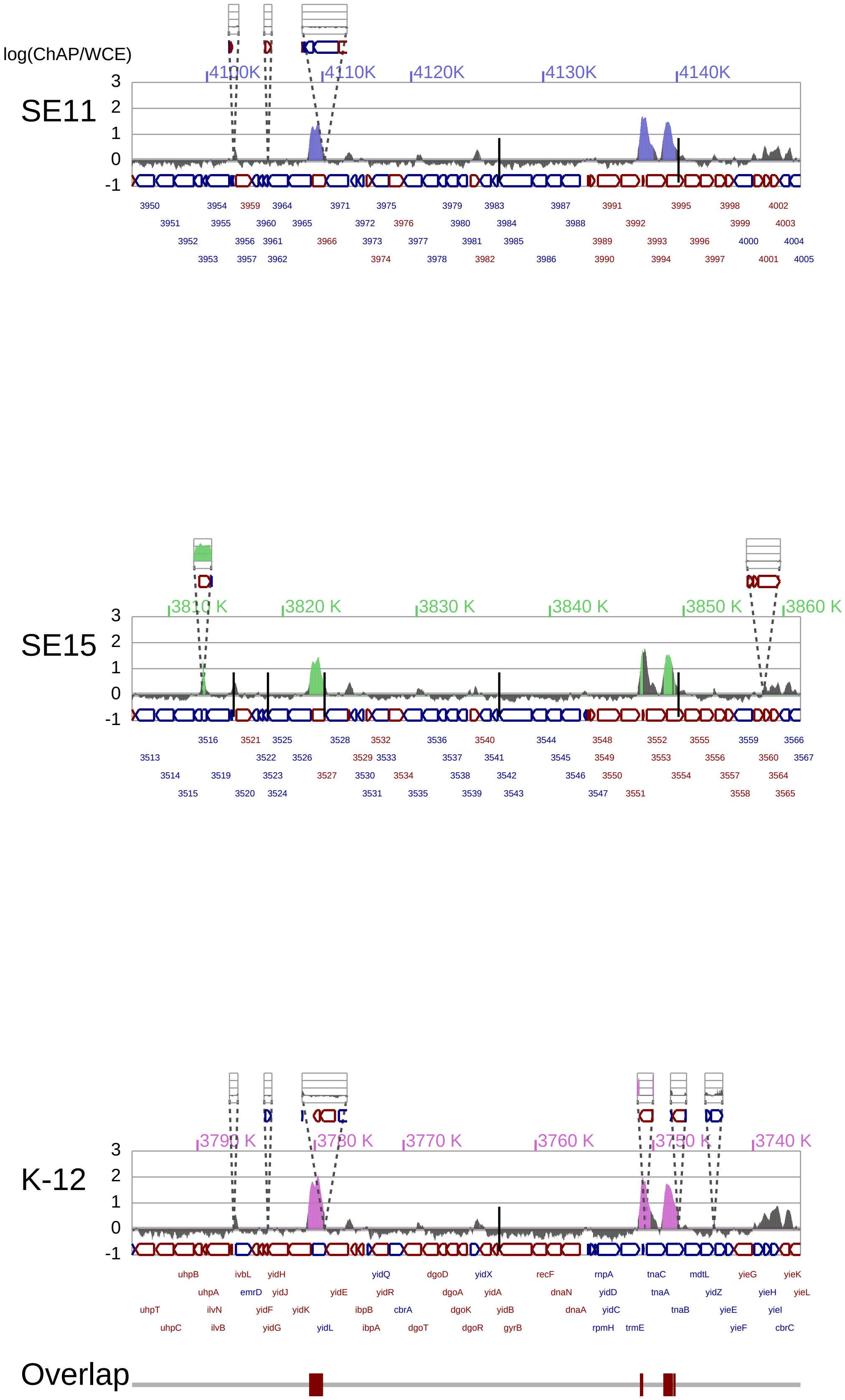

log(ChAP/WCE)
SE11
4100K
4110K
4120K
4130K
4140K
3950 3951 3952 3953 3954 3955 3956 3957 3959 3960 3961 3962 3964 3965 3966 3971 3972 3973 3974 3975 3976 3977 3978 3979 3980 3981 3982 3983 3984 3985 3986 3987 3988 3989 3990 3991 3992 3993 3994 3995 3996 3997 3998 3999 4000 4001 4002 4003 4004 4005
SE15
3810 K
3820 K
3830 K
3840 K
3850 K
3860 K
3513 3514 3515 3516 3519 3520 3521 3522 3523 3524 3525 3526 3527 3528 3529 3530 3531 3532 3533 3534 3535 3536 3537 3538 3539 3540 3541 3542 3543 3544 3545 3546 3547 3548 3549 3550 3551 3552 3553 3554 3555 3556 3557 3558 3559 3560 3564 3565 3566 3567
K-12
3790 K
3780 K
3770 K
3760 K
3750 K
3740 K
uhpT uhpC uhpB uhpA ivbL ilvN ilvB emrD yidH yidJ yidF yidG yidK yidE yidL yidQ yidR ibpB ibpA cbrA dgoT dgoD dgoA dgoK dgoR yidX yidA yidB gyrB recF dnaN dnaA rnpA yidD yidC rpmH trmE tnaC tnaA tnaB mdtL yidZ yieE yieF yieG yieH yieI cbrC yieK yieL
Overlap

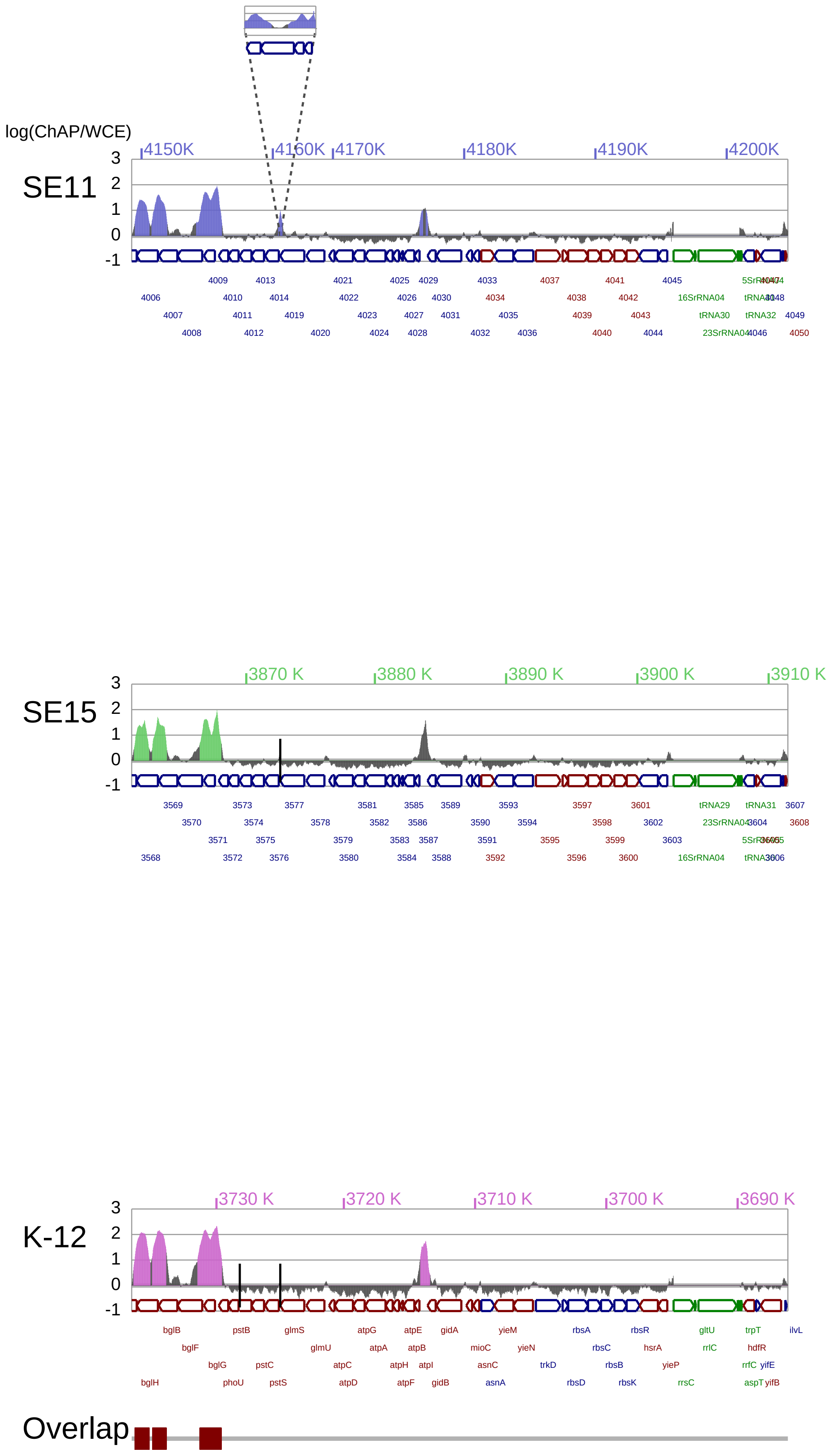

log(ChAP/WCE)
SE11
4150K
4160K
4170K
4180K
4190K
4200K
3
2
1
0
-1
4009 4013 4021 4025 4029 4033 4037 4041 4045
4006 4010 4014 4022 4026 4030 4034 4038 4042 16SrRNA04 4048
4007 4011 4019 4023 4027 4031 4035 4039 4043 tRNA30 tRNA32 4049
4008 4012 4020 4024 4028 4032 4036 4040 4044 23SrRNA04 4046 4050
SE15
3870 K
3880 K
3890 K
3900 K
3910 K
3
2
1
0
-1
3569 3573 3577 3581 3585 3589 3593 3597 3601 tRNA29 tRNA31 3607
3570 3574 3578 3582 3586 3590 3594 3598 3602 23SrRNA04 3604 3608
3571 3575 3579 3583 3587 3591 3595 3599 3603
3568 3572 3576 3580 3584 3588 3592 3596 3600 16SrRNA04 3606
K-12
3730 K
3720 K
3710 K
3700 K
3690 K
3
2
1
0
-1
bglB pstB glmS atpG atpE gidA yieM rbsA rbsR gltU trpT ilvL
bglF glmU atpA atpB mioC yieN rbsC hsrA rrlC hdfR
bglG pstC atpC atpH atpI asnC trkD rbsB yieP rrfC yifE
bglH phoU pstS atpD atpF gidB asnA rbsD rbsK rrsC aspT yifB
Overlap

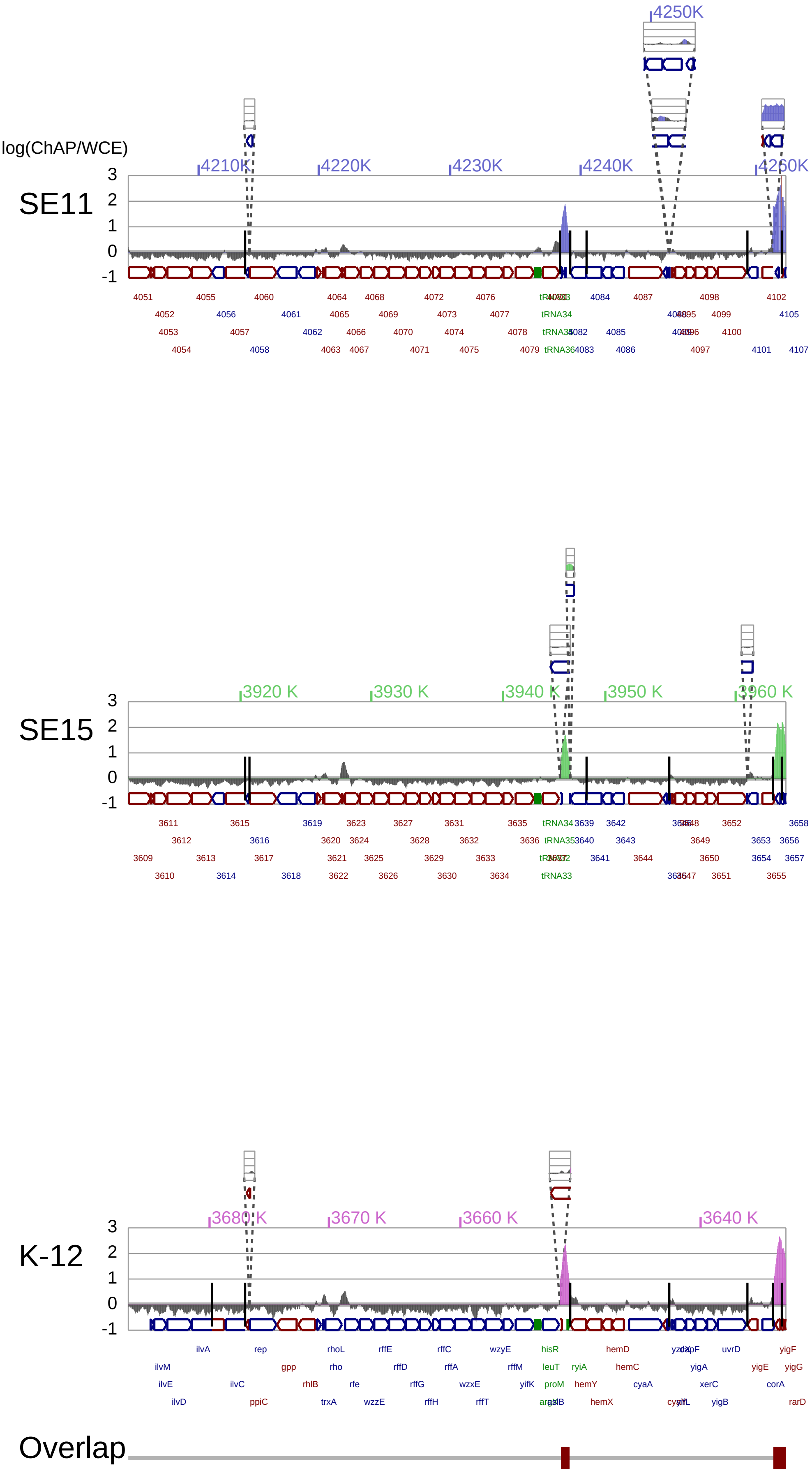

log(ChAP/WCE)
SE11
SE15
K-12
Overlap
3
2
1
0
-1
4210K
4220K
4230K
4240K
4250K
4260K
3920 K
3930 K
3940 K
3950 K
3960 K
3680 K
3670 K
3660 K
3640 K
4051 4052 4053 4054 4055 4056 4057 4058 4060 4061 4062 4063 4064 4065 4066 4067 4068 4069 4070 4071 4072 4073 4074 4075 4076 4077 4078 4079 4082 4083 4084 4085 4086 4087 4097 4098 4099 4100 4101 4102 4105 4107
tRNA34
3609 3610 3611 3612 3613 3614 3615 3616 3617 3618 3619 3620 3621 3622 3623 3624 3625 3626 3627 3628 3629 3630 3631 3632 3633 3634 3635 3636 3639 3640 3641 3642 3643 3644 3649 3650 3651 3652 3653 3654 3655 3656 3657 3658
tRNA34 tRNA35 tRNA33
ilvA ilvM ilvE ilvD rep ilvC gpp ppiC rhoL rho rhlB trxA rffE rfe wzzE rffD rffG rffH rffC rffA wzxE rffT wzyE rffM yifK hisR leuT proM ryiA hemY hemX hemD hemC cyaA yigA xerC yigB uvrD yigE corA yigF yigG rarD

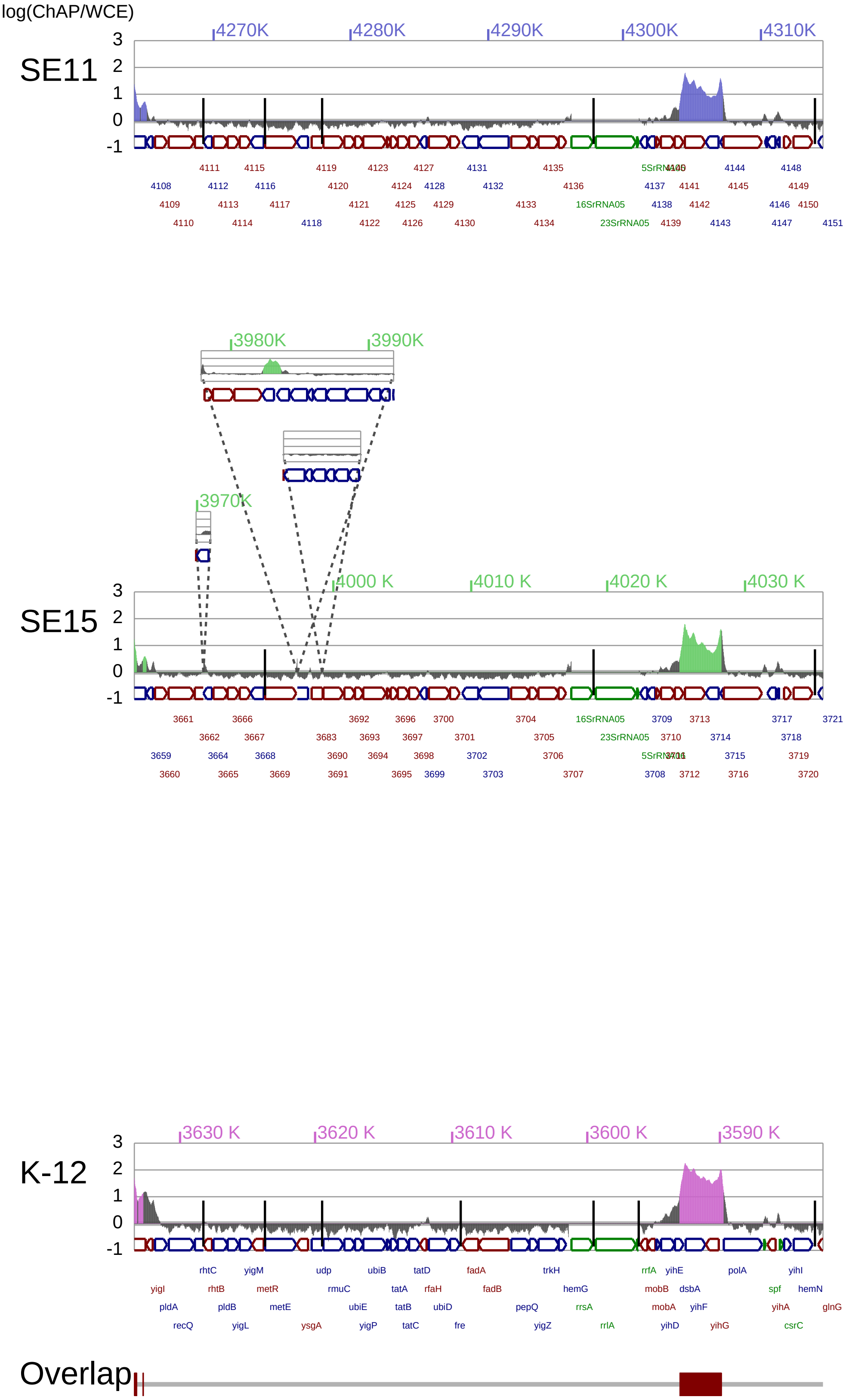

log(ChAP/WCE)
SE11
4270K
4280K
4290K
4300K
4310K
3
2
1
0
-1
4111 4115 4119 4123 4127 4131 4135 5SrRNA05 4140 4144 4148
4108 4112 4116 4120 4124 4128 4132 4136 4137 4141 4145 4149
4109 4113 4117 4121 4125 4129 4133 16SrRNA05 4138 4142 4146 4150
4110 4114 4118 4122 4126 4130 4134 23SrRNA05 4139 4143 4147 4151
3980K
3990K
3970K
SE15
4000 K
4010 K
4020 K
4030 K
3
2
1
0
-1
3661 3666 3692 3696 3700 3704 16SrRNA05 3709 3713 3717 3721
3662 3667 3683 3693 3697 3701 3705 23SrRNA05 3710 3714 3718
3659 3664 3668 3690 3694 3698 3702 3706 5SrRNA05 3711 3715 3719
3660 3665 3669 3691 3695 3699 3703 3707 3708 3712 3716 3720
K-12
3630 K
3620 K
3610 K
3600 K
3590 K
3
2
1
0
-1
rhtC yigM udp ubiB tatD fadA trkH rrfA yihE polA yihI
yigI rhtB metR rmuC tatA rfaH fadB hemG mobB dsbA spf hemN
pldA pldB metE ubiE tatB ubiD pepQ rrsA mobA yihF yihA glnG
recQ yigL ysgA yigP tatC fre yigZ rrlA yihD yihG csrC
Overlap

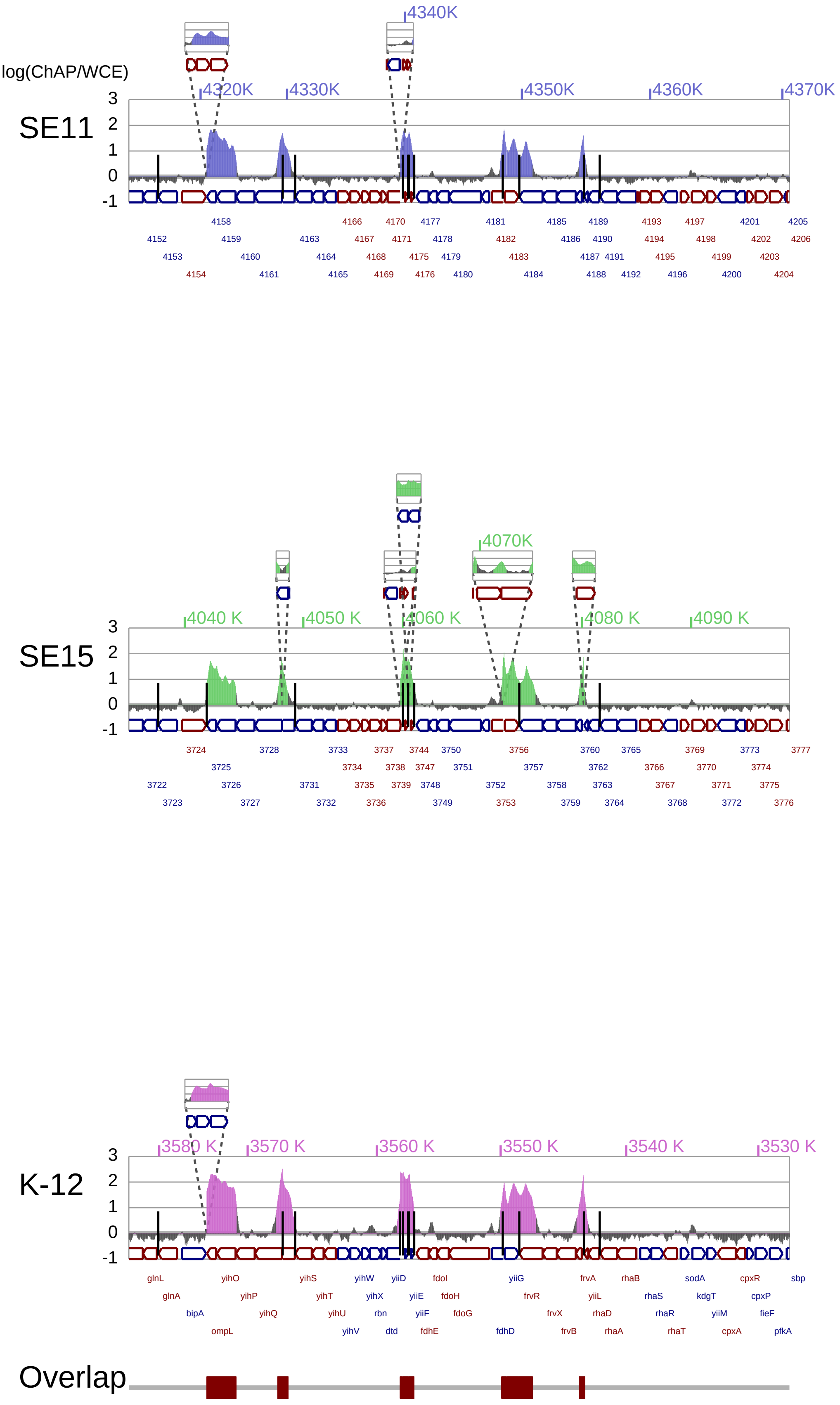

log(ChAP/WCE)
SE11
4320K
4330K
4340K
4350K
4360K
4370K
3
2
1
0
-1
4158 4166 4170 4177 4181 4185 4189 4193 4197 4201 4205
4152 4159 4163 4167 4171 4178 4182 4186 4190 4194 4198 4202 4206
4153 4160 4164 4168 4175 4179 4183 4187 4191 4195 4199 4203
4154 4161 4165 4169 4176 4180 4184 4188 4192 4196 4200 4204
SE15
4040 K
4050 K
4060 K
4070K
4080 K
4090 K
3724 3728 3733 3737 3744 3750 3756 3760 3765 3769 3773 3777
3725 3734 3738 3747 3751 3757 3762 3766 3770 3774
3722 3726 3731 3735 3739 3748 3752 3758 3763 3767 3771 3775
3723 3727 3732 3736 3749 3753 3759 3764 3768 3772 3776
K-12
3580 K
3570 K
3560 K
3550 K
3540 K
3530 K
glnL yihO yihS yihW yiiD fdoI yiiG frvA rhaB sodA cpxR sbp
glnA yihP yihT yihX yiiE fdoH frvR yiiL rhaS kdgT cpxP
bipA yihQ yihU rbn yiiF fdoG frvX rhaD rhaR yiiM fieF
ompL yihV dtd fdhE fdhD frvB rhaA rhaT cpxA pfkA
Overlap

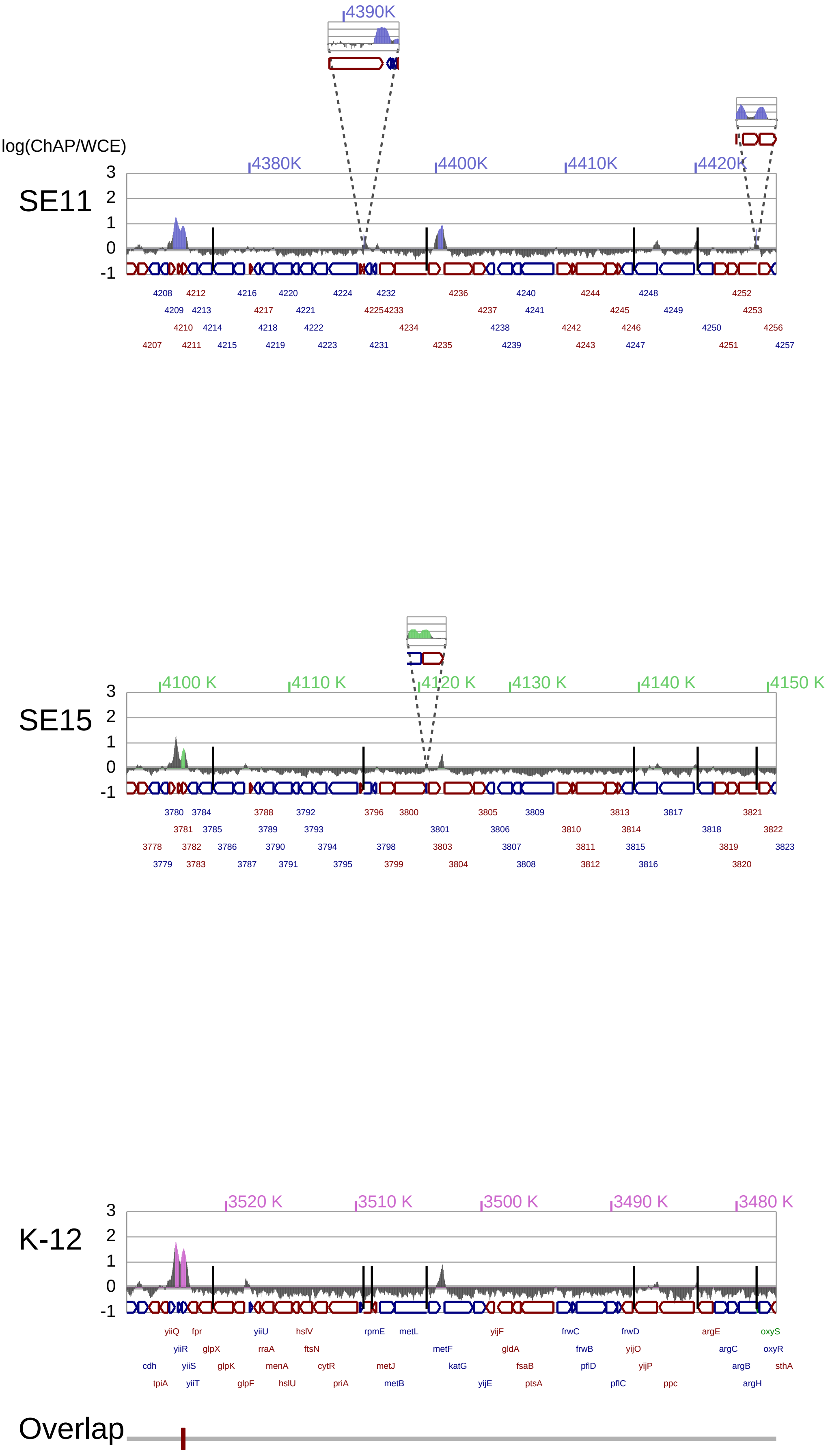
log(ChAP/WCE)
SE11
3
2
1
0
-1
4380K
4390K
4400K
4410K
4420K
4208 4212 4216 4220 4224 4232 4236 4240 4244 4248 4252
4209 4213 4217 4221 4225 4233 4237 4241 4245 4249 4253
4210 4214 4218 4222 4234 4238 4242 4246 4250 4256
4207 4211 4215 4219 4223 4231 4235 4239 4243 4247 4251 4257
SE15
4100 K
4110 K
4120 K
4130 K
4140 K
4150 K
3780 3784 3788 3792 3796 3800 3805 3809 3813 3817 3821
3781 3785 3789 3793 3801 3806 3810 3814 3818 3822
3778 3782 3786 3790 3794 3798 3803 3807 3811 3815 3819 3823
3779 3783 3787 3791 3795 3799 3804 3808 3812 3816 3820
K-12
3520 K
3510 K
3500 K
3490 K
3480 K
yiiQ fpr yiiU hslV rpmE metL yijF frwC frwD argE oxyS
yiiR glpX rraA ftsN metF gldA frwB yijO argC oxyR
cdh yiiS glpK menA cytR metJ katG fsaB pflD yijP argB sthA
tpiA yiiT glpF hslU priA metB yijE ptsA pflC ppc argH
Overlap

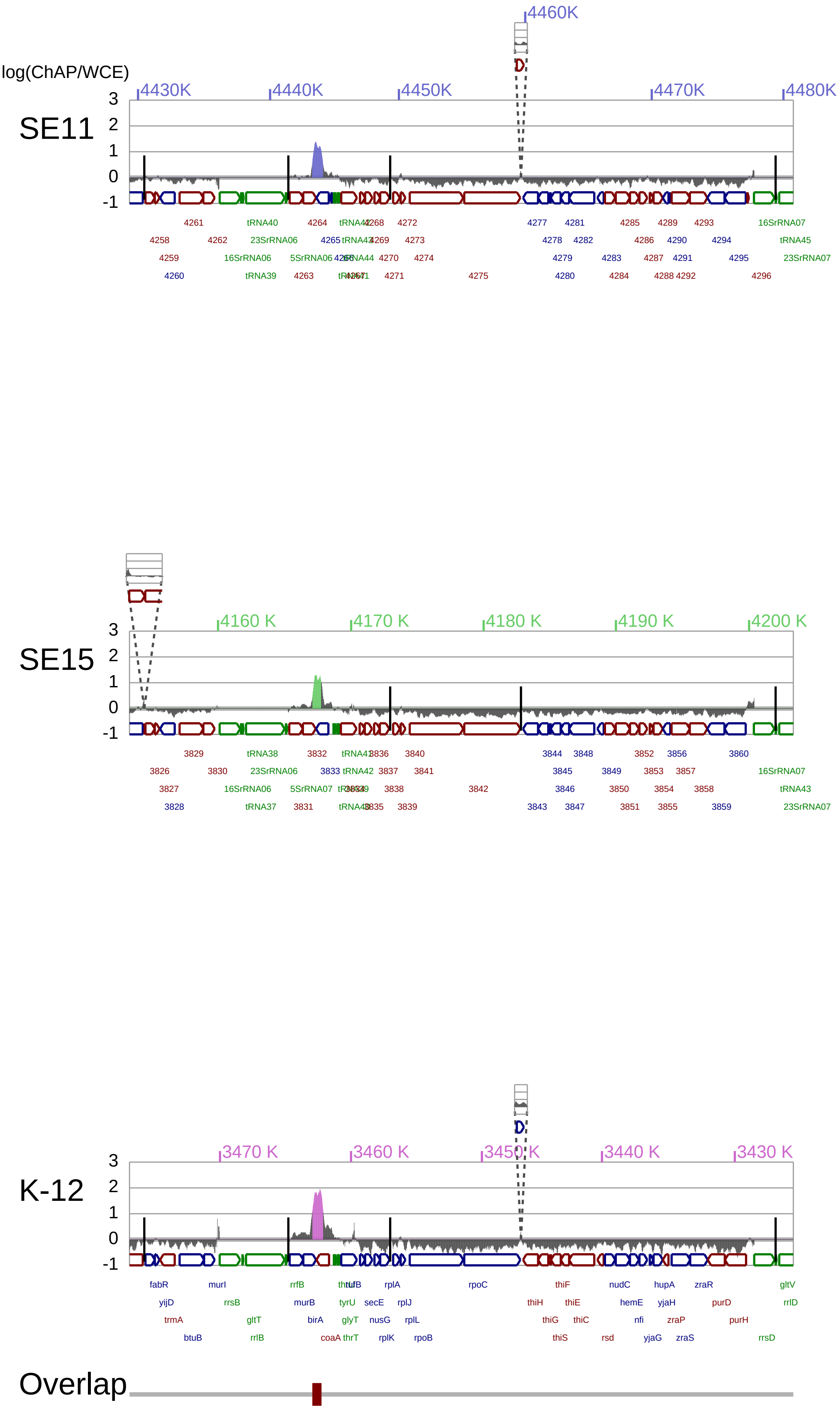

log(ChAP/WCE)
SE11
3
2
1
0
-1
4430K
4440K
4450K
4460K
4470K
4480K
4261
tRNA40
4264
4272
4277
4281
4285
4289
4293
16SrRNA07
4258
4262
23SrRNA06
4265
4269
4273
4278
4282
4286
4290
4294
tRNA45
4259
16SrRNA06
5SrRNA06
tRNA44
4270
4274
4279
4283
4287
4291
4295
23SrRNA07
4260
tRNA39
4263
4271
4275
4280
4284
4288
4292
4296
SE15
4160 K
4170 K
4180 K
4190 K
4200 K
3829
tRNA38
3832
3836
3840
3844
3848
3852
3856
3860
3826
3830
23SrRNA06
3833
tRNA42
3837
3841
3845
3849
3853
3857
16SrRNA07
3827
16SrRNA06
5SrRNA07
3838
3842
3846
3850
3854
3858
tRNA43
3828
tRNA37
3831
3835
3839
3843
3847
3851
3855
3859
23SrRNA07
K-12
3470 K
3460 K
3450 K
3440 K
3430 K
fabR
murI
rrfB
tufB
rplA
rpoC
thiF
nudC
hupA
zraR
gltV
yijD
rrsB
murB
tyrU
secE
rplJ
thiH
thiE
hemE
yjaH
purD
rrlD
trmA
gltT
birA
glyT
nusG
rplL
thiG
thiC
nfi
zraP
purH
btuB
rrlB
coaA
thrT
rplK
rpoB
thiS
rsd
yjaG
zraS
rrsD
Overlap

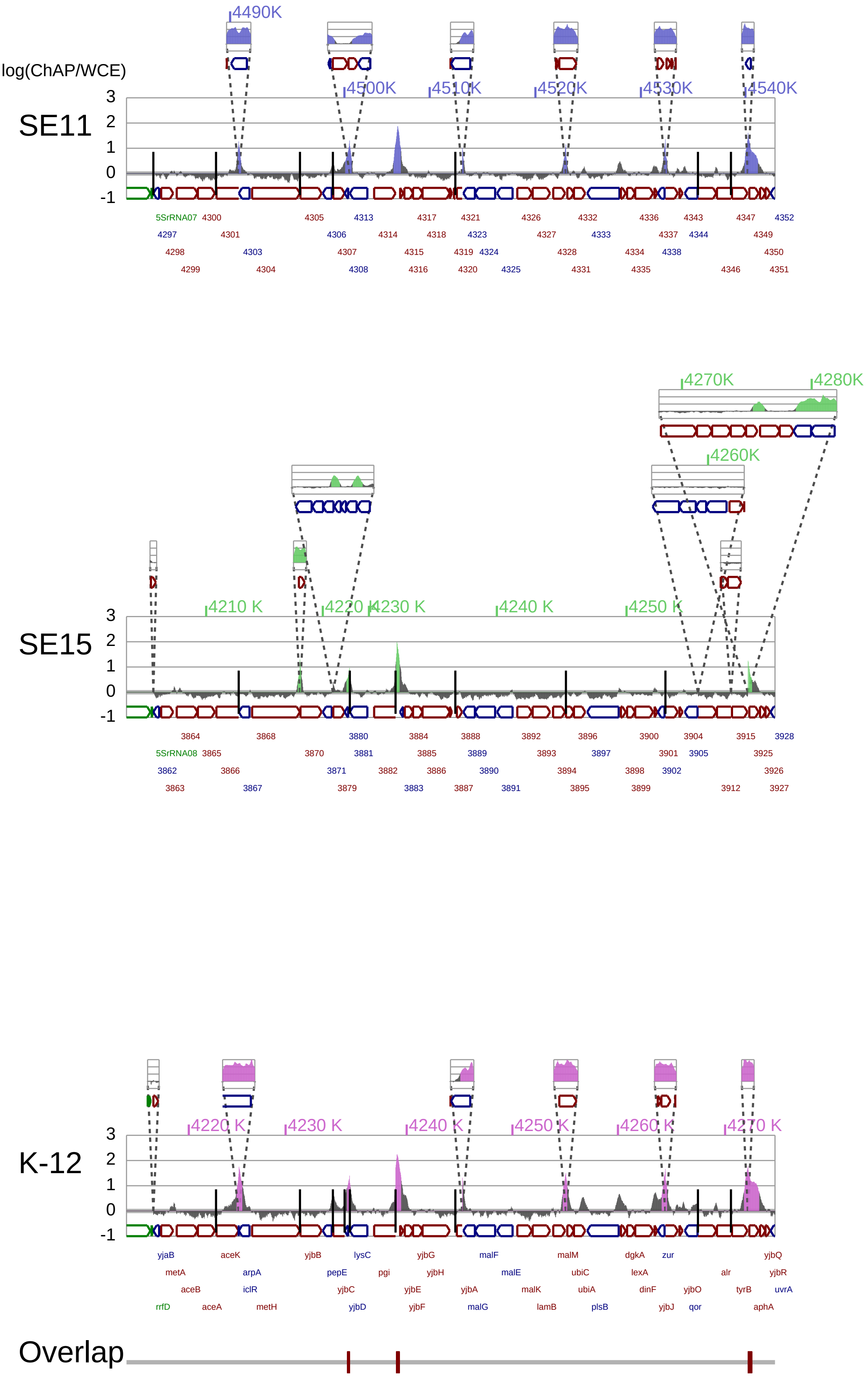

log(ChAP/WCE)
SE11
4490K
4500K
4510K
4520K
4530K
4540K
3
2
1
0
-1
5SrRNA07 4300 4305 4313 4317 4321 4326 4332 4336 4343 4347 4352
4297 4301 4306 4314 4318 4323 4327 4333 4337 4344 4349
4298 4303 4307 4315 4319 4324 4328 4334 4338 4350
4299 4304 4308 4316 4320 4325 4331 4335 4346 4351
SE15
4270K
4280K
4260K
4210 K
4220 K
4230 K
4240 K
4250 K
3864 3868 3880 3884 3888 3892 3896 3900 3904 3915 3928
5SrRNA08 3865 3870 3881 3885 3889 3893 3897 3901 3905 3925
3862 3866 3871 3882 3886 3890 3894 3898 3902 3926
3863 3867 3879 3883 3887 3891 3895 3899 3912 3927
K-12
4220 K
4230 K
4240 K
4250 K
4260 K
4270 K
yjaB aceK yjbB lysC yjbG malF malM dgkA zur yjbQ
metA arpA pepE pgi yjbH malE ubiC lexA alr yjbR
aceB iclR yjbC yjbE yjbA malK ubiA dinF yjbO tyrB uvrA
rrfD aceA metH yjbD yjbF malG lamB plsB yjbJ qor aphA
Overlap

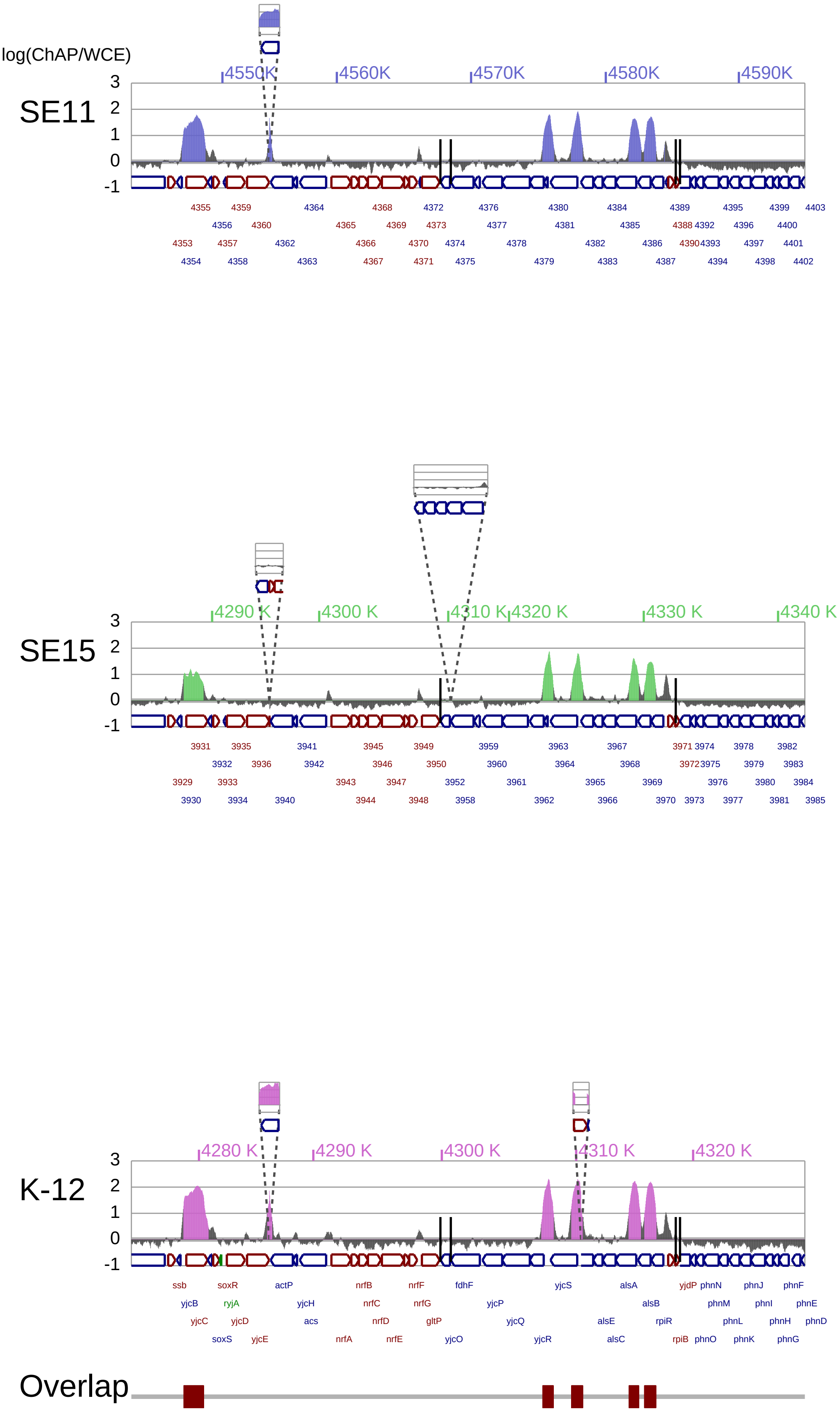
log(ChAP/WCE)
SE11
4550K
4560K
4570K
4580K
4590K
3
2
1
0
-1
4355 4359 4364 4368 4372 4376 4380 4384 4389 4395 4399 4403
4356 4360 4365 4369 4373 4377 4381 4385 4388 4392 4396 4400
4353 4357 4362 4366 4370 4374 4378 4382 4386 4390 4393 4397 4401
4354 4358 4363 4367 4371 4375 4379 4383 4387 4394 4398 4402
SE15
4290 K
4300 K
4310 K
4320 K
4330 K
4340 K
3
2
1
0
-1
3931 3935 3941 3945 3949 3959 3963 3967 3971 3974 3978 3982
3932 3936 3942 3946 3950 3960 3964 3968 3972 3975 3979 3983
3929 3933 3943 3947 3952 3961 3965 3969 3976 3980 3984
3930 3934 3940 3944 3948 3958 3962 3966 3970 3973 3977 3981 3985
K-12
4280 K
4290 K
4300 K
4310 K
4320 K
3
2
1
0
-1
ssb soxR actP nrfB nrfF fdhF yjcS alsA yjdP phnN phnJ phnF
yjcB ryjA yjcH nrfC nrfG yjcP alsB phnM phnI phnE
yjcC yjcD acs nrfD gltP yjcQ alsE rpiR phnL phnH phnD
soxS yjcE nrfA nrfE yjcO yjcR alsC rpiB phnO phnK phnG
Overlap

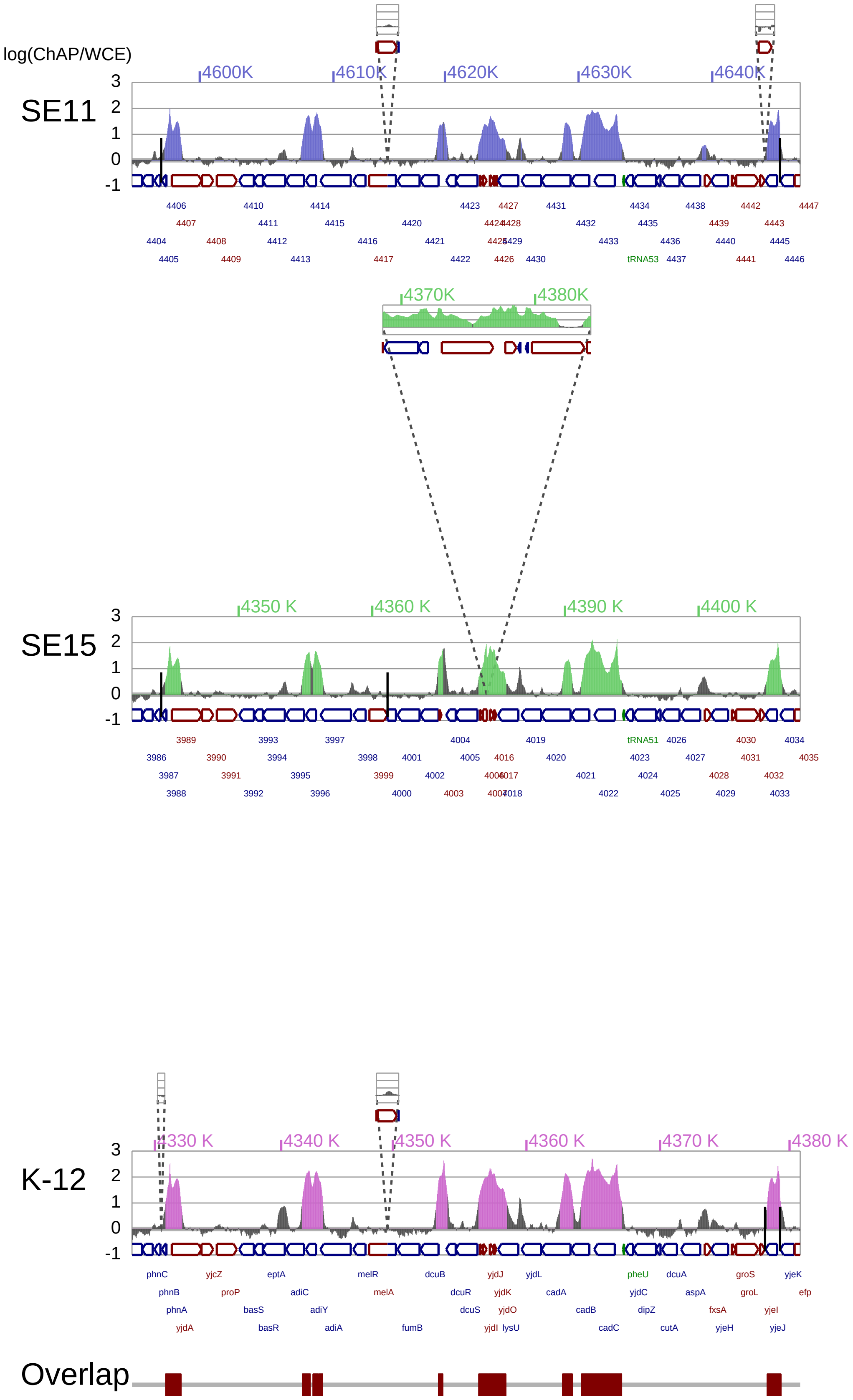
log(ChAP/WCE)
SE11
4600K
4610K
4620K
4630K
4640K
3
2
1
0
-1
4406 4410 4414 4423 4427 4431 4434 4438 4442 4447
4407 4411 4415 4420 4424 4428 4432 4435 4439 4443
4404 4408 4412 4416 4421 4425 4429 4433 4436 4440 4445
4405 4409 4413 4417 4422 4426 4430 tRNA53 4437 4441 4446
4370K
4380K
SE15
4350 K
4360 K
4390 K
4400 K
3989 3993 3997 4004 4019 tRNA51 4026 4030 4034
3986 3990 3994 3998 4001 4005 4016 4020 4023 4027 4031 4035
3987 3991 3995 3999 4002 4006 4017 4021 4024 4028 4032
3988 3992 3996 4000 4003 4007 4018 4022 4025 4029 4033
K-12
4330 K
4340 K
4350 K
4360 K
4370 K
4380 K
phnC yjcZ eptA melR dcuB yjdJ yjdL pheU dcuA groS yjeK
phnB proP adiC melA dcuR yjdK cadA yjdC aspA groL efp
phnA basS adiY dcuS yjdO cadB dipZ fxsA yjeI
yjdA basR adiA fumB yjdI lysU cadC cutA yjeH yjeJ
Overlap

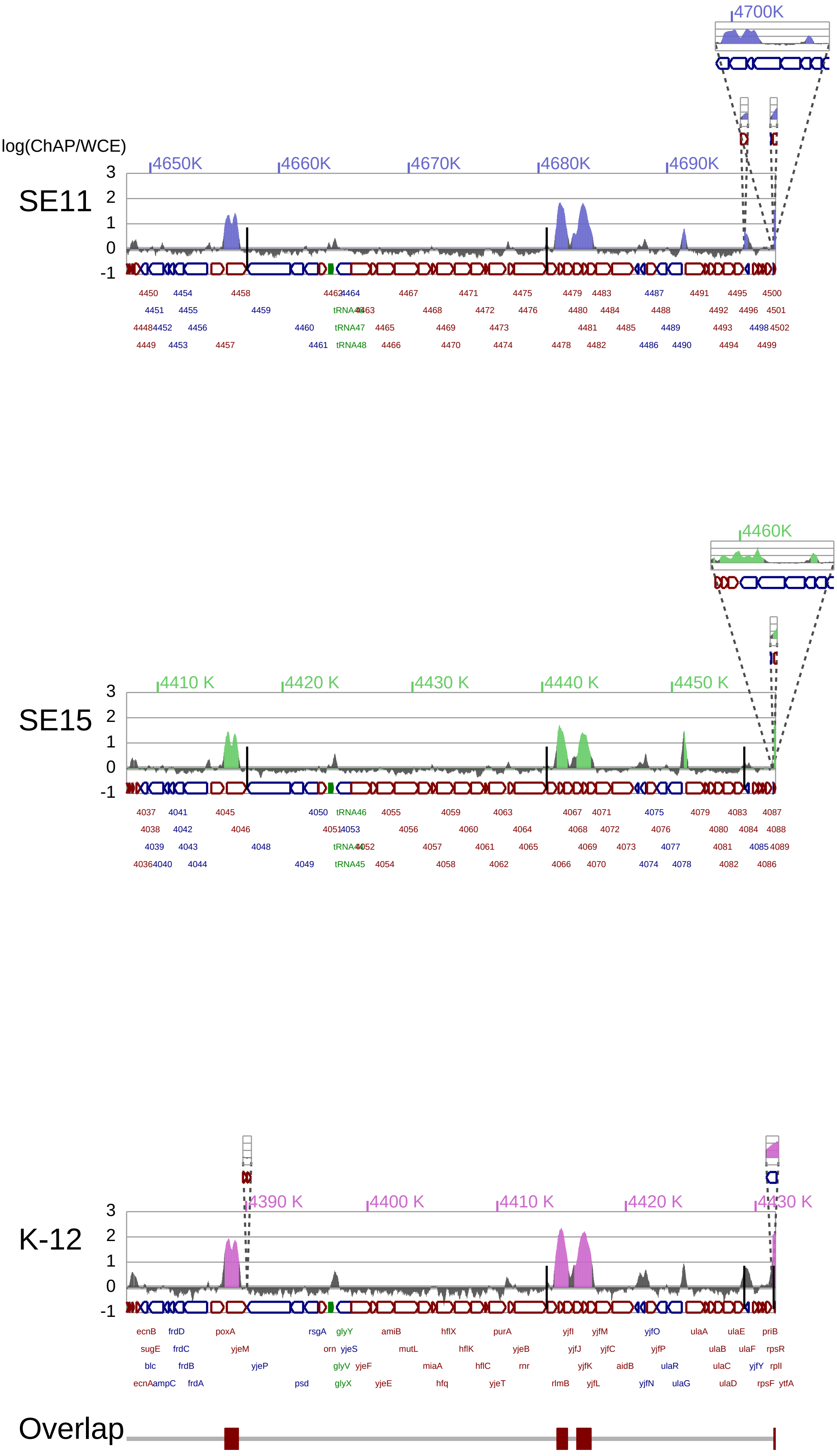
log(ChAP/WCE)
SE11
4650K
4660K
4670K
4680K
4690K
4700K
3
2
1
0
-1
4450 4454 4458 4462 4464 4467 4471 4475 4479 4483 4487 4491 4495 4500
4451 4455 4459 tRNA46 4463 4468 4472 4476 4480 4484 4488 4492 4496 4501
4448 4452 4456 4460 tRNA47 4465 4469 4473 4481 4485 4489 4493 4498 4502
4449 4453 4457 4461 tRNA48 4466 4470 4474 4478 4482 4486 4490 4494 4499
SE15
4410 K
4420 K
4430 K
4440 K
4450 K
4460K
4037 4041 4045 4050 tRNA46 4055 4059 4063 4067 4071 4075 4079 4083 4087
4038 4042 4046 4051 4053 4056 4060 4064 4068 4072 4076 4080 4084 4088
4039 4043 4048 tRNA44 4052 4057 4061 4065 4069 4073 4077 4081 4085 4089
4036 4040 4044 4049 tRNA45 4054 4058 4062 4066 4070 4074 4078 4082 4086
K-12
4390 K
4400 K
4410 K
4420 K
4430 K
ecnB frdD poxA rsgA glyY amiB hflX purA yjfI yjfM yjfO ulaA ulaE priB
sugE frdC yjeM orn yjeS mutL hflK yjeB yjfJ yjfC yjfP ulaB ulaF rpsR
blc frdB yjeP glyV yjeF miaA hflC rnr yjfK aidB ulaR ulaC yjfY rplI
ecnA ampC frdA psd glyX yjeE hfq yjeT rlmB yjfL yjfN ulaG ulaD rpsF ytfA
Overlap

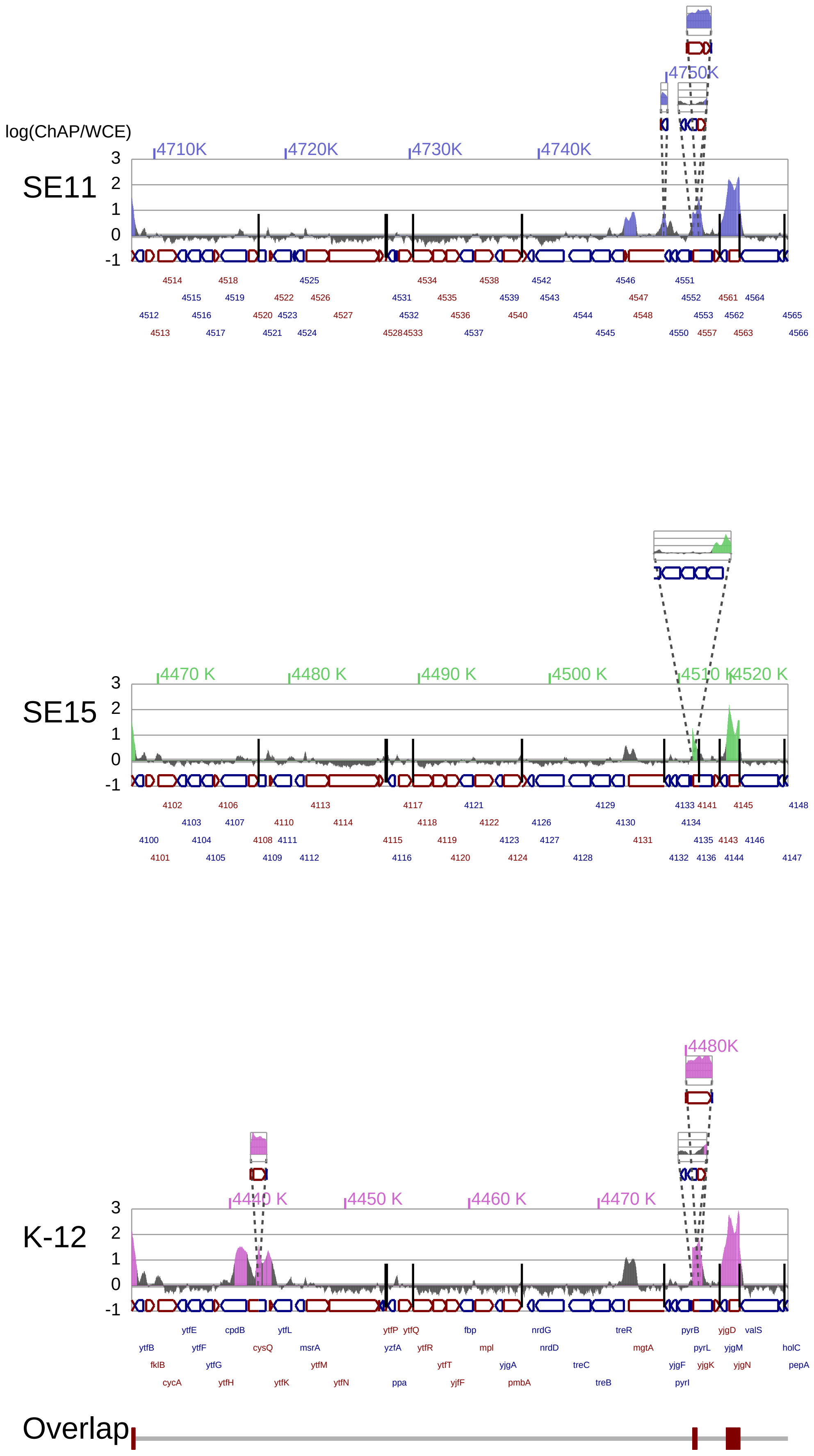

log(ChAP/WCE)
SE11
4710K
4720K
4730K
4740K
4750K
SE15
4470 K
4480 K
4490 K
4500 K
4510 K
4520 K
K-12
4440 K
4450 K
4460 K
4470 K
4480K
ytfB fklB cycA ytfE ytfF ytfG ytfH cpdB cysQ ytfL msrA ytfK ytfM ytfN ytfP ytfQ yzfA ytfR ppa ytfT yjfF fbp mpl yjgA pmbA nrdG nrdD treC treB treR mgtA pyrB pyrL yjgF pyrI yjgK yjgD yjgM yjgN valS holC pepA
Overlap

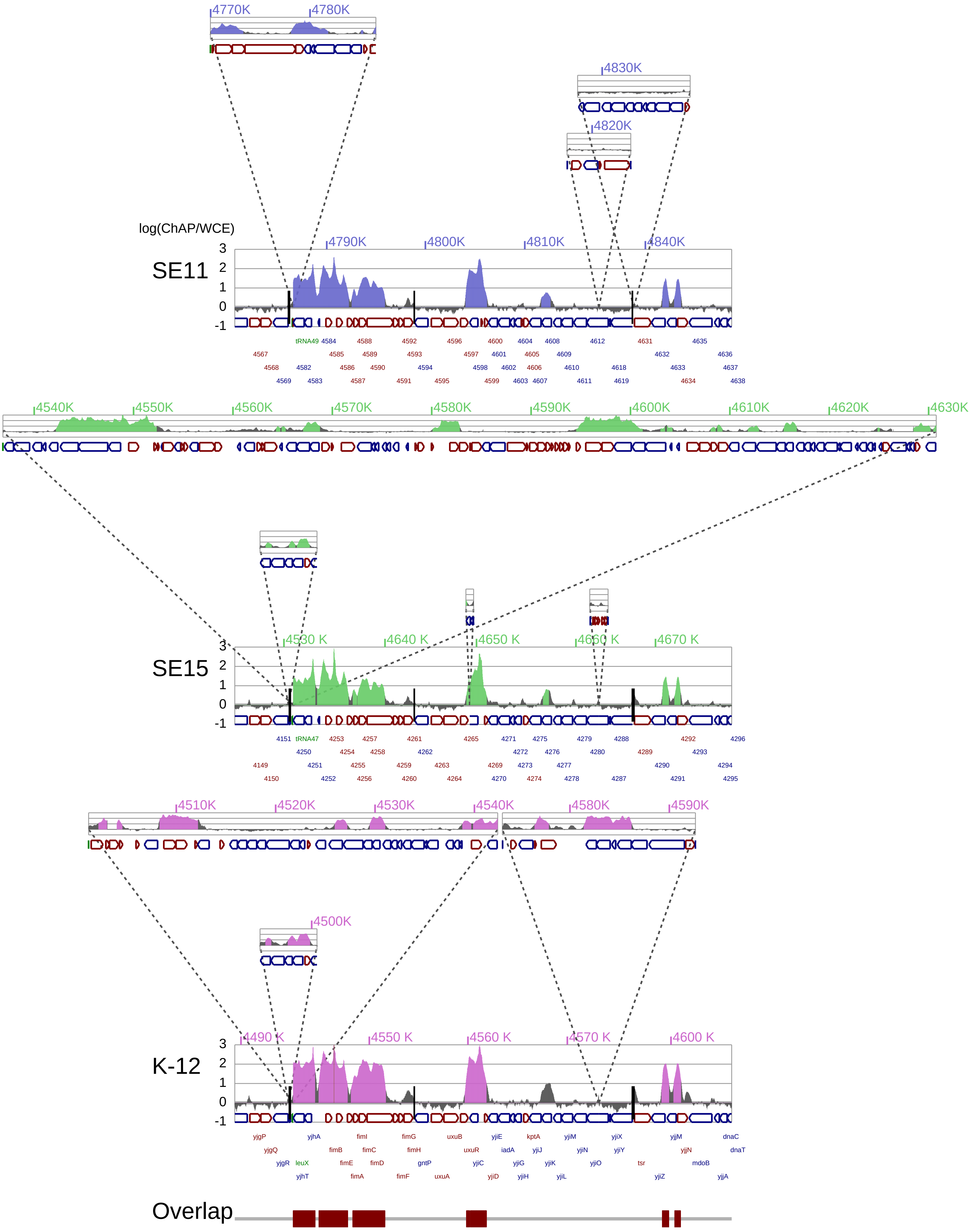
4770K
4780K
4830K
4820K
log(ChAP/WCE)
SE11
4790K
4800K
4810K
4840K
3
2
1
0
-1
tRNA49 4584 4588 4592 4596 4600 4604 4608 4612 4631 4635
4567 4585 4589 4593 4597 4601 4605 4609 4632 4636
4568 4582 4586 4590 4594 4598 4602 4606 4610 4618 4633 4637
4569 4583 4587 4591 4595 4599 4603 4607 4611 4619 4634 4638
4540K
4550K
4560K
4570K
4580K
4590K
4600K
4610K
4620K
4630K
SE15
4530 K
4640 K
4650 K
4660 K
4670 K
4151 tRNA47 4253 4257 4261 4265 4271 4275 4279 4288 4292 4296
4250 4254 4258 4262 4272 4276 4280 4289 4293
4149 4251 4255 4259 4263 4269 4273 4277 4290 4294
4150 4252 4256 4260 4264 4270 4274 4278 4287 4291 4295
4510K
4520K
4530K
4540K
4580K
4590K
4500K
K-12
4490 K
4550 K
4560 K
4570 K
4600 K
yjgP yjhA fimI fimG uxuB yjiE kptA yjiM yjiX yjjM dnaC
yjgQ fimB fimC fimH uxuR iadA yjiJ yjiN yjiY yjjN dnaT
yjgR leuX fimE fimD gntP yjiC yjiG yjiK yjiO tsr mdoB
yjhT fimA fimF uxuA yjiD yjiH yjiL yjiZ yjjA
Overlap

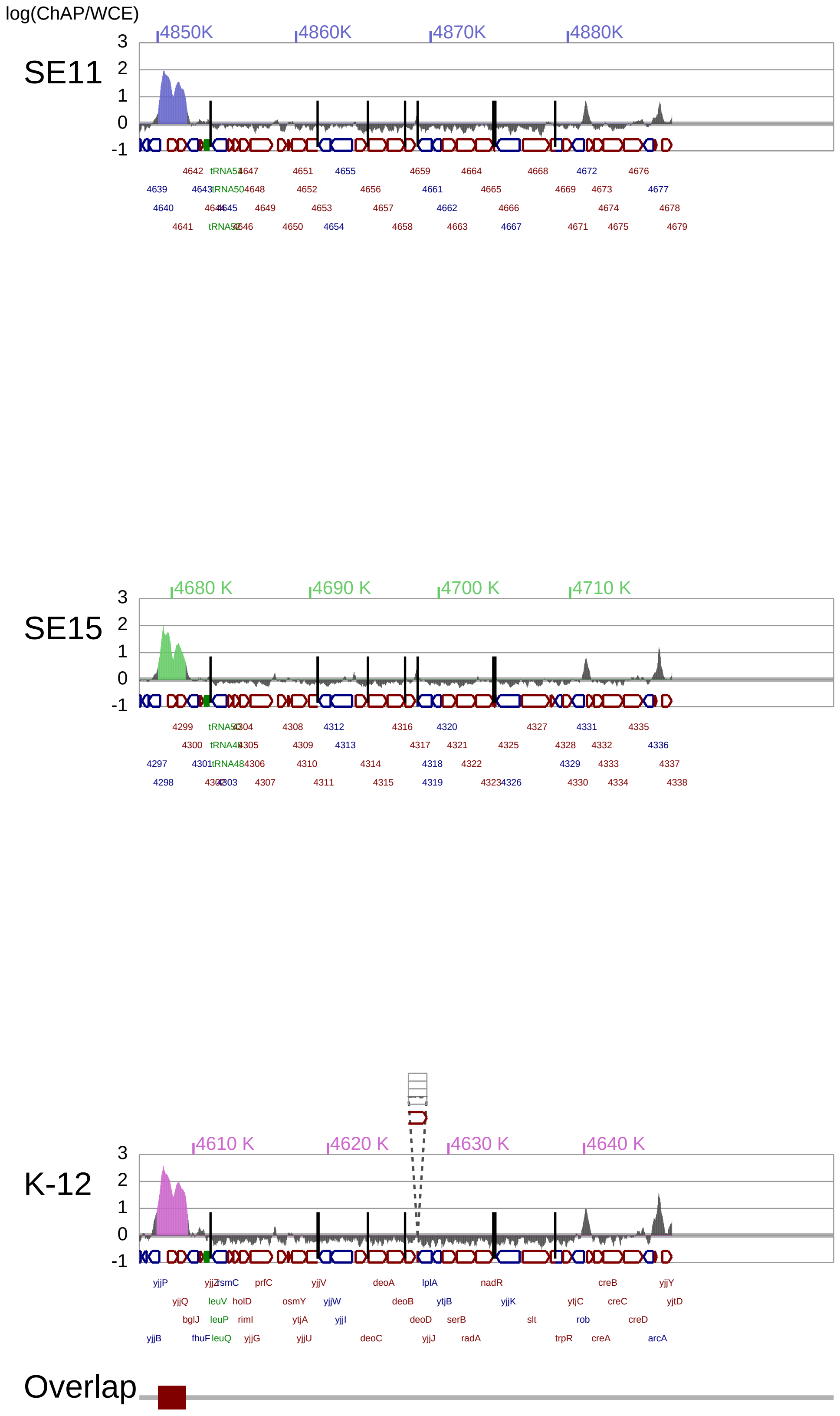
log(ChAP/WCE)
SE11
4850K
4860K
4870K
4880K
SE15
4680 K
4690 K
4700 K
4710 K
K-12
4610 K
4620 K
4630 K
4640 K
3
2
1
0
-1
yjjP
yjjQ
bglJ
yjjB
fhuF
leuV
leuP
leuQ
holD
rimI
yjjG
prfC
osmY
ytjA
yjjU
yjjV
yjjW
yjjI
deoC
deoA
deoB
deoD
lplA
ytjB
serB
yjjJ
radA
nadR
yjjK
slt
trpR
ytjC
rob
creA
creB
creC
creD
arcA
yjjY
yjtD
Overlap
